# Morphology and ultrastructure of Tilioideae pollen: how to differentiate *Craigia*, *Mortoniodendron*, and *Tilia*

**DOI:** 10.1186/s40529-025-00463-1

**Published:** 2025-06-09

**Authors:** Christian Geier, Silvia Ulrich, Johannes M. Bouchal, Reinhard Zetter, Valérie Ngô Muller, Bonnie F. Jacobs, Dieter Uhl, Friðgeir Grímsson

**Affiliations:** 1 Department of Botany and Biodiversity Research, University of Vienna, 1030 Vienna, Austria; 2 Department of Historical Archaeology, Austrian Academy of Sciences (OeAW), Austrian Archaeological Institute (OeAI), 1010 Vienna, Austria; 3 AZ Pollen Research (AZPR), Stockerau, Austria; 4 Muséum National d’Histoire Naturelle, Institut de Systématique Évolution Biodiversité (ISYEB, UMR 7205 CNRS, MNHN, UPMC, EPHE), Sorbonne Universités, Herbier National, 57 Rue Cuvier, CP39 – 75005 Paris, France; 5 Life Sciences Department, Paris-Cité University, 5, rue Thomas Mann, 75013 Paris, France; 6 Roy M. Huffington Department of Earth Sciences, Southern Methodist University, Dallas, TX 75275 USA; 7 Senckenberg Forschungsinstitut und Naturmuseum Frankfurt, 60325 Frankfurt am Main, Germany

**Keywords:** Brevicolporate, Columellae, Costae, Nexine thickening, Palynotaxonomy, Pollen wall, Tectum, Transmission electron microscopy

## Abstract

Assigning fossil Tilioideae pollen grains to the extant genera *Craigia*, *Mortoniodendron*, and *Tilia* is difficult. Firstly, the pollen from these genera appears similar when studied only with light microscopy. Secondly, comparison data showing the morphological and ultrastructural range of *Mortoniodendron* pollen need to be better defined, and there is a lack of transmission electron micrographs of *Craigia* pollen. Cenozoic palynorecords from Europe, especially of Paleogene age, document several Tilioideae-type pollen of taxonomic uncertainty. It is unclear if they represent extant Tilioideae genera, extinct taxa within the subfamily, or other closely related fossil or living Malvaceae. To assist in future taxonomic assignments of fossil Tilioideae-type pollen, we investigated extant *Craigia*, *Mortoniodendron*, and *Tilia* pollen with light, scanning electron, and transmission electron microscopy. New pollen data are provided for all three genera, and previous research was compiled to provide the most up-to-date comprehensive morphological and ultrastructural summary of these genera. Our study shows that combining light- and electron microscopy makes it possible to distinguish among *Craigia*, *Mortoniodendron*, and *Tilia* pollen. This will help paleopalynologists to identify these genera in pollen assemblages or to exclude them and focus on other taxa. The correct assignment of Tilioideae pollen is important for unravelling the paleophytogeographic history of this subfamily and closely related Malvaceae. Also, Tilioideae genera are typical for particular climates and/or vegetation biomes. Therefore, assigning fossil pollen to any of the three extant genera will affect the paleovegetation reconstruction and paleoclimate evaluation.

**Graphical Abstract:**

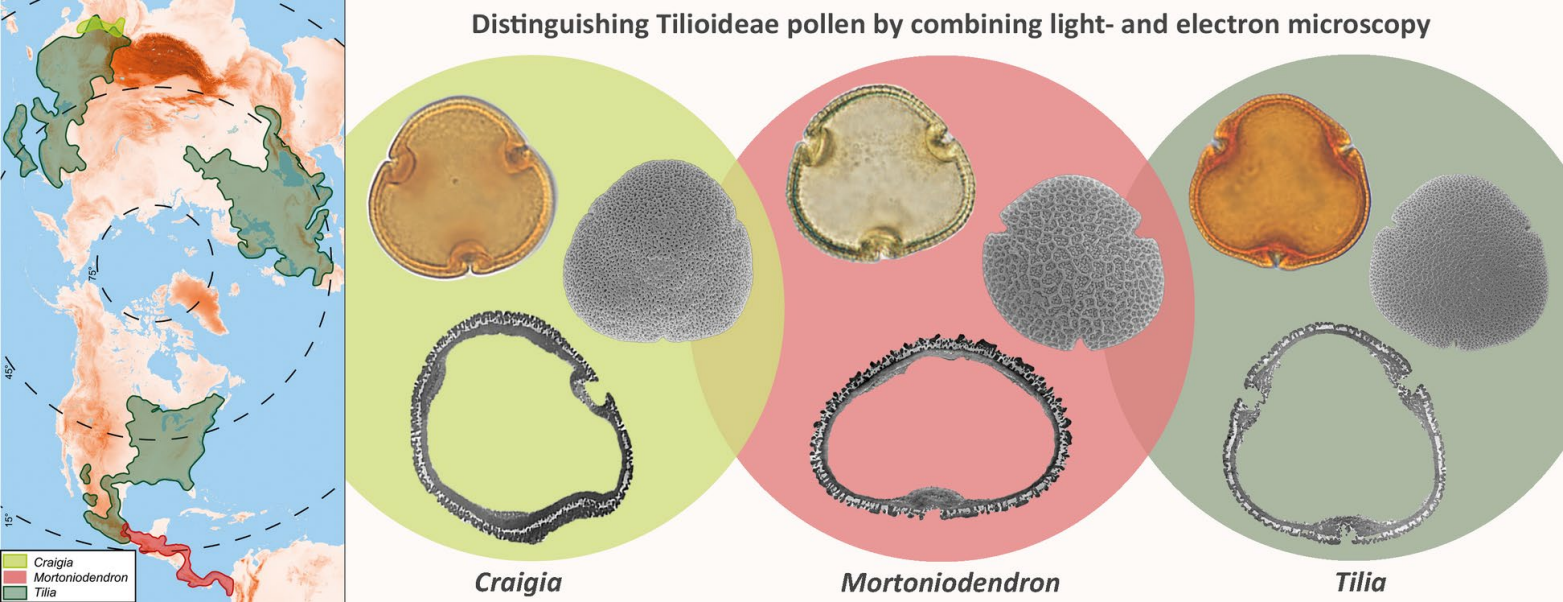

**Supplementary Information:**

The online version contains supplementary material available at 10.1186/s40529-025-00463-1.

## Introduction

The Tilioideae Arnott is a small northern hemispheric subfamily within the Malvaceae *s. lat.* consisting of about 50 species in three genera: *Craigia* W.W.Sm. & W.E.Evans, *Mortoniodendron* Standl. & Steyerm., and *Tilia* L. The genus *Craigia*, comprising only two extant species, *C. yunnanensis* W.W.Sm. & W.E.Evans and *C. kwangsiensis* H.H.Hsue, is native to subtropical southern China and Vietnam (Pigott 2012; POWO 2024). *Mortoniodendron* comprises 16 extant species occurring in tropical Central to South America in the region between southern Mexico and Colombia (POWO 2024). *Tilia* is the most species-rich and widespread of the three Tilioideae genera, with 32 extant species and a distribution extending more or less across the entire temperate Northern Hemisphere. The diversity centers of *Tilia* occur in southeast Asia, western Eurasia, and eastern to south North America (Pigott 2012; POWO 2024). In its current circumscription, Tilioideae represents a monophyletic clade within the Malvaceae *s.lat.*, that diverged from the remaining family in the Late Cretaceous, ca. 79 Ma (85.6–72.6 Ma) and then further diversified ca. 73 Ma (80.4–65.4 Ma) (Hernández-Gutiérrez and Magallón 2019). The oldest fossil Tilioidea-type pollen grains are from the Paleocene (*e.g*., Mai 1961; Muller 1981; Krutzsch 2004 and references therein). Many of the Paleogene Tilioideae pollen records, based on light microscopy (LM) only, have been assigned to *Tilia*. This has been criticised by Manchester (1994), who pointed out similarities between *Tilia* pollen and that from closely related genera such as *Craigia* and *Mortoniodendron*. Manchester further stated that, “*reports based on pollen that are not documented by electron microscopy or corroborated by megafossil evidence should be viewed with caution*” (Manchester 1994 p. 1176). Relying on scanning electron microscopy (SEM), a few recently published records now indicate the potential presence of *Mortoniodendron* in the Paleogene of Europe (Grímsson et al. 2017; Bouchal et al. 2025) and even Asia (Hofmann et al. 2019). There seem to be few reliable Paleogene records of *Craigia* pollen (Bouchal et al. 2025), but combined LM and SEM, and even transmission electron microscopy (TEM) micrographs verify the presence of this genus in the Miocene of Europe (*e.g*., Kvaček et al. 2002; Zetter et al. 2002; Stuchlik et al. 2014; Vieira et al. 2018; Vieira and Zetter 2020). Still, because of 1) the overlapping morphology between Tilioideae pollen when observed with LM only, 2) the current lack of data on the pollen morphology and ultrastructure of different *Mortoniodendron* species, 3) limited TEM investigations available on *Tilia* pollen, and 4) the absence of TEM micrographs showing recent *Craigia* pollen, it is hard to segregate between fossil pollen of these three genera reliably. As mentioned before, the geographic distributions of Tilioideae genera vary significantly, with each genus today being characteristic of a particular climate (temperature, precipitation) and biome range. The ability to distinguish between fossil pollen from *Craigia*, *Mortoniodendron*, and *Tilia* is therefore important to resolve both the origin and dispersal histories of the three genera and for paleoecological estimations. Furthermore, it will enable assigning parent plants of fossil Tilioideae pollen to applicable vegetation units, when reconstructing paleovegetation, and to estimate the appropriate paleoclimate based on present day ecological aspects of Tilioideae species.

To test if it is possible to differentiate pollen from the three extant Tilioideae genera, we compiled the current palynological knowledge (LM, SEM, and TEM based descriptions and micrographs) on *Craigia*, *Mortoniodendron*, and *Tilia* (Chambers and Godwin 1971; (IBSCIB-CAS) 1982; Zhang and Chen 1984; Huo et al. 1985; Reille 1992; Kvaček et al. 2002; Beug 2004; Perveen et al. 2004; Li et al. 2010; Miyoshi et al. 2011; Ishiki and Wendt 2014; Sam and Auer 2021; Auer et al. 2024; Geier et al. 2024a, b, c, d, e, f; Halbritter et al. 2024a, b; Sam et al. 2024; Stebler 2024). We also investigated several *Mortoniodendron* species for the first time, providing a comprehensive pollen morphology and ultrastructure dataset. Pollen from several *Tilia* species were also investigated in more detail, especially with TEM, and *Craigia* pollen was also studied using combined LM, SEM, and TEM. Based on this, we compare pollen grains within and between the three genera, point out both similarities and differences of pollen features between taxa, and reveal how to distinguish *Craigia*, *Mortoniodendron*, and *Tilia* pollen using LM and/or SEM and/or TEM. In addition, we summarize the main climate preferences of the three extant Tilioideae genera. This is to underscore the importance of correctly assigning fossil Tilioideae pollen when reconstructing paleovegetation or evaluating paleoclimate. We also test the applicability of the dataset provided herein on five fossil European Tilioideae pollen types, four from the Eocene and the other from the Miocene. Finally, a few suggestions are provided for the re-investigation of some Paleogene Tilioideae-type pollen records where comparison to our results could be used to verify/reject/adjust previous assignments.

## Material and methods

### Origin of modern plant material

For this investigation, we used dry herbarium samples provided by the herbariums of the Muséum National d’Historie Naturelle (P), Paris, France, the Missouri Botanical Garden (MO), St. Louis, Missouri, United States of America, and the University of Vienna (WU), Vienna, Austria. In total, we investigated 23 herbarium specimens palynologically. We investigated one species of *Craigia*, eight of *Mortoniodendron*, and ten of *Tilia*. The specimens investigated include: *Craigia yunnanensis* (P 06708550, P 06708553), *Mortoniodendron anisophyllum* (MO 4675916, WU 0151992), *M. abelianum* (MO 05015040), *M. apetalum* (MO 6297780), *M. costaricense* (MO 3414249, MO 4294522, MO 6370106,), *M. hirsutum* (MO 6066092), *M. sulcatum* (MO 6709840), *M. uxpanapense* (MO 05027111), *M. vestitum* (MO 6199328), *Tilia americana* (WU 0151993), *T. chinensis* (WU 0151971), *T. cordata* (WU 097095), *T. endochrysea* (WU 0059569), *T. henryana*, *T. japonica* (WU 0151997), *T. miqueliana* (WU 0066018), *T. mongolica* (WU 0151969), *T. platyphyllos* (WU 0151970), *T. tomentosa* (WU 0151994). Additionally, data from the literature were compiled and analysed.

### Origin of fossil plant material

We studied fossil flowers from the Fossillagerstätte Grube Messel (Germany), a Maar Lake structure that has yielded countless fossils over the last 150 years, including amphibian, fish, invertebrate and vertebrate fossils, as well as plant remains like cuticles, flowers, fruits, pollen, seeds, and wood (Smith et al. 2018; Uhl et al. 2024; and references therein). The flowers are housed at the Senckenberg Forschungsinstitut und Naturmuseum Frankfurt and have the collection numbers SMB ME 7412, SMB ME 7415, SMB ME 30688 and SMB ME 31205. They originate from the upper layers of the oil-shale deposits, which are of middle Eocene age (Lutetian; Kaboth-Bahr et al. 2024). The specimens are stored submerged in glycerine to avoid desiccation and destruction and can be accessed through the collection management department.

The Miocene flowers stem from the Inden Formation, accessible in the Hambach Lignite Mine, Lower Rhine Basin, Germany. Some 150 flower buds have been recovered, of which 25 were studied in detail (Kvaček et al. 2002; Zetter et al. 2002). Most of the specimens are part of the private collection of Maria Pingen, while published specimens are stored in the Naturmuseum Augsburg. Micrographs presented herein were taken by one of the co-authors (R.Z.). The TEM micrographs result from a reinvestigation of one of the resin blocks containing embedded anthers of a fossil flower bud (Zetter et al. 2002).

### Sample preparation and pollen documentation

Anthers from herbarium specimens were extracted from flowers or mature flower buds and then acetolysed following the procedure of Erdtman (1960) and Halbritter et al. (2018), with minor changes. Anthers were put in Eppendorf tubes and crushed gently with a glass rod. Fresh acetolysis fluid was poured into the tube. The sample was heated in the acetolysis solution in a water bath at ca. 80° C for 8–10 minutes, followed by several washing steps (pure acetic acid, water, ethanol). Half of the resulting pollen residue was suspended in pure glycerine, while the other half was mixed with glycerine gelatine. One LM slide per sample was prepared without applying a cover slip (unsealed slide), so manipulation (turning) of individual pollen grains was possible. Polar and equatorial views of pollen grains were photographed with LM. The LM slides with the pollen suspended in glycerine gelatine were sealed with a cover slip and nail polish. From the unsealed slides, representative pollen grains of each species were extracted and transferred to SEM stubs, applying the single-grain-method (Zetter 1989). The glycerine was carefully washed away with pure ethanol. Pollen was sputter-coated with gold using a BAL-TEC SCD 050 sputter-coater. To investigate both hemispheres of the same grain, some pollen grains were viewed in the SEM on one side first, then flipped with a micro-manipulator (nasal hair), sputter-coated again, and viewed on the other hemisphere.

The Eocene Tilioideae pollen from the Fossillagerstätte Grube Messel (Germany) was extracted from four fossil compressed flowers (SMB ME 7412, SMB ME 7415, SMB ME 30688, SMB ME 31205) using minute insect pins, followed by processing with bleach and acetolysis fluid (Geier et al. 2023b). The fossil pollen was investigated in LM, SEM, and TEM using the single-grain method (Zetter 1989; Halbritter et al. 2018; Ulrich and Grímsson 2020). Extraction and primary investigation of *Craigia bronii* flower buds is described in Kvaček et al. (2002) and Zetter et al. (2002).

For TEM analysis, acetolysed pollen grains were selected from the glycerine samples and directly embedded in agar-low viscosity resin through an infiltration step using a mixture of resin and acetone, followed by polymerisation at 70° C overnight (Ulrich and Grímsson 2020; Grímsson et al. 2021). Ultra-thin sections (70 nm) were cut using a Leica EM UC6 ultramicrotome and DiATOME Ultra 45° diamond knife. Sections were transferred to Formvar-coated copper/gold grids (Halbritter et al. 2018) and investigated with TEM without further contrast.

Light microscopy micrographs were taken with an Olympus BX53 microscope equipped with an Olympus MPlanApo 60 × /0.90 objective, an Olympus UPlanApo × 100/1.35 oil iris objective, and an Olypmus EP50 camera. SEM micrographs were taken with a JEOL-JSM-6390 at 10 kV, and TEM micrographs were taken with a Zeiss EM 900N at 80 kV, with an integrated digital camera (CCD controller). TEM panorama scans were made at a magnification of 7000 or 12,000 for better overviews, and picture alignment and picture merge were made with an Image SP-Program (ISPViewer64). All micrographs have been edited (brightness, contrast) using Photoshop.

### Quantification of the costae / wall thickening at aperture

**Fig. 1:**
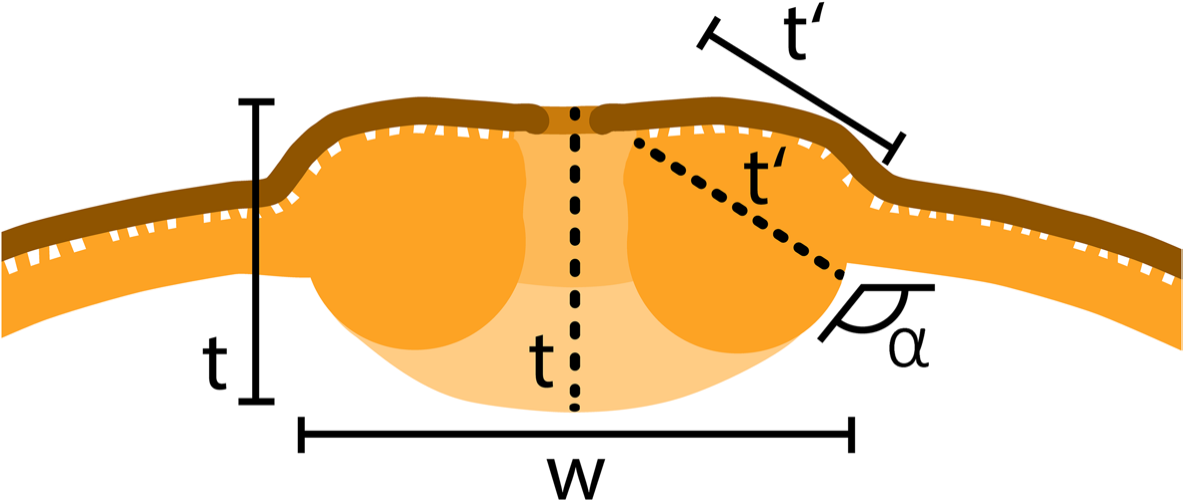
Schematic drawing of an Tilioideae aperture based on acetolysed pollen observed with LM. Thickness of the costae were measured in two places, in centre of the apertural thickening (*t*) and in the lateral part of the thickening (*t’*). w = width of costae, t and t’ = thickness of costae, α = inner angle

The apertures in Tilioideae pollen have characteristic features that need to be documented when differentiating pollen of this subfamily. Here, we used LM micrographs of pollen in polar views to clarify configuration of the costae. As illustrated in Fig. 1, we measured the width (*w*) and thickness (*t*) of the costae. The width equals the distance between the two points where the interapertural walls transition into the aperture complex. The thickness of the costae were measured between the center of the aperture opening and the margin of the aperture thickening (*t*), and from the starting point of the nexine towards the transition point of the interapertural wall and aperture complex (*t’*). The inner angle (α) formed by the aperture thickening was also measured.

### Harvesting, handling, and presentation of climate and biome data

For extant Tilioideae genera georeferenced distribution data were downloaded from GBIF.org (*Craigia*: https://doi.org/10.15468/dl.smspt4; *Mortoniodendron*: https://doi.org/10.15468/dl.xuyb52; *Tilia*: https://doi.org/10.15468/dl.kmnb4p, https://doi.org/10.15468/dl.uuskzp). Outliers and multiple occurrences were omitted from the datasets. The adjusted datasets comprise 18 georeferenced occurrences of *Craigia* (one species), 745 occurrences of *Mortoniodendron* (16 species), and 108.325 occurrences of *Tilia* (20 species) (Supplementary Material 1). The datasets were plotted onto the world biome dataset of Olson et al. (2001), using the “Intersection geoprocessing” tool in Qgis (v.3.34.11-Prizren), to create biome profiles for the investigated taxa. Additionally, the datasets were plotted onto 1 km^2^ grid Köppen-Geiger climate maps (data covering the years 1979–2013, from Cui et al. 2021) to establish Köppen profiles for the species/genera studied. Köppen profiles reflect the proportional Köppen-Geiger climate zone coverage of modern plant species based on their gridded distribution (Supplementary Material 1). The Köppen-Geiger climates occupied by extant *Craigia*, *Mortoniodendron*, and *Tilia* are provided as frequency diagrams (showing proportional distribution) and distribution maps generated and compiled in Qgis (Supplementary Material 2). The georeferenced datasets and the Köppen-Geiger climate maps, with 1 km^2^ resolution, were processed using the ‘Sample Raster Values’ Toolbox in Qgis (v.3.34.11-Prizren). Additionally, climate diagrams covering mean monthly temperatures (MMT), minimum monthly temperatures (MinMT), and the mean monthly precipitation (MMP) for most Tilioideae species are provided (Supplementary Material 2). These were compiled relying on historical climate data from the years 1970–2000, using World Clim v.2.1 (https://www.worldclim.org/data/worldclim21.html) at a resolution of 30s (ca. 1km^2^; Fick and Hijmans 2017; Supplementary Material 2).

## Results

The pollen descriptions of Tilioideae are presented in alphabetical order of the genera.

**Fig. 2:**
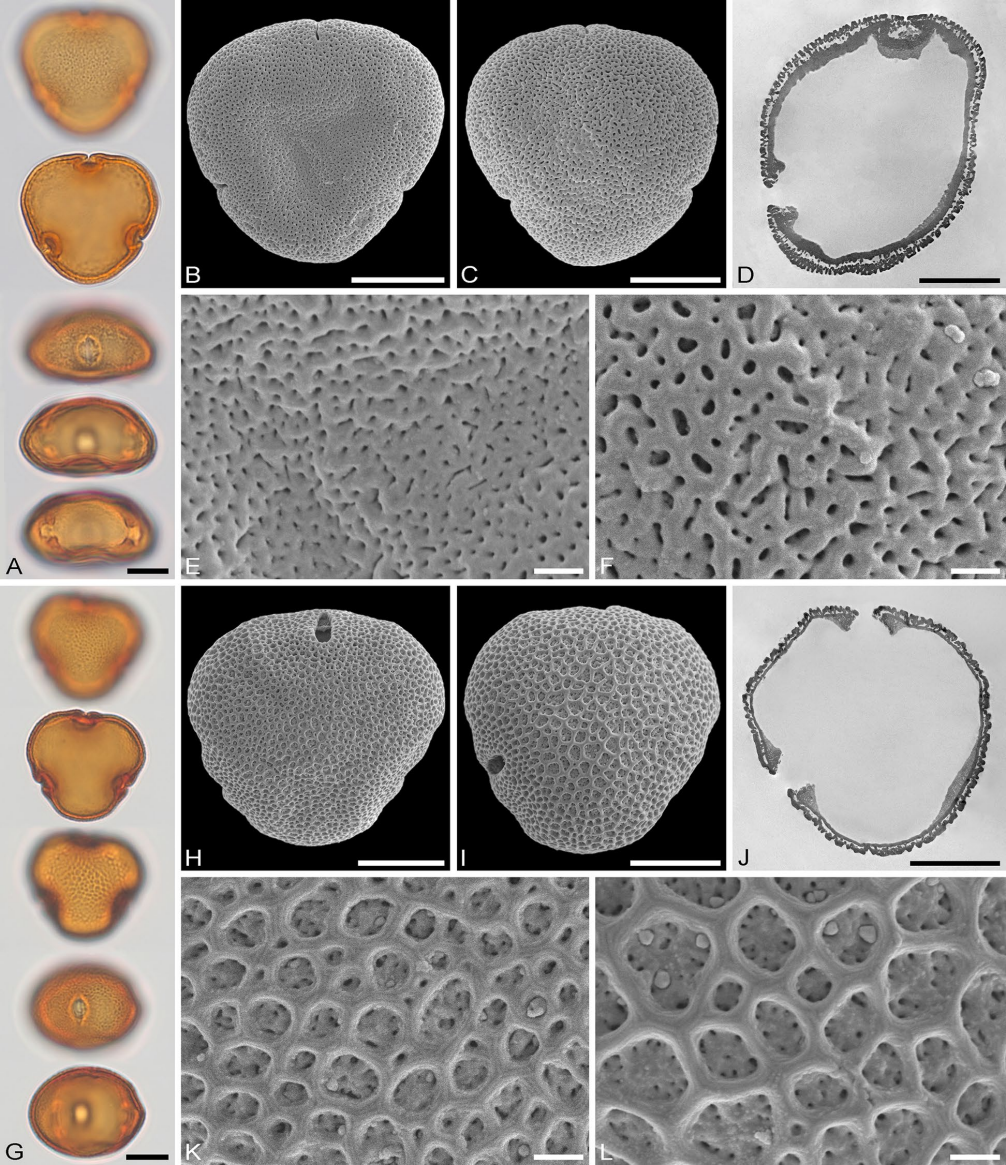
Pollen morphology of *Craigia yunnanensis*. **A**–**F** *C. yunnanensis* P 06708550 (China). **G**–**L** *C. yunnanensis* P 06708553 (Vietnam). **A**, **G** LM micrographs. **B**, **C**, **H**, **I** SEM overview micrographs (**B**, **H** proximal pole; **C**, **I** distal pole). **D**, **J** TEM overview micrographs. **E**, **F**, **K**, **L** close-up SEM micrographs (**E**, **K** proximal pole; **F**, **L** distal pole). Scale bars: 10 µm (**A–D**, **G–J**), 1 µm (**E**, **F**, **K**, **L**)

**Fig. 3:**
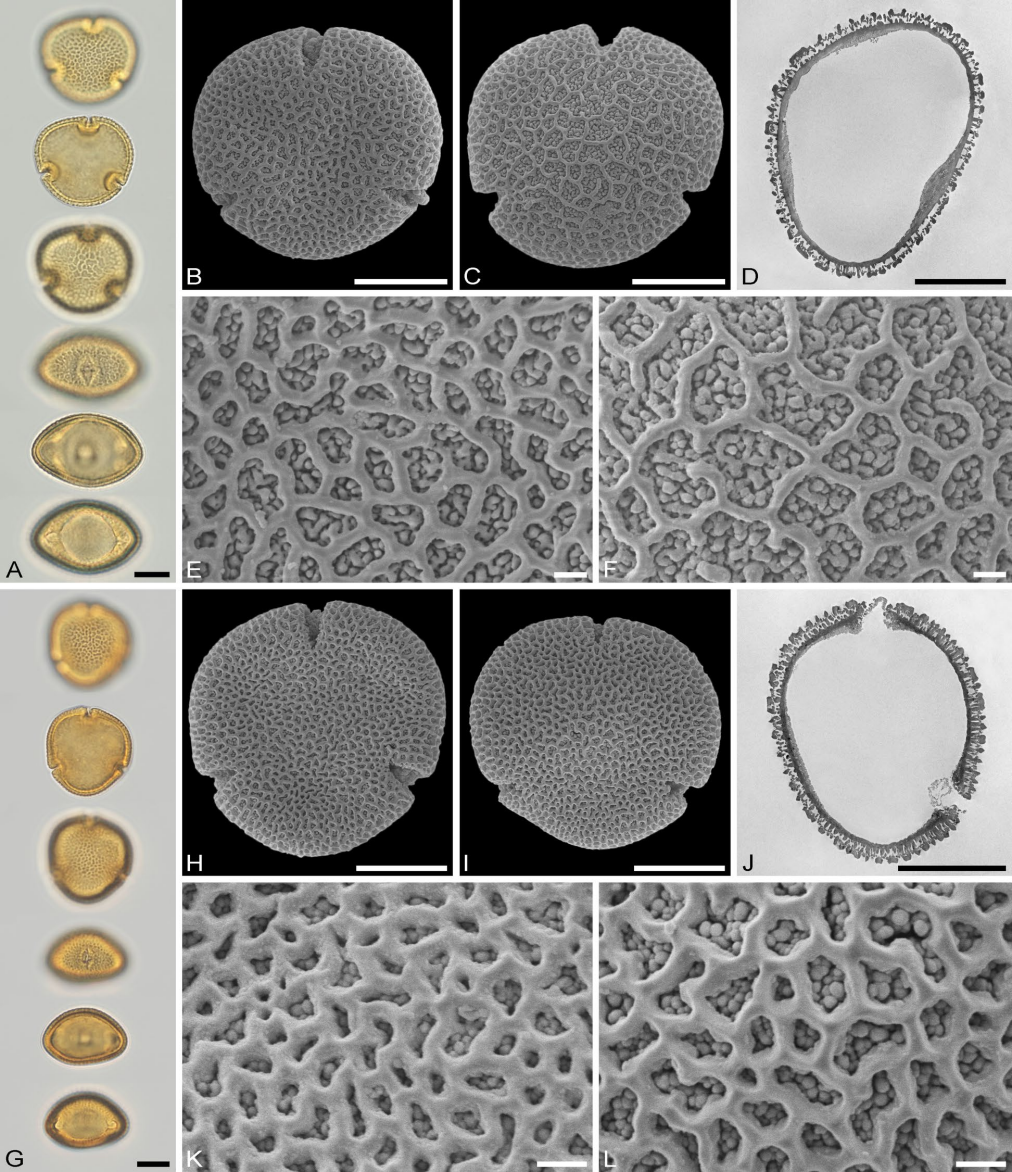
Pollen morphology of *Mortoniodendron*. **A**–**F** *M. costaricense* MO 6370106 (Honduras). **G**–**L** *M. vesititum* MO 6199328 (Mexico). **AbG** LM micrographs. **B**, **C**, **H**, **I** SEM overview micrographs (**B**, **H** proximal pole; **C**, **I** distal pole). **D**, **J** TEM overview micrographs. **E**, **F**, **K**, **L** close-up SEM micrographs (**E, K** proximal pole; **F, L** distal pole). Scale bars: 10 µm (**A–D**, **G–J**), 1 µm (**E**, **F**, **K**, **L**)

**Fig. 4:**
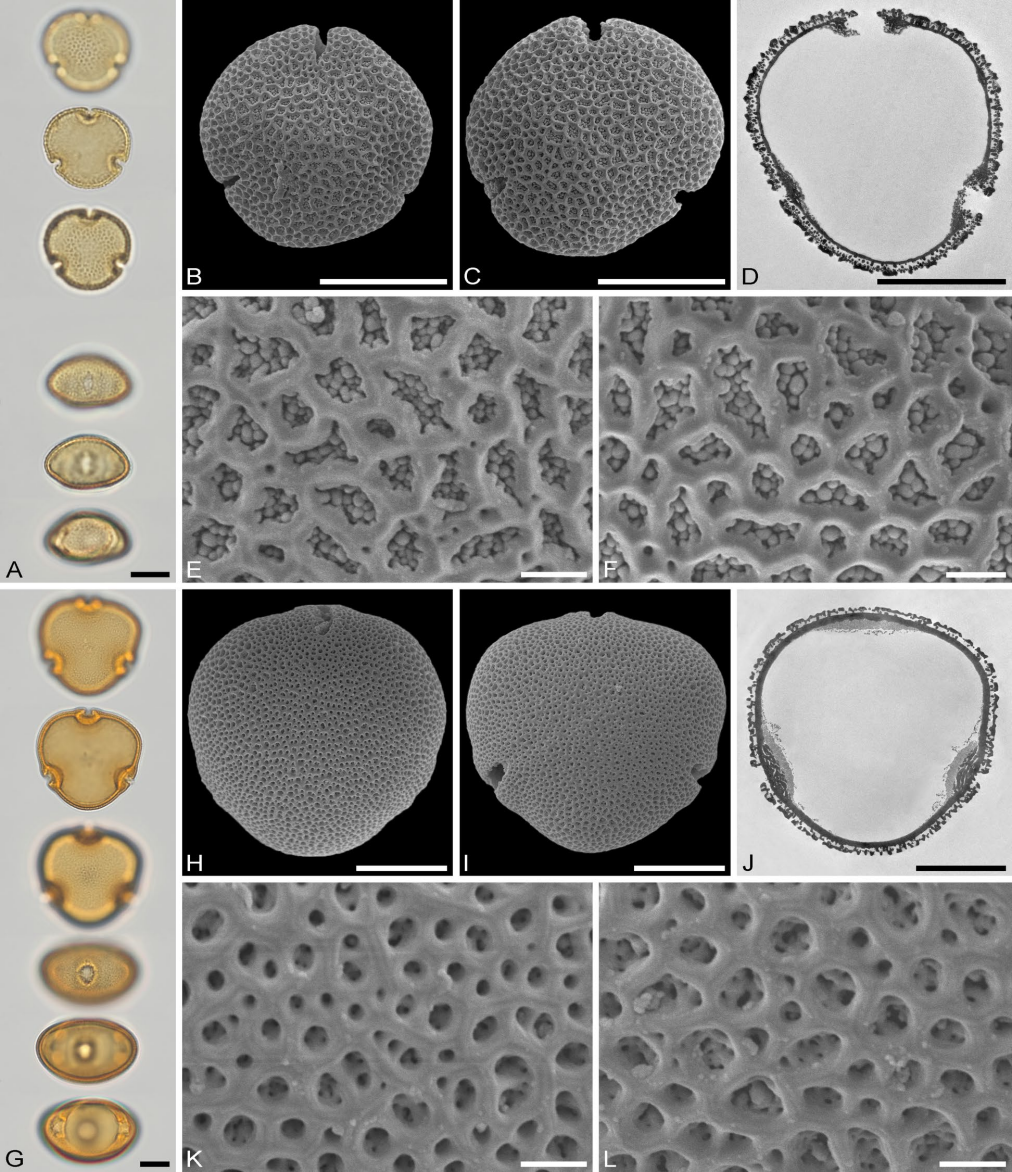
Pollen morphology of *Mortoniodendron* and *Tilia*. **A**–**F** *M. anisophyllum* WU 0151992 (Costa Rica). **G**–**L** *T. endochrysea* WU 0059569 (China). **A**, **G** LM micrographs. **B**, **C**, **H**, **I** SEM overview micrographs (**B**, **H** proximal pole; **C**, **I** distal pole). **D**, **J** TEM overview micrographs. **E**, **F**, **K**, **L** close-up SEM micrographs (**E**, **K** proximal pole; **F**, **L** distal pole). Scale bars: 10 µm (**A**–**D**, **G**–**J**), 1 µm (**E**, **F**, **K**, **L**)

**Fig. 5:**
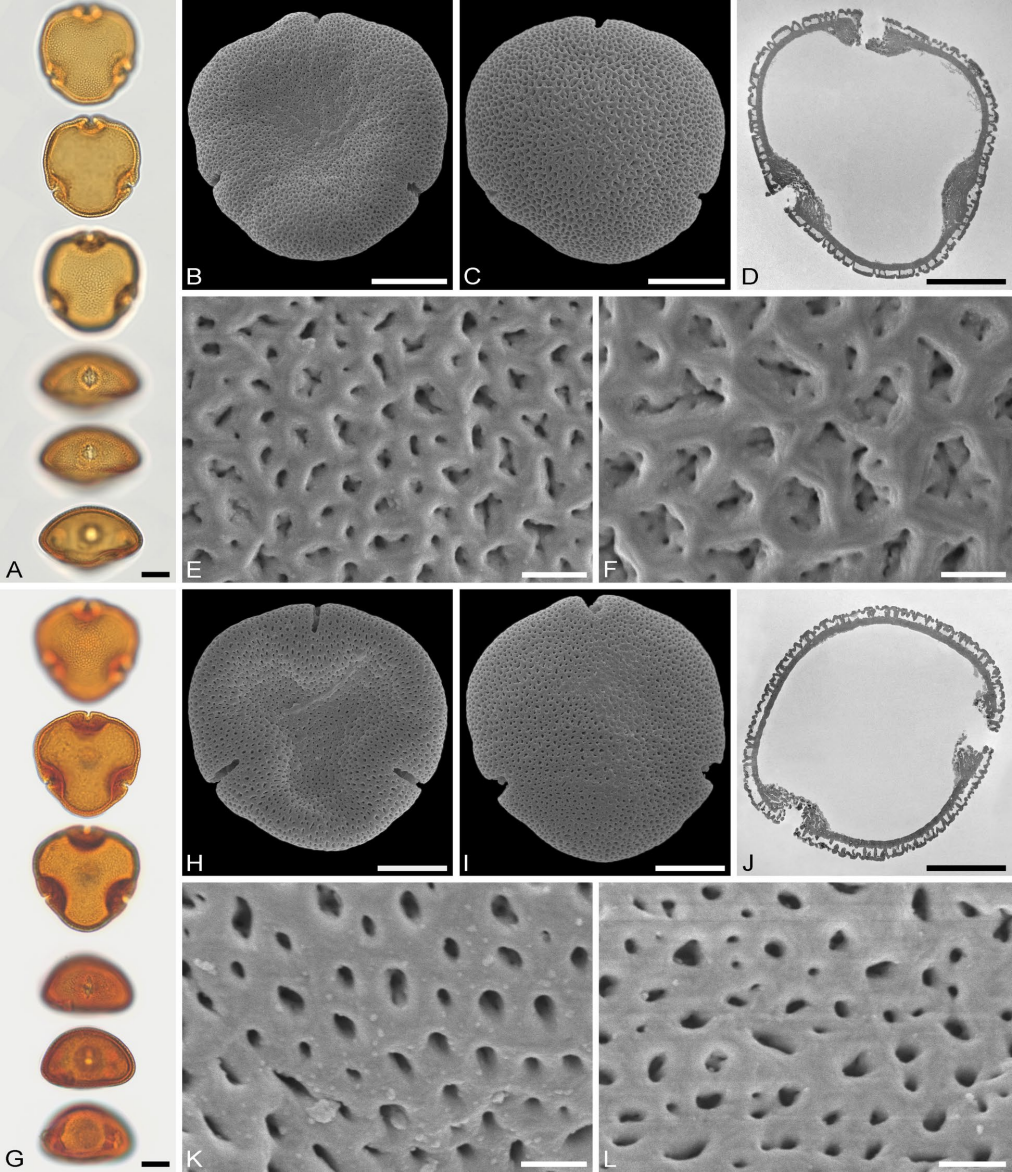
Pollen morphology of *Tilia*. **A**–**F** *T. miqueliana* WU 0066018 (Japan). **G**–**L** *T. mongolica* WU 0151969 (China). **A**, **G** LM micrographs. **B**, **C**, **H**, **I** SEM overview micrographs (**B**, **H** proximal pole; **C, I** distal pole). **D, J** TEM overview micrographs. **E**, **F**, **K**, **L** close-up SEM micrographs (**E**, **K** proximal pole; **F**, **L** distal pole). Scale bars: 10 µm (**A**–**D**, **G**–**J**), 1 µm (**E**, **F**, **K**, **L**)

**Fig. 6:**
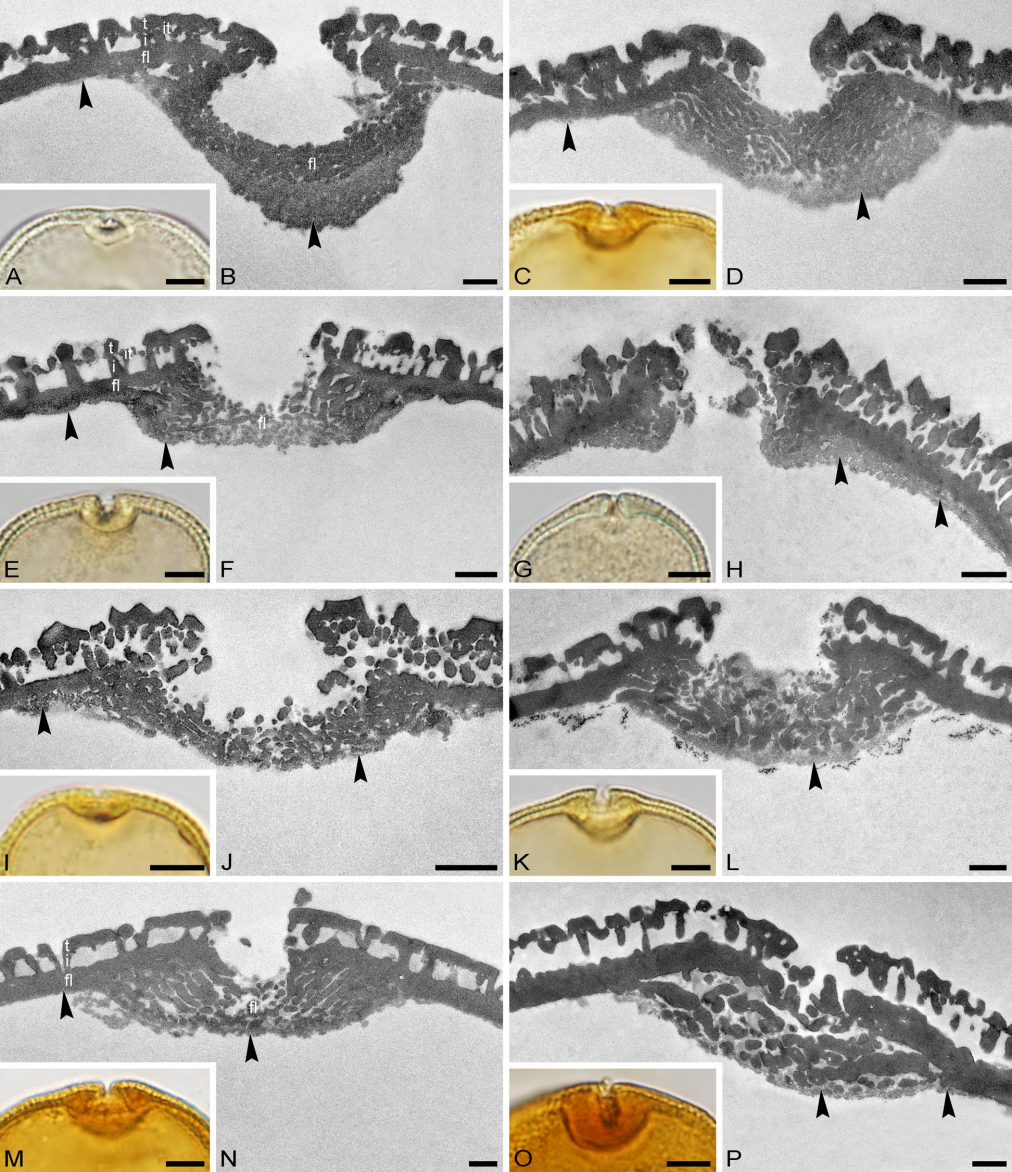
Apertures in Tilioideae pollen. LM micrographs (**A**, **C**, **E**, **G**, **I**, **K**, **M**, **O**), TEM micrographs (**B**, **D**, **F**, **H**, **J**, **L**, **N**, **P**). **A**, **B** *Craigia yunnanensis* P 06708550. **C**, **D** *C. yunnanensis* P 06708553. **E**, **F** *Mortoniodendron costaricense* MO 6370106. **G**, **H** *M. vesititum* MO 6199328. **I**, **J** *M. anisophyllum* WU 0151992. **K**, **L** *Tilia endochrysea* WU 0059569. **M**, **N** *T. miqueliana* WU 0066018. **O**, **P** *T. mongolica* WU 0151969. Black arrowheads indicating thickened endexine. Scale bars: 5 µm (**A**, **C**, **E**, **G**, **I**, **K**, **M**, **O**), 1 µm (**B**, **D**, **F**, **H**, **J**, **L**, **N**, **P**)

**Fig. 7:**
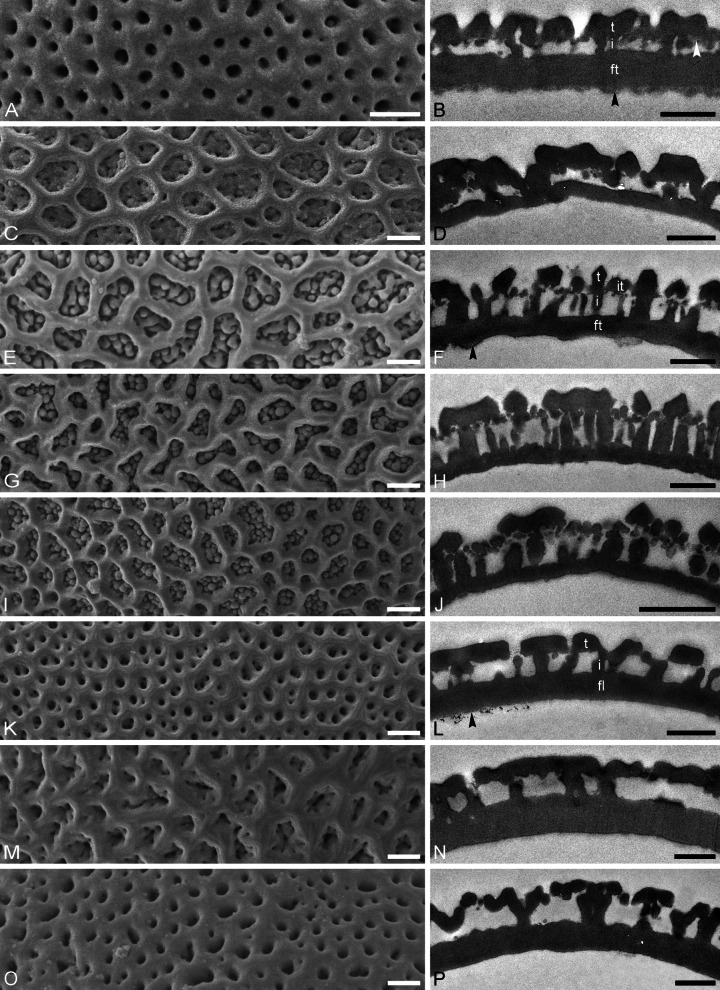
Interapertural wall sections in Tilioideae pollen. SEM micrographs (**A**, **C**, **E**, **G**, **I**, **K**, **M**, **O**), TEM micrographs (**B**, **D**, **F**, **H**, **J**, **L**, **N**, **P**). **A, B** *Craigia yunnanensis* P 06708550. **C**, **D** *C. yunnanensis* P 06708553. **E**, **F** *Mortoniodendron costaricense* MO 6370106. **G**, **H** *M. vesititum* MO 6199328. **I**, **J** *M. anisophyllum* WU 0151992. **K**, **L** *Tilia endochrysea* WU 0059569. **M**, **N** *T. miqueliana* WU 0066018. **O**, **P** *T. mongolica* WU 0151969. t = tectum, it = internal tectum, i = infratectum, fl = foot layer, black arrowhead (**B**, **F**, **L**) indicates endexine, white arrowhead (**B**) indicates indistinct internal tectum. Scale bars: 1 µm (**A**–**P**)

**Fig. 8:**
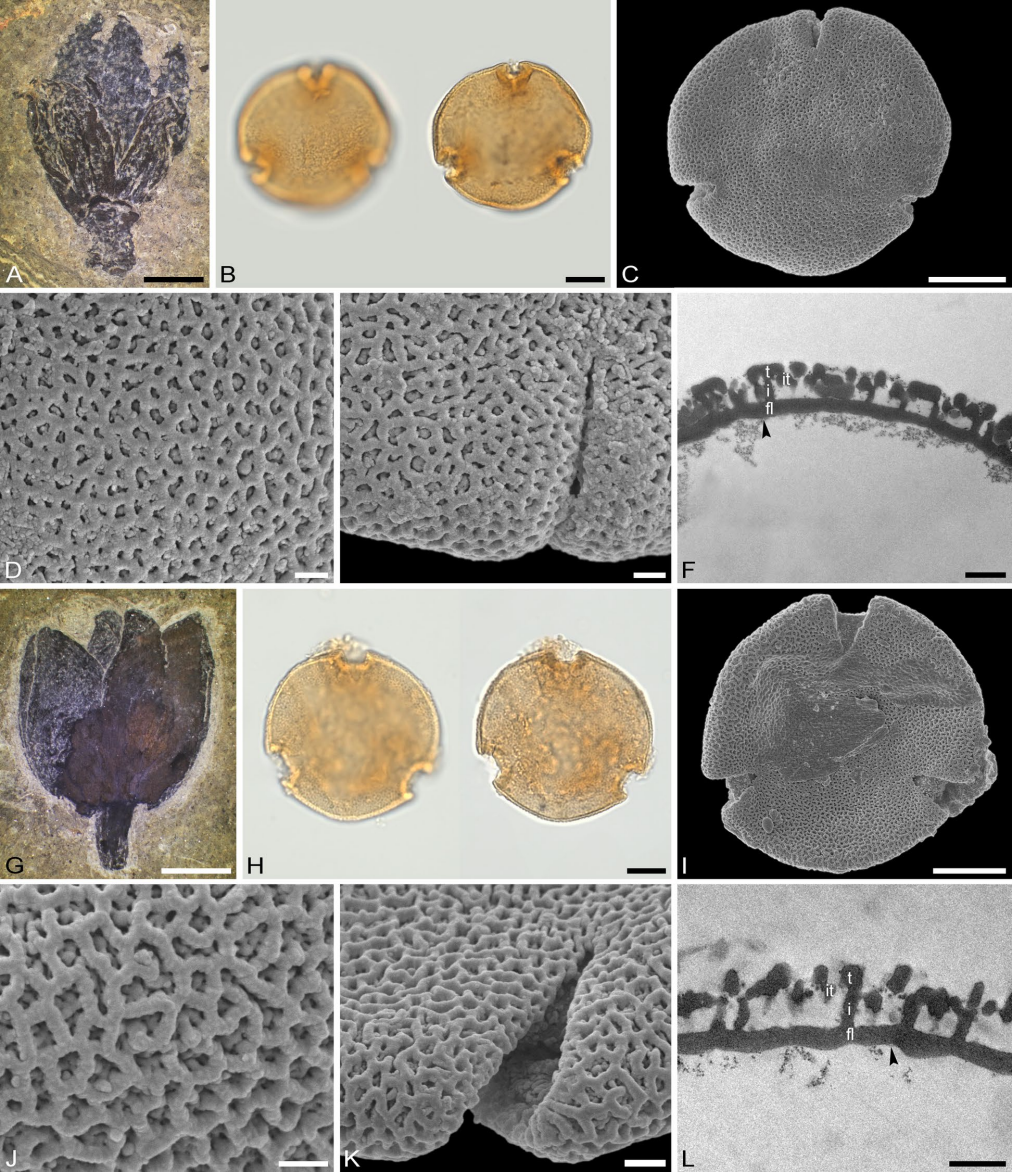
Tilioideae flowers with in-situ pollen from the Eocene of Messel, Germany. Pollen morphology and ultrastructure investigated with LM, SEM, and TEM. **A**–**F** Tilioideae flower A (SMB ME 30688). **G**–**L** Tilioideae flower B (SMB ME 7415). t = tectum, it = internal tectum, i = infratectum, fl = foot layer, black arrowhead indicates endexine. Scale bars: 5 mm (**A**), 1 mm (**G**), 10 µm (**B**, **C**, **H**, **I**), 1 µm (**D**, **E**, **F**, **J**, **K**, **L**)

**Fig. 9:**
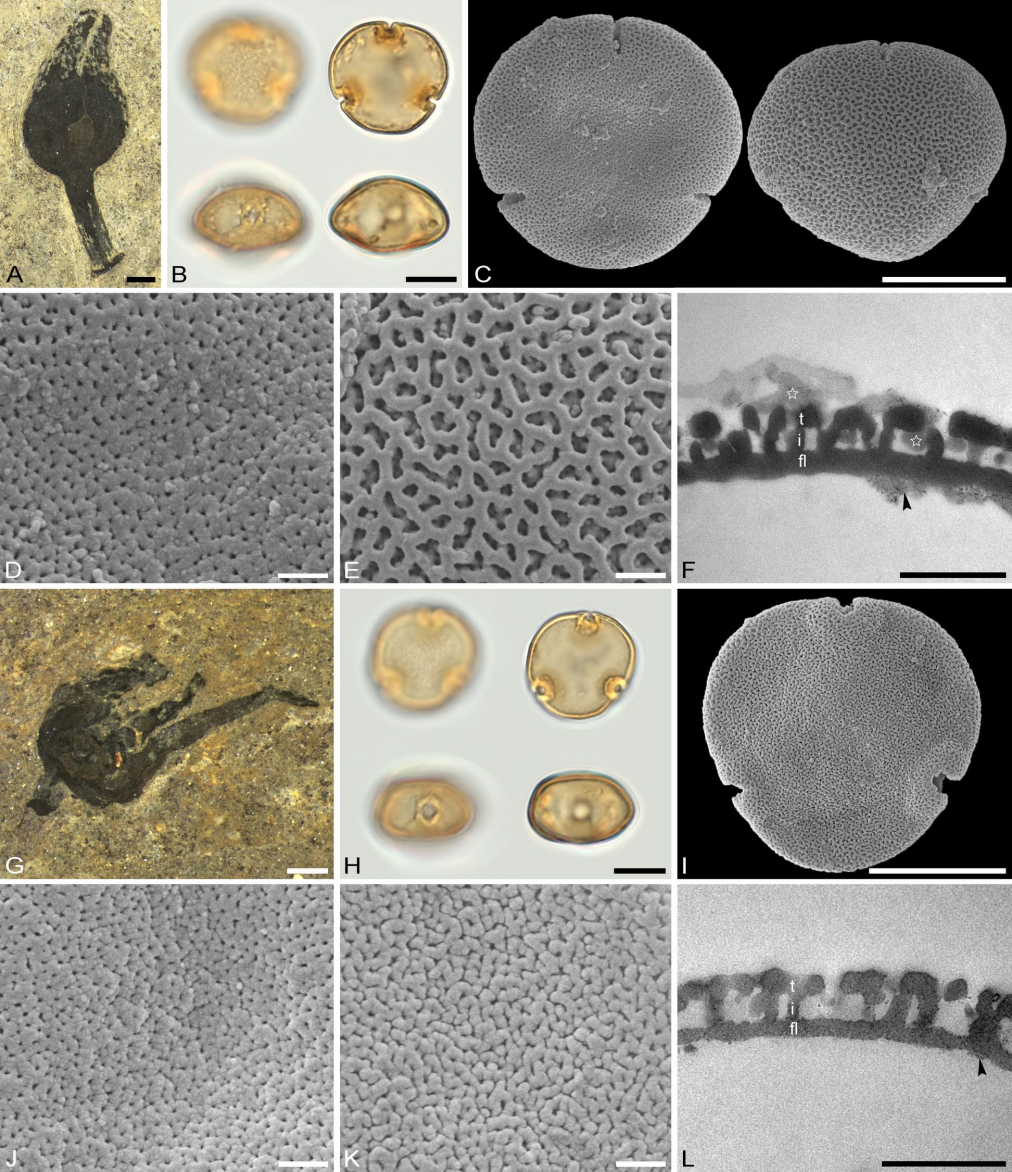
Tilioideae flowers with in-situ pollen from the Eocene of Messel, Germany. Pollen morphology and ultrastructure investigated with LM, SEM, and TEM. **A**–**F** Tilioideae flower C (SMB ME 7412). **G**–**L** Tilioideae flower D (SMB ME 31205). t = tectum, i = infratectum, fl = foot layer, asterisks indicate pollen coatings and black arrowhead indicates endexine. Scale bars: 1 mm (**A**), 10 µm (**B**, **C**, **H**, **I**), 1 µm (**D**, **E**, **F**, **J**, **K**, **L**)

**Fig. 10:**
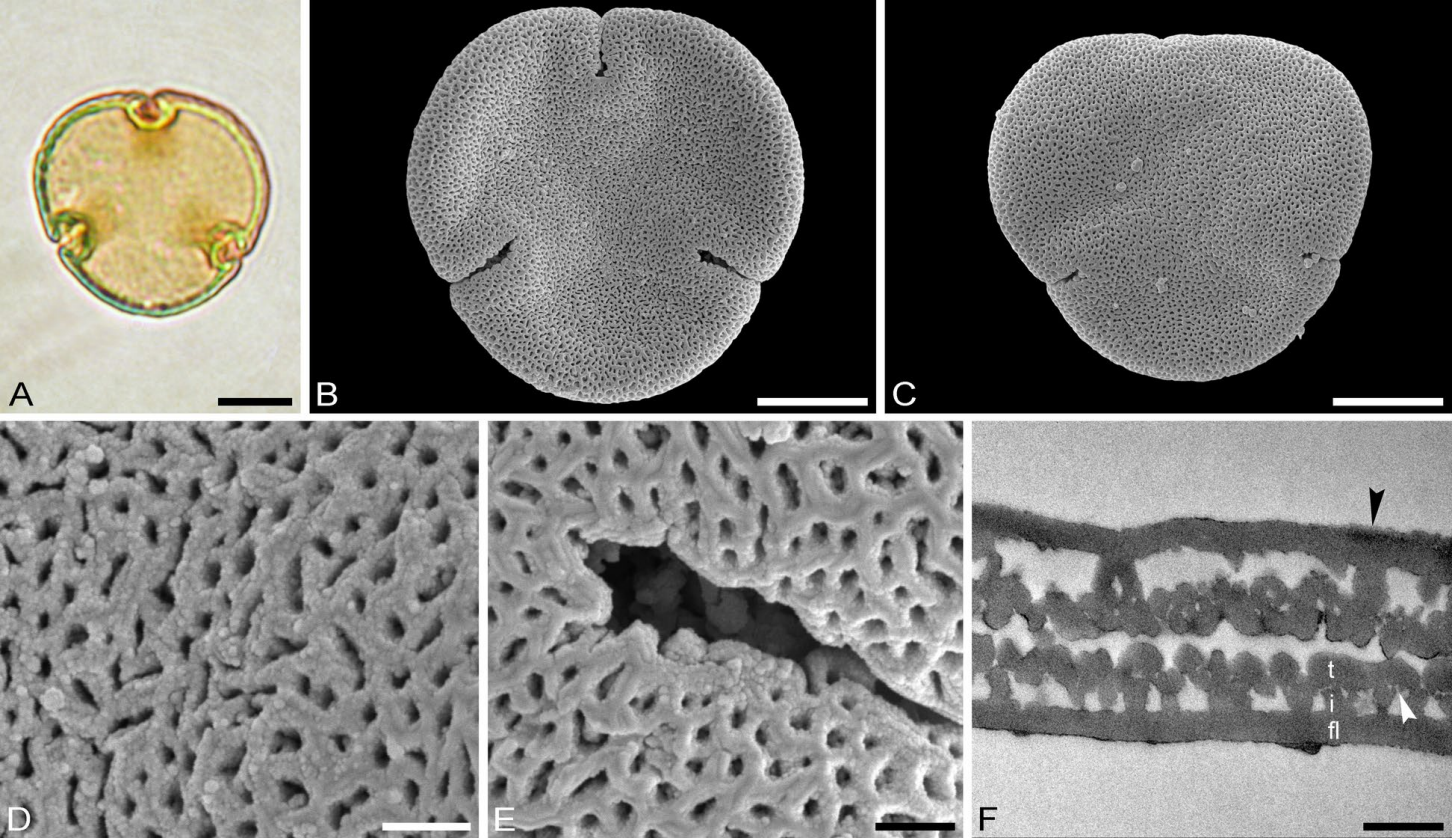
Fossil *Craigia* pollen from the Late Miocene of Hambach, Germany. Partially reinvestigated with combined LM, SEM, and TEM (Kvaček et al. 2002; Zetter et al. 2002). t = tectum, i = infratectum, fl = foot layer, black arrowhead indicates endexine, white arrowhead indicates indistinct internal tectum. Scale bars: 10 µm (**A**–**C**), 1 µm (**D**–**F**)

**Fig. 11:**
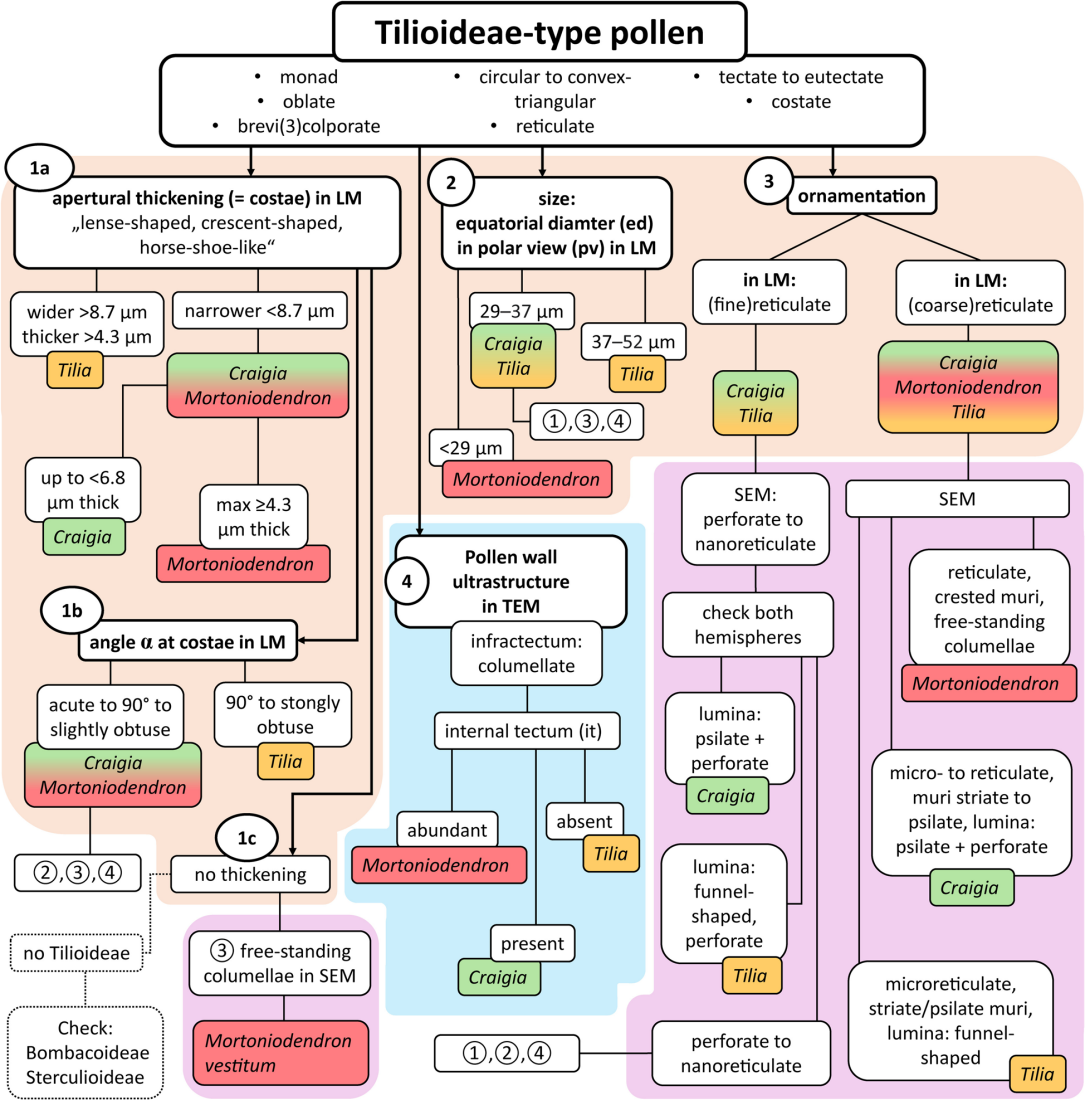
Graphical identification key to the genera of Tilioideae pollen. Abbreviations: ed = equatorial diameter; LM = light microscope; pv = polar view; SEM = scanning electron microscope; TEM = transmission electron microscope. Colour codes of background: light orange: LM data; light magenta: SEM data; light blue: TEM data. Colour codes of species: green: *Craigia*; red: *Mortoniodendron*; yellow: *Tilia*. Dashed lines indicate pollen data not analysed within this work

The descriptions are at generic level, encompassing all relevant morphological and ultrastructural features and their range for pollen from each Tilioideae genus. Exceptions in morphology or ultrastructure are noted separately for the relevant species/taxa. The descriptions are based on new LM, SEM, and TEM data provided herein (Table 1, Figs. 2, 3, 4, 5, 6, 7, 8, 9 and 10, Supplementary Material 3 and 4) as well as compiled from the literature (Supplementary Material 3). Each description covers the main morphological features observed with LM and SEM, followed by ultrastructural traits observed with TEM. The descriptive terminology follows Punt et al. (2007; for LM) and Halbritter et al. (2018; for LM, SEM, and TEM). The results of the investigation allow the differentiation of genera within the Tilioideae (or exclude the subfamily) and are given in text as well as a graphical identification key (Fig. 11).

**Table 1:** Morphology and ultrastructure of Tilioideae pollen

		*Craigia*	*Mortoniodendron*	*Tilia*
General morphology (LM, SEM)	Pollen dispersal unit	Monad	Monad	Monad
	Size class	medium	small/medium	medium
	Polar axis, LM	13–28	13–20	16–37
	Equatorial diameter, LM	29–37	20–33	30–52
	Polar axis, SEM	19–20	11–19	16–26
	Equatorial diameter, SEM	26–31	16–29	27–46
	P/E ratio	Oblate	Oblate	Oblate
	Outline in PV	Convex-triangular/ circular	Circular (convex-triangular)	Convex-triangular/ circular
	Outline in EV	Elliptic, proximal hemispere slightly concave	Elliptic	Elliptic, proximal hemispere slightly concave
	Aperture condition	Brevi(3)colporate	Brevi(3)colporate	Brevi(3)colporate
	Exine thickness LM	1.2–2.2; S = N to S = 2N	1.1–2.2; S ≥ N to S = 2N	1.6–2.2; S = N to S ≥ N
Costae (LM)	Shape	Crescent- to lense-shaped	Crescent- to lense-shaped	Crescent- to lense-shaped, massive; sexine sometimes bulging outward
	Measurmets (thickness [*t, t'*] × width [*w*])	1.5–6.8 × 5.3–8.7	1.3–4.3 × 4.3–8.3	2.1–7 × 8.7–16
	Angle [α]	Acute (rare: obtuse)	90° to slightly obtuse	Obtuse
Ornamentation (LM, SEM)	LM	Reticulate	Reticulate	Reticulate
	SEM	Nanoreticulate to reticulate, perforate, lumen perforate	Nanoreticulate to reticulate, perforate, free-standing columellae in lumen, gemmate	Microreticulate, perforate, lumen irregular in outline, lumen surface perforate
	Lumen diameter, SEM	0.14–1.98	0.36–3.17	0.15–1.72
	Muri width, SEM	0.21–0.50	0.19–0.51	0.18–1.69
	Supratectal elements, SEM	Psilate or striate	Predominantly crested	Psilate or striate
Ultrastructure (TEM)	Tectum	0.39–0.66; Semitectate	0.25–0.72; Semitectate	0.27–0.65; Semitectate
	Infratectum	0.11–0.33; columellate	0.27–0.6; Columellate	0.31–0.73; Columellate
	Footlayer	0.31–0.65; Thick, continuous	0.2–0.59; Continuous	0.53–0.93; Thick, continuous
	Endexine	< 0.1–0.2; Thin, discontinuous, thickened towards aperture	< 0.1; very thin, Discontinuous; thickened towards aperture	< 0.1–0.13; Thin, discontinuous; thickened towards aperture
	Exine thickness	1.27–1.51	0.89–1.6	0.9–1.96
	Peculiarities	Indistinct internal tectum	Internal tectum	None

*PV* polar view, *EV* equatorial view, *S* sexine, *N* nexine; All measurements are given in [µm]

For references please see the Supplementary Material 3

### Pollen morphology and ultrastructure of *Craigia*

Genus: *Craigia* (Figs. 2A–L, 6A–D, 7A–D, Table 1, Supplementary Material 3).

*Description*: Pollen, dispersal unit monad, size class medium, polar axis 13–28 µm in LM, 19–20 µm in SEM, equatorial diameter 29–37 µm in LM, 26–31 µm in SEM, P/E-ratio oblate, outline in polar view convex-triangular to circular (Fig. 2A–D, G–J); aperture condition brevi(3)colporate; costae at apertures crescent- to lense-shaped in polar view (LM), 1.5–6.8 µm thick and 5.3–8.7 µm wide (LM), nexine at aperture sometimes curled inward forming a more or less acute to 90° angle α, rarely obtuse α (LM) (Fig. 6A–D). Ornamentation reticulate in LM, nano- to microreticulate to reticulate, perforate in SEM (Figs. 2E, F, K, L, 7A, C), muri of reticulum either psilate or striate, striae parallel to murus direction, murus width 0.21–0.50 µm (SEM), transition from muri to lumen at a steep angle (~ 90°), lumen diameter 0.14–1.98 µm (SEM), lumen can be rounded/polygonal or narrow and elongated, lumina of reticulum filled with branching columellae forming a psilate base interrupted by many perforations, opposite hemispheres having slightly different ornamentation, reticulum coarser on distal face (Fig. 2E, F, K, L); pollen wall 1.2–2.2 µm (LM), 1.27–1.51 µm (TEM), sexine equally thick to double the thickness of the nexine (LM), tectum semitectate 0.39–0.66 µm, indistinct internal tectum (Fig. 6B), infratectum columellate 0.11–0.33 µm, foot layer thick, continuous 0.31–0.65 µm, endexine thin, discontinuous < 0.1–0.2 µm (Supplementary Material 3).

*Remarks*: We investigated two herbarium specimens for pollen, one open flower and a closed flower bud. The range in pollen morphology discovered was astonishing. In pollen from the flower bud the grains are barely reticulate in LM and nano- to microreticulate in SEM with the presence of very faint striae on the muri (Fig. 2E, F). On the contrary, pollen grains from the open flower have a very prominent reticulum observed with LM and large lumina in SEM. Their striae are also more distinct (Fig. 2K, L). We assume that this covers the entire morphological range of *Craigia* pollen. Despite the size differences of lumina in pollen from the two specimens, their muri are comparable and between 0.21–0.50 µm wide. The pollen in the flower bud was not fully mature, as evidenced by its thicker pollen wall compared to that of the open flower. An internal tectum (it) is described as an “additional more or less continuous layer within the infratectum” (Halbritter et al. 2018 pp. 391–392). It is often encountered in pollen of Asteraceae but also occurs in Rosaceae and Ranunculaceae pollen.

### Pollen morphology and ultrastructure of *Mortoniodendron*

Genus: *Mortoniodendron* (Figs. 3 A–L, 4 A–F, 6 E–J, 7 E–J; Table 1; Supplementary Material 3 and 4).

*Description*: Pollen, dispersal unit monad, size class small to medium, polar axis 13–20 µm in LM, 11–19 µm in SEM, equatorial diameter 20–33 µm in LM, 16–29 µm in SEM, P/E-ratio oblate, outline in polar view circular, rarely convex-triangular (Figs. 3A–D, G–J, 4A–D; Supplementary Material 4, Figs. S1A–D, G–J, S2A–C, G–J, S7A–D, H–K); aperture condition brevi(3)colporate; nexine thickening at apertures crescent- to lense-shaped in polar view (LM), 1.3–4.3 µm thick and 4.3–8.3 µm wide (LM), angle α 90° to slightly obtuse (Fig.6E, F, I, J; Supplementary Material 4, Figs. S1A, G, S2A, G, S3A, H). Ornamentation reticulate in LM, nano- to microreticulate to reticulate, perforate in SEM, muri width 0.19–0.51 µm (SEM), lumen diameter 0.36–3.17 µm (SEM), lumina polygonal and angular in outline, lumina filled with densely packed free-standing columellae, these end in gemmae, gemmae of varying sizes, muri crested (Figs. 3E, F, K, L, 4E, F, 6E, G, I; Supplementary Material 4, Figs. S1E, F, K, L, S2D–F, K, L, S3E, F, L, M); pollen wall 1.1–2.2 µm thick (LM), exine 0.89–1.6 µm (TEM), sexine equally thick to double the thickness of the nexine (LM), tectum semitectate 0.25–0.72, internal tectum (Figs. 6F, H, J, 7F, H, J; Supplementary Material 4, Fig. S3G); infratectum columellate 0.27–0.6 µm, foot layer thick, continuous 0.2–0.59 µm, endexine very thin, discontinuous < 0.1 µm (Supplementary Material 3), thickening at apertures can be indistinct (Fig. 6G, H).

*Remarks*: Pollen of *Mortoniodendron* is generally the smallest within the subfamily. All species have a reticulate ornamentation in both LM and SEM, and the lumina are filled by free-standing columellae. The free-standing columellae seem to form a layer extending below the tectum (the muri of the reticulum). Under that perspective, the gemmae form an internal tectum, and the muri represent the regular tectum. The size of the brochi can vary slightly but is uniform overall. Muri are usually crested and have a triangular outline in cross-section. Only *M. apetalum* has flat muri (Supplementary Material 4, Fig. S1K, L). Pollen from all species, but one, have prominent costae formed by thickened nexine at the apertures. *Mortoniodendron vestitum* pollen has apertures without such a thickening (Fig. 6 G, H). *Mortoniodendron sulcatum* is so far the only member of the genus producing pollen with a reticulum that decreases in size from the poles towards the equator (Supplementary Material 4, Fig. S3D) and occasionally brevi(4)colporate aperture condition (Supplementary Material 4, Fig. S3G).

### Pollen morphology and ultrastructure of *Tilia*

Genus: *Tilia* (Figs. 4G–L, 5A–L, 6K–P, 7K–P; Table 1; Supplementary Material 3 and 4).

*Description*: Pollen, dispersal unit monad, size class medium (rarely large), polar axis 16–37 µm in LM, 16–26 µm in SEM, equatorial diameter 30–52 µm in LM, 27–46 µm in SEM, P/E-ratio oblate, outline in polar view convex-triangular to circular (Figs. 4G–J, 5A–D, G–J); aperture condition brevi(3)colporate; massive costae at apertures, crescent- to lense- shaped in polar view (LM), 2.1–7 µm thick and 8.7–16 µm wide (LM), angle α obtuse, sexine bulging outward in some species (Table 1; Fig. 6K–P; Supplementary Material 3). Ornamentation reticulate in LM, nano- to microreticulate, perforate, rarely reticulate in SEM, outline of lumina rounded polygonal to irregular, lumen diameter 0.15–1.72 µm (SEM), lumina filled with branching columellae, columellae fuse with muri, space between columellae forming perforations, muri width 0.18–1.69 µm (SEM), muri sometimes striate, striae parallel to muri direction (Figs. 4K, L, 5E, F, K, L, 7K, M, O; Supplementary Material 4, Fig. S4A, C, E, G, I, K), muri tend to have a trapezoidal shape in cross-section (TEM) with a narrower top and broader base leading to lumina that are funnel-shaped (Fig. 7L, N, P; Supplementary Material 4, Fig. S4B, D, F, H, J, L); pollen wall 1.1–2.2 µm thick (LM), exine 0.9–1.96 µm tick (TEM), sexine equally thick or thicker than nexine (LM), tectum semitectate 0.27–0.65 µm, infratectum columellate 0.31–0.73 µm, foot layer thick, continuous 0.53–0.93 µm, endexine thin, discontinuous < 0.1–0.13 µm (Table 1; Figs. 6L, N, P, 7L, N, P; Supplementary Material 3; Supplementary Material 4, Fig. S4B, D, F, H, J, L).

*Remarks*: *Tilia* pollen is well-studied and has a very uniform morphology. The genus produces the largest pollen grains in the subfamily regarding both the equatorial diameter and the length of the polar axis. In addition, *Tilia* pollen has a massive thickening (costae) around the apertures, up to 7 µm thick and 16 µm wide (Table 1). The nexine is thickened towards the inside of the pollen grain, as in the other genera, but sometimes the sexine is also bulged outwards, adding to the apertural thickening. The ornamentation is reticulate in LM and sometimes with striate suprasculpture (occurring in *T. americana*, *T. endochrysea* [Fig. 4K, L], and *T. tomentosa*).

### *Pollen morphology and ultrastructure of fossil *in situ* Tilioideae pollen from Messel*

#### *Specimen: Tilioideae Messel A (*Fig. 8A–F*; *Supplementary Material 3*)*

Collection number: SMB ME 30688.

*Description*: Pollen, dispersal unit monad, size class medium, polar axis ca. 15 µm in LM, equatorial diameter 38–40 µm in LM, 34–36 µm in SEM, P/E-ratio oblate, outline in polar view circular (Fig. 8B, C); aperture condition brevi(3)colporate; costae at apertures crescent- to lense-shaped in polar view (LM), 3.0–7.5 µm thick and 9.3–10 µm wide (LM), acute to right angle α (LM) (Fig. 8B). Ornamentation reticulate in LM, nano- to microreticulate, perforate in SEM (Fig. 8B–E), free-standing columellae in lumina, muri of reticulum striate, striae perpendicular to murus direction (Fig. 8D, E), murus width 0.3–0.4 µm (SEM), lumen diameter 0.3–0.9 µm (SEM); pollen wall 1.5–1.6 µm (LM), 0.71–0.99 µm (TEM), sexine thicker than nexine (LM), tectum semitectate 0.32–0.37 µm, indistinct internal tectum (Fig. 8F), infratectum columellate 0.31–0.34, foot layer continuous 0.3–0.36 µm, endexine thin < 0.1 µm and thickened towards aperture (Fig. 8F; Supplementary Material 3).

#### *Specimen: Tilioideae Messel B (*Fig. 8G–L*; *Supplementary Material 3*)*

Collection number: SMB ME 7415.

*Description*: Pollen, dispersal unit monad, size class medium, equatorial diameter 44–46 µm in LM, 37–39 µm in SEM, P/E-ratio oblate, outline in polar view circular (Fig. 8H, I); aperture condition brevi(3)colporate; costae at apertures crescent- to lens-shaped in polar view (LM), 3.5–6.3µm thick and 11.4–14.3 µm wide (LM), obtuse angle α (LM) (Fig. 8H). Ornamentation reticulate in LM, nano- to microreticulate, perforate, rarely reticulate in SEM (Fig. 8I-K), free-standing columellae in lumina, muri of reticulum with faint striae, striae perpendicular to muri direction (Fig. 8J, K), muri width 0.25–0.33 µm (SEM), lumen diameter 0.3–1 µm (SEM); pollen wall ca. 1.5 (LM), 0.96–1.11µm (TEM), sexine thicker than nexine (LM), tectum semitectate 0.27–0.39 µm, indistinct internal tectum (Fig. 8L), infratectum columellate 0.39–0.46, foot layer continuous 0.23–0.33 µm, endexine thin < 0.1 µm and thickened towards aperture (Fig. 8L; Supplementary Material 3).

#### *Specimen: Tilioideae Messel C (*Fig. 9A–F*; *Supplementary Material 3*)*

Collection number: SMB ME 7412.

*Description*: Pollen, dispersal unit monad, size class small, polar axis ca. 15 µm, equatorial diameter 22–23 µm in LM, 19–21 µm in SEM, P/E-ratio oblate, outline in polar view circular (Fig. 9B, C); aperture condition brevi(3)colporate; costae at apertures crescent shaped in polar view (LM), 2.0–4.7µm thick and 5.3–5.7 µm wide (LM), acute to right angle α (LM) (Fig. 9B). Ornamentation scabrate to reticulate in LM, nano- to microreticulate, perforate, rarely reticulate in SEM (Fig. 9I–K), muri of reticulum with faint striae, striae perpendicular to muri direction (Fig. 9D, E), reticulum on proximal hemisphere (Fig. 9D) finer than on distal pole (Fig. 9E), muri width 0.18–0.29 µm (SEM), lumen diameter 0.08–1 µm (SEM); pollen wall 0.9–1.0 µm thick (LM), 0.7–0.78 µm (TEM), sexine and nexine equally thick (LM), tectum semitectate 0.22–0.24 µm, infratectum columellate ca. 0.23 µm, foot layer continuous 0.25–0.27 µm, endexine thin 0.1–0.17 µm and thickened towards aperture (Fig. 9F; Supplementary Material 3).

#### *Specimen: Tilioideae Messel D (*Fig. 9G–L*; *Supplementary Material 3*)*

Collection number: SMB ME 31205.

*Description*: Pollen, dispersal unit monad, size class small, polar axis ca. 14 µm, equatorial diameter 21–23 µm in LM, 19–20 µm in SEM, P/E-ratio oblate, outline in polar view circular (Fig. 9H, I); aperture condition brevi(3)colporate; costae at apertures crescent shaped in polar view (LM), 2.1–4.6 µm thick and 4.7–5.0 µm wide (LM), acute angle α (LM) (Fig. 9H). Ornamentation scabrate in LM, nanoreticulate and perforate in SEM (Fig. 9J, K), muri of reticulum with faint suprasculpture (Fig. 9K), reticulum on proximal hemisphere (Fig. 9J) finer than on distal pole (Fig. 9K), muri width 0.16–0.26 µm (SEM), lumen diameter < 0.2 µm (SEM); pollen wall 0.9–1.0 µm thick (LM), 0.43–0.59 µm (TEM), sexine and nexine equally thick (LM), tectum semitectate 0.18–0.25 µm, infratectum columellate 0.21–0.25 µm, foot layer continuous 0.11–0.14 µm, endexine thin < 0.1 µm and thickened towards aperture (Fig. 9L; Supplementary Material 3).

### *How to distinguish Tilioideae pollen (*Fig. 11*)*

To start identifying Tilioideae-type pollen to the genus level (or exclude this subfamily) we suggest separating the identification workflow into 4 steps (Fig. 11). The steps can be followed in numerical order (1–4) or the order of your choice. Generally, it makes sense to start with the LM identification, followed by SEM, and finally TEM.

The thickening of the pollen wall around apertures (*i.e.*, the costae; Fig. 11, step 1a) is formed by nexine, corresponding roughly to the foot layer of the ektexine. When the thickening is wider than 8.7 µm and thicker than 4.3 µm, the pollen is probably from *Tilia* (Table 1; Supplementary Material 3). If the thickening is narrower than 8.7 µm, both *Craigia* and *Mortoniodendron* are potential candidates for the pollen origin. If the thickening is up to 6.8 µm thick, the pollen probably came from *Craigia*, while *Mortoniodendron* shows a maximum thickening of 4.3 µm (Table 1; Supplementary Material 3). When the thickening around apertures falls within the lower values that overlap between genera, additional characters need to be considered. Another trait of the thickening around apertures is the angle α between the thickening and the interapertural wall (Fig. 1; Fig. 11, step 1b). When the angle α between the costae and the interapertural wall is obtuse to 90°, it is most likely *Tilia* pollen (*e.g.*, Fig. 6K, M, O; Table 1; Supplementary Material 3). If the angle is acute to 90° (or only slightly obtuse), it indicates affiliation to *Craigia* or *Mortoniodendron*. Consult size, ornamentation, and ultrastructure of the pollen wall (Fig. 11, steps 2, 3, 4). There exists one special case, where no particular thickening of the wall can be observed (Fig. 11, step 1c): *Mortoniodendron vestitum* pollen lacks the characteristic exine thickening around apertures (costae), but the pollen can be assigned to *Mortoniodendron* based on the morphology of the reticulum observed with SEM, namely the free-standing columellae (Fig. 3K, L; Fig. 11, step 3). When pollen lacks the apertural thickening and free-standing columellae but otherwise resembles Tilioideae pollen, it is helpful to examine the pollen morphology of related subfamilies within the Malvaceae, *i.e.*, Bombacoideae and Sterculioideae.

The size of the pollen can also be used when segregating Tilioideae-type pollen, specifically the equatorial diameter in polar view observed with LM (Fig. 11, step 2; Table 1; Supplementary Material 3). When the pollen is smaller than 29 µm, *Craigia* and *Tilia* can be excluded, then it most likely belongs to *Mortoniodendron*. When the pollen size is between 29 and 37 µm, then both *Craigia* and *Tilia* are potential parent plant candidates. When pollen is larger than 37 µm (and up to 52 µm), it comes most likely from *Tilia*.

To assign Tilioideae-type pollen to genus level (or exclude this subfamily) the reticulate ornamentation needs to be investigated (Fig. 11, step 3). When this ornamentation is relatively coarse in LM, it can fit any of the three genera, *Craigia*, *Mortoniodendron,* or *Tilia*. SEM micrographs need to be consulted. *Mortoniodendron* pollen is characterized by a reticulum that varies greatly in lumen size, from 0.36–3.17 µm in diameter, and is paired with crested muri and lumina are filled with free-standing columellae (Fig. 3F). *Craigia* pollen is characterized by ornamentation ranging from nanoreticulate to microreticulate or reticulate (0.14–1.98 µm), psilate or striate muri, and the lumina are flat and psilate with perforations (Fig. 2K, L). *Tilia* pollen is ornamented similarly to that of *Craigia* pollen – psilate or striate muri and lumina of 0.15–1.72 µm in size – but the lumina are funnel-shaped due to branching columellae with perforations in-between (Fig. 5E, F). When the reticulum is very fine in LM and SEM, *Mortoniodendron* can be excluded as the potential source plant. Pollen with nanoreticulate or perforate reticulum needs to be viewed on both hemispheres in SEM. When the reticulum is open enough to see the lumen, then the same characteristics can be applied as above: a psilate and perforate lumen indicates *Craigia* (Fig. 2K, L), a funnel-shaped lumen with perforations indicates *Tilia* (Fig. 5E, F). If the reticulum is so small that no statement about the nature of the lumen can be made (Figs. 2E, F, 5K, L), then LM and TEM observations must be consulted for the aperture conditions and the ultrastructure of the pollen wall (Fig. 11, steps 1, 2 and 4).

Generally, the pollen walls in Tilioideae are formed by a tectate to eutectate tectum, a columellate infratectum, a thick-continuous foot layer, and some remnants of endexine. Special attention needs to be given to the transition zone between tectum and infratectum. In *Mortoniodendron*, the gemmate columellae subside below the muri of the reticulum and form an internal tectum (Fig. 7F). In *Craigia* pollen, this gemmate to granulate layer is less continuous, forming an indistinct internal tectum (Fig. 6A), and in *Tilia* pollen it is absent, lacking an internal tectum.

Consequently, all four avenues of the identification key given in Fig. 11 must come to the same result. A contradiction can indicate affiliation to a different subfamily within the Malvaceae (Bombacoideae or Sterculioideae) or, in case of fossil pollen, the grain in question might be part of the Tilioideae but represents an extinct taxon. In conclusion, one must study Tilioideae pollen grains with both LM and SEM, and ideally also with TEM, to document enough diagnostic features enabling the identification or rejection of *Craigia*, *Mortoniodendron*, and *Tilia* as potential parent plants of fossil Tilioideae-type pollen.

## Discussion

### Comparing fossil pollen to the Tilioideae dataset and identification key

As an example of the applicability of the Tilioideae dataset presented herein, we use it to analyse 5 fossil records from two different geological ages (Supplementary Material 3). First, in situ pollen from four Tilioideae-type flowers (referred to as flowers A to D) from the Eocene of Messel, Germany, that we investigated with LM, SEM, and TEM (Figs. 8A–L, 9A-L). The second, a well-known fossil *Craigia* pollen type from the Late Miocene of Hambach, Germany, previously investigated with LM, SEM, and TEM (Fig. 10A–F; Kvaček et al. 2002; Zetter et al. 2002).

The in situ pollen grains from the four Eocene flowers all share the typical Tilioideae-type pollen characters. They are monads of small to medium size, circular to triangular-convex outline in polar view, P/E-ratio oblate, brevi(3)colporate with a costae at the apertures, and generally reticulate in ornamentation. Pollen from flowers A and B is reticulate in LM and nano- to microreticulate in SEM (Fig. 8D, E, J, K), with pollen of flower A also being perforate. The lumina in pollen from both flowers range from 0.3 to 1 µm (Supplementary Material 3). Flowers C and D have scabrate to reticulate pollen in LM and nanoreticulate to perforate in SEM (Fig. 9D, E, J, K), with pollen of flower C showing perforations as well and pollen from flower D having lumina smaller than 0.2 µm. The muri of the reticulum in pollen from all four flowers have a striate suprasculpture running perpendicular to the long axis of the muri. These striae are very prominent in pollen from flower A (Fig. 8D), while they are fainter in pollen from the other flowers (B to D). The size of the reticulum, in combination with the suprasculpture, excludes *Mortoniodendron* as a potential parent plant of all four flowers. The reticulum in extant *Mortoniodendron* pollen is coarse, and the muri are crested or psilate. However, *Tilia* and *Craigia* pollen grains can have a fine reticulum (nano- to microreticulate, even perforate). Striae may be present in *Craigia* and *Tilia* pollen but they always run parallel to the long axis of the muri (Figs. 2K, L, 4K, L). In pollen from flowers A and B, the lumina of the reticulum contain free-standing columellae, also observed as an internal tectum in TEM (Fig. 8F, L). Pollen from flowers C and D lack such structures. Extant *Mortoniodendron* pollen has free-standing columellae that form an internal tectum in the wall ultrastructure. *Craigia* has an indistinct internal tectum, but this is rare in *Tilia*. In that regard, pollen from flowers A and B is similar to that occurring in *Mortoniodendron* and *Craigia*, and pollen from flowers C and D compares better with that of *Tilia*. Based on the thickness and width of the costae in the fossil pollen, *Tilia* could be associated with flowers A and B. In contrast, pollen from flowers C and D is more similar to that of *Craigia* and *Mortoniodendron*. Also, pollen from flowers A, C, and D has acute to 90° α angles, as observed for both *Craigia* and *Mortoniodendron* pollen. Only pollen from flower B has an obtuse angle (Fig. 8H), characteristic of *Tilia*. Regarding the pollen size, pollen from flowers A and B fits into the size range of extant *Tilia* pollen, while pollen from flowers C and D is comparable to that of *Mortoniodendron*. Combining all morphological and ultrastructural features, pollen from flower A compares to that of *Tilia* in both pollen and costae size. However, the costae of the fossil pollen have an acute to 90° α angle, which is rare in *Tilia* pollen. Also, the free-standing columellae/ internal tectum (Fig. 8D–F) rather affiliates the fossil pollen to that of *Mortoniodendron*. This contradiction suggests that the pollen from flower A may represent an extinct member of Tilioideae or another closely related Malvaceae. This is underlined by the presence of a completely different suprasculpture. Striate muri with the striae running perpendicular to the long axis of the muri have never been observed in pollen of extant Tilioideae (Supplementary Material 3). Pollen grains from flower B are similar to those of flower A except the α angle. In pollen from flower B, the angle α is obtuse. Flowers C and D pollen resemble extant *Mortoniodendron* pollen in size, aperture, and costae configuration (width, thickness, and α). However, they are vastly different in exine ornamentation and have a much finer reticulum than extant *Mortoniodendron* pollen. Also, *Mortoniodendron* pollen has an internal tectum observed as free-standing columellae in the lumen of the reticulum, the pollen from flowers C and D lack such internal tectum (Fig. 9F, L). This excludes *Mortoniodendron* as potential parent plants of the fossils. Like pollen from flowers A and B, the pollen from flowers C and D most likely belong to an extinct taxon within Tilioideae or other closely related Malvaceae.

Miocene flowers/buds from Hambach, Germany, are believed to originate from the same parent plant as fruits of *Craigia bronnii* (Unger) Z. Kvaček, Bůžek et Manchester. These buds contain in situ pollen and were previously described and assigned to *Craigia* (Kvaček et al. 2002; Zetter et al. 2002). Here, we compare this Miocene pollen’s LM, SEM, and TEM morphology and ultrastructure to the new Tilioideae pollen framework provided herein. Pollen of *Craigia bronnii* is medium-sized and with an equatorial diameter of 31–34 µm in LM and SEM. The pollen has an oblate P/E-ratio and is circular to convex-triangular in outline. The aperture configuration is brevi(3)colporate and the sculpture is reticulate in LM and nano- to microreticulate in SEM. The muri have striate suprasculpture, with the striae running parallel to the long axis of the muri (Fig. 10D, E). These combined characters exclude *Mortoniodendron* as the potential parent plants of this pollen. The costae in the fossil Miocene pollen are 2.0–4.9 µm thick and 6.8–7.5 µm wide, which makes them smaller than the costae in extant *Tilia* pollen but fits perfectly within the range of extant *Craigia yunnanensis* pollen (Supplementary Material 3). The α angle in the fossil pollen is acute to 90° (Fig. 10A), which excludes most extant *Tilia* species and corroborates further similarities with pollen of *Craigia yunnanesis*. Also, the pollen wall of the fossil has an inconspicuous internal tectum similar to that observed in extant *Craigia* pollen. *Tilia* pollen does not have such internal tectum. The fossil Miocene pollen is microreticulate and compares best to extant *Craigia* pollen, especially that extracted from a flower bud (Fig. 2A–F) that is potentially not fully matured as evidenced by the much thicker pollen wall (Furness 2011; Sutthinon et al. 2024).

The few examples listed in this section demonstrate the potentiality of the Tilioideae pollen framework presented in this study. The combined LM, SEM, and TEM analyses can be used to verify or reject the presence of *Craigia*, *Mortoniodendron*, and *Tilia* in the palynological record and/or suggest affiliation to other closely related extinct or extant Malvaceae.

### Is it important to differentiate Tilioideae-type pollen to genus level?

**Fig. 12:**
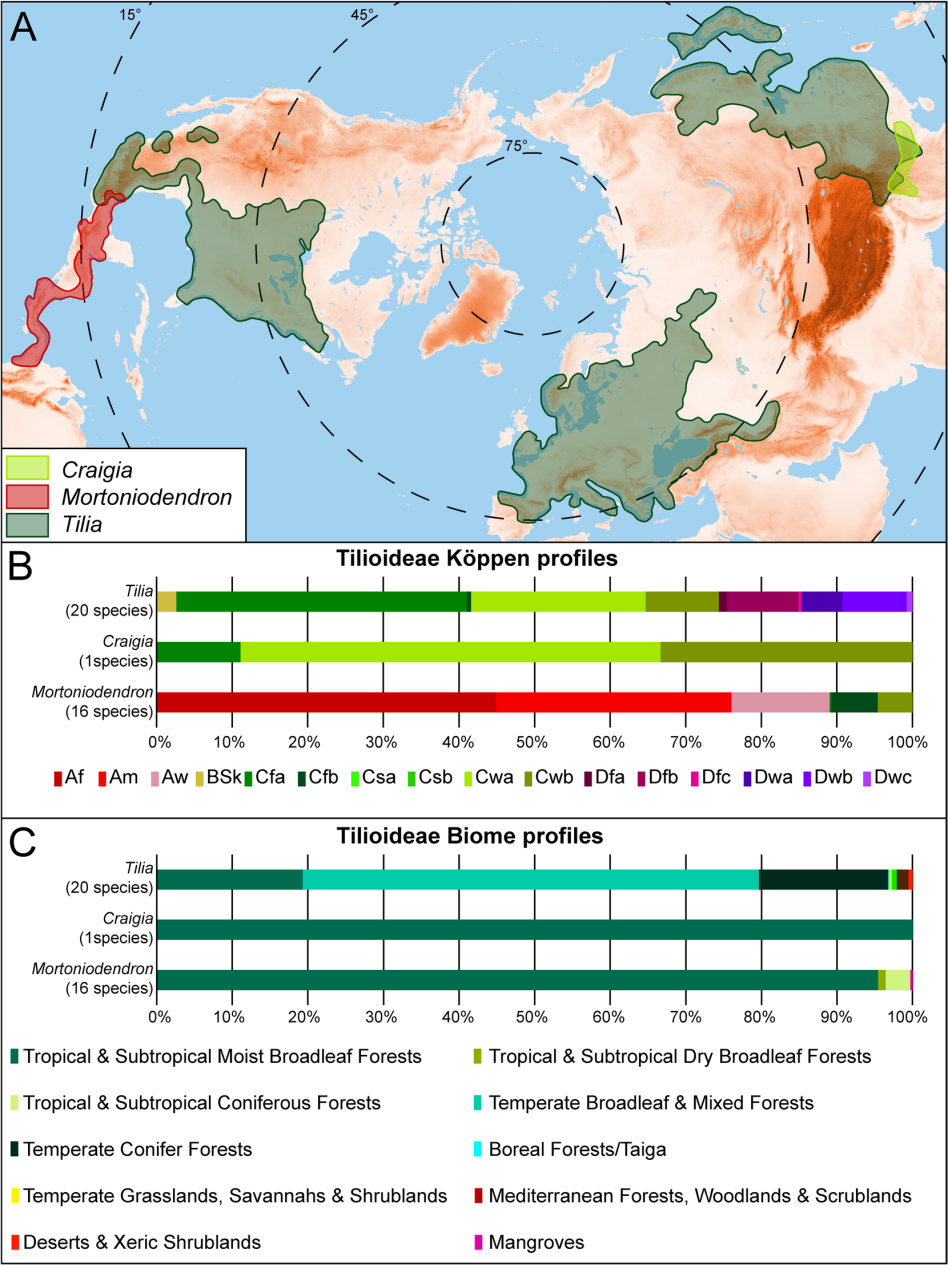
Distribution map and climate/biome profiles for Tilioideae. **A**. Geographic distribution of Tilioideae. **B**. Köppen climate profiles. **C**. Vegetation biome profiles. Based on data provided in Supplementary Material 1

**Fig. 13:**
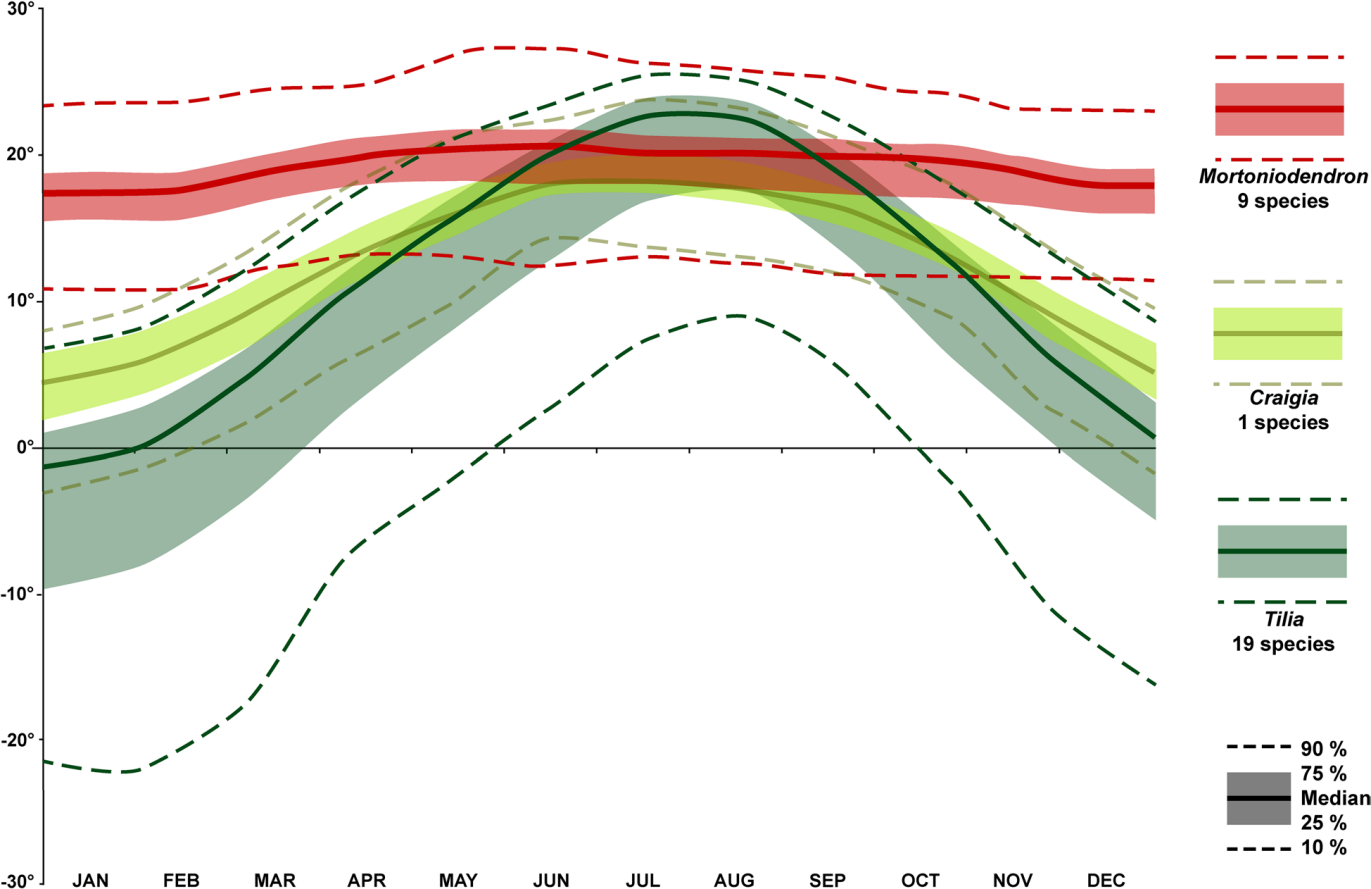
Minimum monthly temperature [°C] of Tilioideae. Graph includes only species with more than ten occurrences (n ≥ 10). The climate data was normalized to avoid bias towards the dominance of species with large data sets. Graph based on Supplementary Material 1 and 2

The effort to taxonomically place fossil Tilioideae pollen into the “correct” genus will not only help document the origins and paleophytogeographic histories of *Craigia*, *Mortoniodendron*, and *Tilia*, but it will also make paleovegetation reconstructions and paleoclimate evaluations more precise. Interpretations of past vegetation units, paleohabitats, and other paleoecological aspects of fossil plants, especially from Cenozoic palynofloras, are based on potential modern analogues or taxa believed to be closely related to the fossils (*e.g.*, Denk et al. 2011; Geier et al. 2022; Bouchal et al. 2024 and references therein). There are numerous Tilioideae pollen records, among others, from the Paleocene to Oligocene of Europe (*e.g.*, Mai 1961; Muller 1981; Stuchlik et al. 2014; and references therein). This time period, especially the Eocene, holds the highest diversity of Tilioideae-type pollen (Mai 1961; Krutzsch 2004), most of which have been lumped into different form species of the genus *Intratriporopollenites* (Mai 1961; Stuchlik et al. 2014). For many of the Paleogene European Tilioideae pollen records, the affiliation to extinct or extant genera/taxa is often vague, and it is unclear which sets of morphological/ultrastructural features have been used to pinpoint their taxonomic assignment. Still, the macro/mesofossils from around the globe suggest that all extant genera of Tilioideae already existed in the Eocene (Manchester 1994; Kvaček et al. 2005; Geier et al. 2023a and references therein), underscoring the importance of differentiating between pollen from the three extant genera as well as extinct groups. Concerning the paleoecological aspects, especially paleoclimate and paleo-biomes, it makes a considerable difference if a fossil Tilioideae pollen is assigned to *Craigia*, *Mortoniodendron*, or *Tilia*. *Mortoniodendron* predominantly occurs in fully humid equatorial rainforest (*Af*) and partly equatorial monsoonal (*Am*) climates with more or less constant minimum monthly temperature ≥ 18C° throughout the year (Figs. 12 and 13). In accordance with the climate preferences, *Mortoniodendron* is more or less confined to the Tropical & Subtropical Moist Broadleaf Forests Biome (Fig. 12). This genus seems to be a niche conservative tropical element. *Craigia* is also more or less confined to the same biome as *Mortoniodendron*, but the climate is strikingly different. *Craigia* grows mainly in areas enduring winter dry warm temperate climates with hot to warm summers (*Cwa*, *Cwb*; Fig. 12), and with a minor presence in fully humid warm temperate climates with hot summers (*Cfa*). This genus is subtropical, enduring some temperature changes between seasons (Fig. 13) as well as dry winter periods, and is more characteristic for deciduous forests within the Tropical & Subtropical Moist Broadleaf Forests Biome (Fig. 12). *Tilia* prefers different biomes, they mostly occur in the Temperate Broadleaf & Mixed Forests and Temperate Conifer Forests Biomes, with a minor presence in Tropical & Subtropical Coniferous Forests and the Tropical & Subtropical Moist Broadleaf Forests Biomes (Fig. 12). This is also reflected in the prevailing climate types where *Tilia* are presently growing. Unlike *Mortoniodendron* and *Craigia*, *Tilia* often thrives in snow climates (*D*, cold-temperate to boreal) but is also typically found in fully humid or seasonally dry warm temperate climates with hot or warm summers (*Cfa*, *Cfb*, *Csa*, *Cwa*). Out of the three Tilioideae genera, *Tilia* is the most cold-tolerant and has the widest distribution within the temperate realm (Fig. 12). The genus is temperate to subtropical, can endure drastic temperature amplitudes between seasons (Fig. 13), is not prominent in areas with dry seasons, and is characteristic of mixed deciduous broadleaf and conifer forests. In short, it does matter for the paleoecological interpretation in which genus the fossil Tilioideae pollen grains are placed.

### Future goals for differentiating fossil Tilioideae-type pollen

As examples for future research applying the Tilioideae dataset provided herein, we highlight a few cases from the Paleogene of Europe. The earliest fossil Tilioideae pollen records from Europe are from the Paleocene. Among these records are four different morphotypes (*Intratriporopollenites* spp.*)* documented from Menat, France, based on LM observations by Kedves and Russell (1982). At that time, the authors suggested botanical affinity with Tiliaceae (preferably *Tilia*). This has recently been questioned by El Atfy et al. (2023) based on the lack of convincing reproductive structures of *Tilia* from pre-Eocene sediments in Europe. These and other Paleocene Tilioideae pollen types need to be reinvestigated using combined LM, SEM, and TEM and compared to the comprehensive morphological and ultrastructural framework provided herein. This will establish if *Craigia*, *Mortoniodendron*, or *Tilia* were already present in Europe during the Paleocene or if some/all the pollen morphologies fall outside the range of living taxa and represent either extinct Tilioideae or other closely related Malvaceae (*e.g.*, Brownlowioideae, Bombacoideae, Sterculioideae).

Tilioideae-type pollen grains have also been reported from numerous Eocene palynofloras of Europe (*e.g.*, Pflug 1952; Mai 1961; Muller 1981; Thiele-Pfeiffer 1988; Nickel 1996; Hammer-Schiemann 1998; Hofmann and Zetter 2001; Stuchlik et al. 2014 and references therein). From Messel, Thiele-Pfeiffer (1988) reported two Tilioideae pollen types (*Intratriporopollenites* spp.) that were hard to place taxonomically but suggested affinities to both Brownlowioideae and *Tilia* for one of the pollen types. Similarly, Nickel (1996) described three Tilioideae pollen types from the middle Eocene of Eckfeld (Germany), again with no decisive taxonomic placement but suggesting affinities to both Brownlowioideae and *Tilia* for one of the pollen types. Based on combined LM and SEM, Bouchal et al. (2025) recently reported both *Craigia* and *Mortoniodendron* plus two additional indet. Tilioideae pollen types from Messel. From a paleoecological point of view, *Tilia* sounds most unfitting for the late early to middle Eocene of Europe, a time when this geographic region was characterized by Paratropical Rainforests (Mai 1995). Based on the climate and biome preferences of *Craigia*, *Mortoniodendron*, and *Tilia* it is most likely that *Mortoniodendron* is represented by late early to middle Eocene European pollen records. Again, this needs to be investigated further with a combined LM, SEM, and TEM study of the fossil Eocene pollen grains so they can be adequately compared to the data set provided herein, especially that of *Mortoniodendron* and *Craigia*.

In Europe, the earliest convincing macrofossil records, including reproductive structures of both *Craigia* and *Tilia*, are from the early Oligocene (Hably et al. 2000; Kvaček and Walther 2004; Kvaček et al. 2005). More Tilioideae-type pollen from Oligocene sediments of Europe needs to be reinvestigated, both with SEM and their ultrastructure. Those results need to be compared to the data on extant taxa presented herein, as well as studies on Eocene pollen from the same geographical region. Only in this way is it possible to pinpoint the actual arrival time of both *Craigia* and *Tilia* to Europe and their regional development during the Eocene and Oligocene.

Highlighting just these three examples for the Paleogene of Europe, it is evident that several research questions need answering and gaps in the fossil record need to be filled. By applying combined light- and electron microscopy when investigating fossil Tilioideae pollen, it is possible to assign them to *Craigia*, *Mortoniodendron*, or *Tilia* or to exclude these genera and suggest comparison to other extinct or extant Malvaceae producing similar pollen. In this way, the origin and dispersal history of Tilioideae can be better resolved.

## Conclusion

At first look, the pollen of *Craigia*, *Mortoniodendron*, and *Tilia* are very similar. When scrutinized using combined LM, SEM, and TEM, it is possible to differentiate between pollen of the three genera. Pollen of *Mortoniodendron* is the smallest of the three genera and consistently has the largest lumina in the reticulate exine. Additionally, the lumina in *Mortoniodendron* pollen are filled with free-standing columellae, whose ends form an internal tectum. *Craigia*, a relict genus, has medium-sized pollen most similar to its sister genus *Tilia*. However, *Craigia* pollen is generally smaller, has apertural costae that are slender, and has an acute to 90° α angle. The costae in *Tilia* pollen are usually wider, thicker, and more prominent with an obtuse α angle. Additionally, *Craigia* pollen has an inconspicuous internal tectum that is not observed in *Tilia* pollen. To highlight the potentiality of the Tilioideae pollen framework presented herein, we applied it to pollen from five fossil flowers/buds. The application shows how fossil Tilioideae-type pollen can be either confirmed as belonging to this subfamily or rejected. The importance of correctly affiliating fossil pollen to extant Tilioideae genera (or excluding them from Tilioideae) is underscored by the three genera’s different climate and biome preferences.

## Supplementary Information

Supplementary Material 1. Georeferenced occurrences of Tilioideae.

Supplementary Material 2. Distribution, climate, and ecoregions of Tilioideae.

Supplementary Material 3. Pollen morphology and ultrastructure of extant species and fossil specimens within Tilioideae.

Supplementary Material 4. Pollen morphology of six *Mortoniodendron* species and pollen wall ultrastructure of six *Tilia* species.

## Data Availability

All data supporting the findings of this study are either part of the published Manuscript or part of the Supplementary Material.
