# Supplementary material for: Morphology and ultrastructure of Tilioideae pollen: how to differentiate Craigia, Mortoniodendron, and Tilia
Source: Bot Stud. 2025 Jun 9;66:15. doi: 10.1186/s40529-025-00463-1 (PMC12149084; doi:10.1186/s40529-025-00463-1)
Supplement: Supplementary file 2 — Supplementary Material 2. Distribution, climate, and ecoregions of Tilioideae. [file 40529_2025_463_MOESM2_ESM.pdf]

Additional file 2
Distribution, climate, and ecoregions of Tilioideae

1. Köppen-Geiger climate symbols and defining criteria (Peel et al. 2007, Kottek et al. 2006, Rubel et al. 2017, Cui et al. 2021).

1st	2nd	3rd	Description and criteria
A			**Equatorial**/tropical—MTCM ≥ 18 °C
	f		Rainforest, fully humid—P_{dry} ≥ 60 mm
	m		Monsoonal—not *Af* & P_{dry} ≥ 100 − MAP/25
	s		Savannah with dry summer—P_{sdry} < 60 mm
	w		Savannah with dry winter—P_{wdry} < 60 mm
B			**Arid**—MAP < 10 × $P_{threshold}$
	W		Desert—MAP < 5 × $P_{threshold}$
	S		Steppe—MAP ≥ 5 × $P_{threshold}$
		h	Hot arid—MAT ≥ 18 °C
		k	Cold arid—MAT < 18 °C
C			**Warm temperate** (subtropical to temperate)—T_{hot} > 10 °C & 0 °C < MTCM < 18 °C
D			**Snow** (cold-temperate to boreal)—T_{hot} > 10 °C & MTCM ≤ 0 °C
	s		Summer dry—P_{sdry} < 40 mm & P_{sdry} < P_{wwet}/3
	w		Winter dry—P_{wdry} < P_{swet}/10
	f		Fully humid, without a dry season, i.e. not *s* or *w*
		a	Hot summer—T_{hot} ≥ 22 °C
		b	Warm summer—not *a* & T_{mon10} ≥ 4
		c	Cool/cold and short summer—not *a/b* & 1 ≤ T_{mon10} < 4
		d	Extremely continental: cold/very short or no summer & very cold winter—not *a/b* & MTCM < −38 °C
E			**Polar—**T_{hot} < 10 °C
	T		Tundra—(T_{hot} > 0)
	F		Ice, permanent frost (‘frost desert’)—(T_{hot} ≤ 0)

MAP = mean annual precipitation, MAT = mean annual temperature, T_{hot} = mean temperature of the hottest month, T_{cold} = mean temperature of the coldest month, T_{mon10} = number of months where the temperature is above 10 °C, P_{dry} = precipitation of the driest month, P_{sdry} = precipitation of the driest month in summer, P_{wdry} = precipitation of the driest month in winter, P_{swet} = precipitation of the wettest month in summer, P_{wwet} = precipitation of the wettest month in winter, $P_{threshold}$ = varies according to the following rules: if 70% of MAP occurs in winter then $P_{threshold}$ = 2 x MAT, if 70% of MAP occurs in summer then $P_{threshold}$ = 2 x MAT + 28, otherwise $P_{threshold}$ = 2 x MAT + 14. Summer (winter) is defined as the warmer (cooler) six months period of October to March and April to September.

2. Color coding.

2.1. Köppen-Geiger climate map colour coding.

Af	Am	As	Aw	BSh	BSk	BWh	BWk	Cfa	Cfb	Cfc
Csa	Csb	Csc	Cwa	Cwb	Cwc	Dfa	Dfb	Dfc	Dfd	Dsa
Dsb	Dsc	Dsd	Dwa	Dwb	Dwc	Dwd	EF	ET		

2.2. Biome map color coding using the Biome categories of Olson et al. (2001).

Tropical & Subtropical Moist Broadleaf Forests

Tropical & Subtropical Dry Broadleaf Forests

Tropical & Subtropical Coniferous Forests

Temperate Broadleaf & Mixed Forests

Temperate Conifer Forests

Boreal Forests / Taiga

Tropical & Subtropical Grasslands, Savannahs & Shrublands

Temperate Grasslands, Savannahs & Shrublands

Flooded Grasslands & Savannahs

Montane Grasslands & Shrublands

Tundra

Mediterranean Forests, Woodlands & Scrub

Desert & Xeric Shrublands

Mangroves

Abbreviations: Tropical & Subtropical Moist Broadleaf Forests (Tropic. & Subtropic. MBLF); Tropical & Subtropical Dry Broadleaf Forests (Tropic. & Subtropic. DBLF); Tropical & Subtropical Coniferous Forests (Tropic. & Subtropic. CF); Temperate Broadleaf & Mixed Forests (Temp. BLMF); Temperate Conifer Forests (Temp. CF); Tropical & Subtropical Grasslands, Savannahs & Shrublands (Tropic. & Subtropic. GLSSL); Temperate Grasslands, Savannahs & Shrublands (Temp. GLSSL); Flooded Grasslands & Savannahs (Flooded GLS); Montane Grasslands & Savannahs (Montane GLS), Mediterranean Forests, Woodlands & Scrub (MFWS); Deserts & Xeric Shrublands (DXSL).

3. Genus *Craigia* W.W.Sm. et W.E.Evans, 1921

3.1. Species *Craigia yunnanensis* W.W.Sm. et W.E.Evans, 1921

3.1.1. Köppen profile, distribution, and climate map – GBIF occurrences of *Craigia yunnanensis*; herbarium specimens (n = 18).

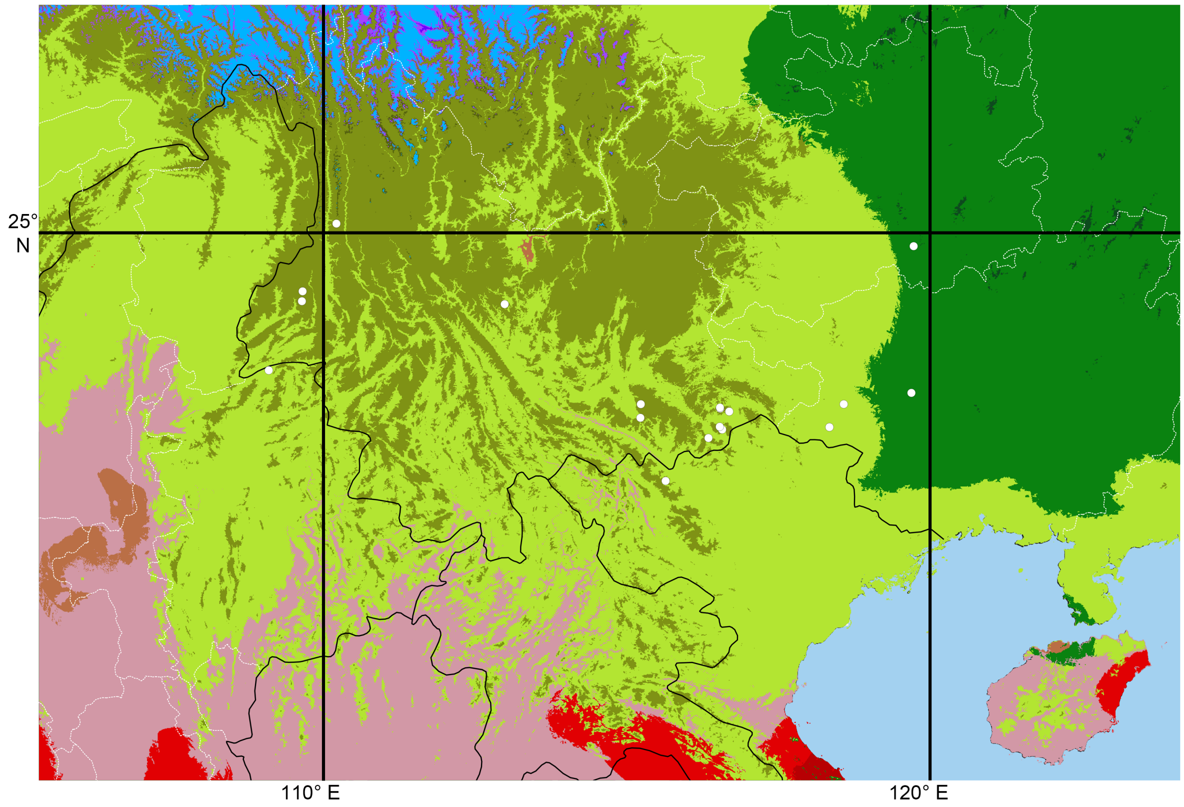

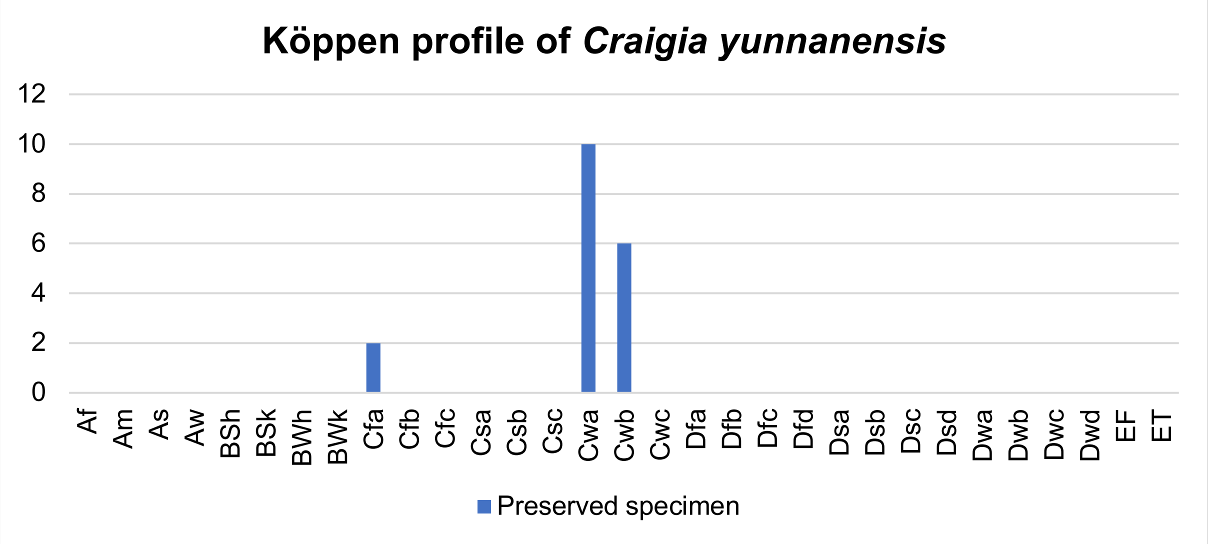

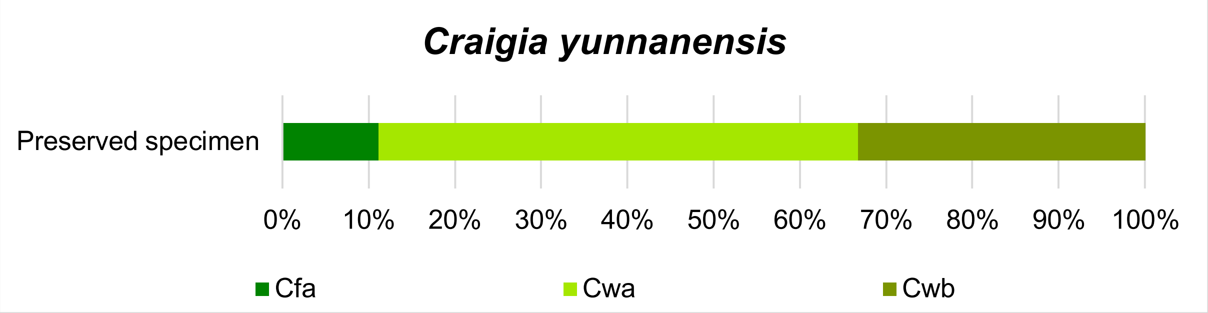

3.1.2. Biome profile, distribution, and biome map – GBIF occurrences of *Craigia yunnanensis*; herbarium specimen (n = 18).

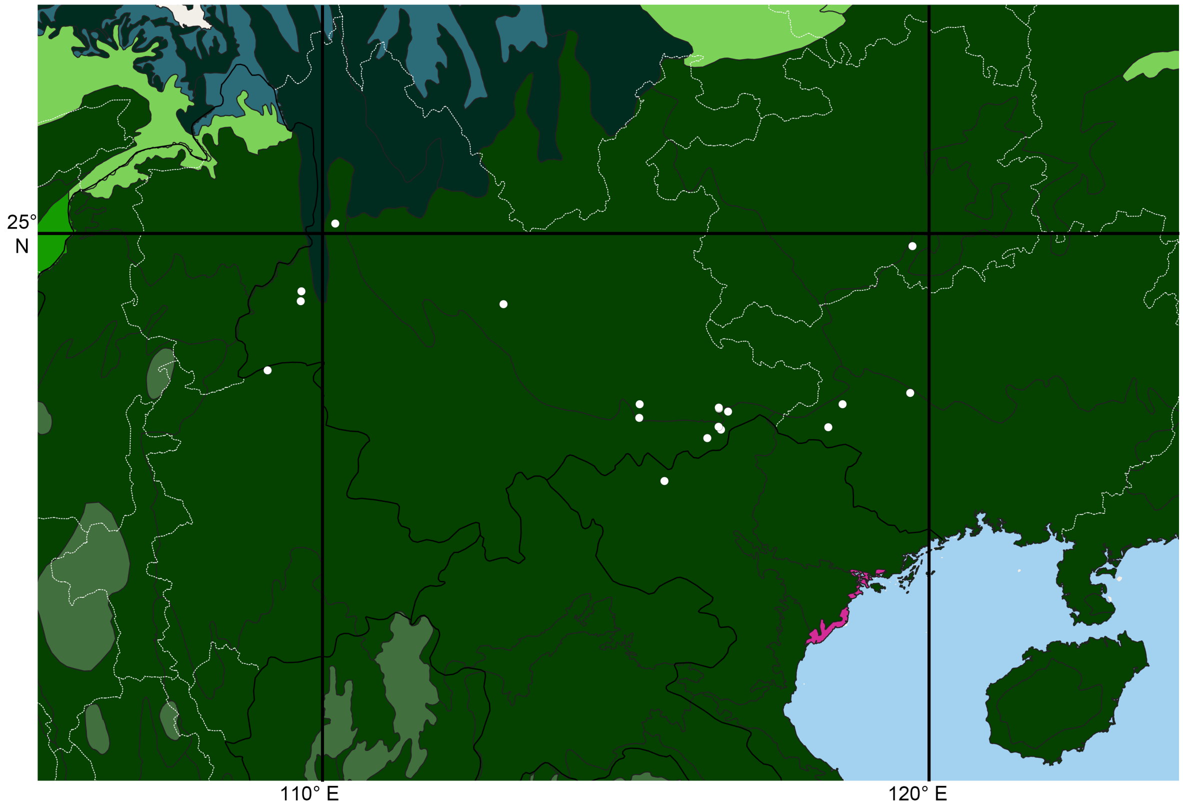

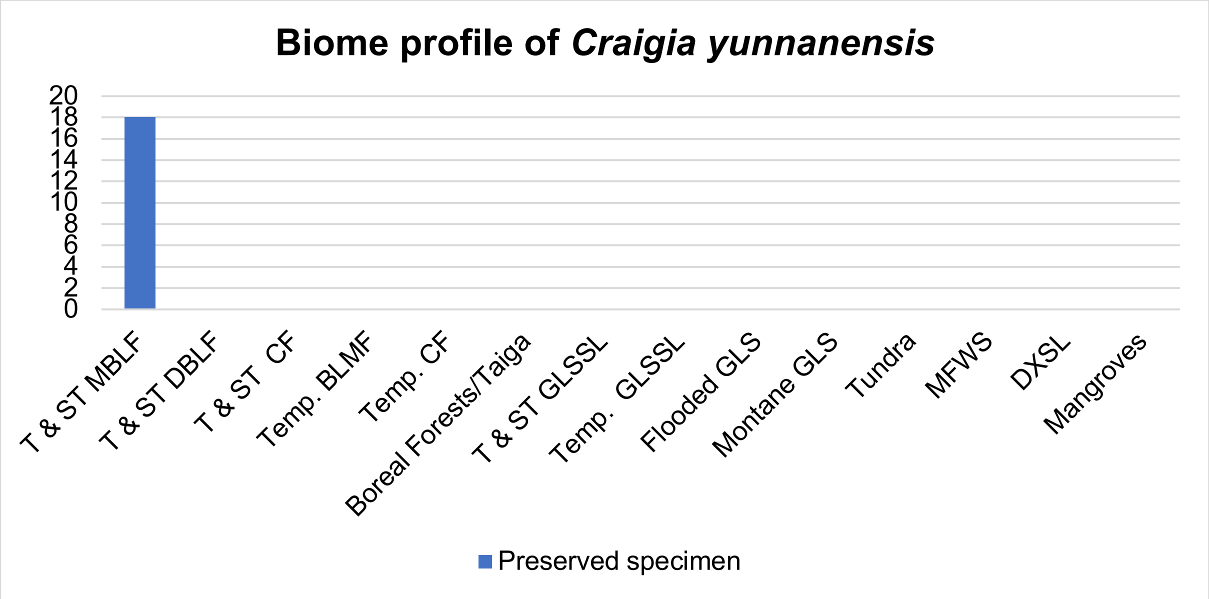

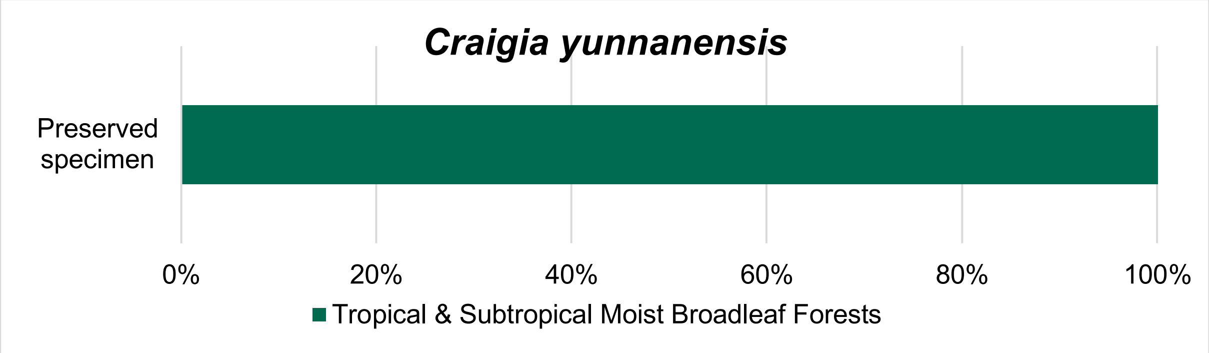

3.1.3. Climate graphs - based on 18 *Craigia yunnanensis* occurrences in GBIF

3.1.3.1. Mean monthly temperature (MMT) [°C]

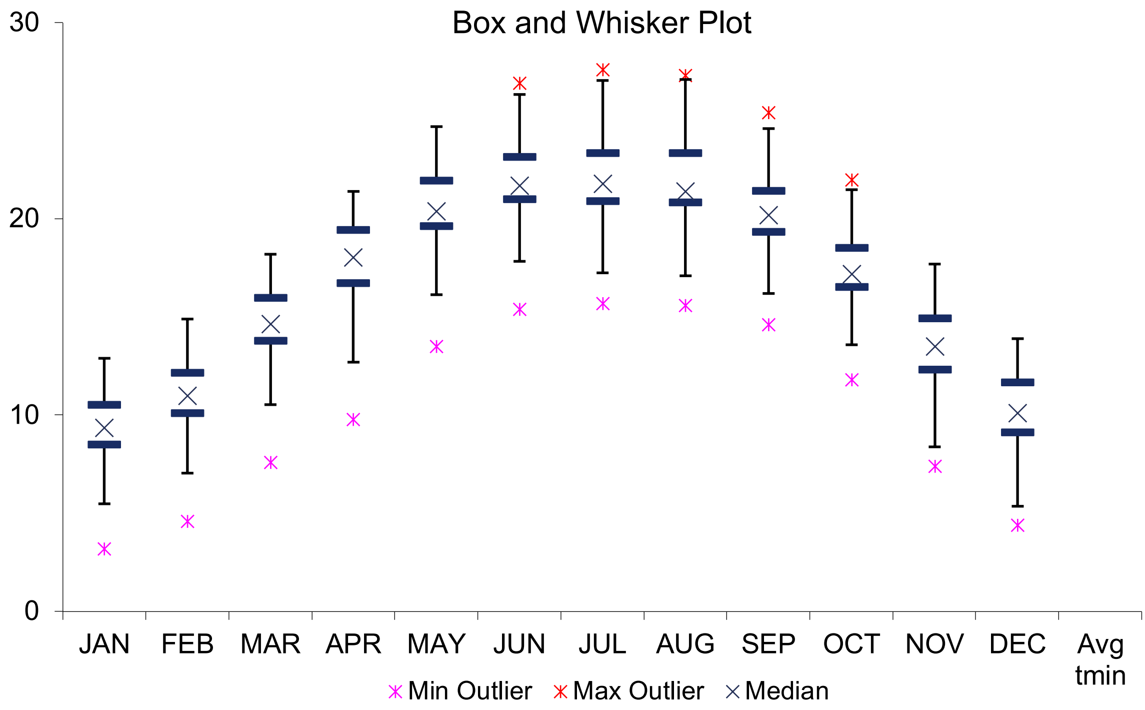

3.1.3.2. Minimum monthly temperature (MinMT) [°C]

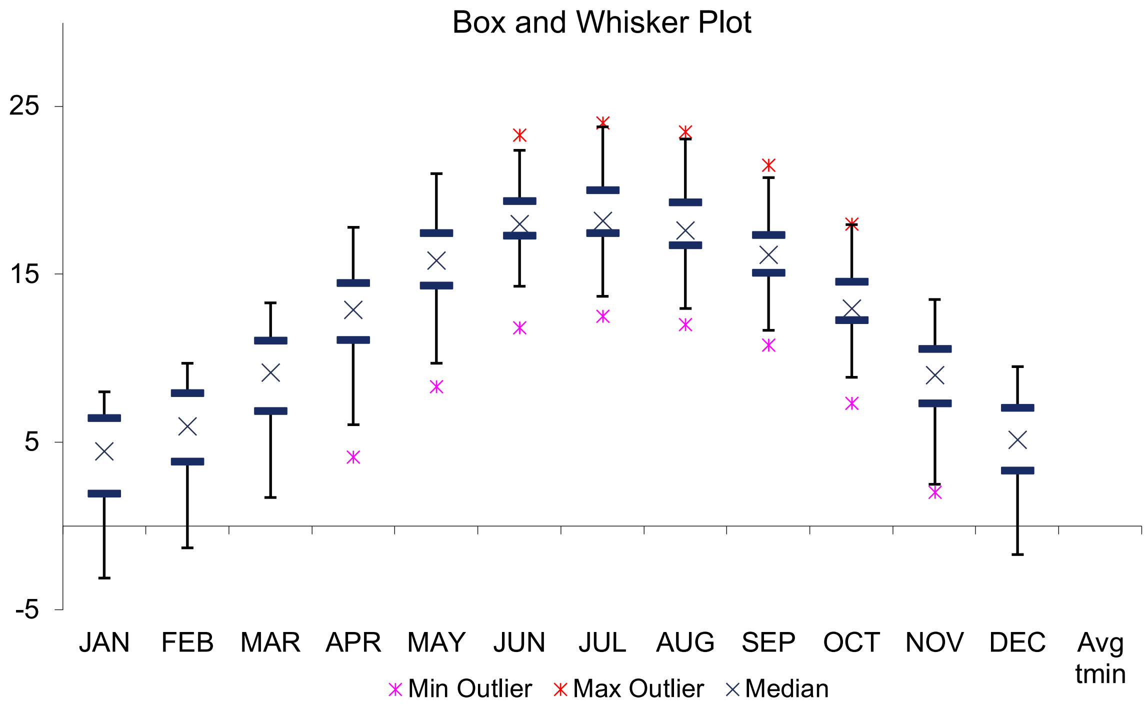

3.1.3.3. Mean monthly precipitation (MMP) [mm]

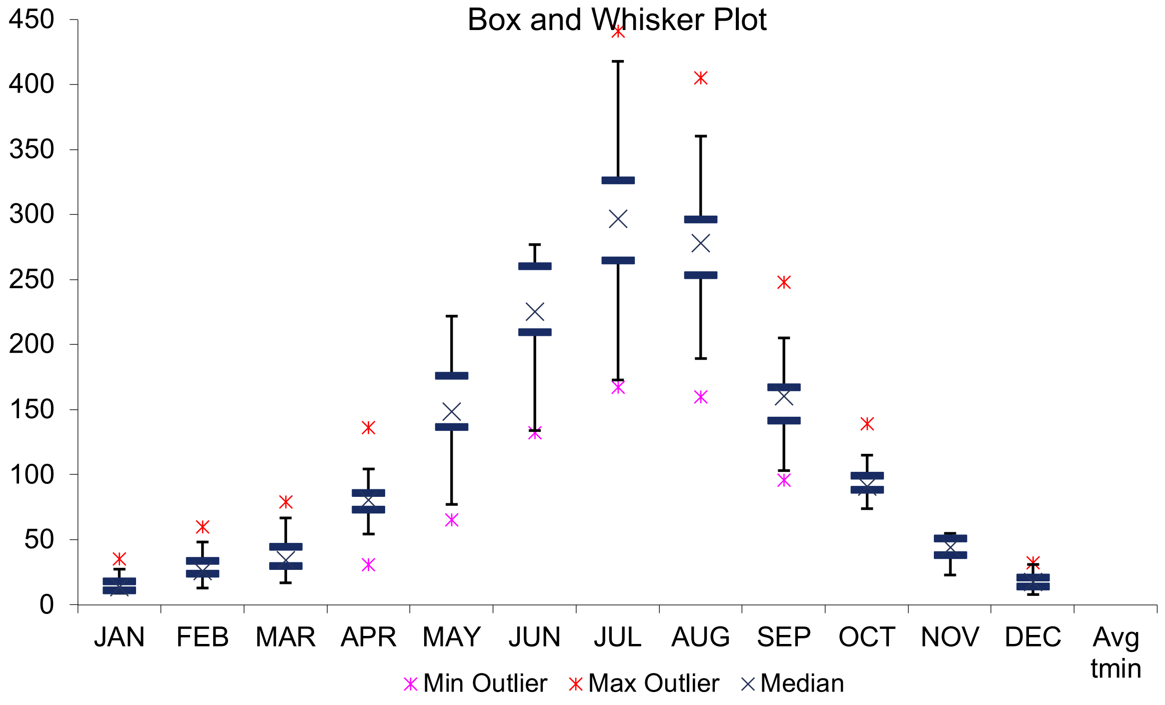

4. Genus *Mortoniodendron* Standl. et Steyerm., 1938

Mortoniodendron distribution map Köppen climate types

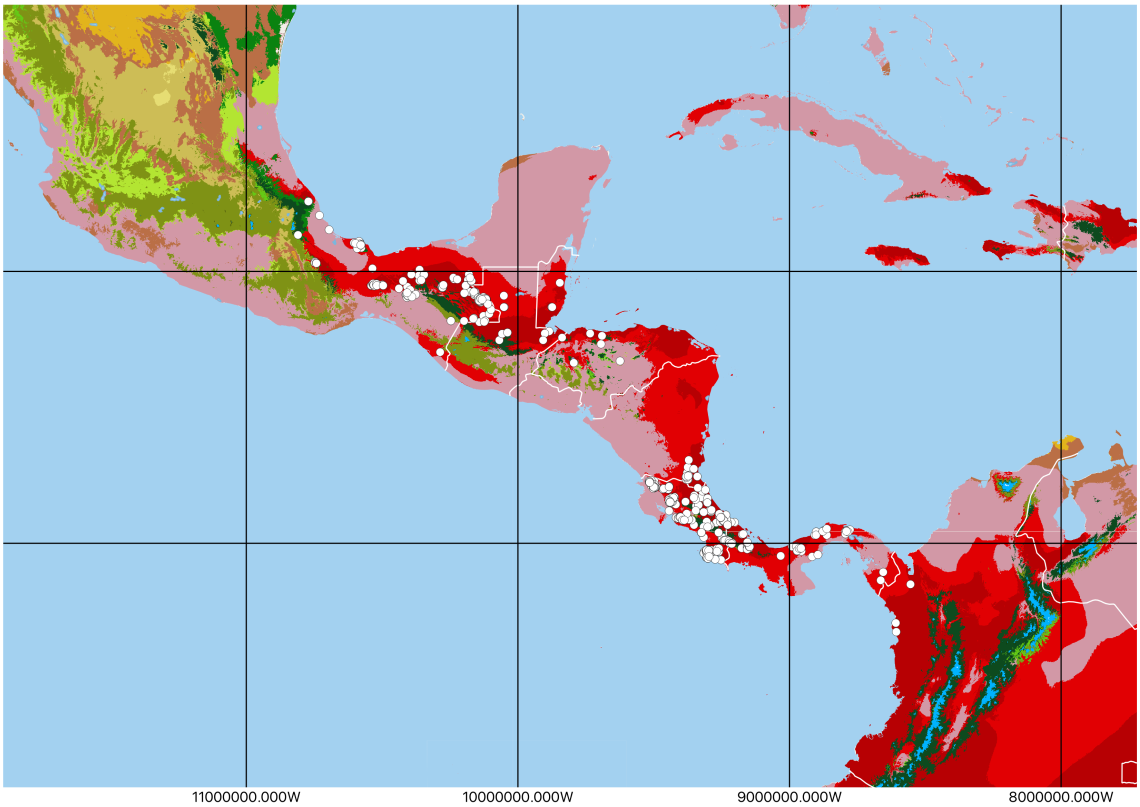

Mortoniodendron distribution map biomes

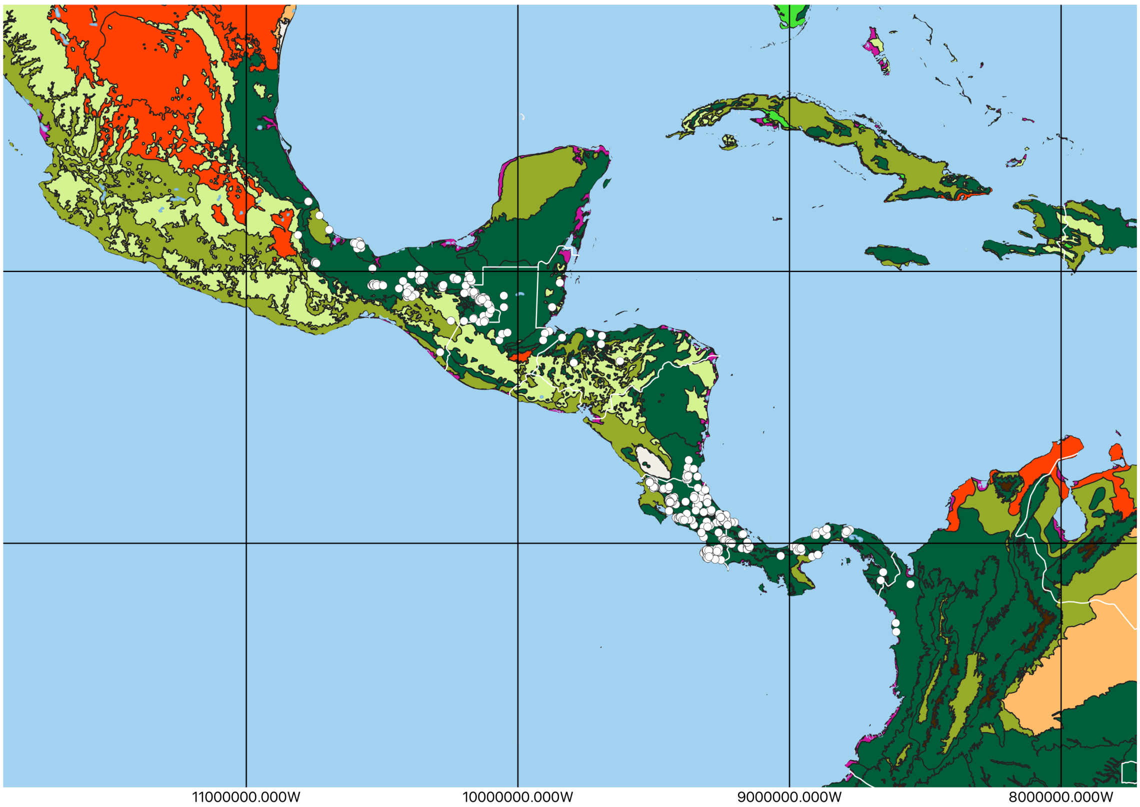

Mortoniodendron genus Köppen profile

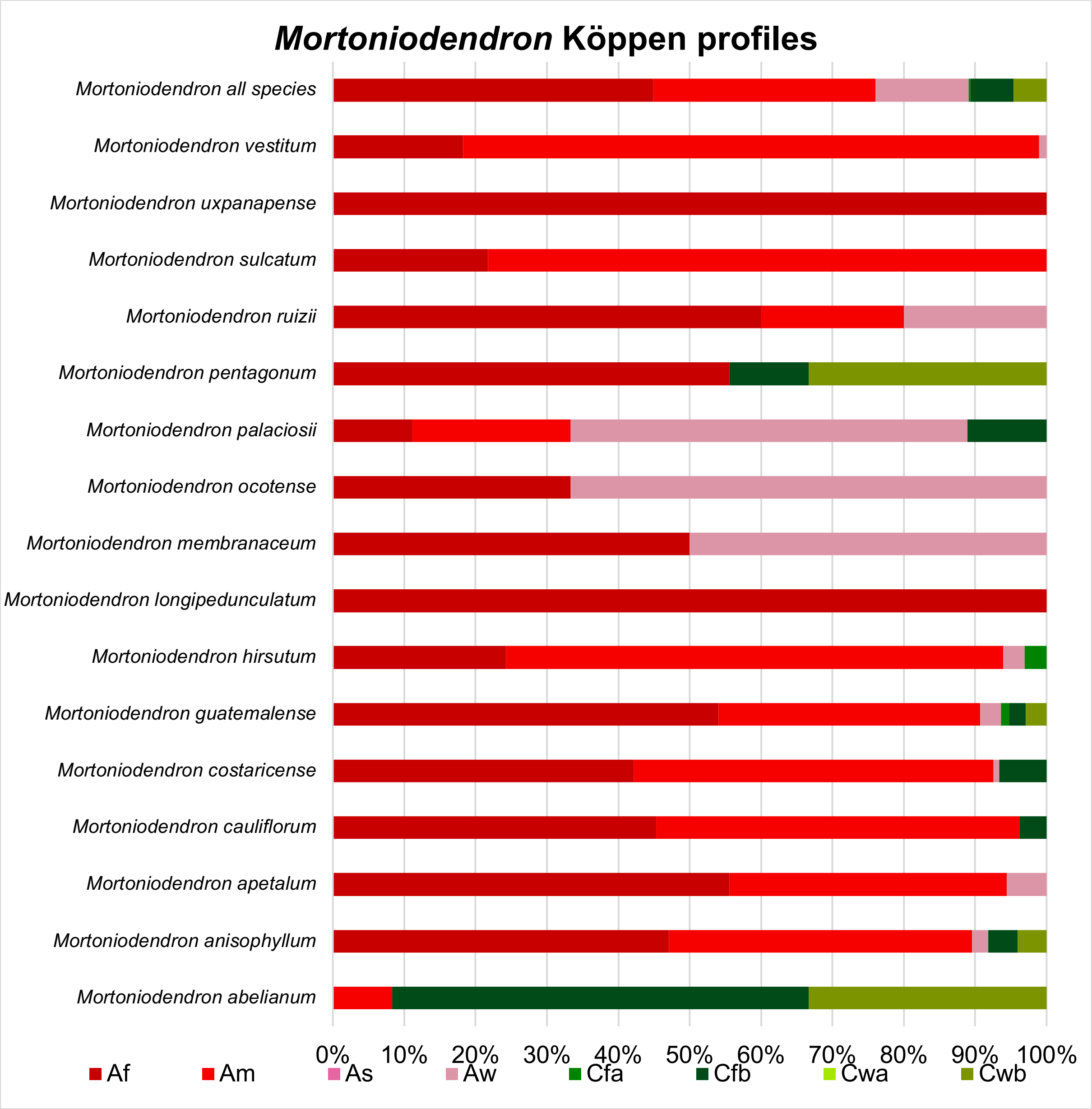

Mortoniodendron genus Biome profile

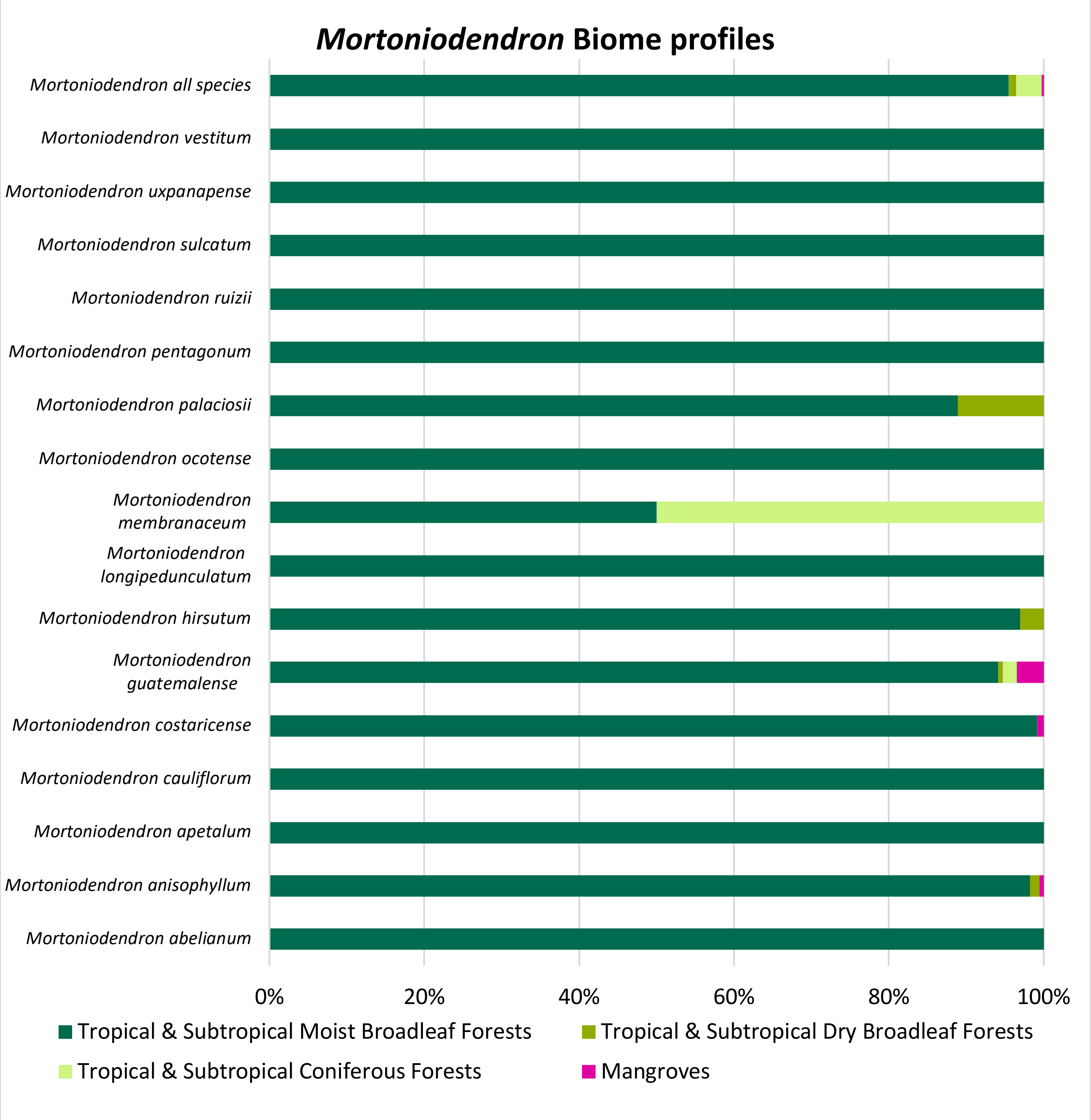

***Mortoniodendron* Climate graphs** (normalized, not including species with less than 10 occurrences) - based on *Mortoniodendron abelianum*, *M. anisophyllum*, *M. apetalum*, *M. cauliflorum*, *M. costaricense*, *M. guatemalense*, *M. hirsutum*, *M. sulcatum*, *M. vestitum*)

(MMT) [°C]

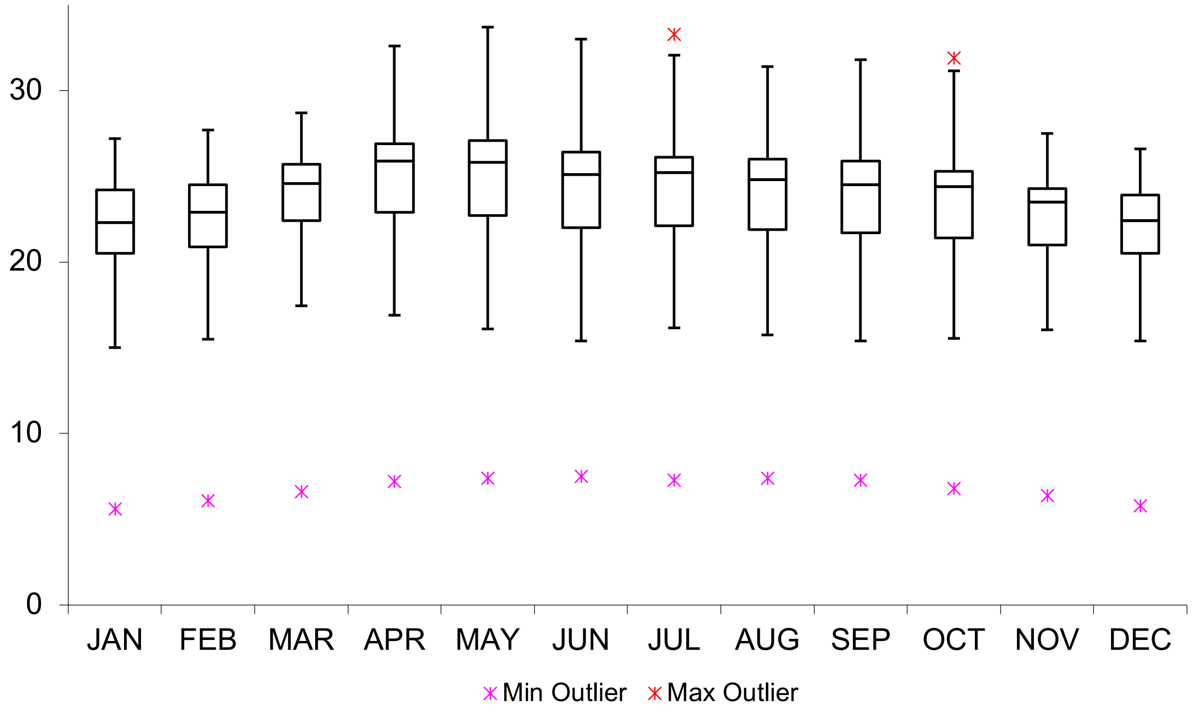

(MinMT) [°C]

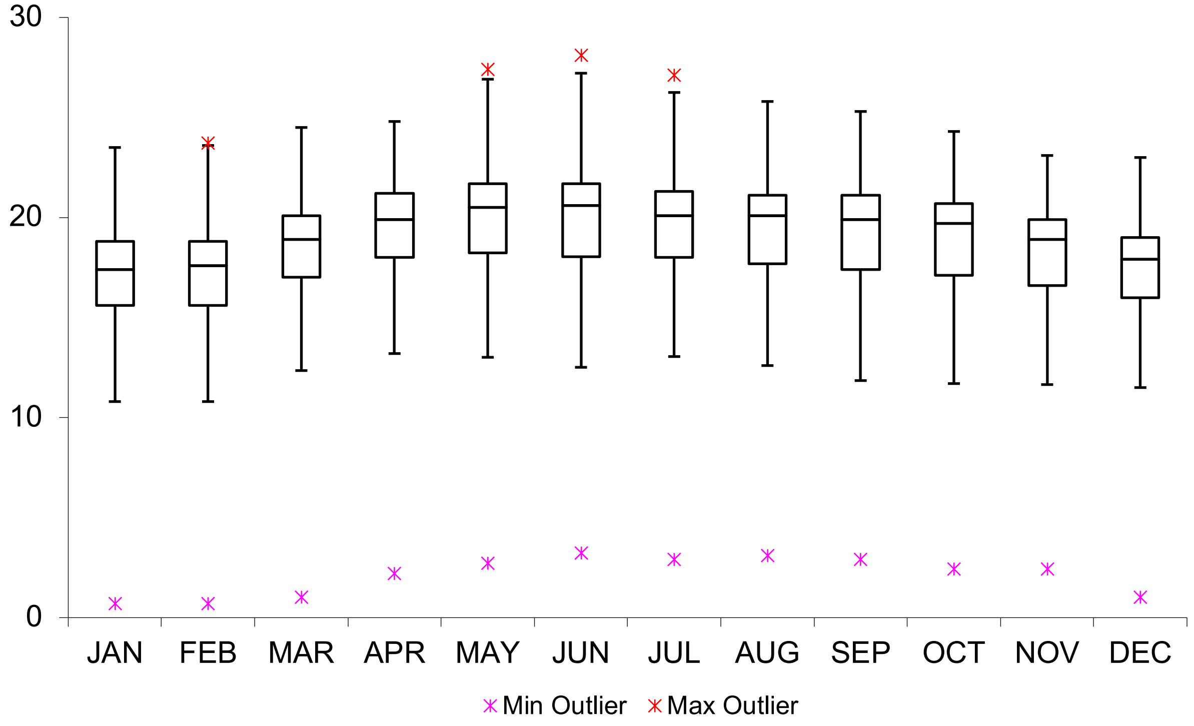

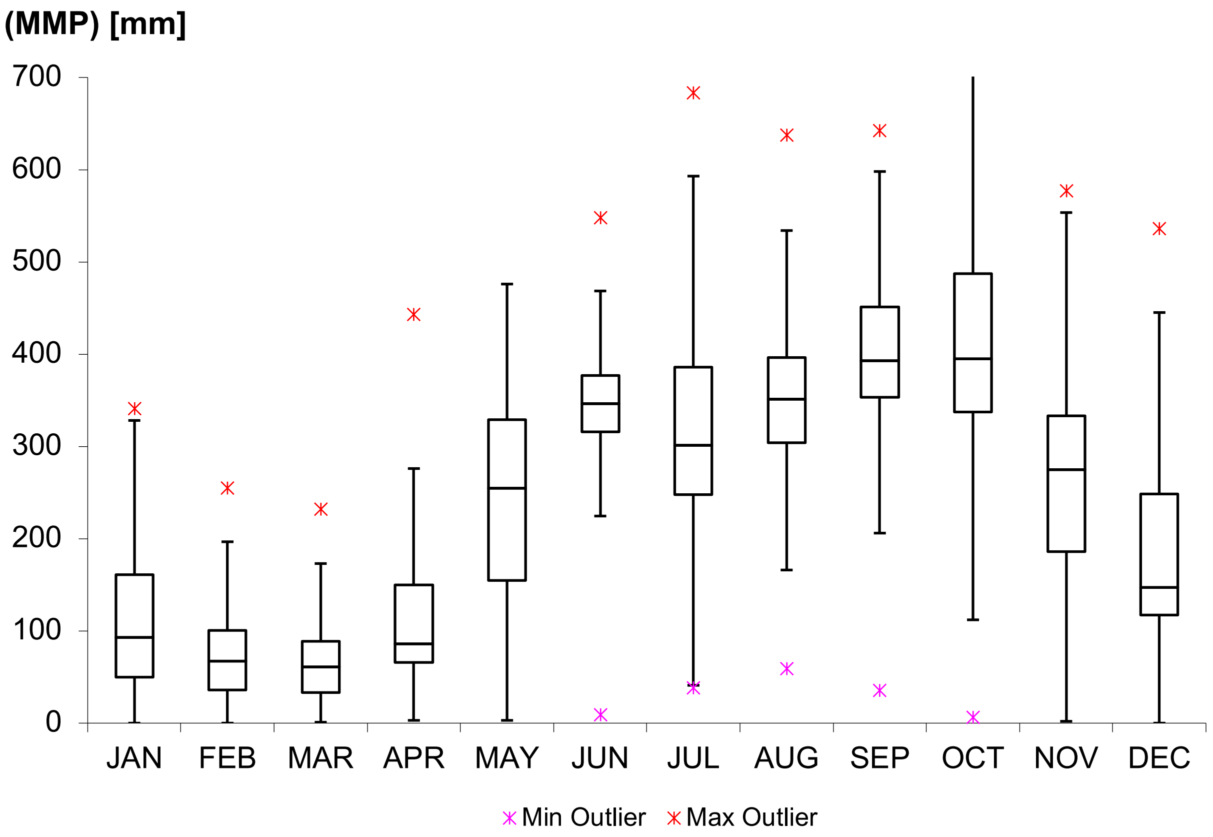
(MMP) [mm]
700
600
500
400
300
200
100
0
JAN
FEB
MAR
APR
MAY
JUN
JUL
AUG
SEP
OCT
NOV
DEC
Min Outlier
Max Outlier

4.1. Species *Mortoniodendron abelianum* Al.Rodr., 2004

4.1.1. Köppen profile, distribution, and climate map – GBIF occurrences of *Mortoniodendron abelianum*; herbarium specimens (n = 24).

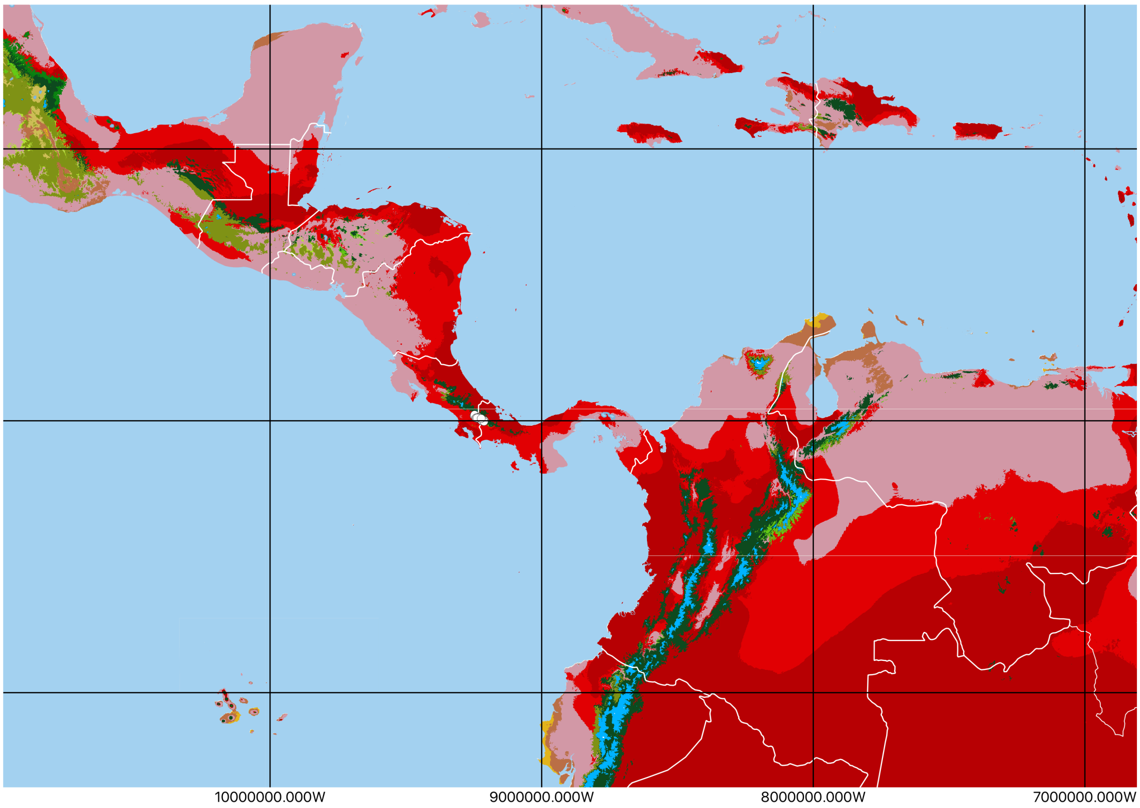

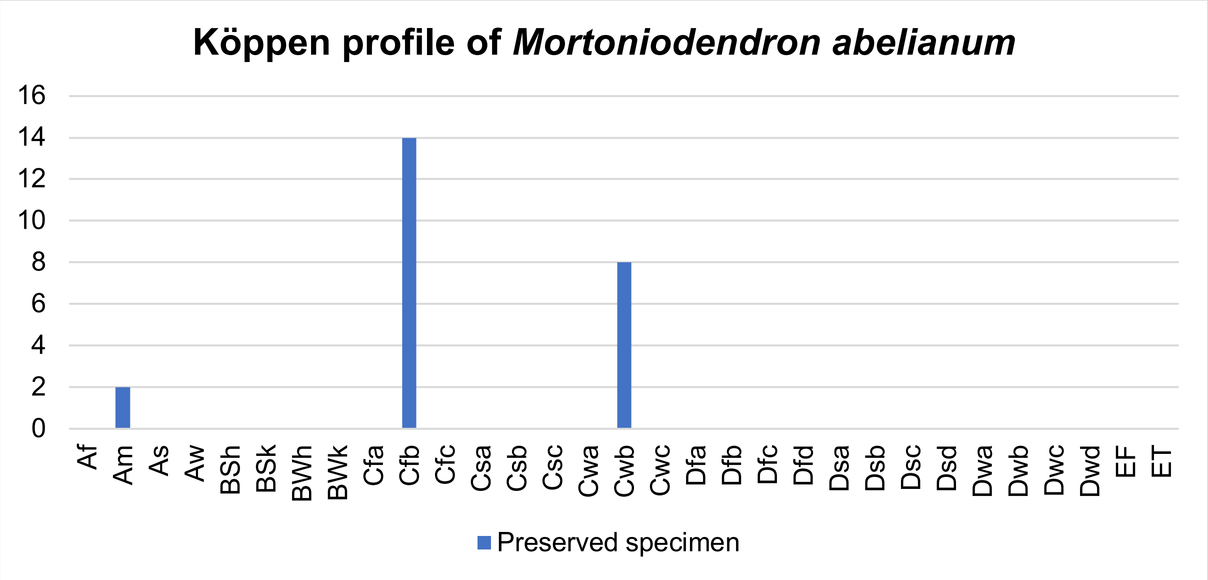

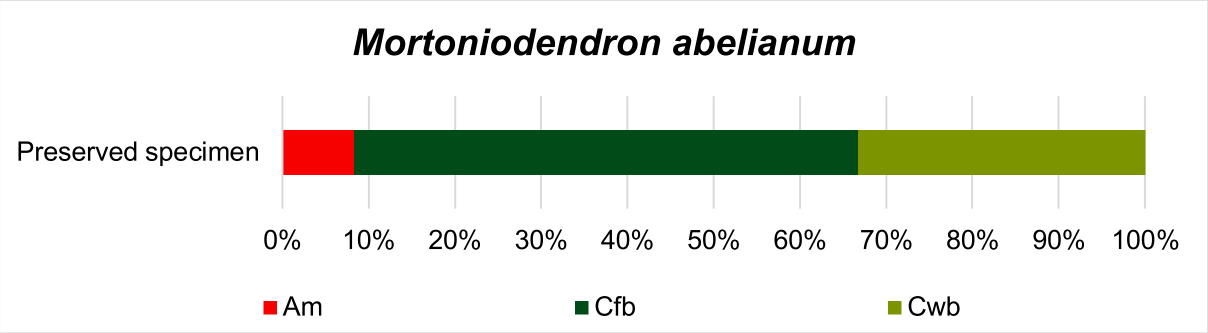

4.1.2. Biome profile, distribution, and biome map – GBIF occurrences of *Mortoniodendron abelianum*; herbarium specimens (n = 24).

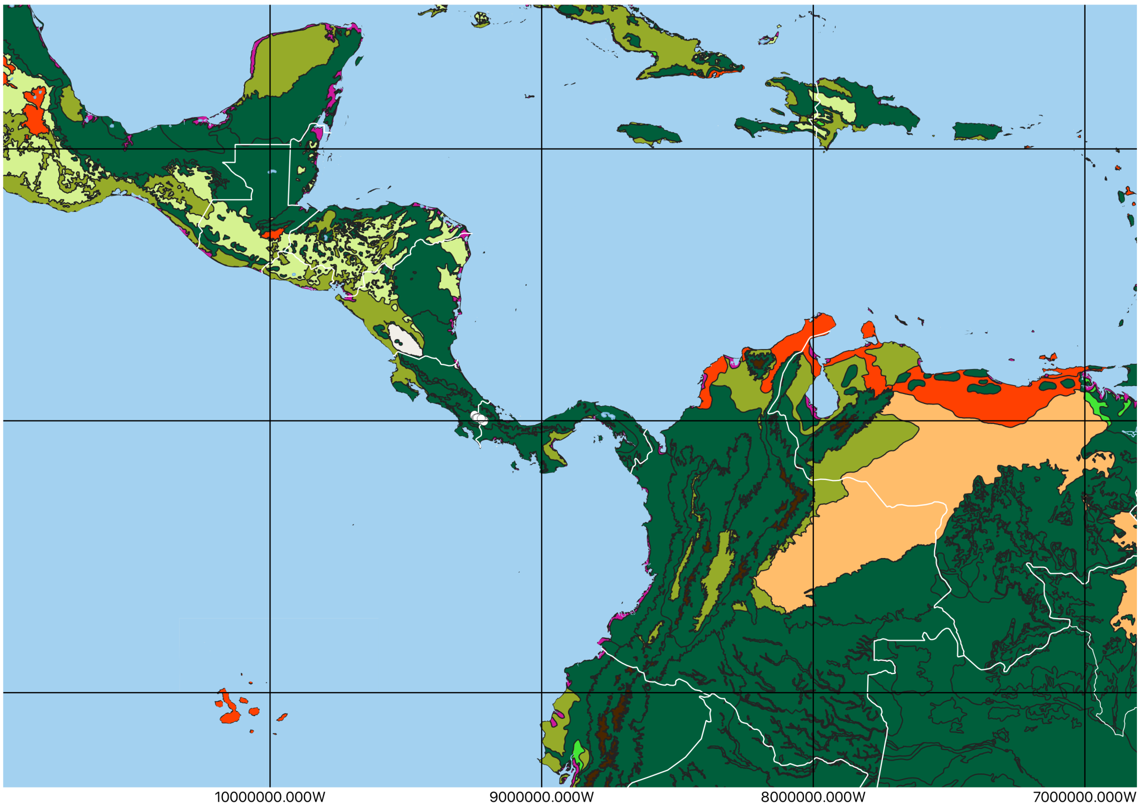

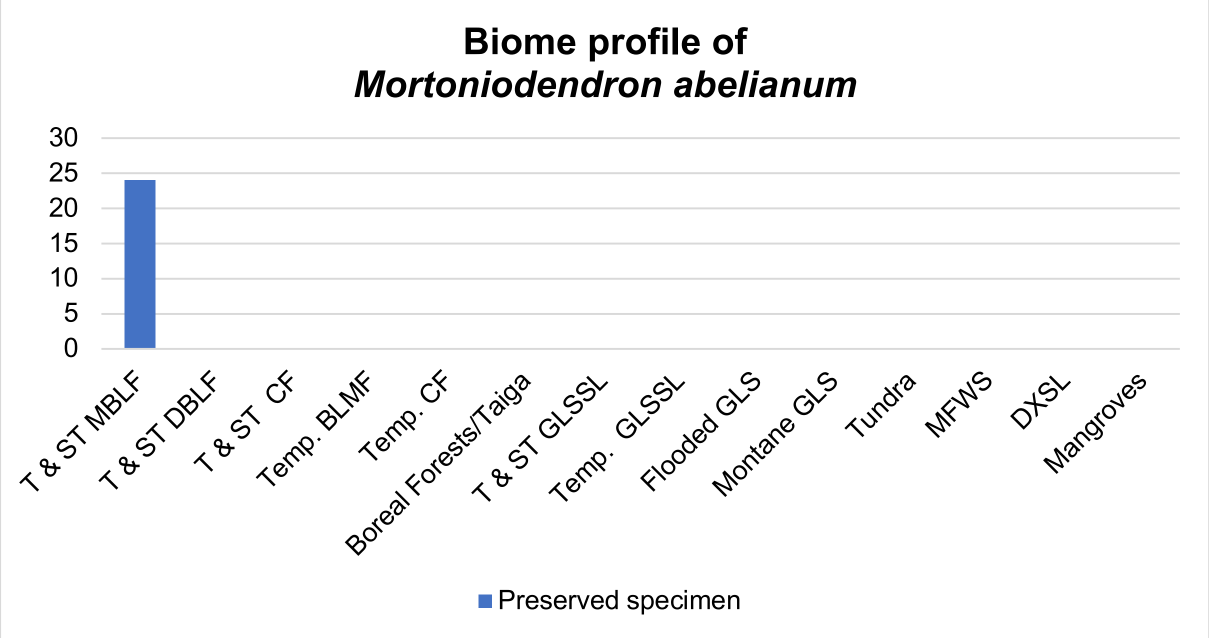

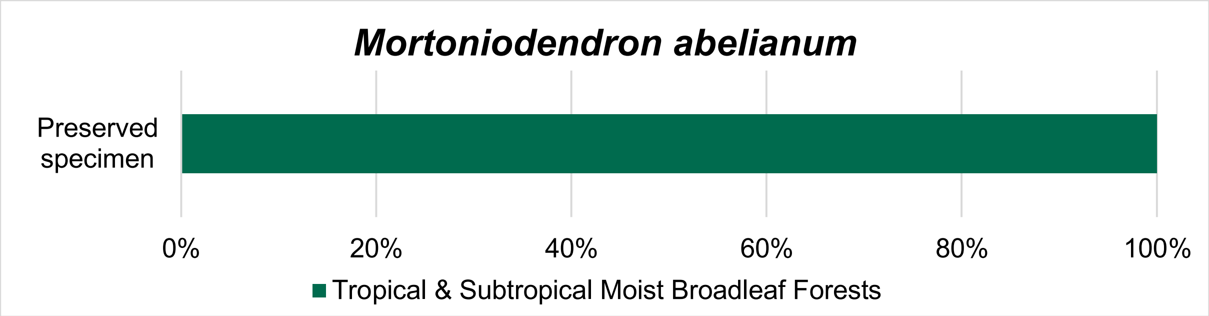

4.1.3. Climate graphs - based on 24 *Mortoniodendron abelianum* occurrences in GBIF

4.1.3.1. Mean monthly temperature (MMT) [°C]

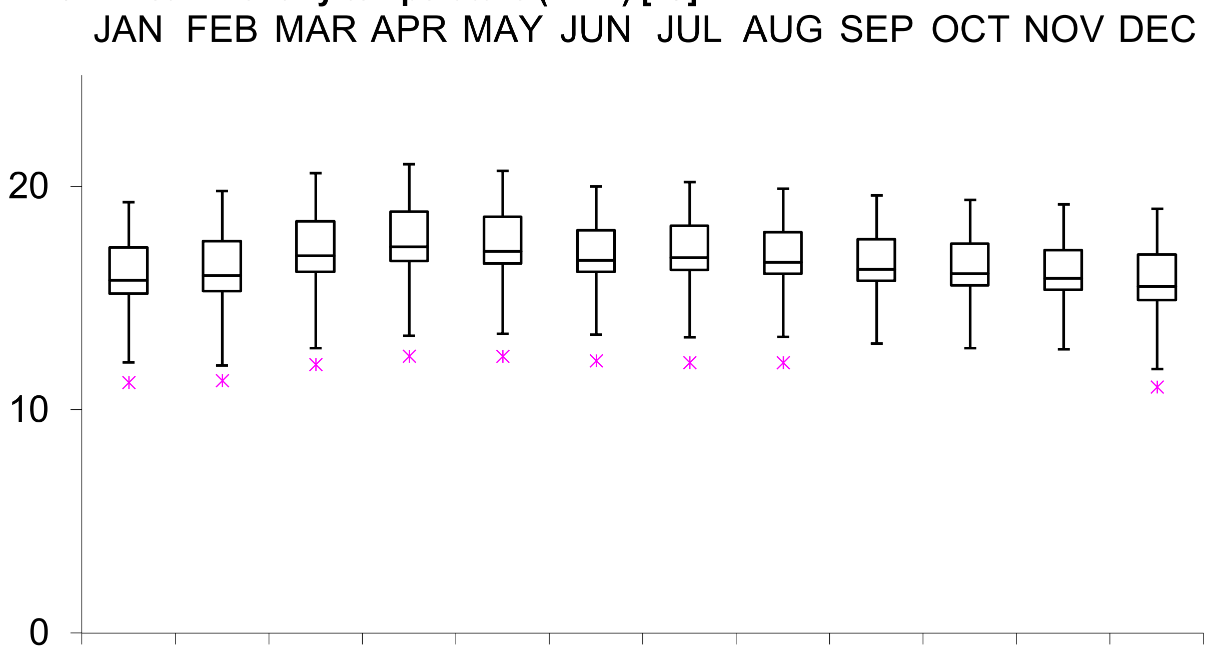

4.1.3.2. Minimum monthly temperature (MinMT) [°C]

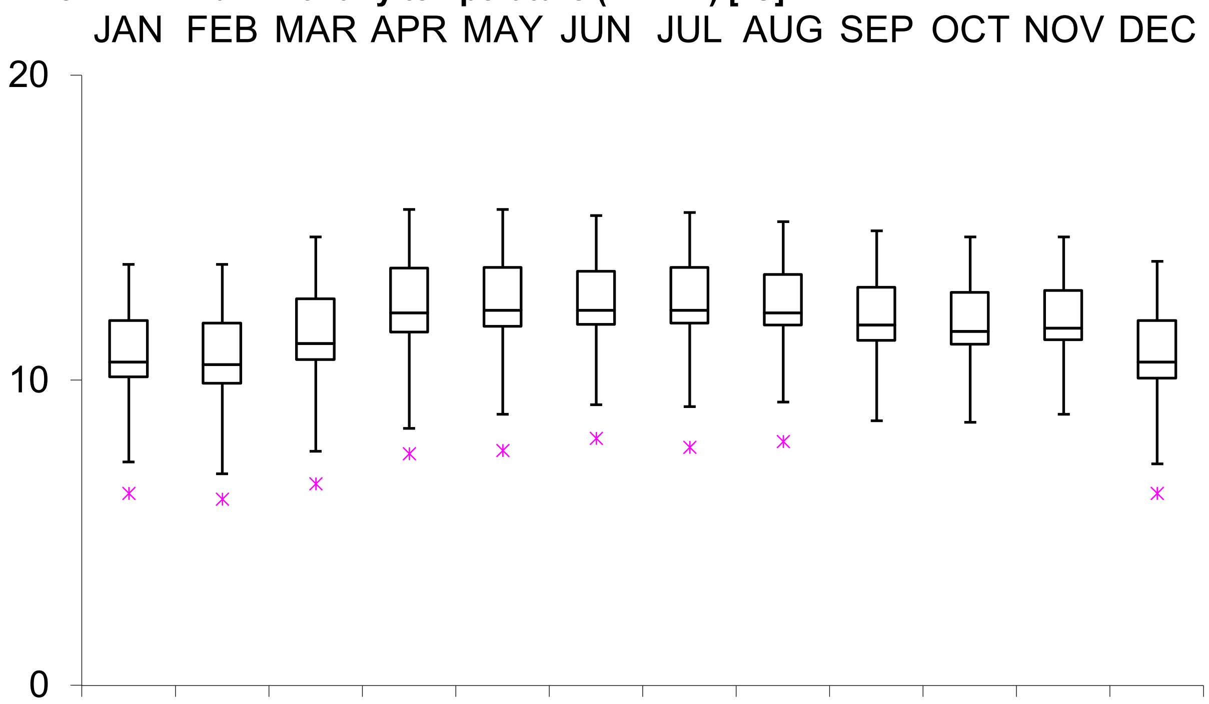

4.1.3.3. Mean monthly precipitation (MMP) [mm]

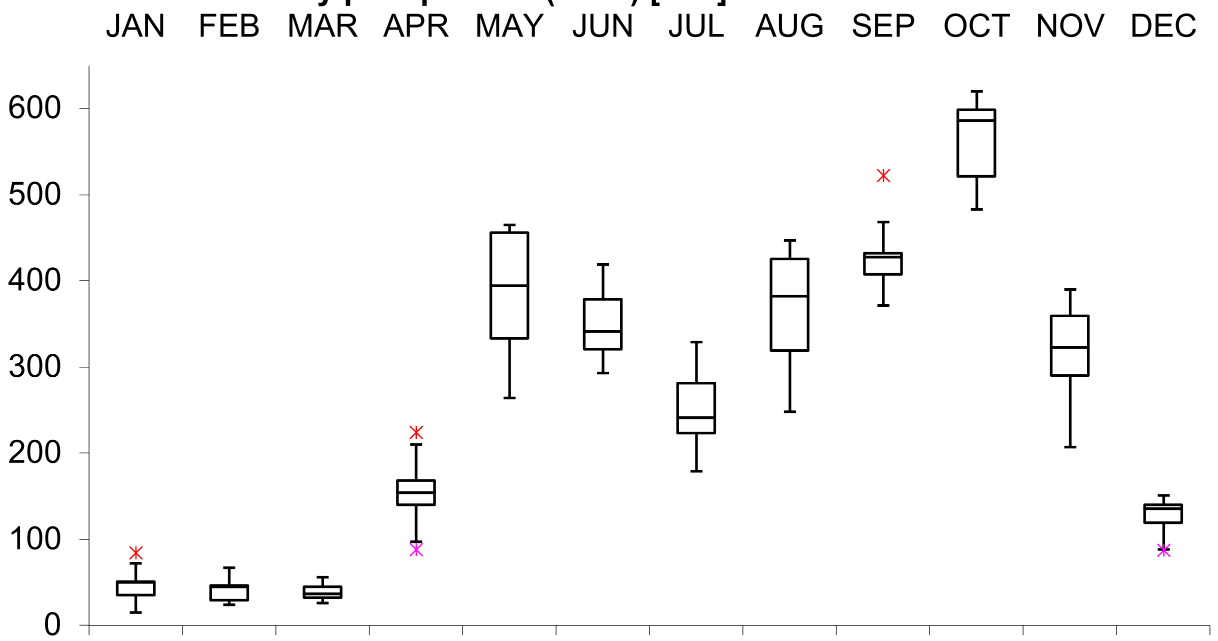

4.2. Species *Mortoniodendron anisophyllum* (Standl.) Standl. et Steyerm., 1938
4.2.1. Köppen profile, distribution, and climate map – GBIF occurrences of *Mortoniodendron anisophyllum*; excluding duplicate occurrences (n = 172), herbarium specimens (n = 168).

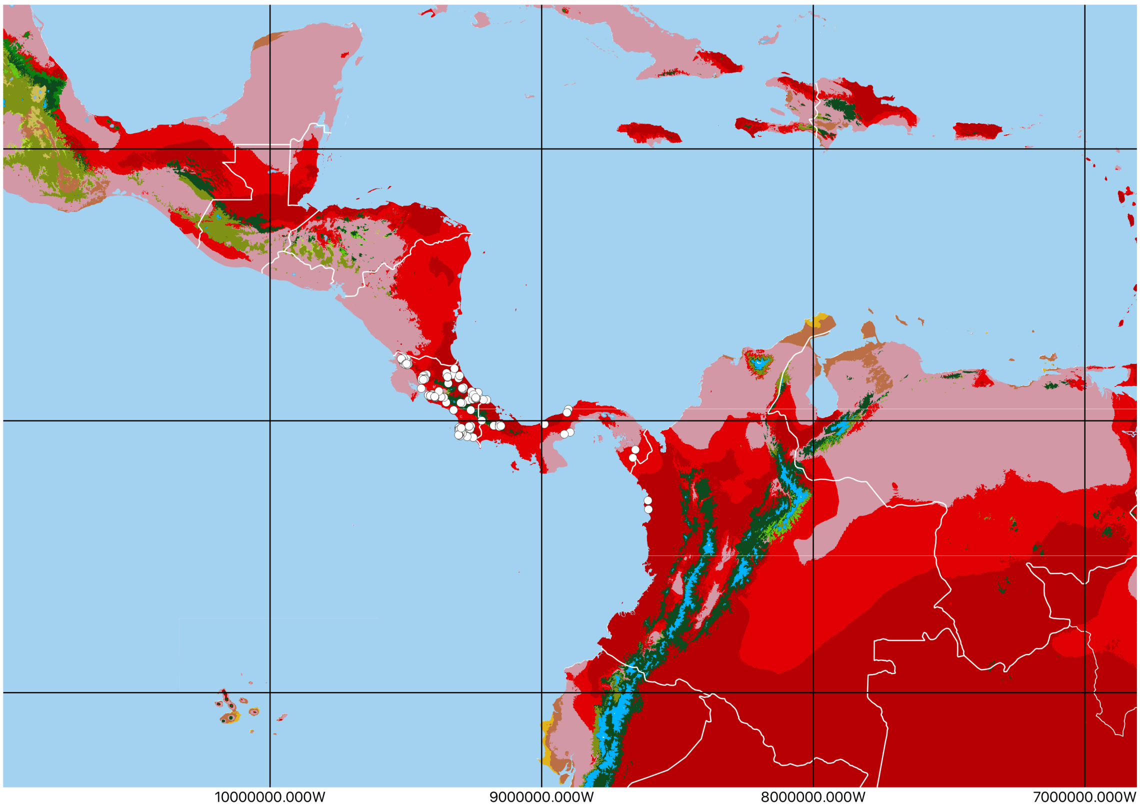

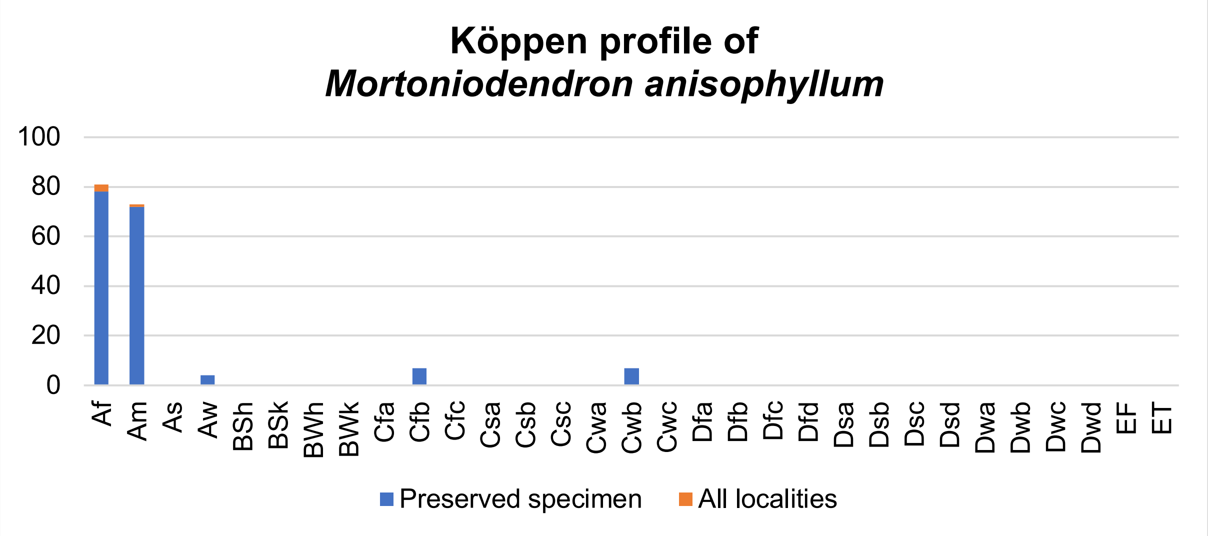

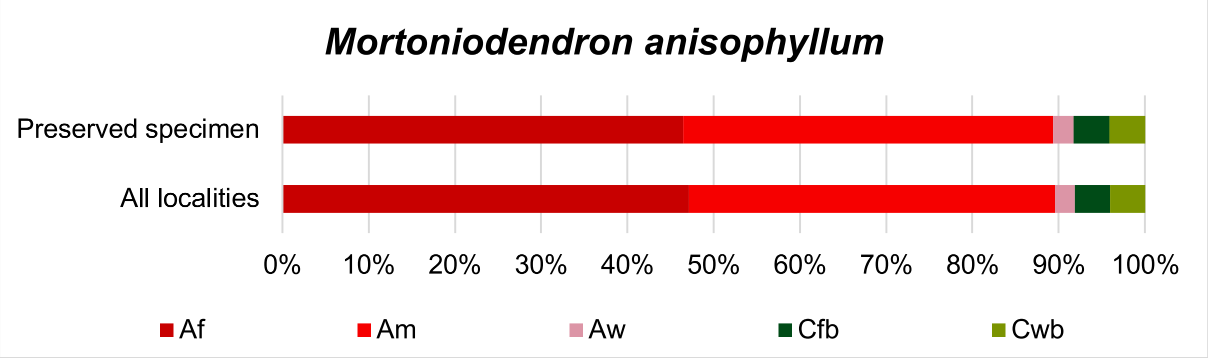

4.2.2. Biome profile, distribution, and biome map – *Mortoniodendron anisophyllum*; excluding duplicate occurrences (n = 172), herbarium specimens (n = 168).

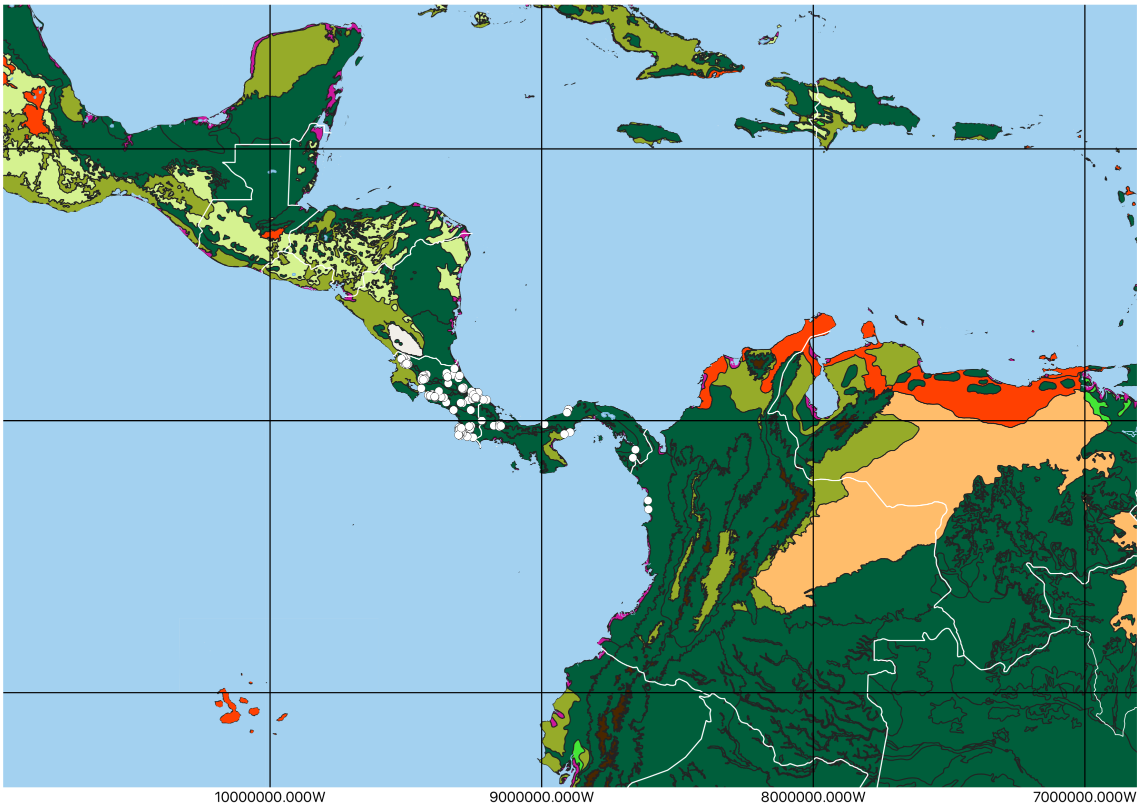

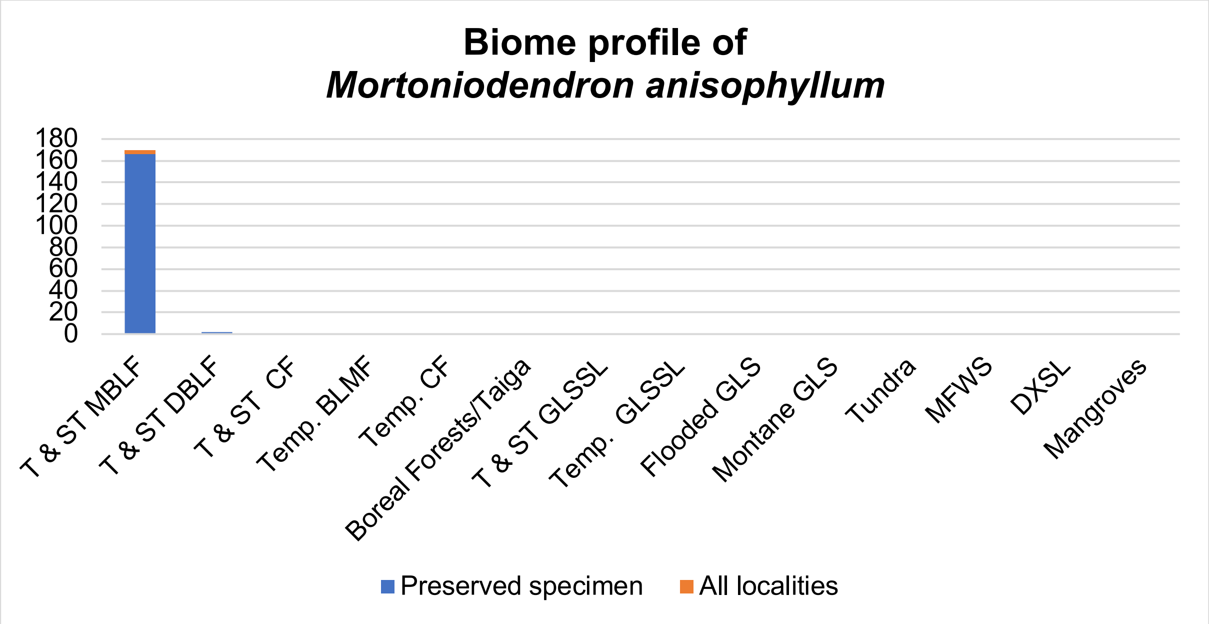

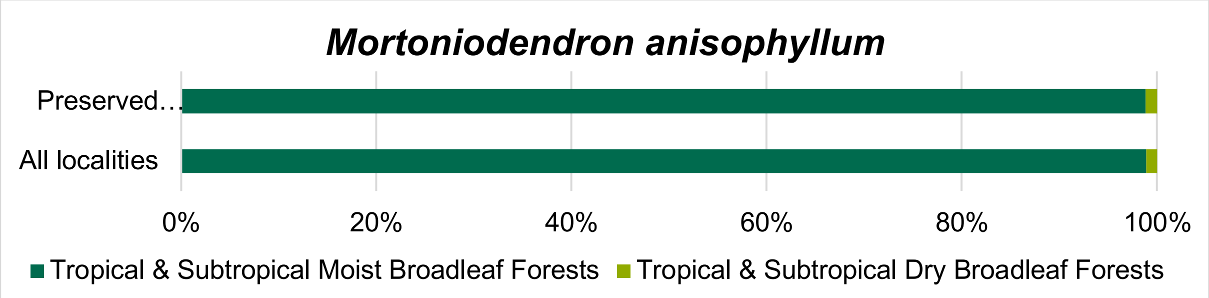

4.2.3. Climate graphs - based on 170 *Mortoniodendron anisophyllum* occurrences in GBIF

4.2.3.1. MMT [°C]

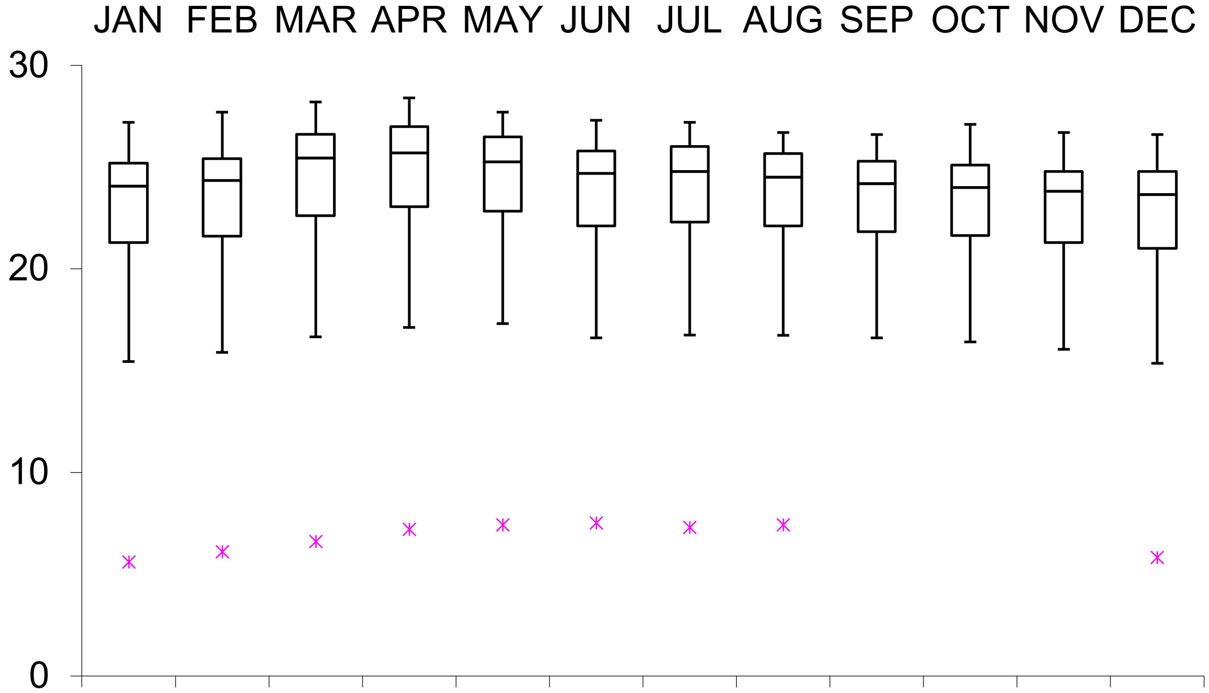

4.2.3.2. MinMT [°C]

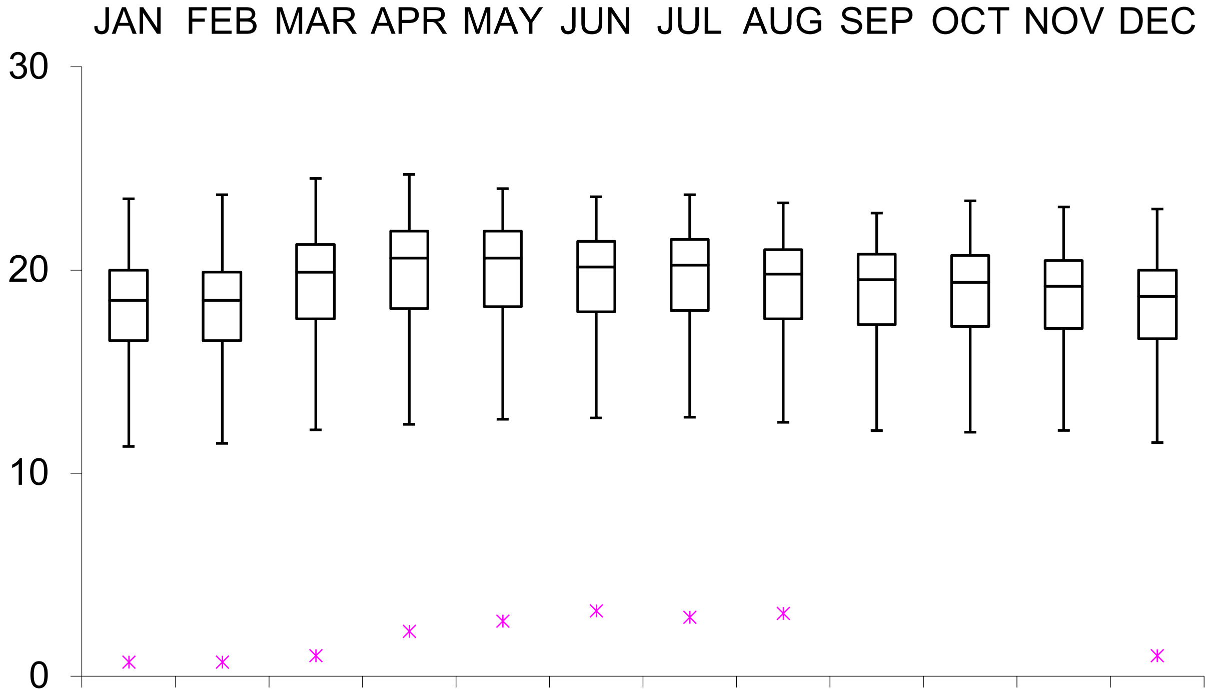

4.2.3.3. MMP [mm]

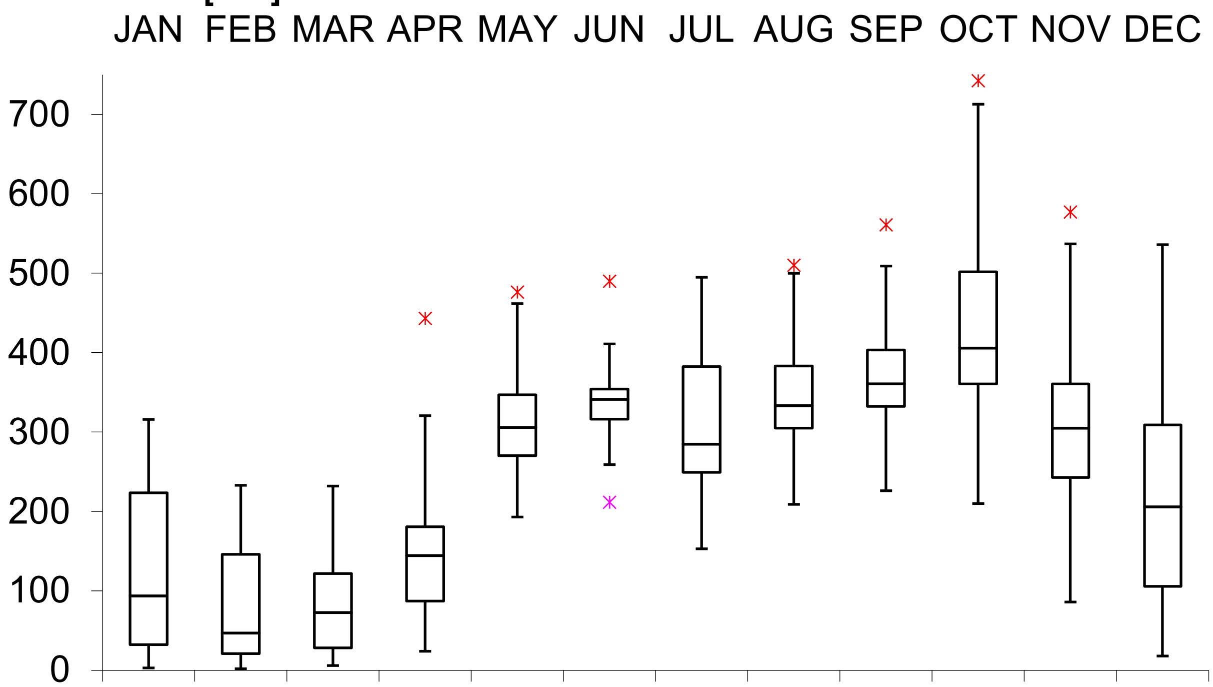
JAN FEB MAR APR MAY JUN JUL AUG SEP OCT NOV DEC
700
600
500
400
300
200
100
0

4.3. Species *Mortoniodendron apetalum* Al.Rodr., 2004

4.3.1. Köppen profile, distribution, and climate map – GBIF occurrences of *Mortoniodendron apetalum*; herbarium specimens (n = 18).

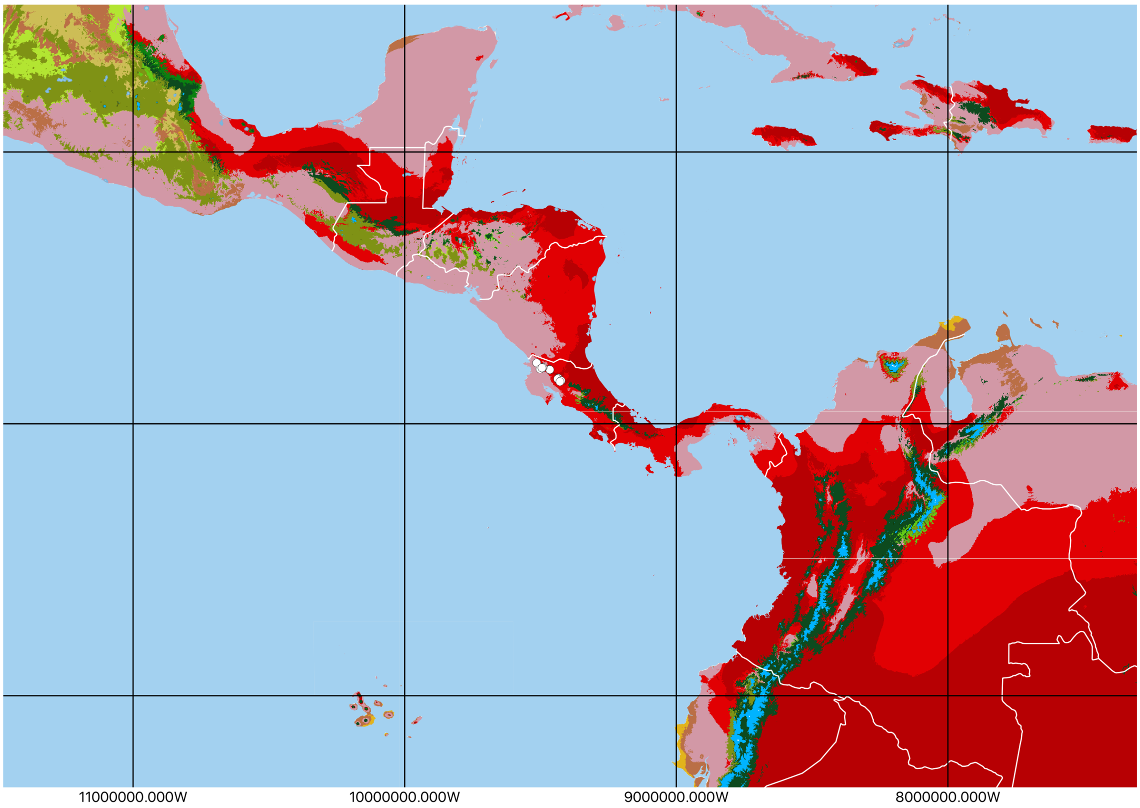

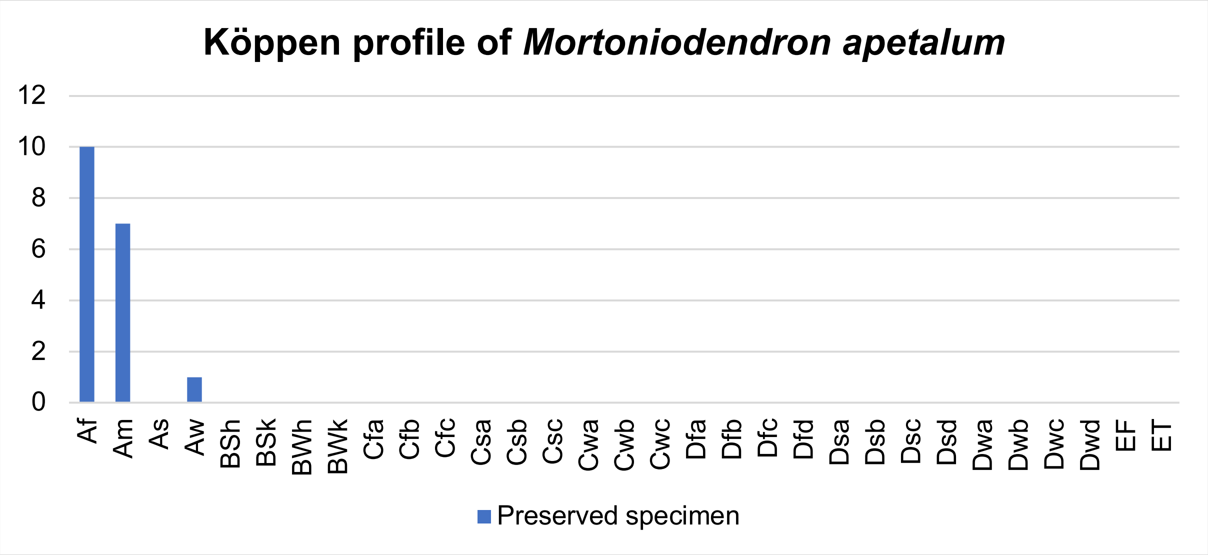

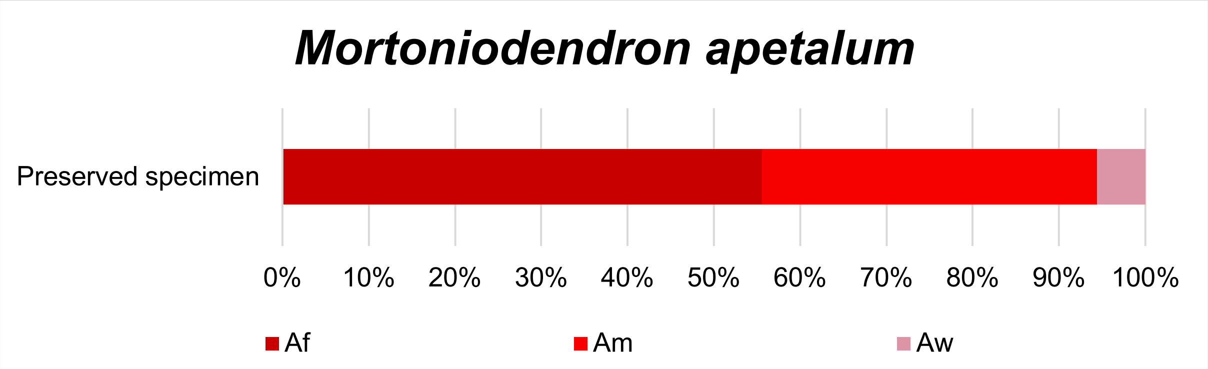

4.3.2. Biome profile, distribution, and biome map – GBIF occurrences of *Mortoniodendron apetalum*; herbarium specimens (n = 18).

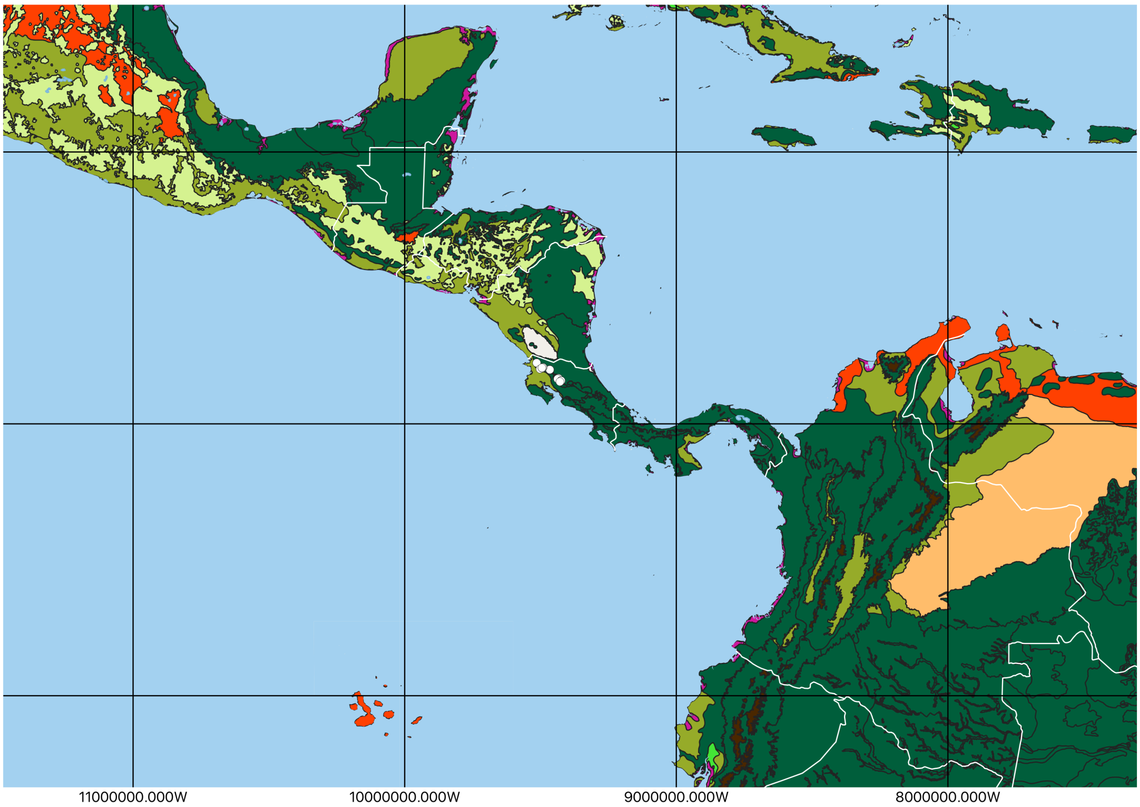

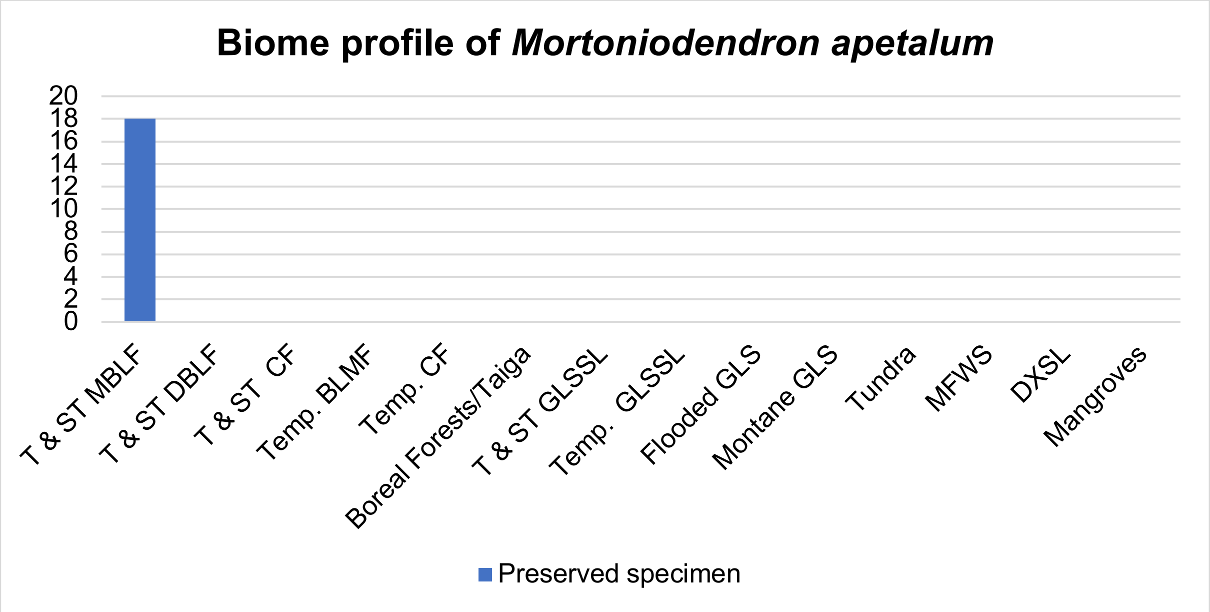

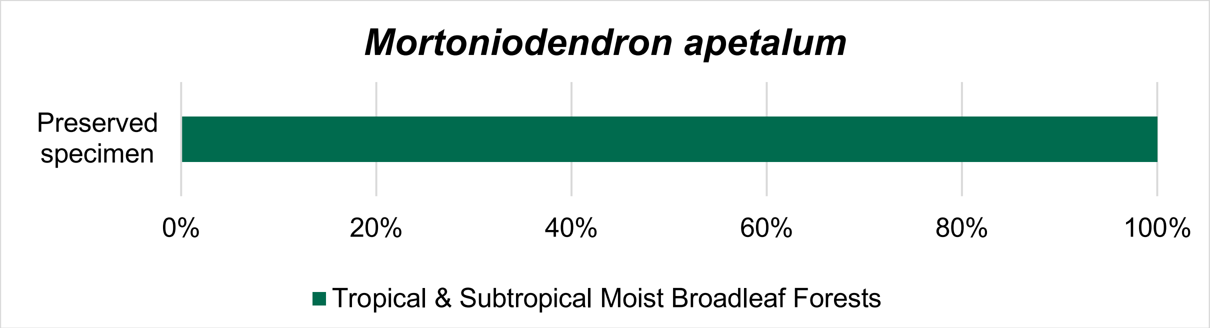

4.3.3. Climate graphs - based on 18 *Mortoniodendron apetalum* occurrences in GBIF

4.3.3.1. MMT [°C]

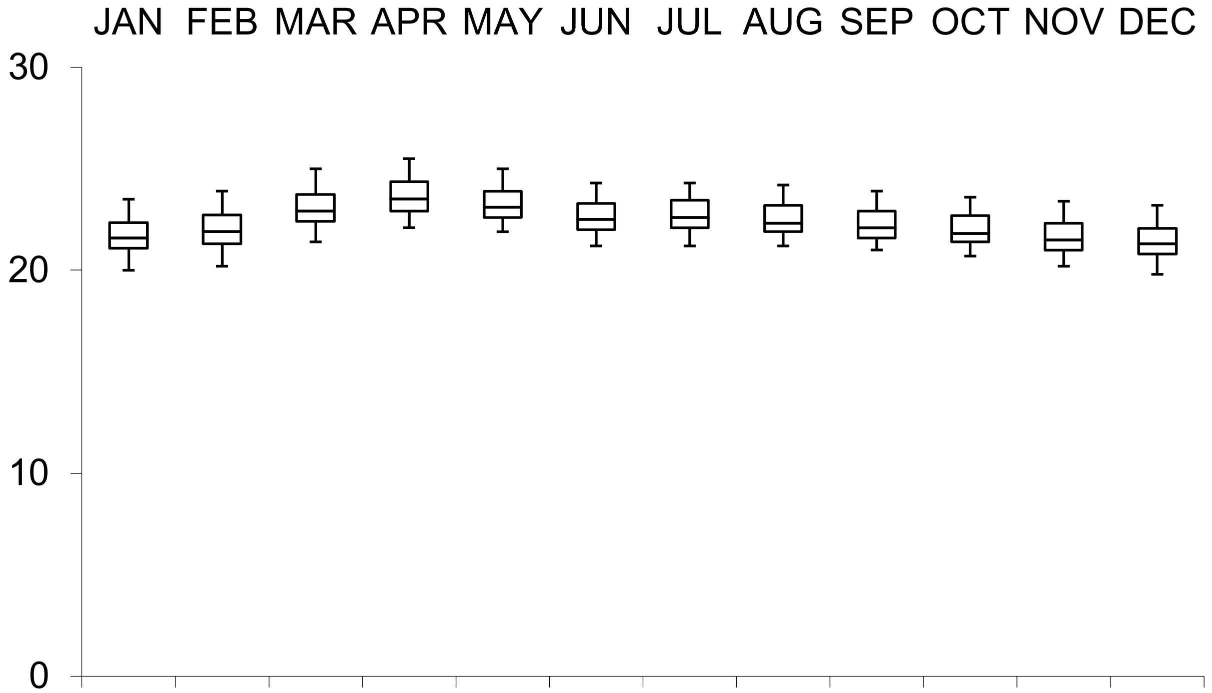

4.3.3.2. MinMT [°C]

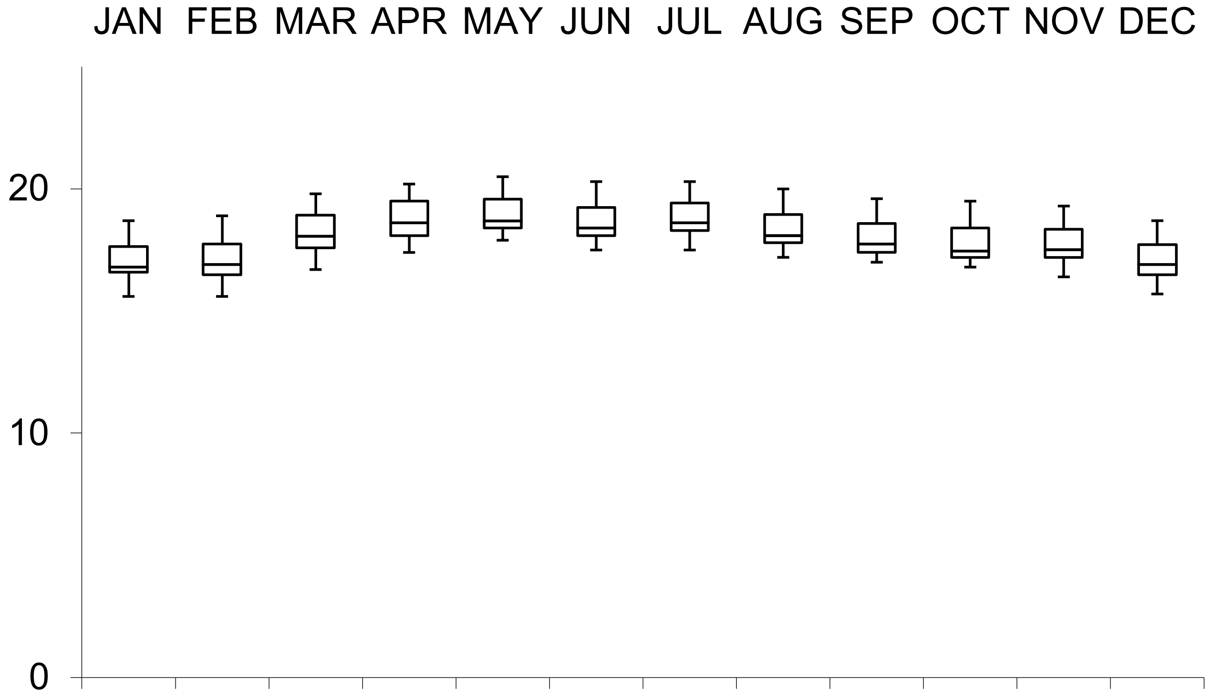

4.3.3.3. MMP [mm]

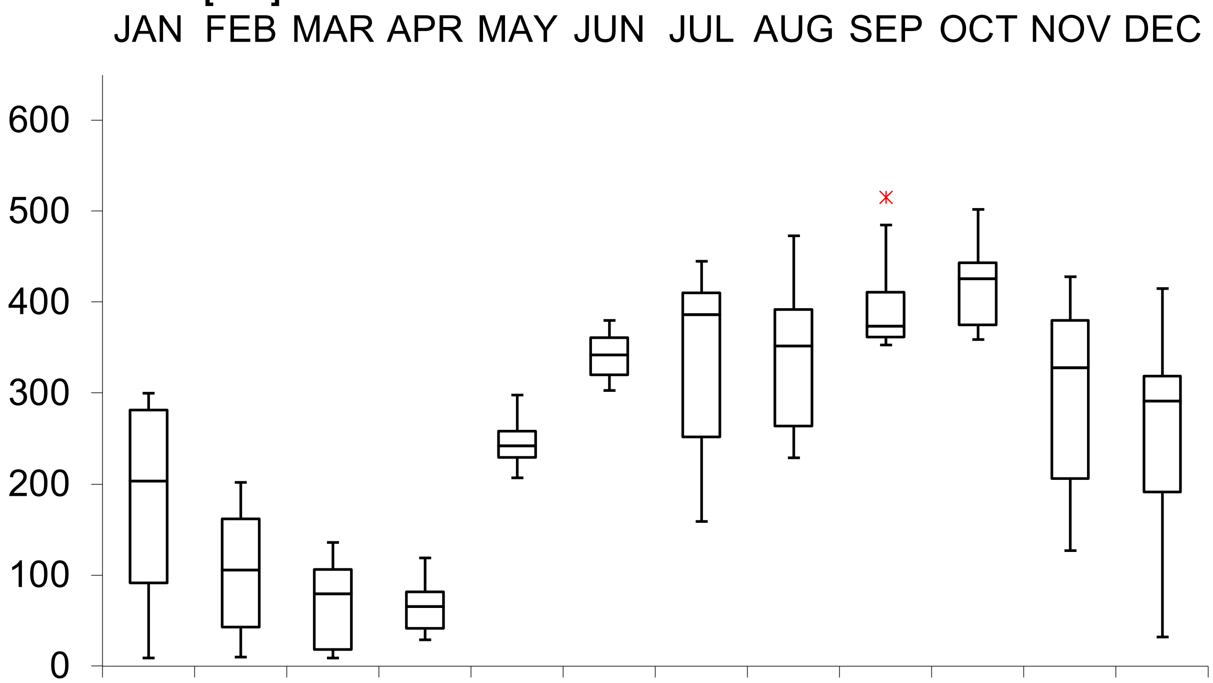

JAN FEB MAR APR MAY JUN JUL AUG SEP OCT NOV DEC
600
500
400
300
200
100
0

4.4. Species *Mortoniodendron cauliflorum* Al.Rodr., 2004

4.4.1. Köppen profile, distribution, and climate map – GBIF specimens of *Mortoniodendron cauliflorum*; excluding duplicate occurrences (n = 52), herbarium specimens (n = 49).

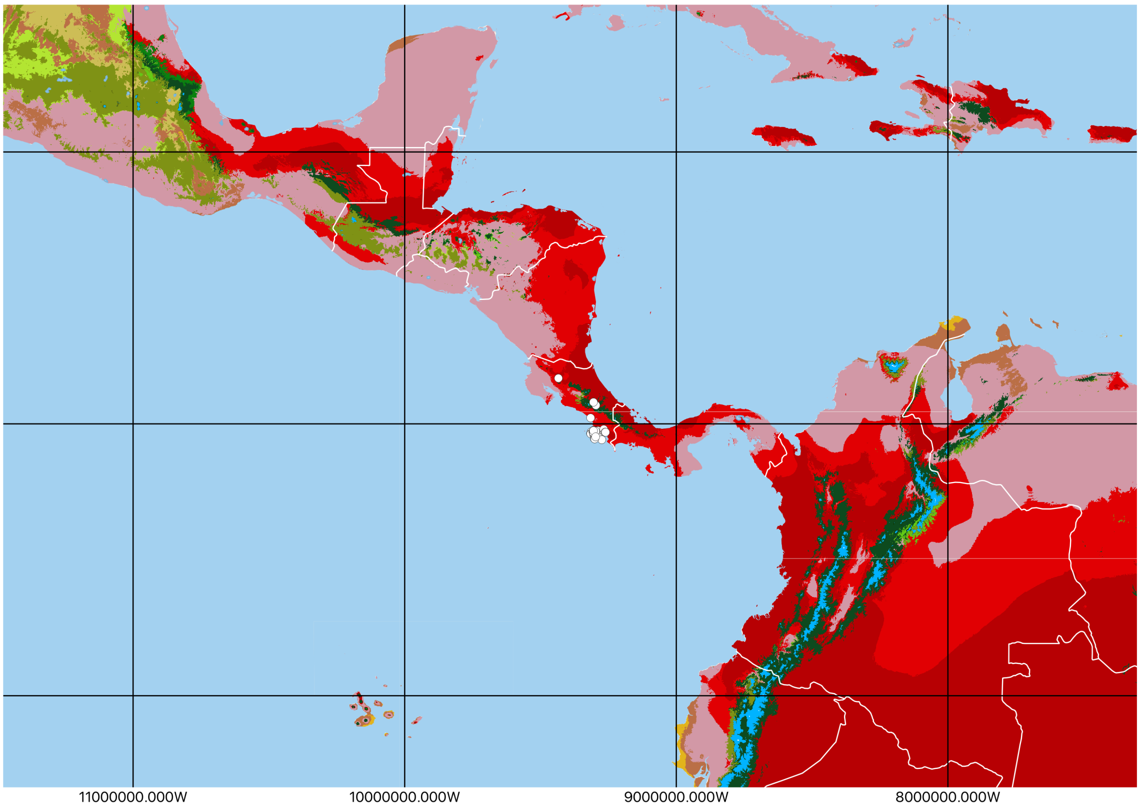

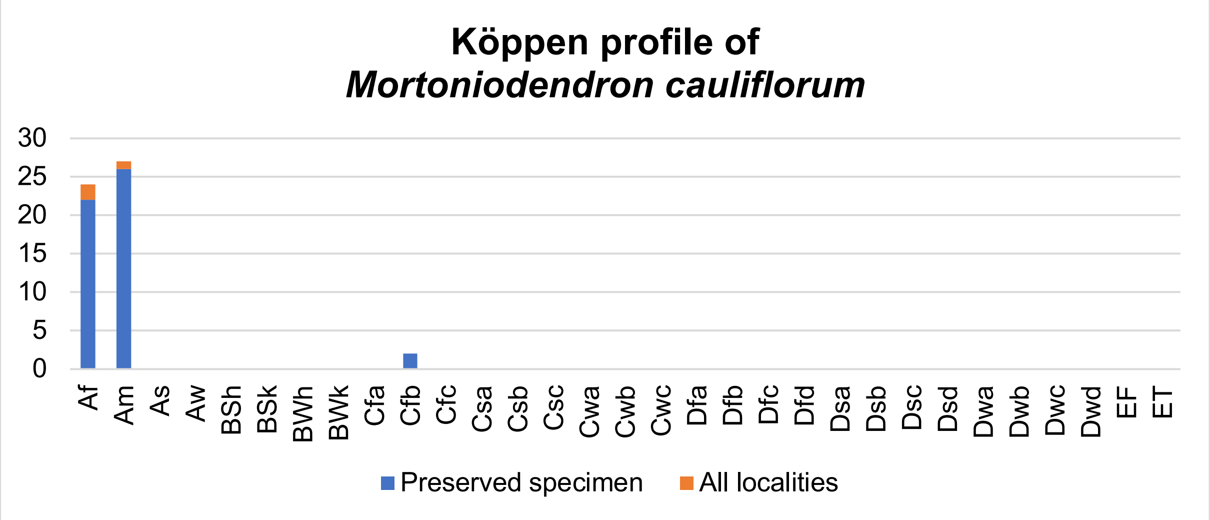

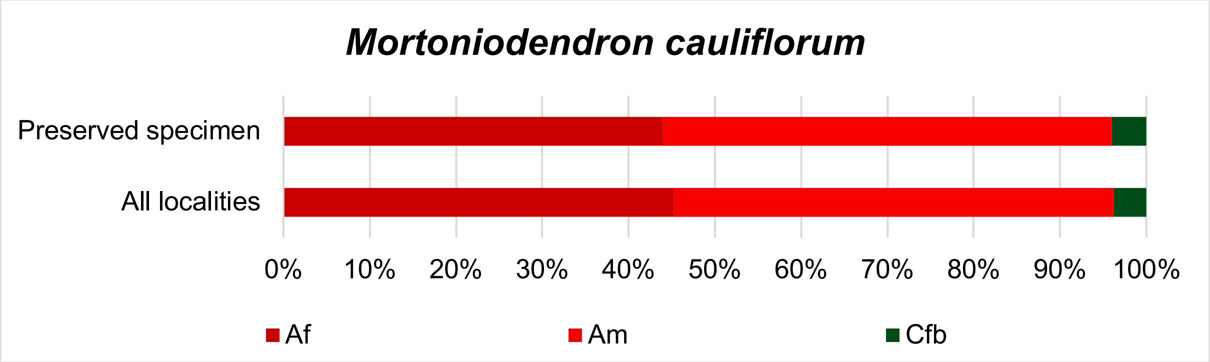

4.4.2. Biome profile, distribution, and biome map – GBIF specimens of *Mortoniodendron cauliflorum*; excluding duplicate occurrences (n = 52), herbarium specimens (n = 49).

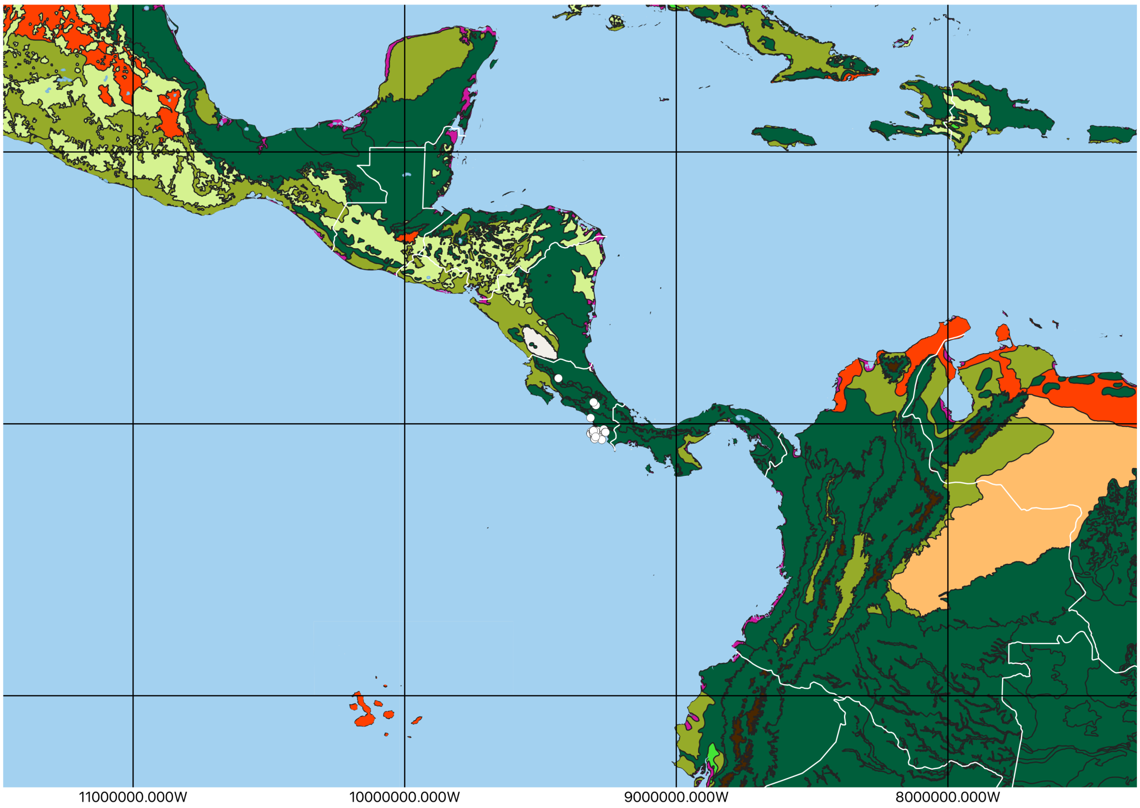

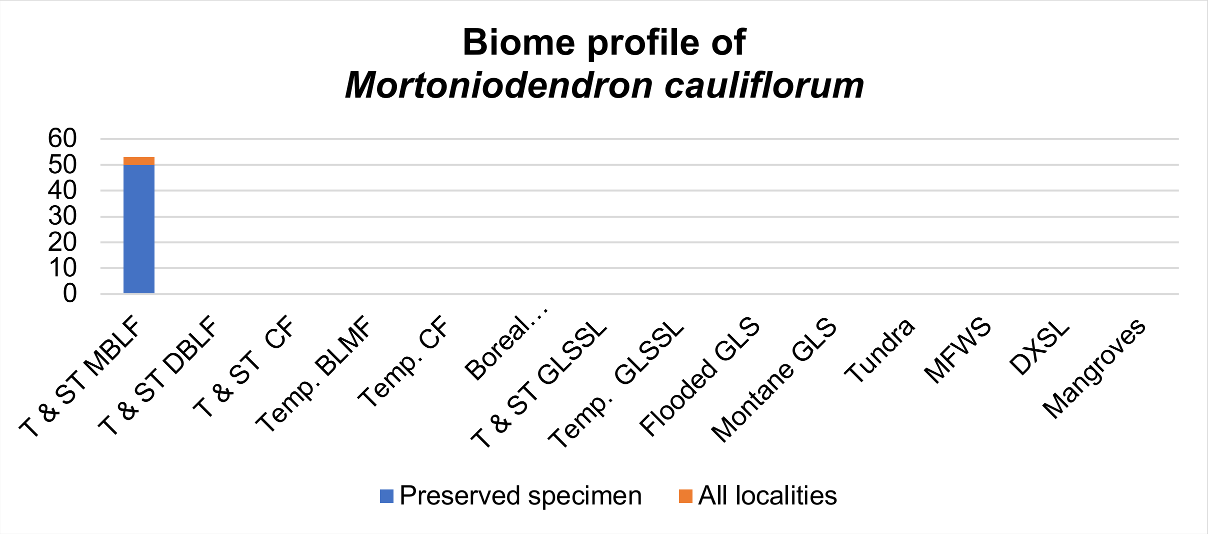

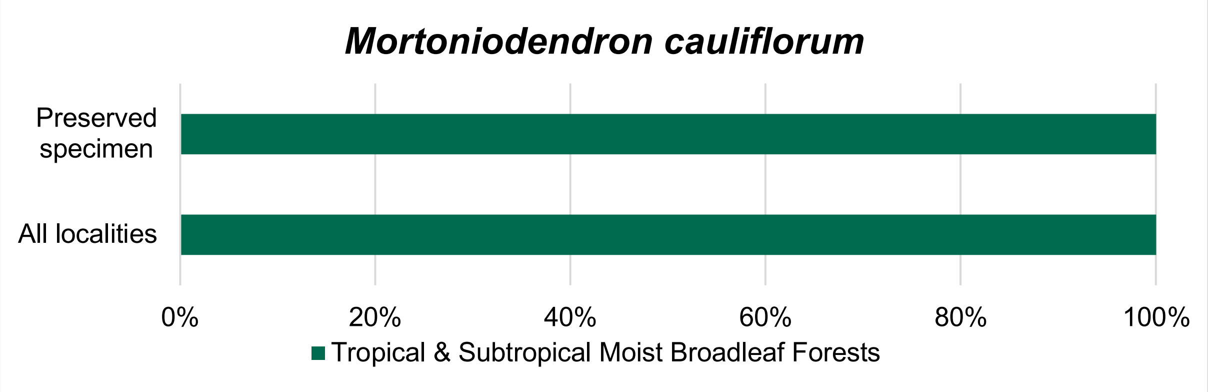

4.4.3. Climate graphs - based on 52 *Mortoniodendron cauliflorum* occurrences in GBIF

4.4.3.1. MMT [°C]

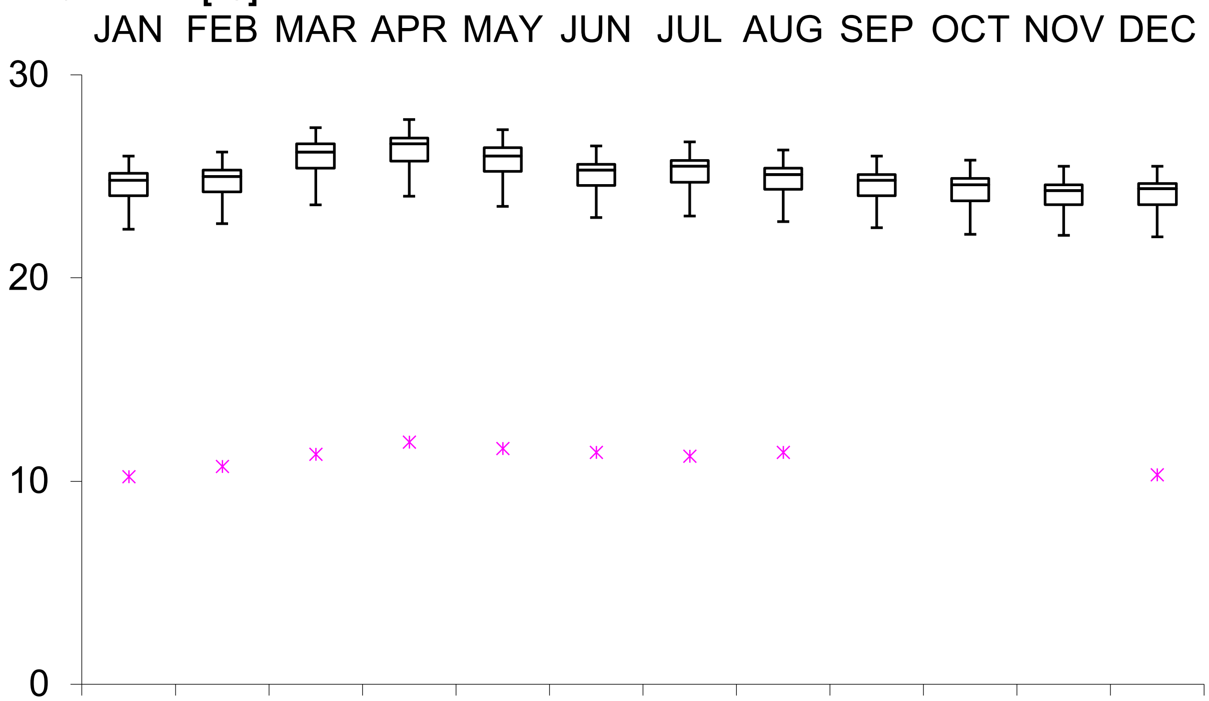

4.4.3.2. MinMT [°C]

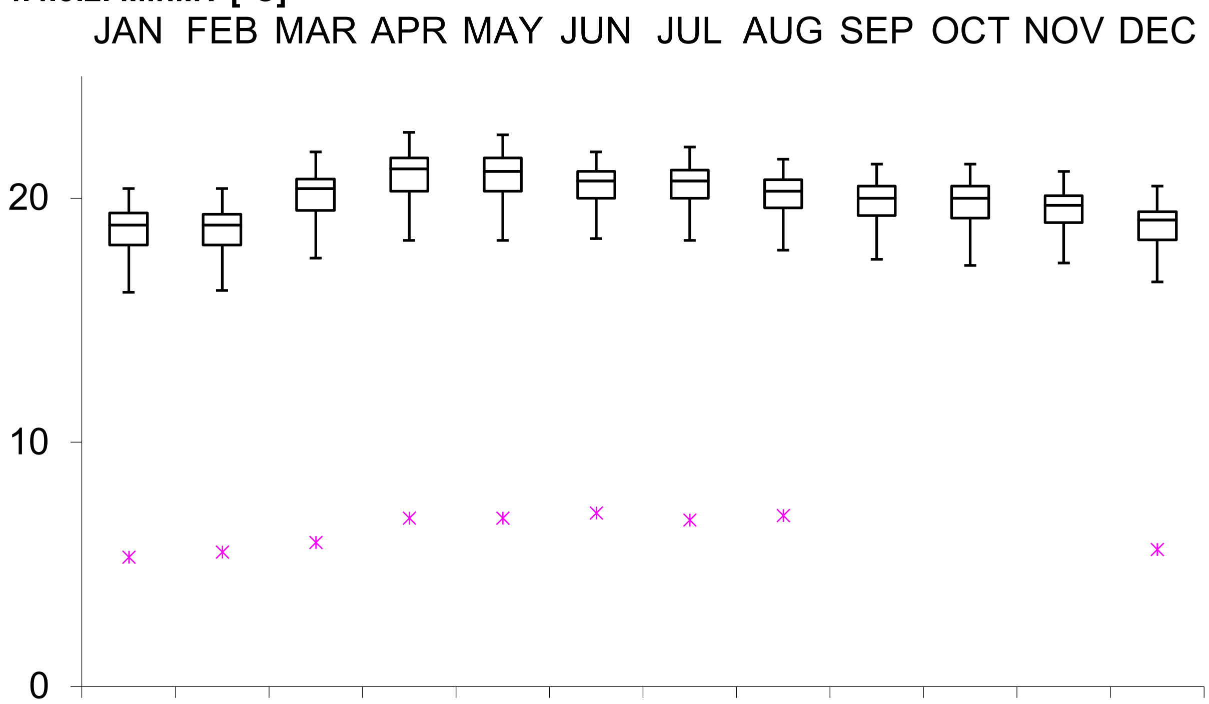

4.4.3.3. MMP [mm]

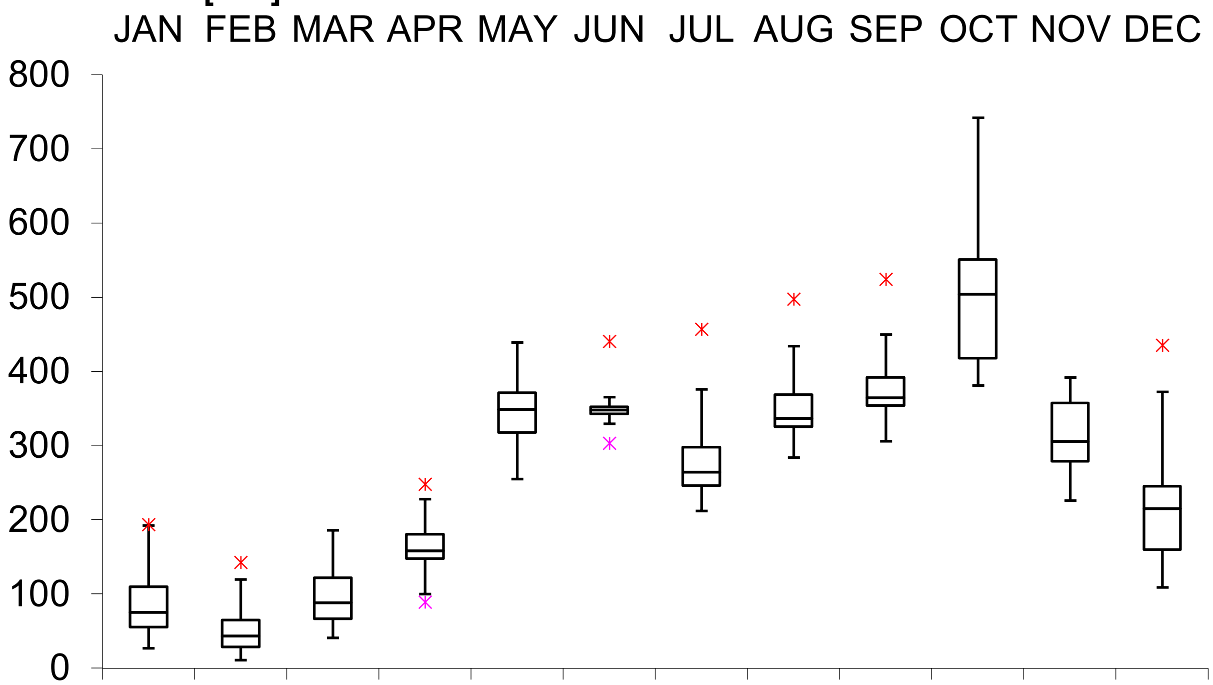
JAN FEB MAR APR MAY JUN JUL AUG SEP OCT NOV DEC
800
700
600
500
400
300
200
100
0

4.5. Species *Mortoniodendron costaricense* Standl. et L.O.Williams, 1951

4.5.1. Köppen profile, distribution, and climate map – GBIF specimens of *Mortoniodendron costaricense*; excluding duplicate occurrences (n = 121), herbarium specimens (n = 119).

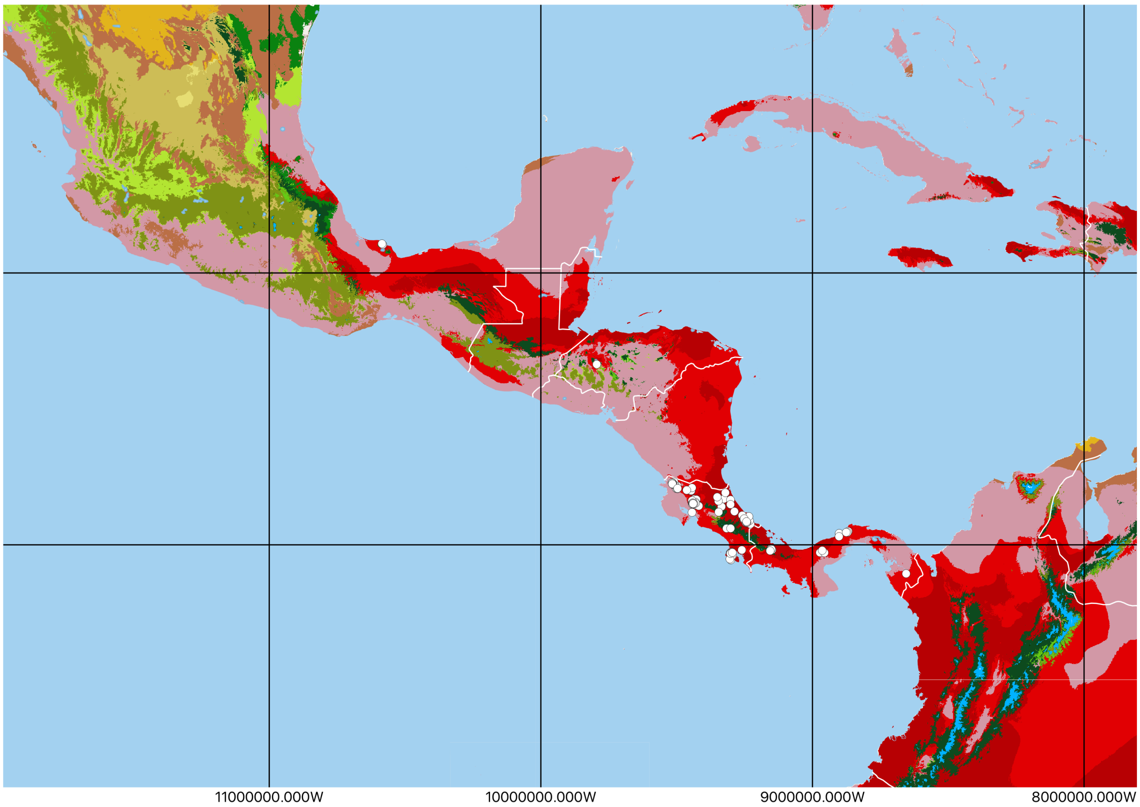

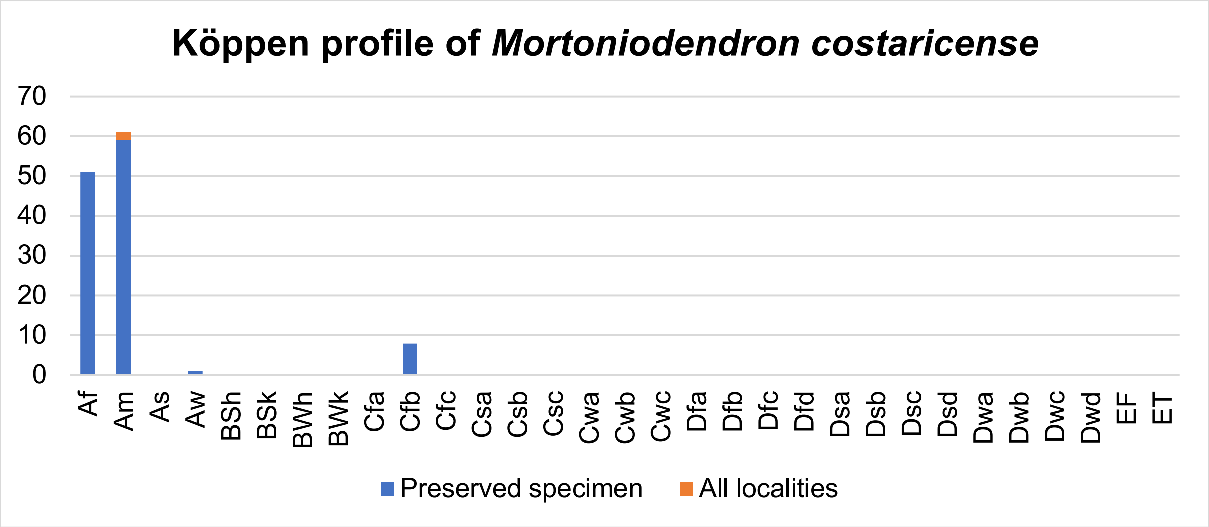

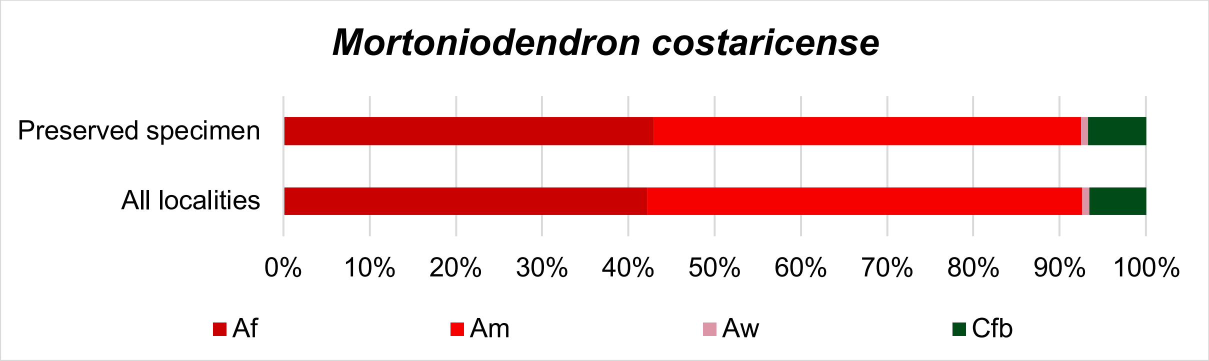

4.5.2. Biome profile, distribution, and biome map – GBIF specimens of *Mortoniodendron costaricense*; excluding duplicate occurrences (n = 121), herbarium specimens (n = 119).

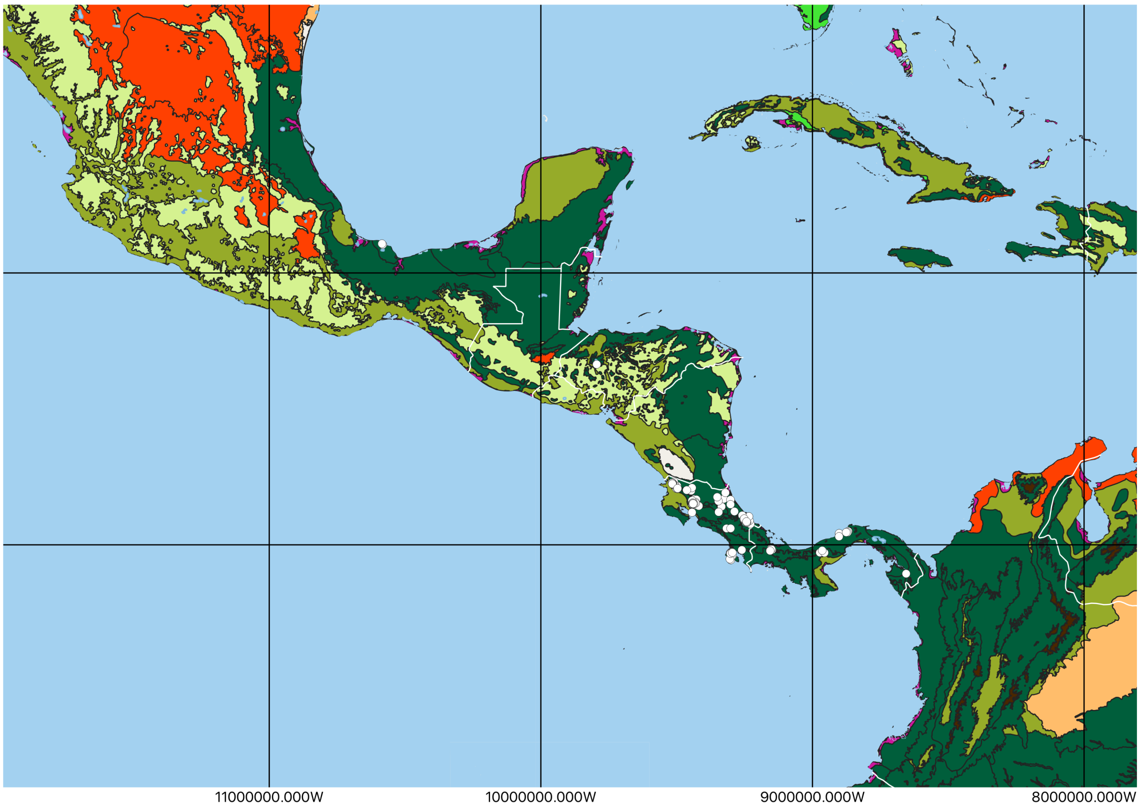

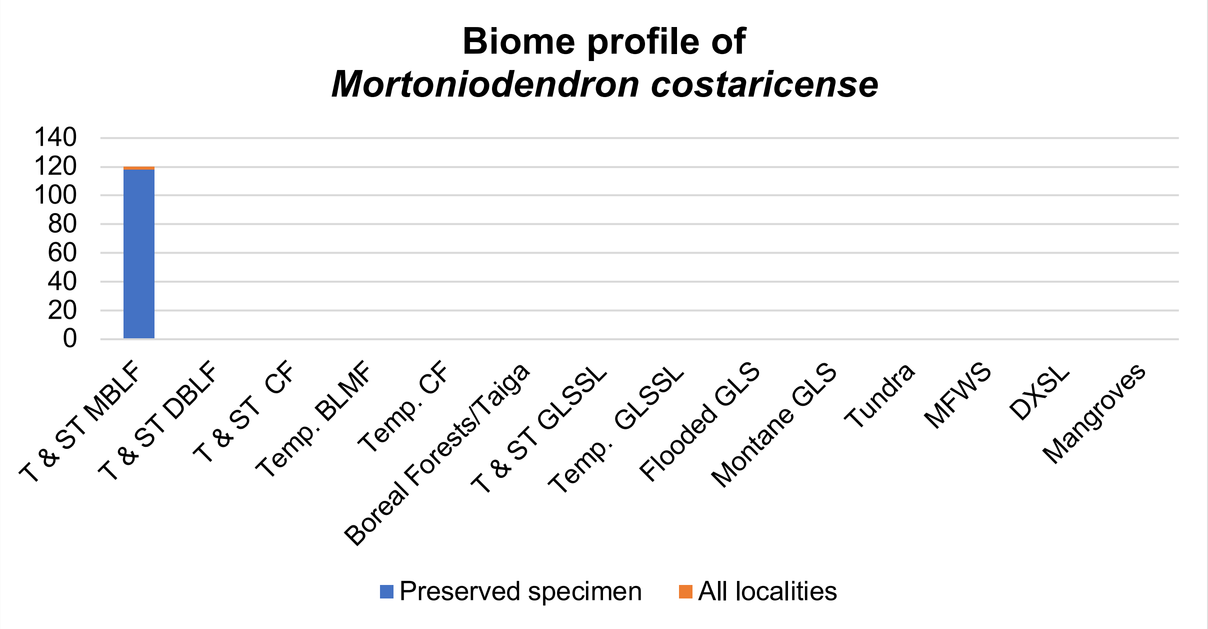

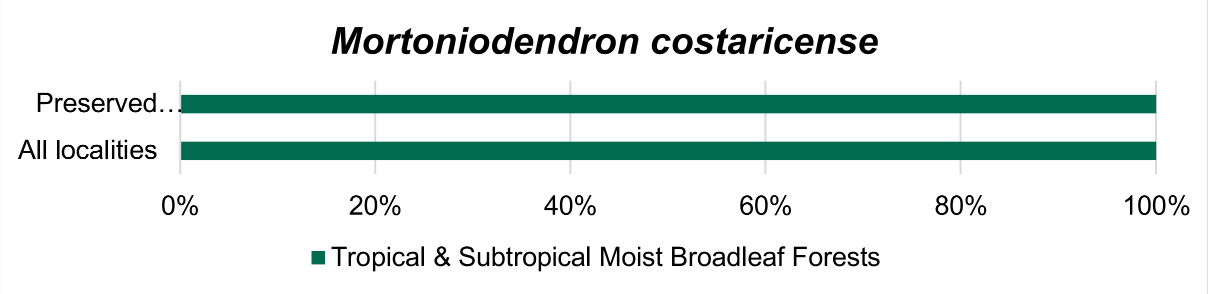

4.5.3. Climate graphs - based on 121 *Mortoniodendron costaricense* occurrences in GBIF

4.5.3.1. MMT [°C]

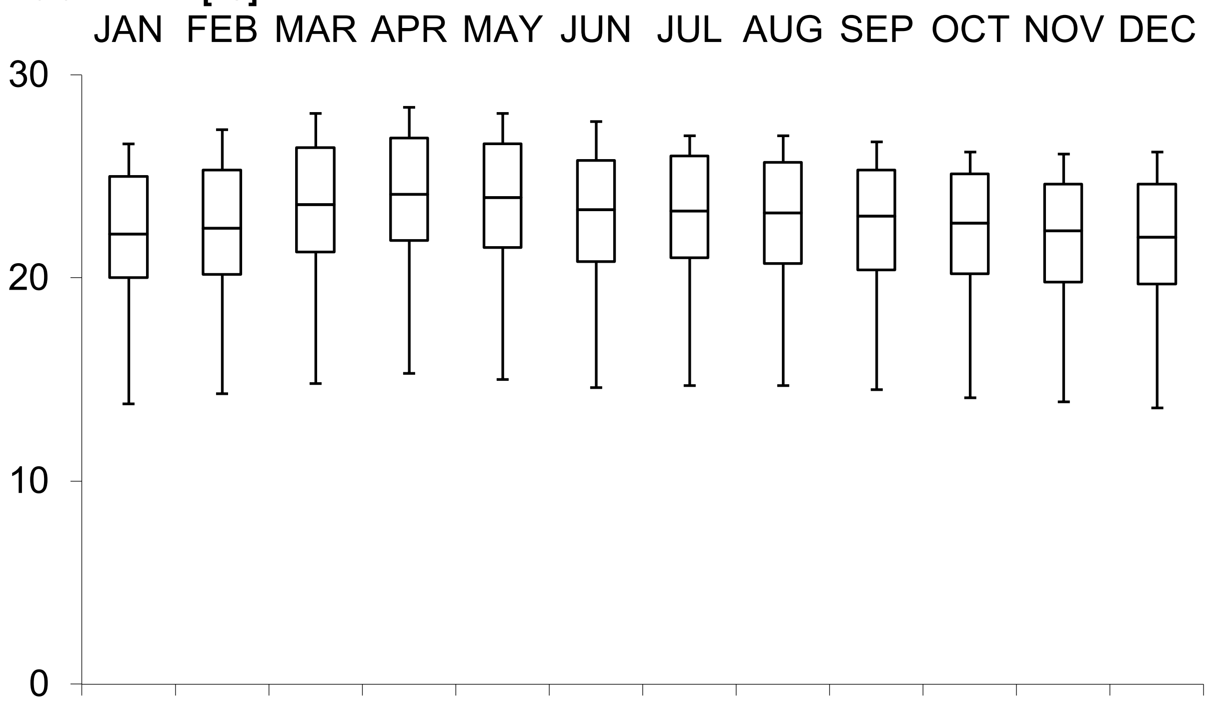

4.5.3.2. Minimum monthly temperature (MinMT) [°C]

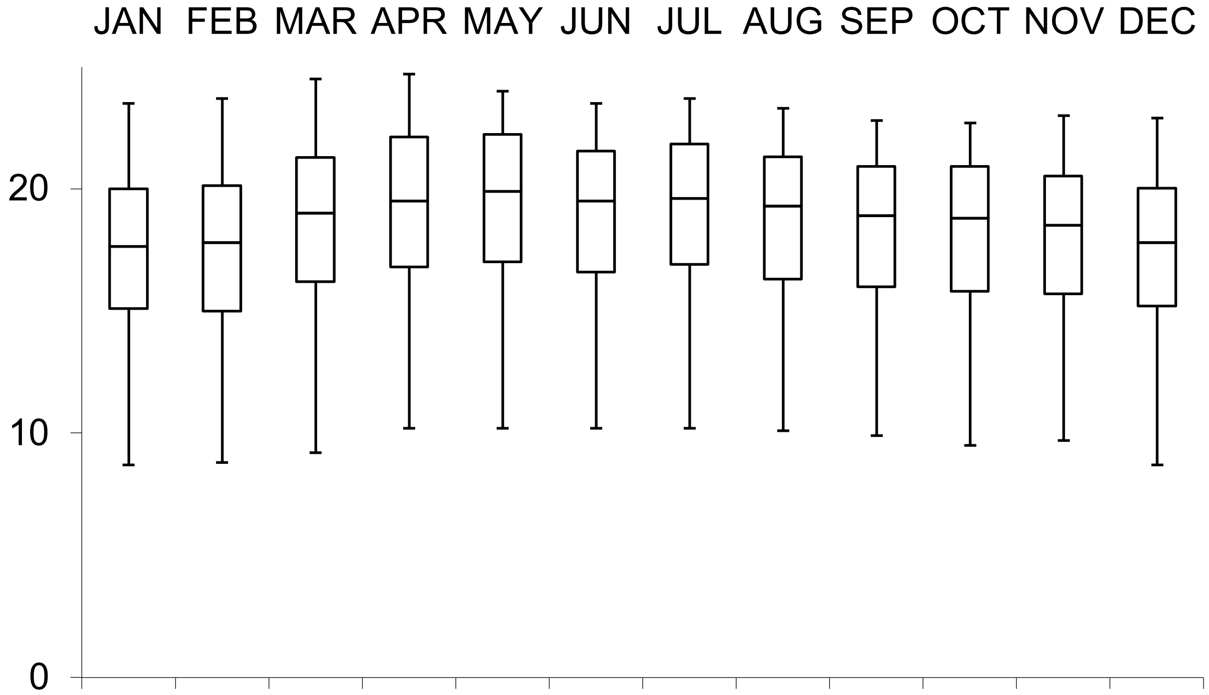

4.5.3.3. Mean monthly precipitation (MMP) [mm]

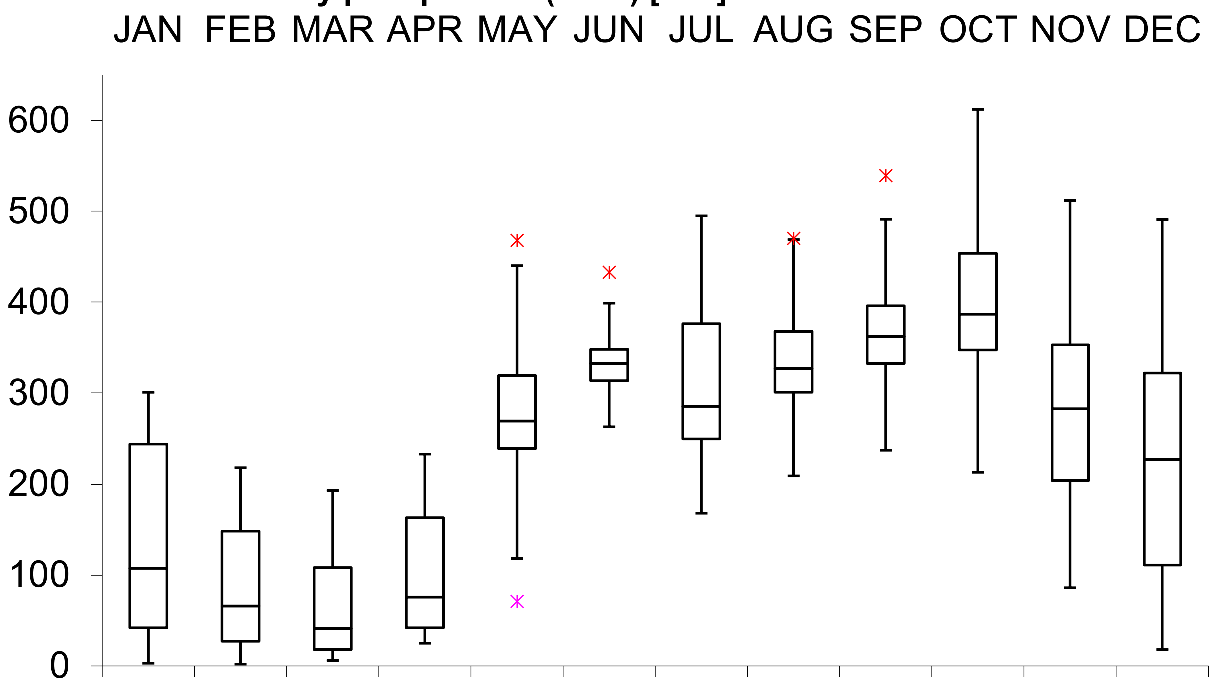

4.6. Species *Mortoniodendron guatemalense* Standl. et Steyerm., 1940

4.6.1. Köppen profile, distribution, and climate map – GBIF occurrences of *Mortoniodendron guatemalense*; excluding duplicate occurrences (n = 171), herbarium specimens (n = 169).

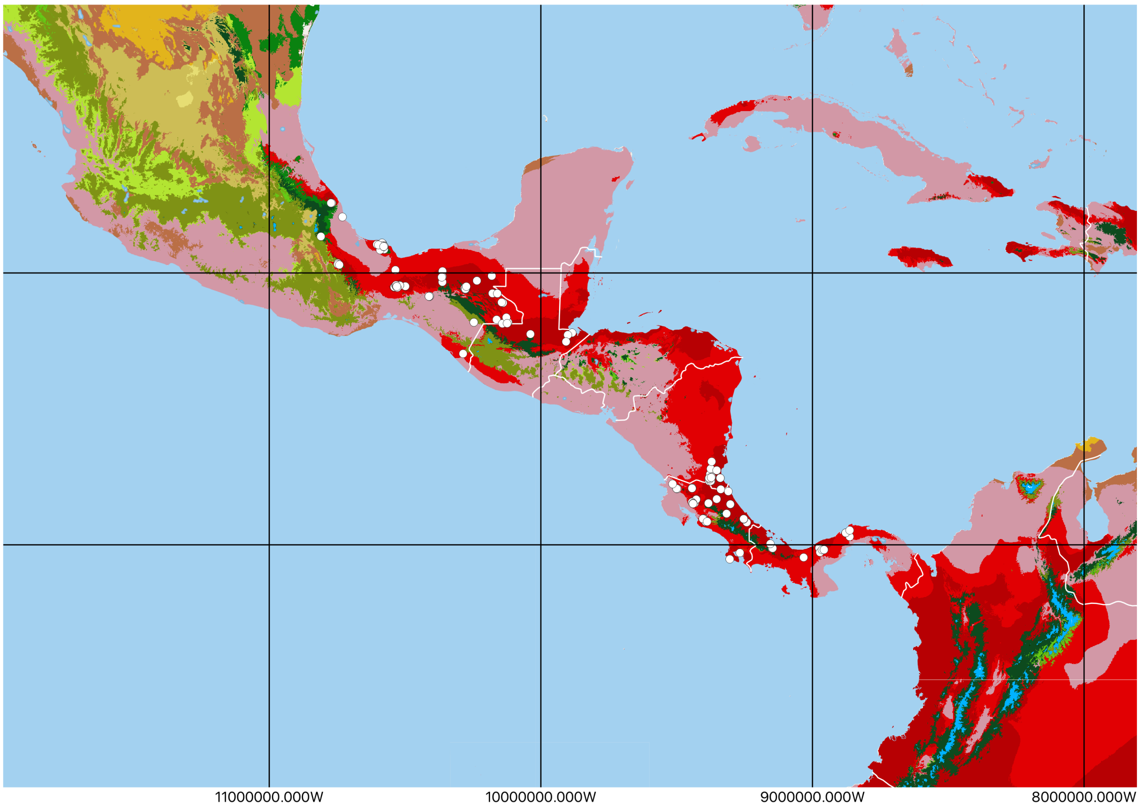

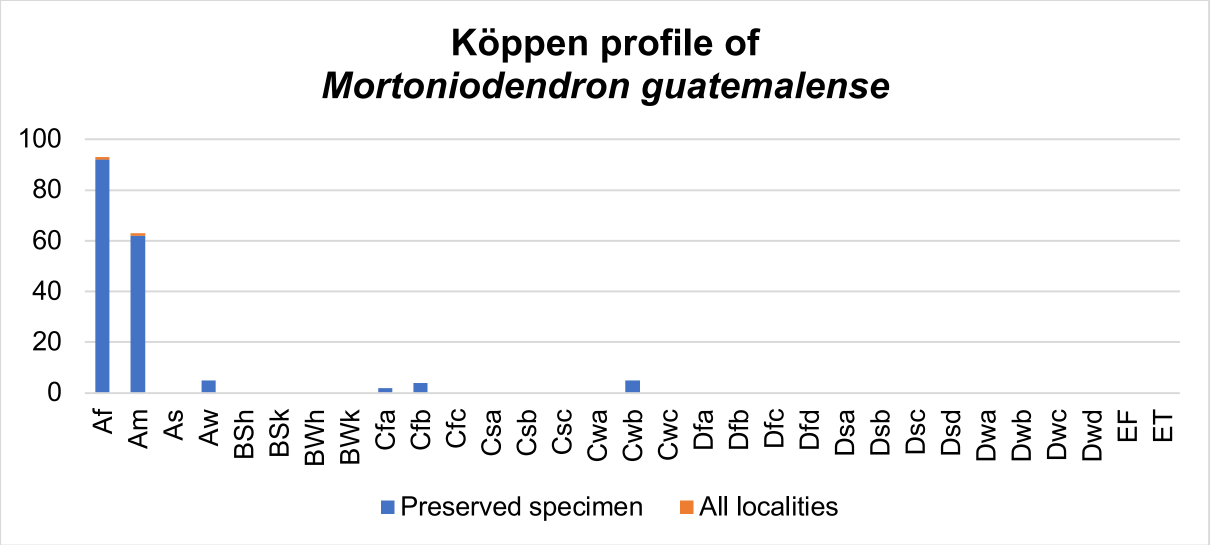

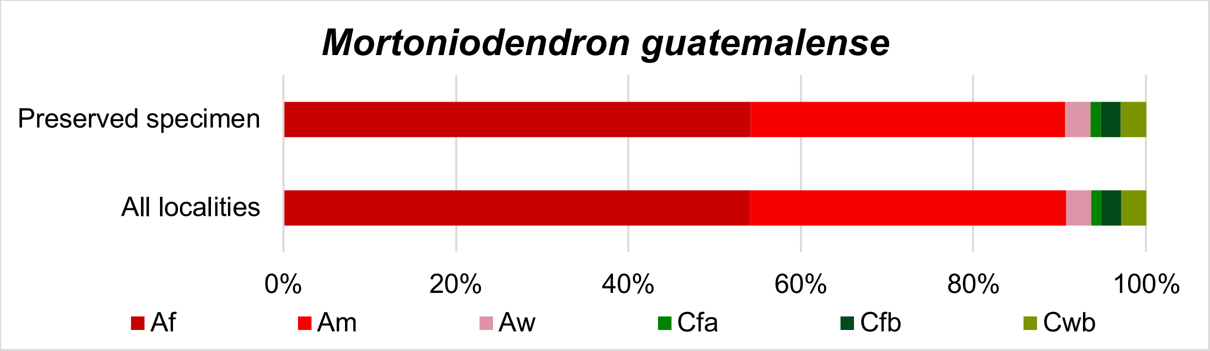

4.6.2. Biome profile, distribution, and biome map – GBIF occurrences of *Mortoniodendron guatemalense*; excluding duplicate occurrences (n = 171), herbarium specimens (n = 169).

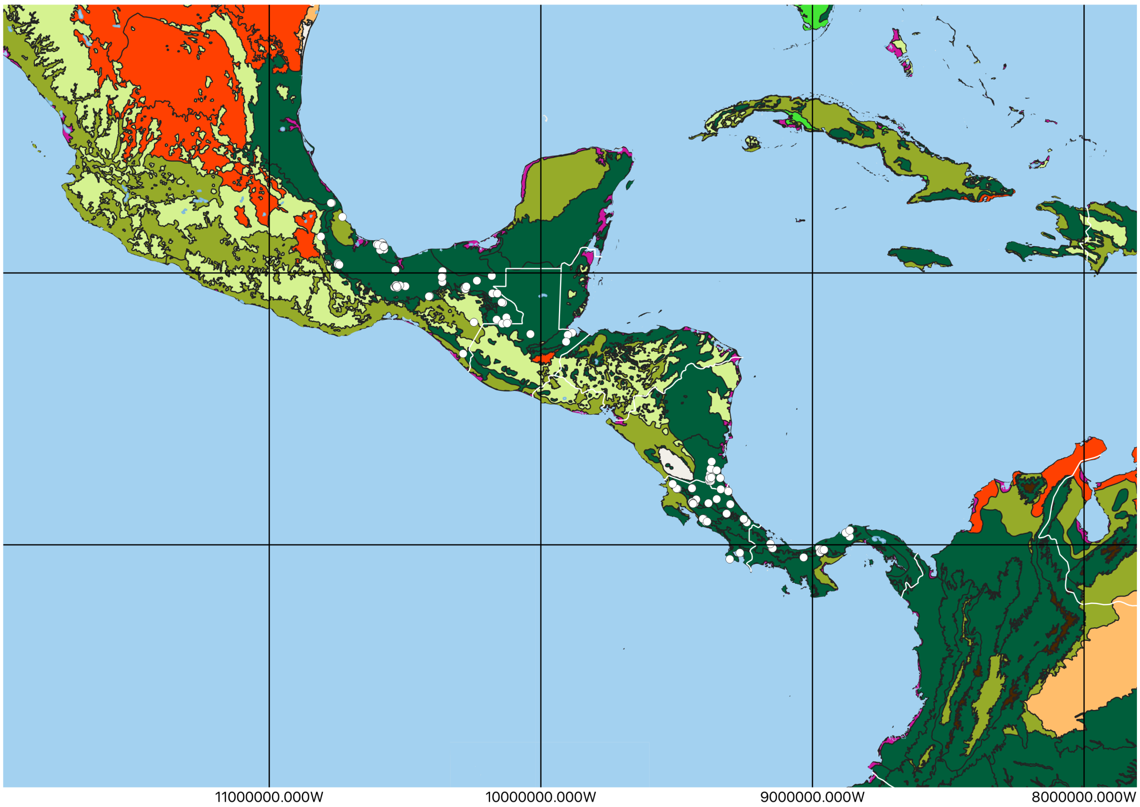

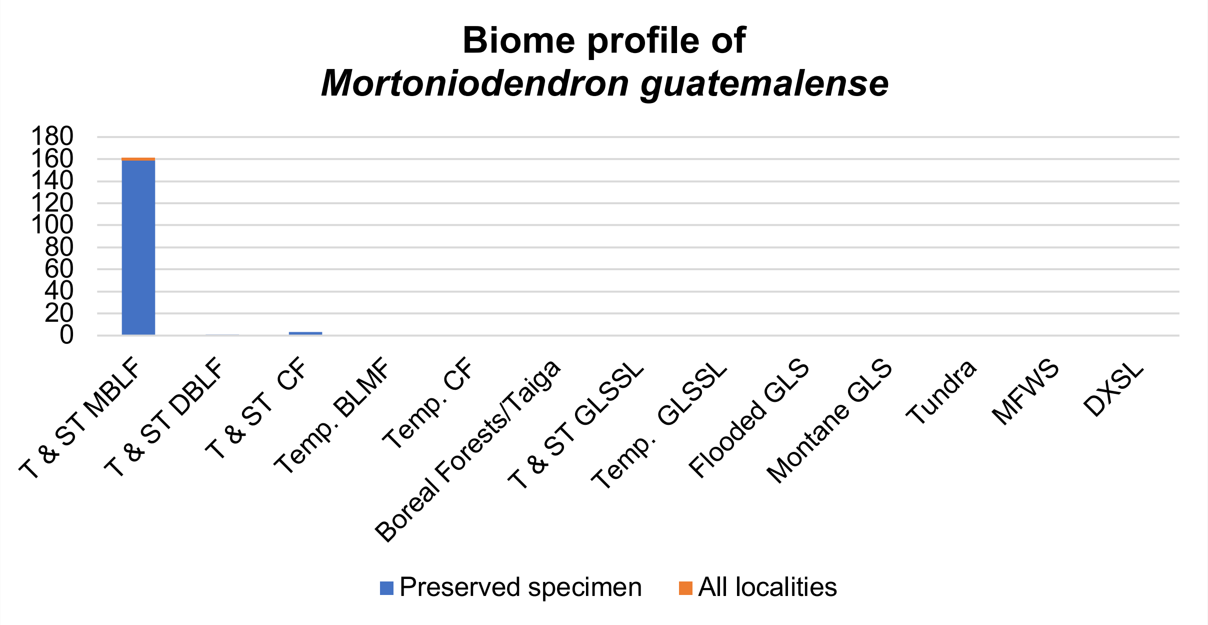

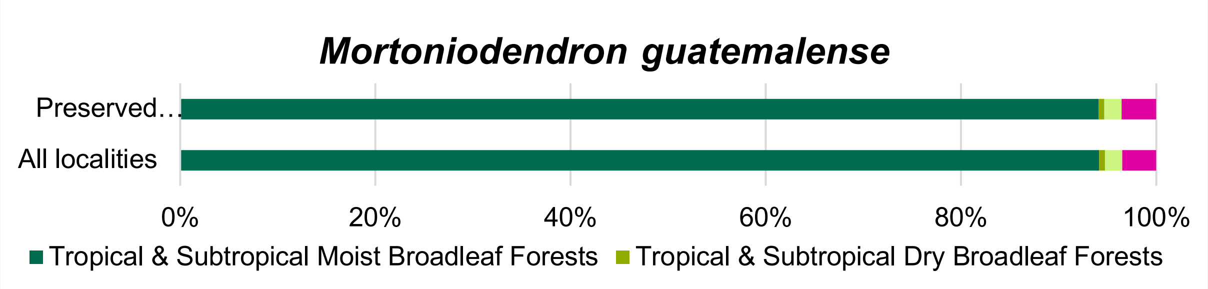

4.6.3. Climate graphs - based on 171 *Mortoniodendron guatemalense* occurrences in GBIF

4.6.3.1. MMT [°C]

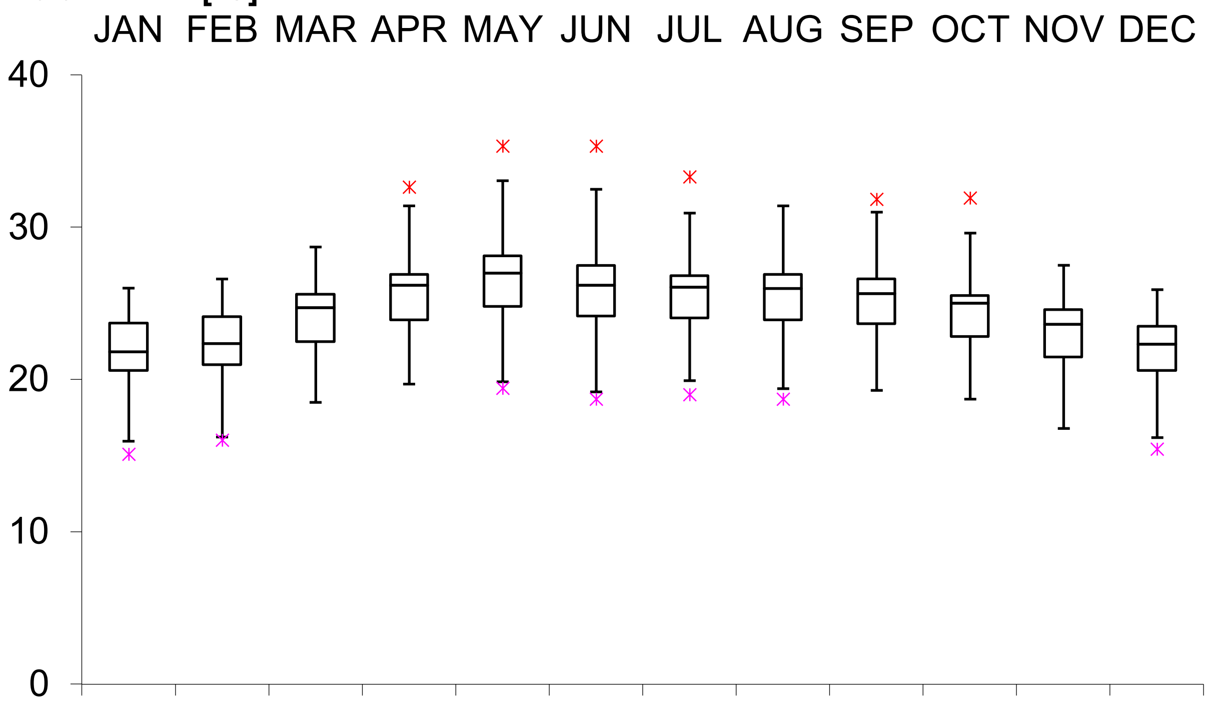

4.6.3.2. MinMT [°C]

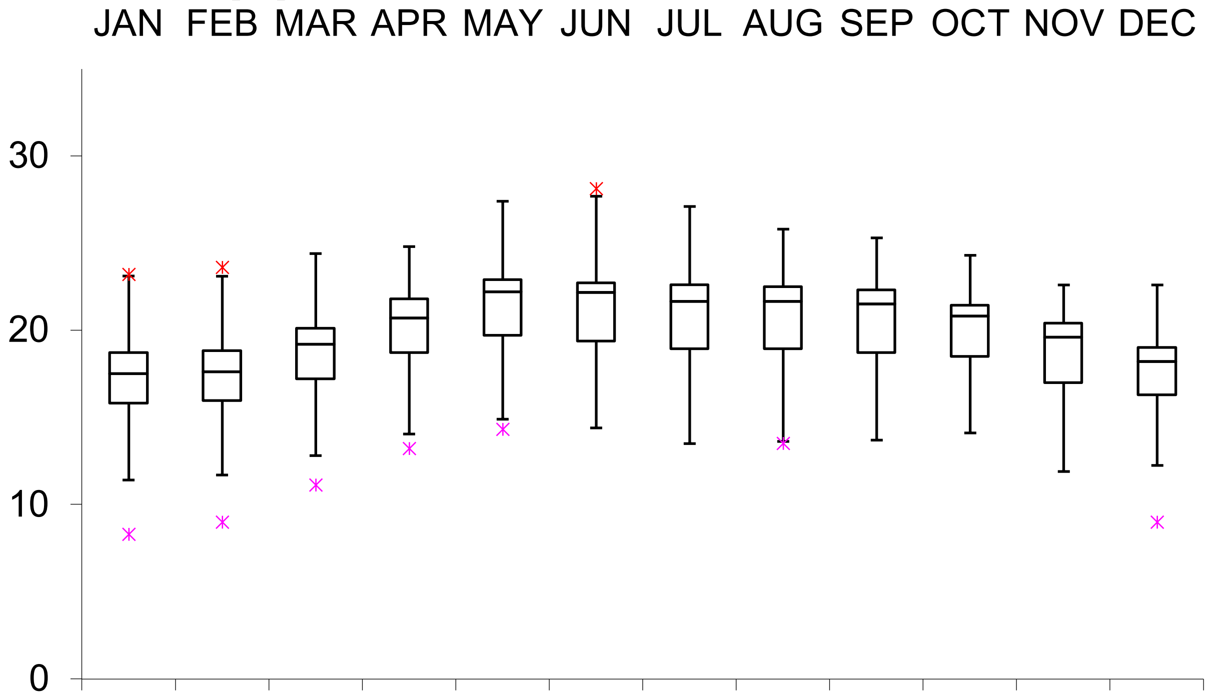

4.6.3.3. MMP [mm]

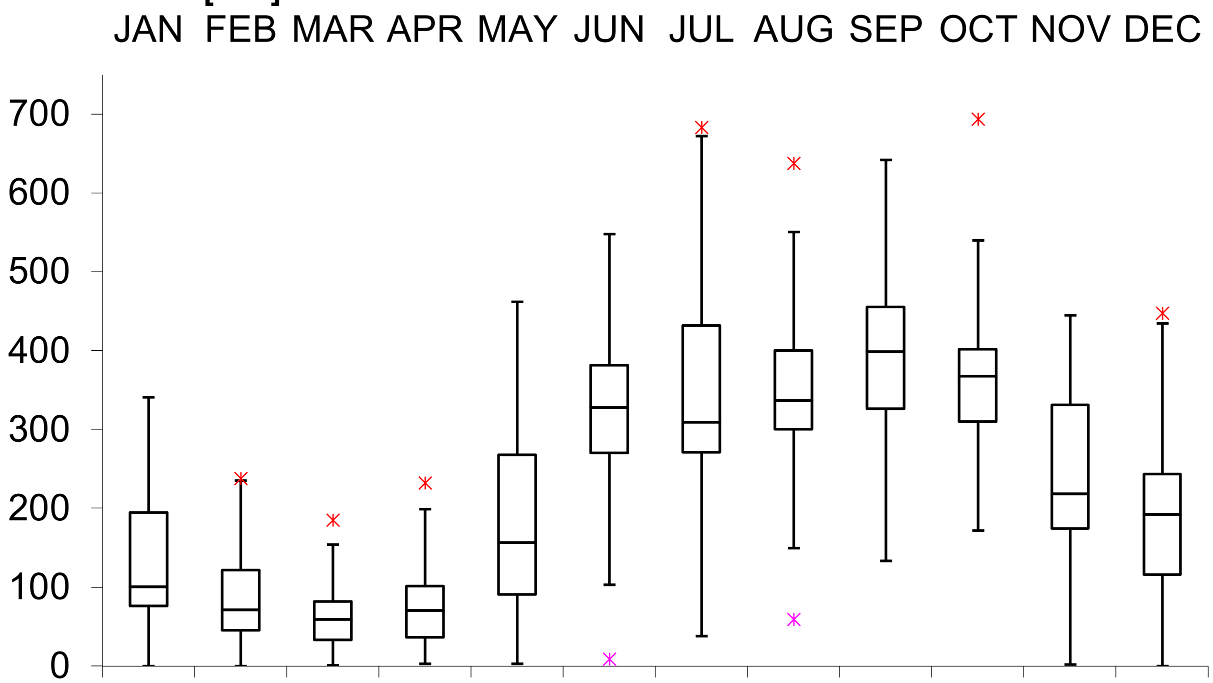

JAN FEB MAR APR MAY JUN JUL AUG SEP OCT NOV DEC
700
600
500
400
300
200
100
0

4.7. Species *Mortoniodendron hirsutum* Standl., 1942

4.7.1. Köppen profile, distribution, and climate map – GBIF occurrences of *Mortoniodendron hirsutum*; herbarium specimens (n = 33).

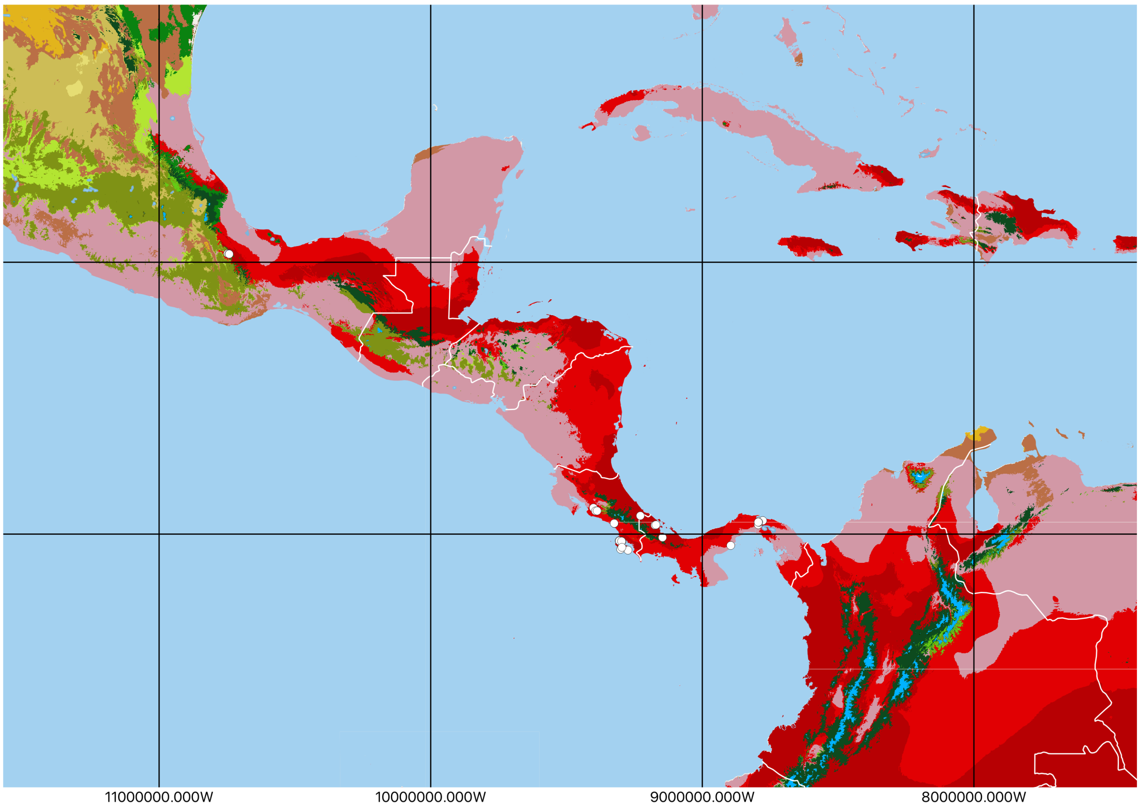

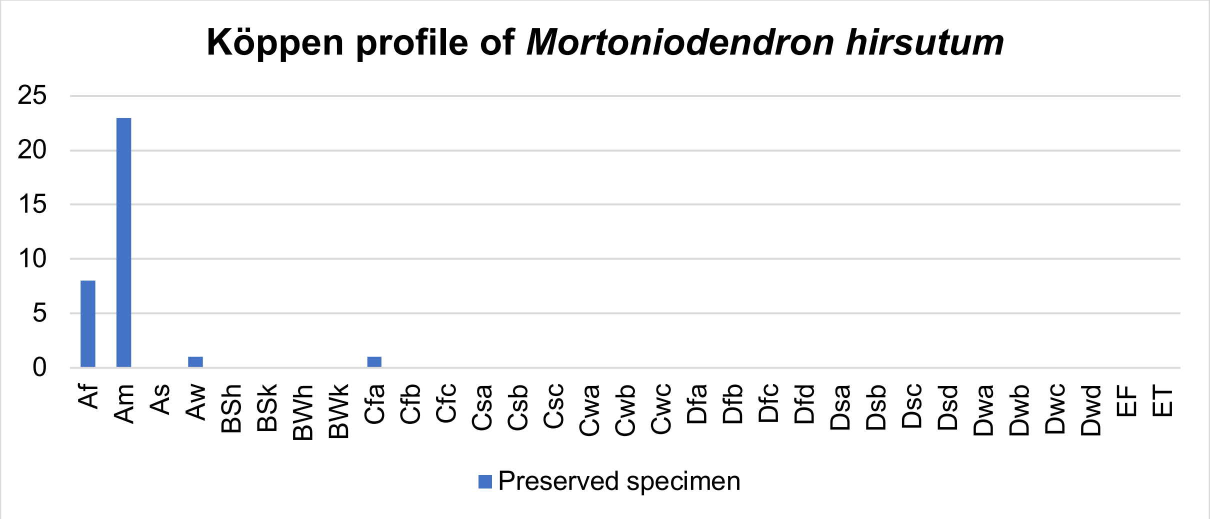

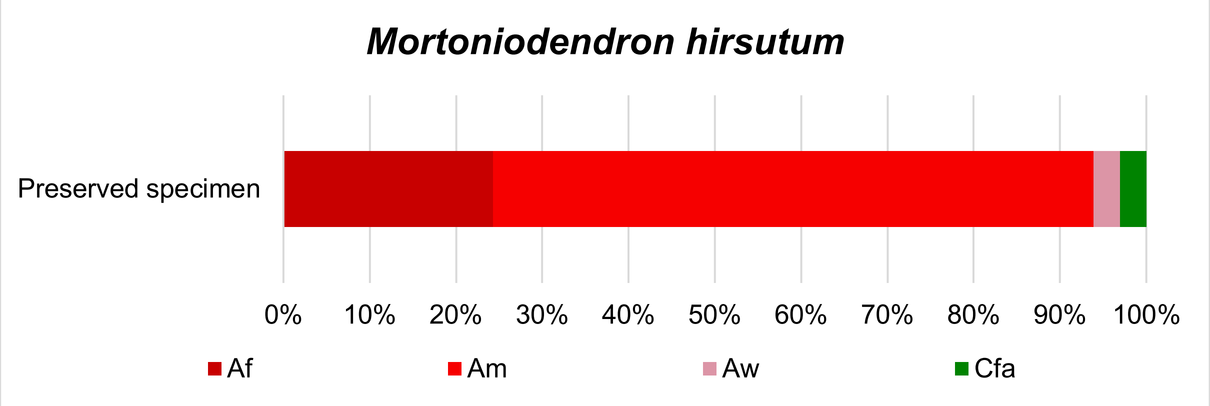

4.7.2. Biome profile, distribution, and biome map – GBIF occurrences of *Mortoniodendron hirsutum*; herbarium specimens (n = 33).

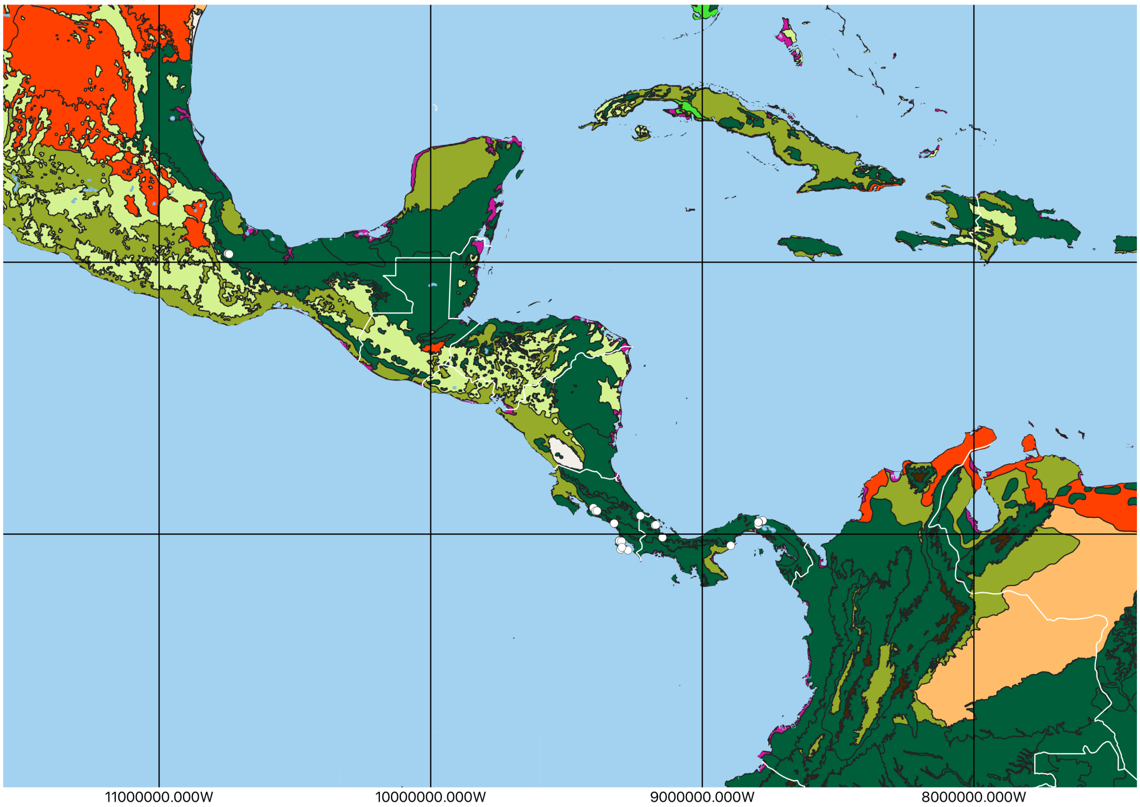

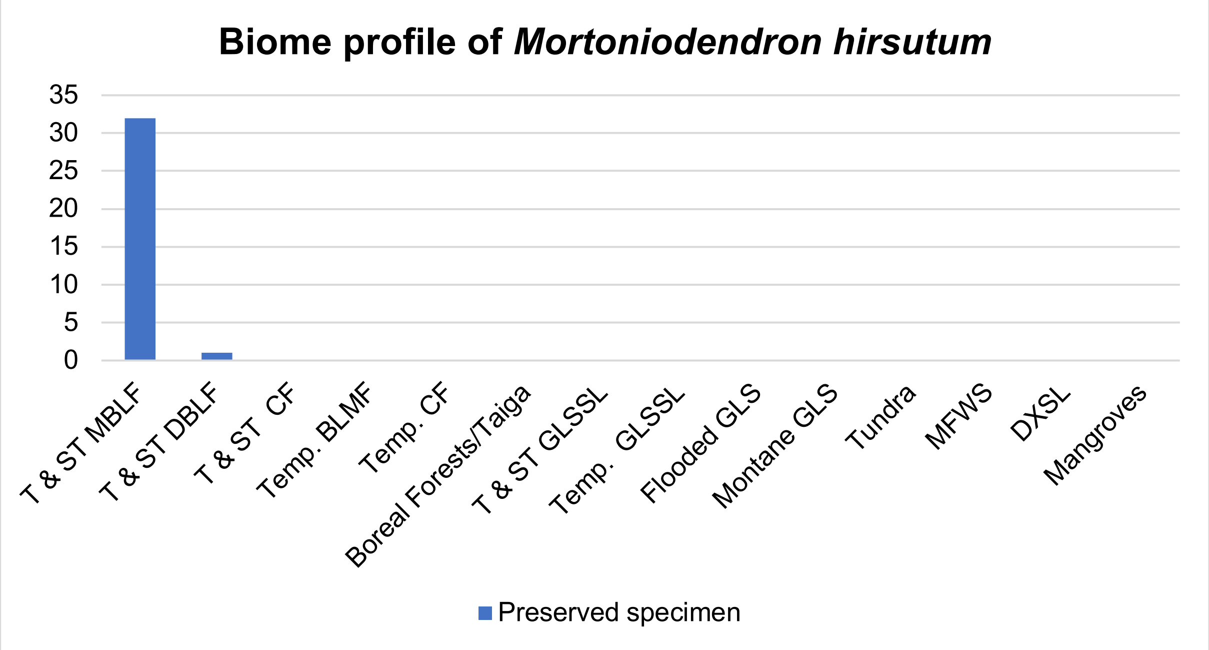

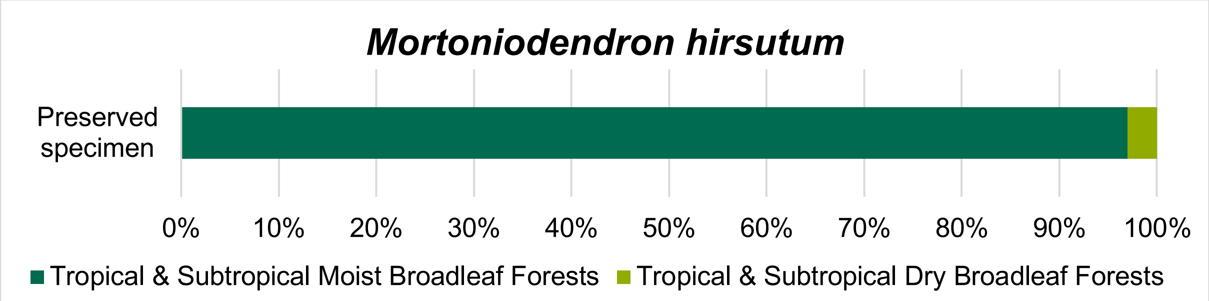

4.7.3. Climate graphs - based on 33 *Mortoniodendron hirsutum* occurrences in GBIF

4.7.3.1. MMT [°C]

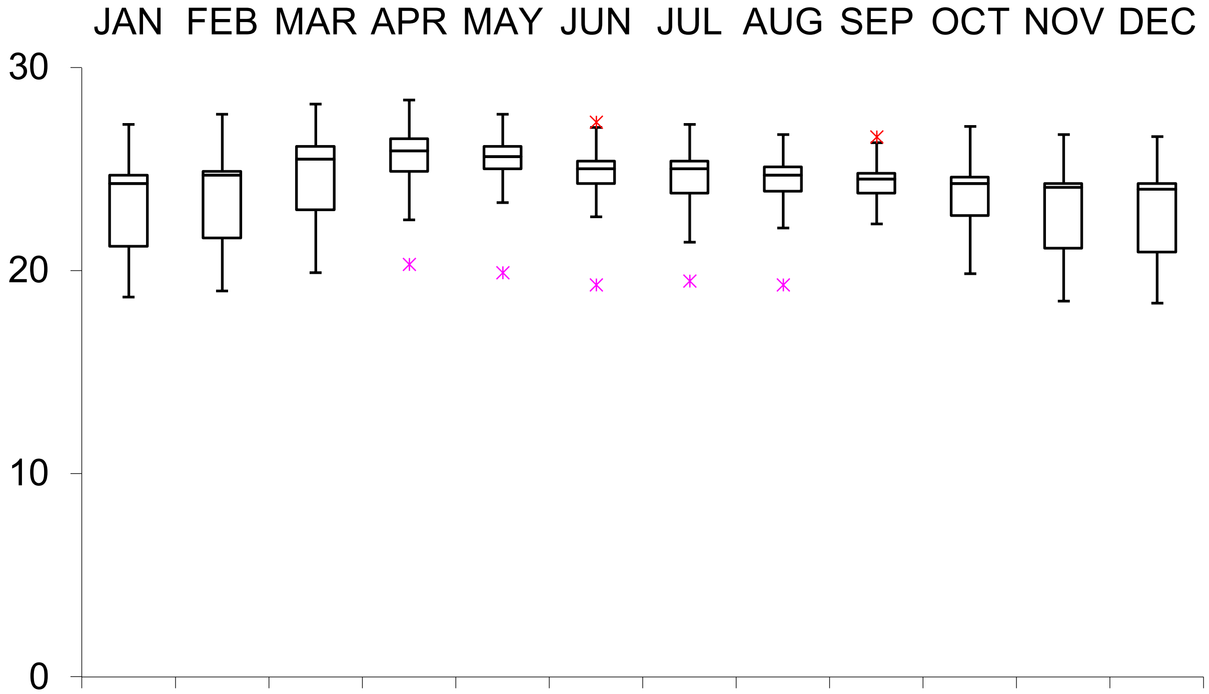

4.7.3.2. MinMT [°C]

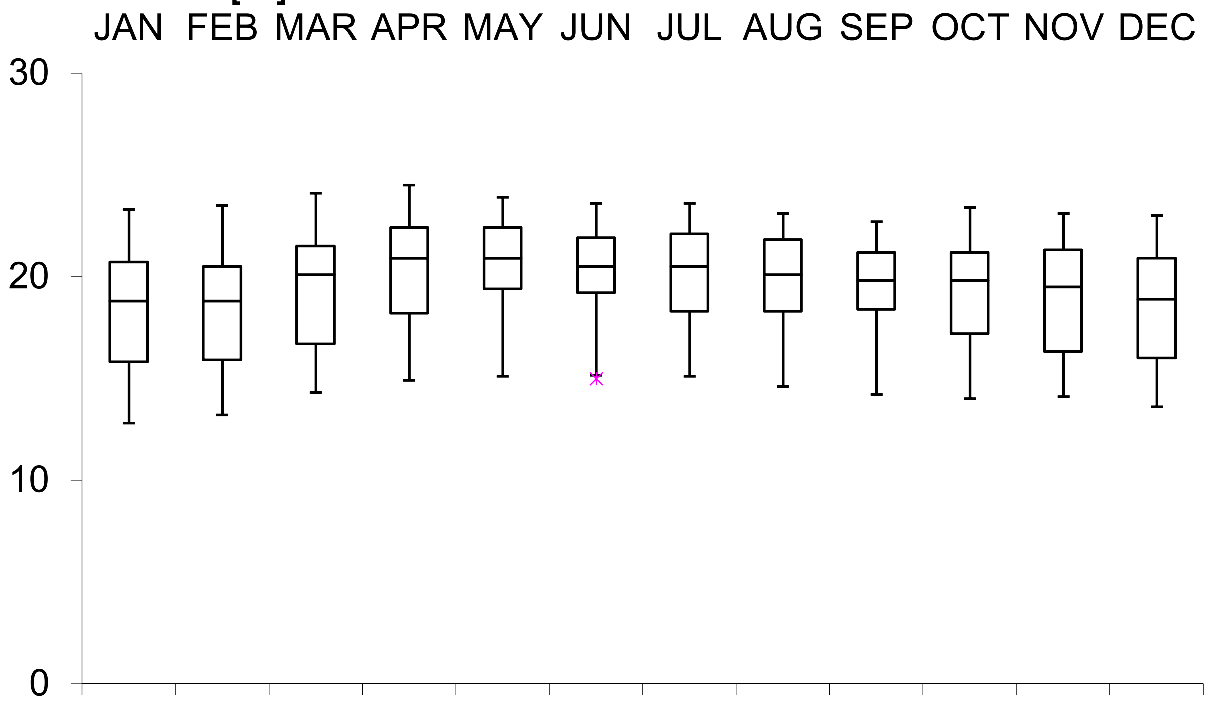

4.7.3.3. MMP [mm]

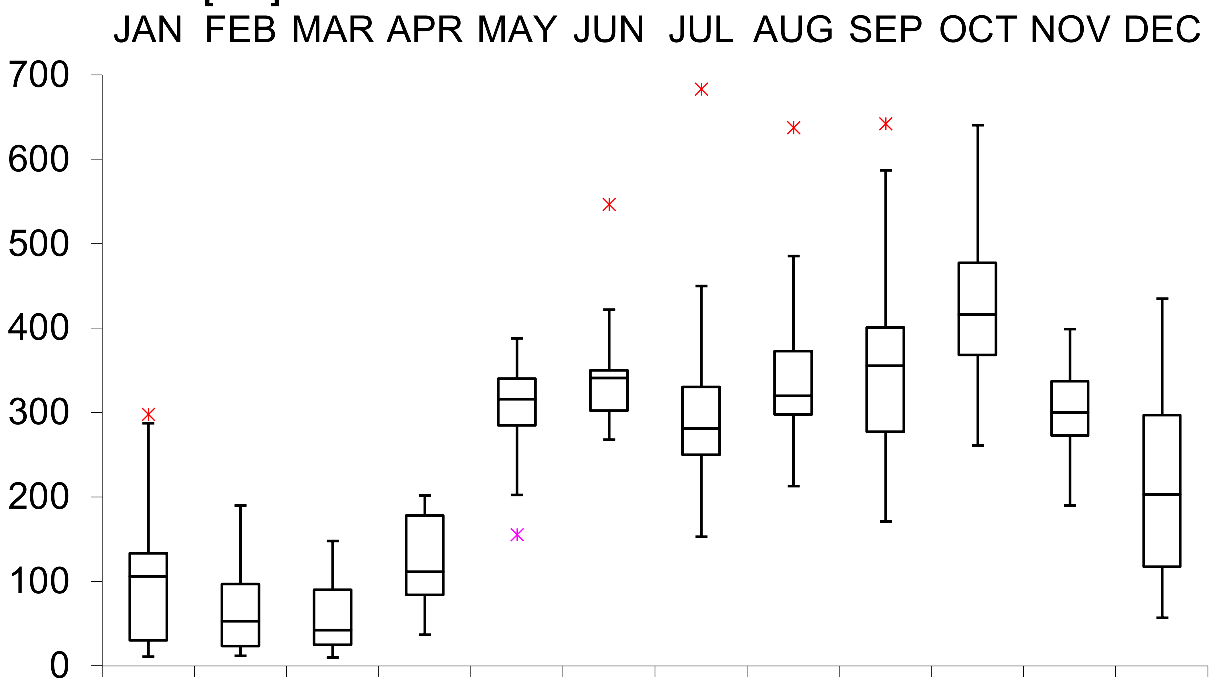
JAN FEB MAR APR MAY JUN JUL AUG SEP OCT NOV DEC
700
600
500
400
300
200
100
0

4.8. Species *Mortoniodendron longipedunculatum* Al.Rodr., 2004

4.8.1. Köppen profile, distribution, and climate map – GBIF occurrences of *Mortoniodendron longipedunculatum*; herbarium specimens (n = 4).

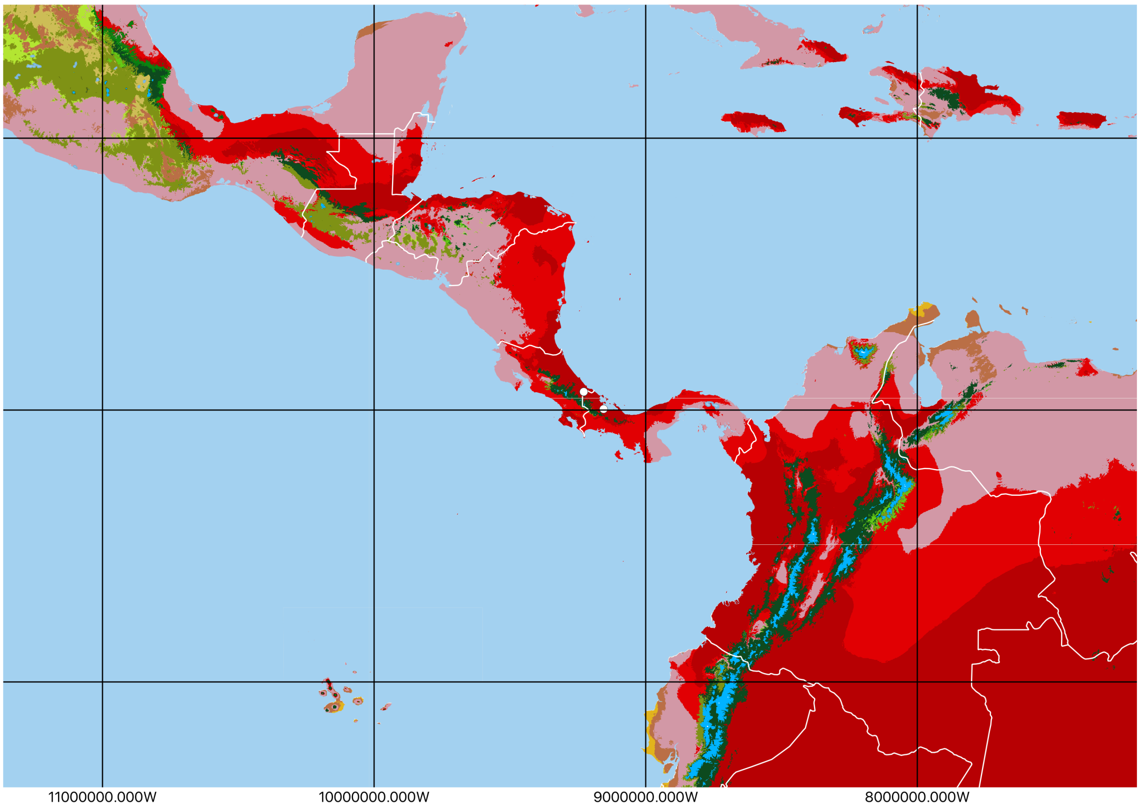

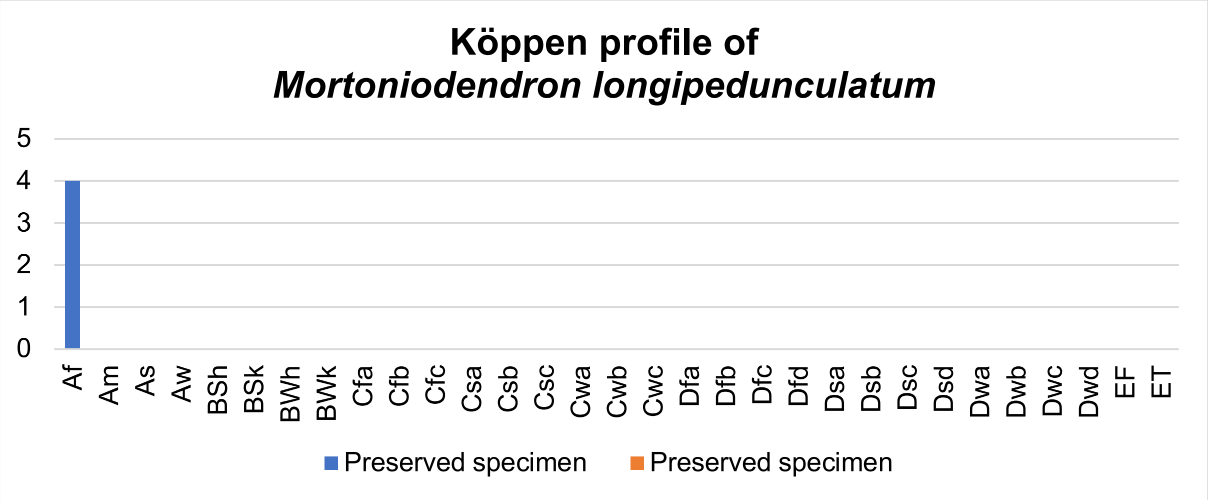

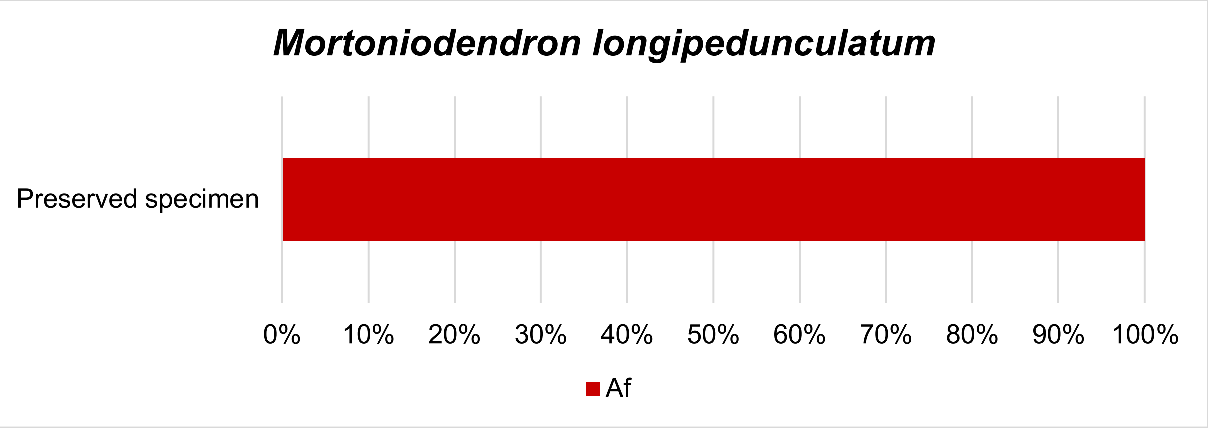

4.8.2. Biome profile, distribution, and biome map – GBIF occurrences of *Mortoniodendron longipedunculatum*; herbarium specimens (n = 4).

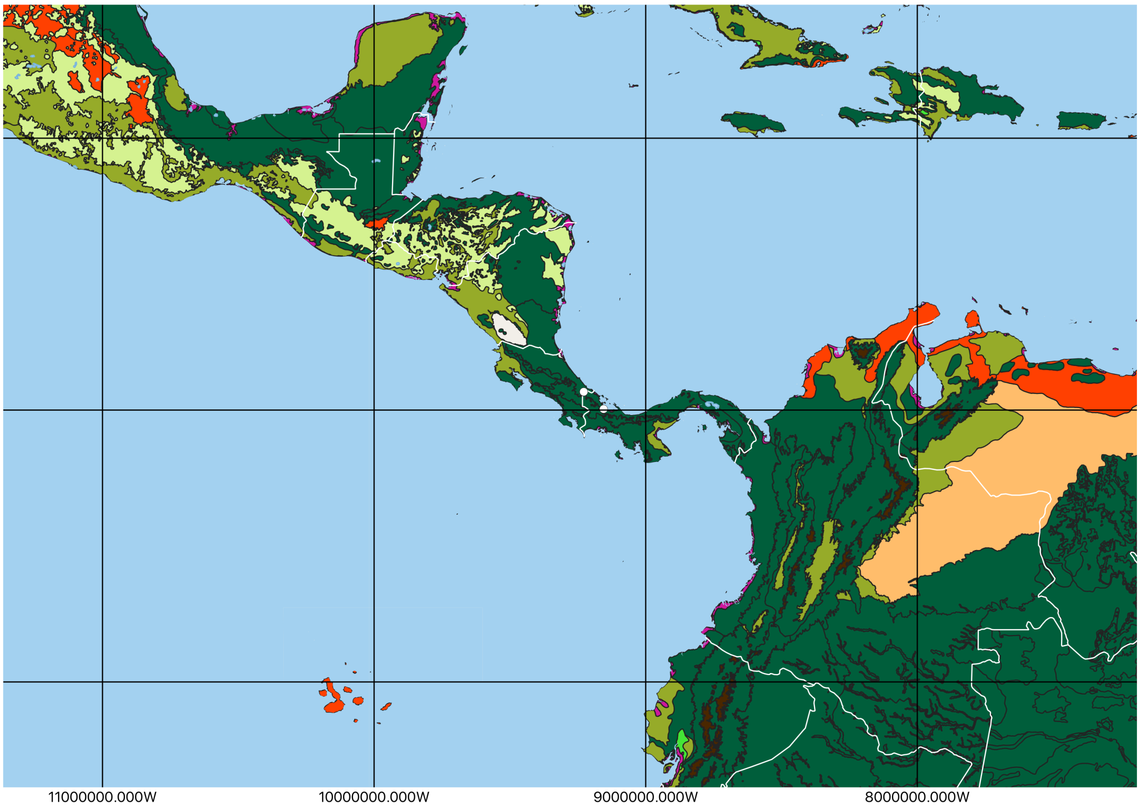

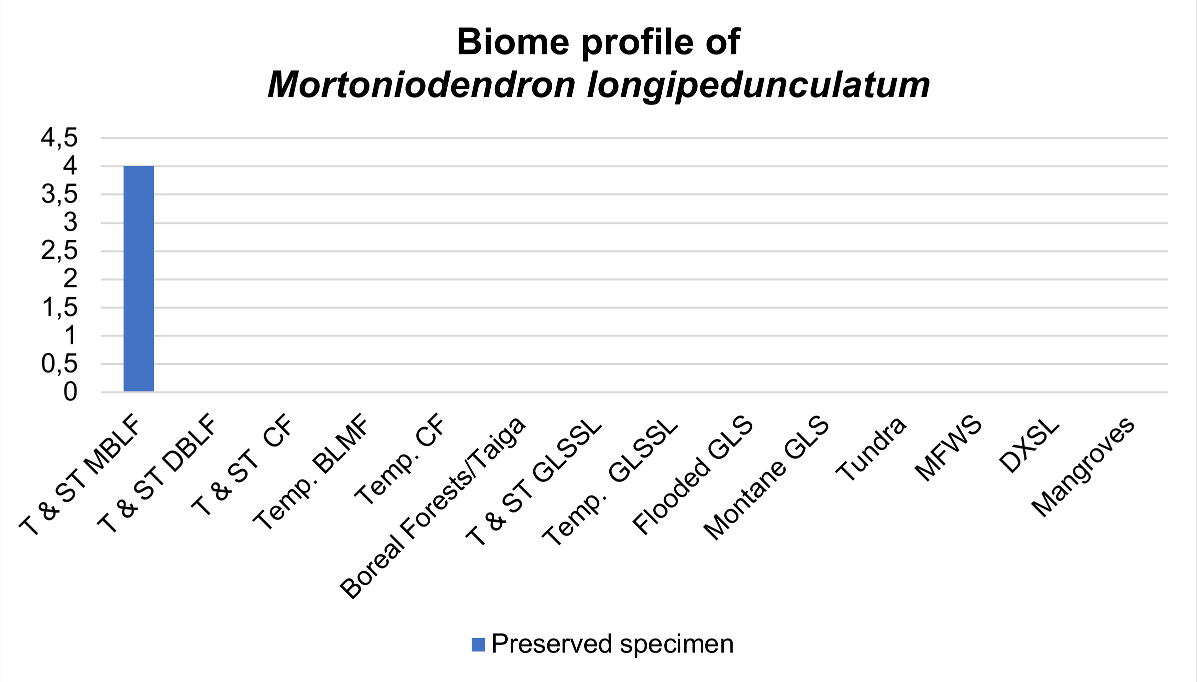

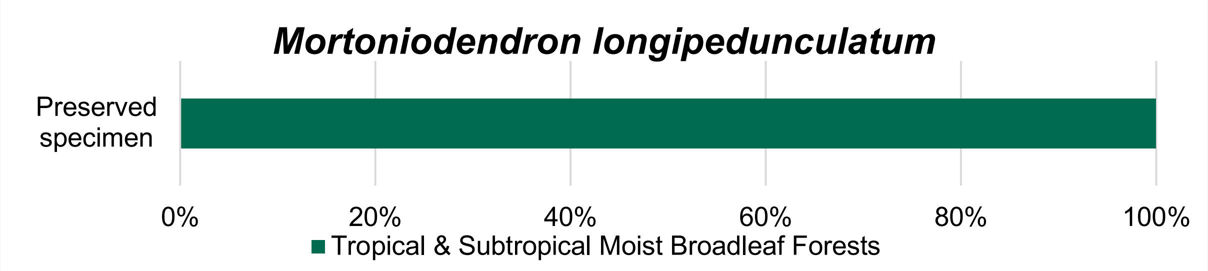

4.8.3. Climate graphs - based on 4 *Mortoniodendron longipedunculatum* occurrences in GBIF

4.8.3.1. MMT [°C]

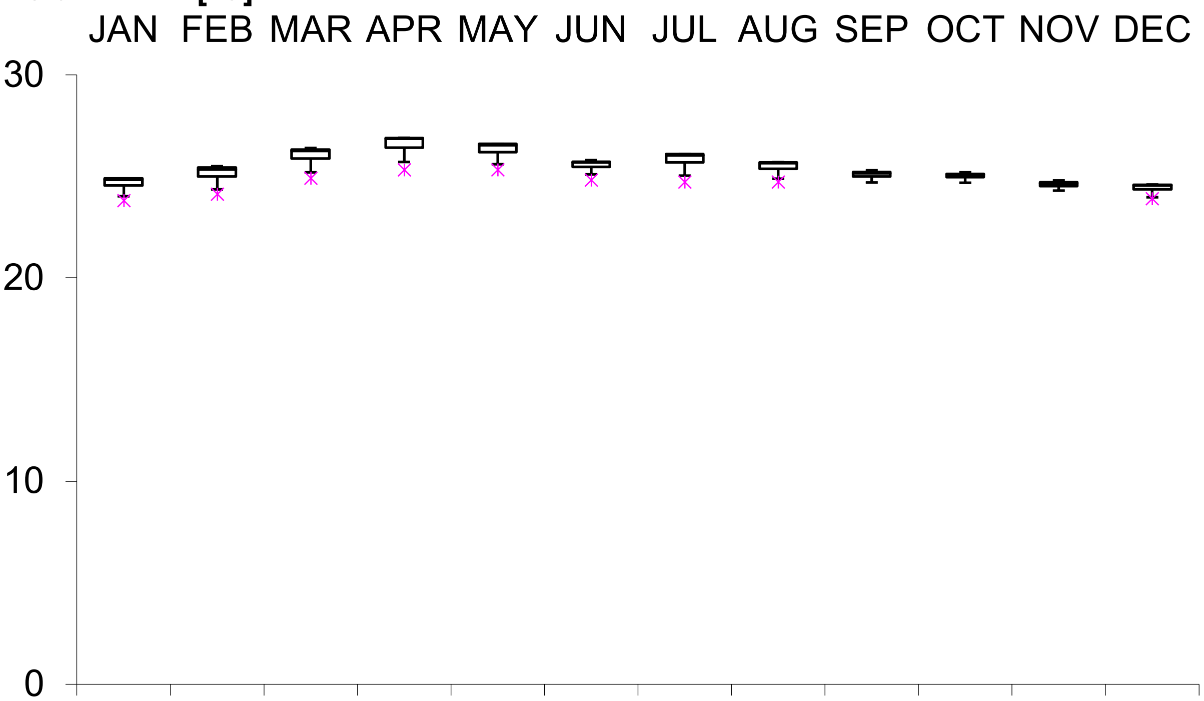

4.8.3.2. MinMT [°C]

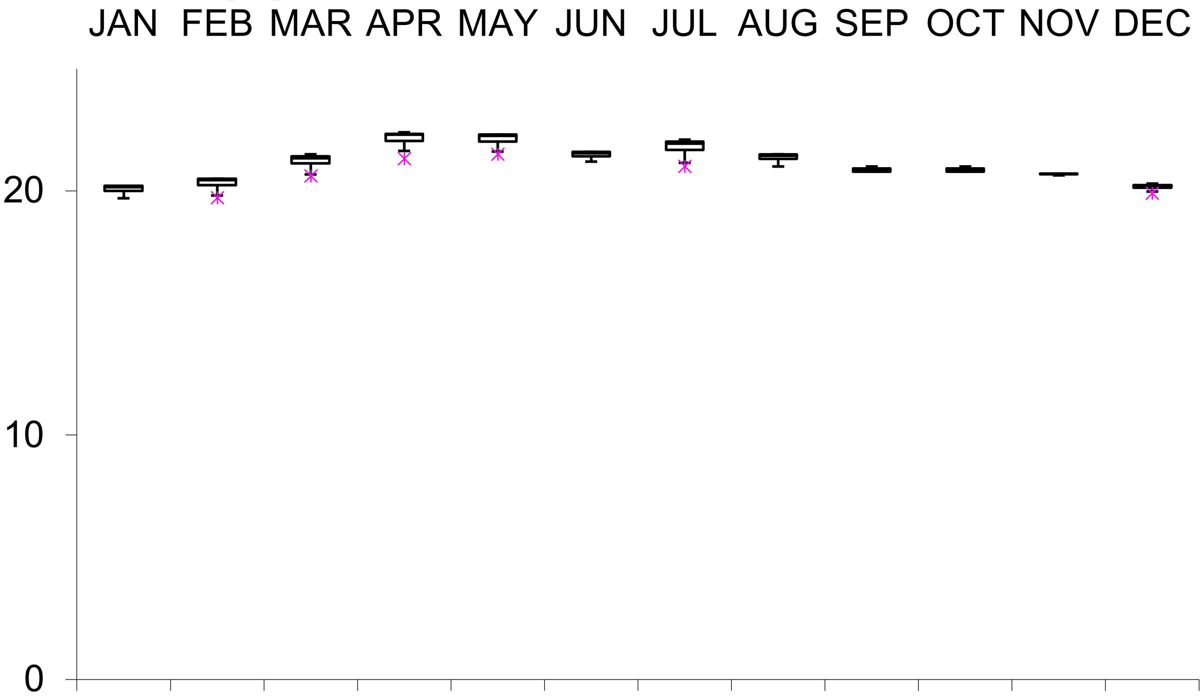

4.8.3.3. MMP [mm]

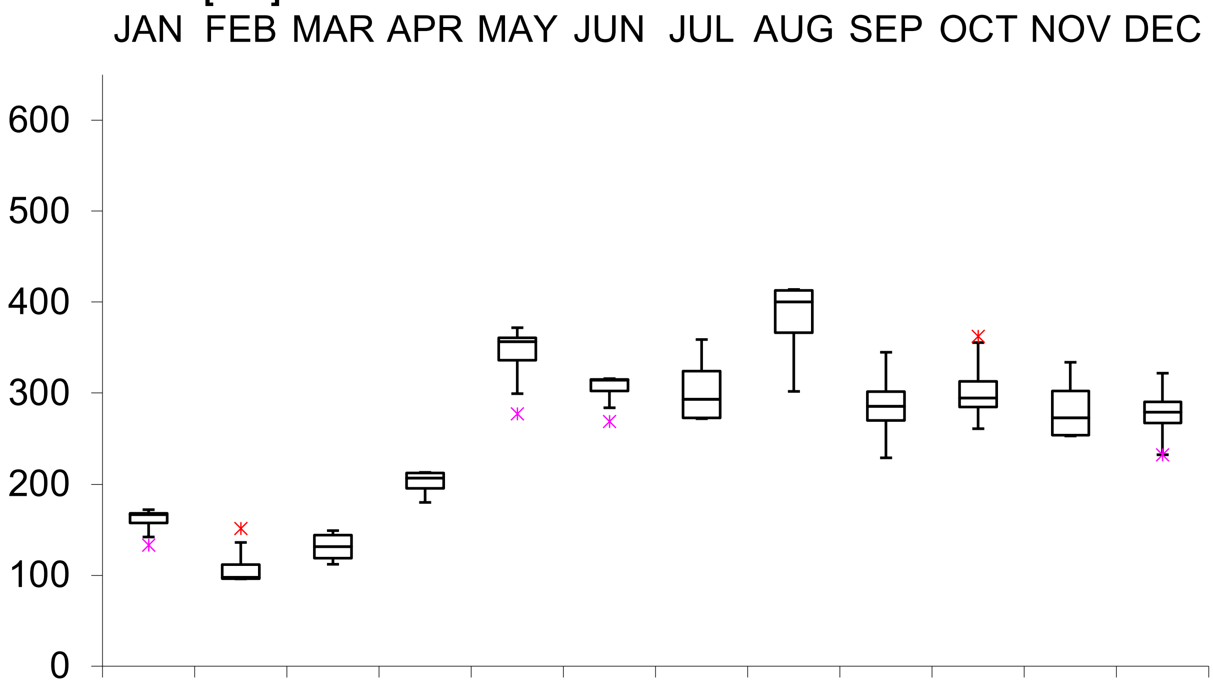
JAN FEB MAR APR MAY JUN JUL AUG SEP OCT NOV DEC
600
500
400
300
200
100
0

4.9. Species *Mortoniodendron membranaceum* Standl. et Steyerm., 1940

4.9.1. Köppen profile, distribution, and climate map – GBIF occurrences of *Mortoniodendron membranaceum*; herbarium specimens (n = 2).

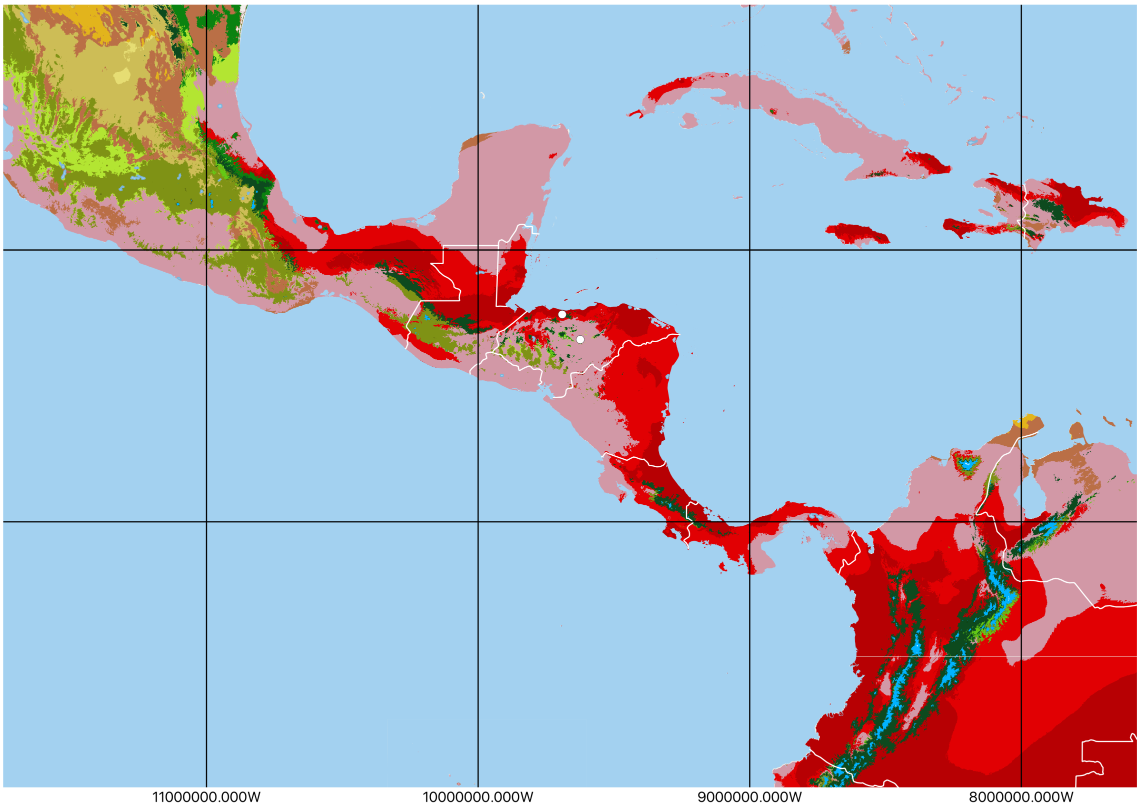

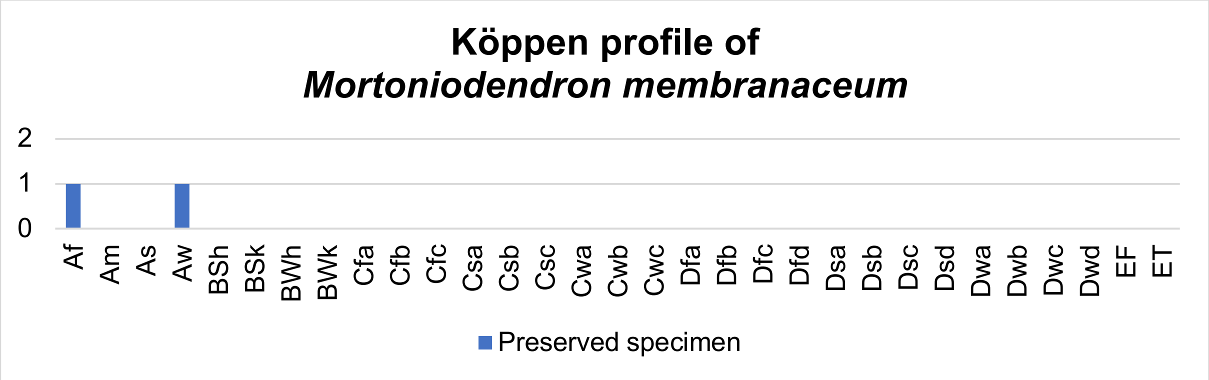

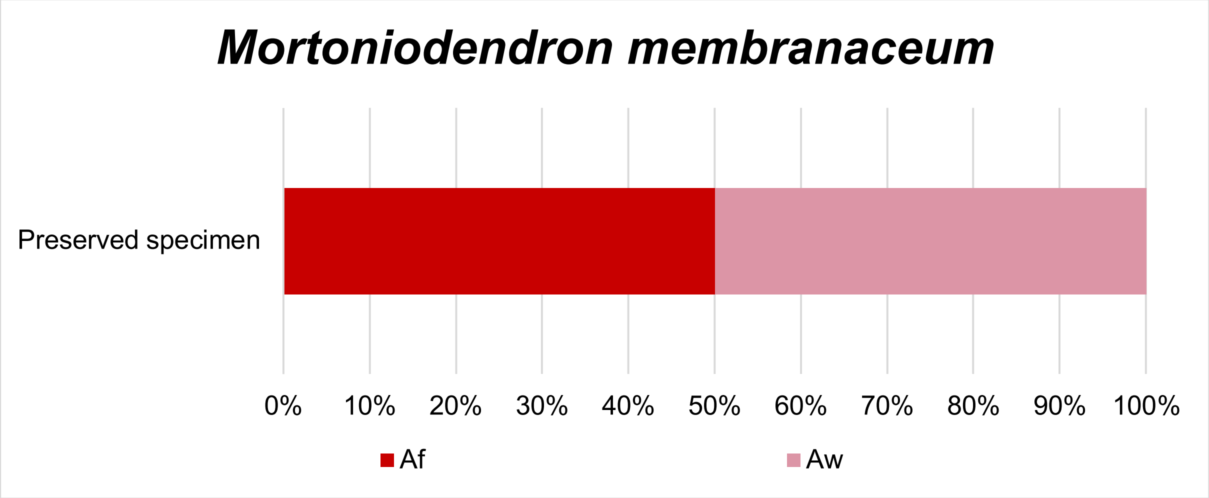

4.9.2. Biome profile, distribution, and biome map – GBIF occurrences of *Mortoniodendron membranaceum*; herbarium specimens (n = 2).

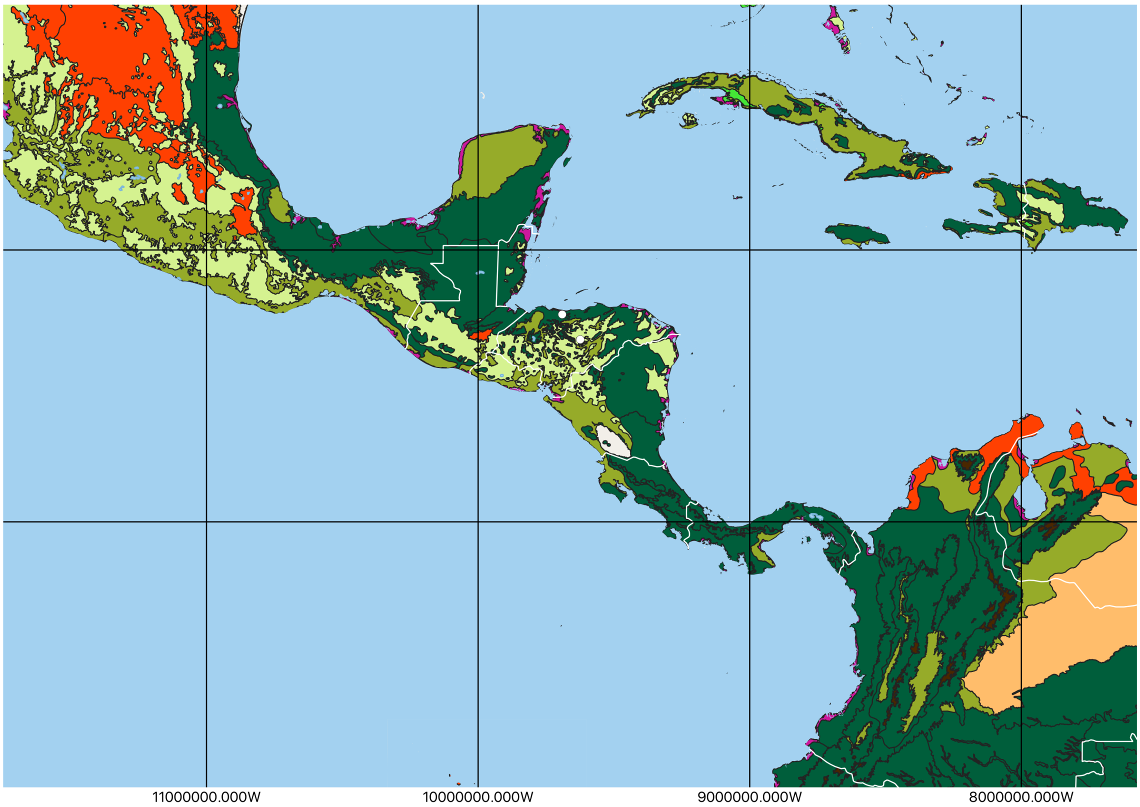

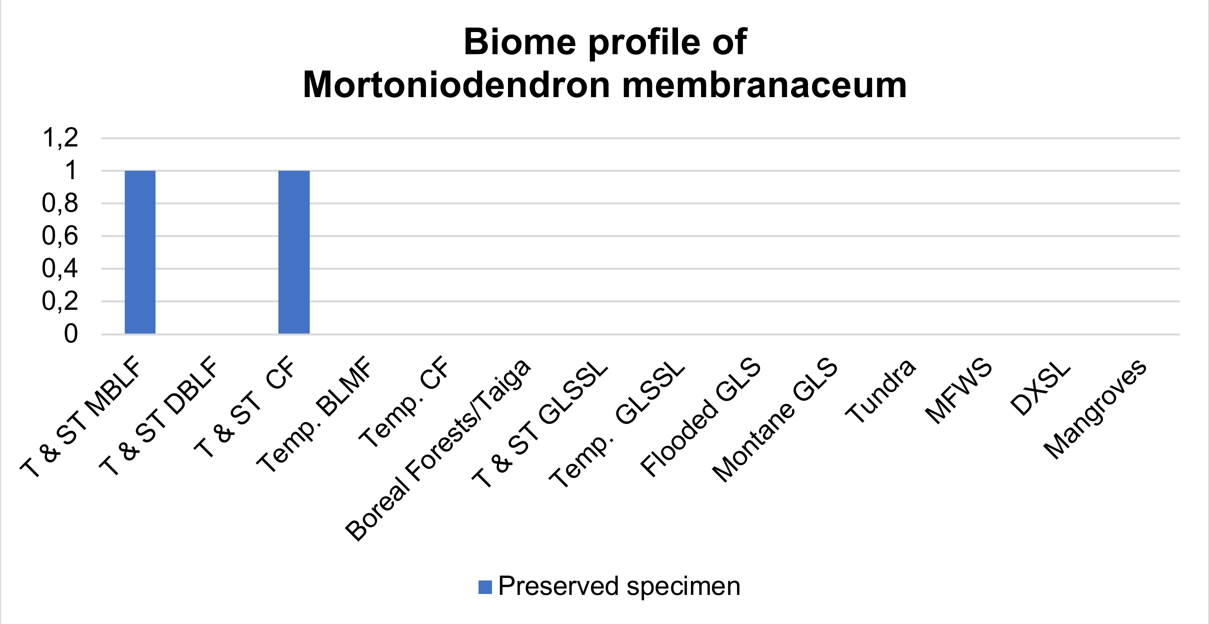

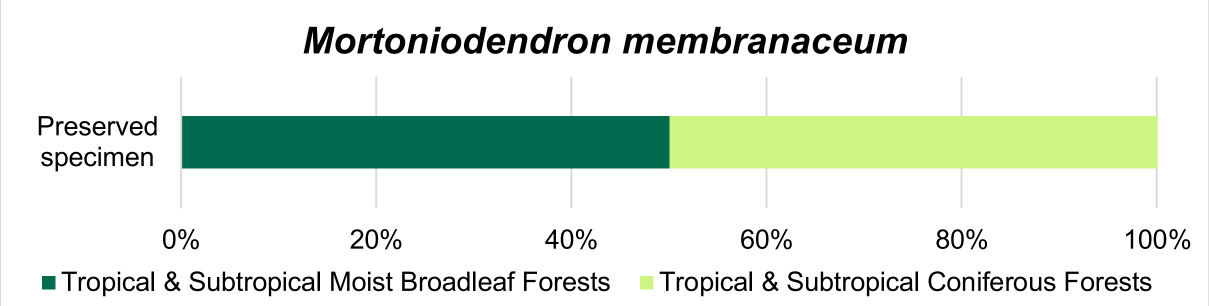

4.9.3. Climate graphs - based on 2 *Mortoniodendron membranaceum* occurrences in GBIF

4.9.3.1. MMT [°C]

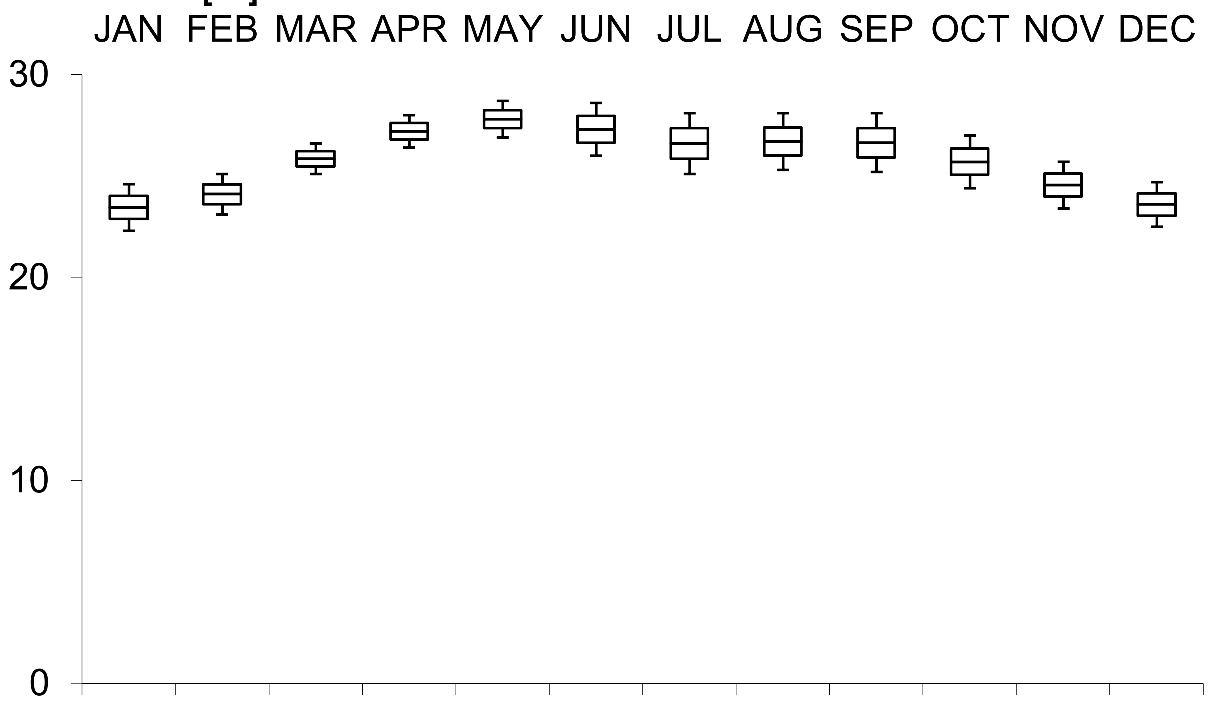

4.9.3.2. MinMT [°C]

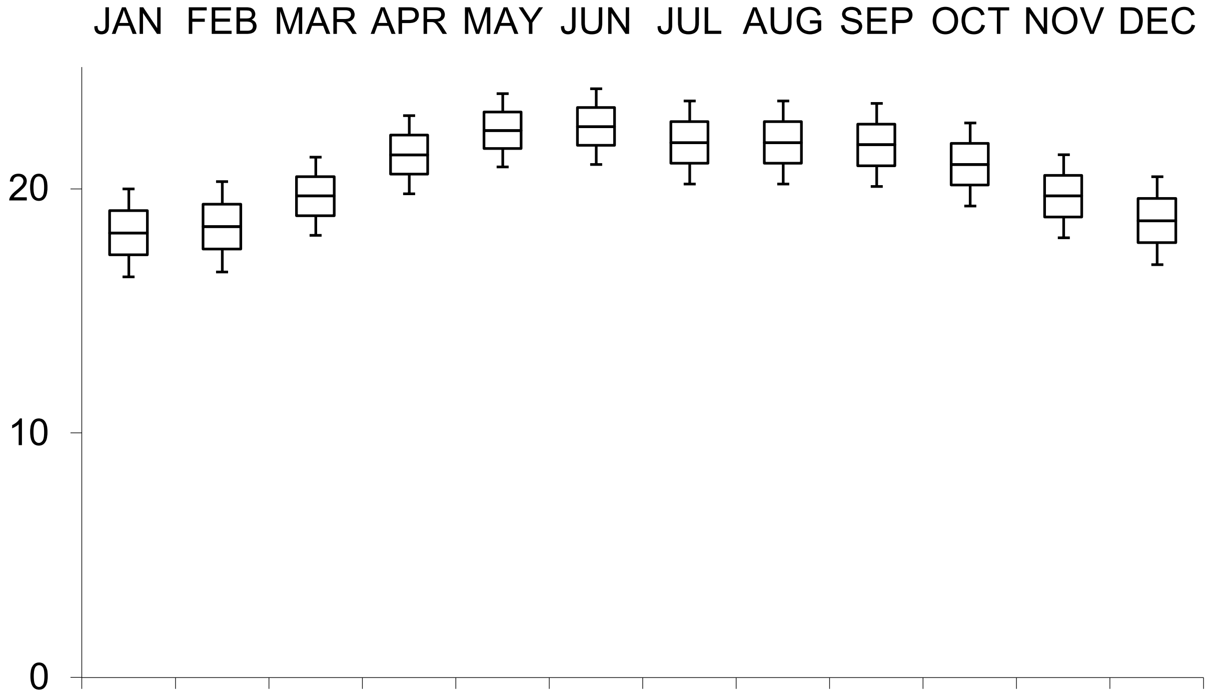

4.9.3.3. MMP [mm]

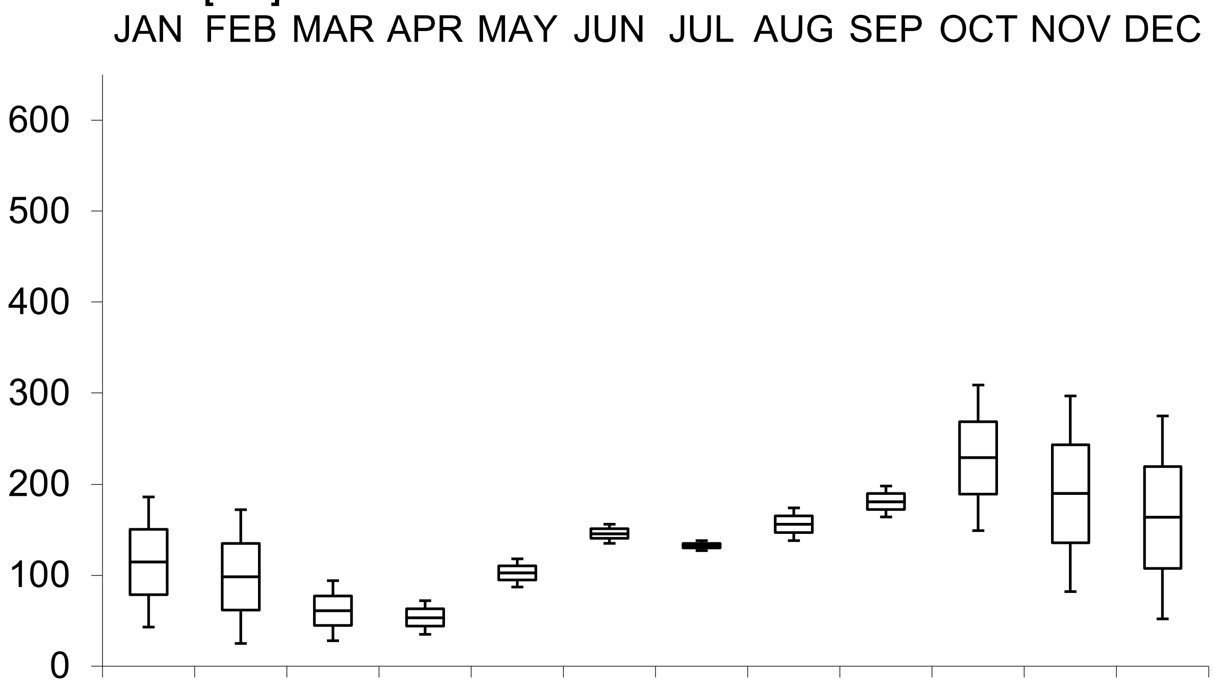
JAN FEB MAR APR MAY JUN JUL AUG SEP OCT NOV DEC
600
500
400
300
200
100
0

4.10. Species *Mortoniodendron ocotense* Ishiki & T.Wendt, 2014

4.10.1. Köppen profile, distribution, and climate map – GBIF occurrences of *Mortoniodendron ocotense*; excluding duplicate occurrences (n = 3), herbarium specimens (n = 1).

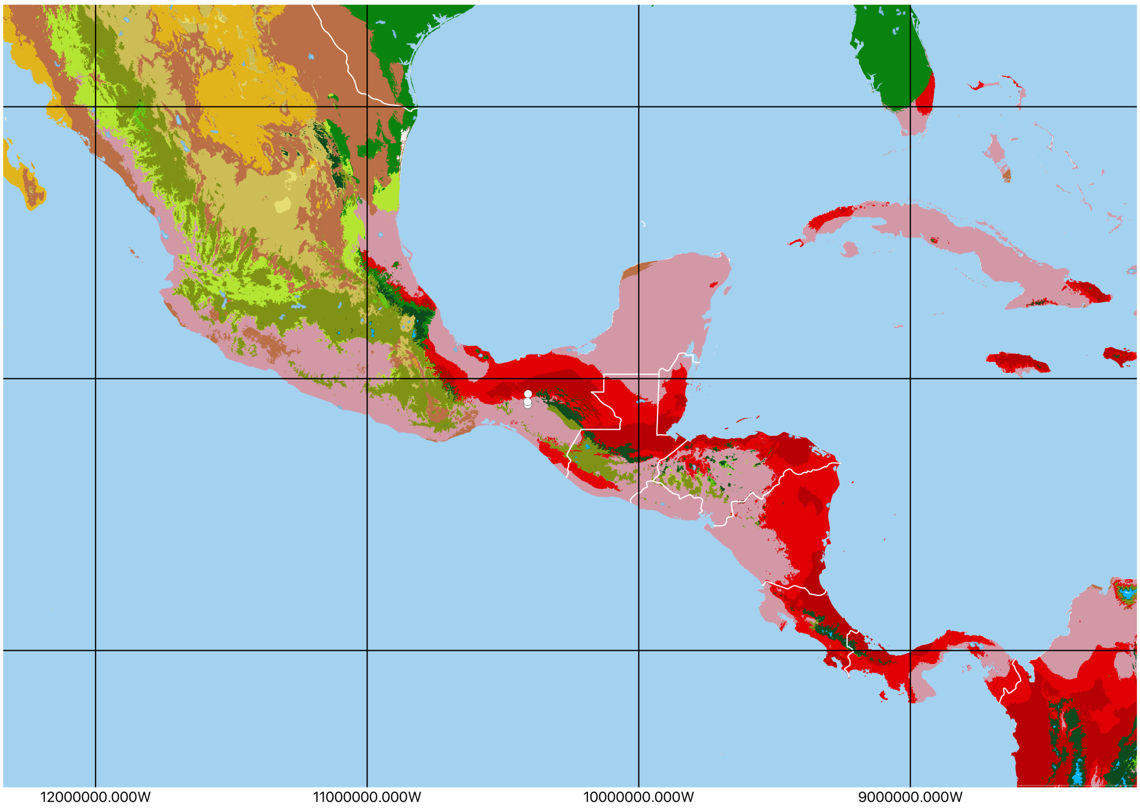

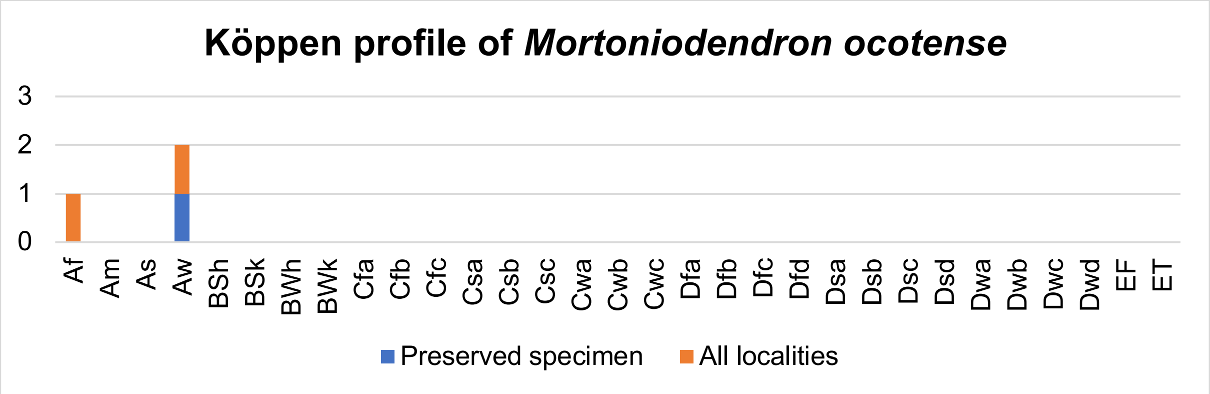

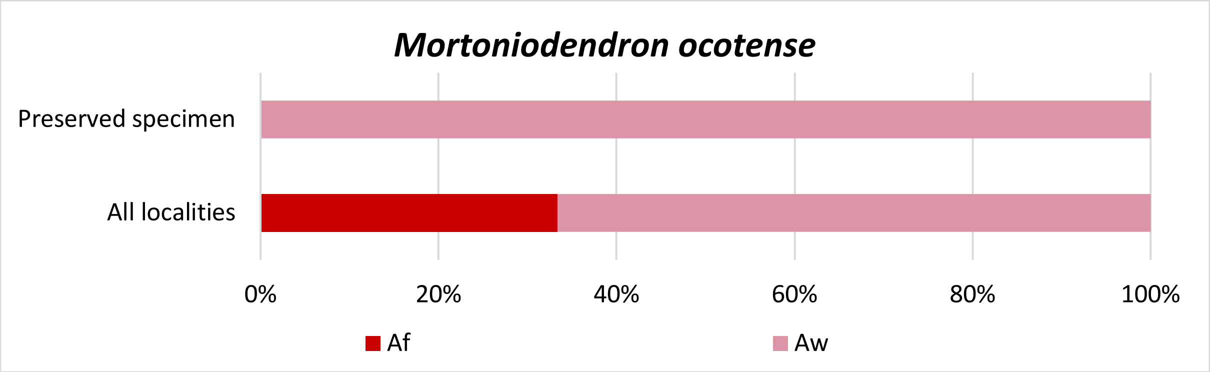

4.10.2. Biome profile, distribution, and biome map – GBIF occurrences of *Mortoniodendron ocotense*; excluding duplicate occurrences (n = 3), herbarium specimens (n = 1).

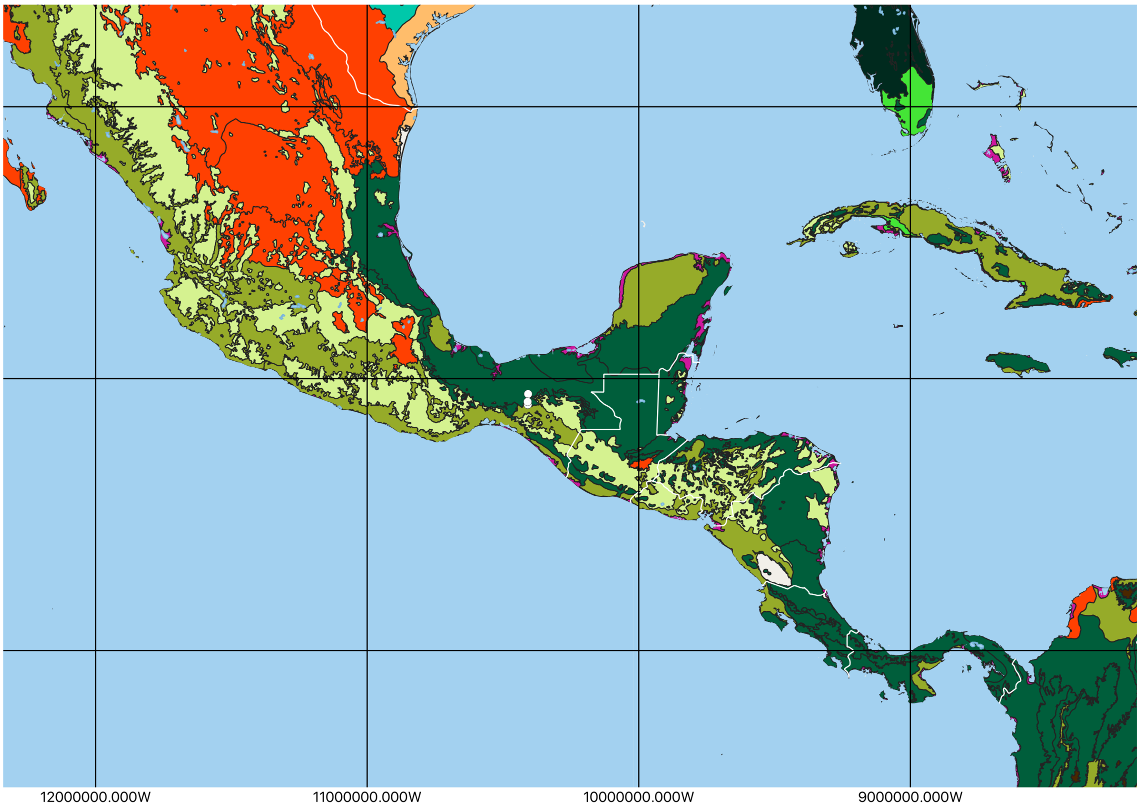

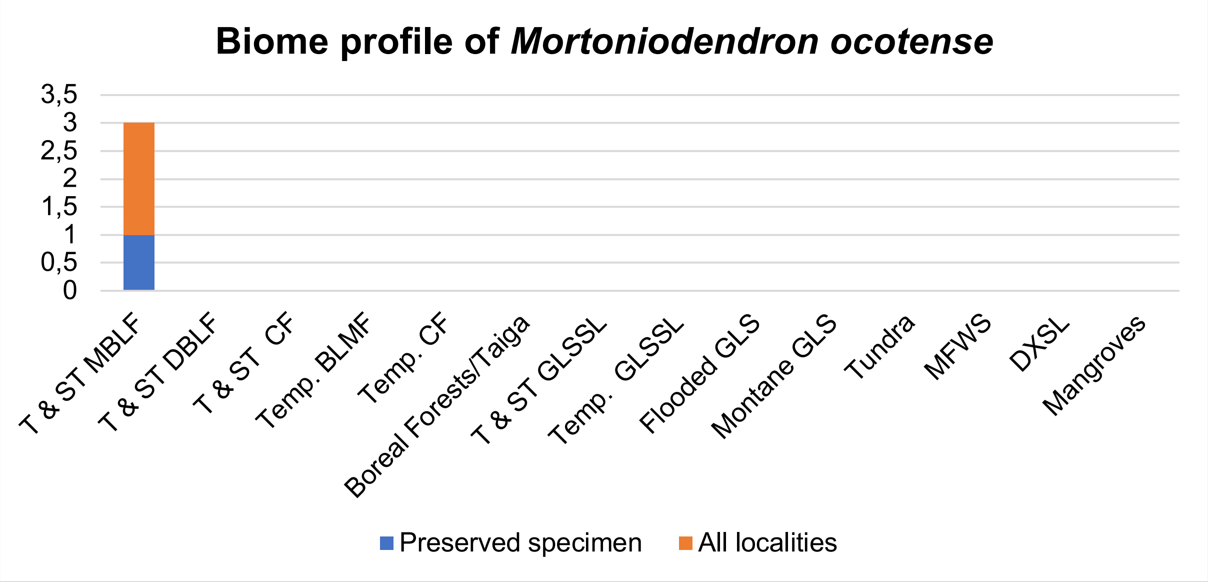

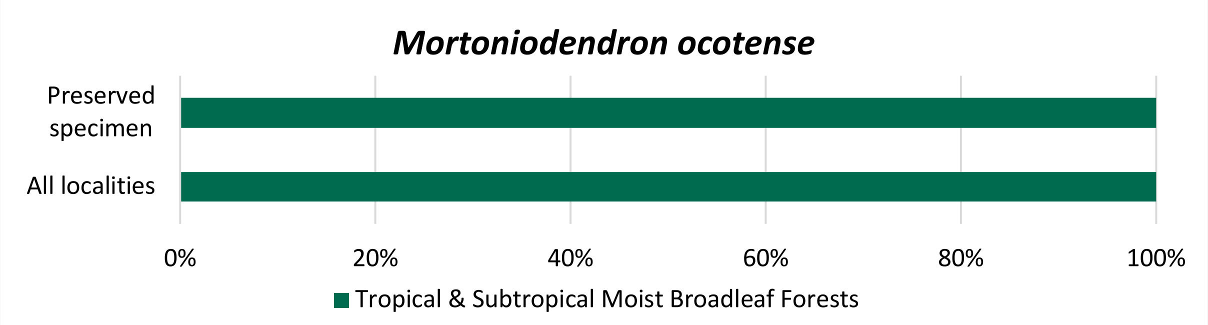

4.10.3. Climate graphs - based on 3 *Mortoniodendron ocotense* occurrences in GBIF

4.10.3.1. MMT [°C]

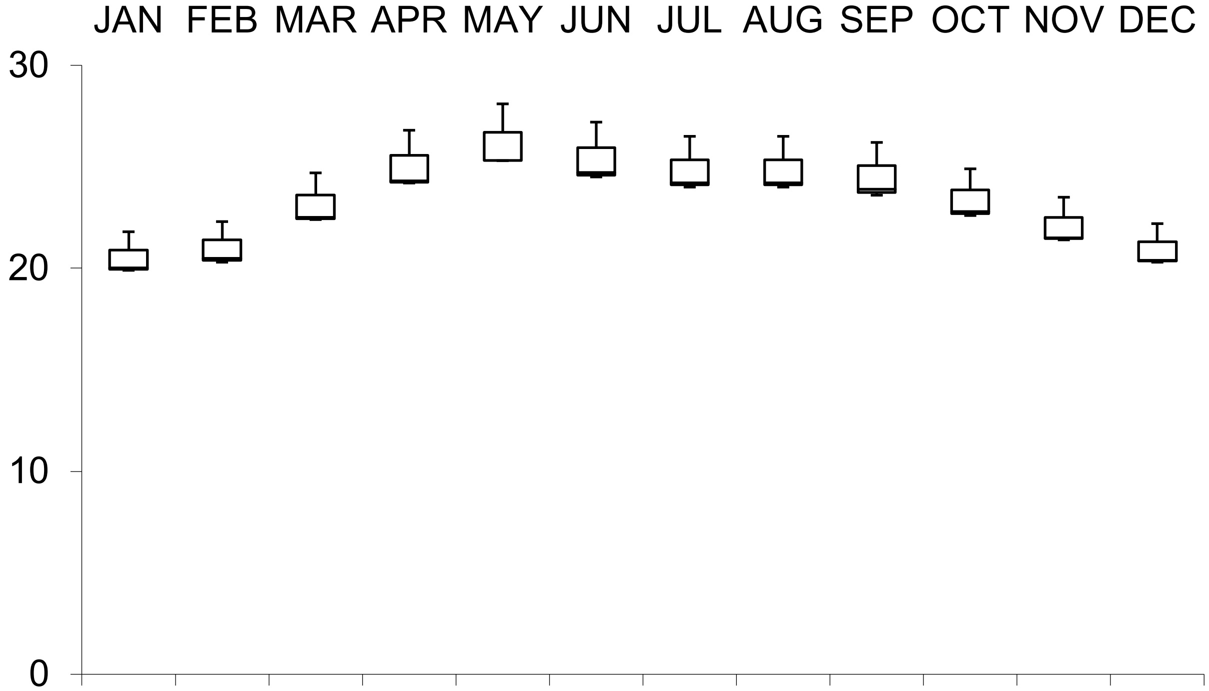

4.10.3.2. MinMT [°C]

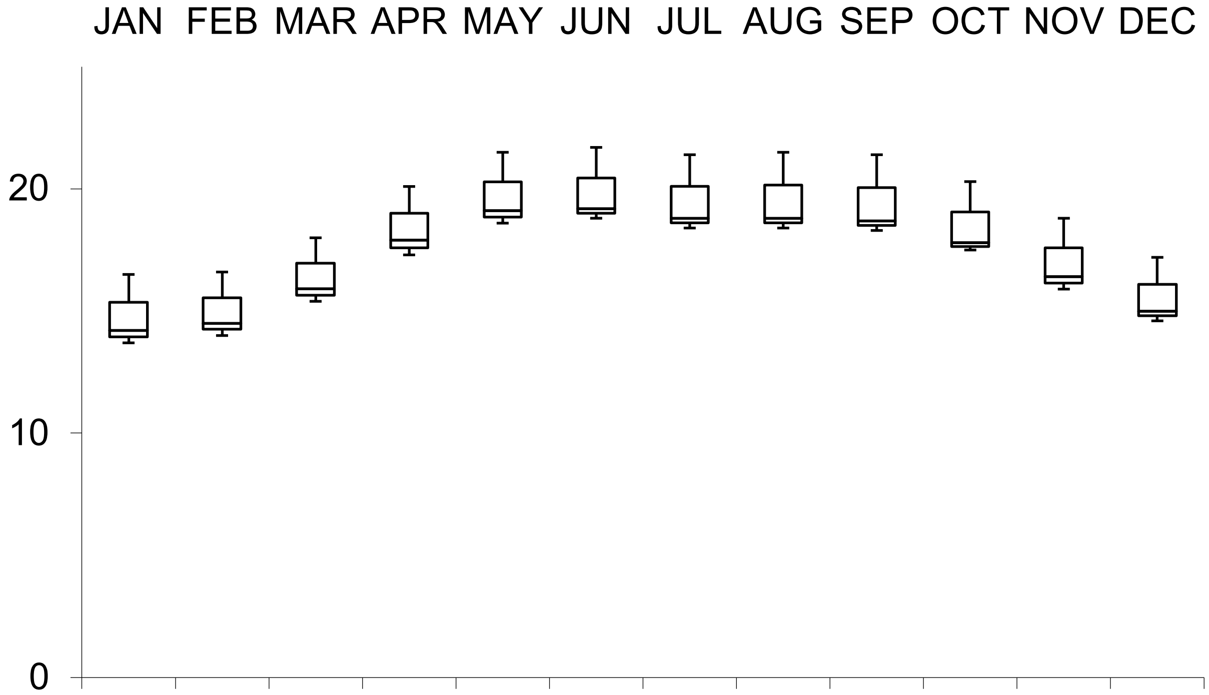

4.10.3.3. MMP [mm]

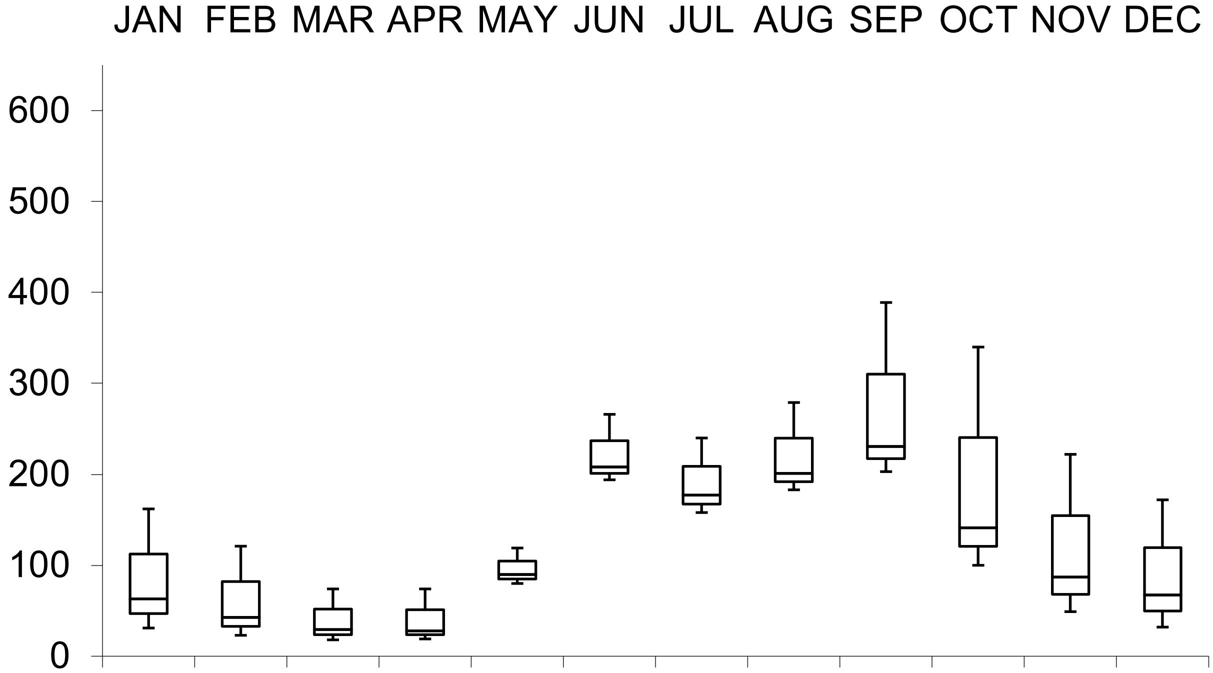
JAN FEB MAR APR MAY JUN JUL AUG SEP OCT NOV DEC
600
500
400
300
200
100
0

4.11. Species *Mortoniodendron palaciosii* Miranda, 1957

4.11.1. Köppen profile, distribution, and climate map – GBIF occurrences of *Mortoniodendron palaciosii*; herbarium specimens (n = 9).

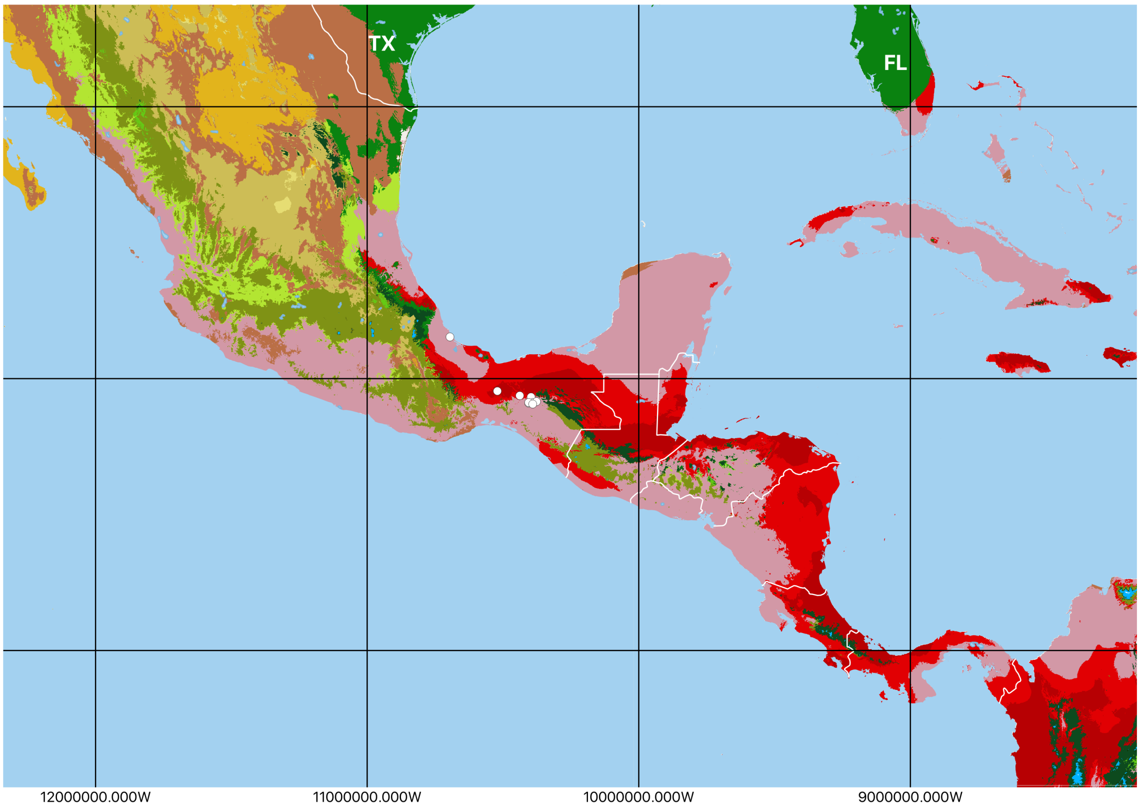

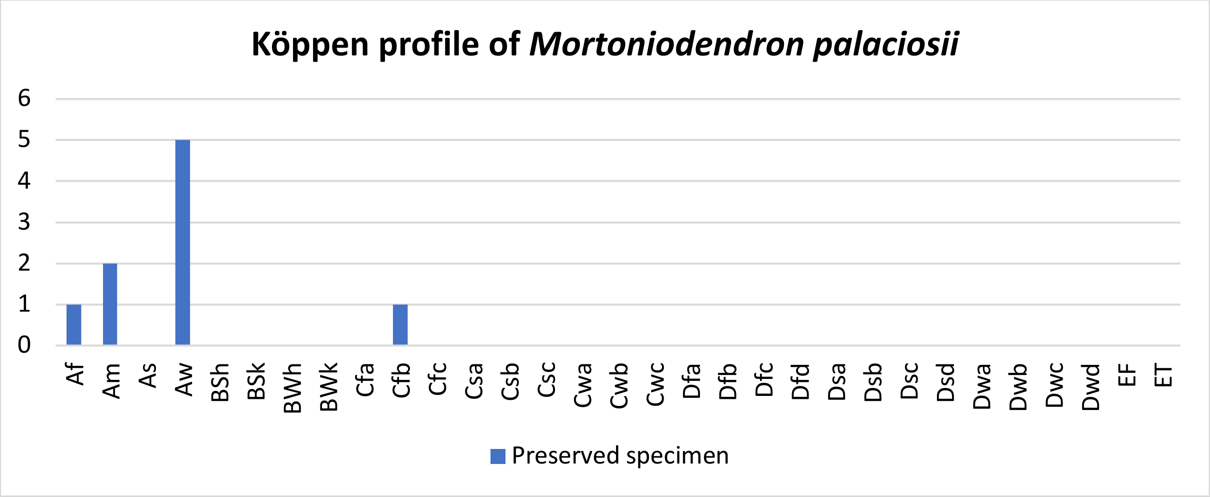

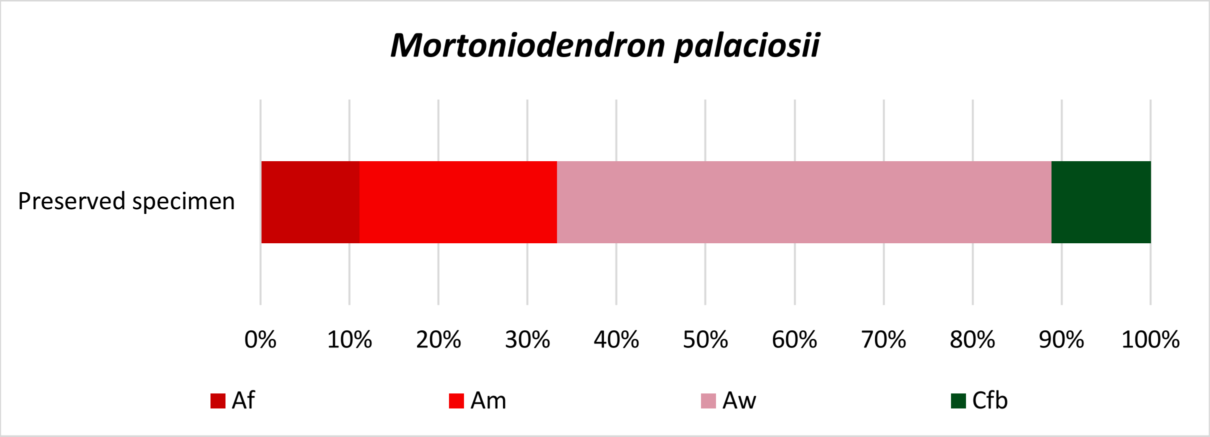

4.11.2. Biome profile, distribution, and biome map – GBIF occurrences of *Mortoniodendron palaciosii*; herbarium specimens (n = 9).

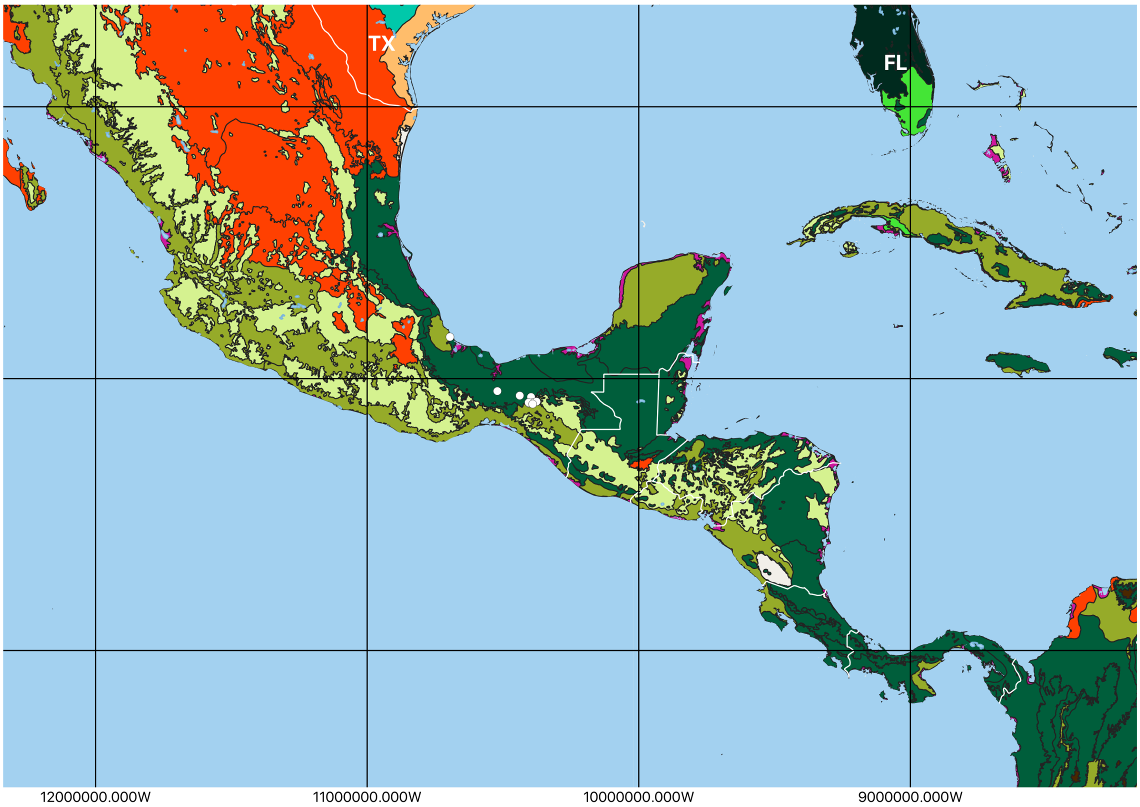

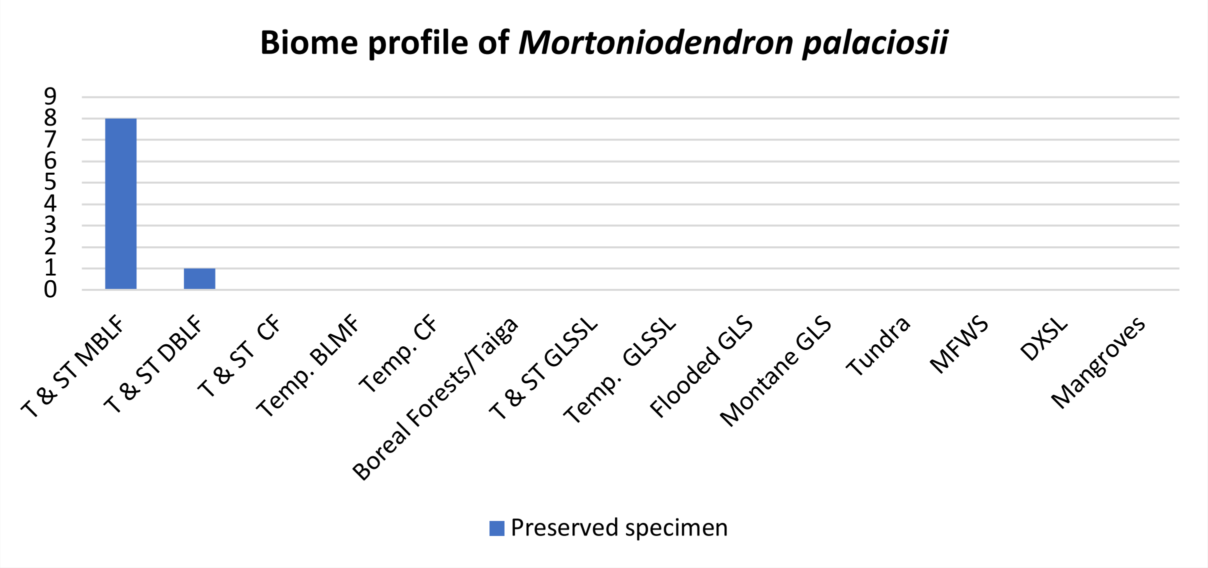

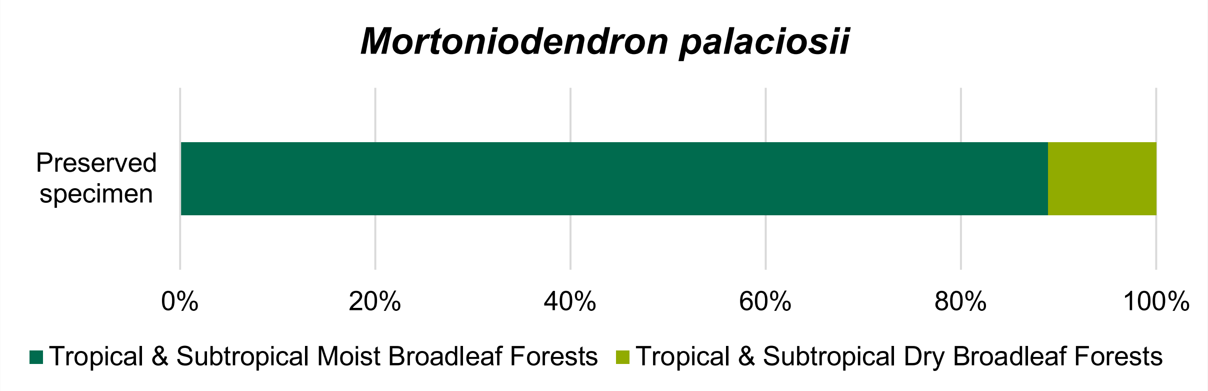

4.11.3. Climate graphs - based on 9 *Mortoniodendron palaciosii* occurrences in GBIF

4.11.3.1. MMT [°C]

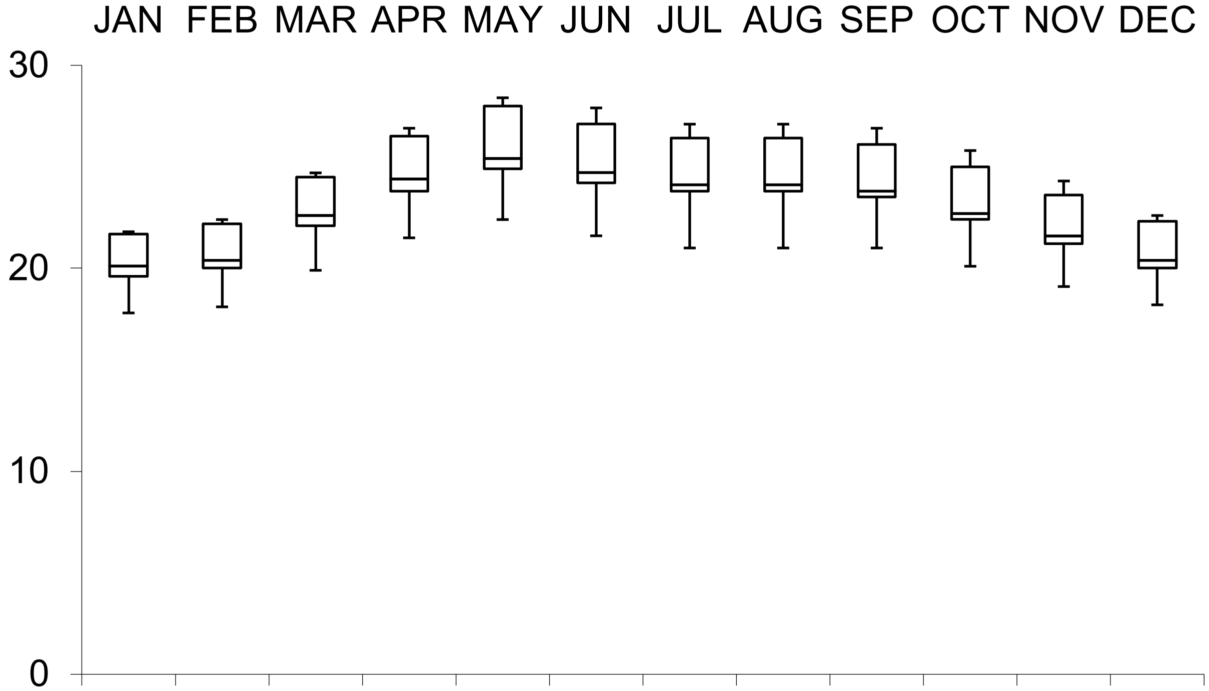

4.11.3.2. MinMT [°C]

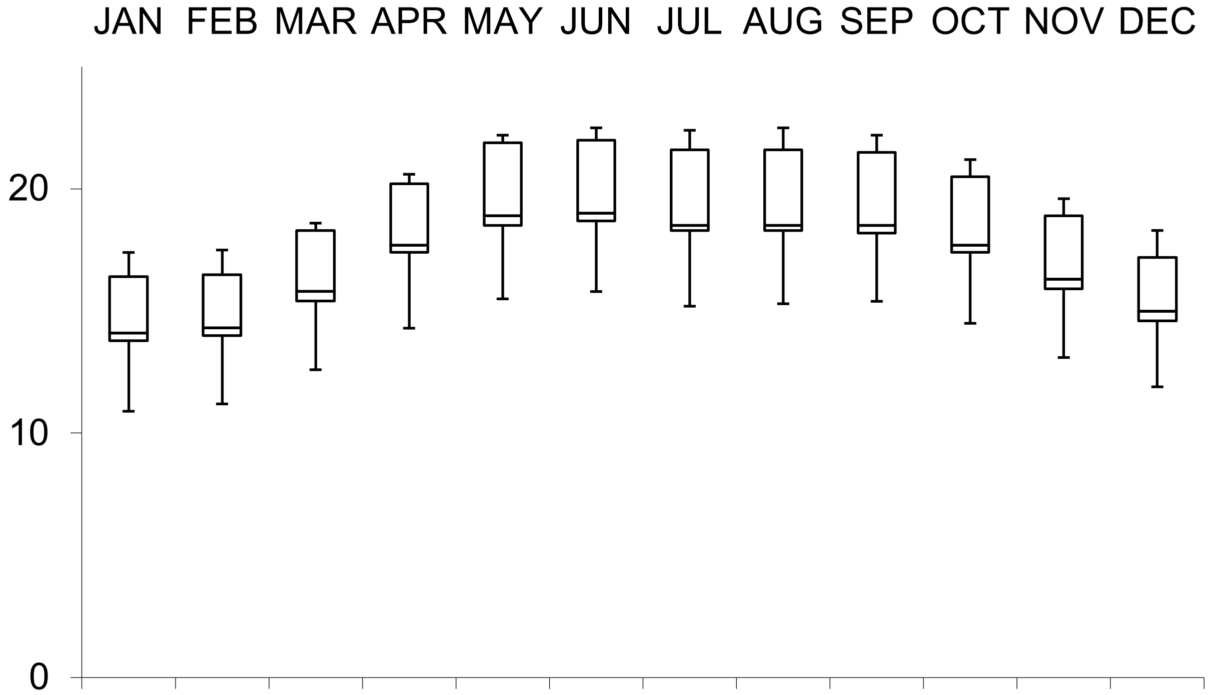

4.11.3.3. MMP [mm]

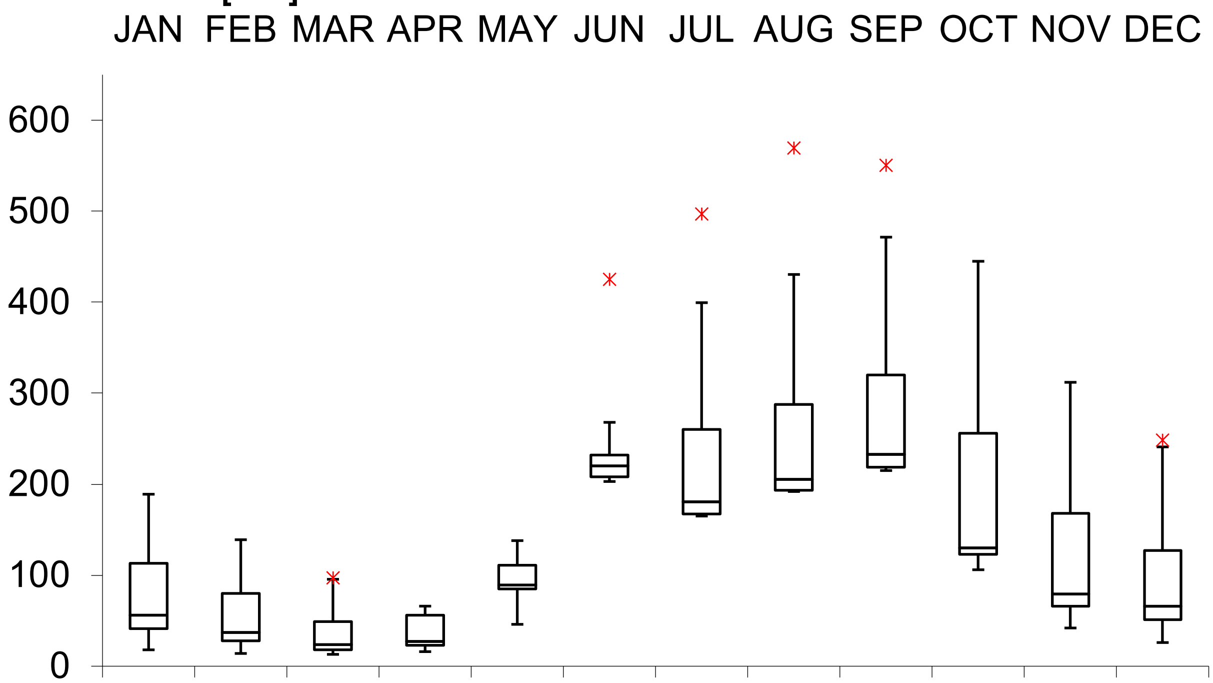
JAN FEB MAR APR MAY JUN JUL AUG SEP OCT NOV DEC
600
500
400
300
200
100
0

4.12. Species *Mortoniodendron pentagonum* (Donn.Sm., 1893) Miranda, 1965
4.12.1. Köppen profile, distribution, and climate map – GBIF occurrences of *Mortoniodendron pentagonum*; herbarium specimens (n = 9).

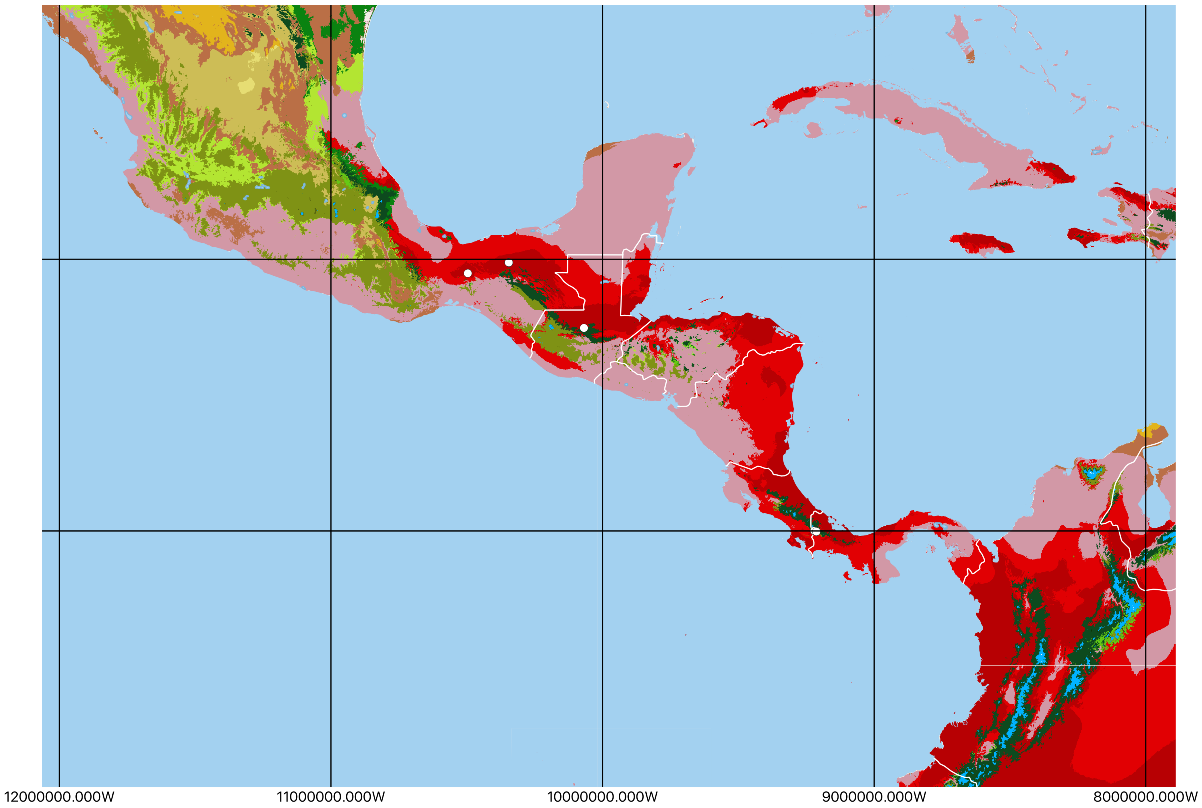

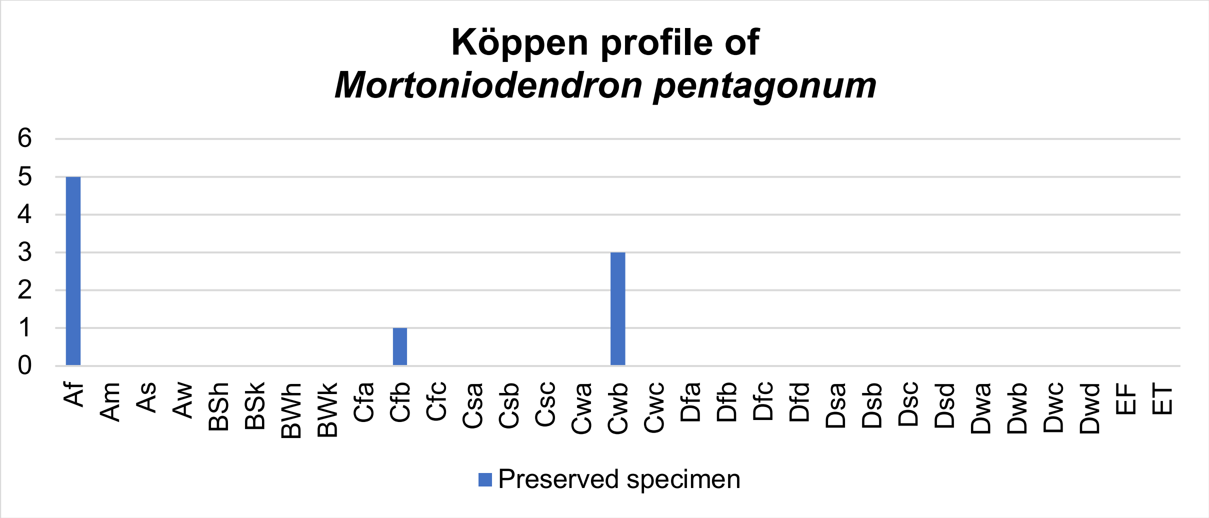

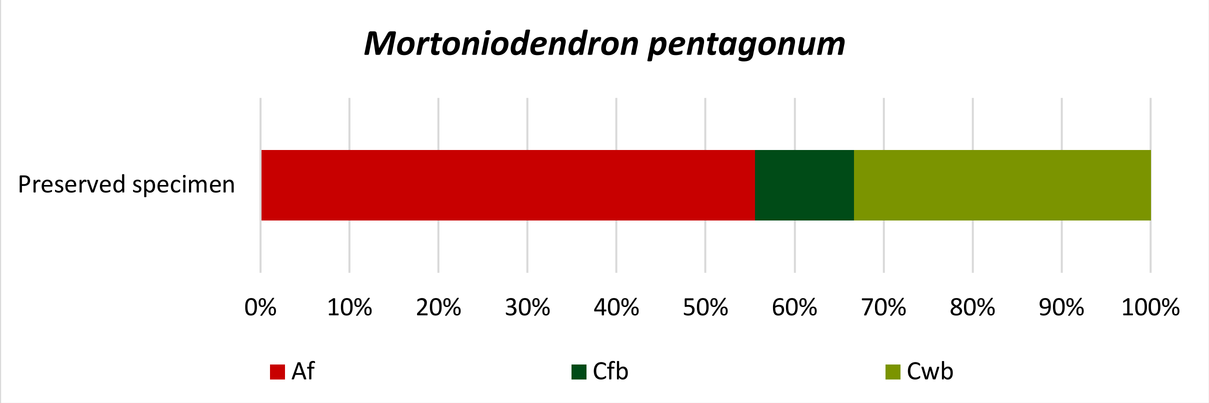

4.12.2. Biome profile, distribution, and biome map – GBIF occurrences of *Mortoniodendron pentagonum*; herbarium specimens (n = 9).

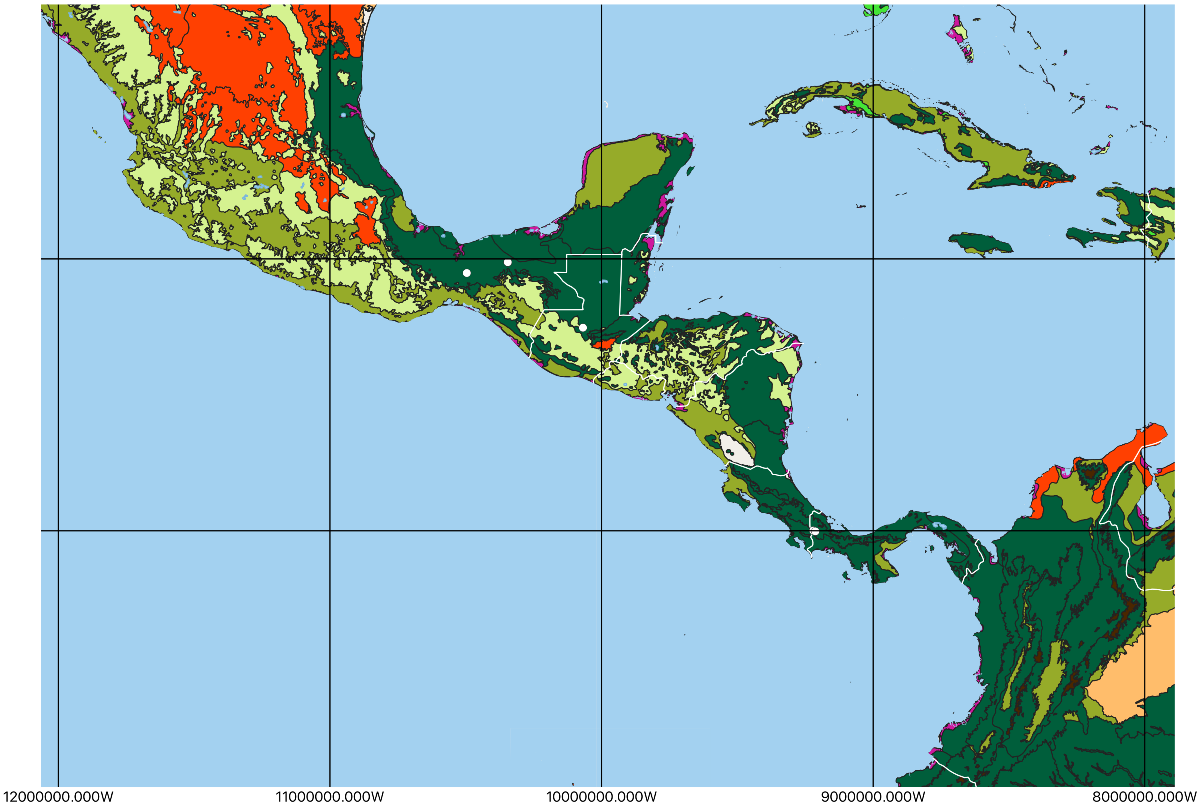

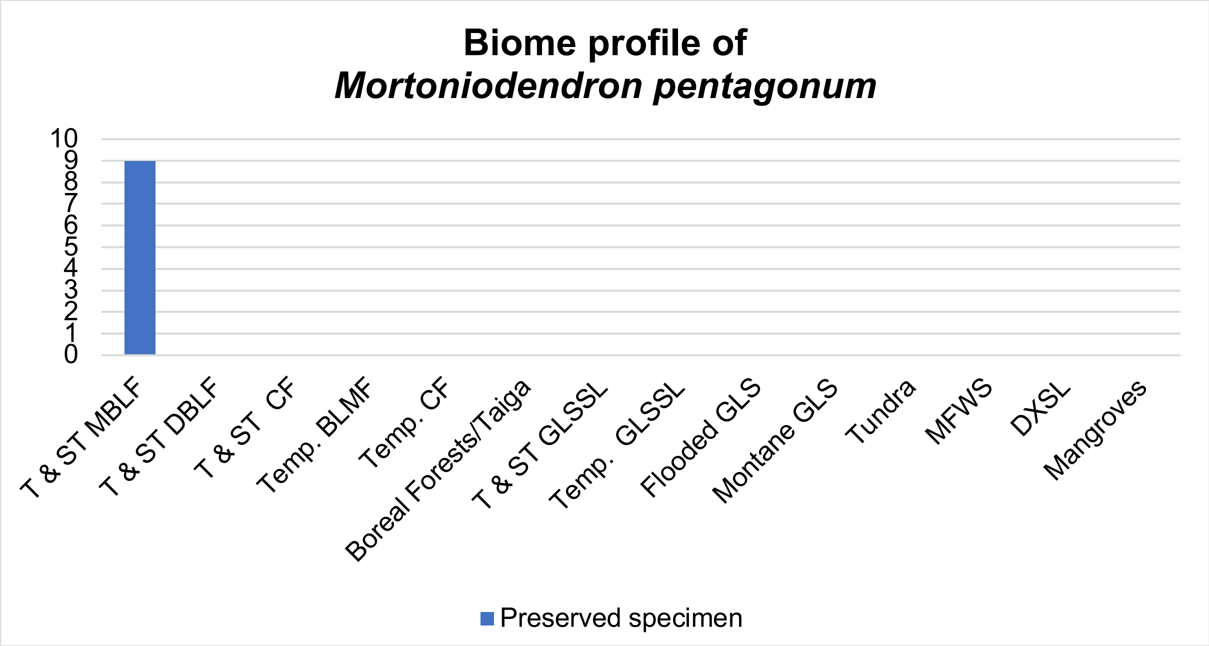

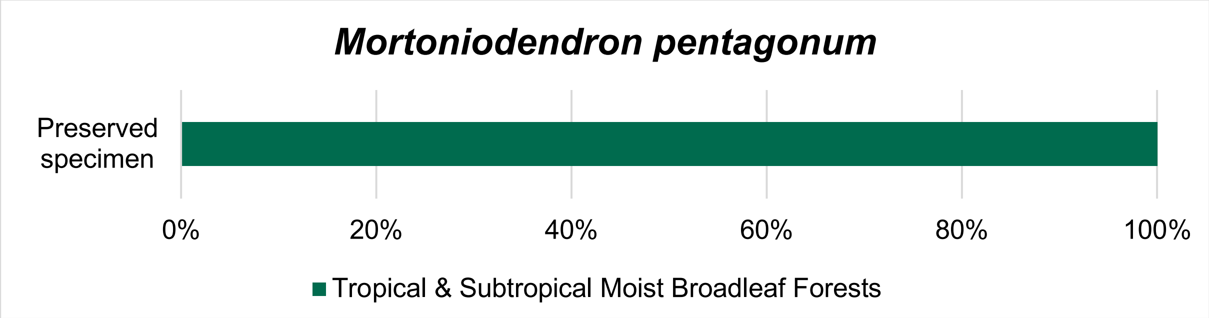

4.12.3. Climate graphs - based on 9 *Mortoniodendron pentagonum* occurrences in GBIF

4.12.3.1. MMT [°C]

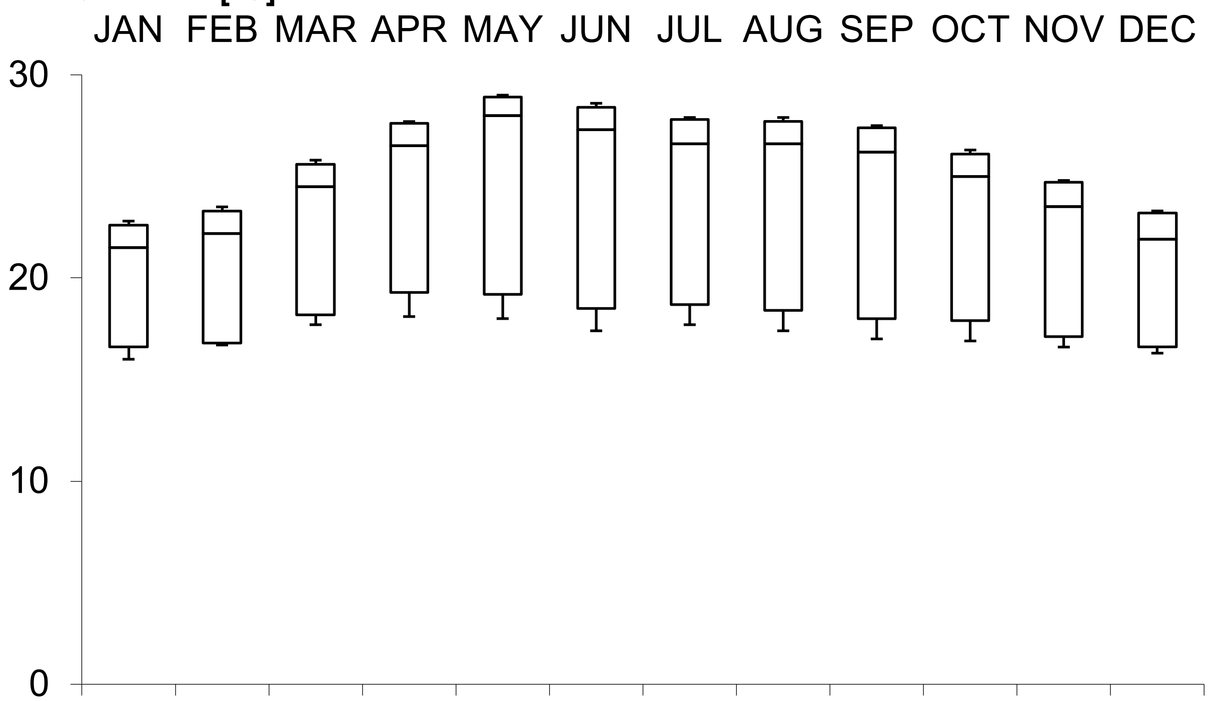

4.12.3.2. MinMT [°C]

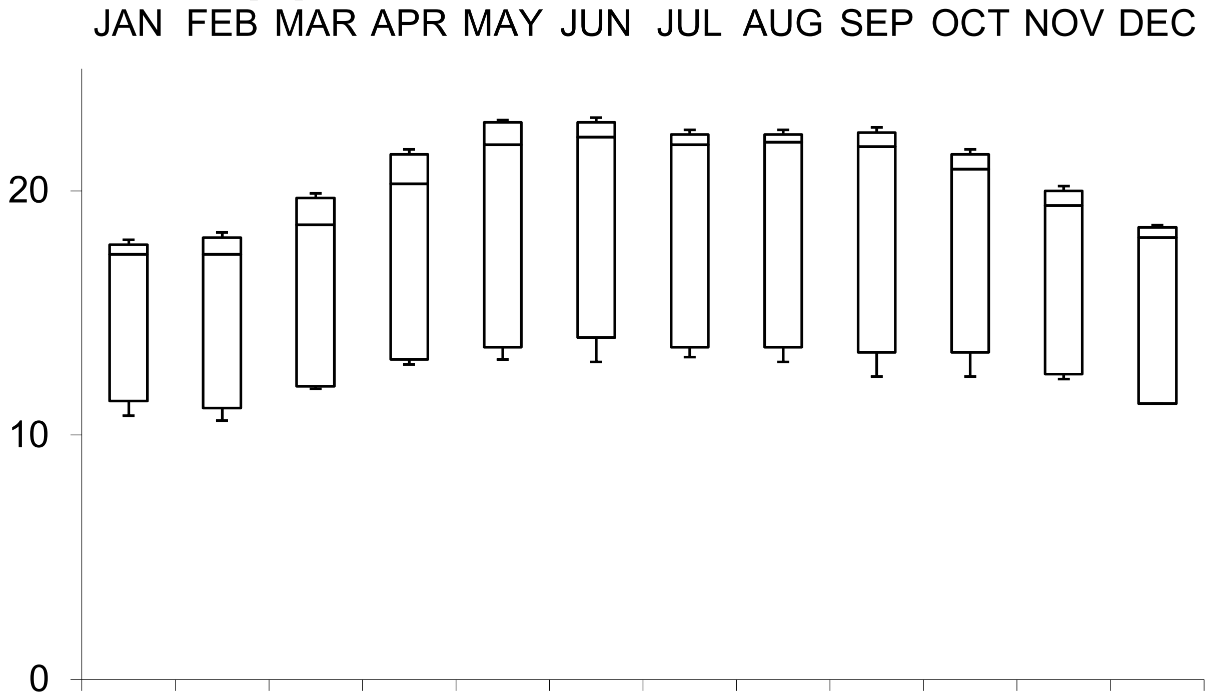

4.12.3.3. MMP [mm]

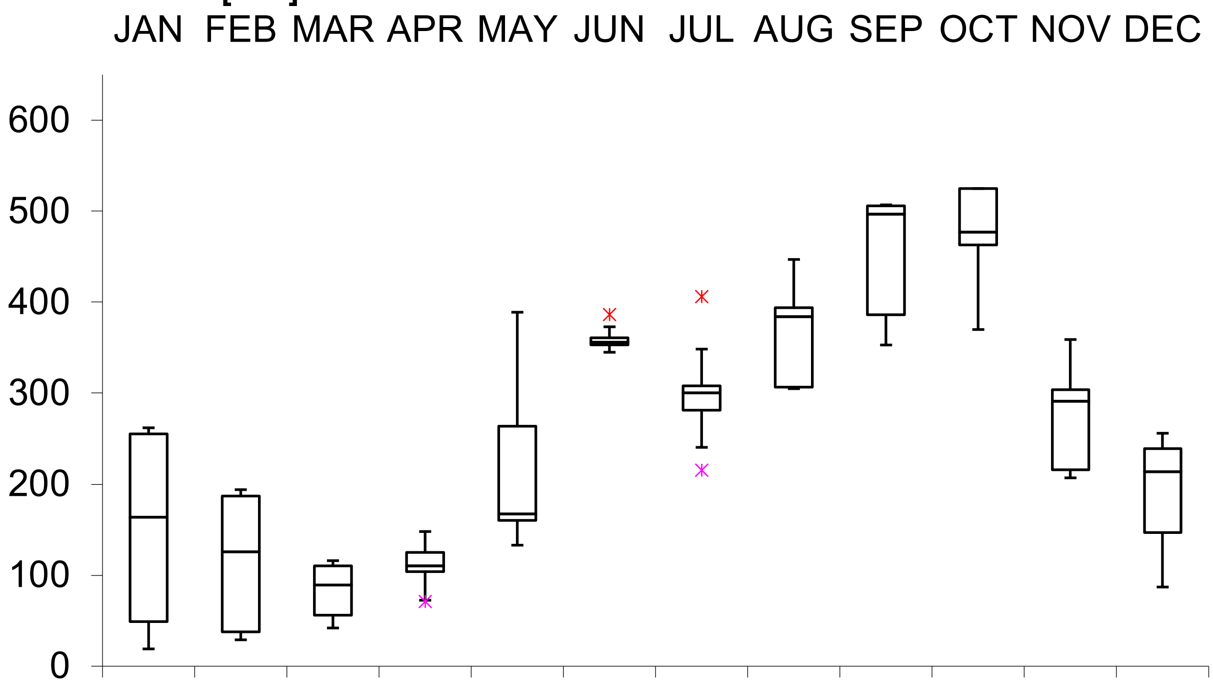

JAN FEB MAR APR MAY JUN JUL AUG SEP OCT NOV DEC
600
500
400
300
200
100
0

4.13. Species *Mortoniodendron ruizii* Miranda, 1957
4.13.1. Köppen profile, distribution, and climate map – GBIF occurrences of *Mortoniodendron ruizii*; herbarium specimens (n = 5).

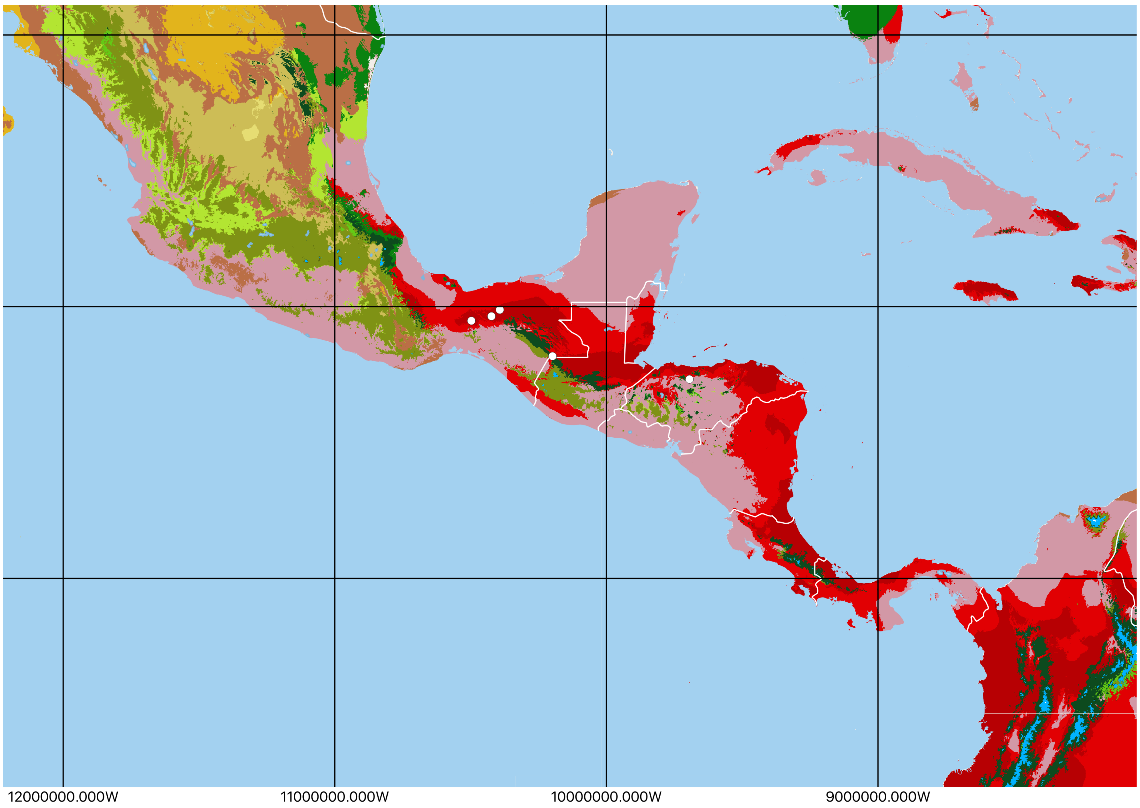

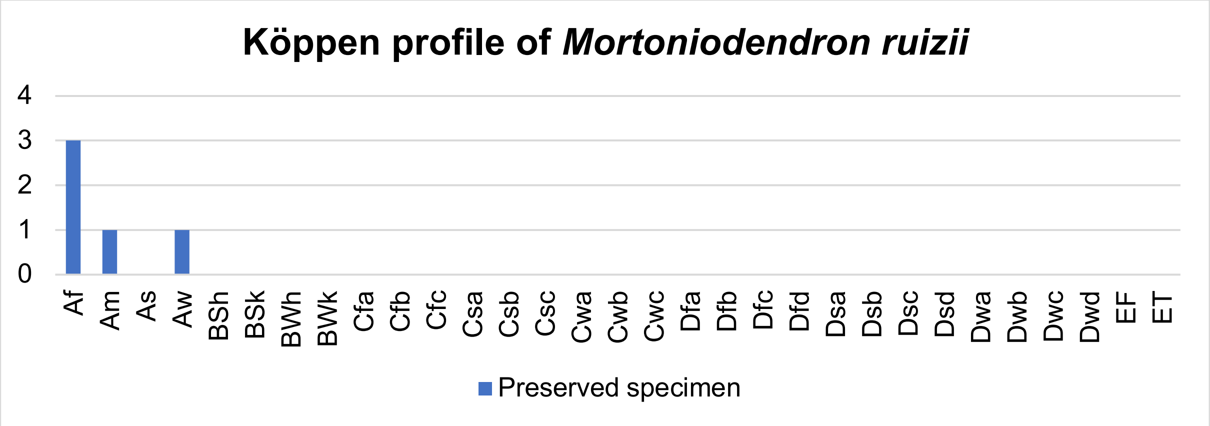

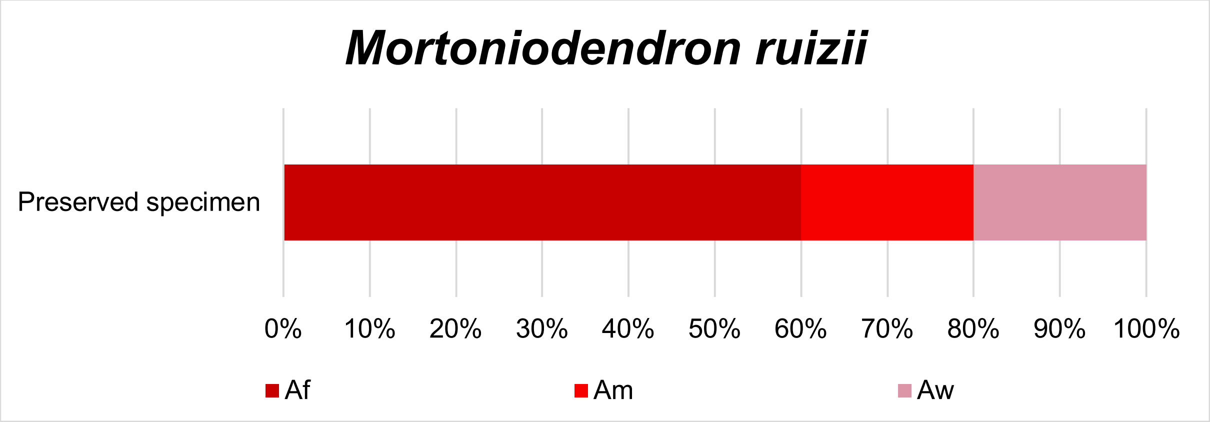

4.13.2. Biome profile, distribution, and biome map – GBIF occurrences of *Mortoniodendron ruizii*; herbarium specimens (n = 5).

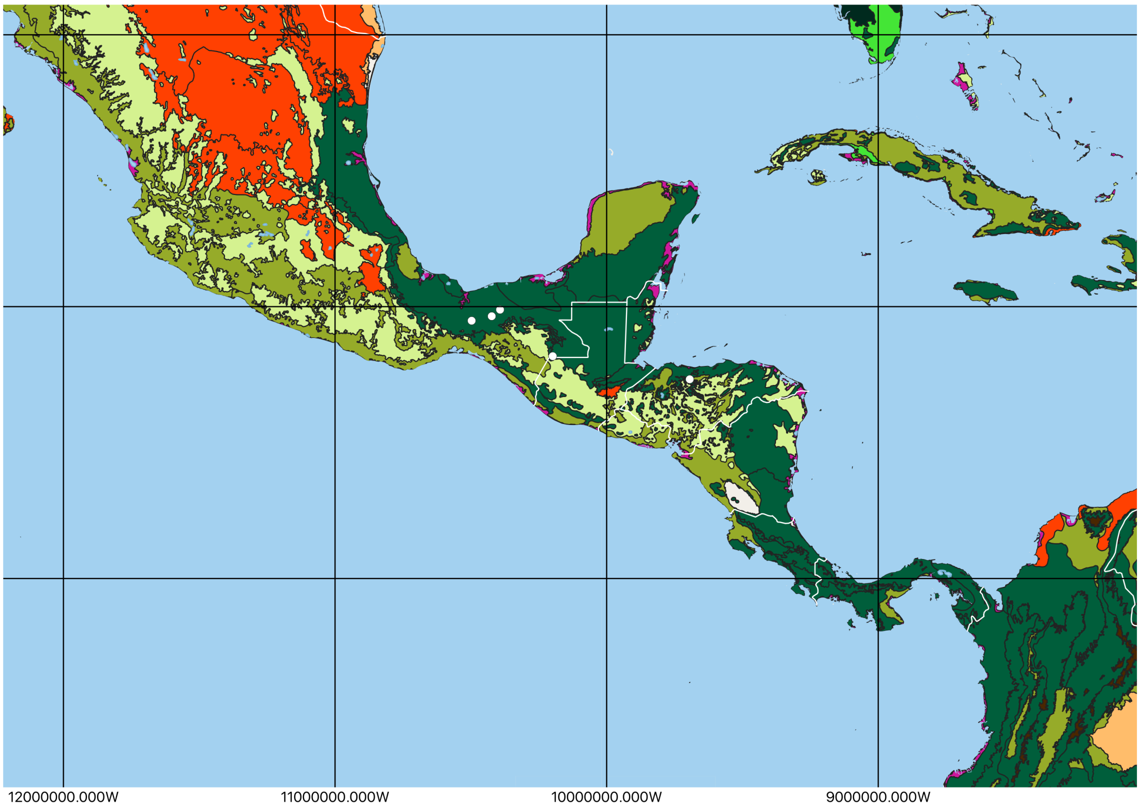

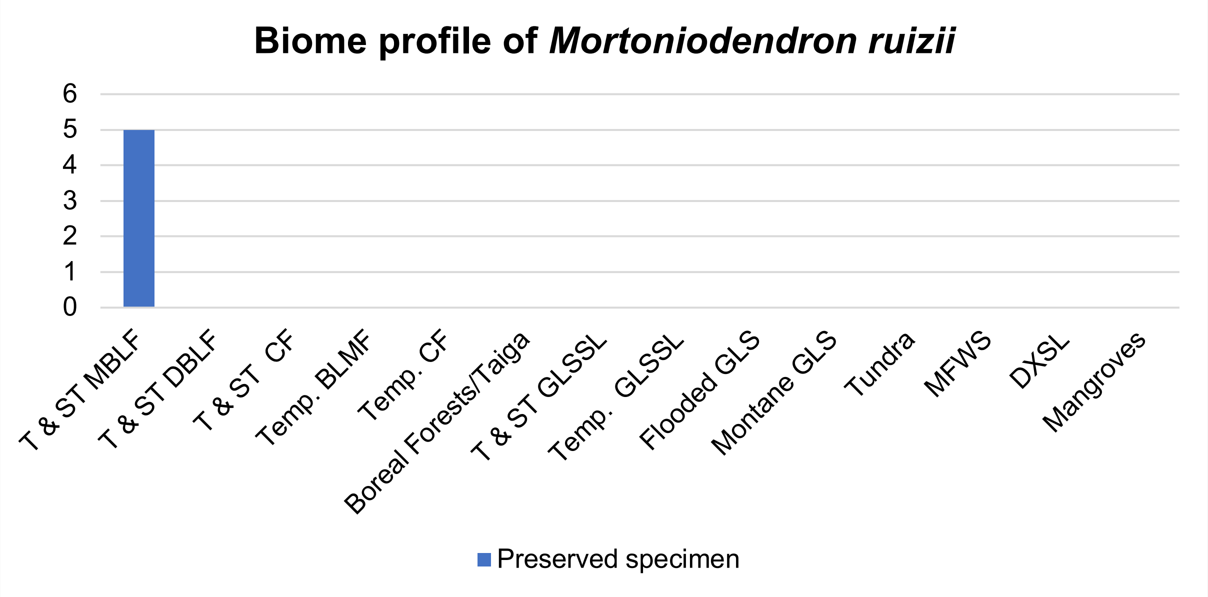

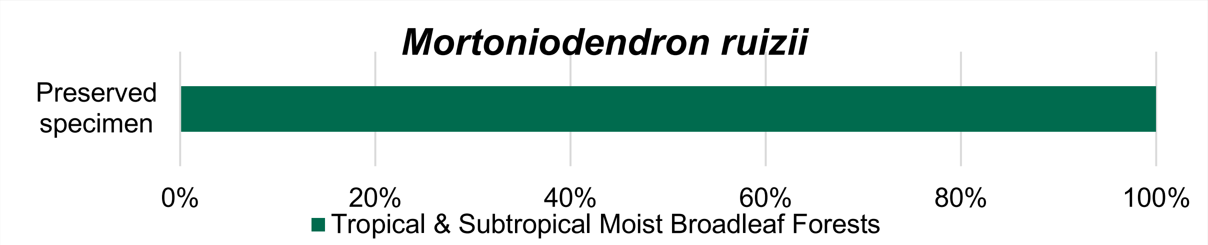

4.13.3. Climate graphs - based on 5 *Mortoniodendron ruizii* occurrences in GBIF

4.13.3.1. MMT [°C]

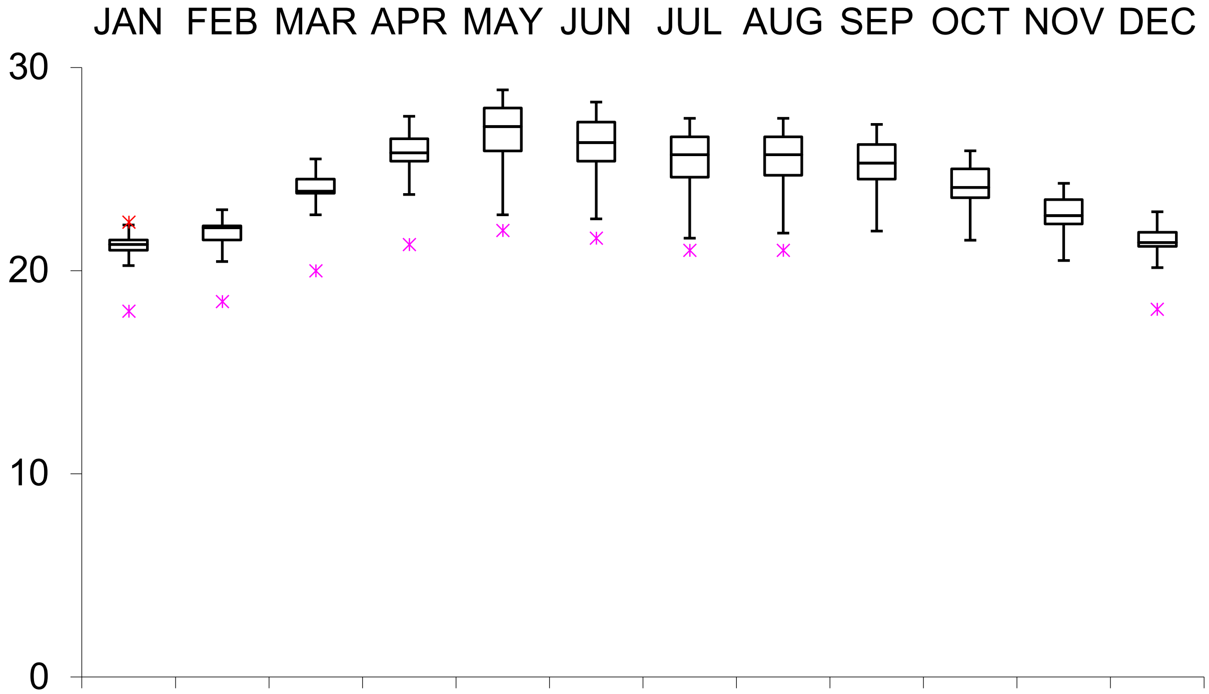

4.13.3.2. MinMT [°C]

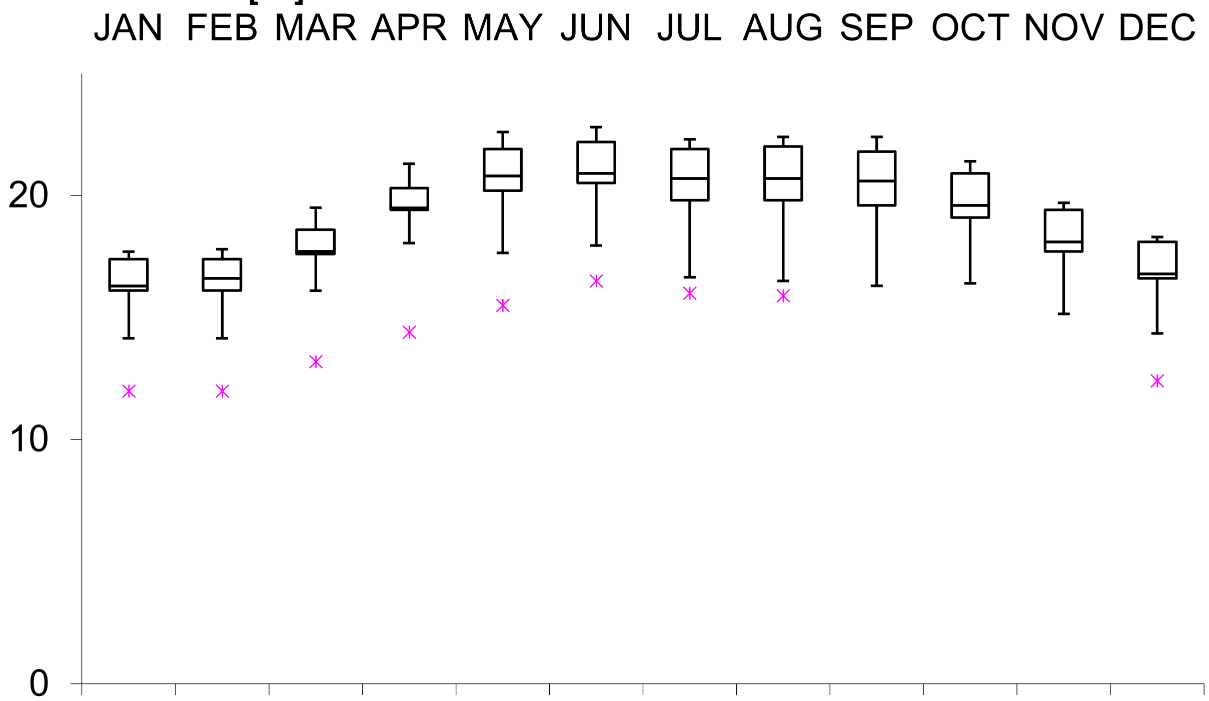

4.13.3.3. MMP [mm]

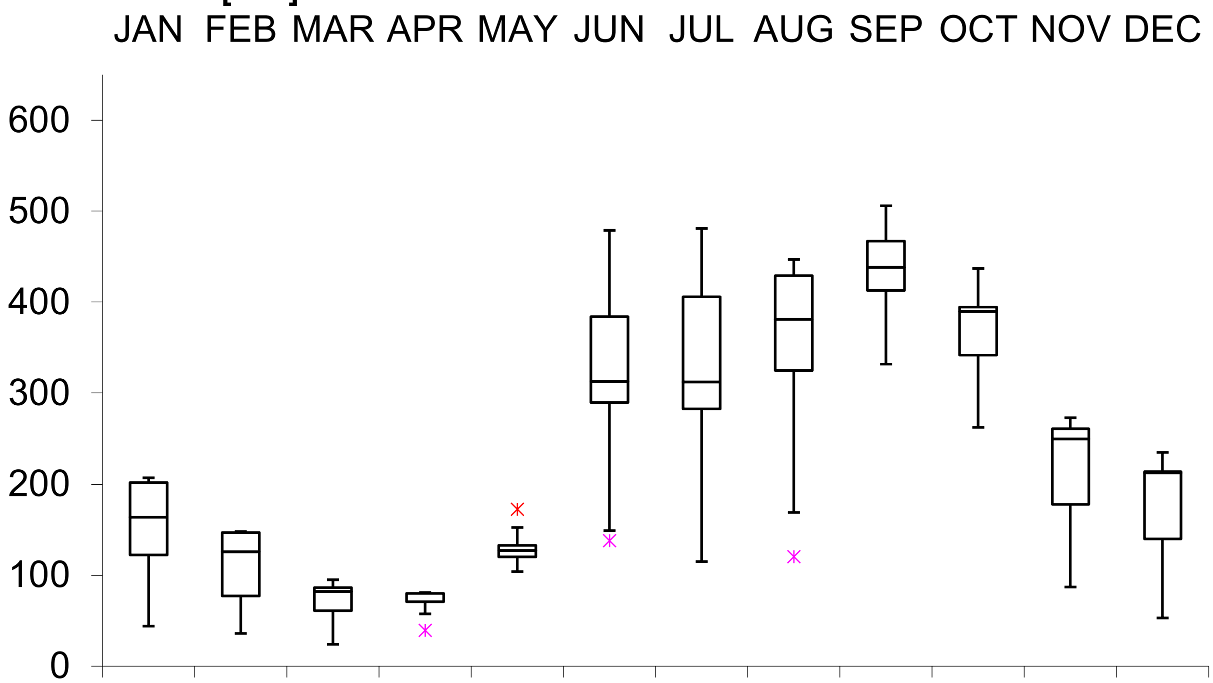

JAN FEB MAR APR MAY JUN JUL AUG SEP OCT NOV DEC
600
500
400
300
200
100
0

4.14. Species *Mortoniodendron sulcatum* Al.Rodr., 2004

4.14.1. Köppen profile, distribution, and climate map – GBIF occurrences of *Mortoniodendron sulcatum*; herbarium specimens (n = 23).

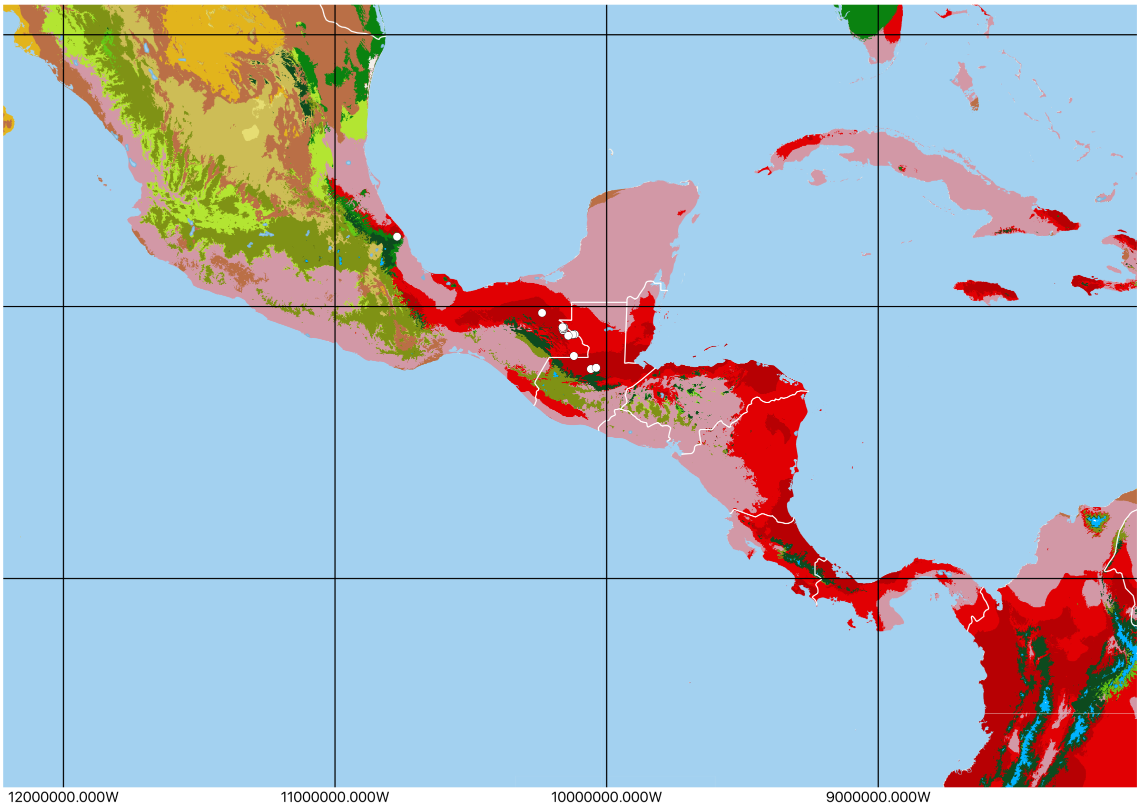

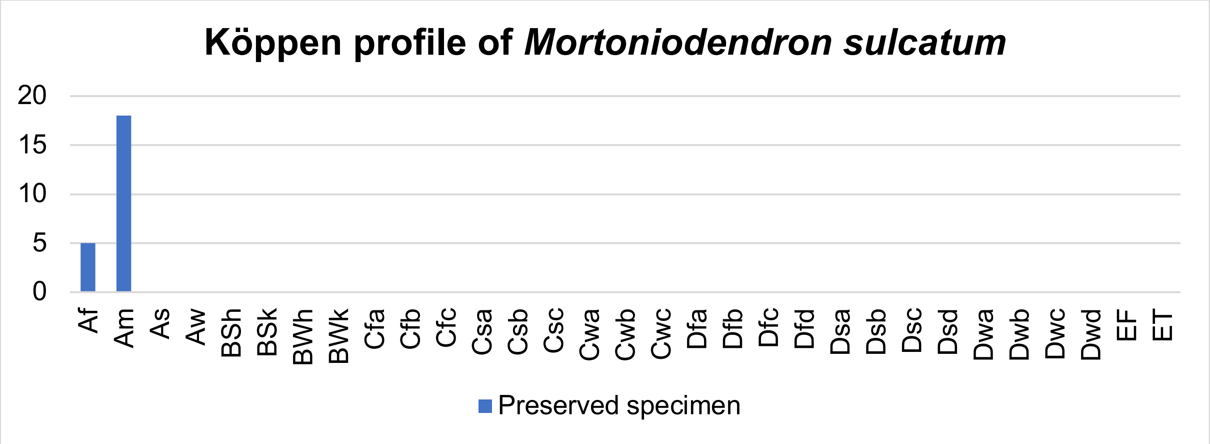

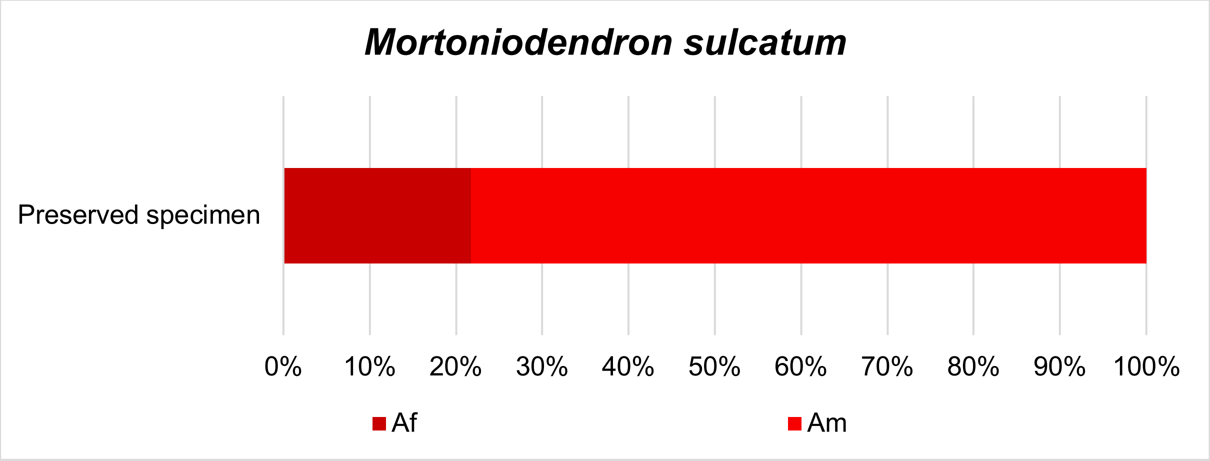

4.14.2. Biome profile, distribution, and biome map – GBIF occurrences of *Mortoniodendron sulcatum*; herbarium specimens (n = 23).

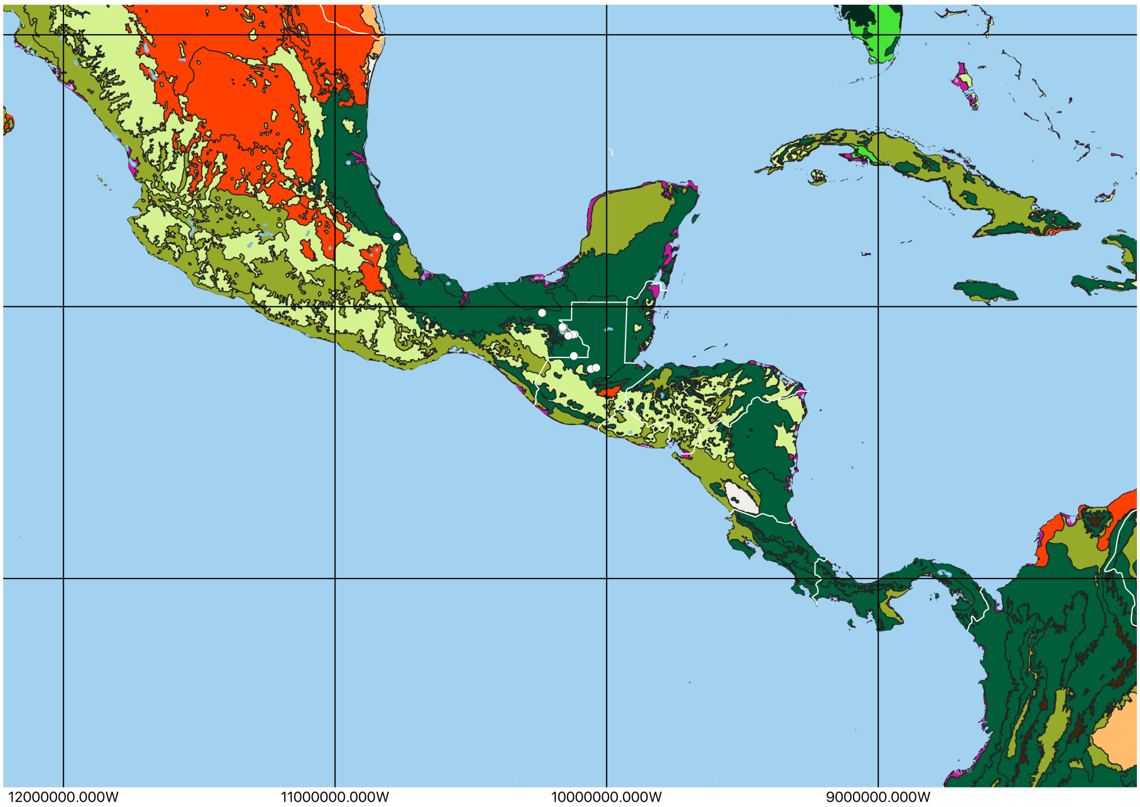

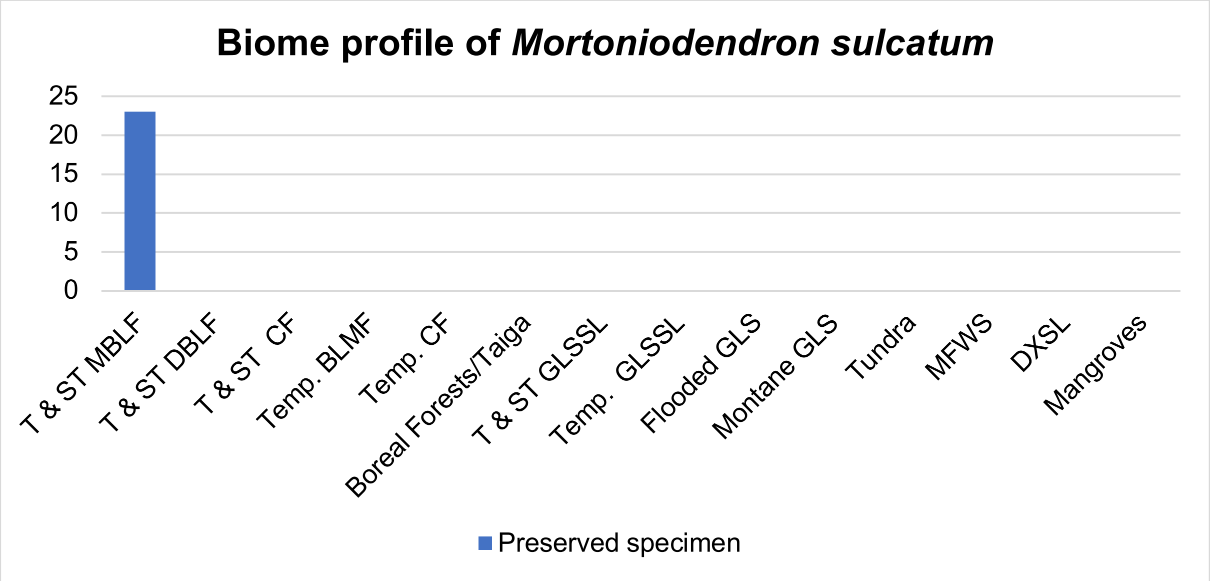

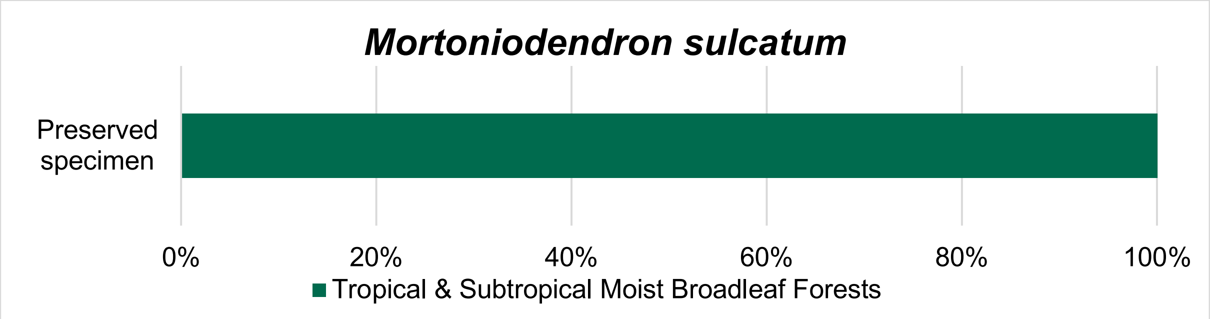

4.14.3. Climate graphs - based on 23 *Mortoniodendron sulcatum* occurrences in GBIF

4.14.3.1. MMT [°C]

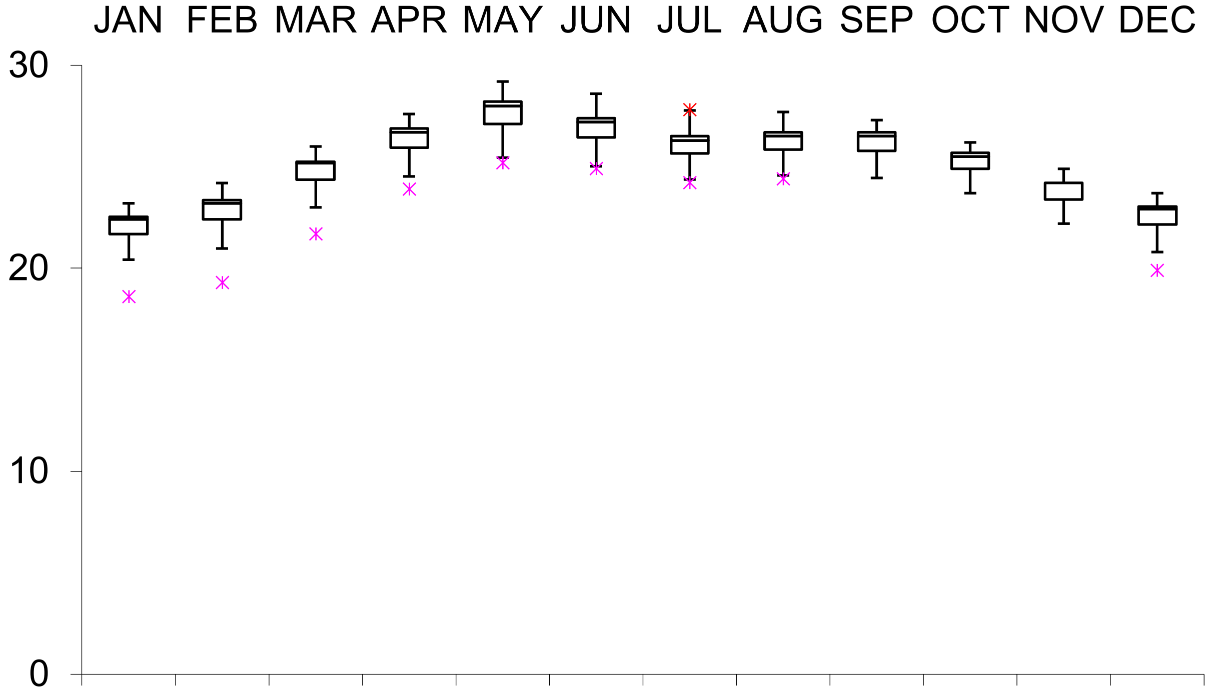

4.14.3.2. MinMT [°C]

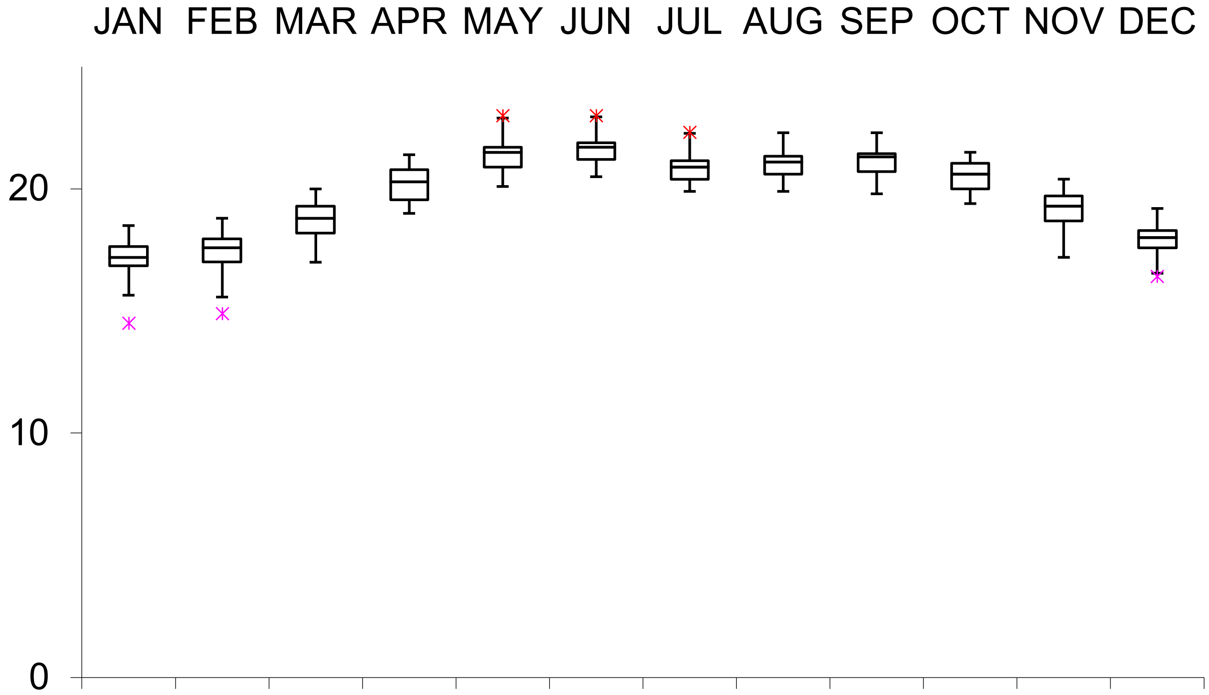

4.14.3.3. MMP [mm]

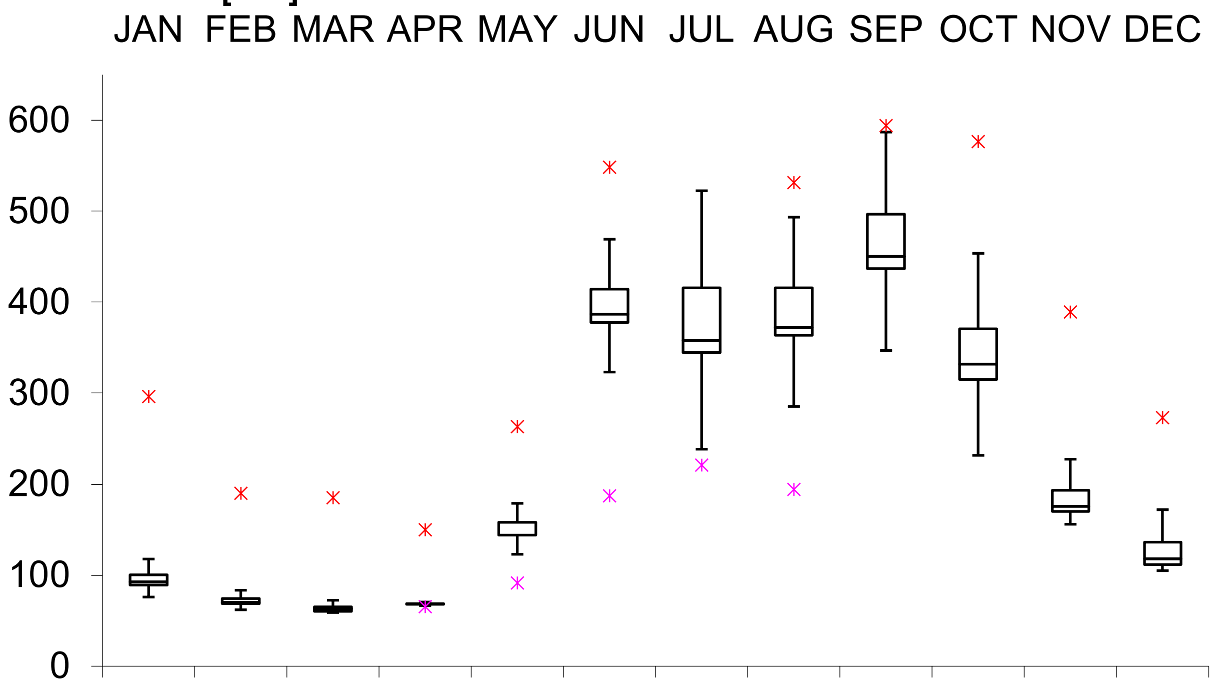
JAN
FEB
MAR
APR
MAY
JUN
JUL
AUG
SEP
OCT
NOV
DEC
600
500
400
300
200
100
0

4.15. Species *Mortoniodendron uxpanapense* Al.Rodr., 2004

4.15.1. Köppen profile, distribution, and climate map – GBIF occurrences of *Mortoniodendron uxpanapense*; herbarium specimens (n = 4).

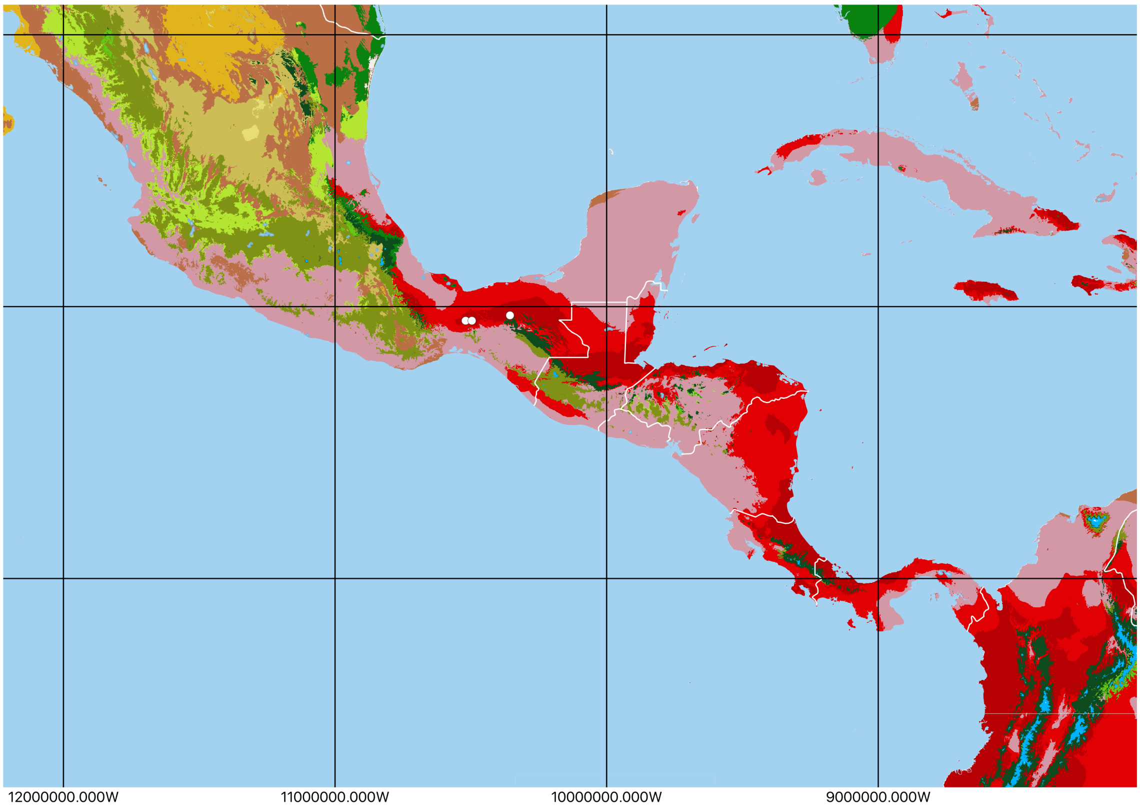

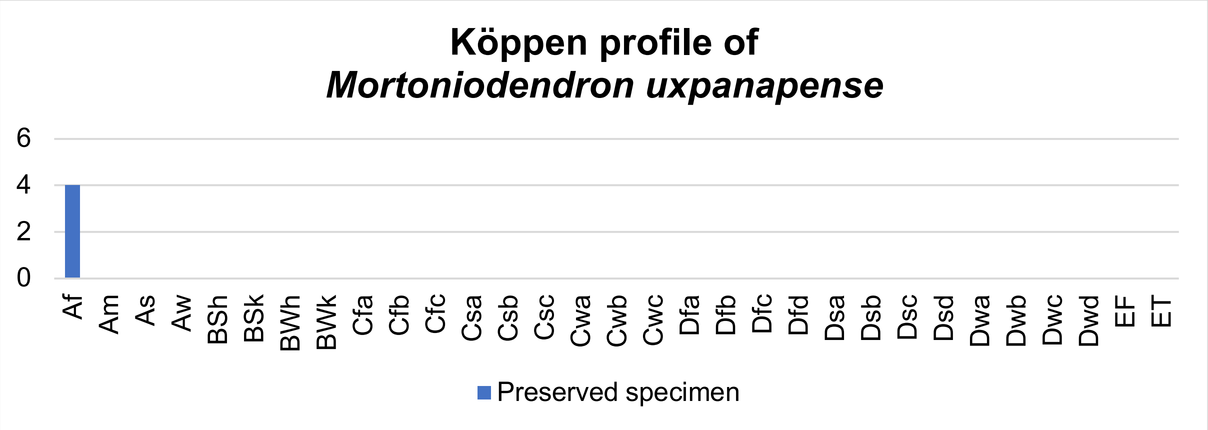

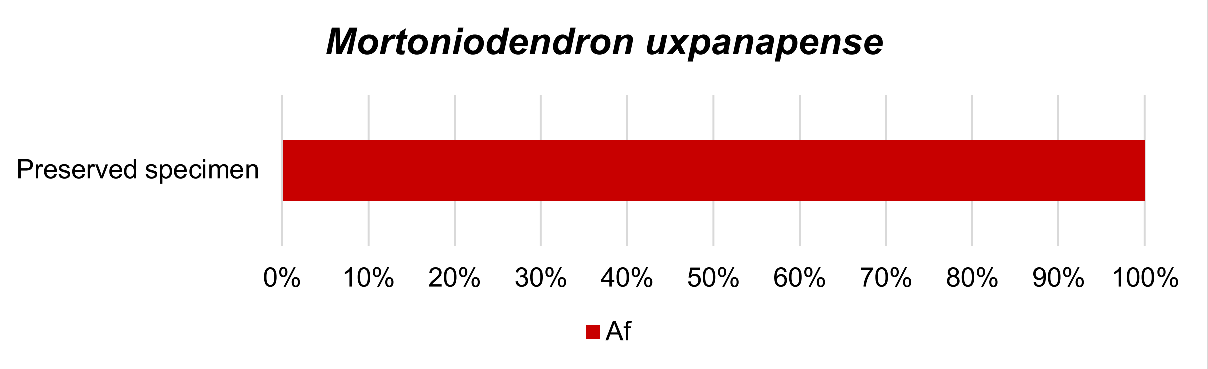

4.15.2. Biome profile, distribution, and biome map – GBIF occurrences of *Mortoniodendron uxpanapense*; herbarium specimens (n = 4).

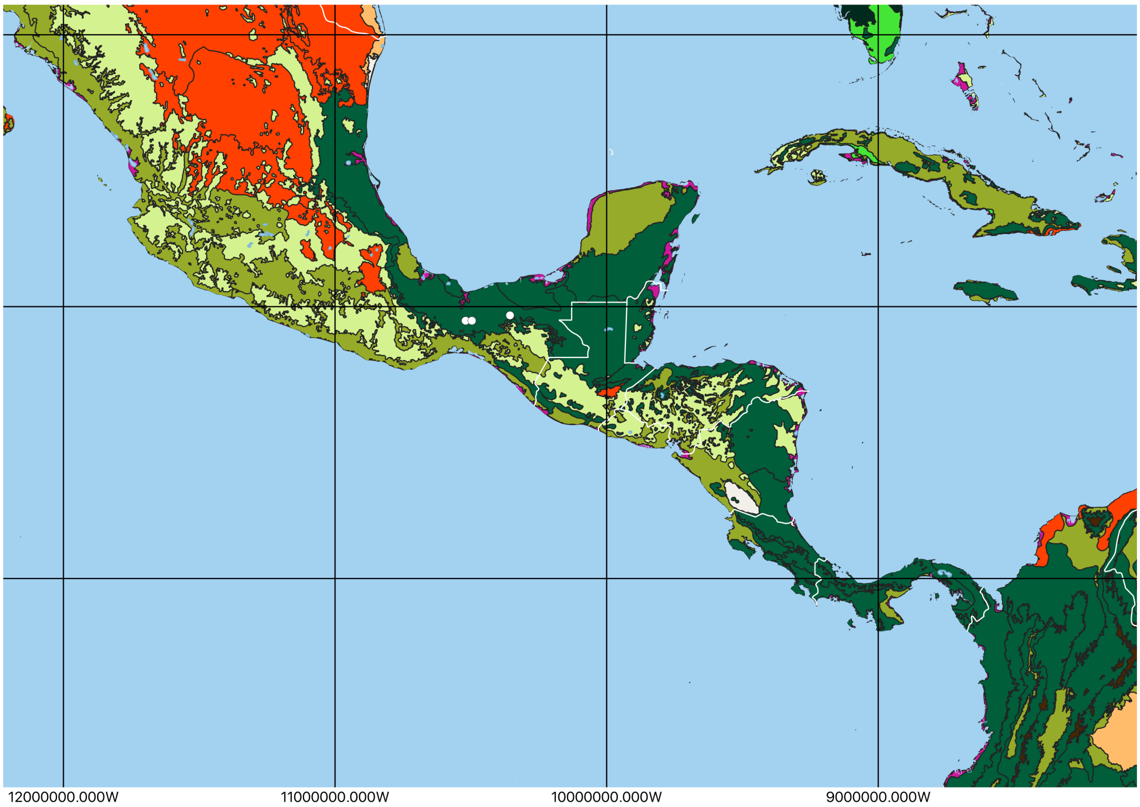

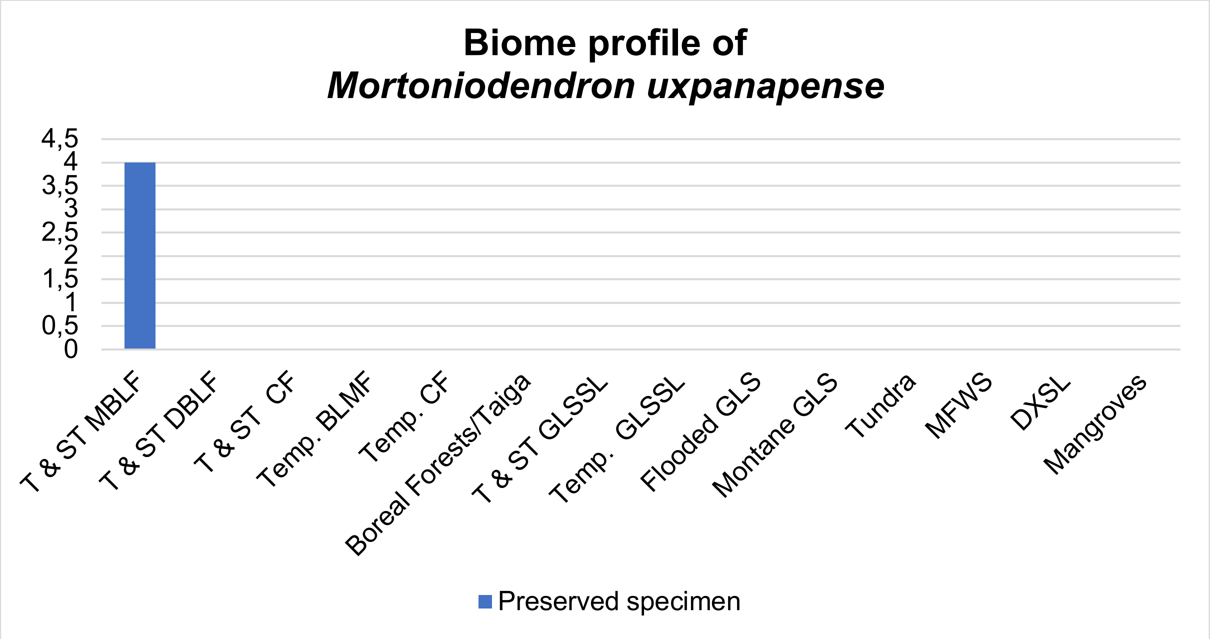

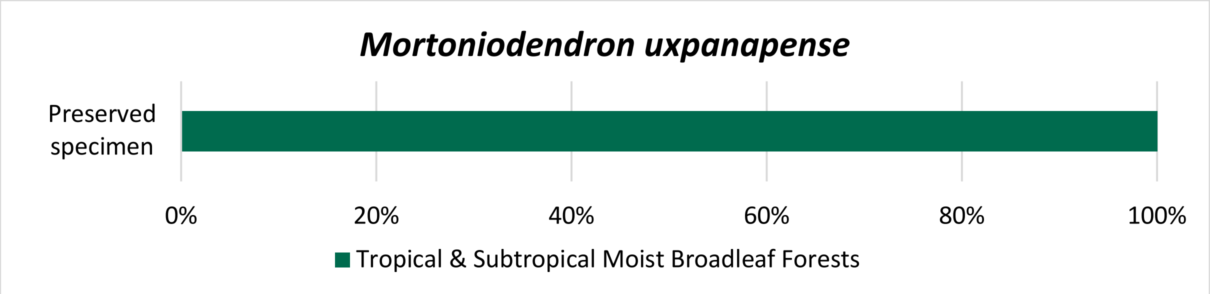

4.15.3. Climate graphs - based on 4 *Mortoniodendron uxpanapense* occurrences in GBIF

4.15.3.1. MMT [°C]

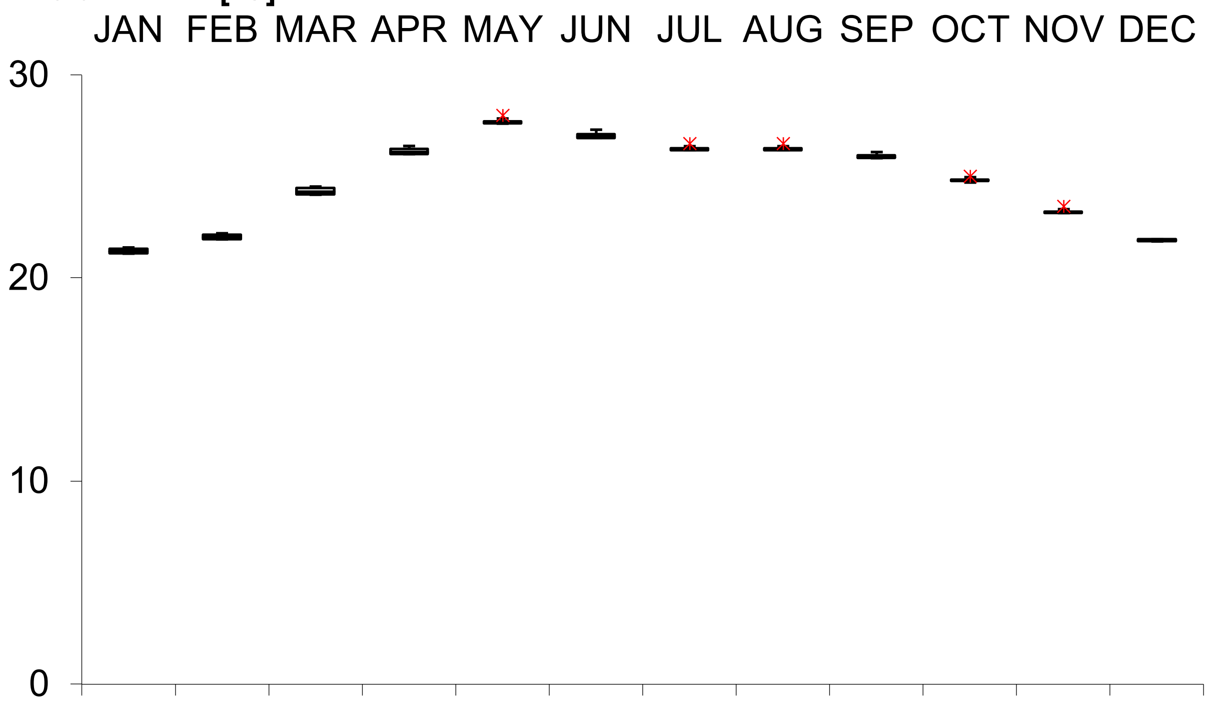

4.15.3.2. MinMT [°C]

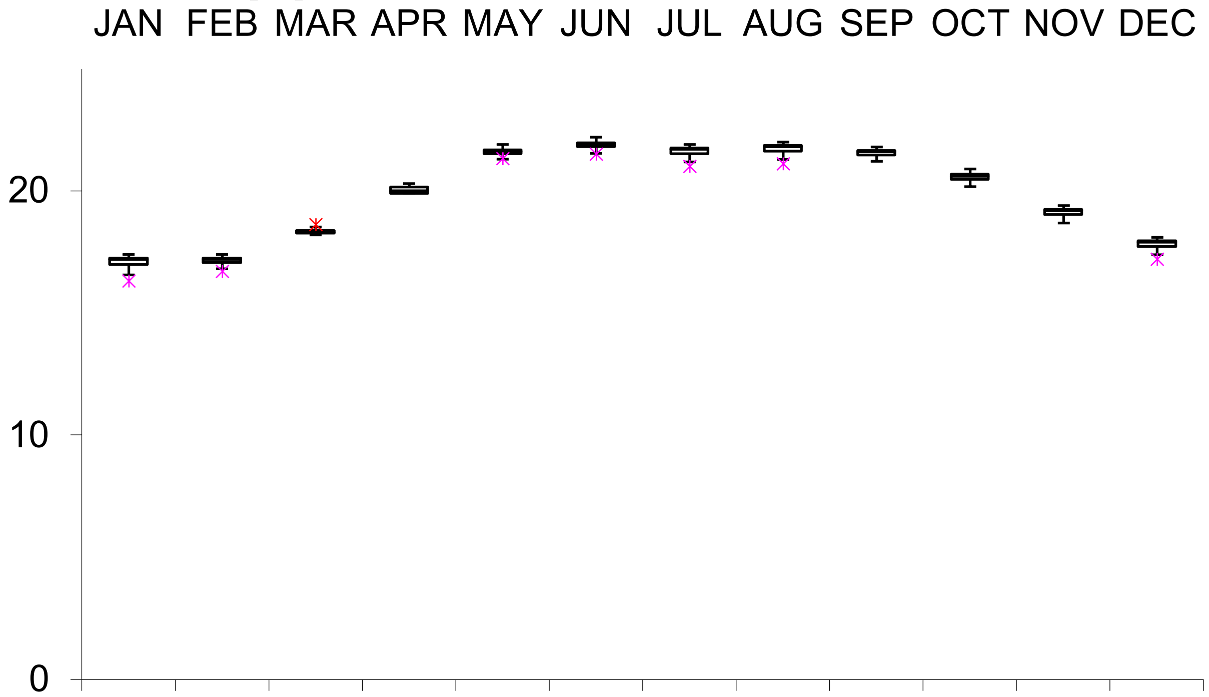

4.15.3.3. MMP [mm]

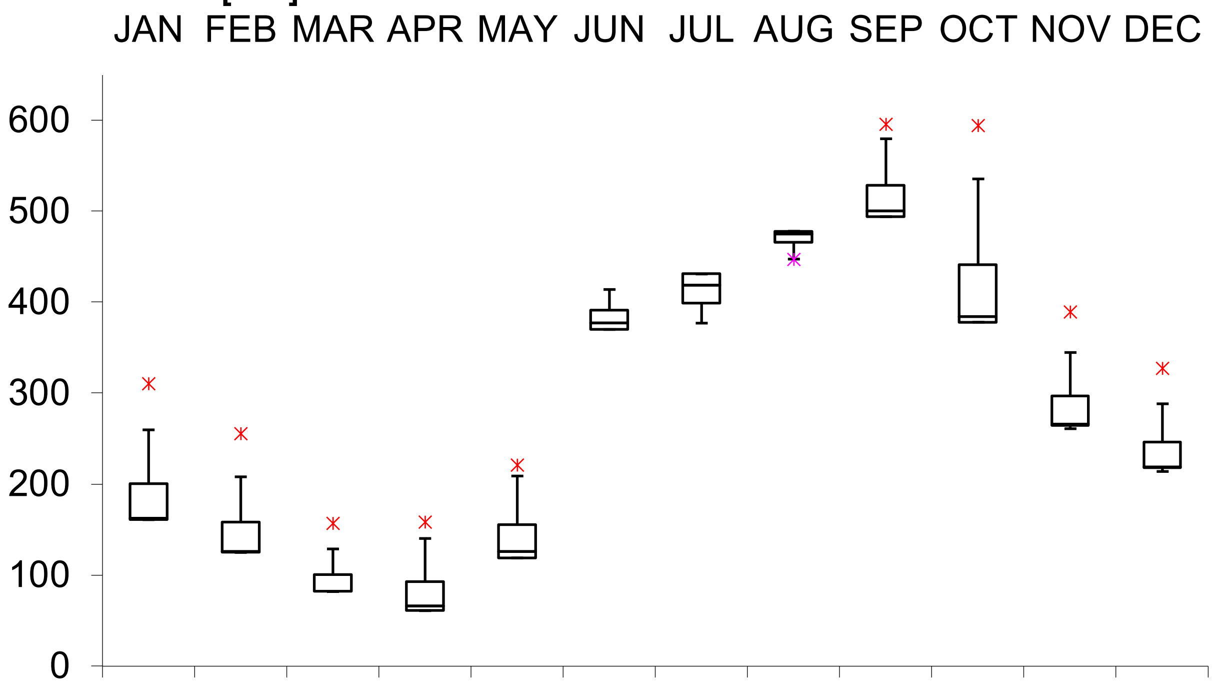
JAN FEB MAR APR MAY JUN JUL AUG SEP OCT NOV DEC
600
500
400
300
200
100
0

4.16. Species *Mortoniodendron vestitum* Al.Rodr., 2004

4.16.1. Köppen profile, distribution, and climate map – GBIF occurrences of *Mortoniodendron vestitum*; herbarium specimens (n = 92).

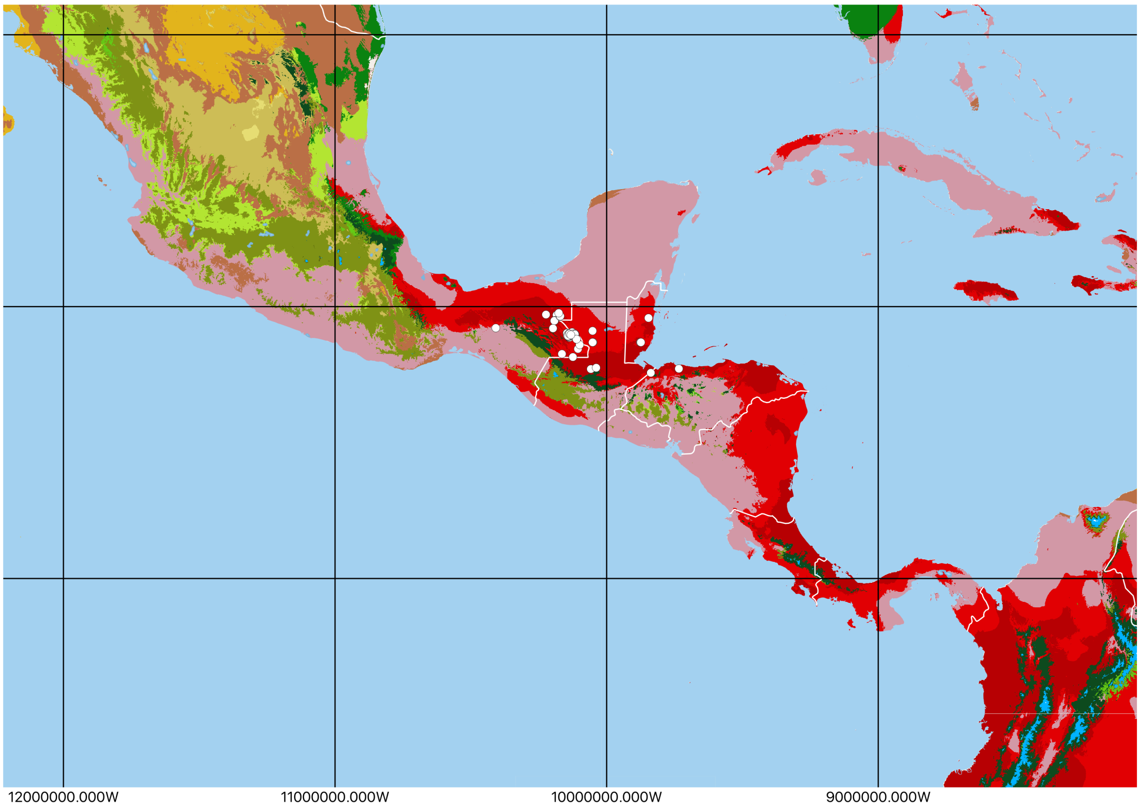

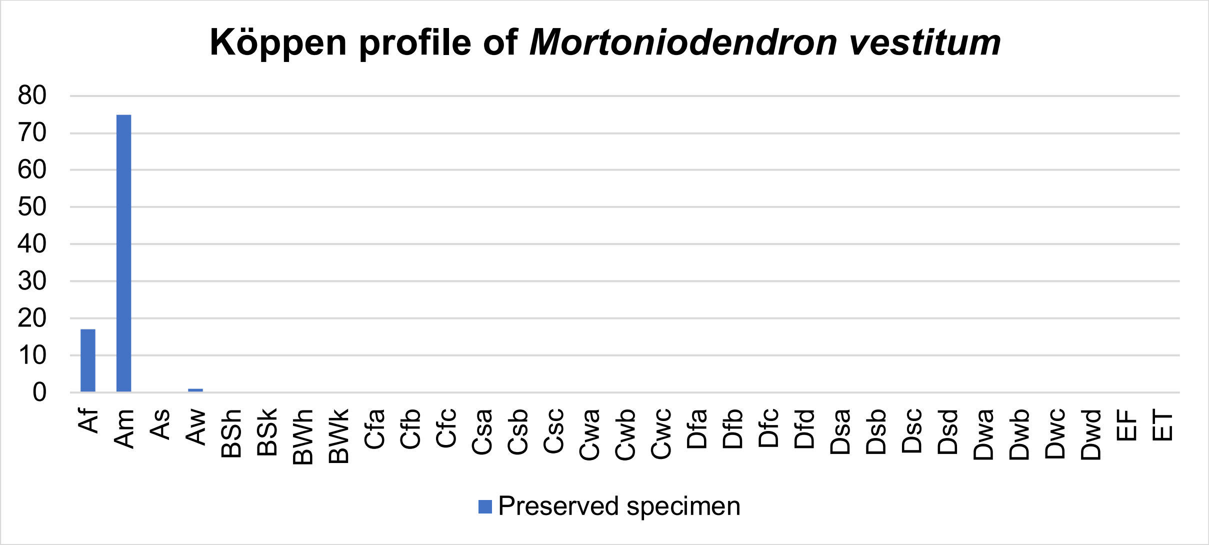

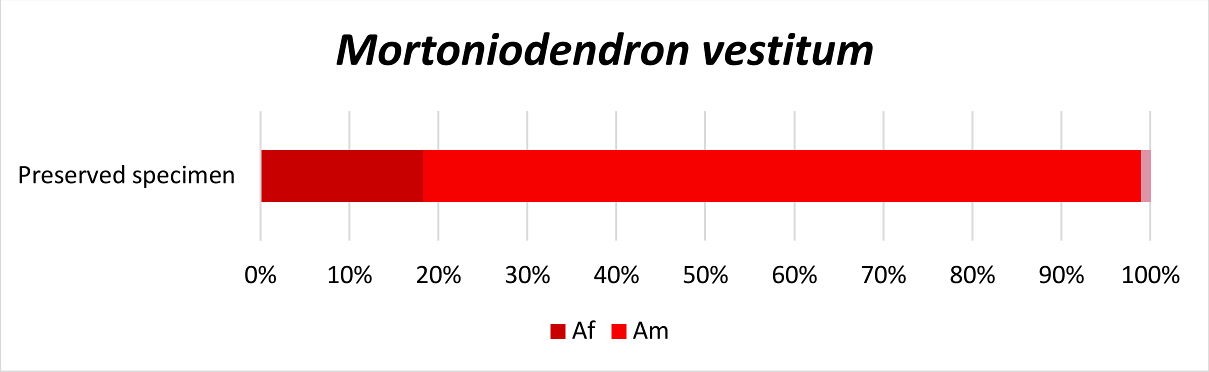

4.16.2. Biome profile, distribution, and biome map – GBIF occurrences of *Mortoniodendron vestitum*; herbarium specimens (n = 92).

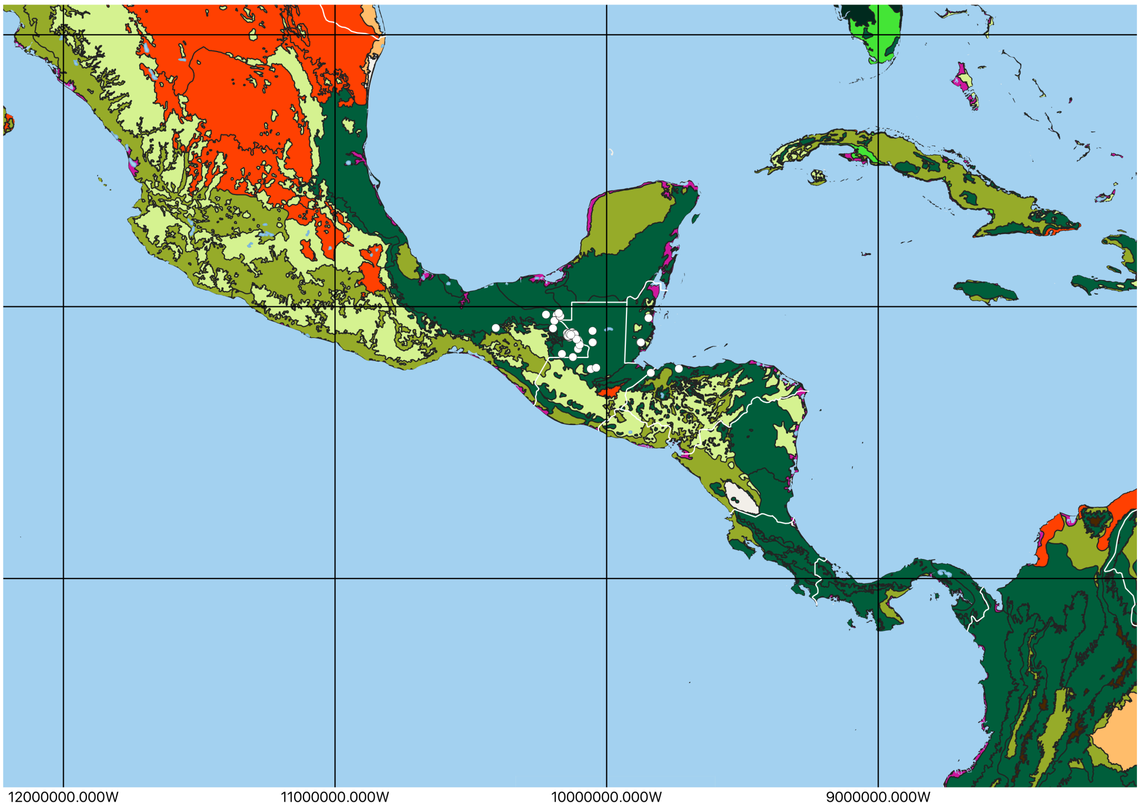

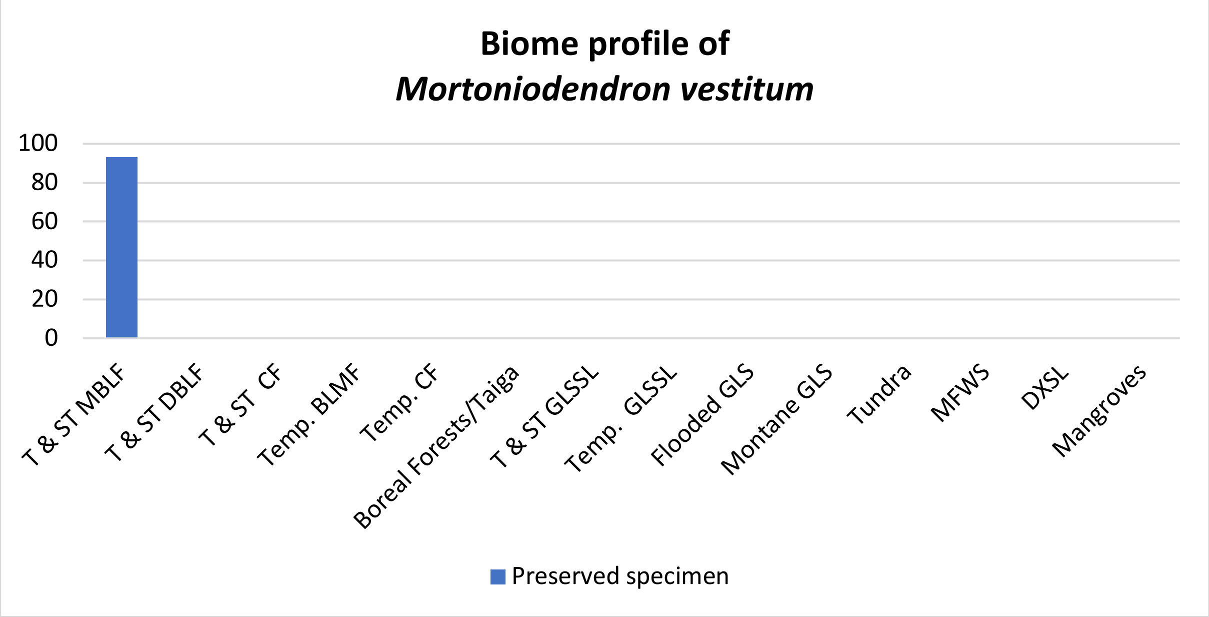

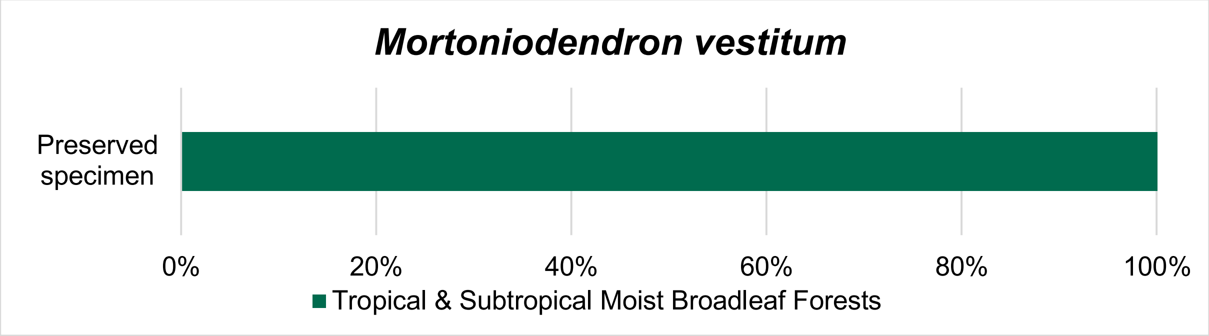

4.16.3. Climate graphs - based on 92 *Mortoniodendron vestitum* occurrences in GBIF

4.16.3.1. MMT [°C]

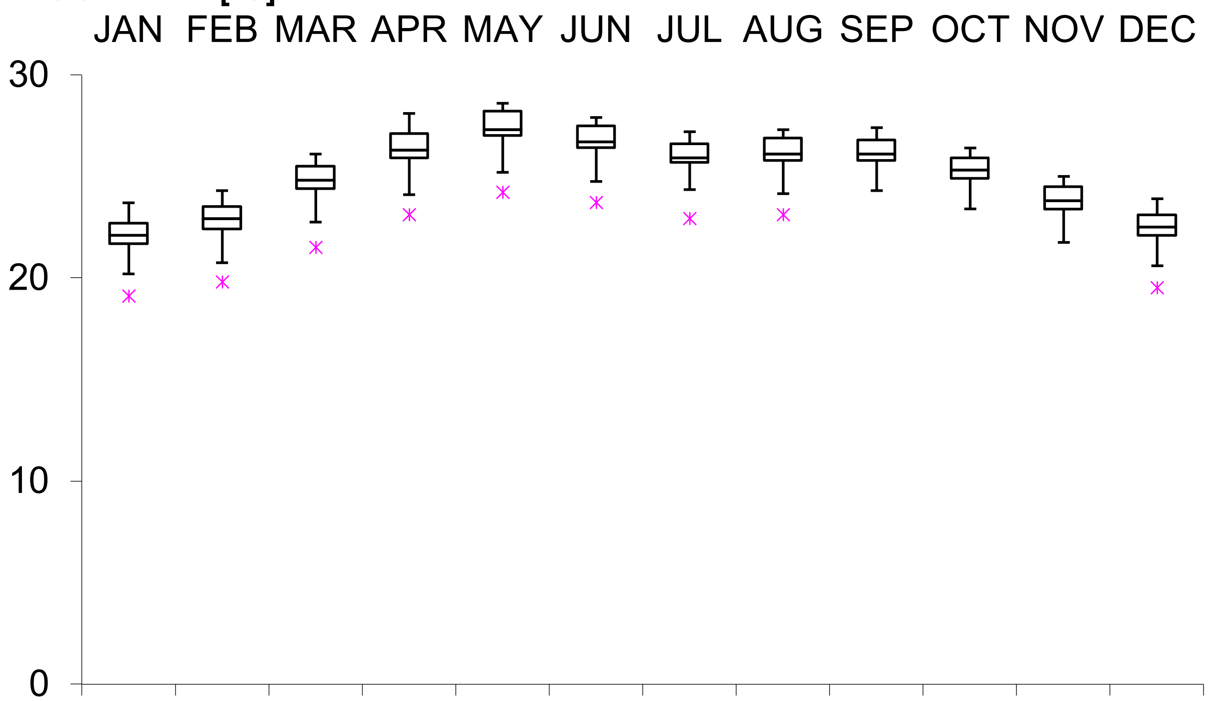

4.16.3.2. MinMT [°C]

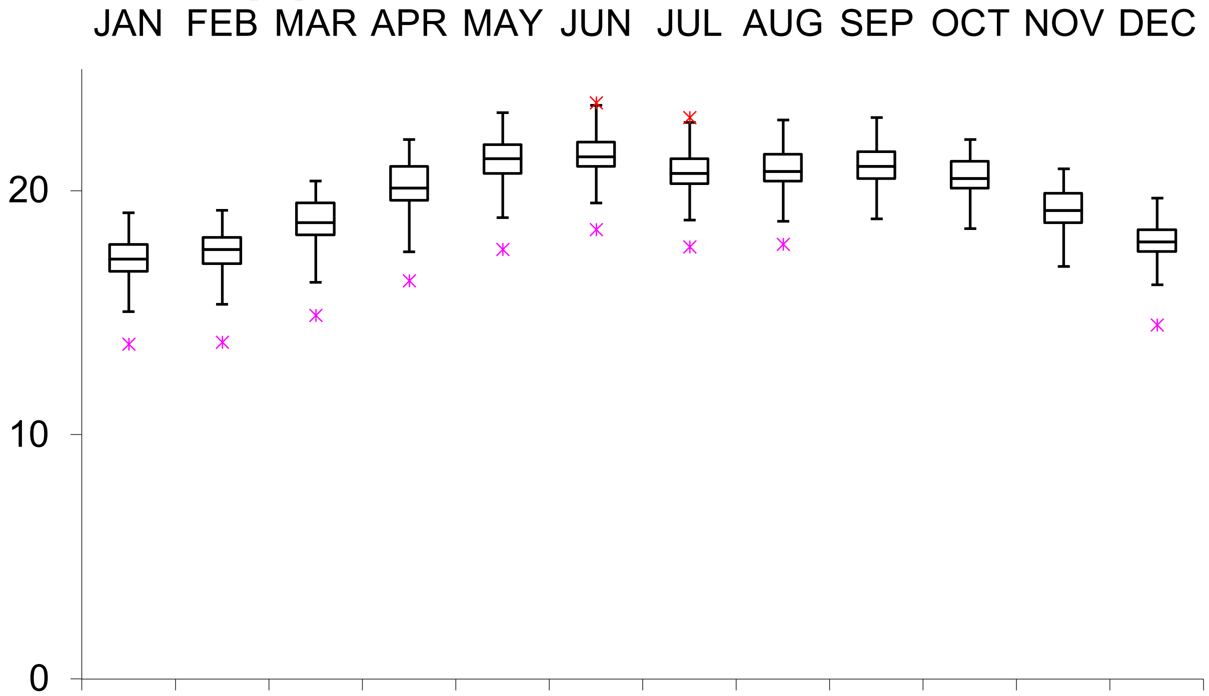

4.16.3.3. MMP [mm]

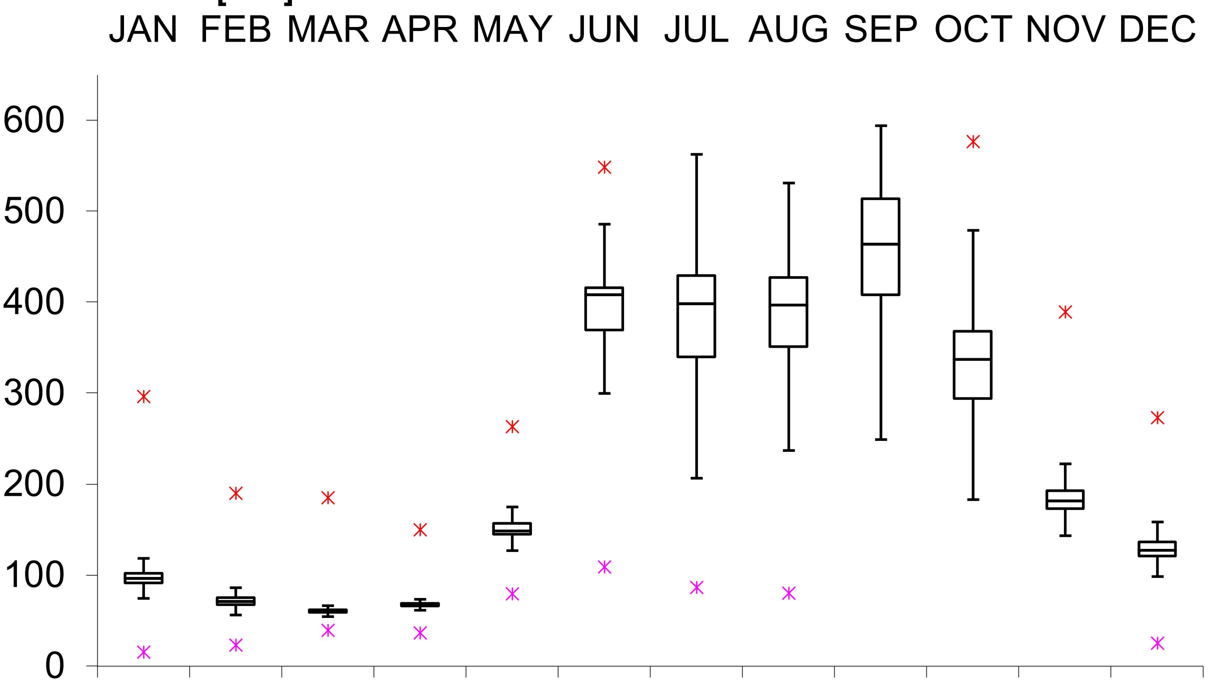

JAN FEB MAR APR MAY JUN JUL AUG SEP OCT NOV DEC
600
500
400
300
200
100
0

5. ***Tilia* L., 1753 (systematics, mainly following Pigott, 2011)**

Köppen profiles

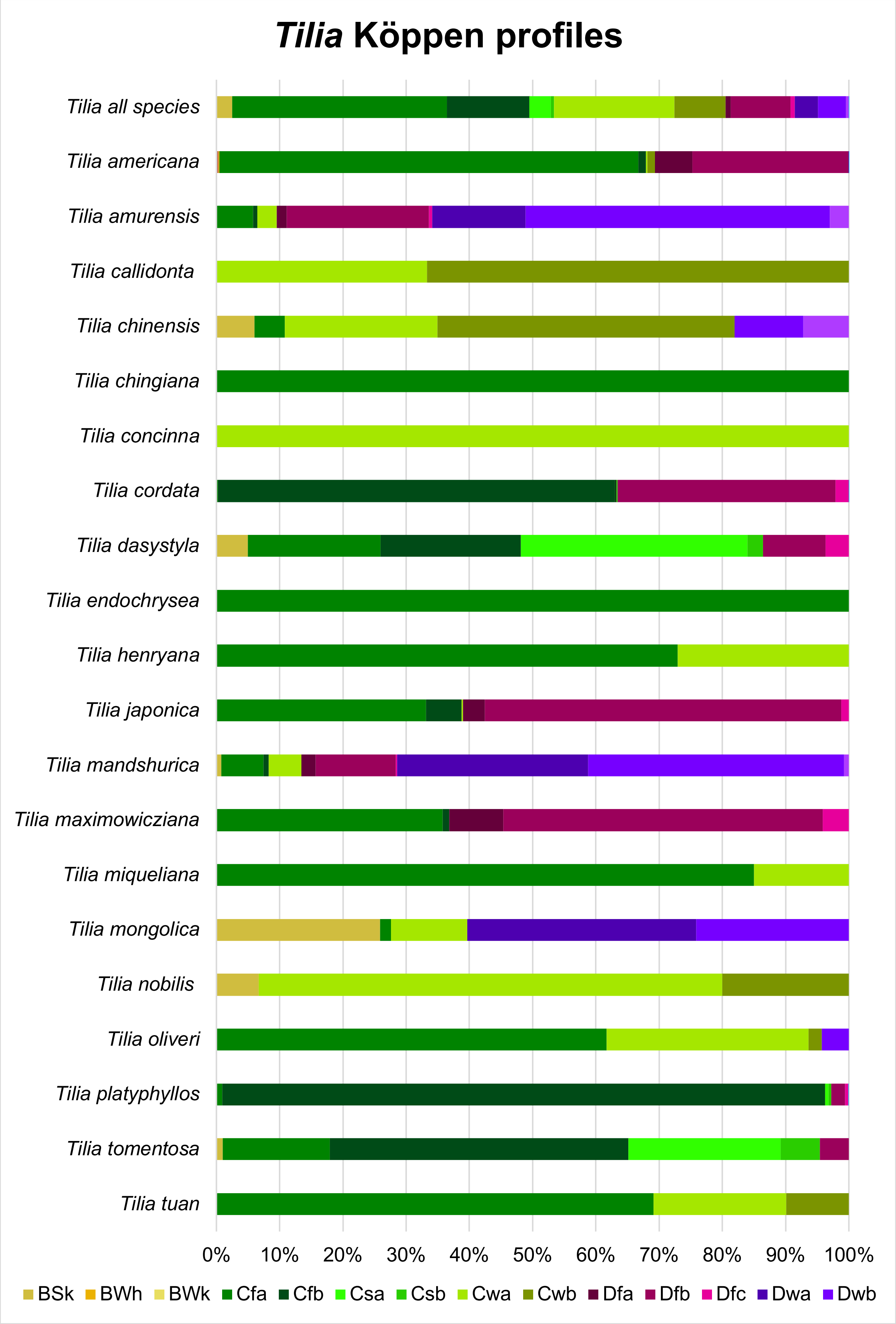

Biome profiles

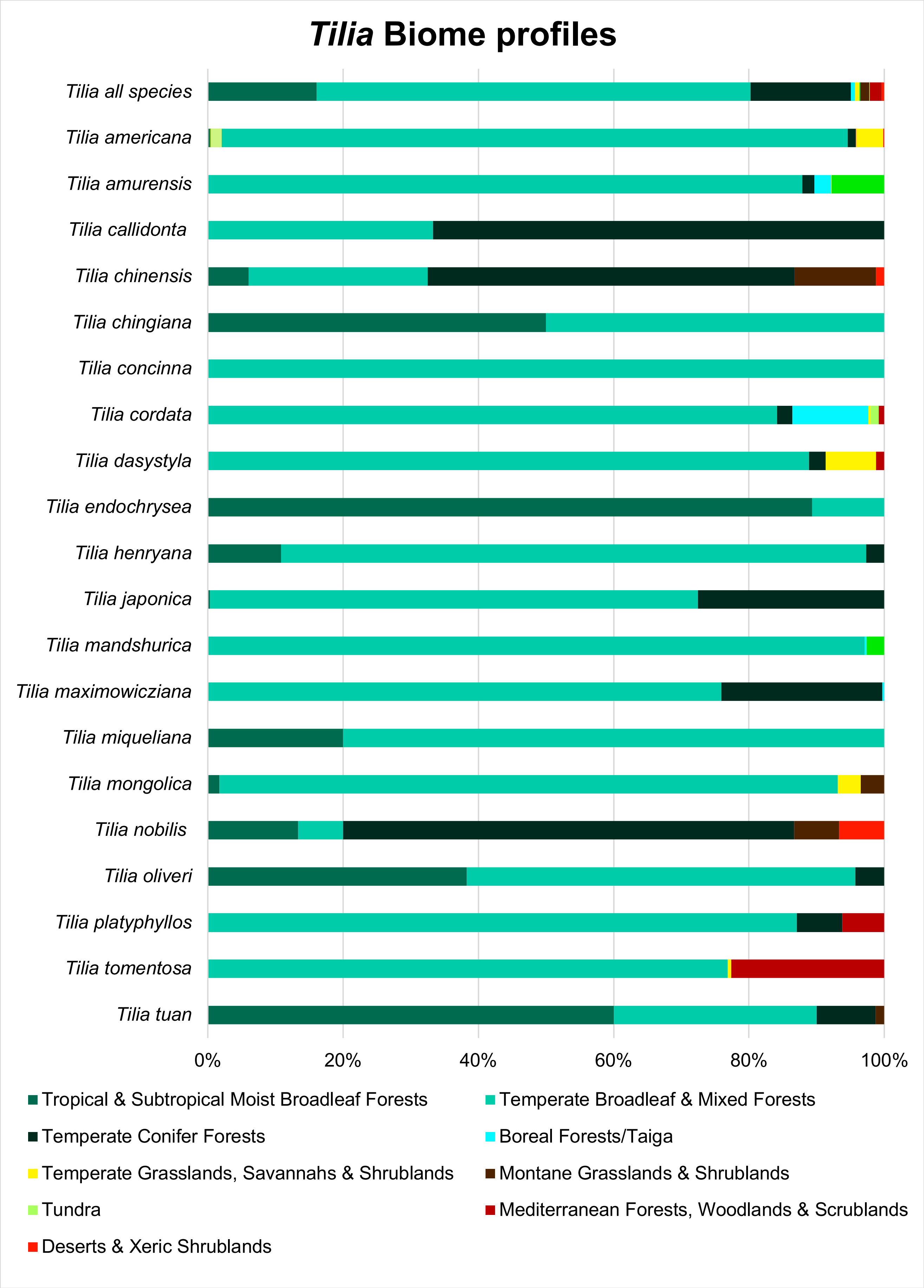

5.1. European and western Asian Tilia taxa

European and western Asian *Tilia* distribution map Köppen climate types

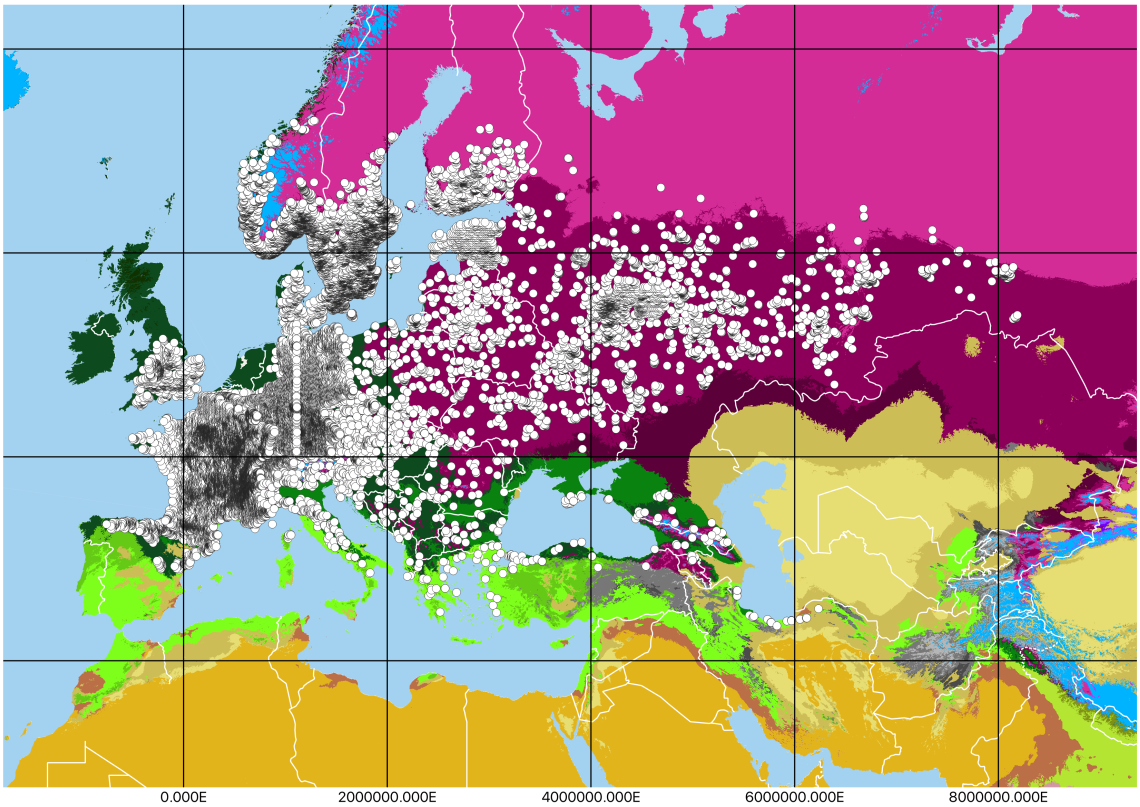

European and western Asian *Tilia* distribution map biomes

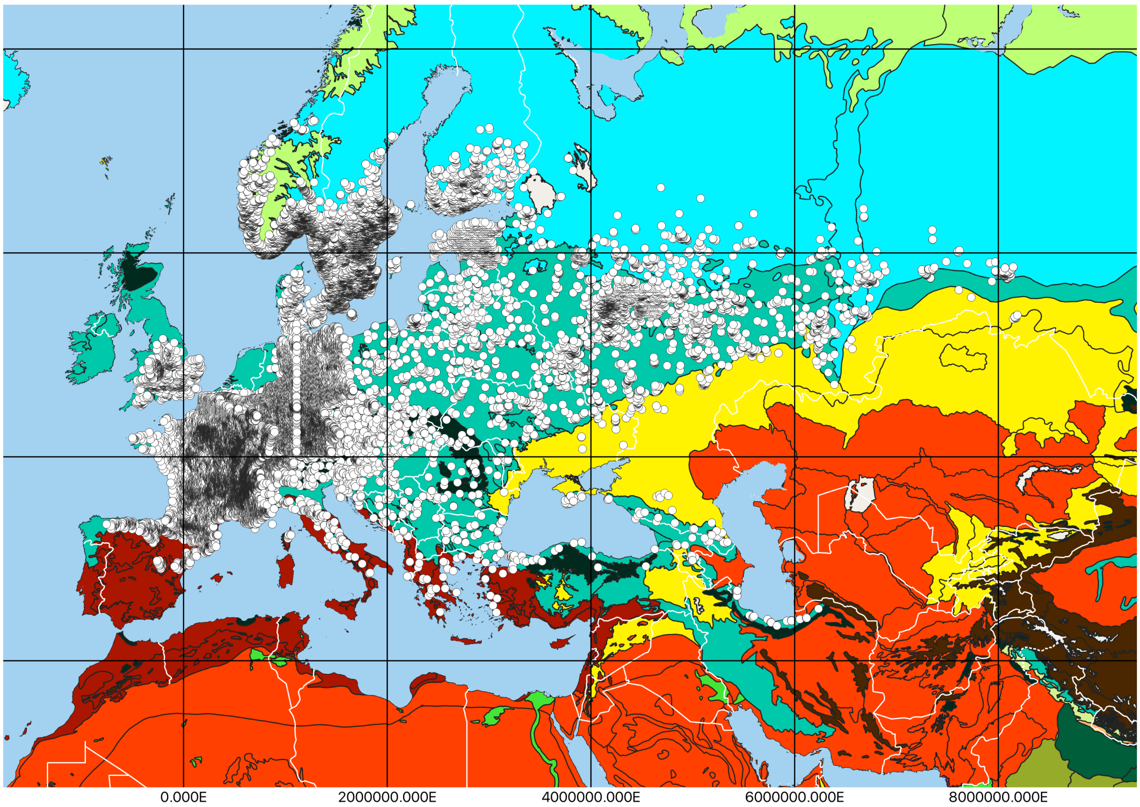

5.1.1. Species *Tilia cordata* Mill., 1768

5.1.1.1 Köppen profile, distribution, and climate map – GBIF occurrences of *Tilia cordata*; excluding duplicate occurrences (n = 43183), herbarium specimens (n = 346).

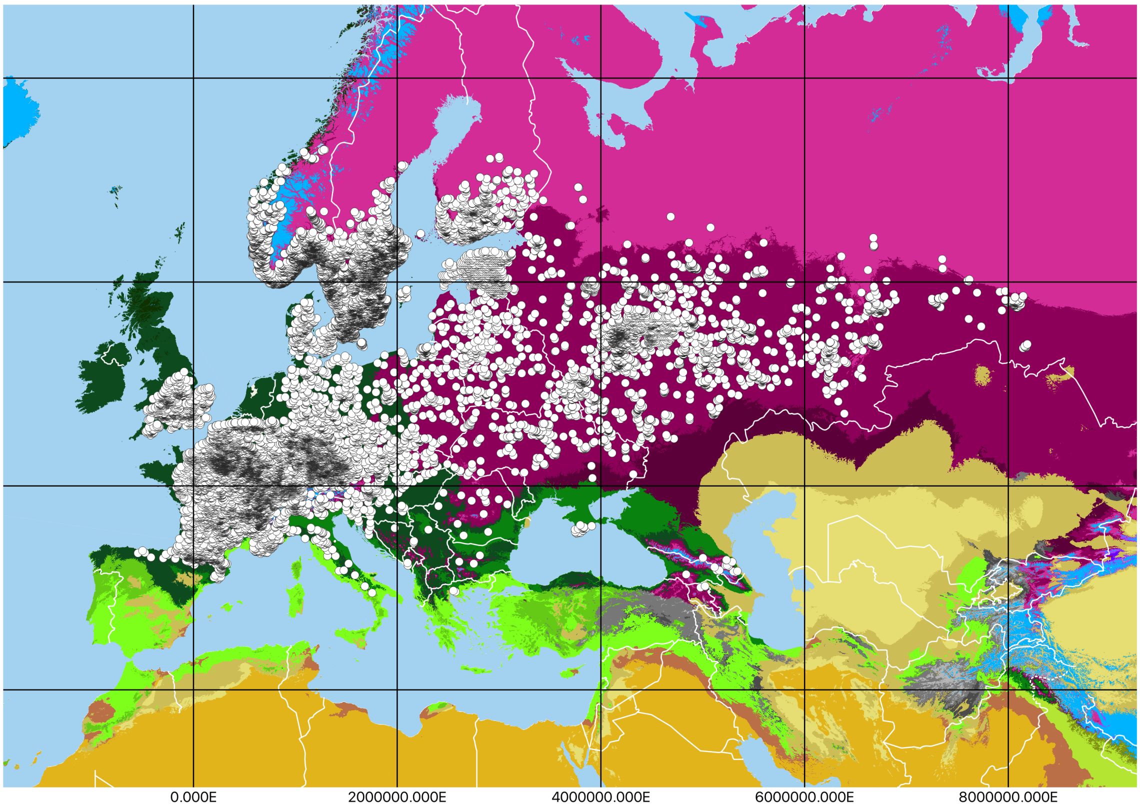

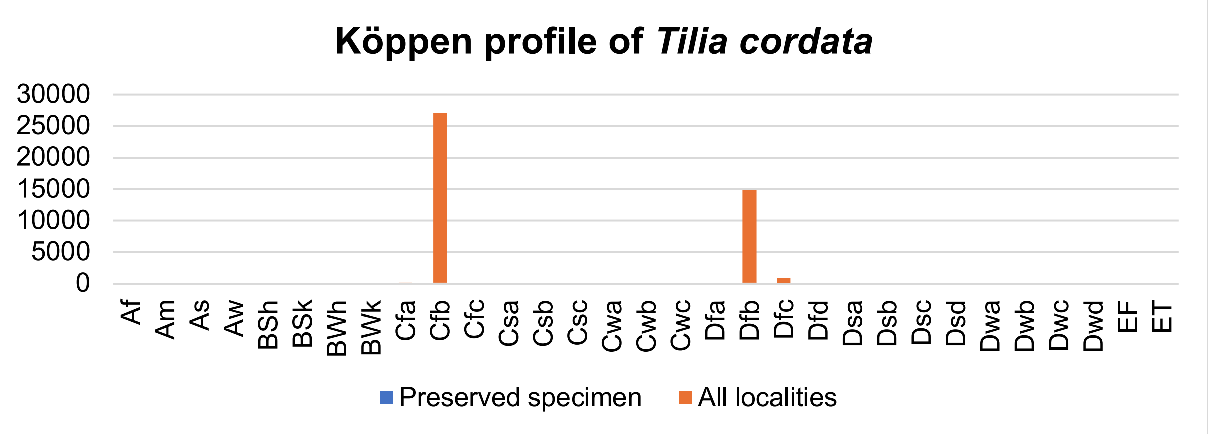

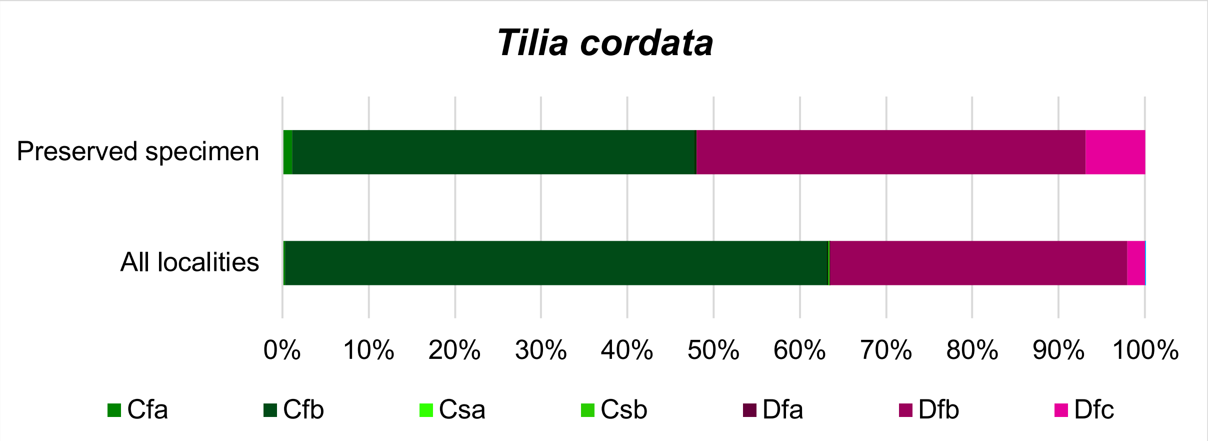

5.1.1.2. Biome profile, distribution, and biome map – GBIF occurrences of *Tilia cordata*; excluding duplicate occurrences (n = 43183), herbarium specimens (n = 346).

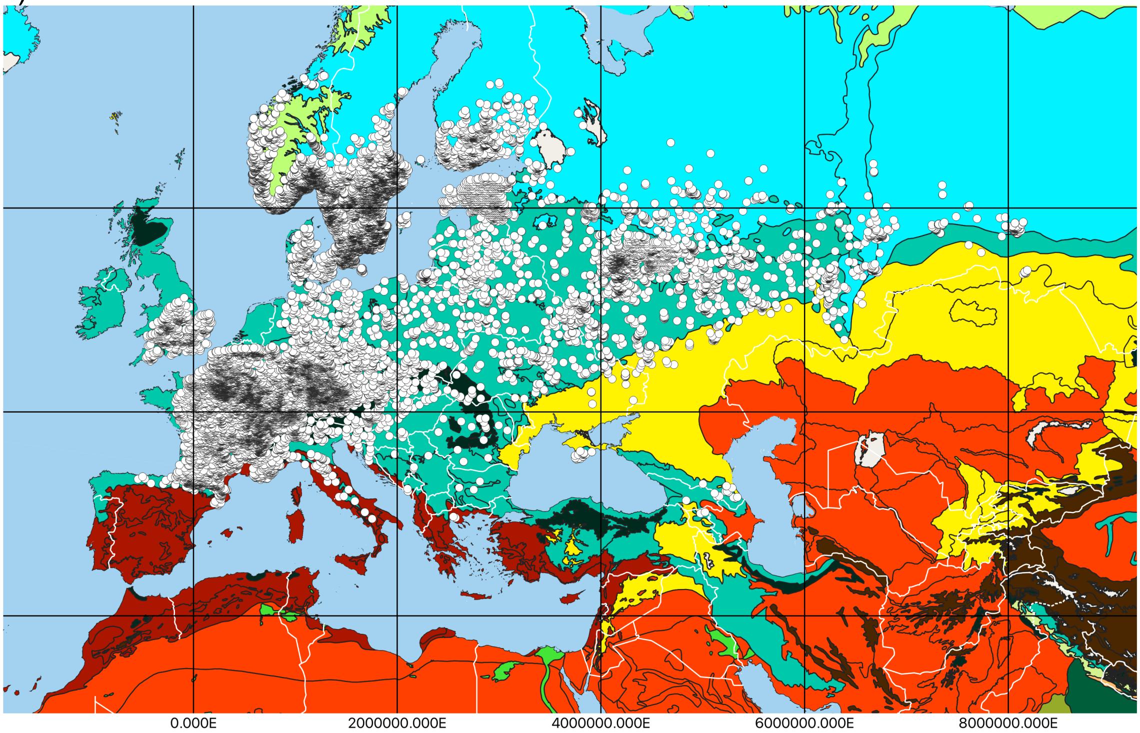

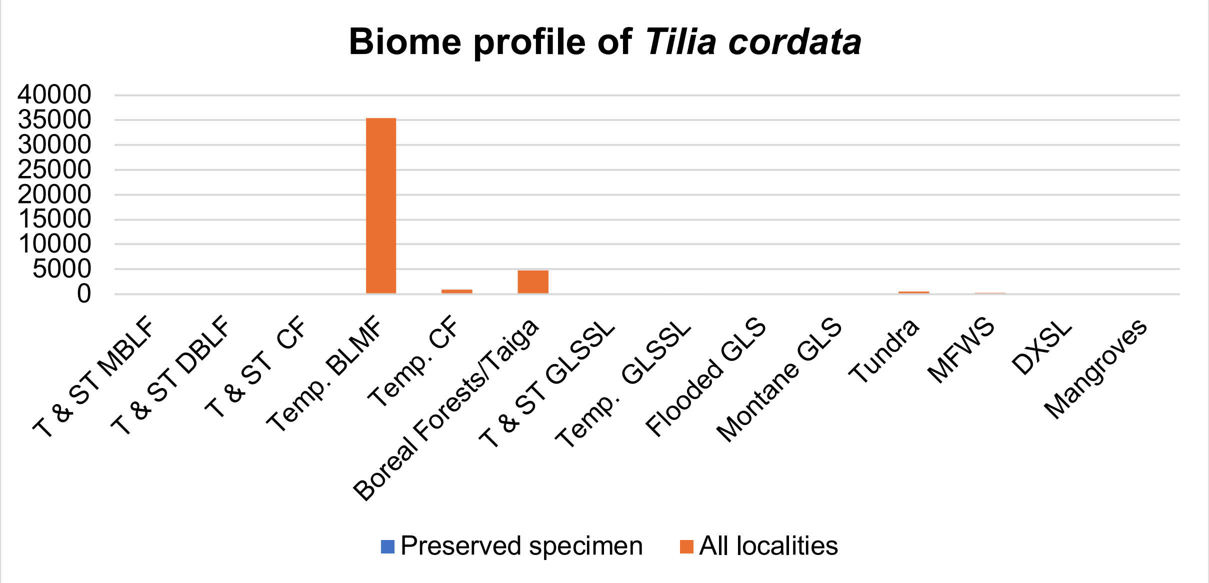

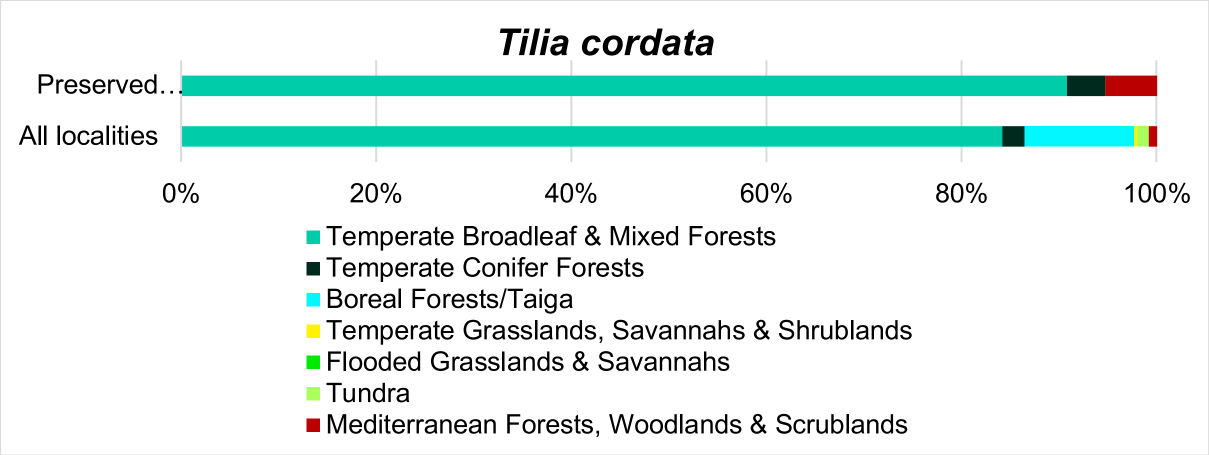

5.1.1.3. Climate graphs - based on 43183 *Tilia cordata* occurrences in GBIF **5.1.3.1. MMT [°C]**

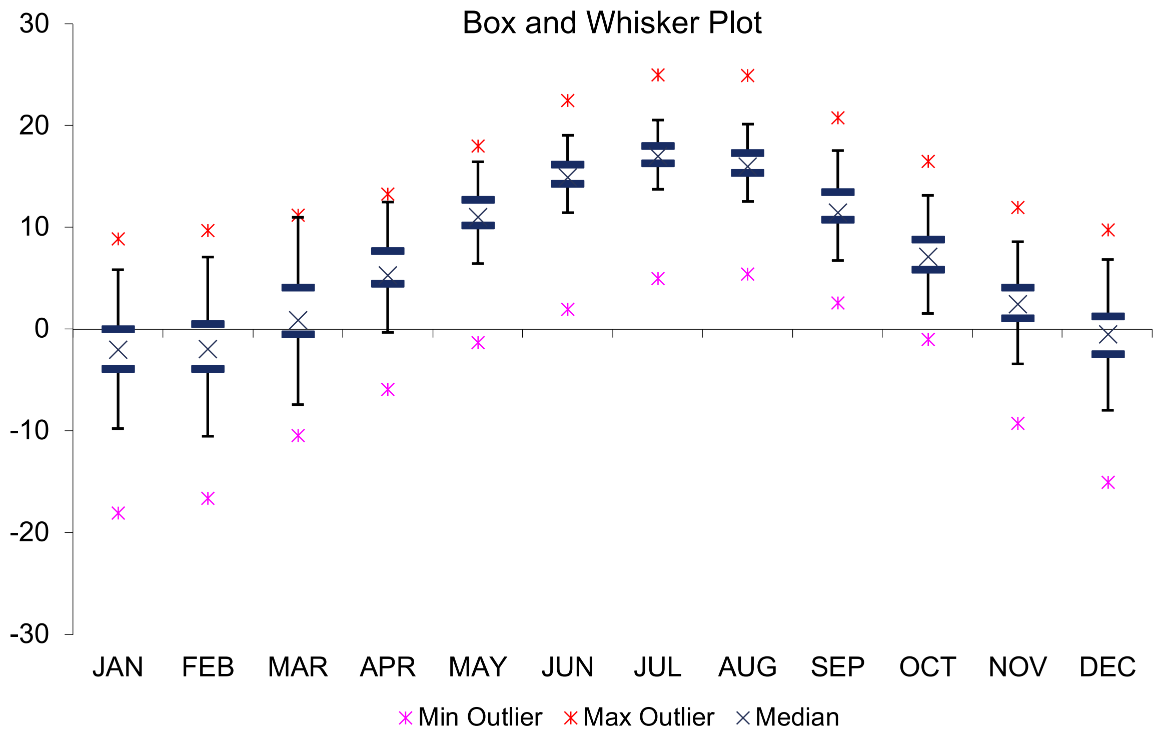

5.1.3.2. MinMT [°C]

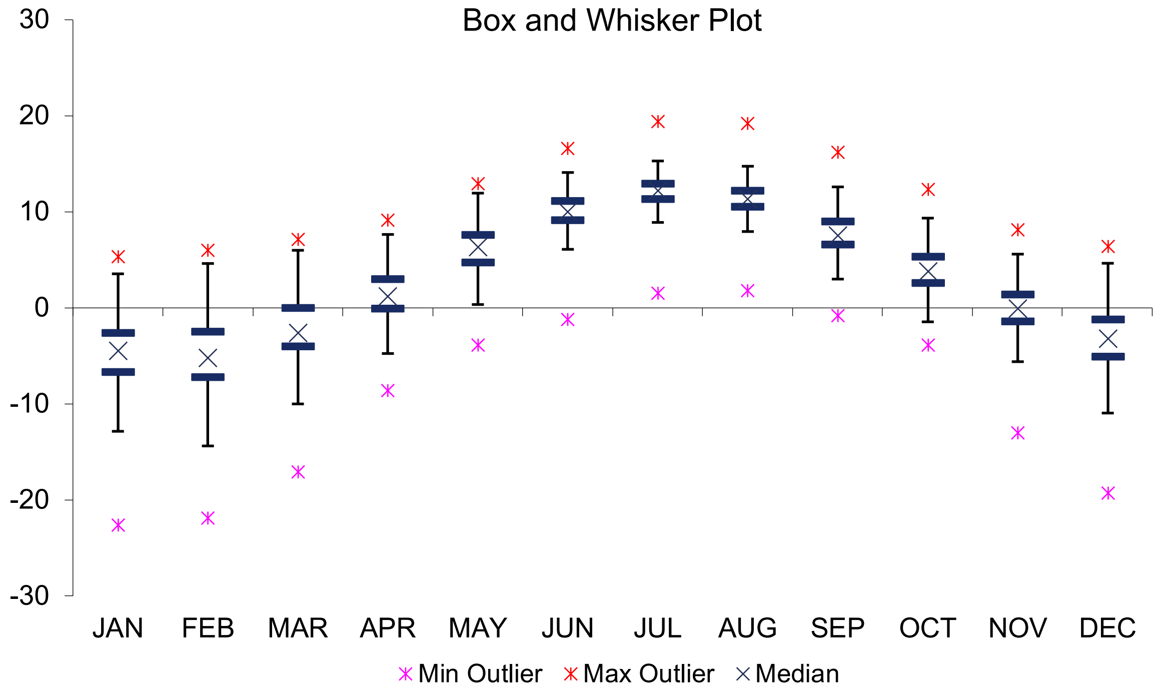

5.1.3.3. MMP [mm]

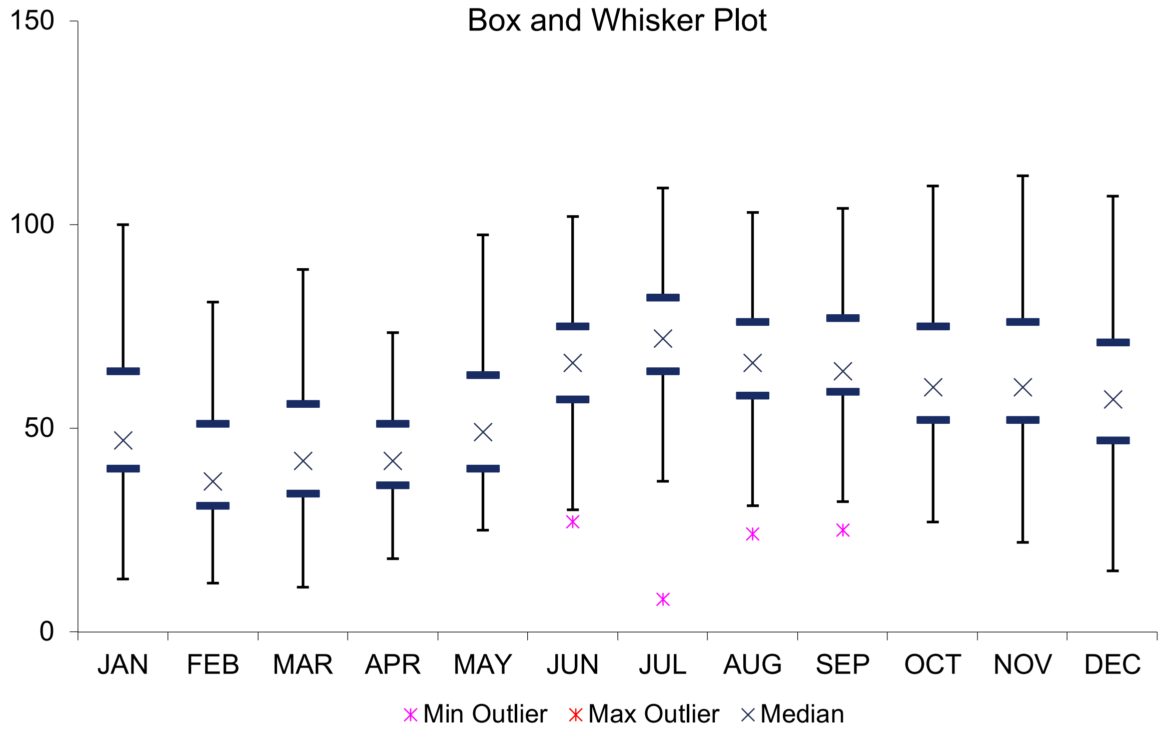

Box and Whisker Plot
150
100
50
0
JAN
FEB
MAR
APR
MAY
JUN
JUL
AUG
SEP
OCT
NOV
DEC
Min Outlier
Max Outlier
Median

5.1.2. Species *Tilia dasystyla* Steven, 1832

5.1.2.1 Köppen profile, distribution, and climate map – GBIF occurrences of *Tilia dasystyla*; excluding duplicate occurrences (n = 81), herbarium specimens (n = 56).

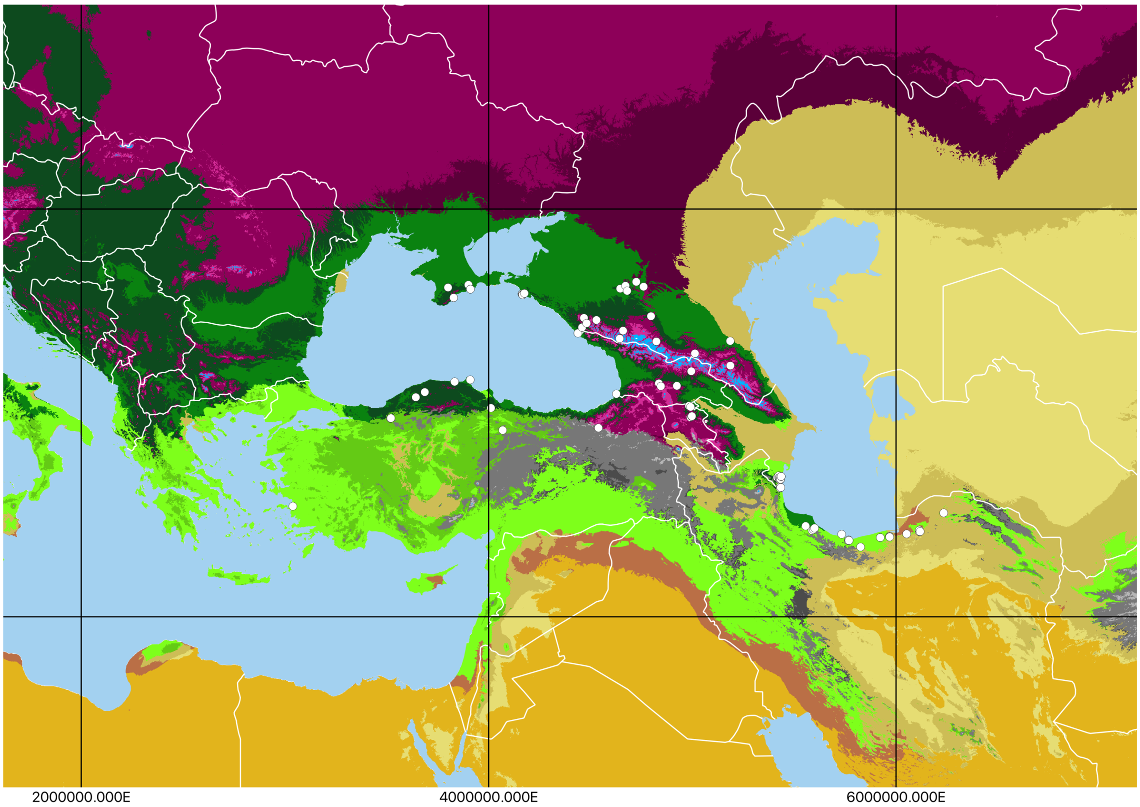

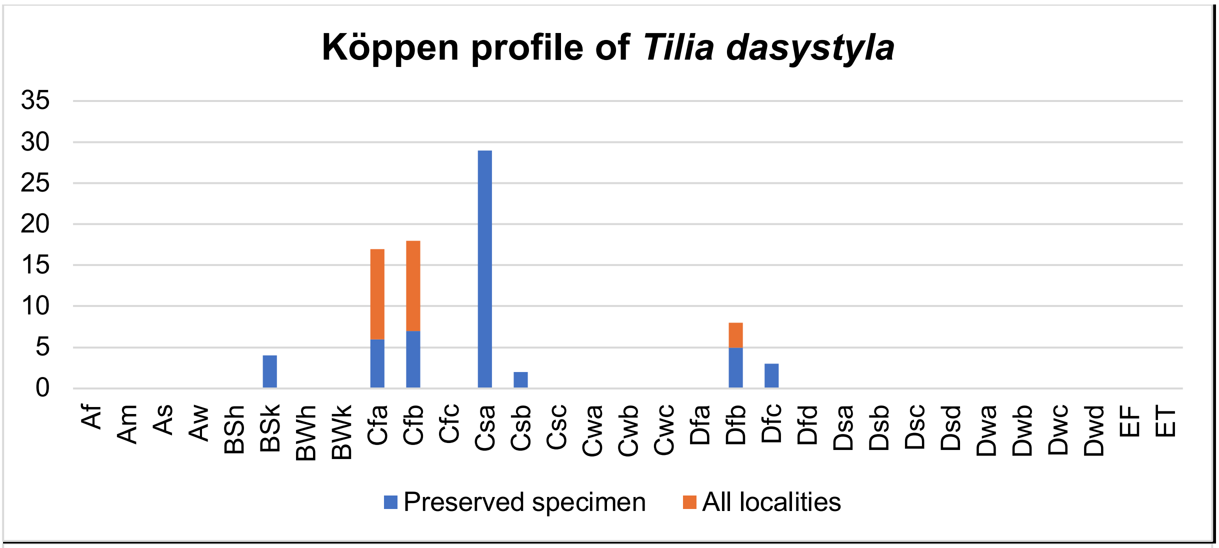

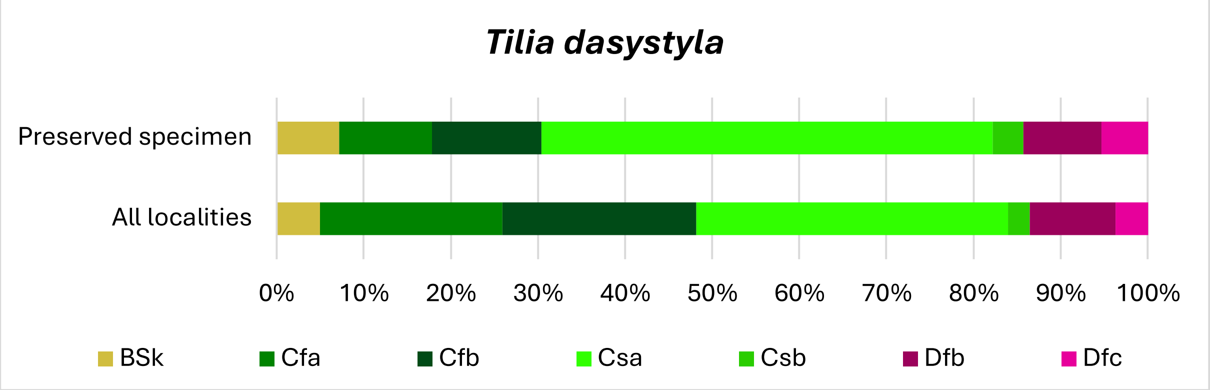

5.1.2.2. Biome profile, distribution, and biome map – GBIF occurrences of *Tilia dasystyla*; excluding duplicate occurrences (n = 81), herbarium specimens (n = 56).

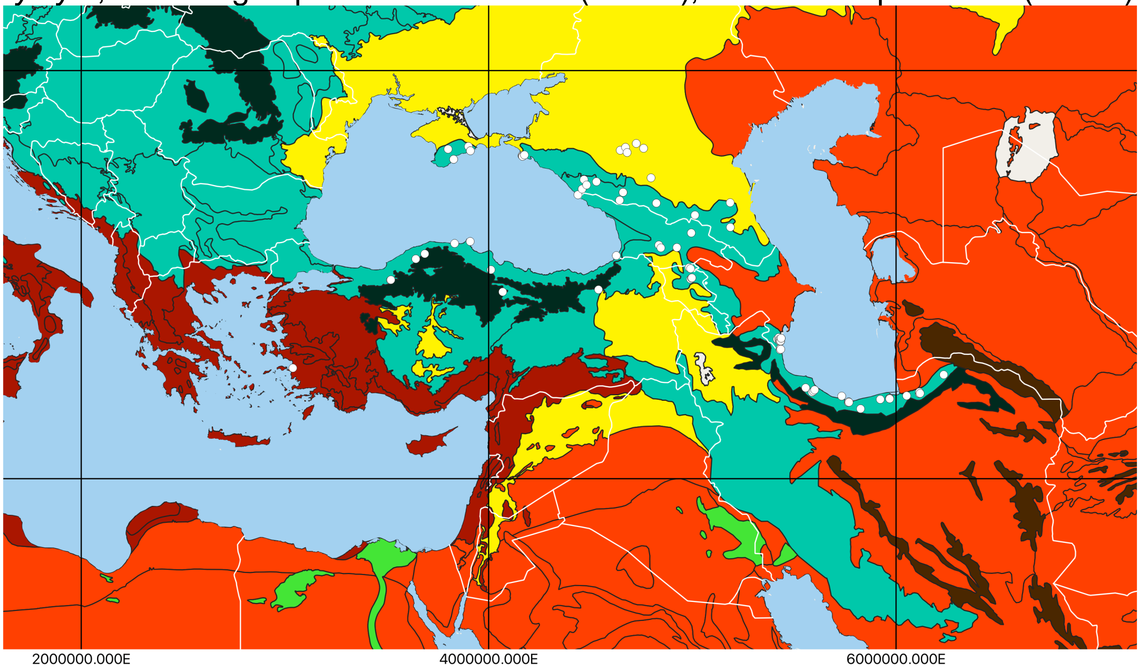

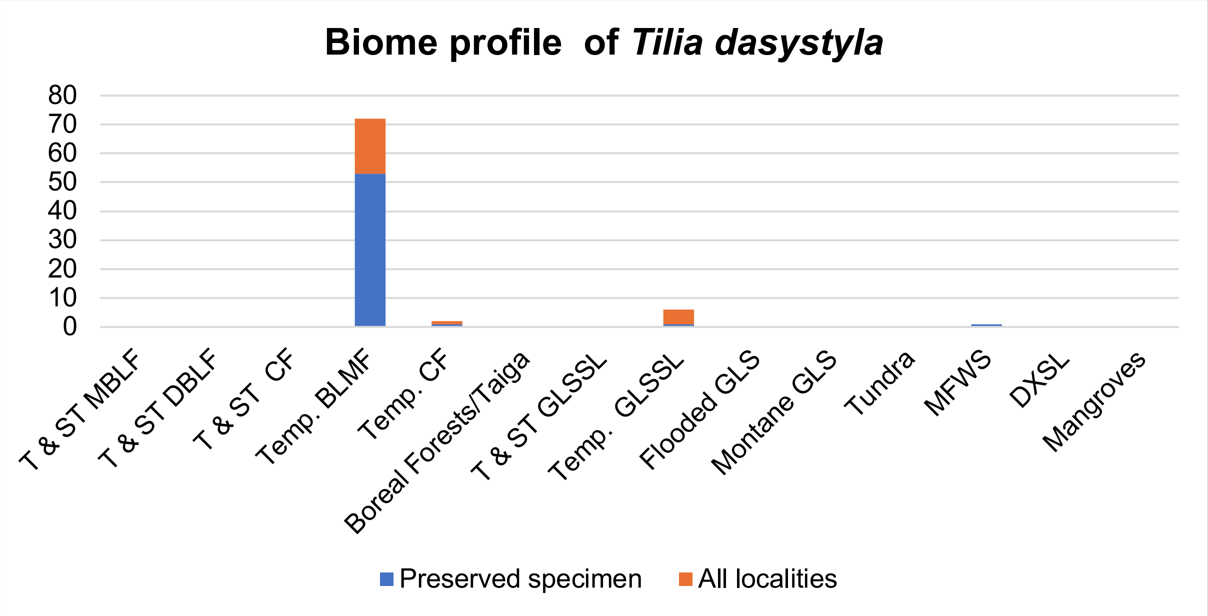

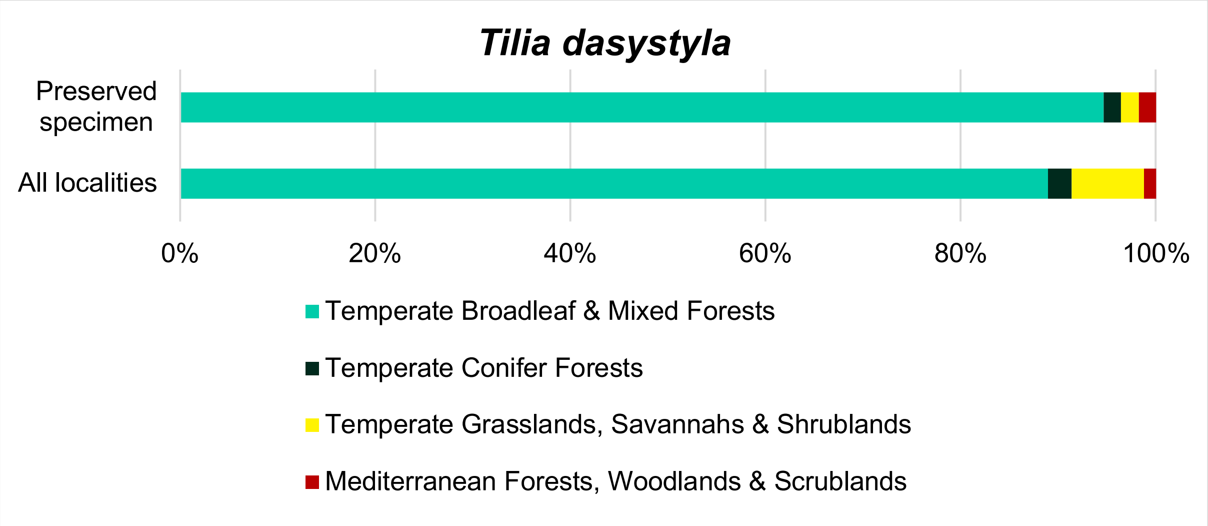

5.1.2.3. Climate graphs - based on 81 *Tilia dasystyla* occurrences in GBIF

5.1.2.3.1. MMT [°C]

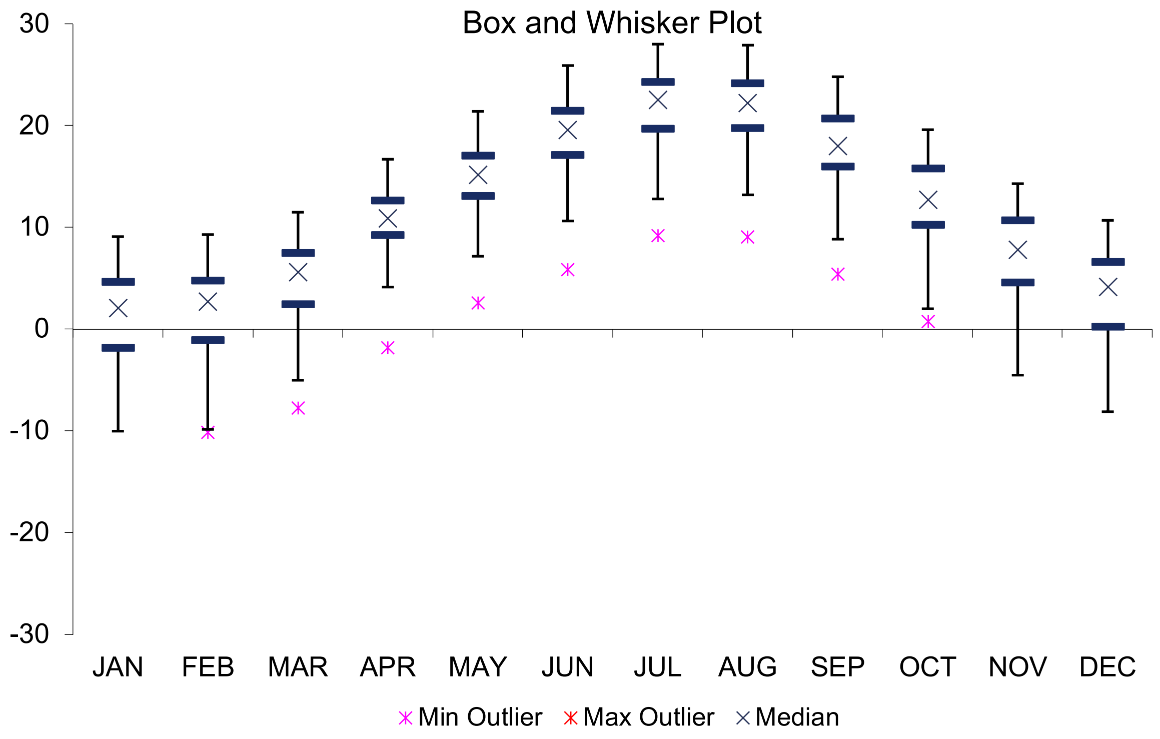

5.1.2.3.2. MinMT [°C]

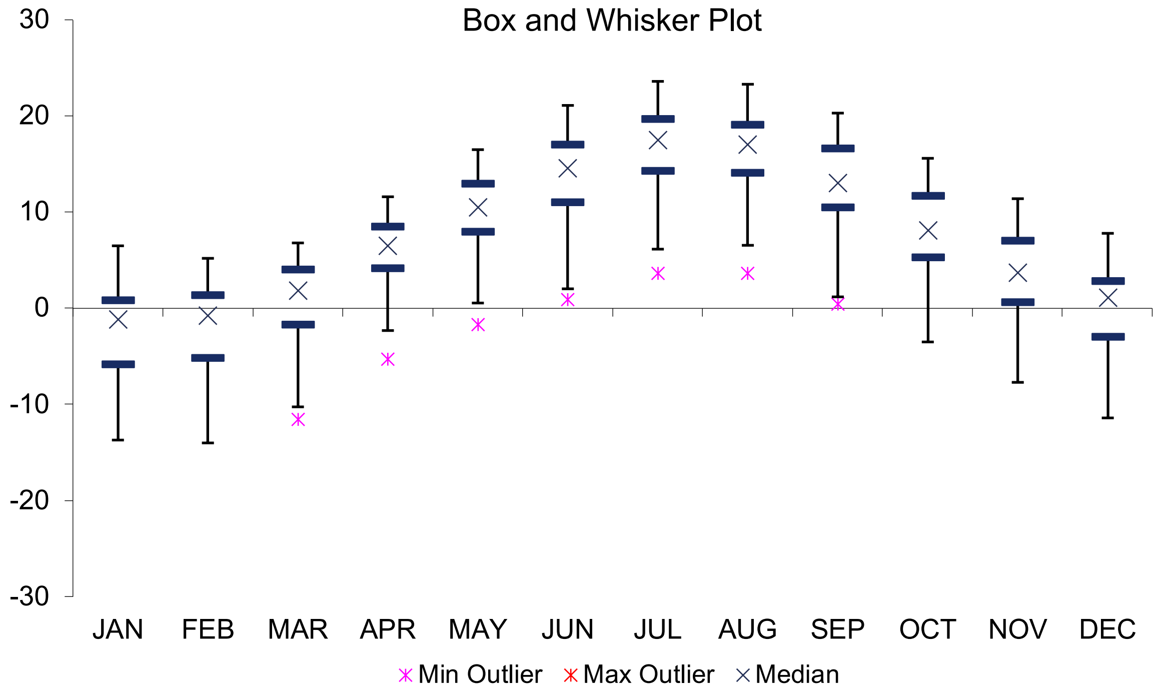

5.1.2.3.3. MMP [mm]

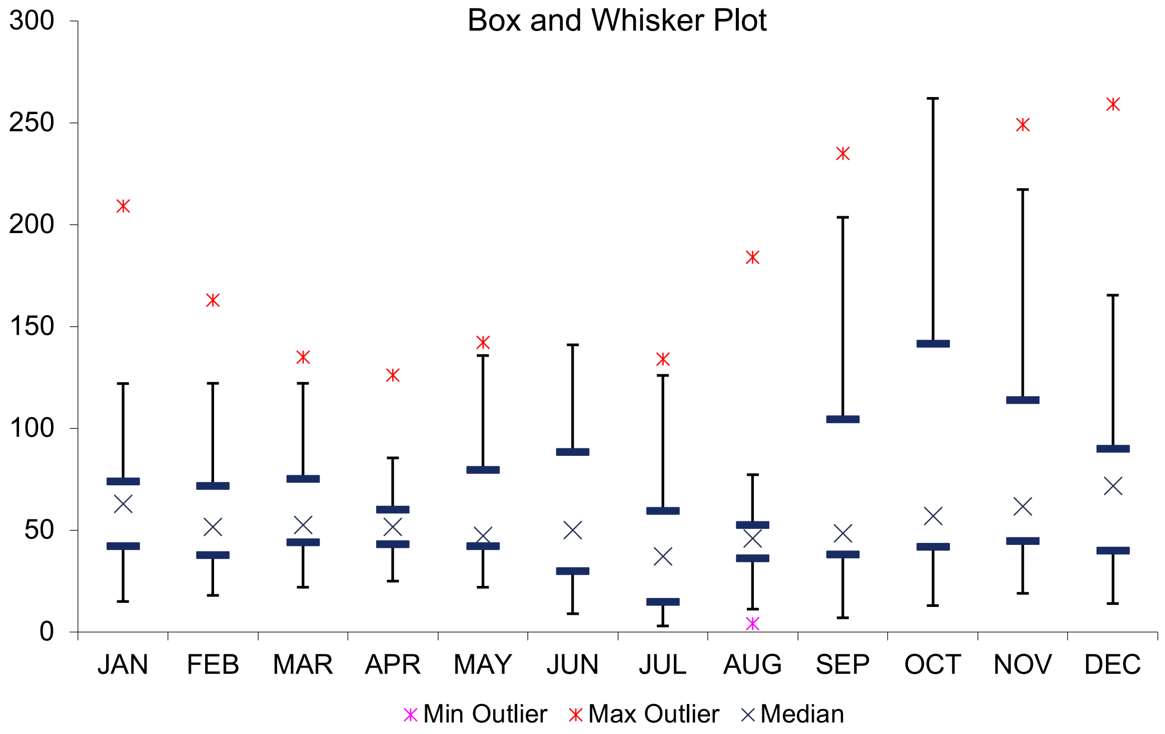

Box and Whisker Plot
300
250
200
150
100
50
0
JAN
FEB
MAR
APR
MAY
JUN
JUL
AUG
SEP
OCT
NOV
DEC
Min Outlier
Max Outlier
Median

5.1.3. Species *Tilia platyphyllos* Mill., 1768

5.1.3.1. Köppen profile, distribution, and climate map – GBIF occurrences of *Tilia platyphyllos*; excluding duplicate occurrences (n = 32684), herbarium specimens (n = 501).

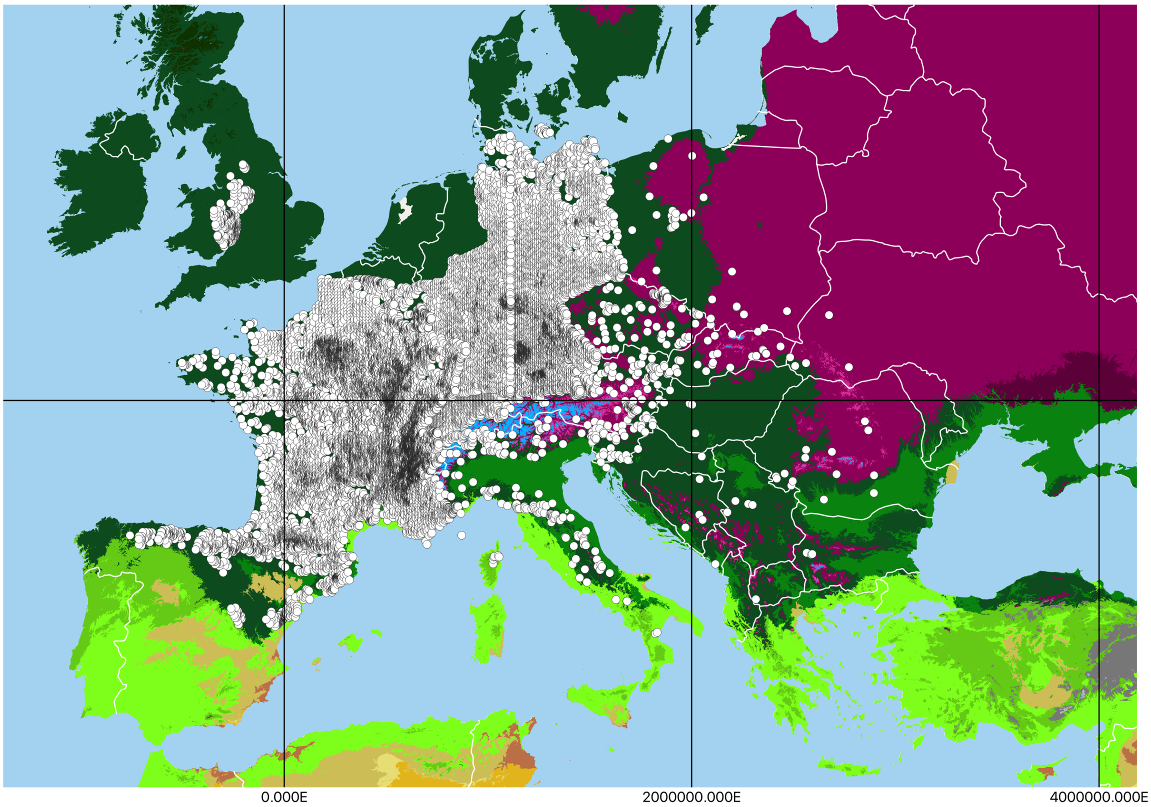

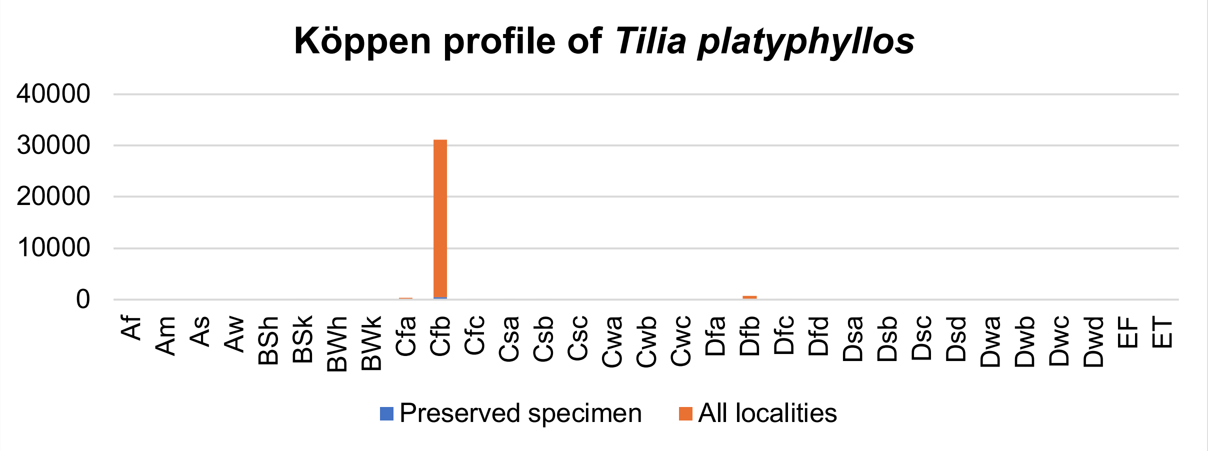

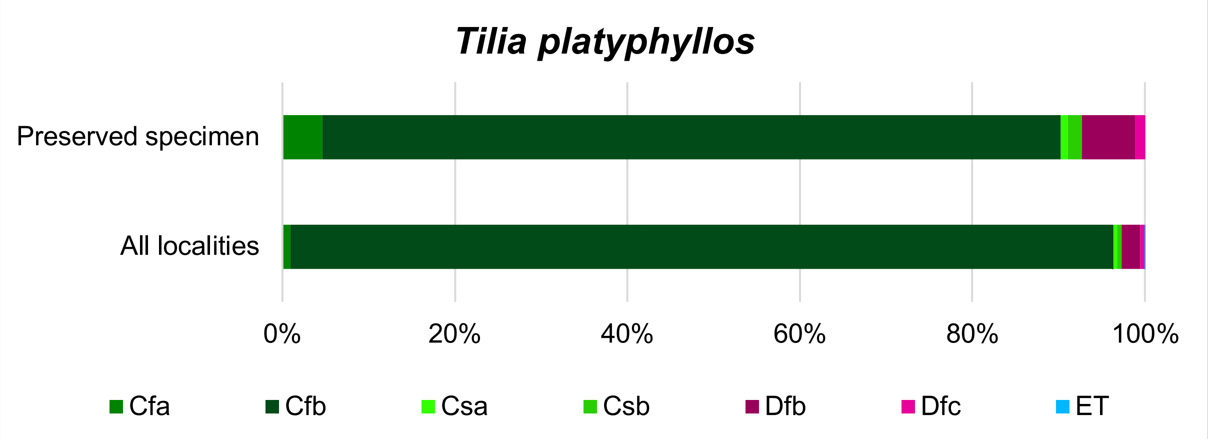

5.1.3.2. Biome profile, distribution, and biome map – GBIF occurrences of *Tilia platyphyllos*; excluding duplicate occurrences (n = 32530), herbarium specimens (n = 479).

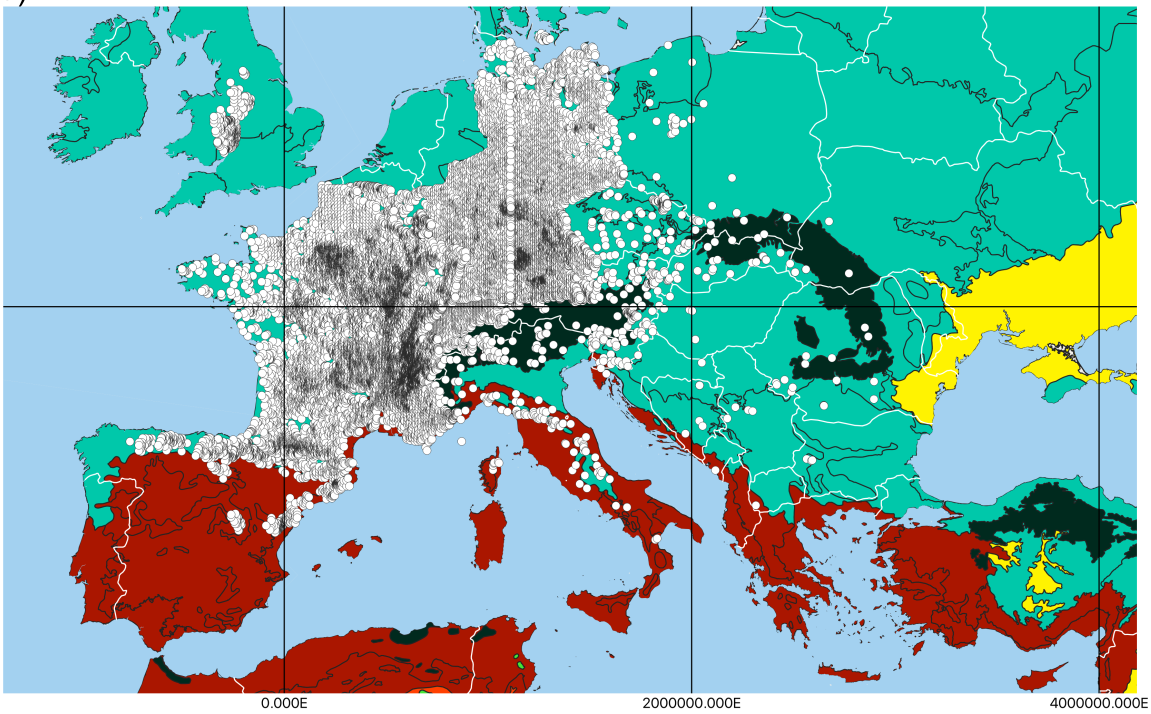

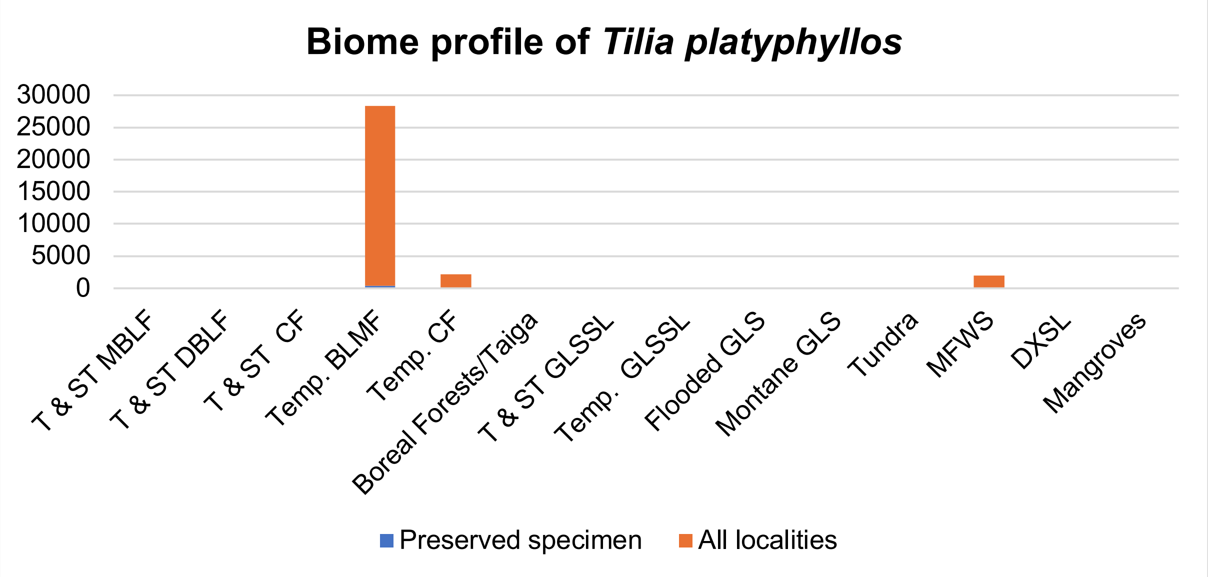

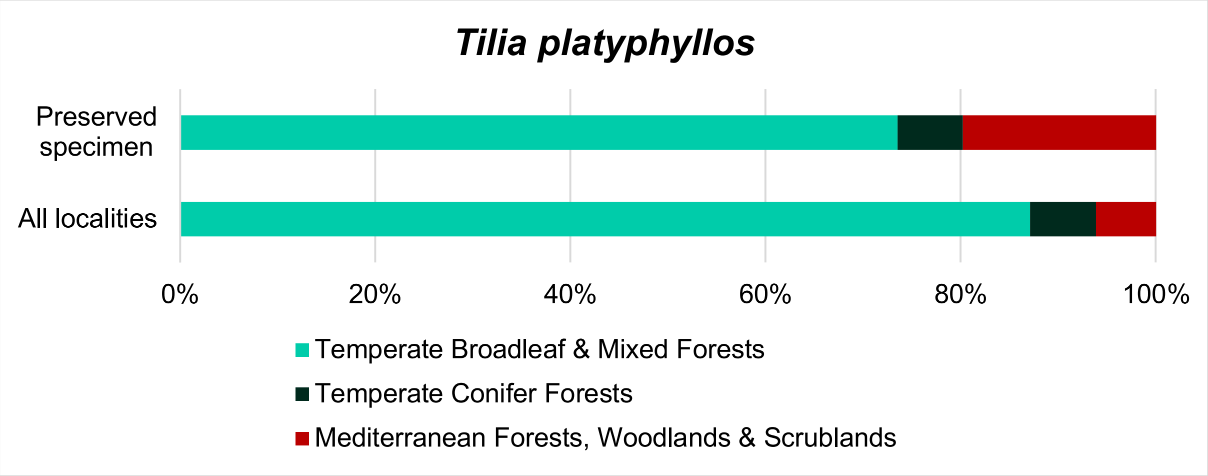

5.1.3.3. Climate graphs - based on 32665 *Tilia platyphyllos* occurrences in GBIF

5.1.3.3.1. MMT [°C]

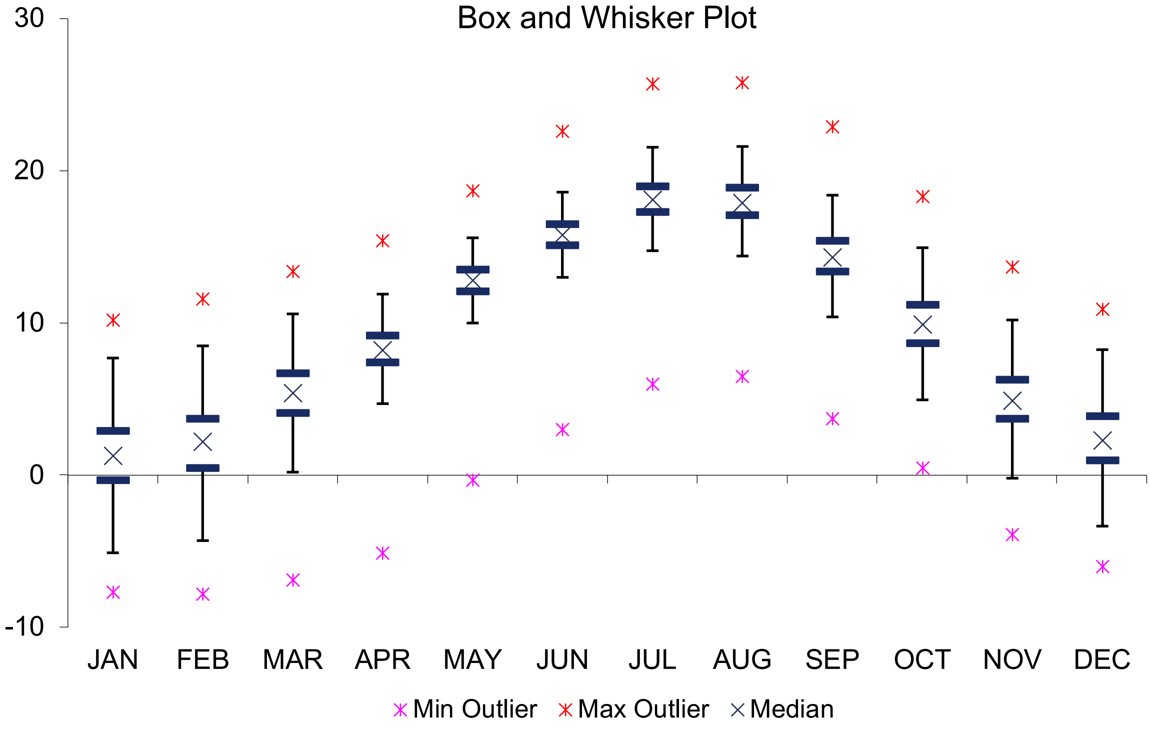

5.1.3.3.2. MinMT [°C]

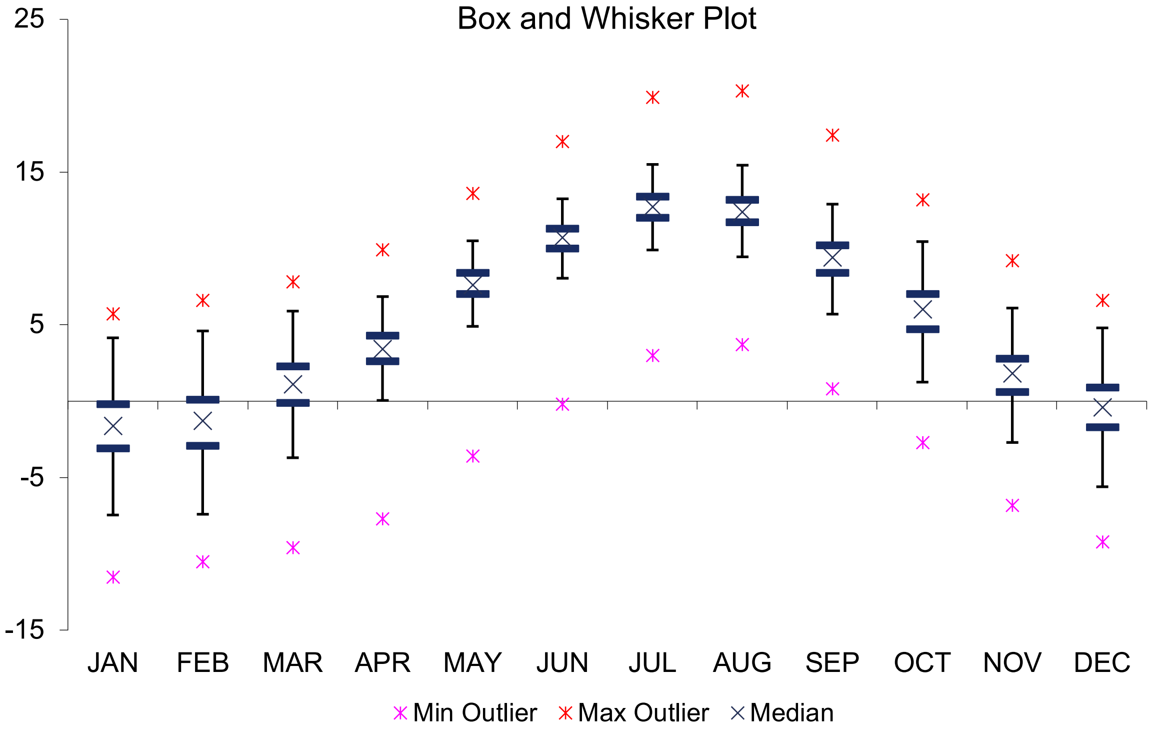

5.1.3.3.3. MMP [mm]

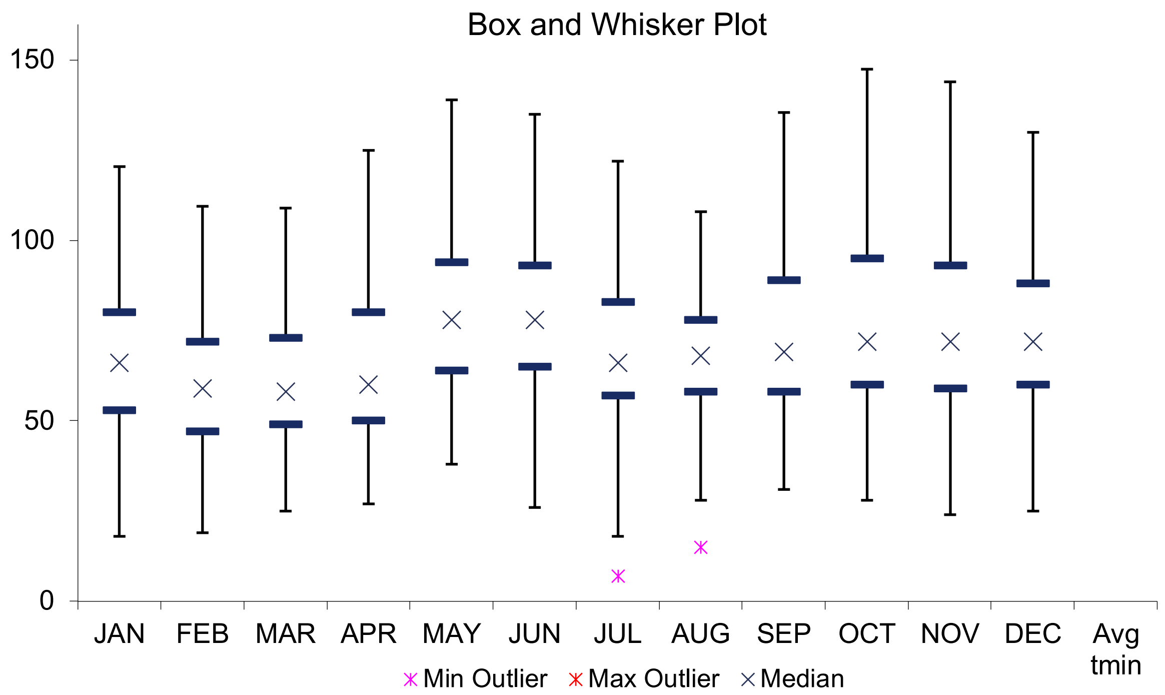
Box and Whisker Plot
150
100
50
0
JAN
FEB
MAR
APR
MAY
JUN
JUL
AUG
SEP
OCT
NOV
DEC
Avg tmin
Min Outlier
Max Outlier
Median

5.1.4. Species *Tilia tomentosa* Mill., 1768

5.1.4.1 Köppen profile, distribution, and climate map – GBIF occurrences of *Tilia tomentosa*; excluding duplicate occurrences (n = 195), herbarium specimens (n = 37).

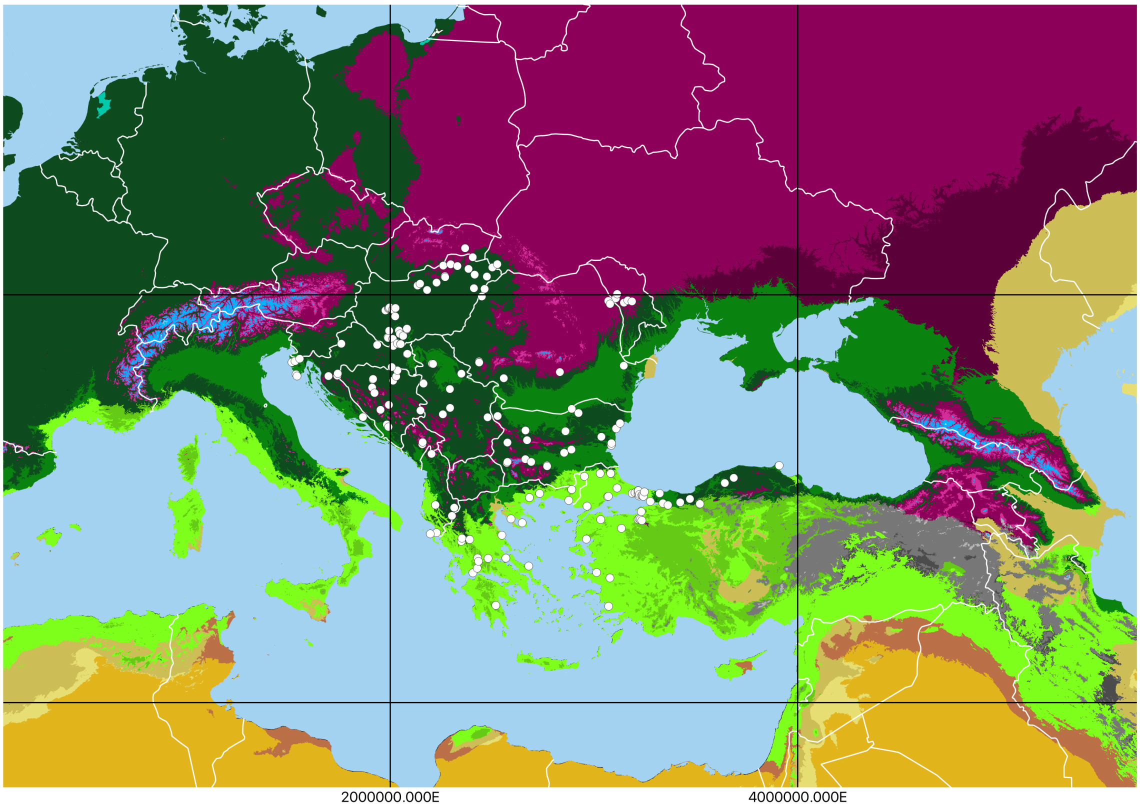

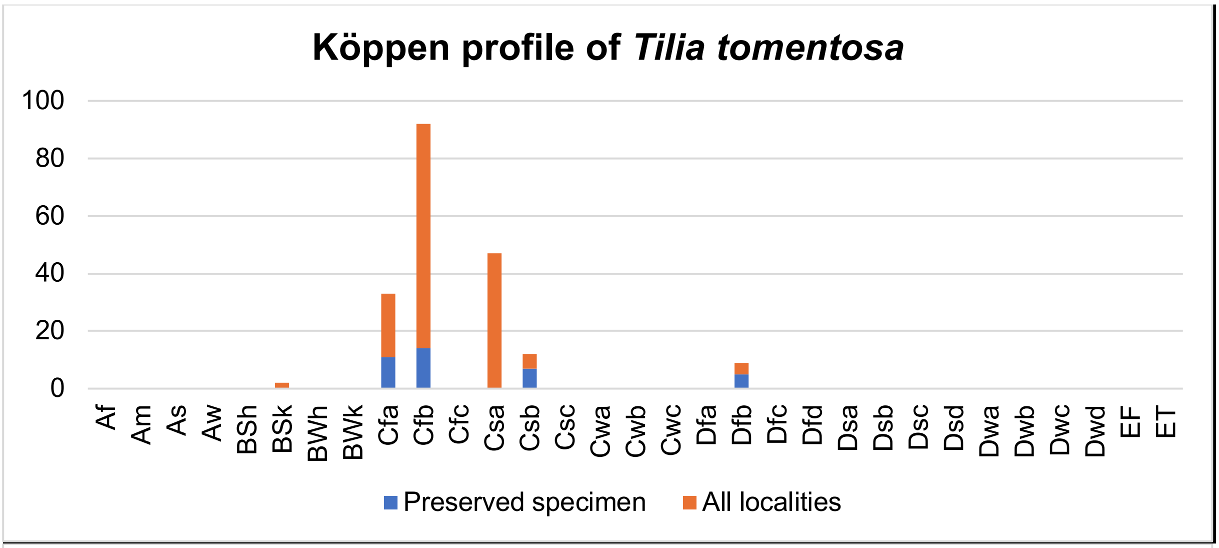

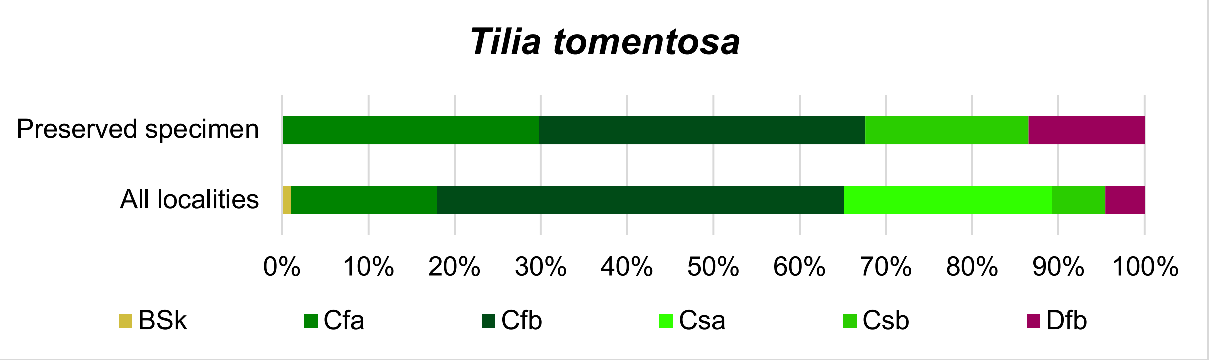

5.1.4.2. Biome profile, distribution, and biome map – GBIF occurrences of *Tilia tomentosa*; excluding duplicate occurrences (n = 190), herbarium specimens (n = 37).

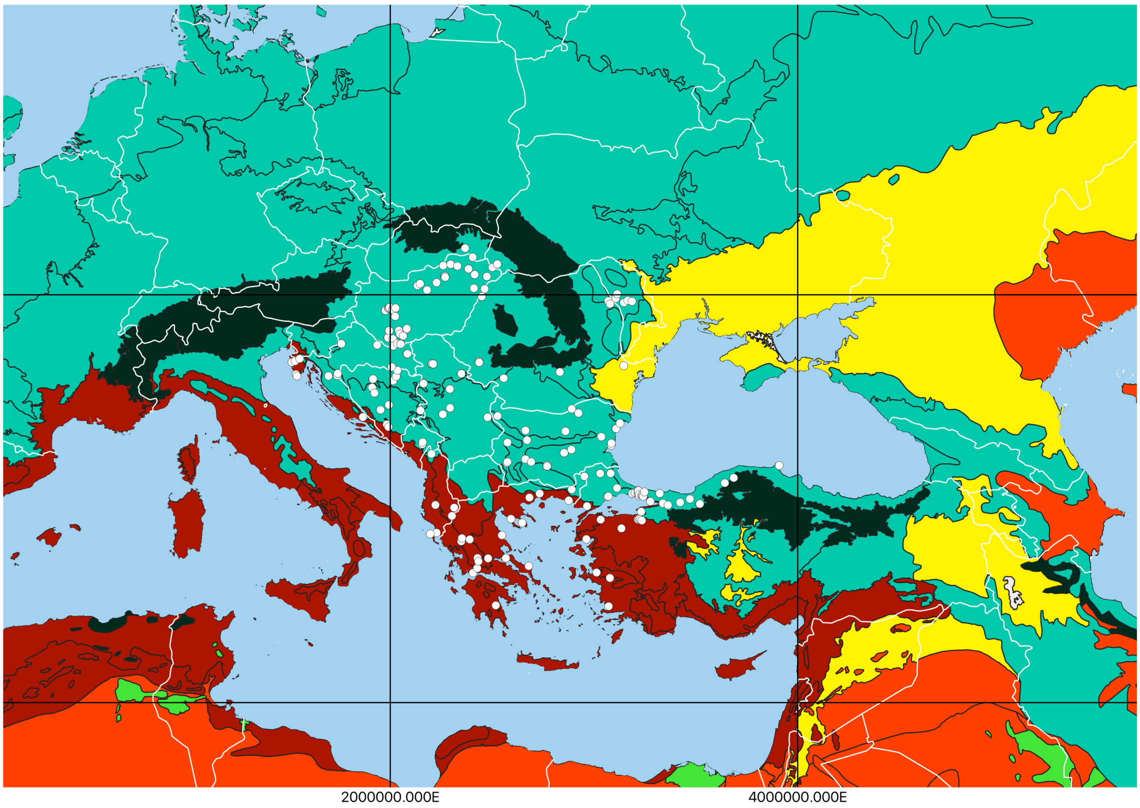

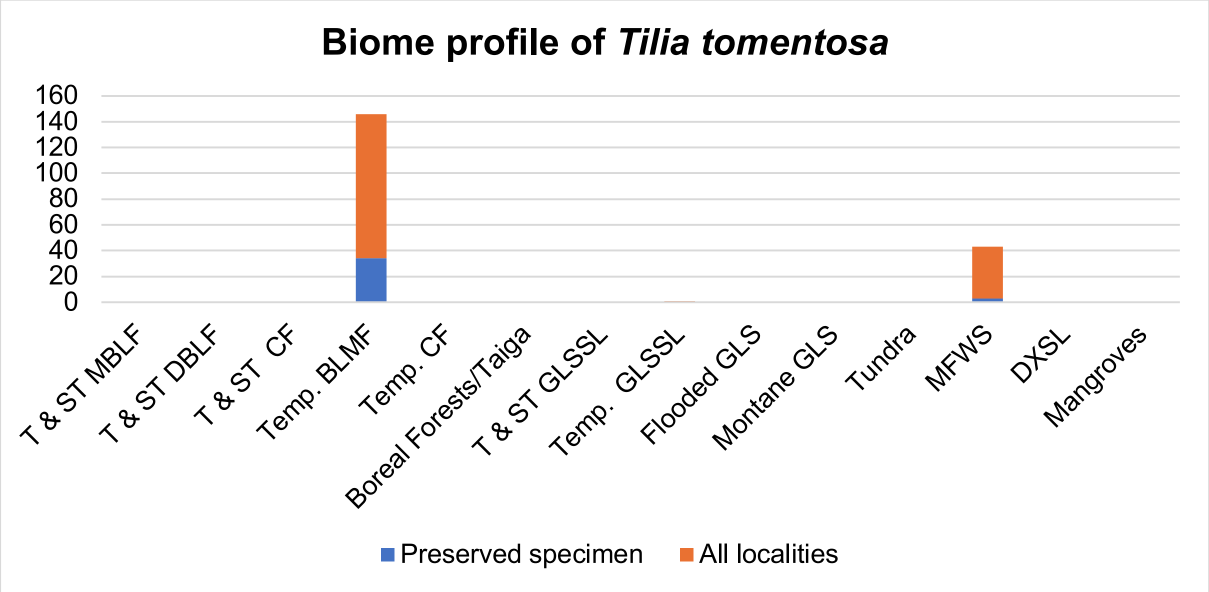

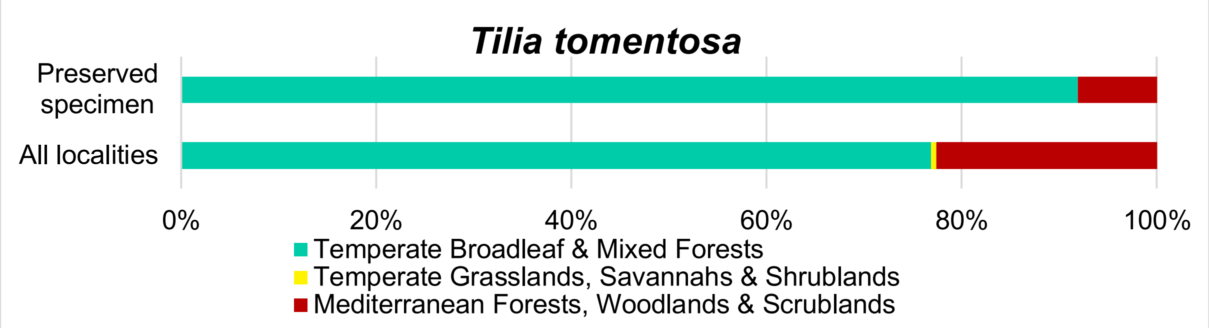

5.1.4.3. Climate graphs - based on 193 *Tilia tomentosa* occurrences in GBIF

5.1.4.3.1. MMT [°C]

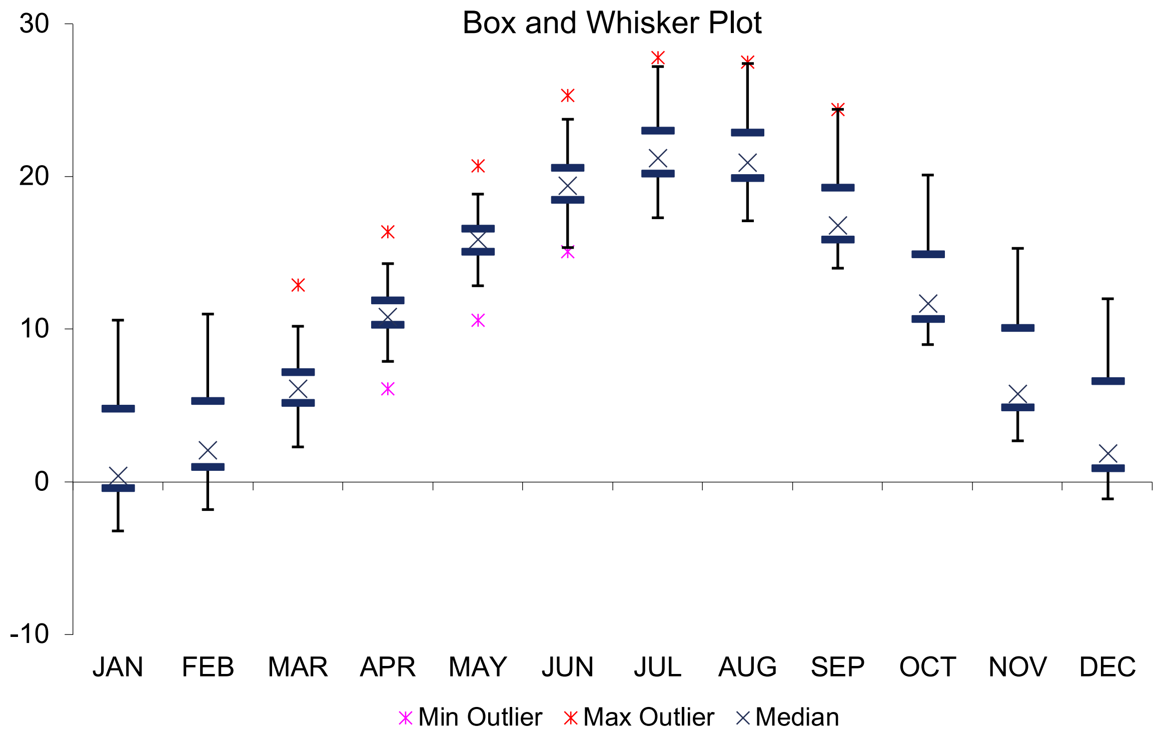

5.1.4.3.2. MinMT [°C]

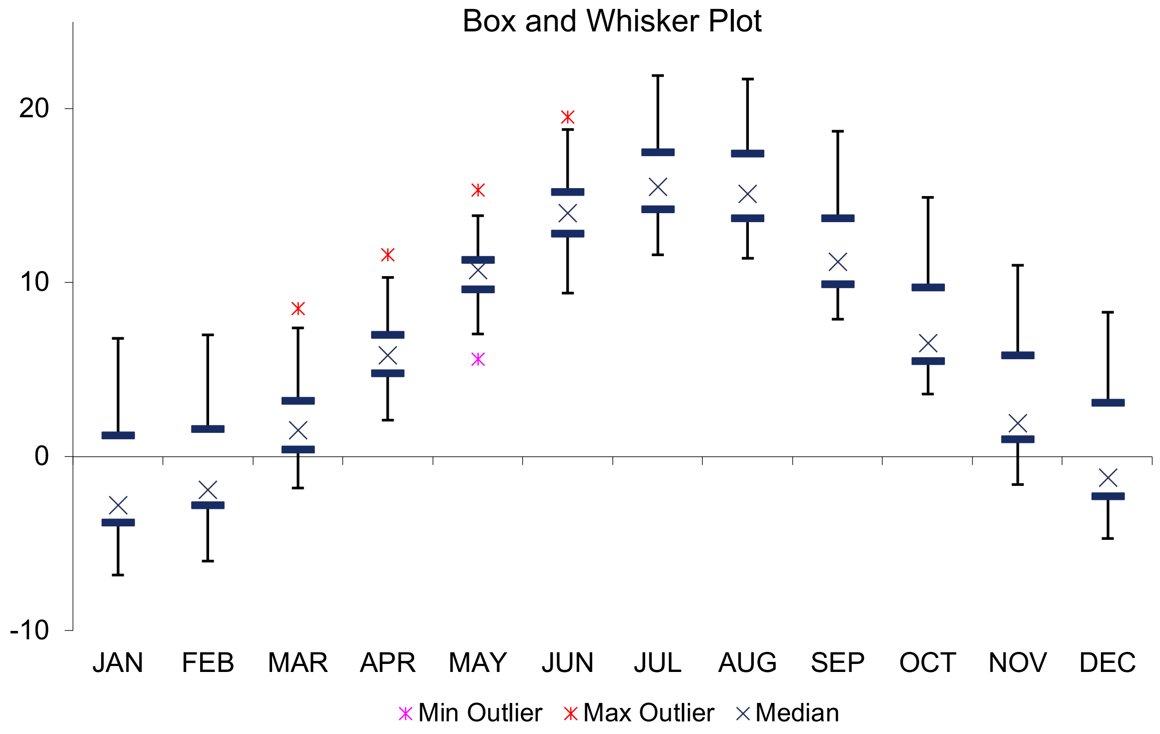

5.1.4.3.3. MMP [mm]

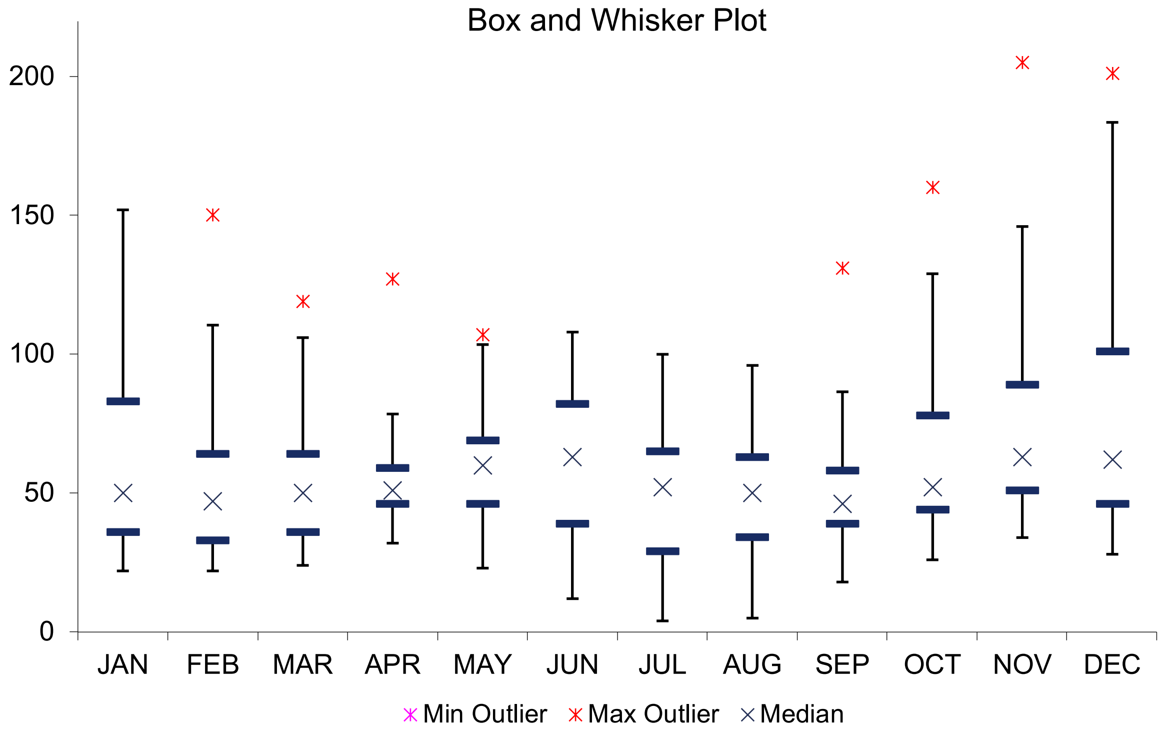
Box and Whisker Plot
200
150
100
50
0
JAN
FEB
MAR
APR
MAY
JUN
JUL
AUG
SEP
OCT
NOV
DEC
Min Outlier
Max Outlier
Median

5.2. American Tilia taxa

5.2.1. Species *Tilia americana* Mill., 1768

5.2.1.1 Köppen profile, distribution, and climate map – GBIF occurrences of *Tilia americana*; excluding duplicate occurrences (n = 28968), herbarium specimens (n = 2928).

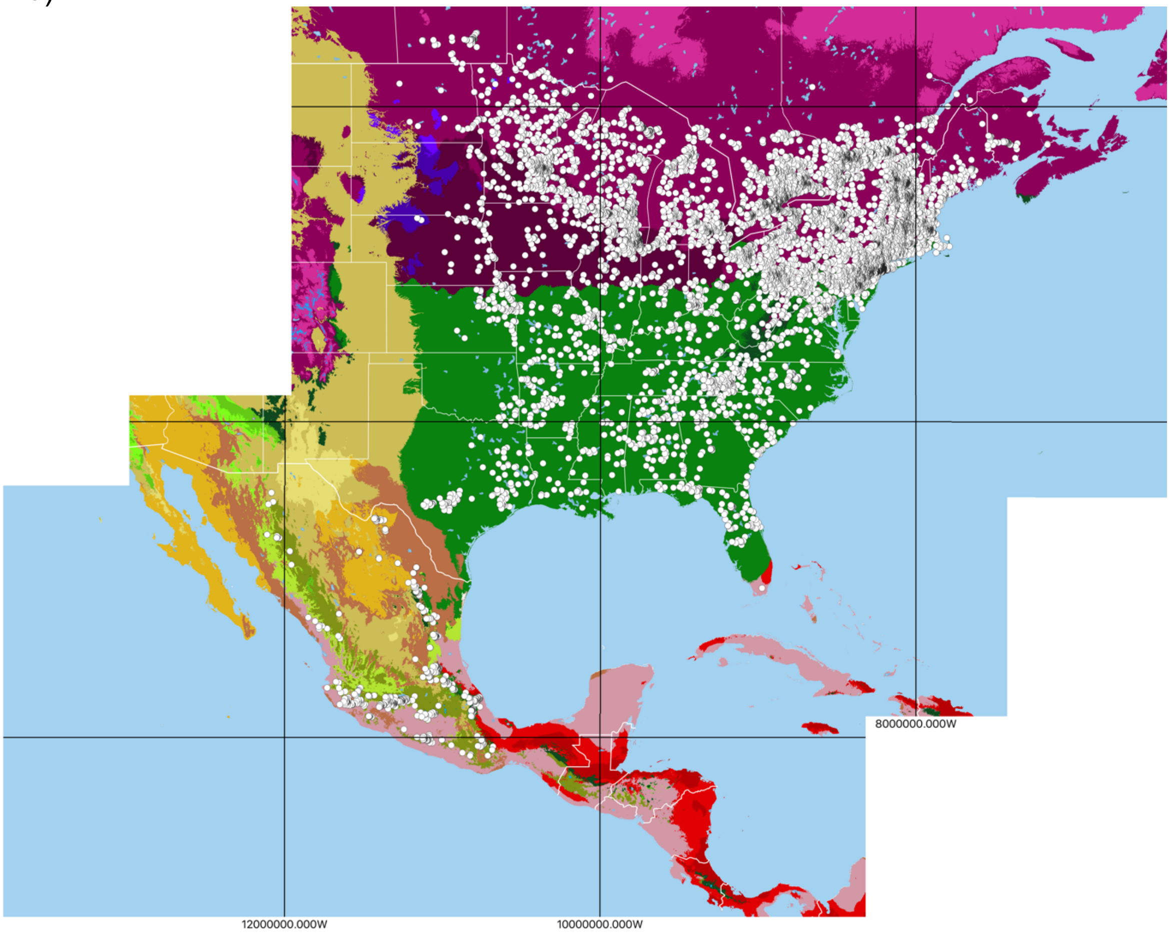

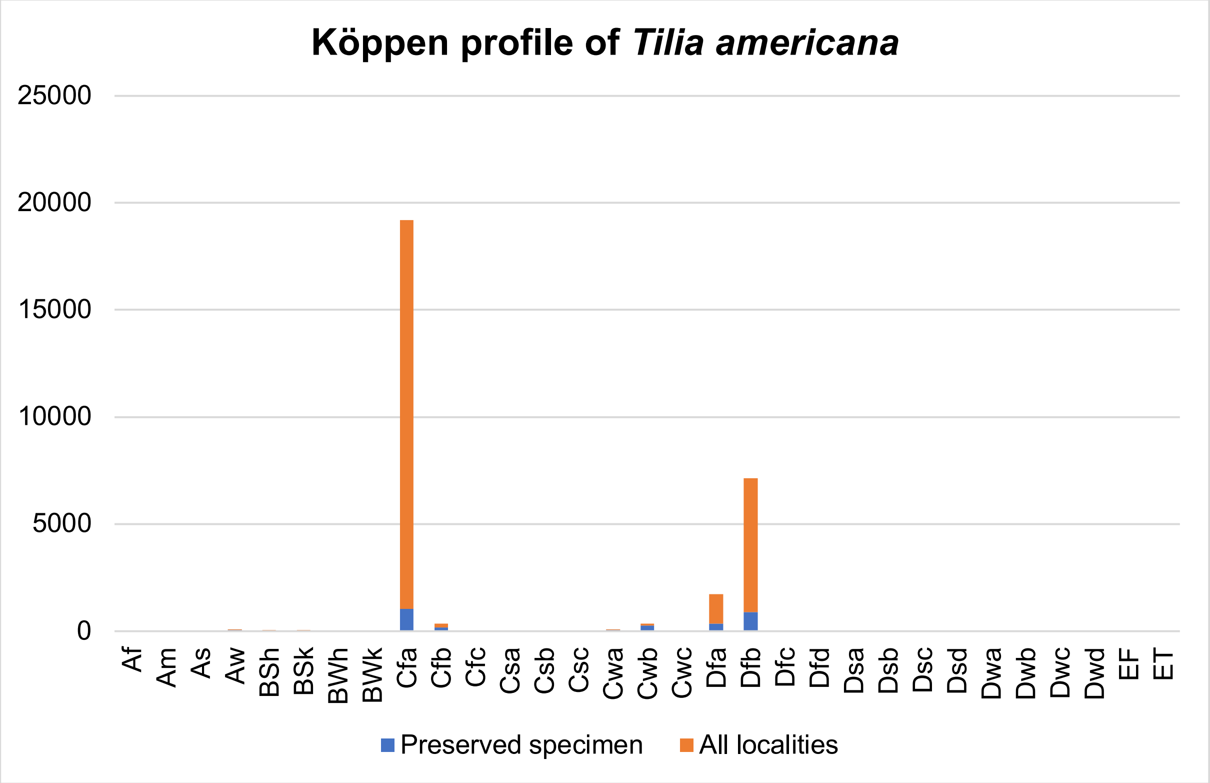

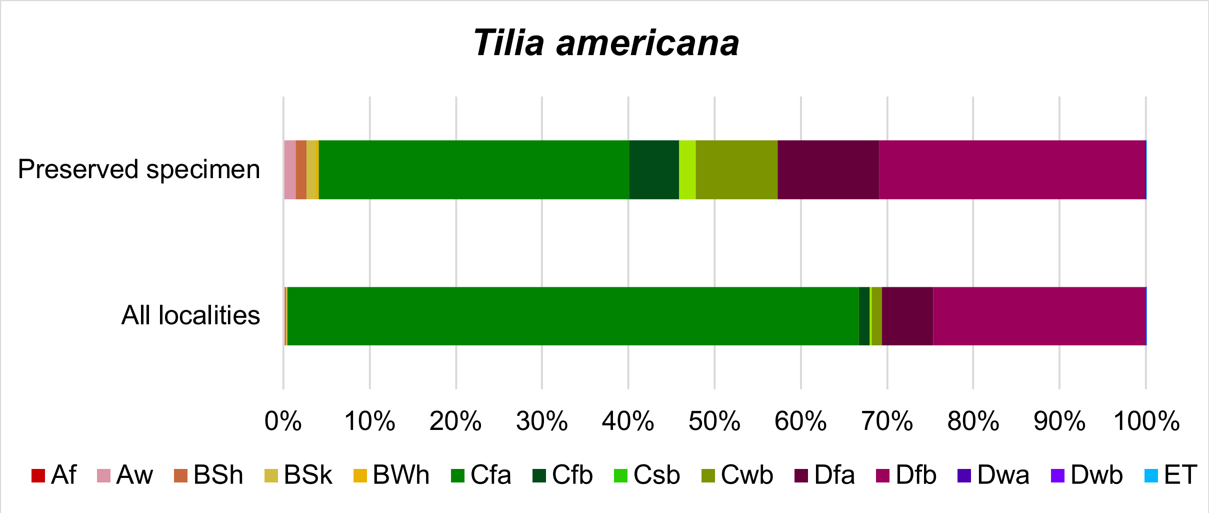

5.2.1.2. Biome profile, distribution, and biome map – GBIF occurrences of *Tilia americana*; excluding duplicate occurrences (n = 28825), herbarium specimens (n = 2904).

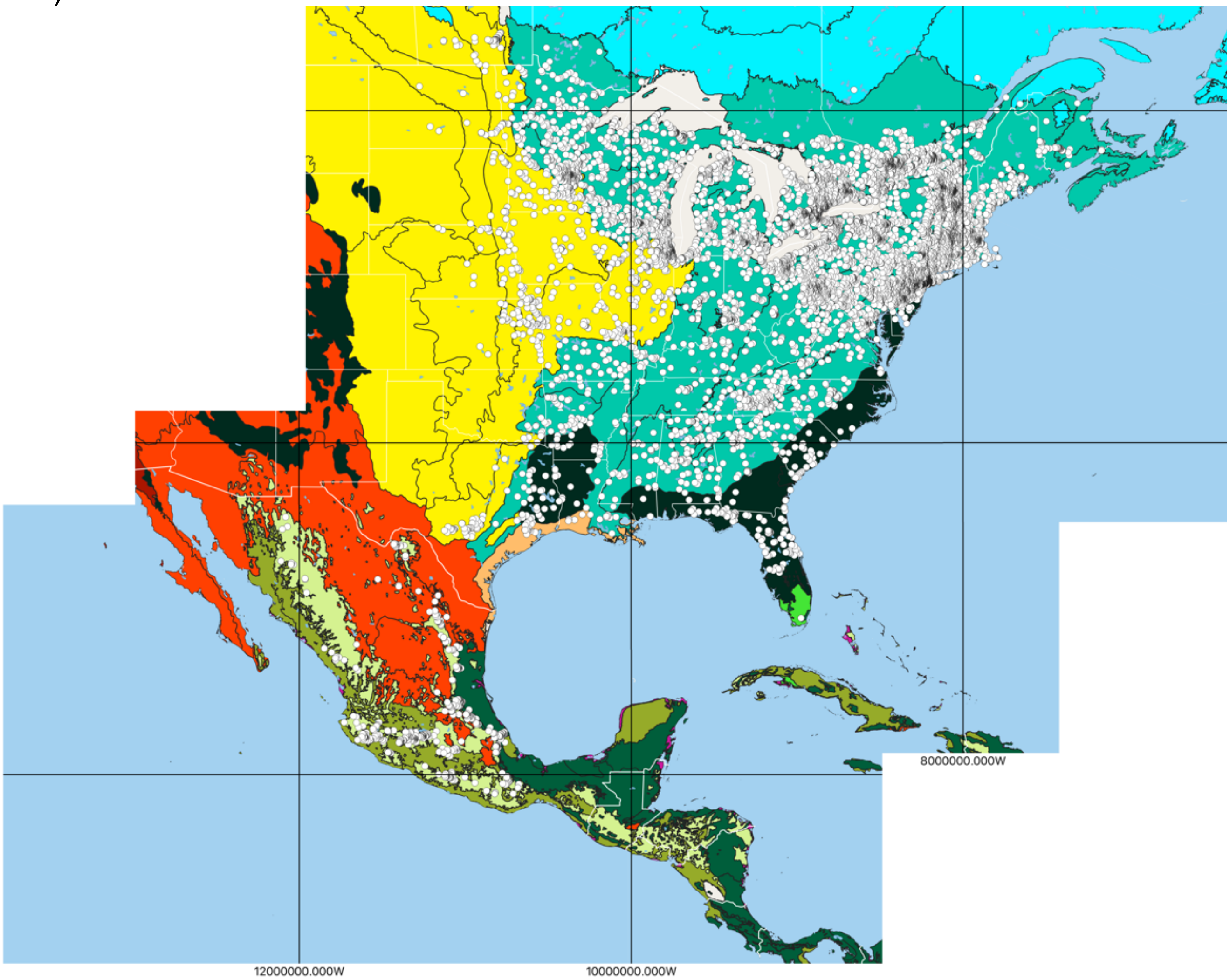

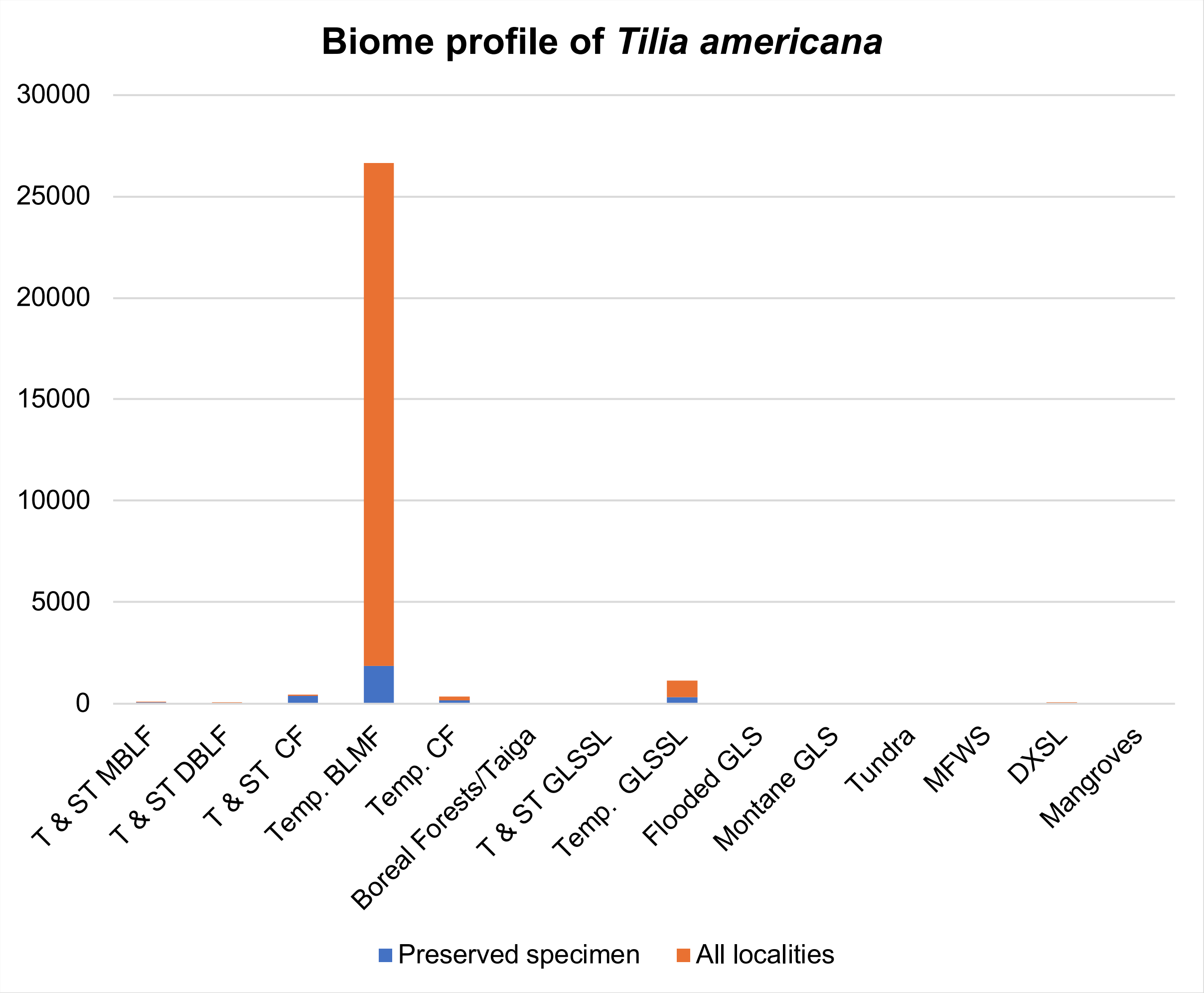
Biome profile of Tilia americana
30000
25000
20000
15000
10000
5000
0
T & ST MBLF
T & ST DBLF
T & ST CF
Temp. BLMF
Temp. CF
Boreal Forests/Taiga
T & ST GLSSL
Temp. GLSSL
Flooded GLS
Montane GLS
Tundra
MFWS
DXSL
Mangroves
Preserved specimen
All localities

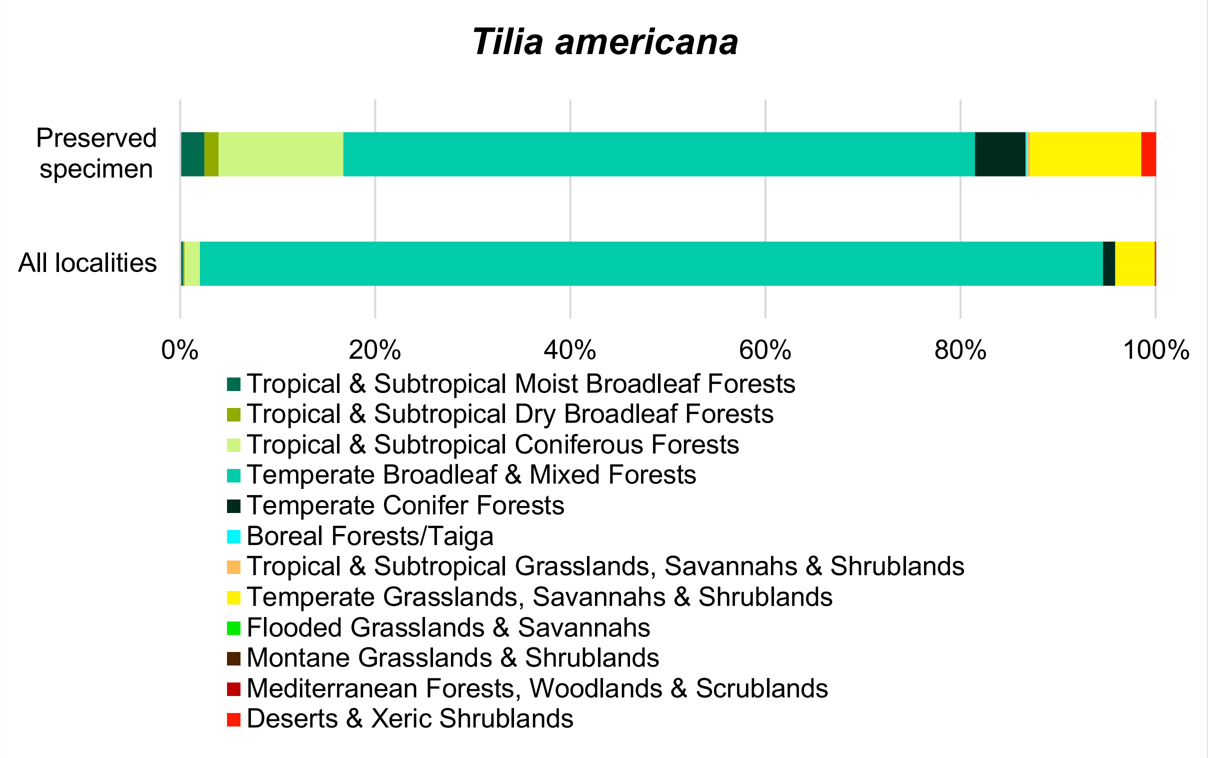
Tilia americana
Preserved specimen
All localities
0%
20%
40%
60%
80%
100%
Tropical & Subtropical Moist Broadleaf Forests
Tropical & Subtropical Dry Broadleaf Forests
Tropical & Subtropical Coniferous Forests
Temperate Broadleaf & Mixed Forests
Temperate Conifer Forests
Boreal Forests/Taiga
Tropical & Subtropical Grasslands, Savannahs & Shrublands
Temperate Grasslands, Savannahs & Shrublands
Flooded Grasslands & Savannahs
Montane Grasslands & Shrublands
Mediterranean Forests, Woodlands & Scrublands
Deserts & Xeric Shrublands

5.2.1.3. Climate graphs - based on 27967 *Tilia americana* occurrences in GBIF

5.2.1.3.1. MMT [°C]

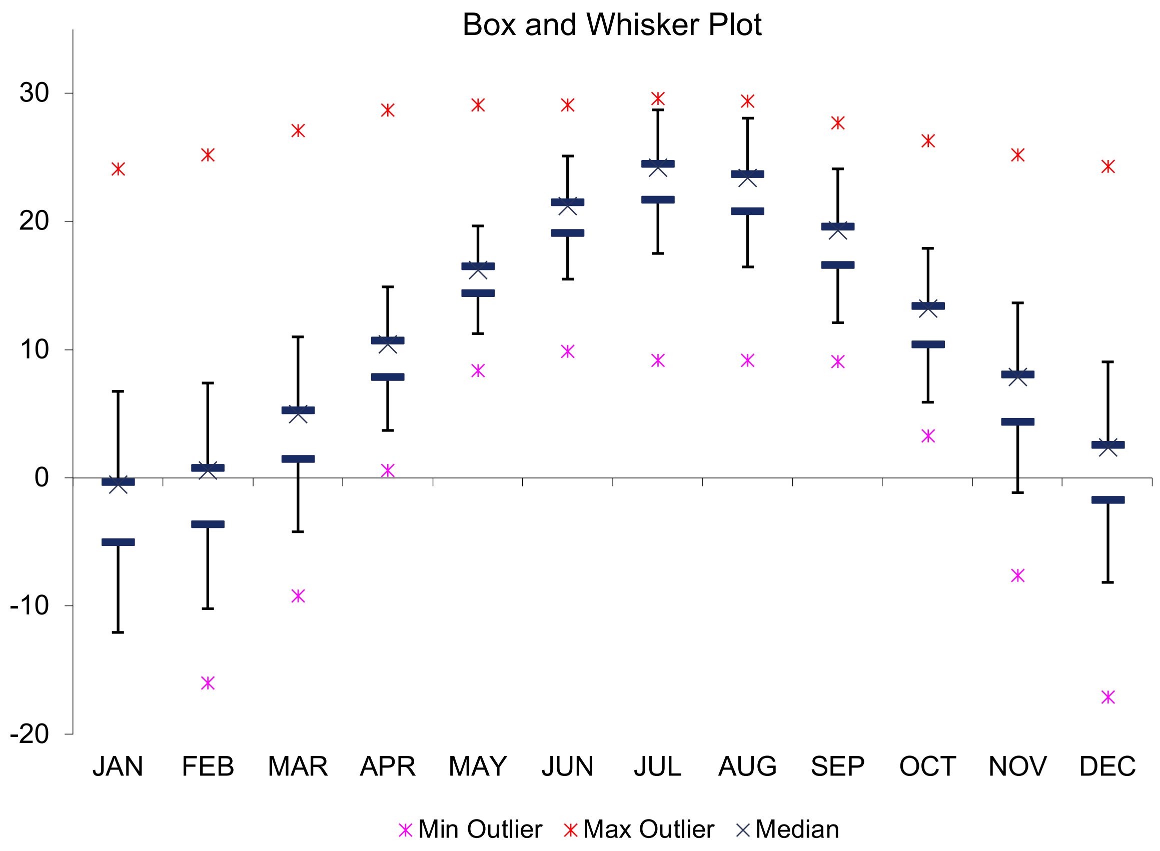

5.2.1.3.2. MinMT [°C]

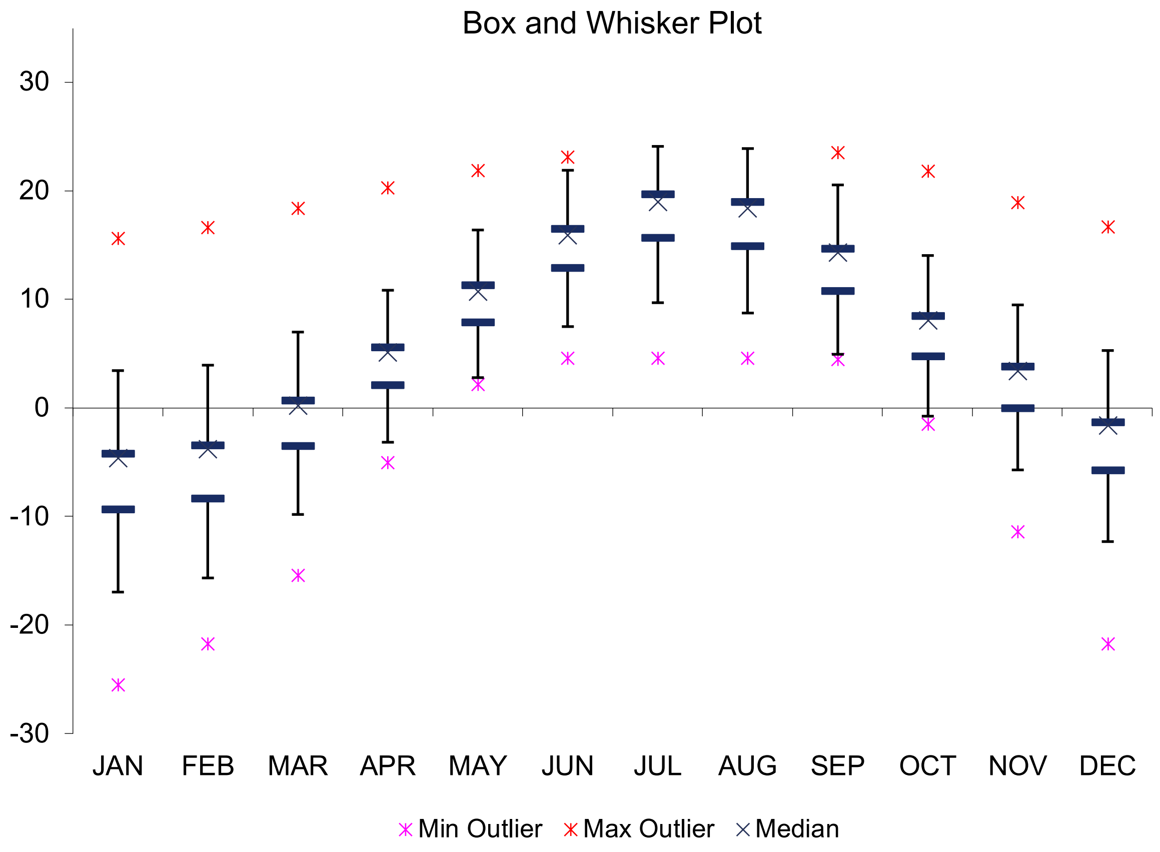

5.2.1.3.3. MMP [mm]

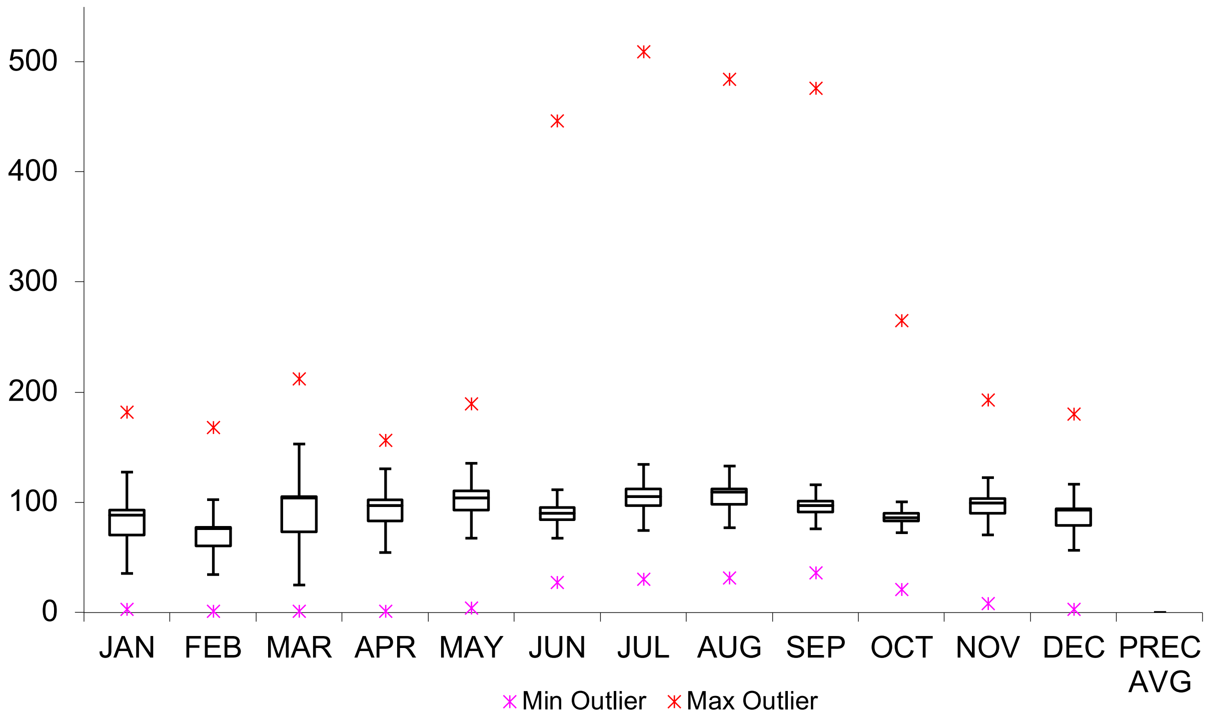

500
400
300
200
100
0
JAN
FEB
MAR
APR
MAY
JUN
JUL
AUG
SEP
OCT
NOV
DEC
PREC AVG
Min Outlier
Max Outlier

5.3. East Asian *Tilia* taxa
5.3.1. Tilia section Anastraea
5.3.1.1. Species *Tilia amurensis* Rupr., 1869
5.3.1.1.1. Köppen profile, distribution, and climate map – GBIF occurrences of *Tilia amurensis*; excluding duplicate occurrences (n = 1019), herbarium specimens (n = 464).

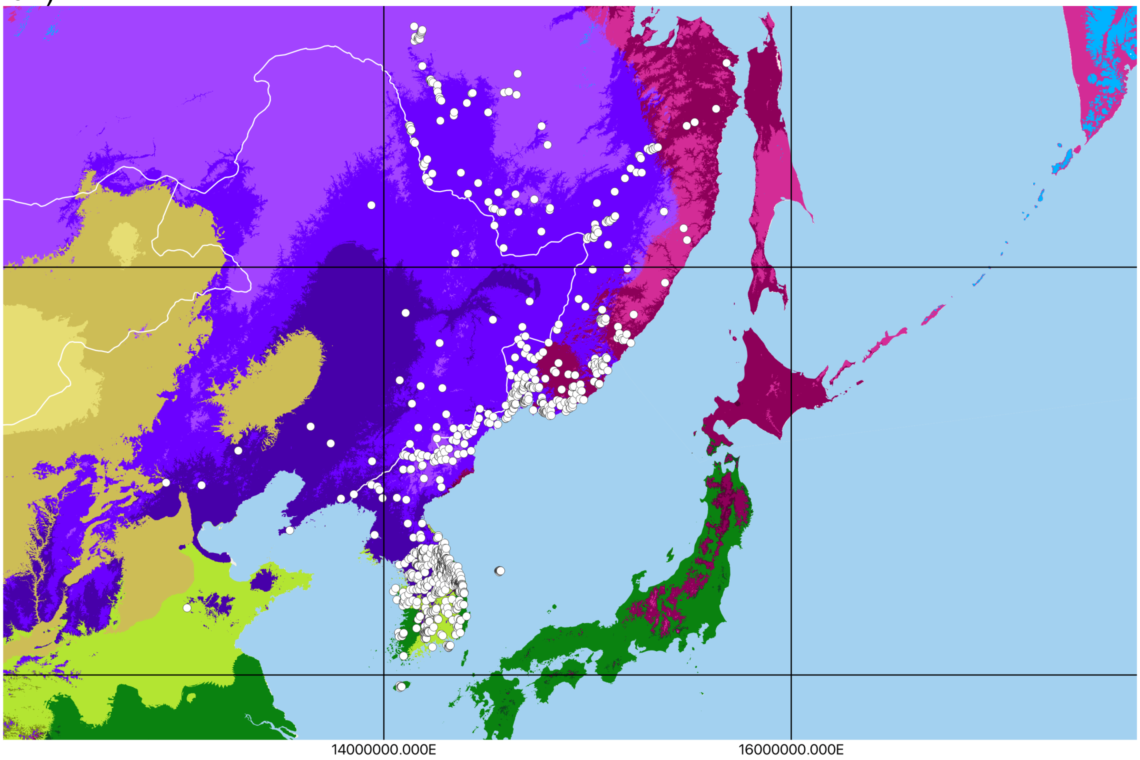

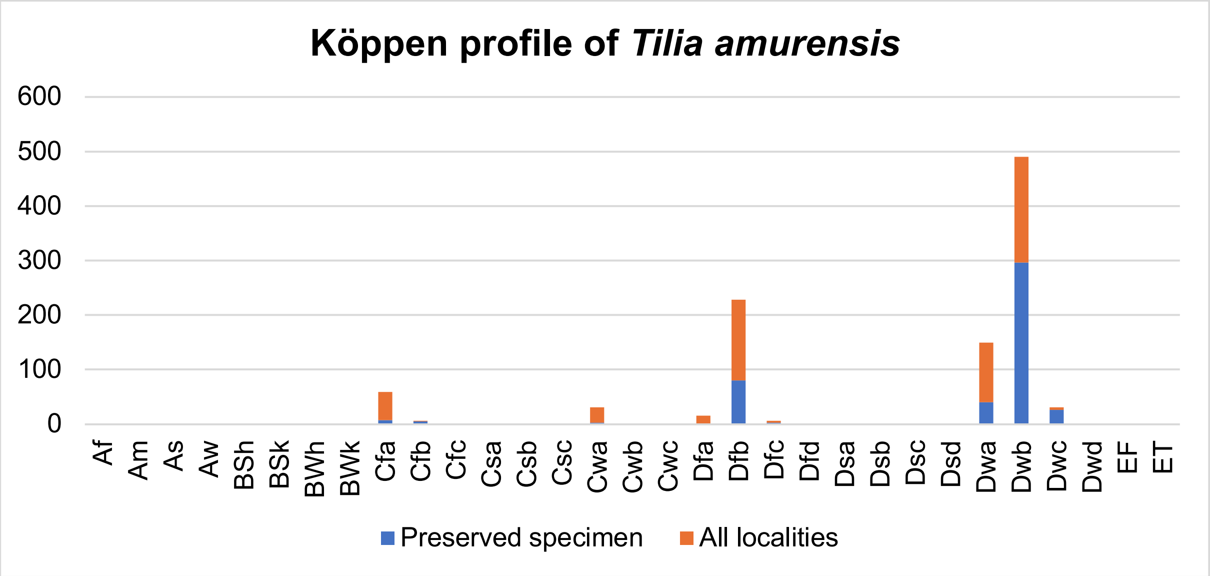

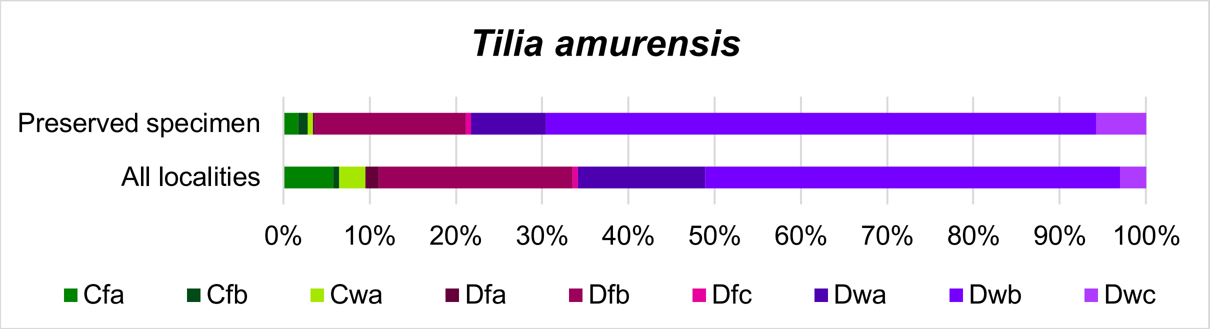

5.3.1.1.2. Biome profile, distribution, and biome map – GBIF occurrences of *Tilia amurensis*; excluding duplicate occurrences (n = 990), herbarium specimens (n = 450).

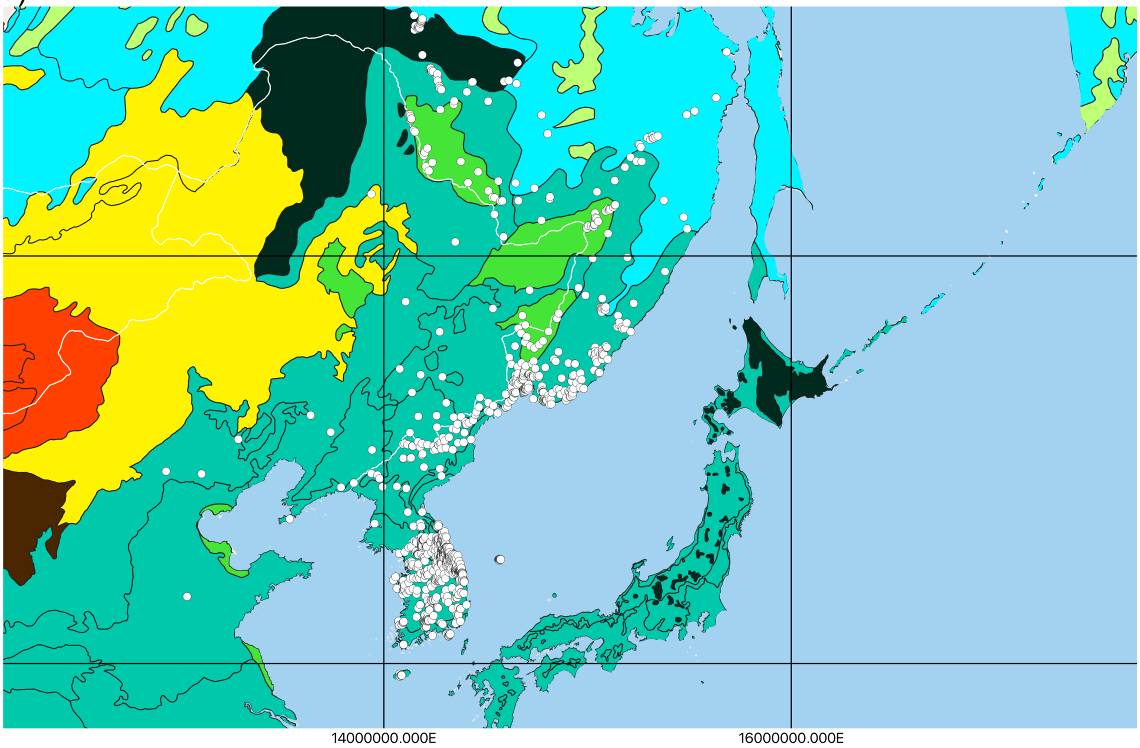

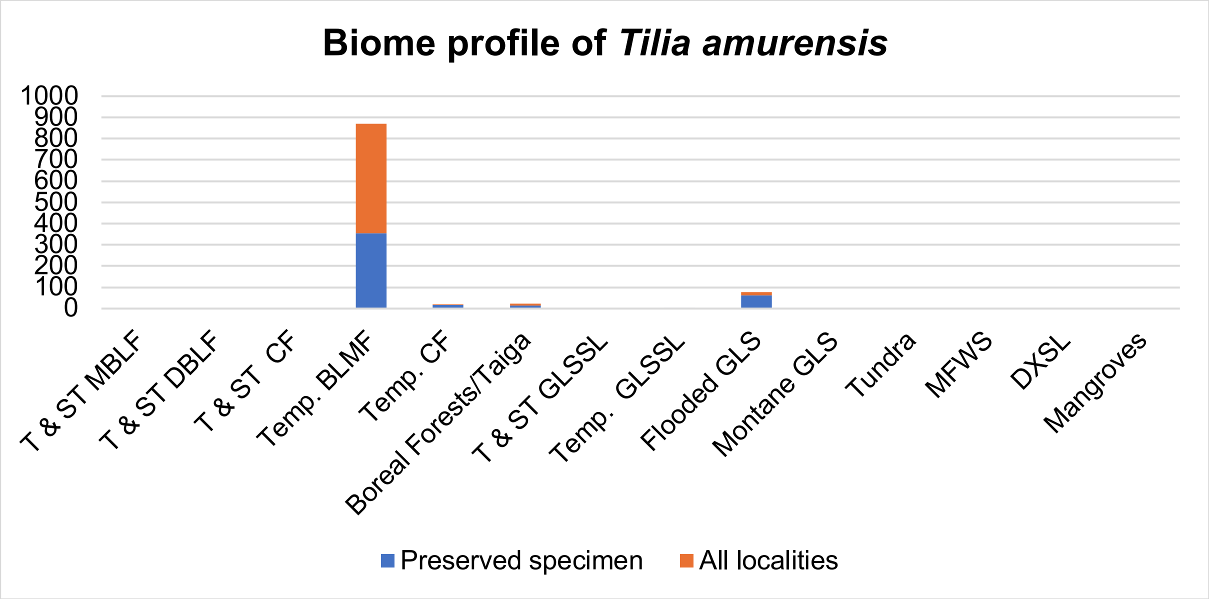

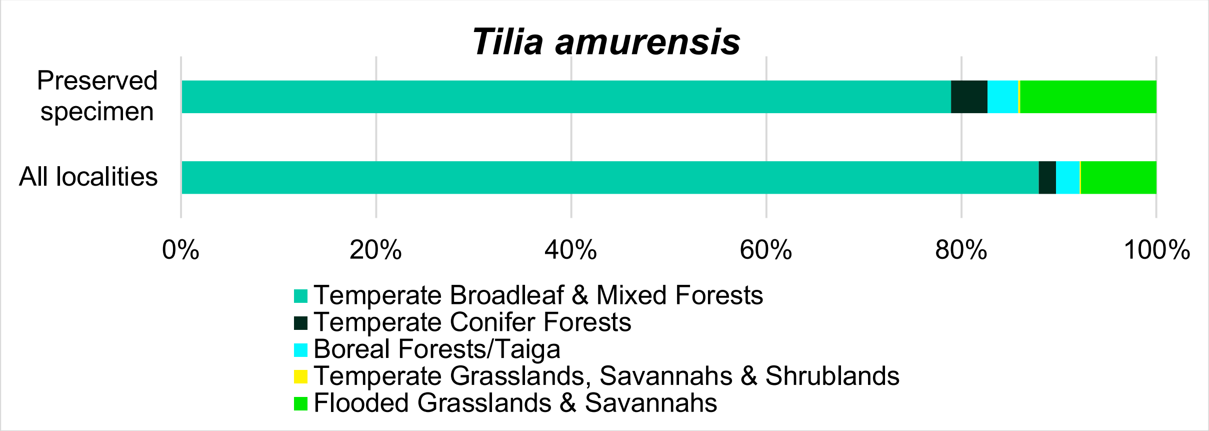

5.3.1.1.3. Climate graphs - based on 1010 *Tilia amurensis* occurrences in GBIF

5.3.1.1.3.1. MMT [°C]

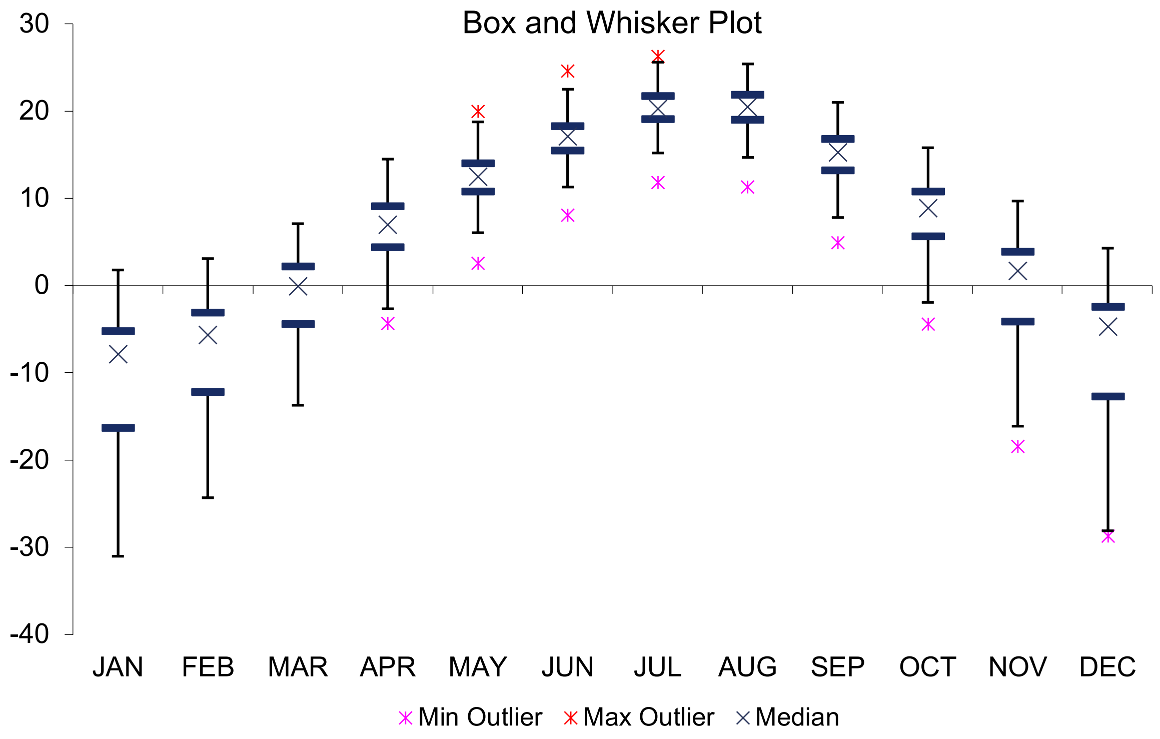

5.3.1.1.3.2. MinMT [°C]

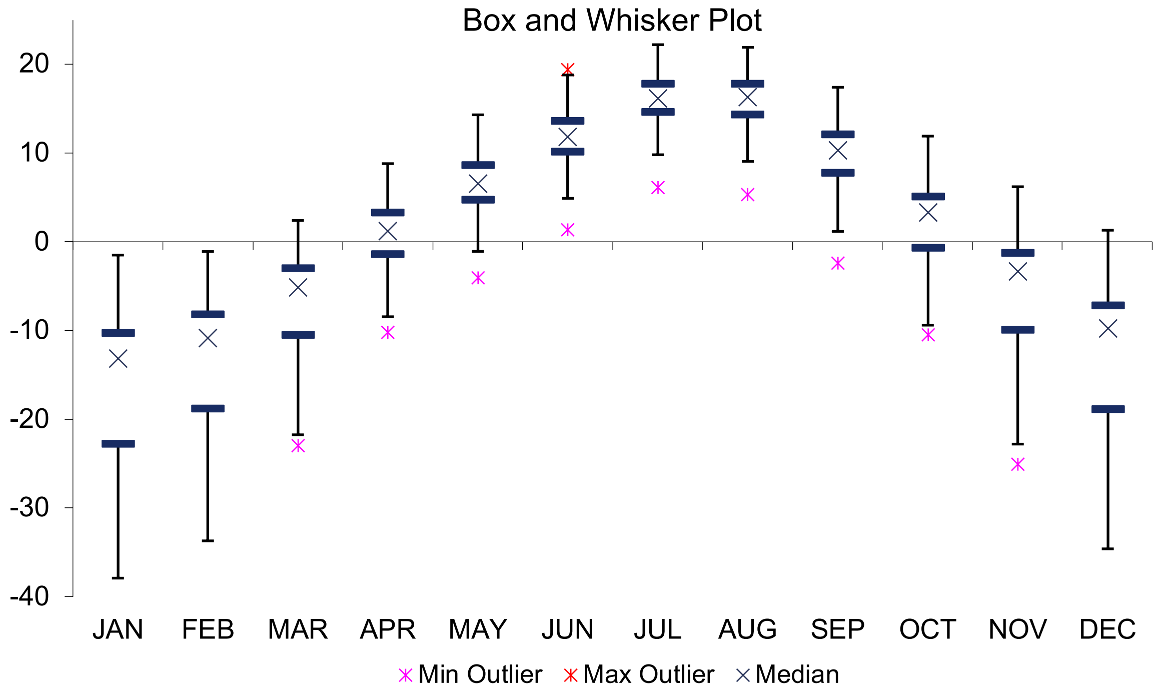

5.3.1.1.3.3. MMP [mm]

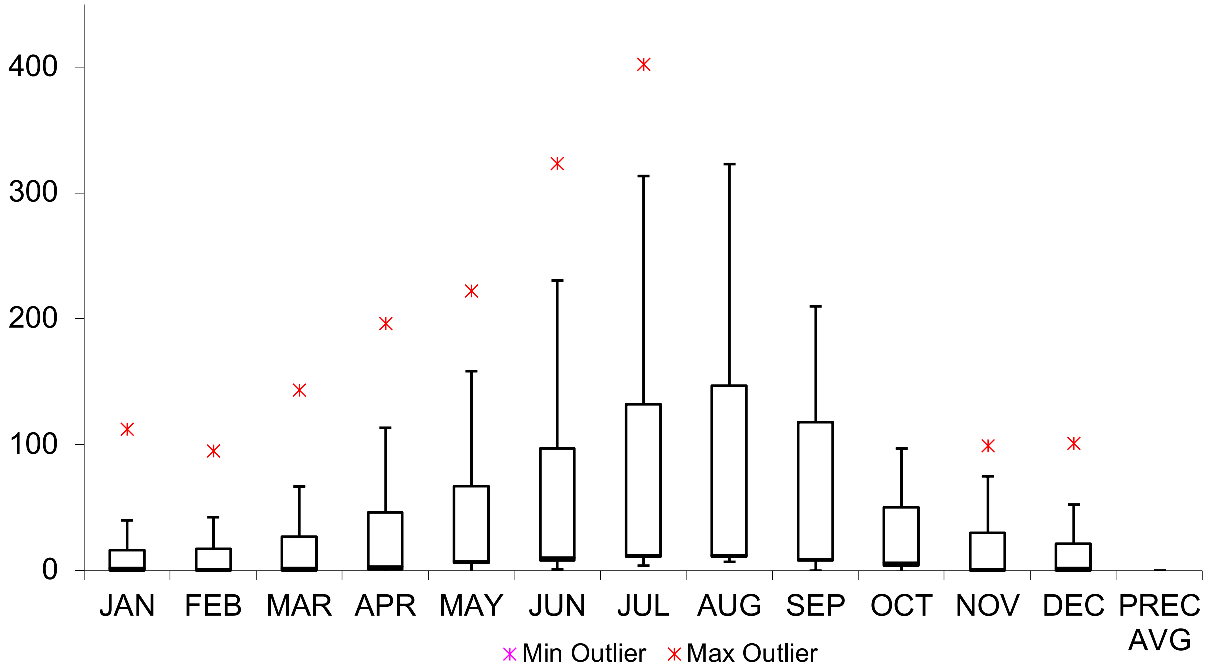

5.3.1.2. Species *Tilia japonica* Rupr., (Miq., 1867) Simonk., 1888

5.3.1.2.1. Köppen profile, distribution, and climate map – GBIF occurrences of *Tilia japonica*; excluding duplicate occurrences (n = 1008), herbarium specimens (n = 746).

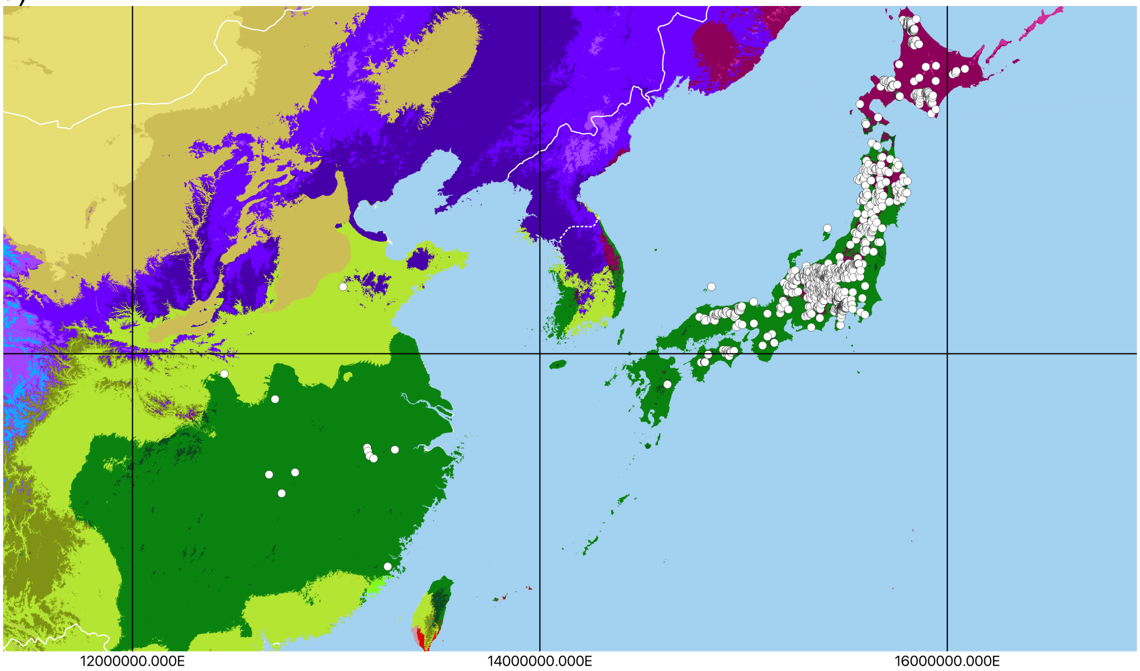

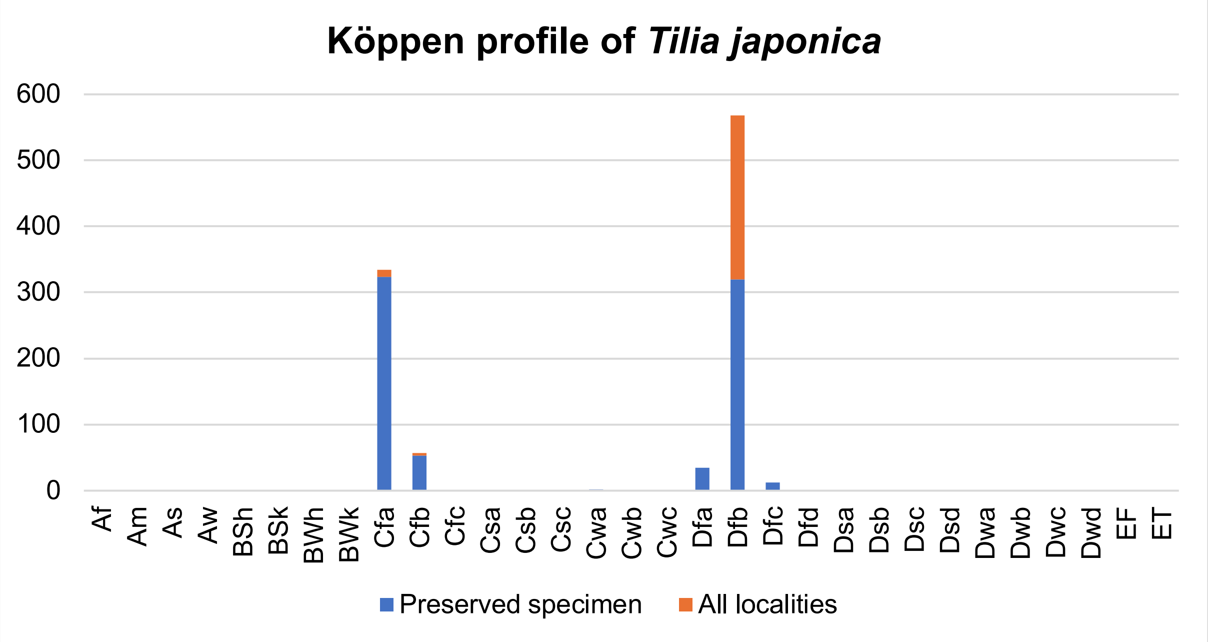

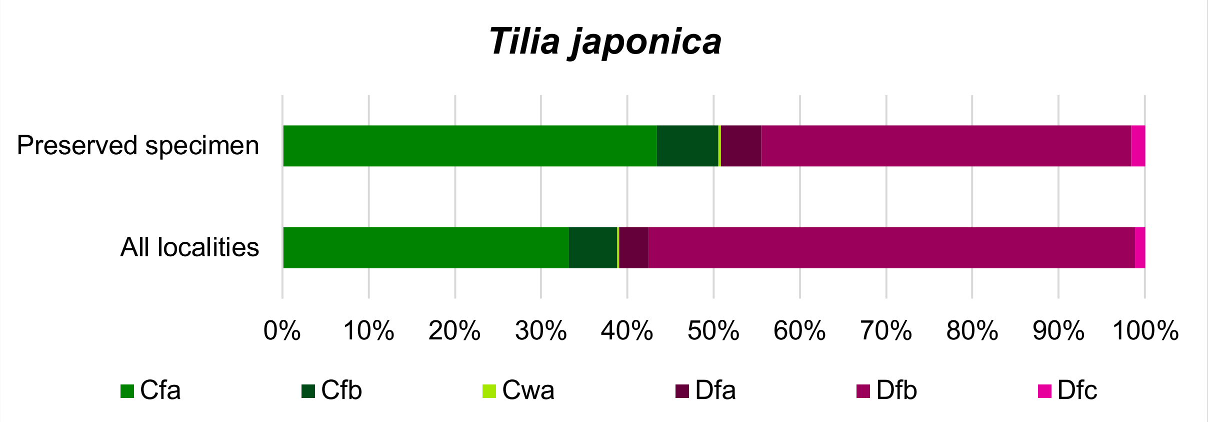

5.3.1.2.2. Biome profile, distribution, and biome map – GBIF occurrences of *Tilia japonica*; excluding duplicate occurrences (n = 1003), herbarium specimens (n = 742).

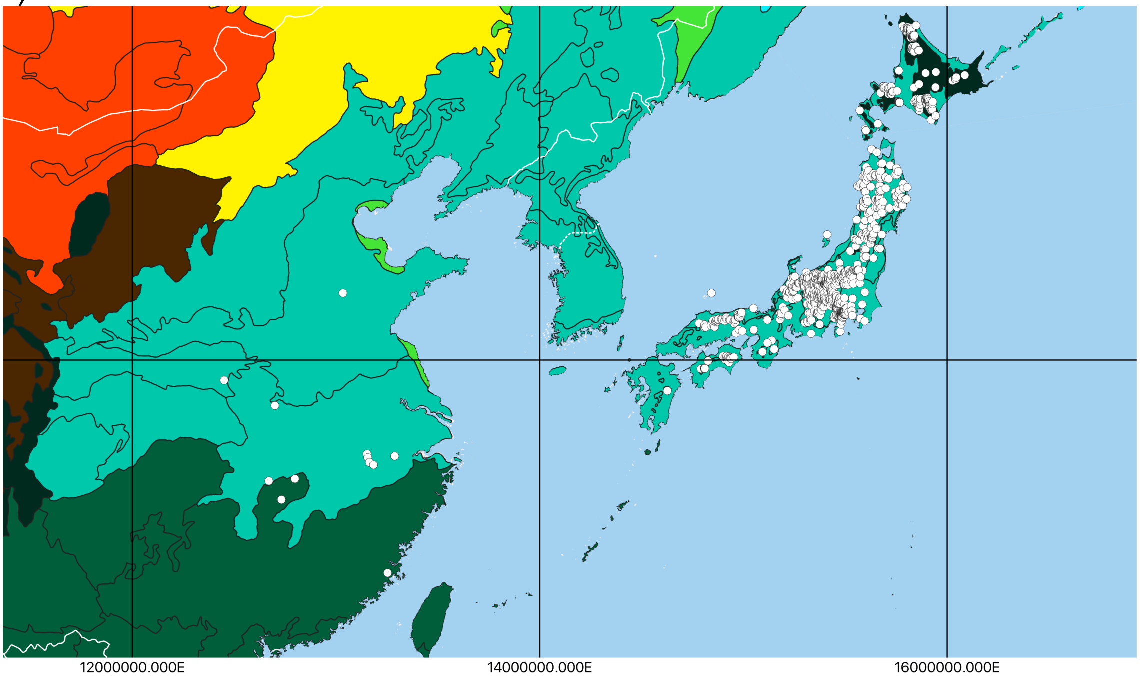

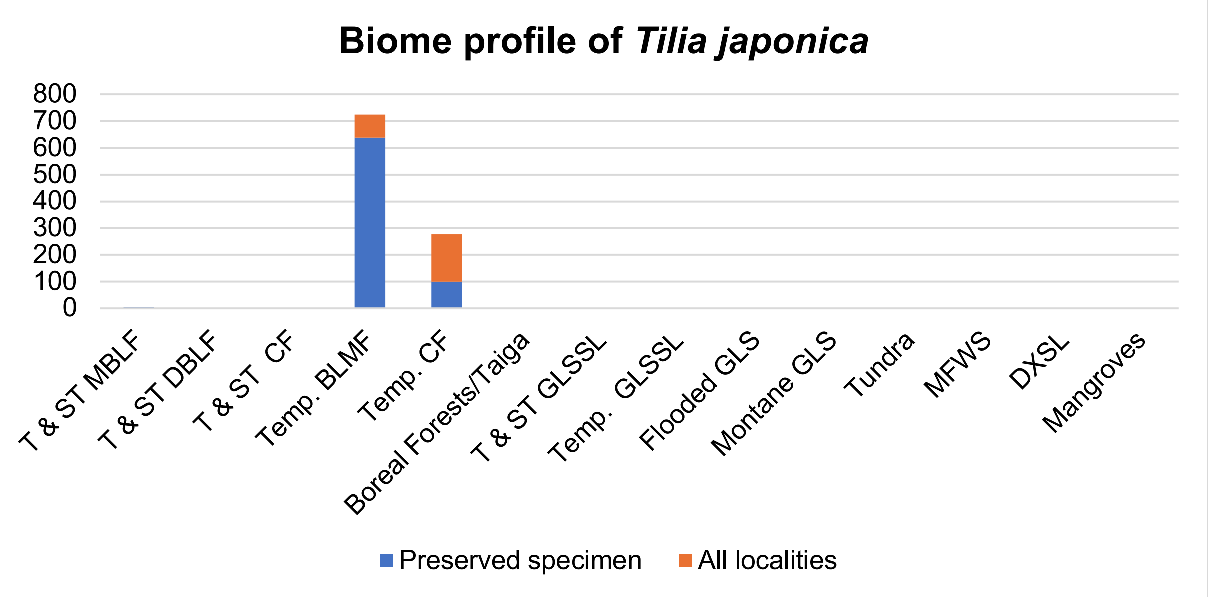

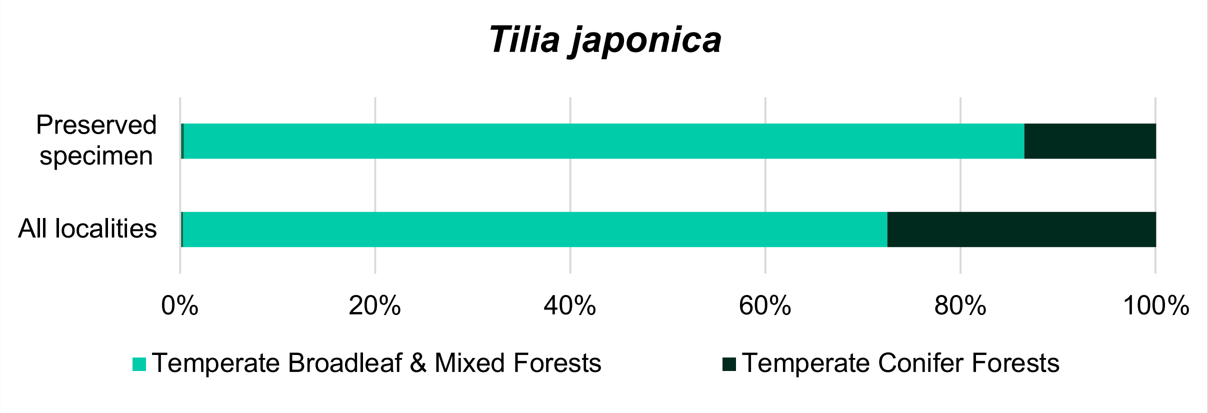

5.3.1.2.3. Climate graphs - based on 1005 *Tilia japonica* occurrences in GBIF

5.3.1.2.3.1. MMT [°C]

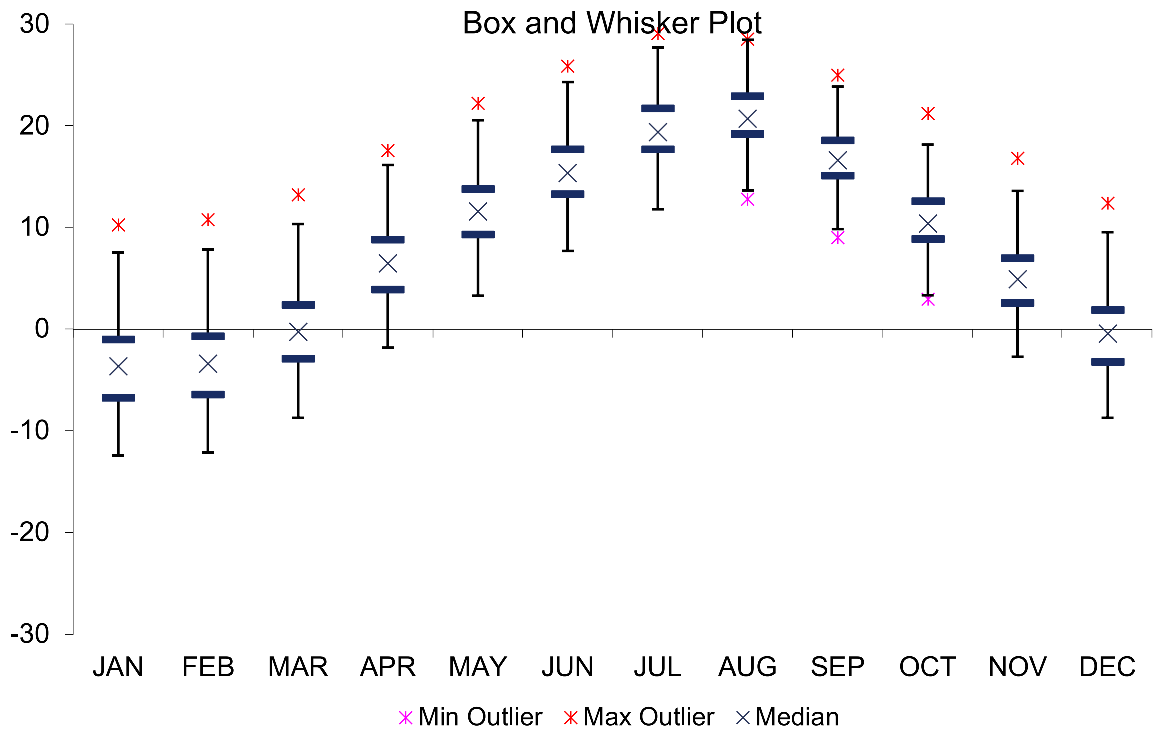

5.3.1.2.3.2. MinMT [°C]

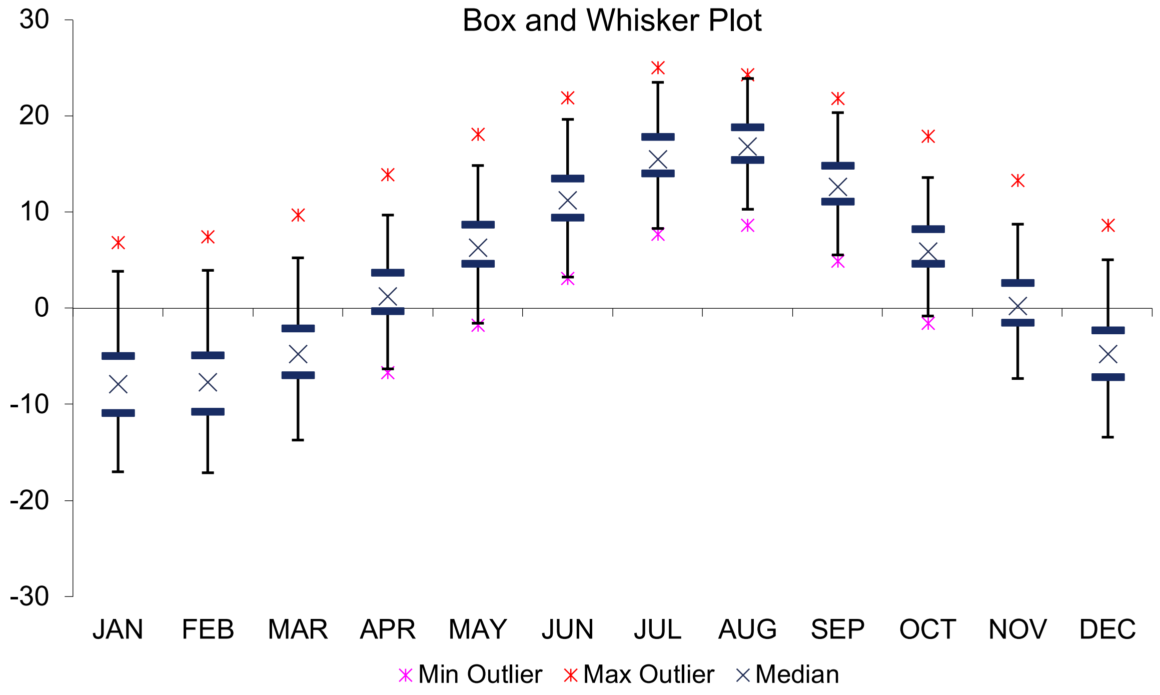

5.3.1.2.3.3. MMP [mm]

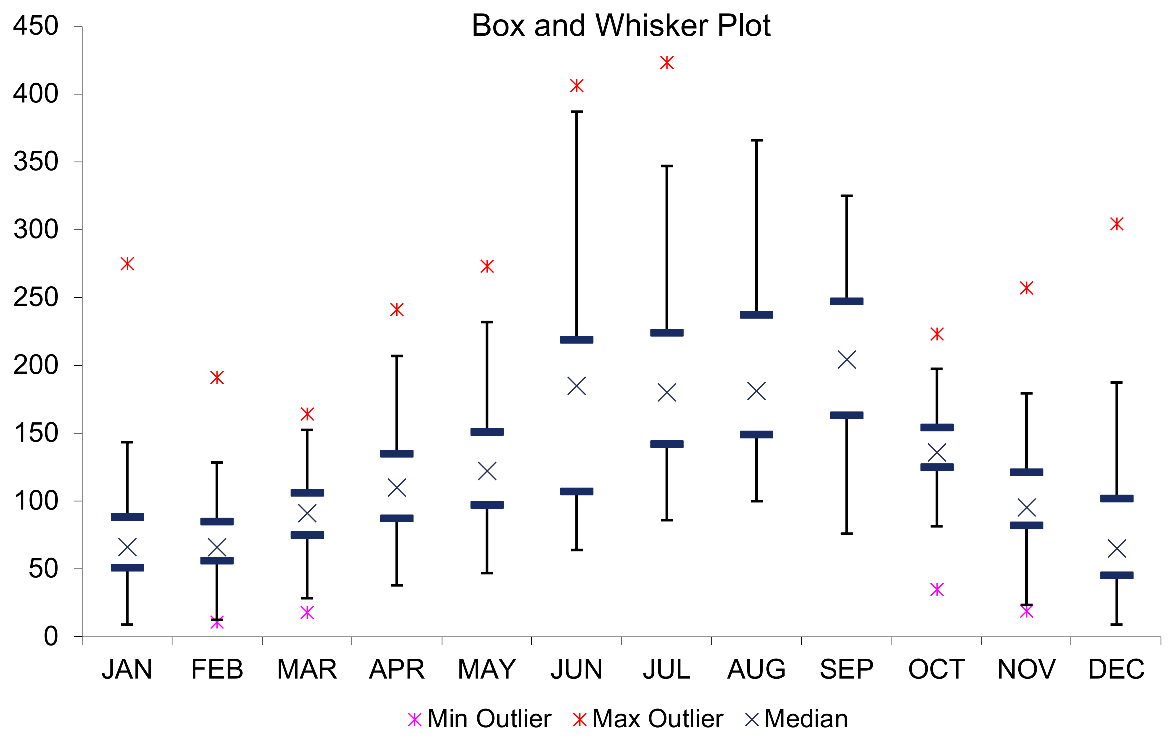

5.3.1.3. Species *Tilia mongolica* Maxim., 1880

5.3.1.3.1. Köppen profile, distribution, and climate map – GBIF occurrences of *Tilia mongolica*; excluding duplicate occurrences (n = 58), herbarium specimens (n = 50).

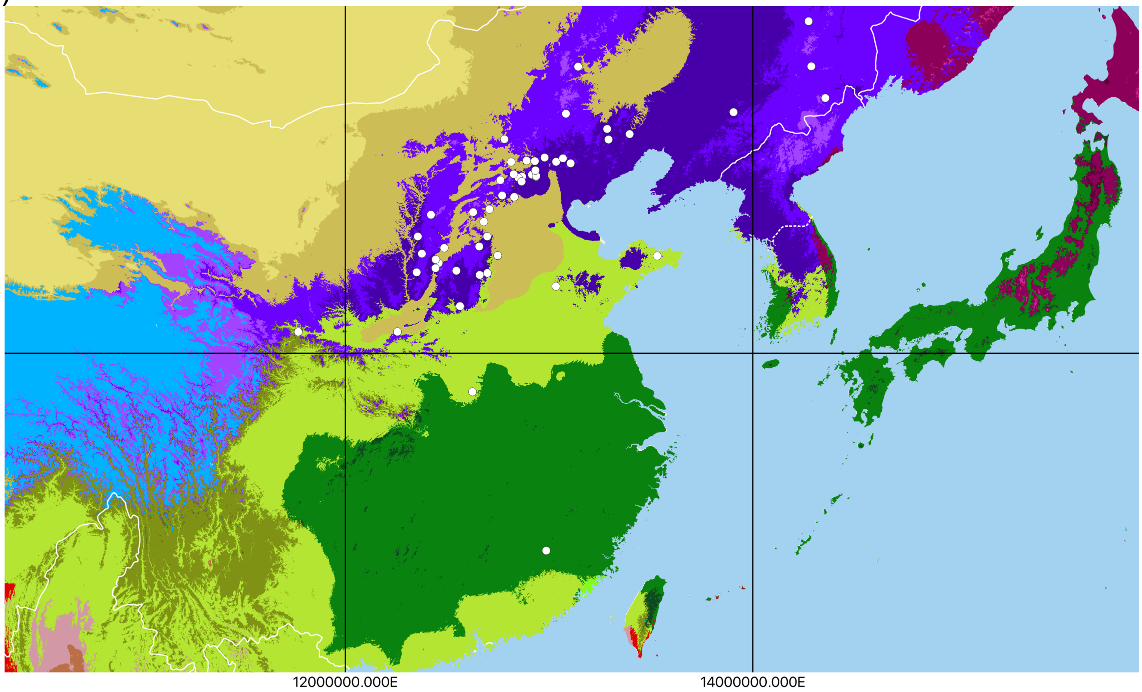

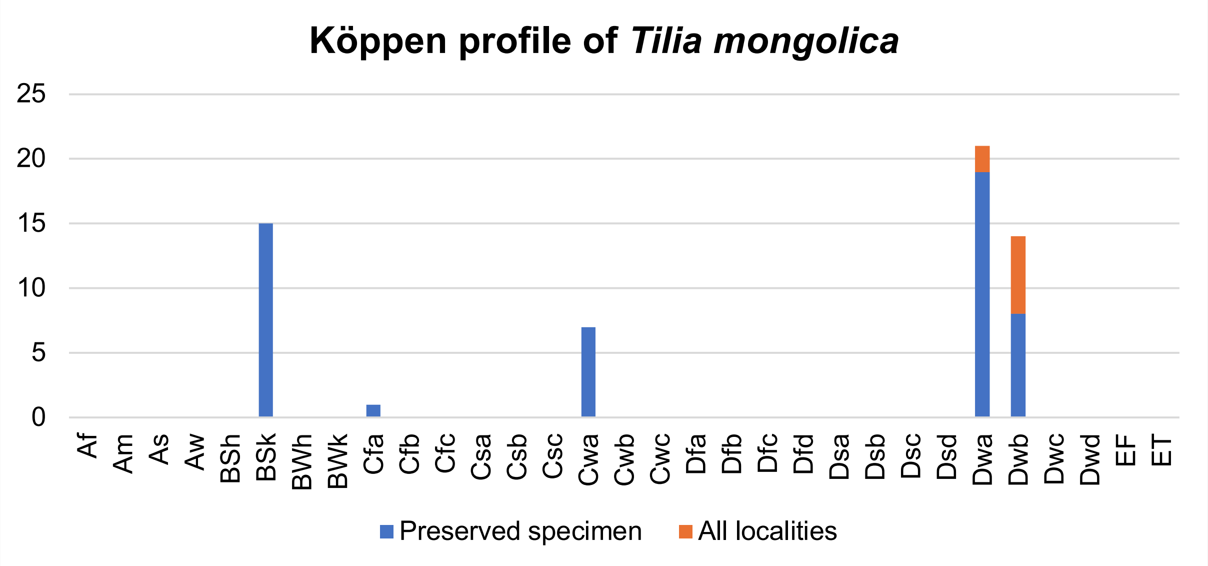

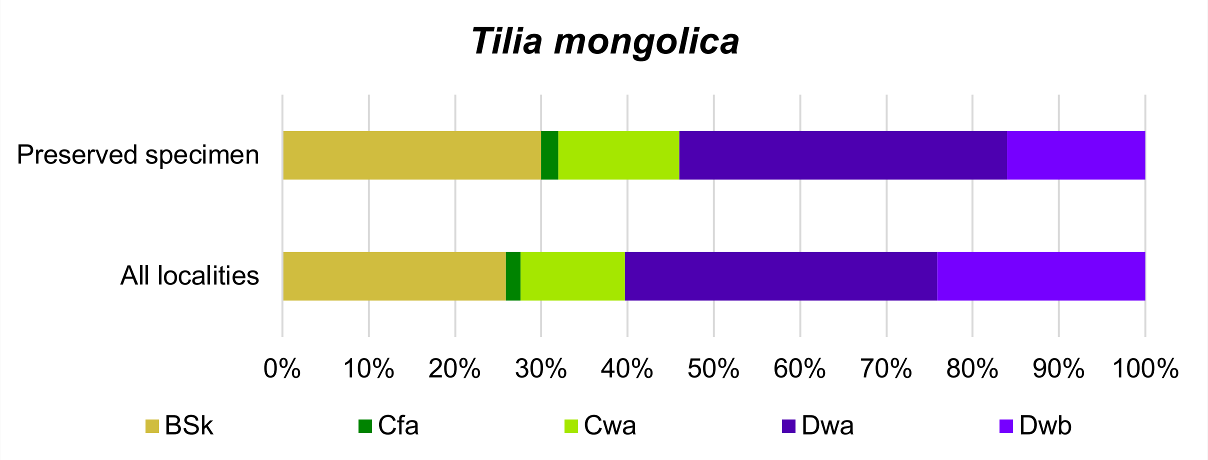

5.3.1.3.2. Biome profile, distribution, and biome map – GBIF occurrences of *Tilia mongolica*; excluding duplicate occurrences (n = 58), herbarium specimens (n = 50).

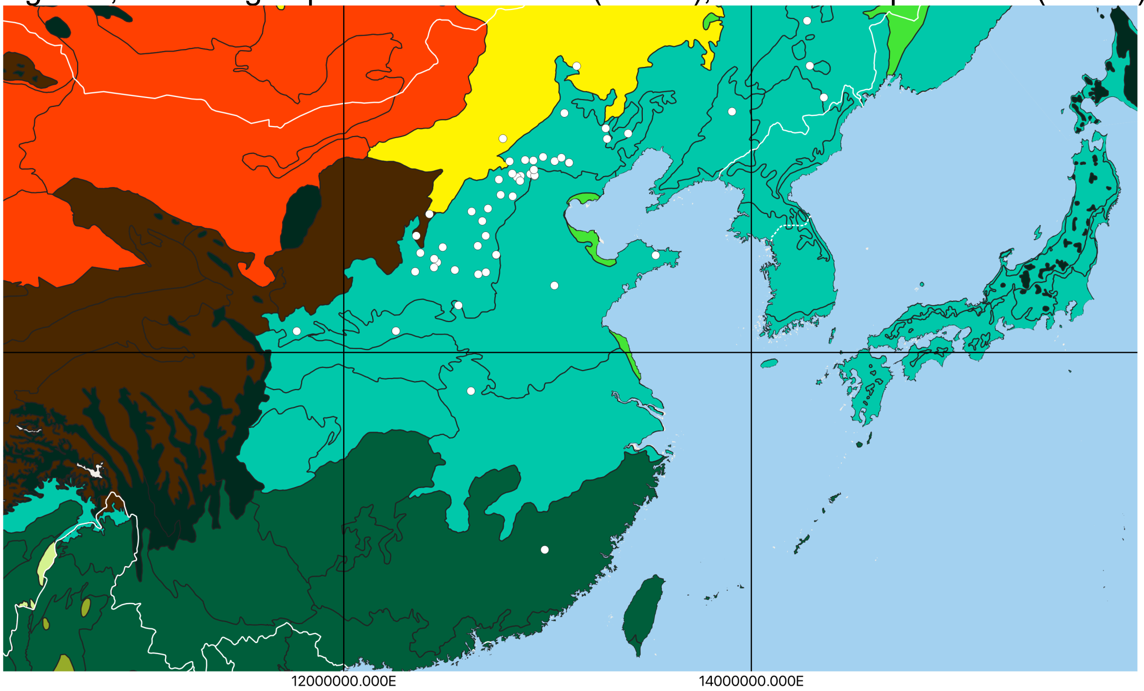

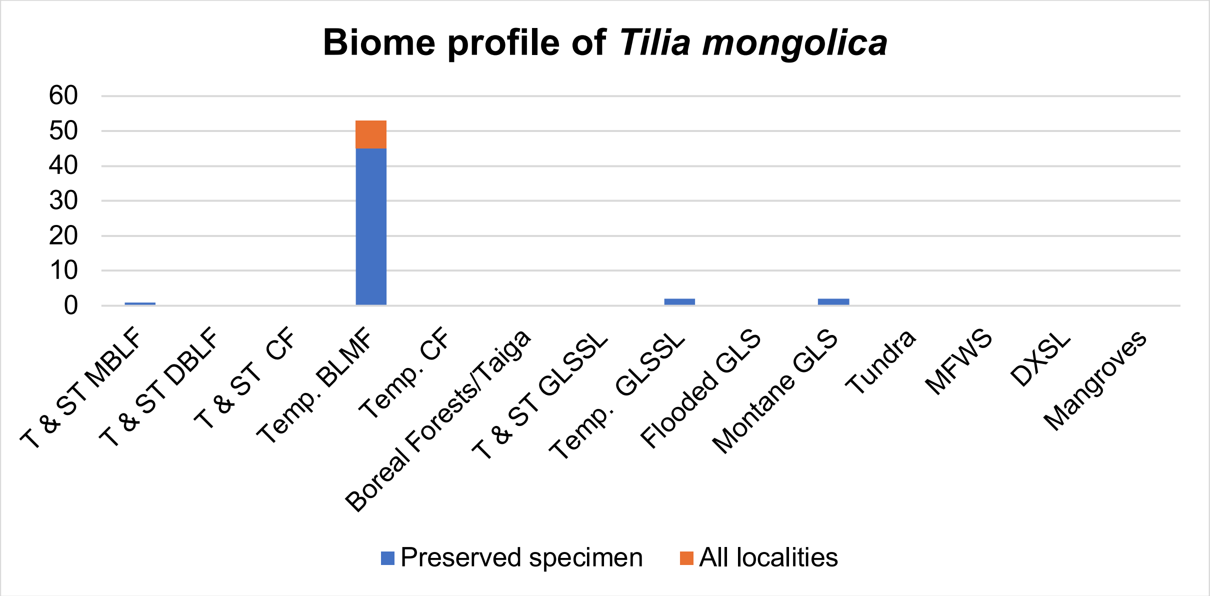

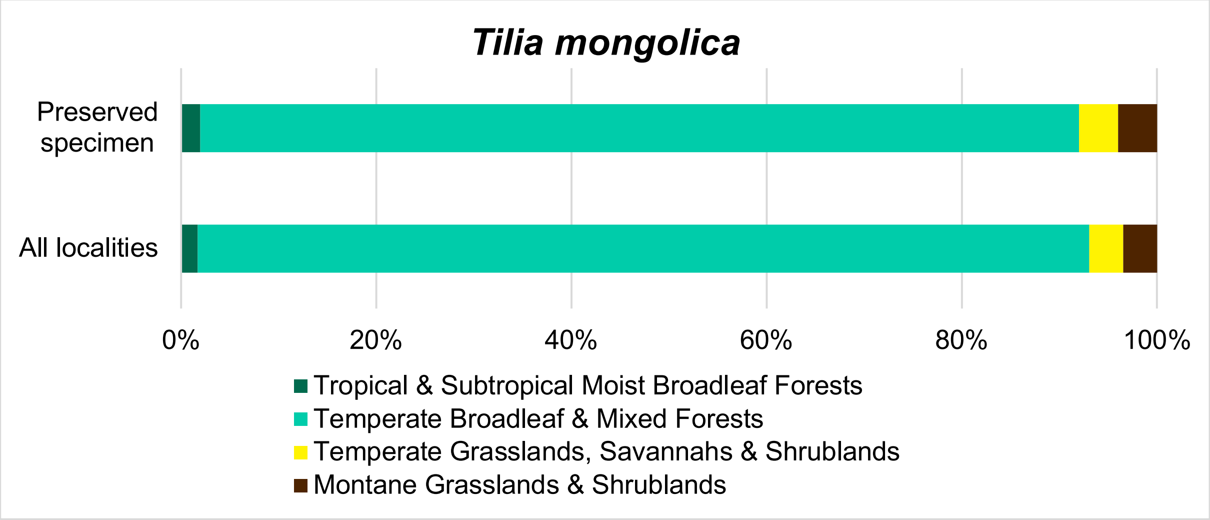

5.3.1.3.3. Climate graphs - based on 50 *Tilia mongolica* occurrences in GBIF

5.3.1.3.3.1. MMT [°C]

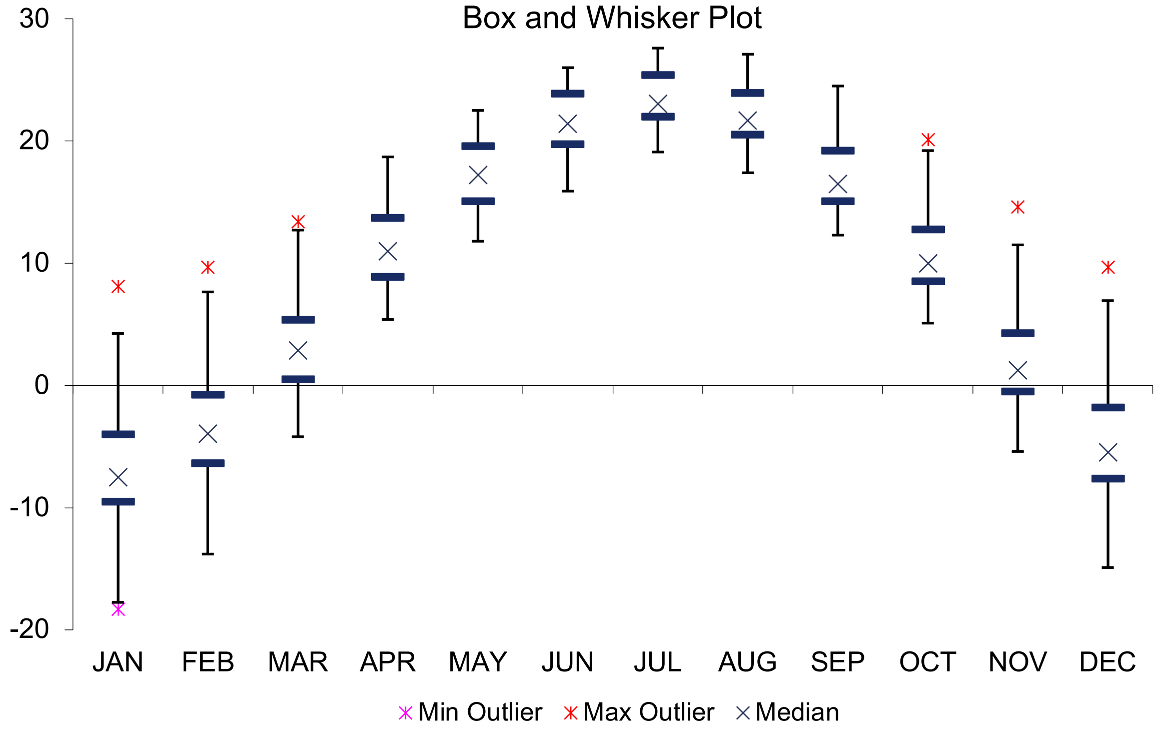

5.3.1.3.3.2. MinMT [°C]

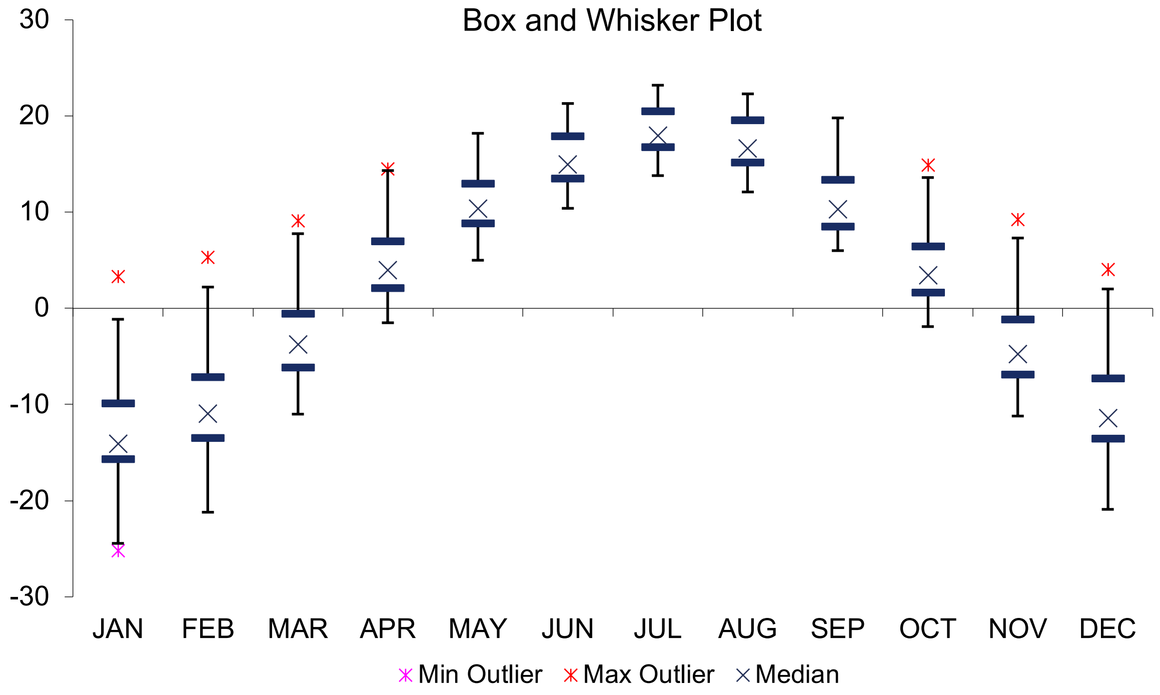

5.3.1.3.3.3. MMP [mm]

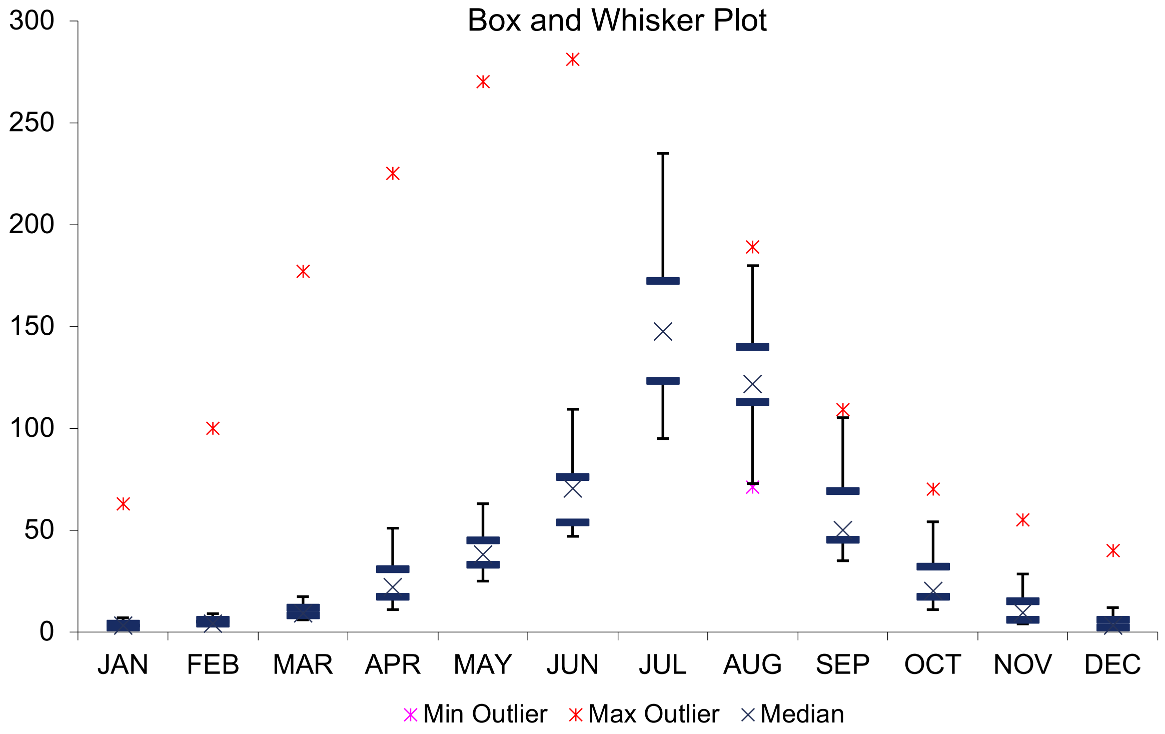

5.3.2. Tilia section Endochrysea
5.3.2.1. Species *Tilia endochrysea* Hand.-Mazz., 1926
5.3.2.1.1. Köppen profile, distribution, and climate map – GBIF occurrences of *Tilia endochrysea*; excluding duplicate occurrences herbarium specimens (n = 47).

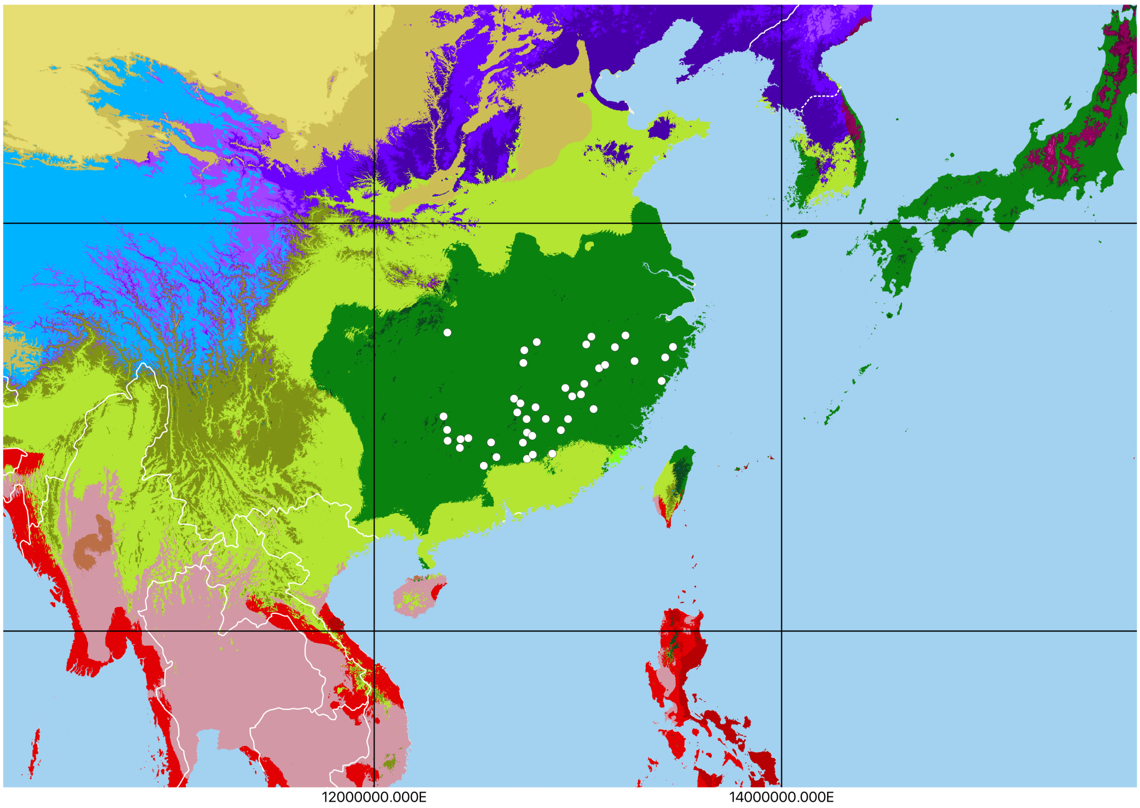

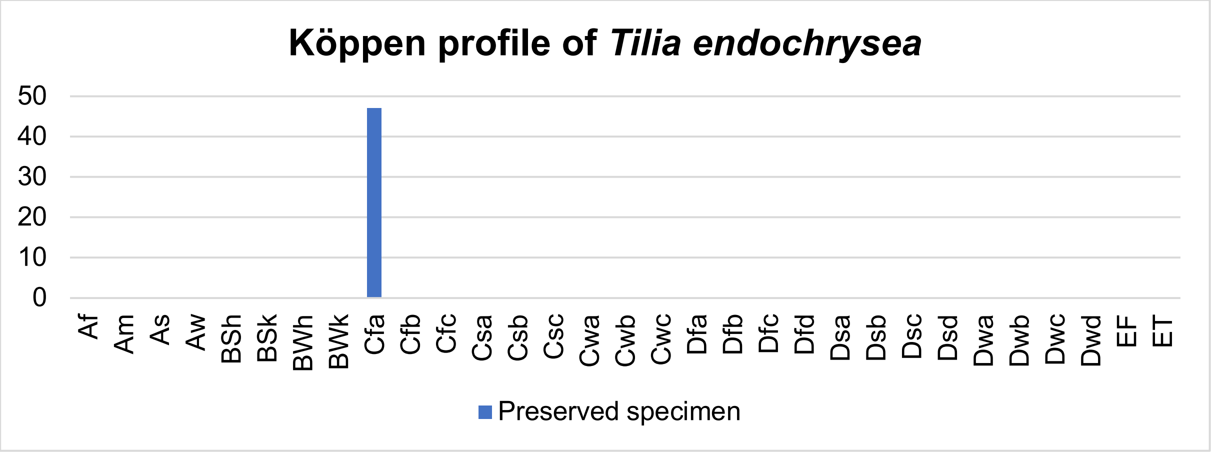

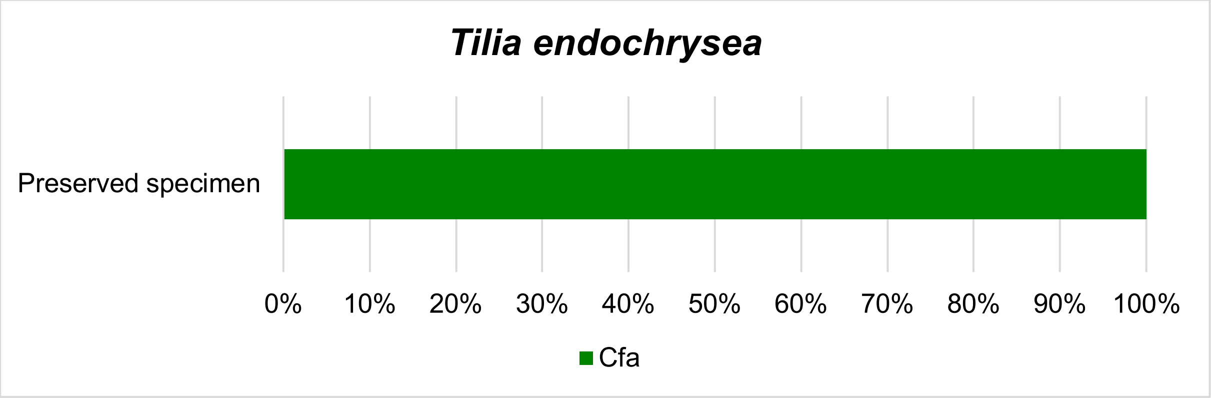

5.3.2.1.2. Biome profile, distribution, and biome map GBIF occurrences of *Tilia endochrysea*; excluding duplicate occurrences herbarium specimens (n = 47).

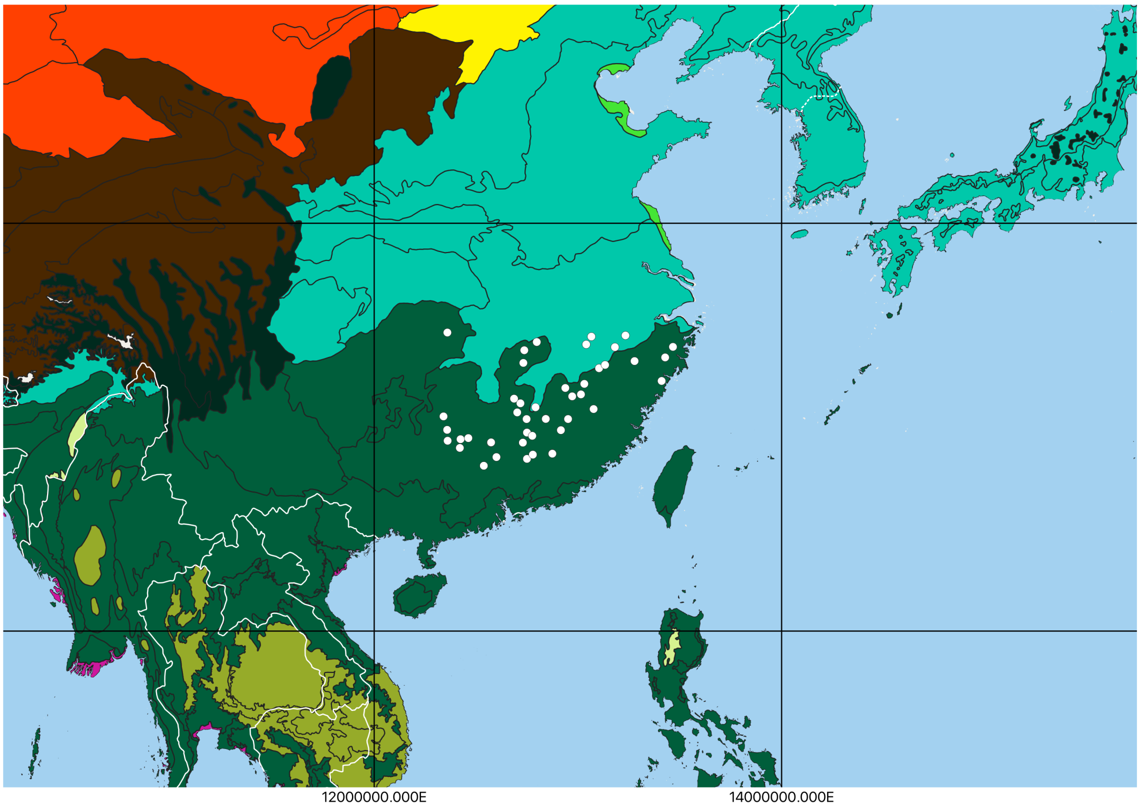

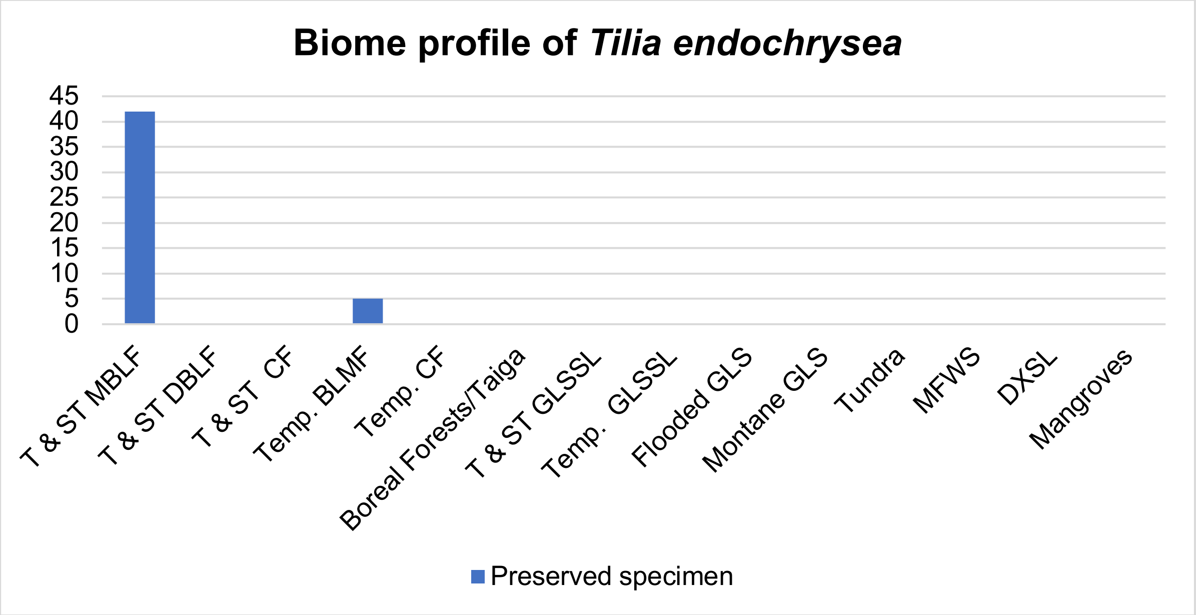

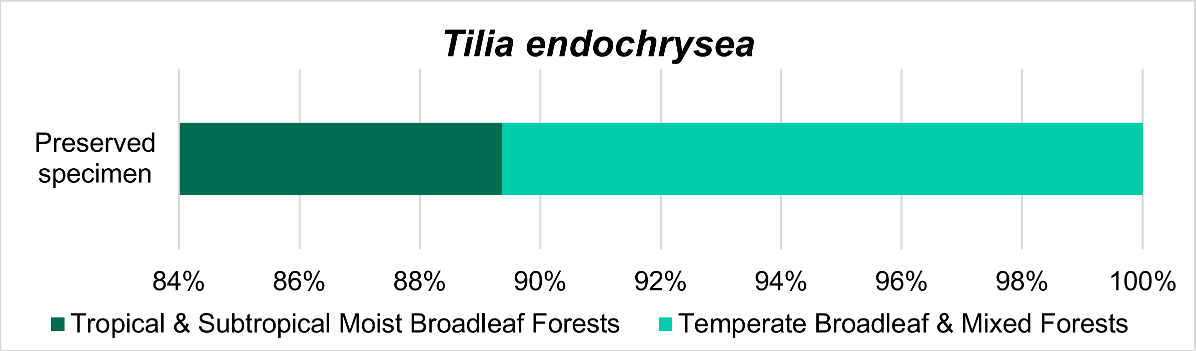

5.3.2.1.3. Climate graphs - based on 1010 *Tilia amurensis* occurrences in GBIF

5.3.2.1.3.1. MMT [°C]

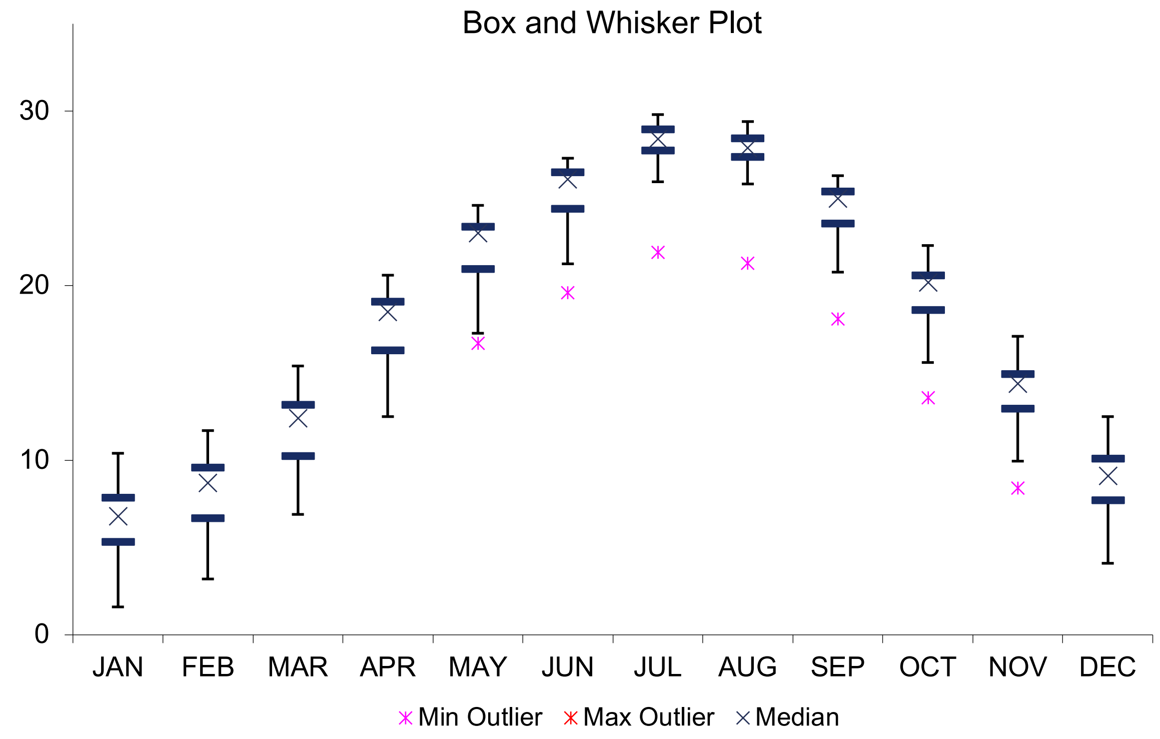

5.3.2.1.3.2. MinMT [°C]

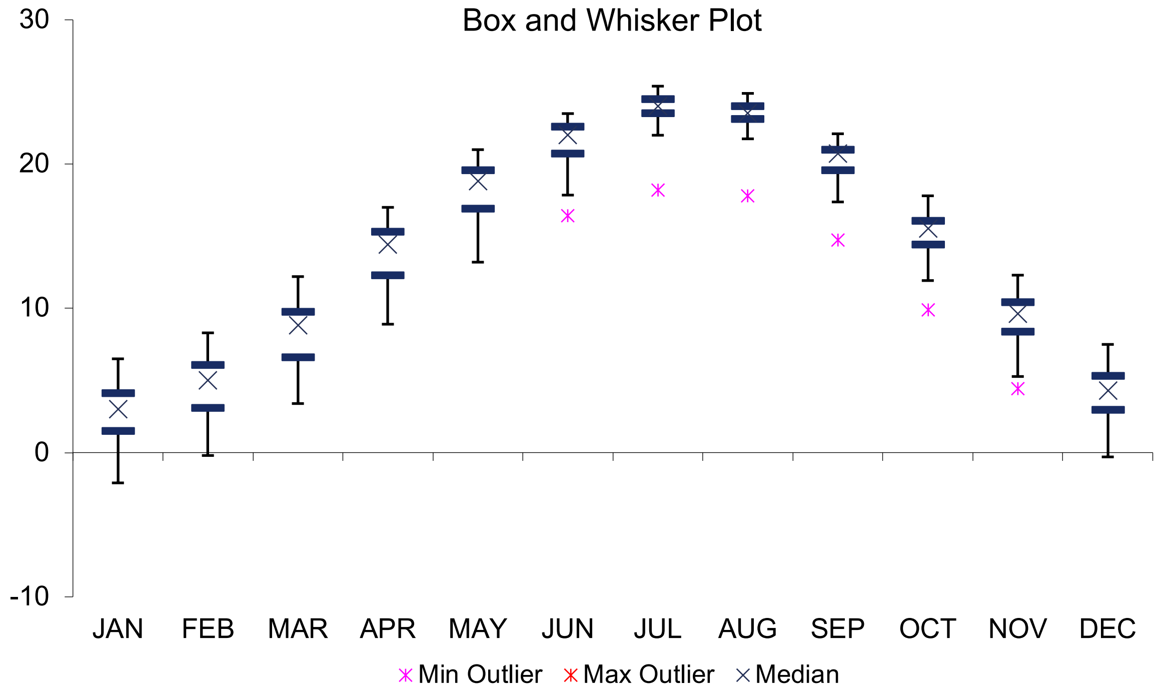

5.3.2.1.3.3. MMP [mm]

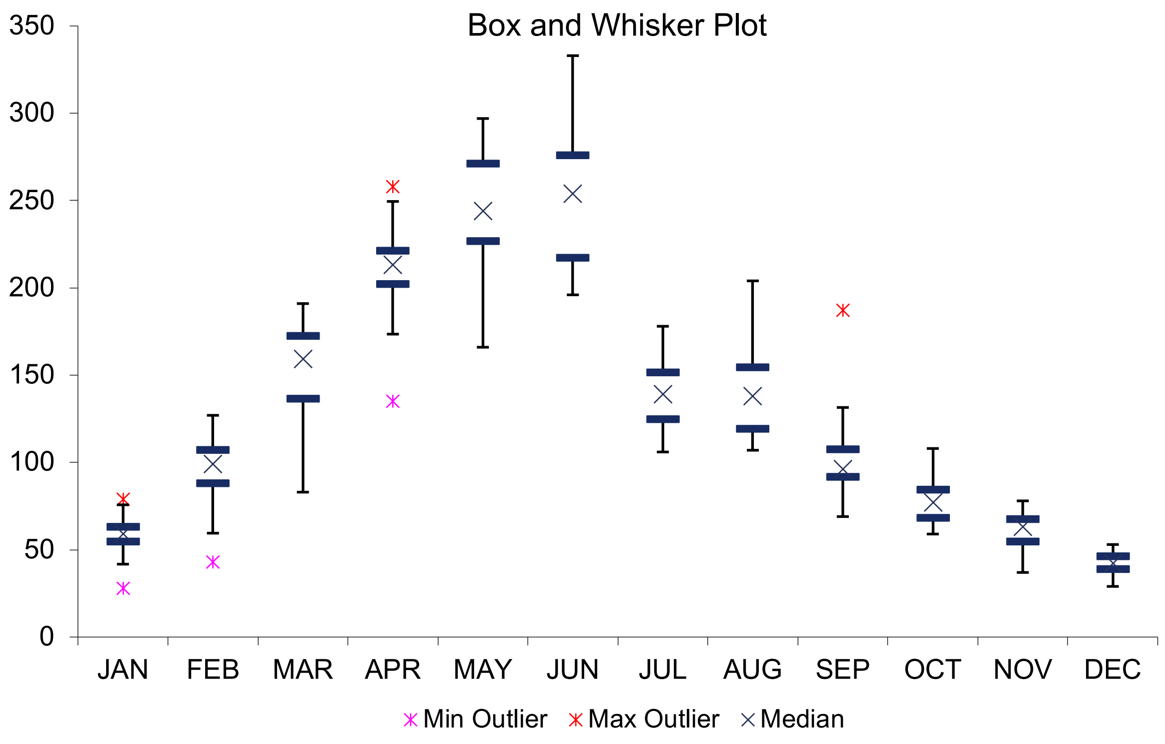

5.3.3. Tilia section Henryana

5.3.3.1. Species *Tilia henryana* Szyszył., 1890

5.3.3.1.1. Köppen profile, distribution, and climate map – GBIF occurrences of *Tilia henryana*; excluding duplicate occurrences herbarium specimens (n = 36).

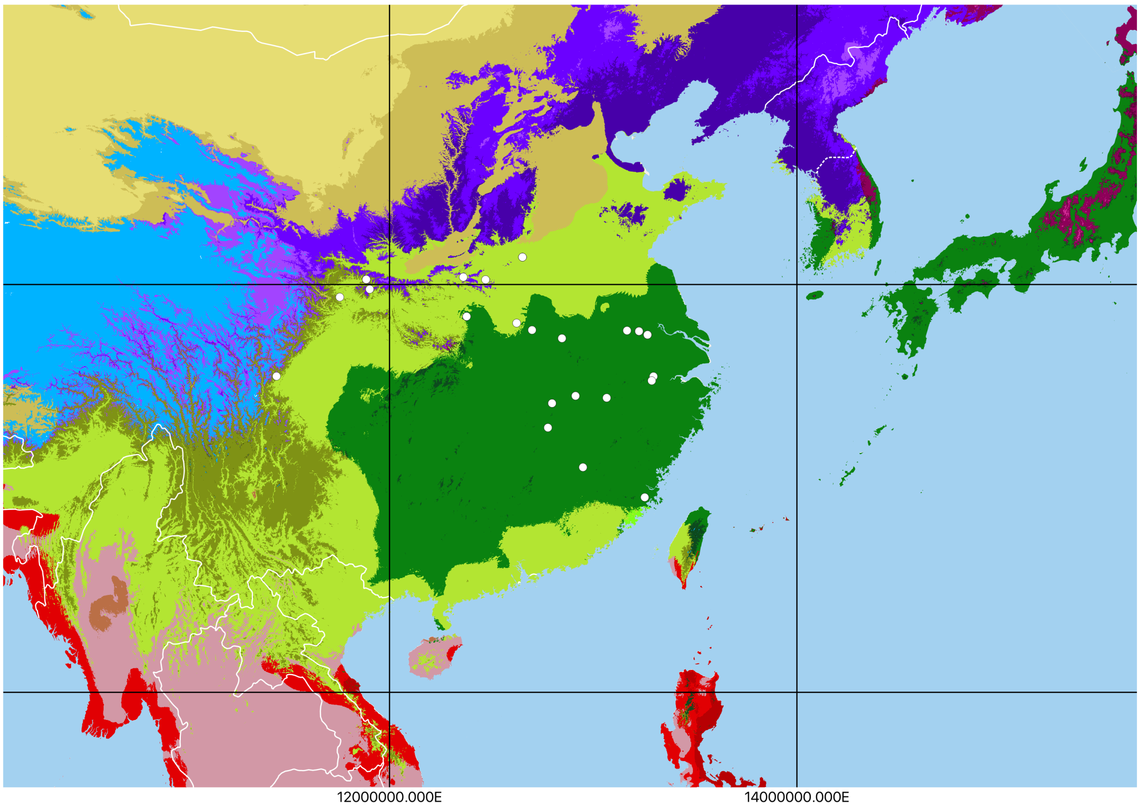

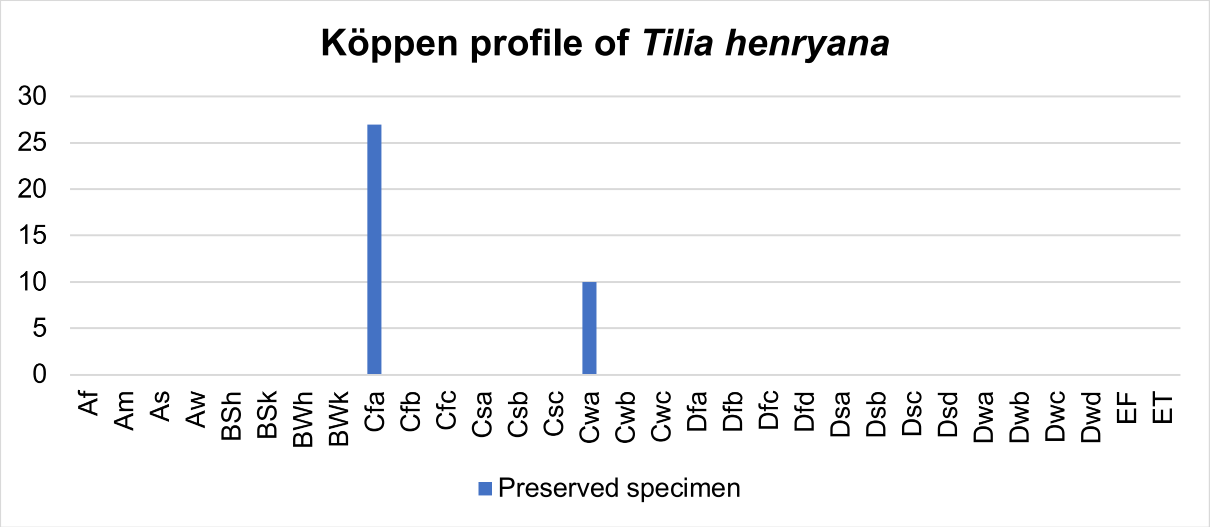

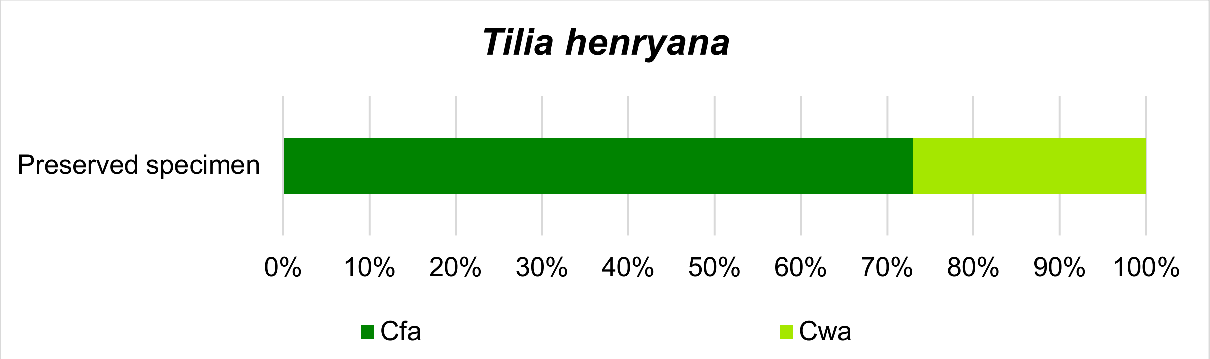

5.3.3.1.2. Biome profile, distribution, and biome map – GBIF occurrences of *Tilia henryana*; excluding duplicate occurrences herbarium specimens (n = 36).

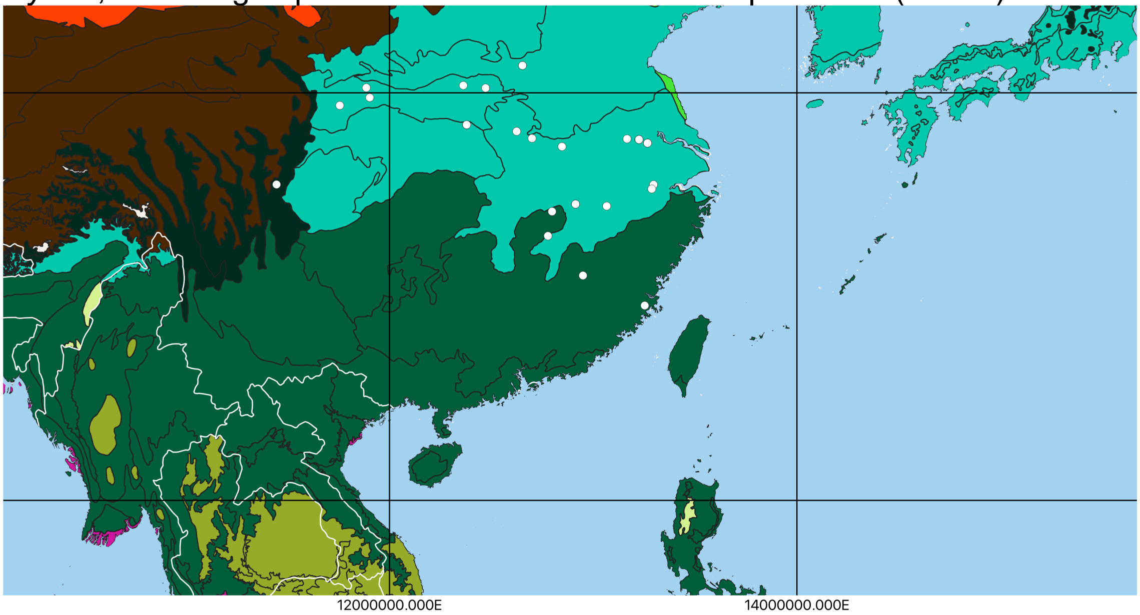

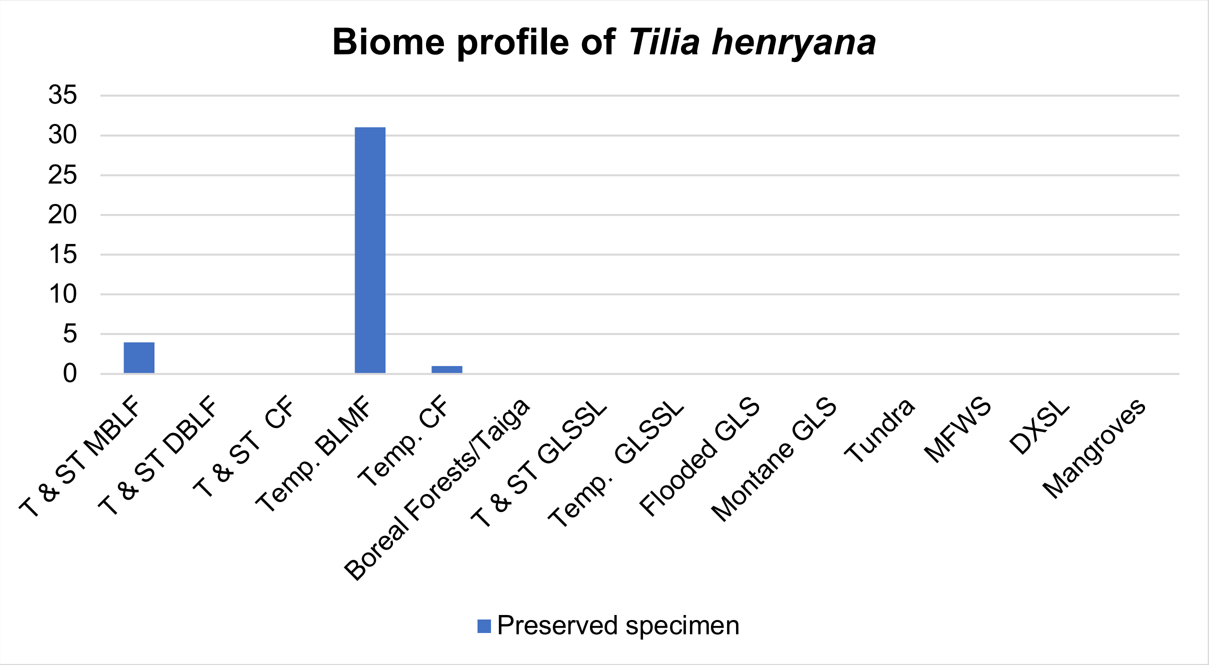

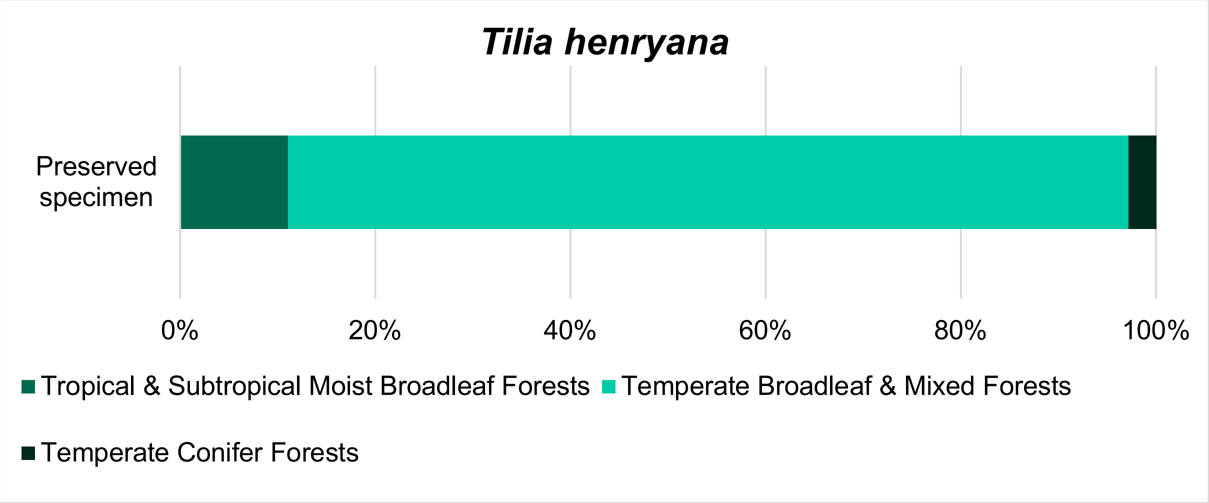

5.3.3.1.3. Climate graphs - based on 37 *Tilia henryana* occurrences in GBIF

5.3.3.1.3.1. MMT [°C]

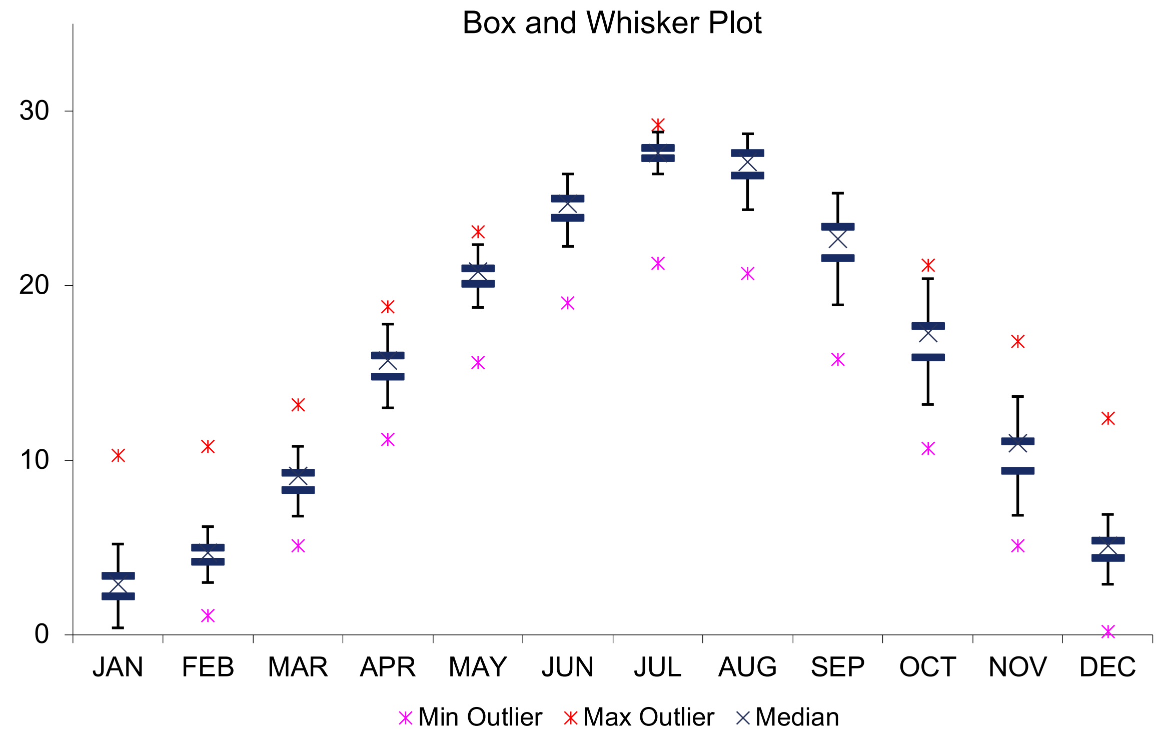

5.3.3.1.3.2. MinMT [°C]

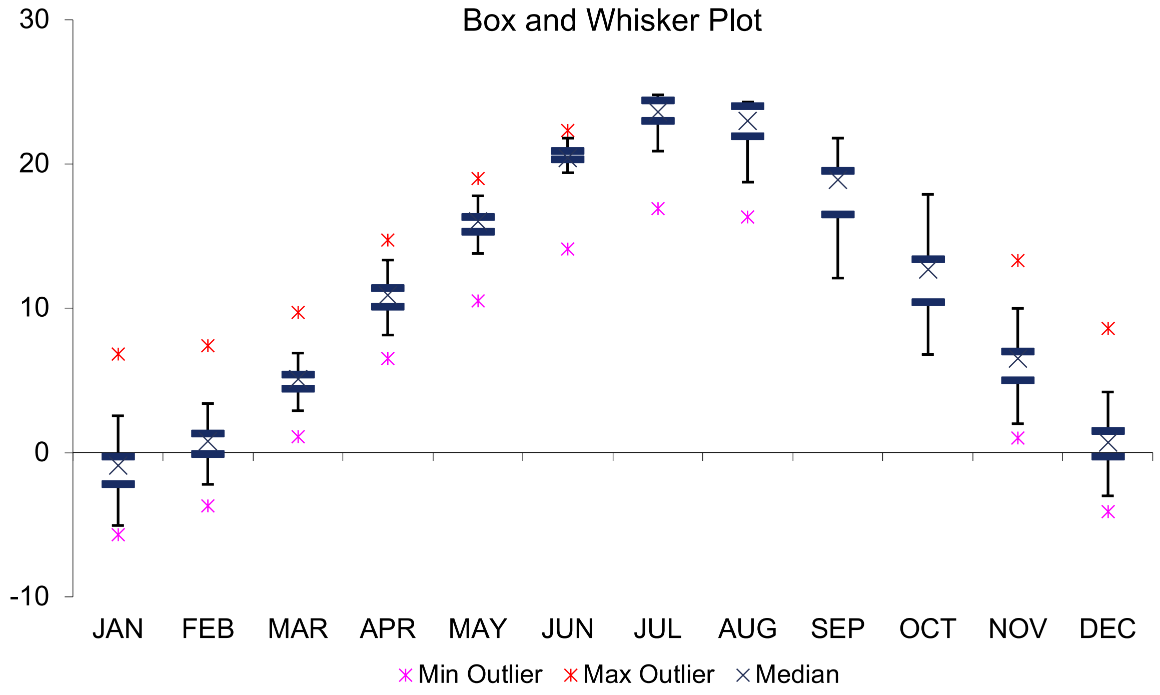

5.3.3.1.3.3. MMP [mm]

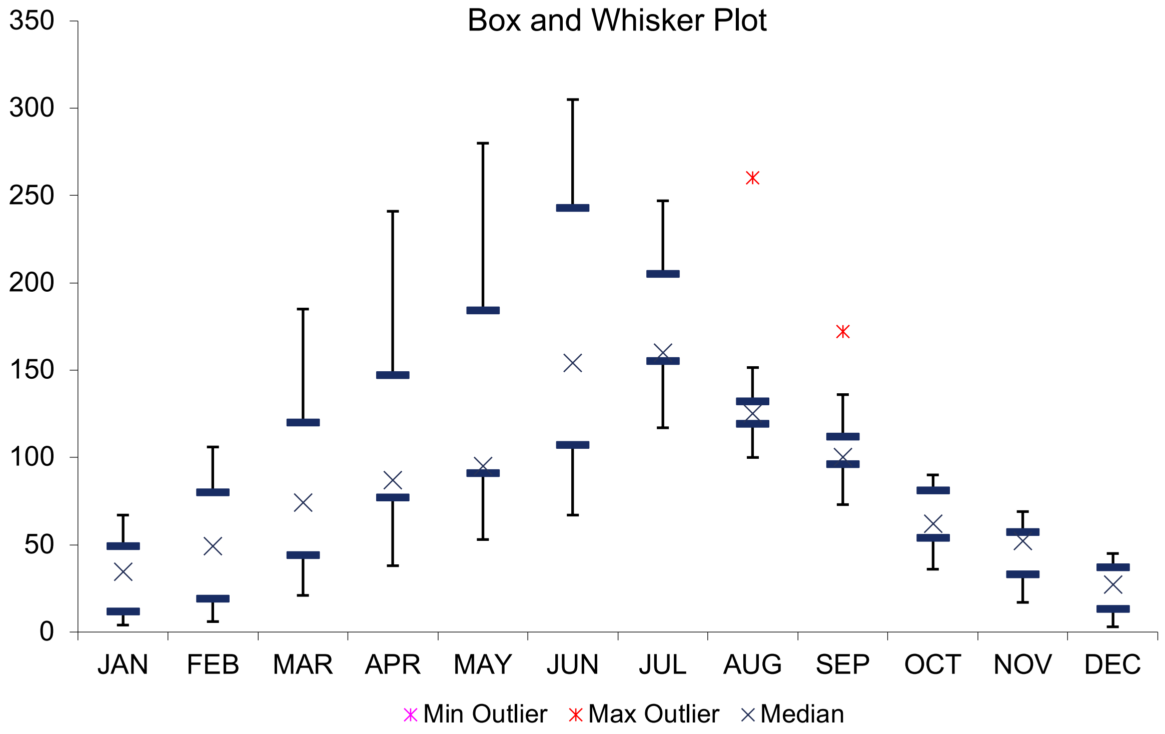

5.3.4. Tilia section Astrophilyra
5.3.4.1. Species *Tilia callidonta* Hung T.Chang, 1982
5.3.4.1.1. Köppen profile, distribution, and climate map – GBIF occurrences of *Tilia callidonta*; excluding duplicate occurrences herbarium specimens (n = 3).

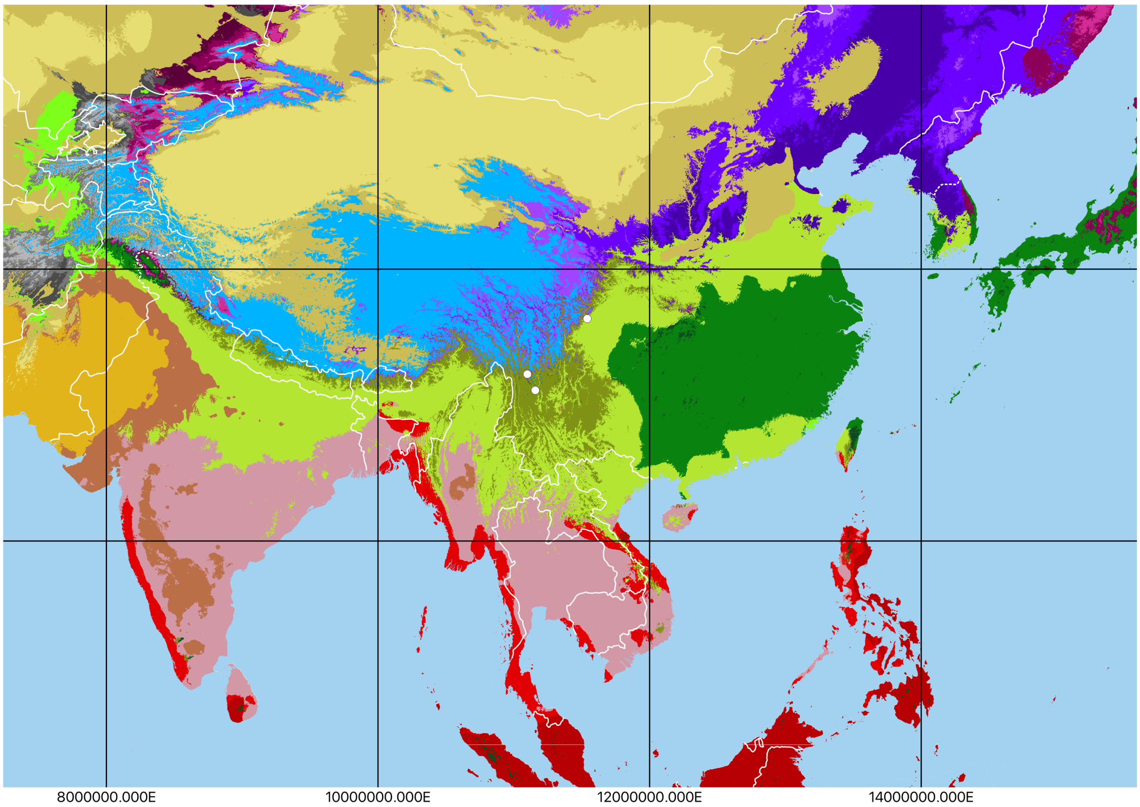

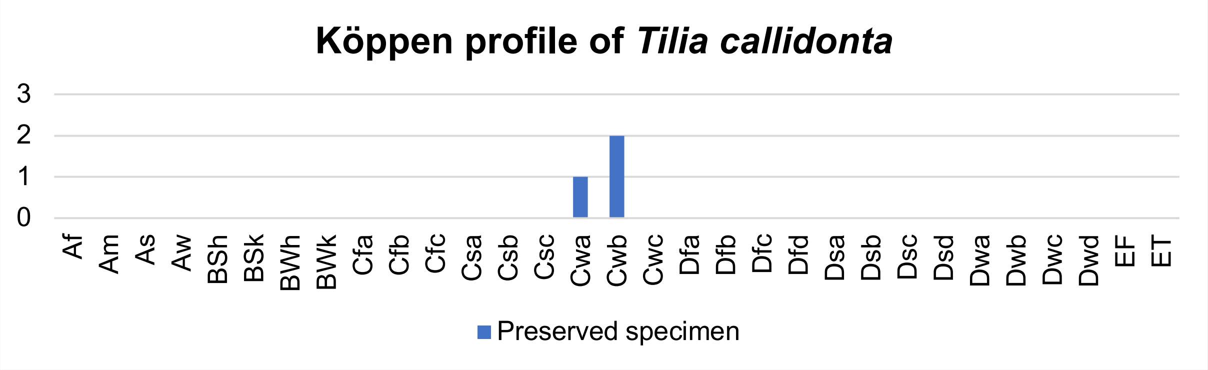

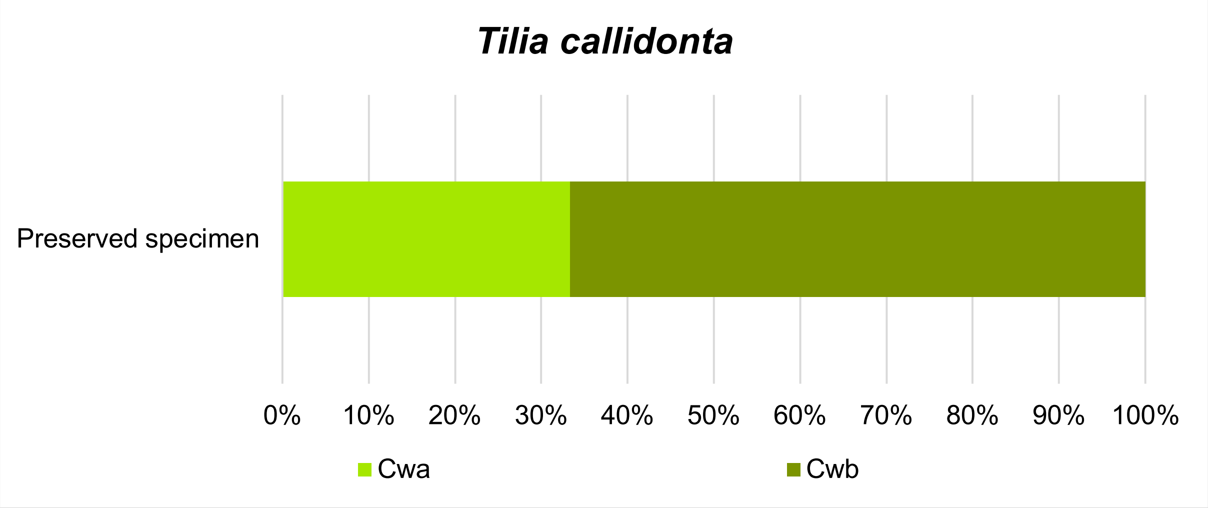

5.3.4.1.2. Biome profile, distribution, and biome map – GBIF occurrences of *Tilia callidonta*; excluding duplicate occurrences herbarium specimens (n = 3).

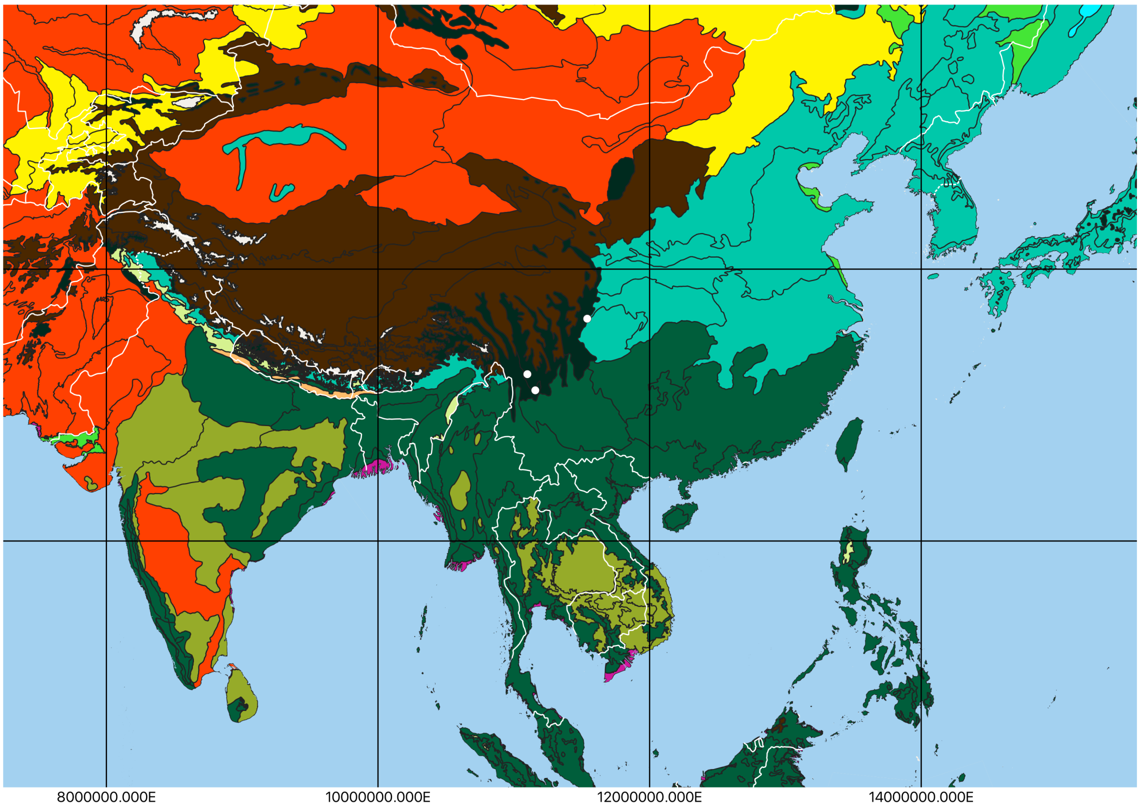

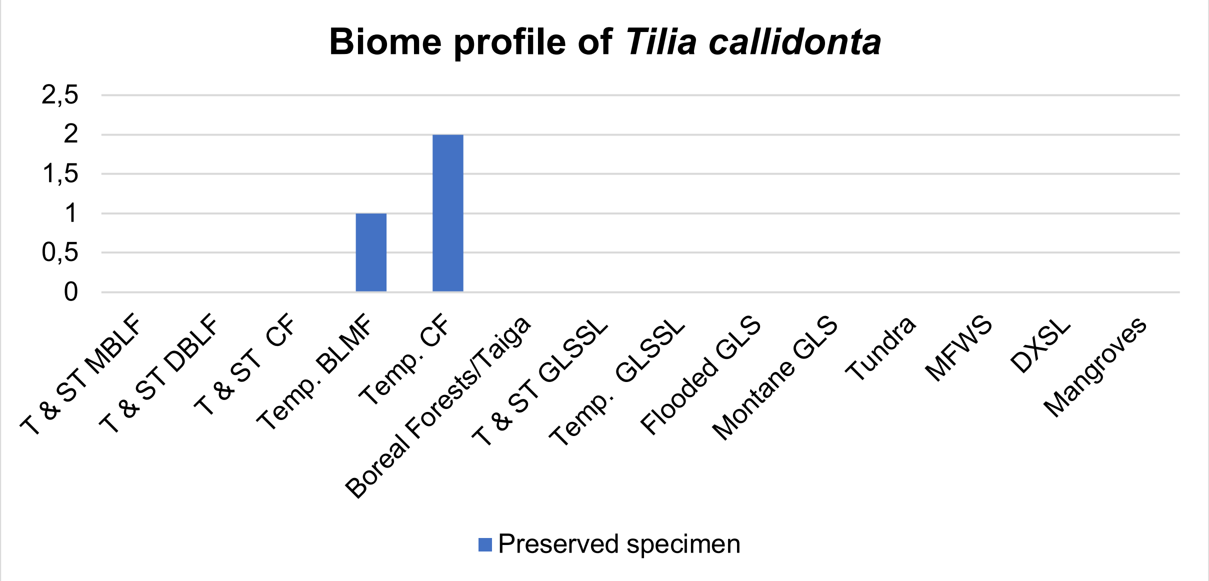

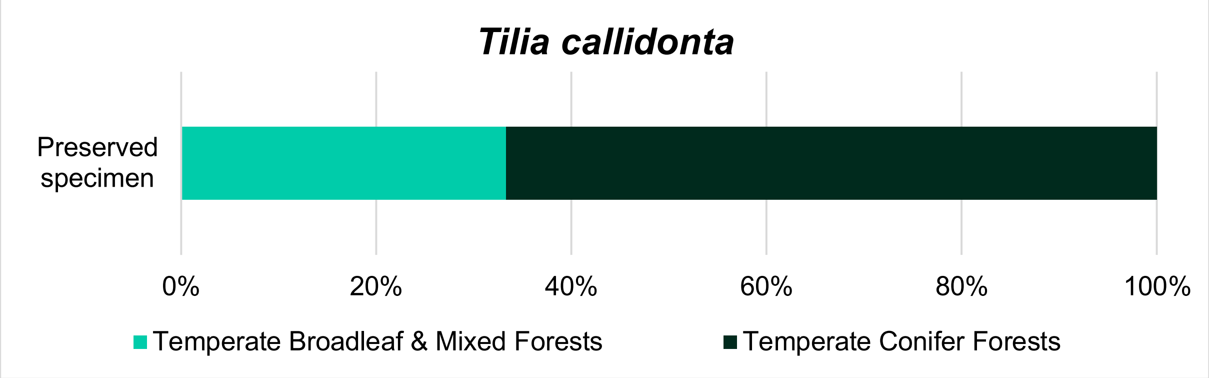

5.3.4.1.3. Climate graphs - based on 3 *Tilia callidonta* occurrences in GBIF

5.3.4.1.3.1. MMT [°C]

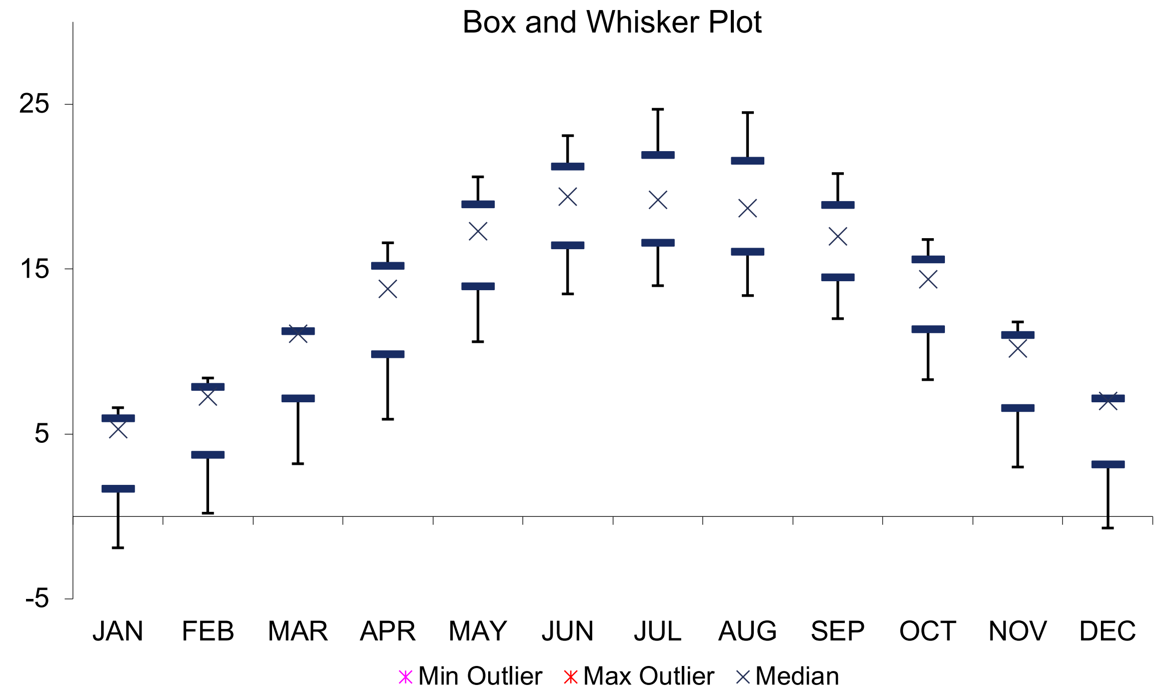

5.3.4.1.3.2. MinMT [°C]

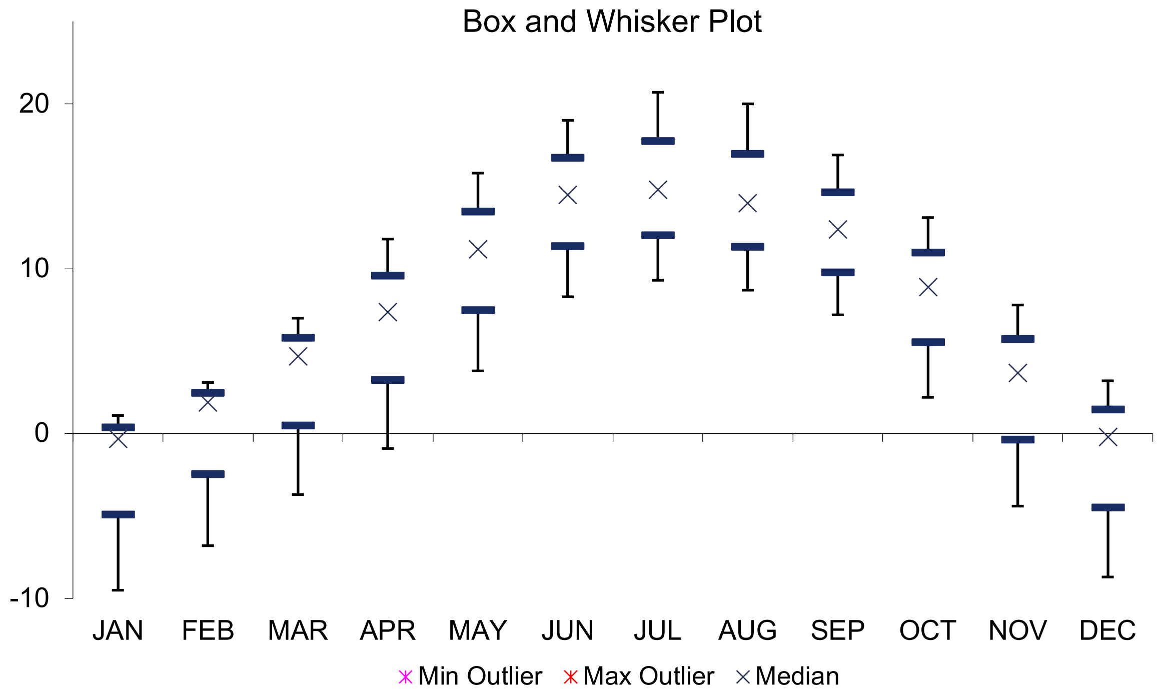

5.3.4.1.3.3. MMP [mm]

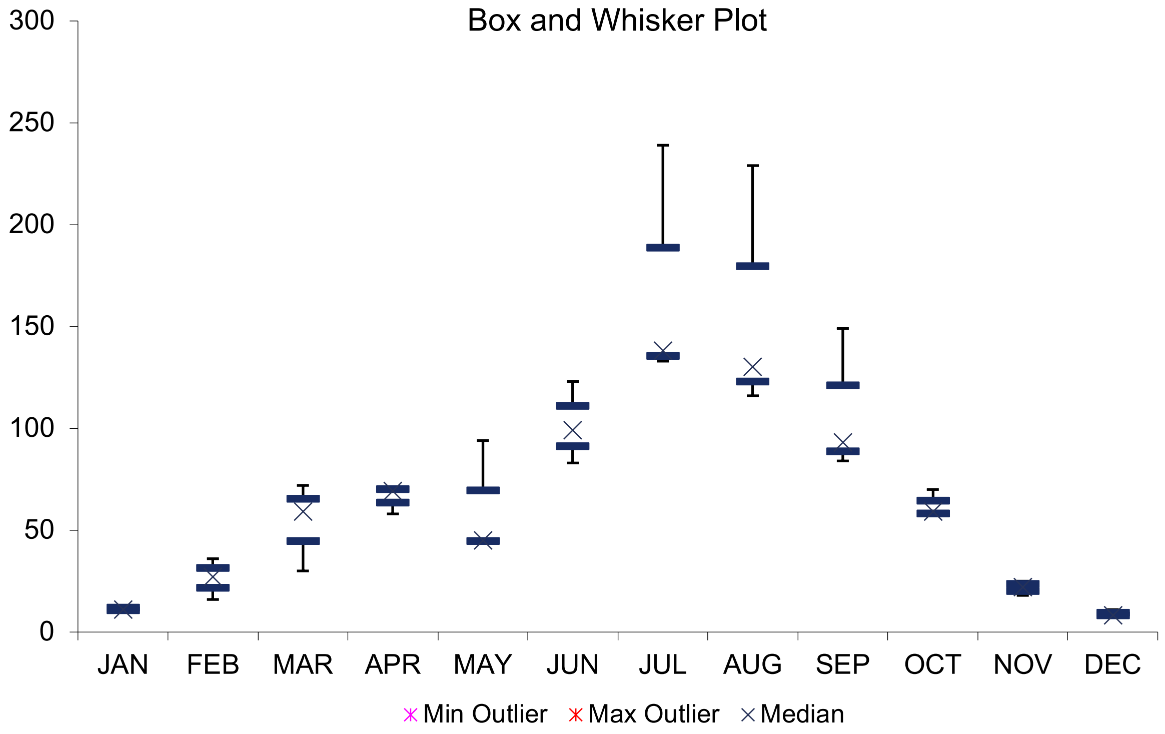

5.3.4.2. Species *Tilia chinensis* Hung T.Chang, 1982

5.3.4.2.1. Köppen profile, distribution, and climate map – GBIF occurrences of *Tilia chinensis*; excluding duplicate occurrences (n = 83), herbarium specimens (n = 77).

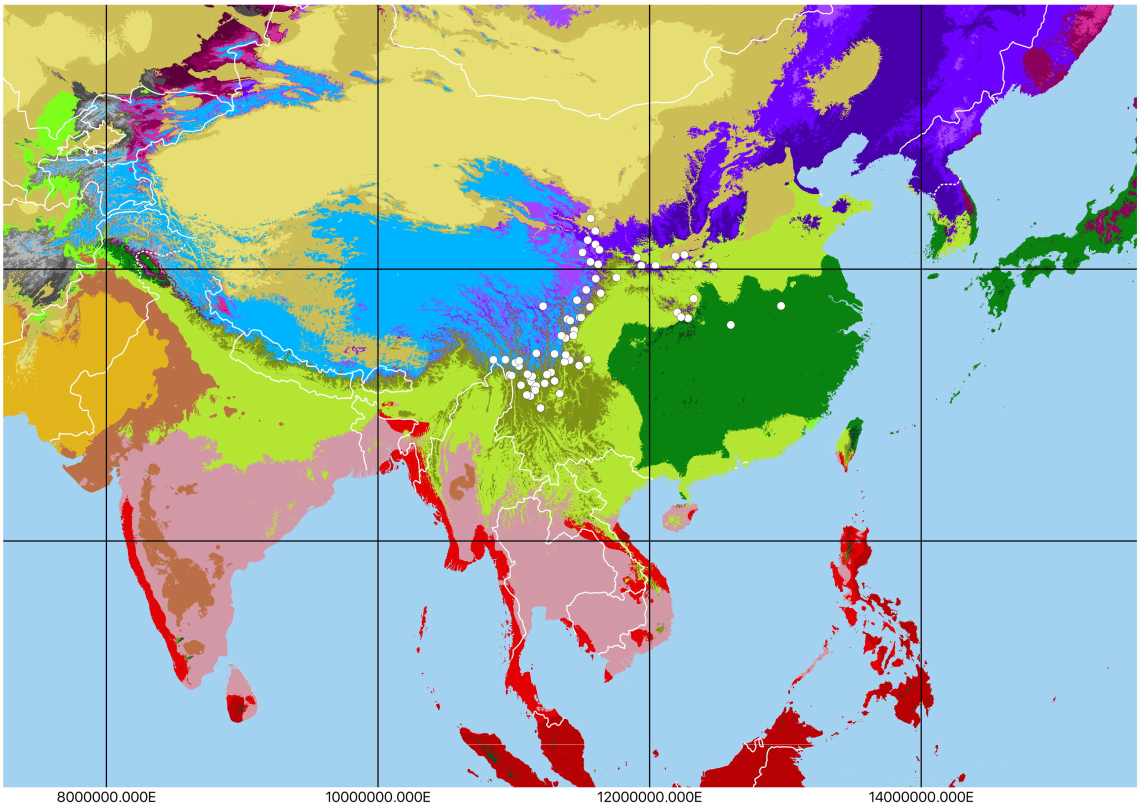

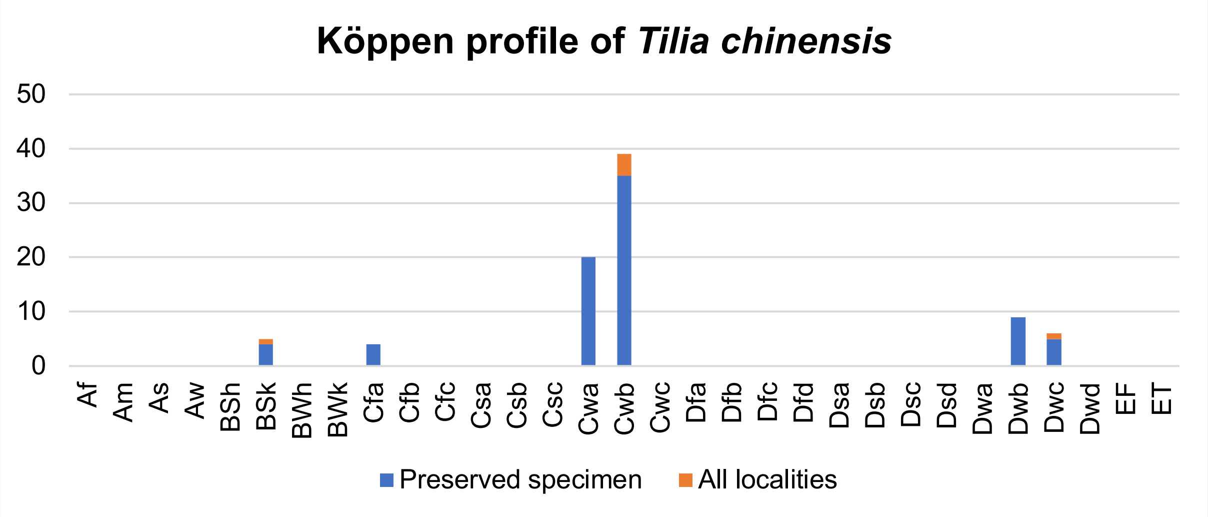

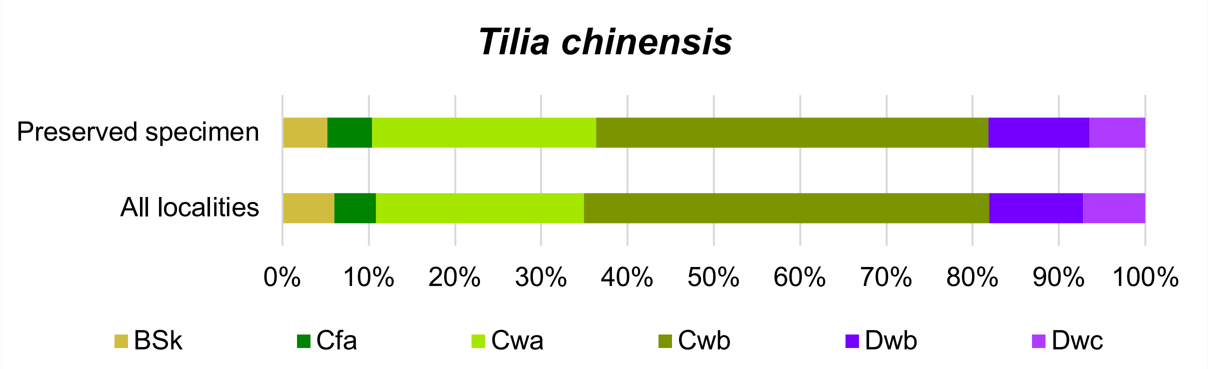

5.3.4.2.2. Biome profile, distribution, and biome map – GBIF occurrences of *Tilia chinensis*; excluding duplicate occurrences (n = 83), herbarium specimens (n = 77).

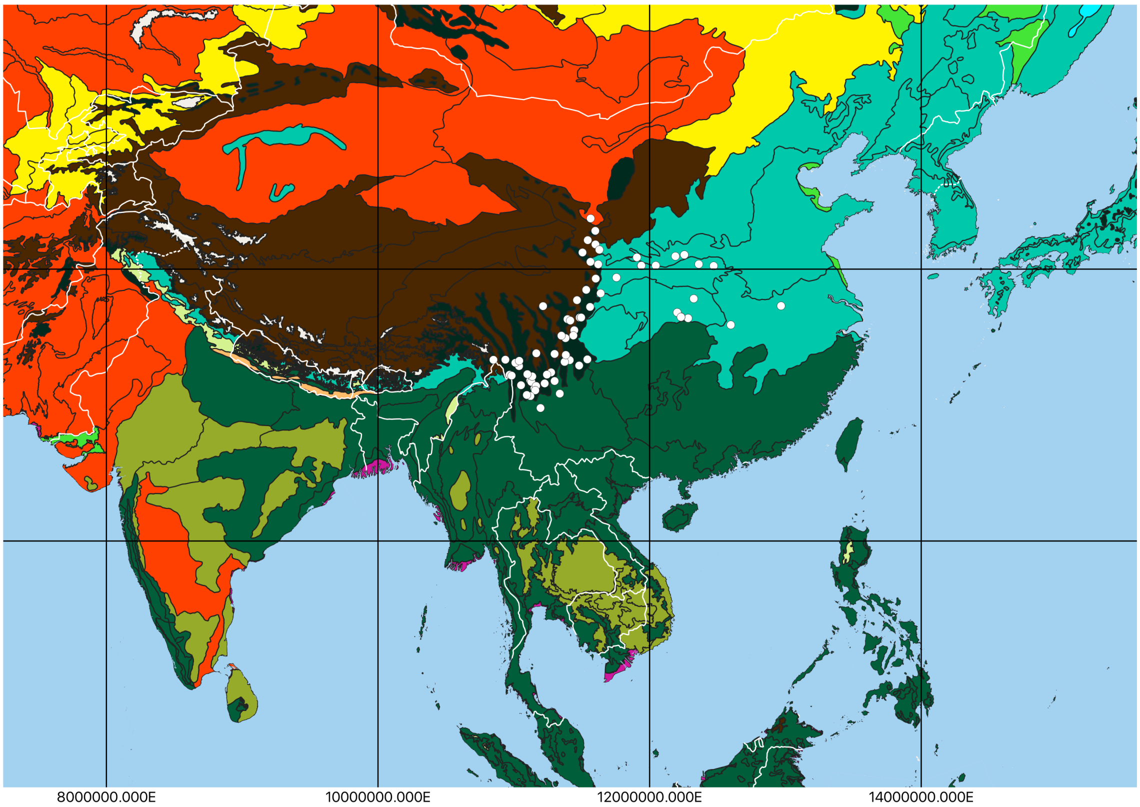

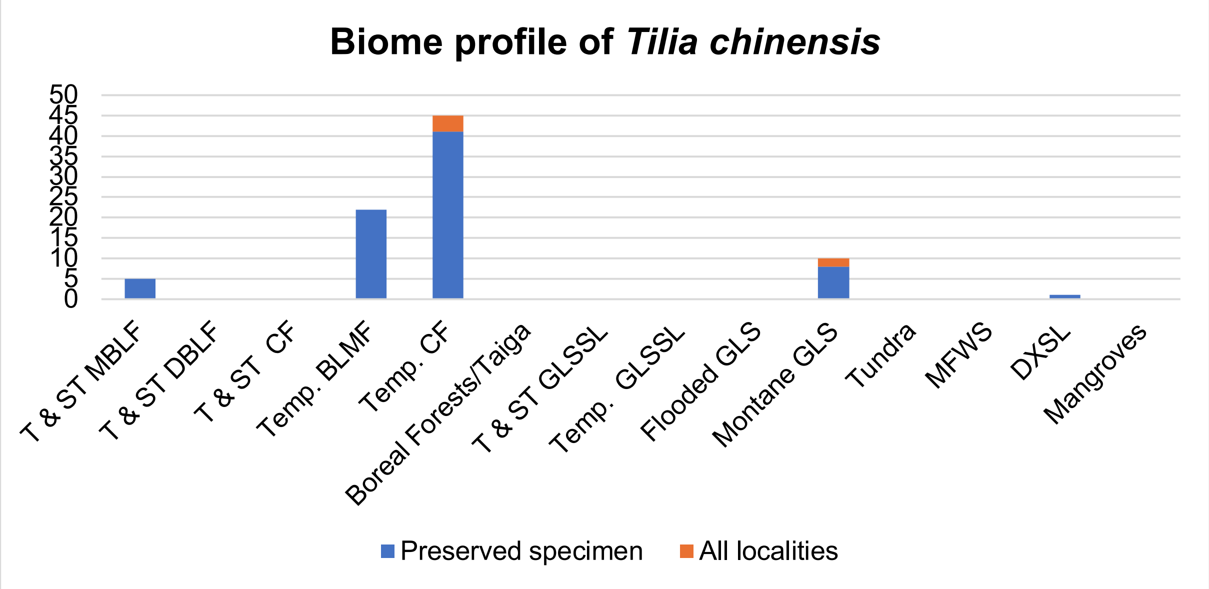

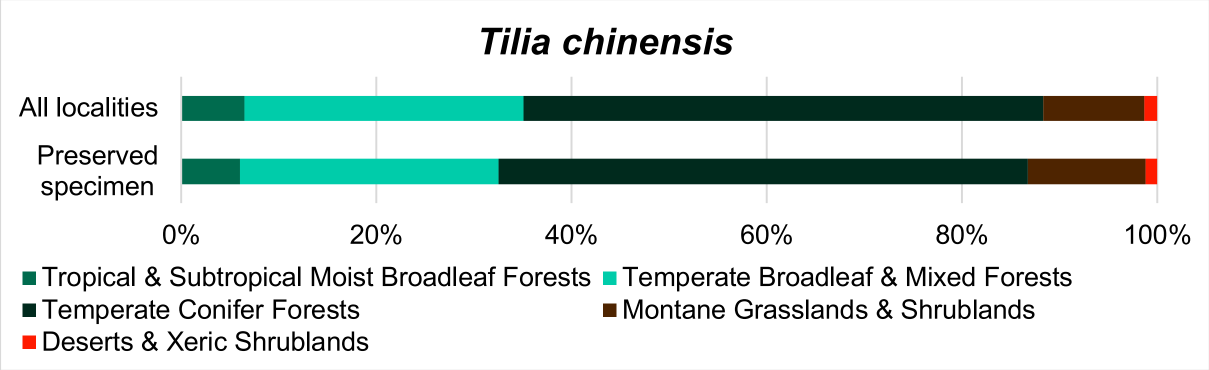

5.3.4.2.3. Climate graphs - based on 83 *Tilia chinensis* occurrences in GBIF

5.3.4.2.3.1. MMT [°C]

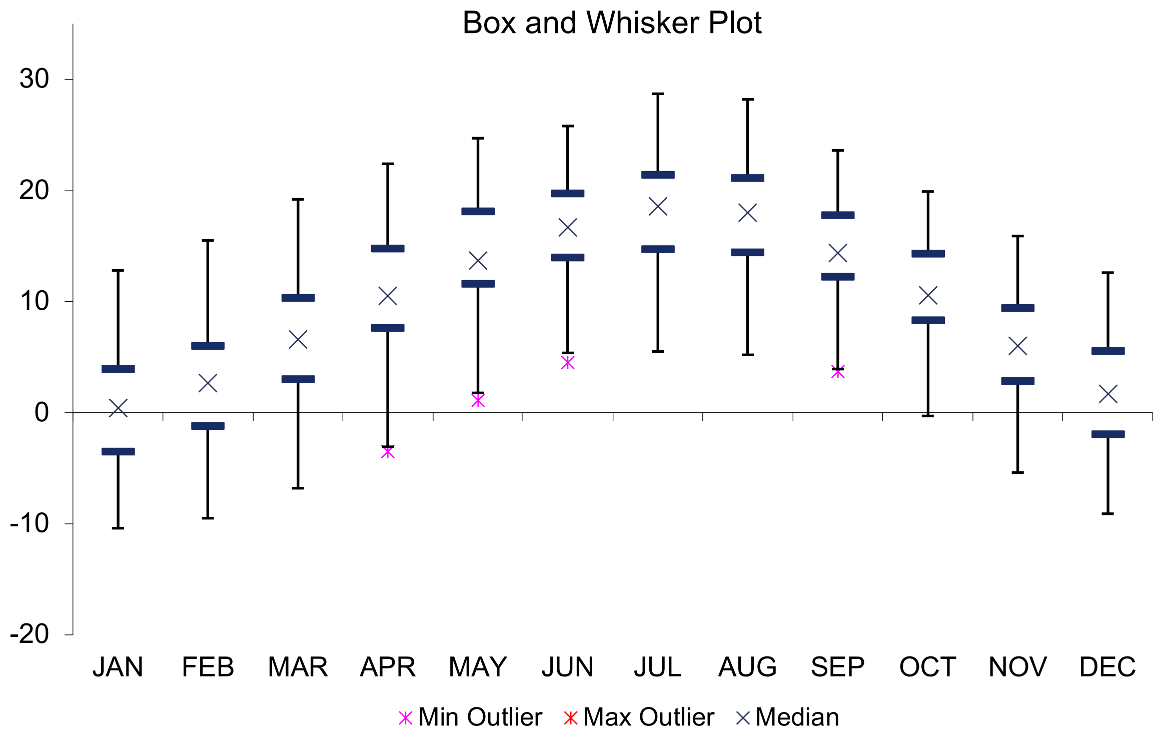

5.3.4.2.3.2. MinMT [°C]

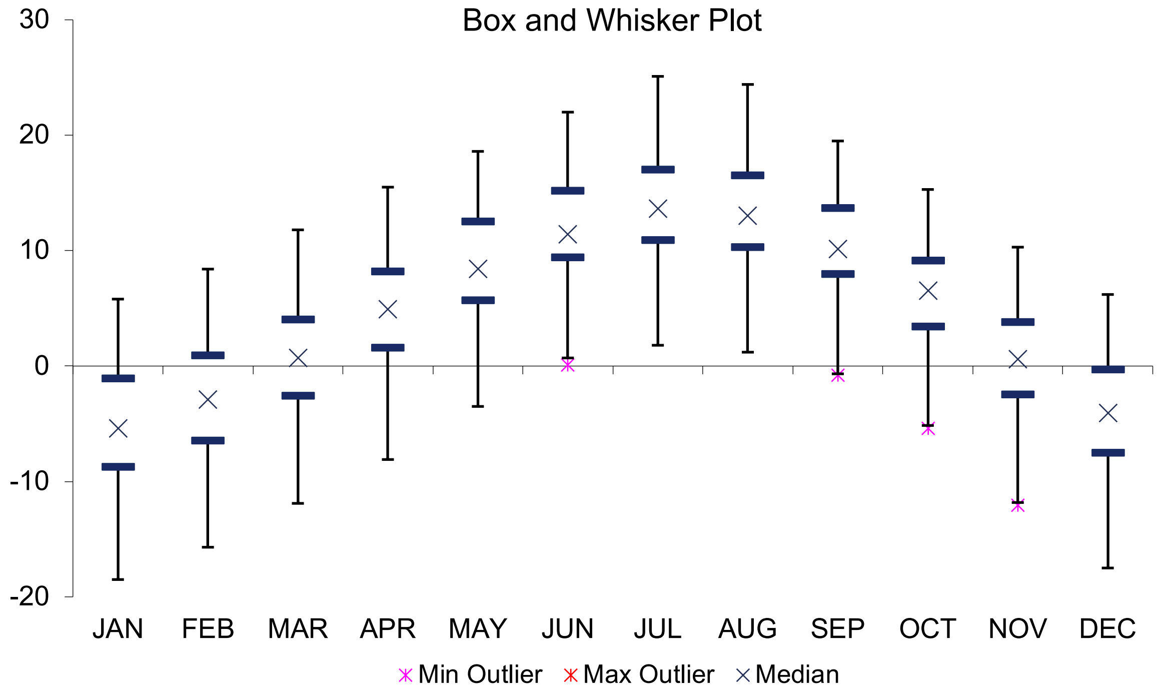

5.3.4.2.3.3. MMP [mm]

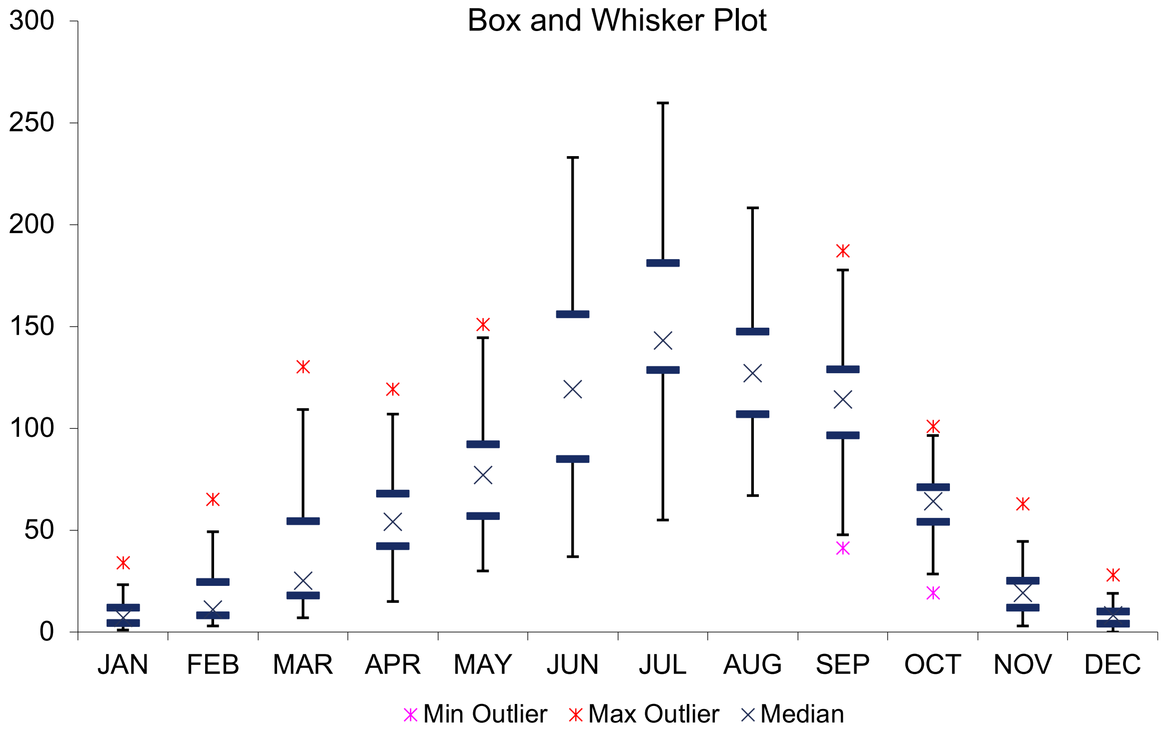

5.3.4.3. Species *Tilia chingiana* Hu et W.C.Cheng, 1935

5.3.4.3.1. Köppen profile, distribution, and climate map – GBIF occurrences of *Tilia chingiana*; excluding duplicate occurrences herbarium specimens (n = 12).

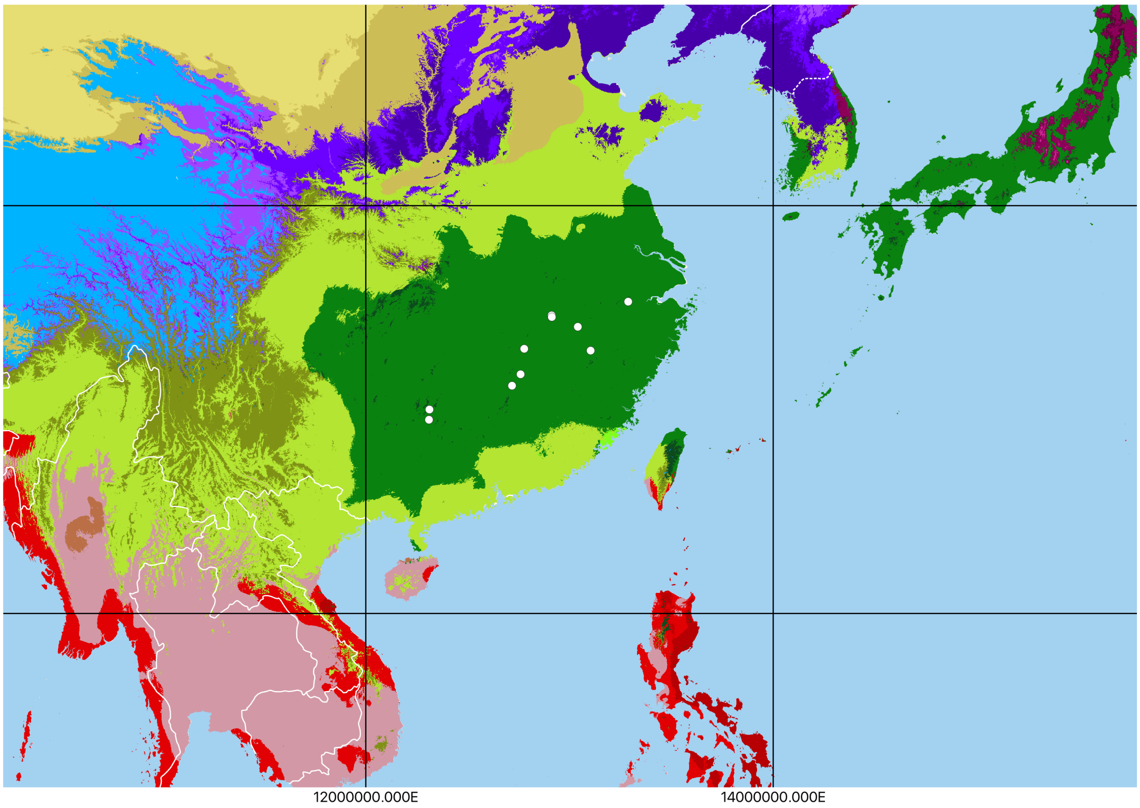

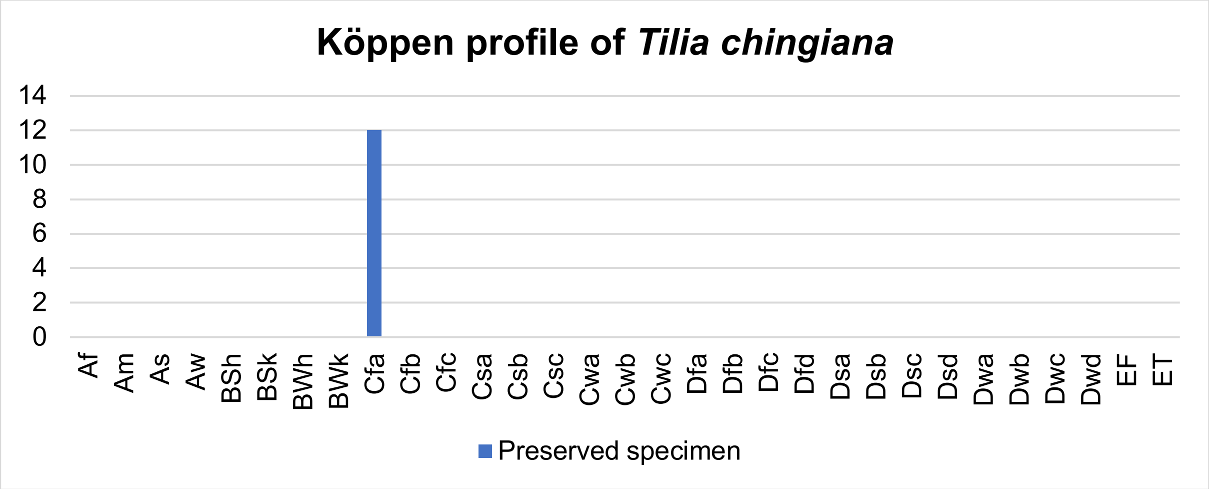

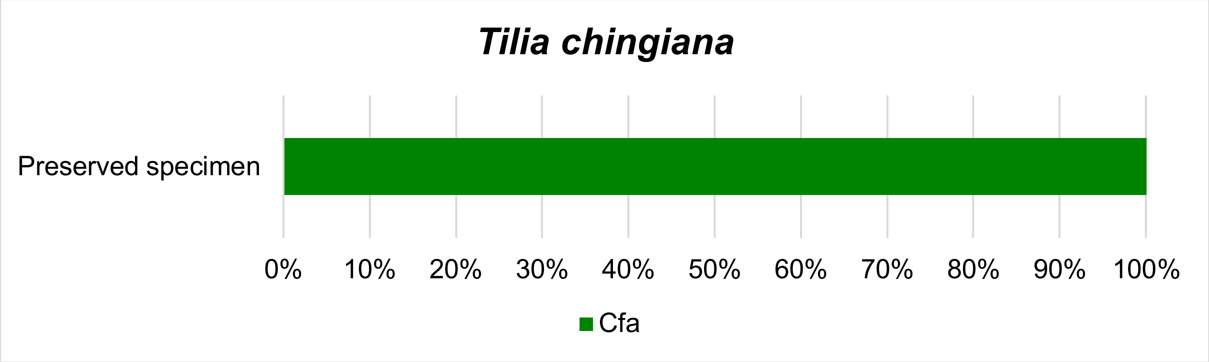

5.3.4.3.2. Biome profile, distribution, and biome map – GBIF occurrences of *Tilia chingiana*; excluding duplicate occurrences herbarium specimens (n = 12).

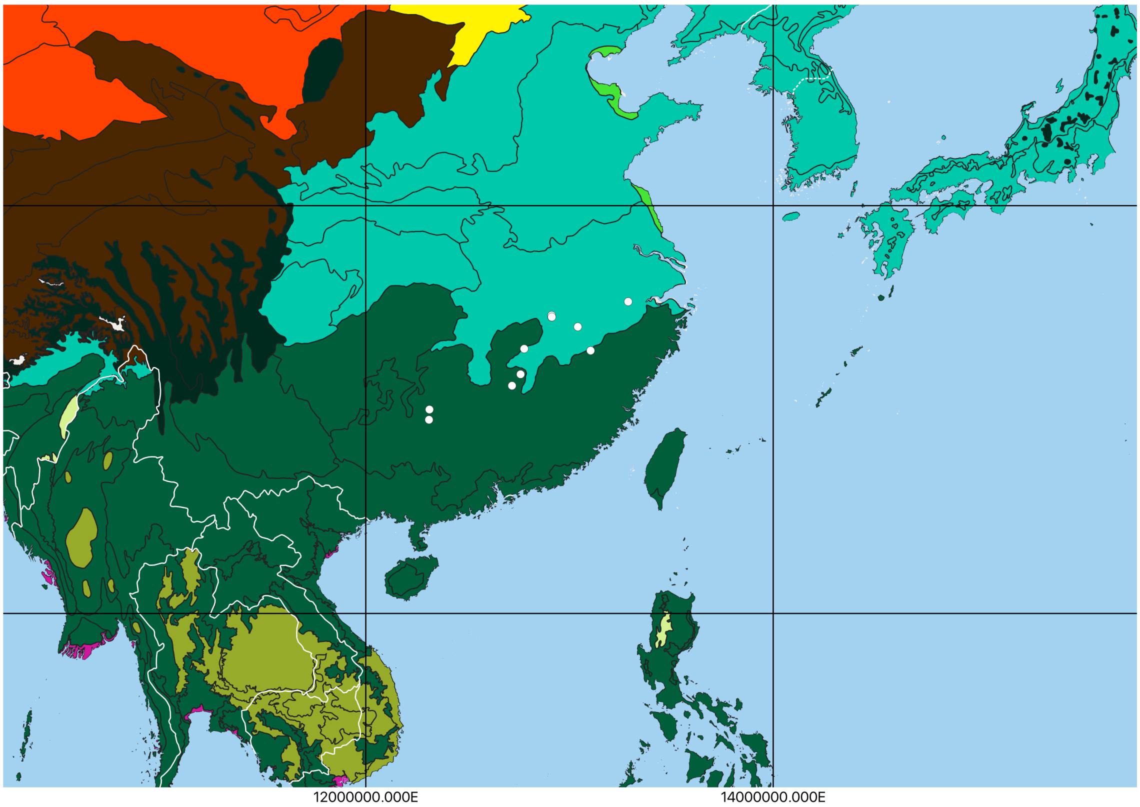

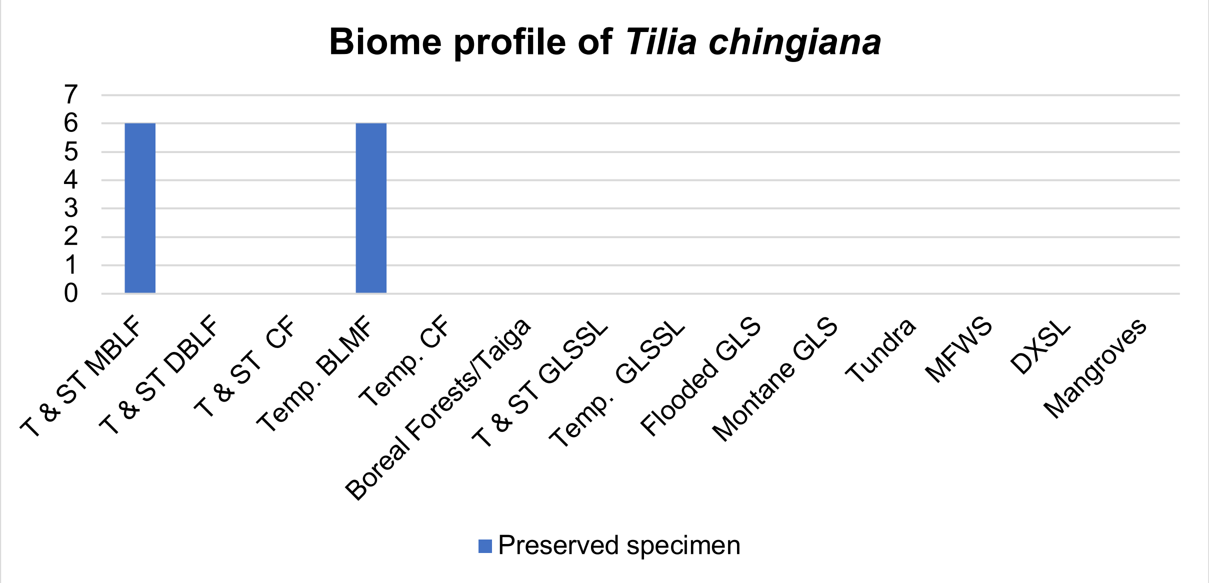

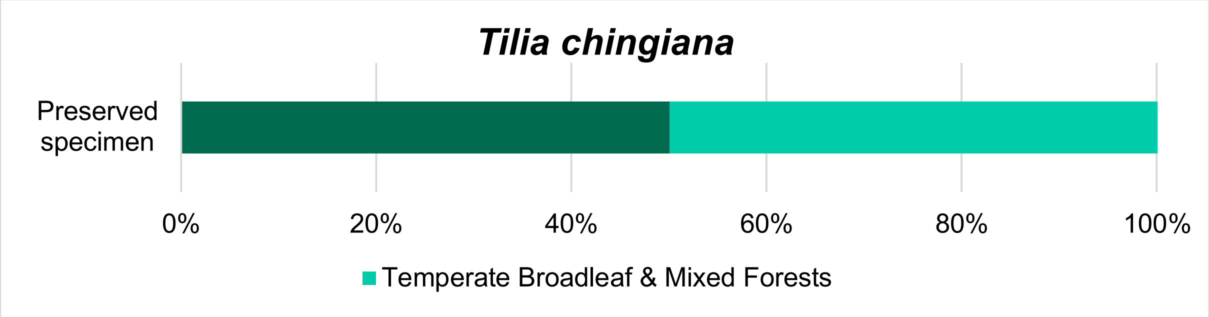

5.3.4.3.3. Climate graphs - based on 12 *Tilia chingiana* occurrences in GBIF
5.3.4.3.3.1. MMT [°C]

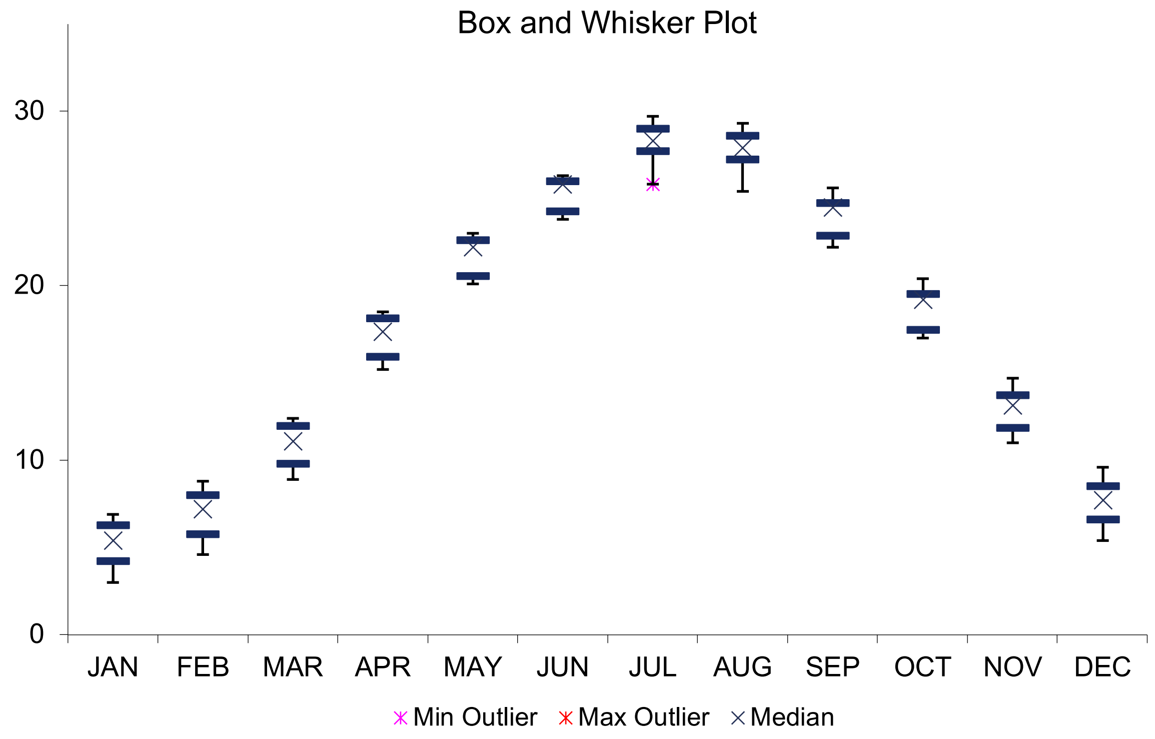

5.4.1.3.3.2. MinMT [°C]

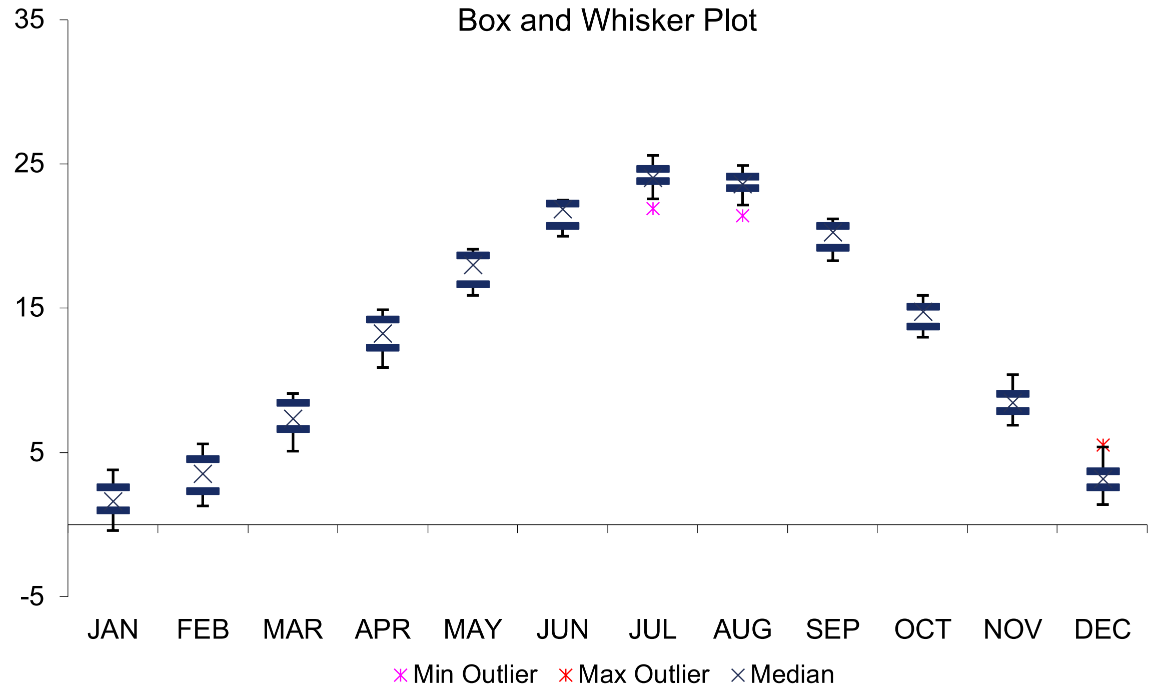

5.3.4.3.3.3. MMP [mm]

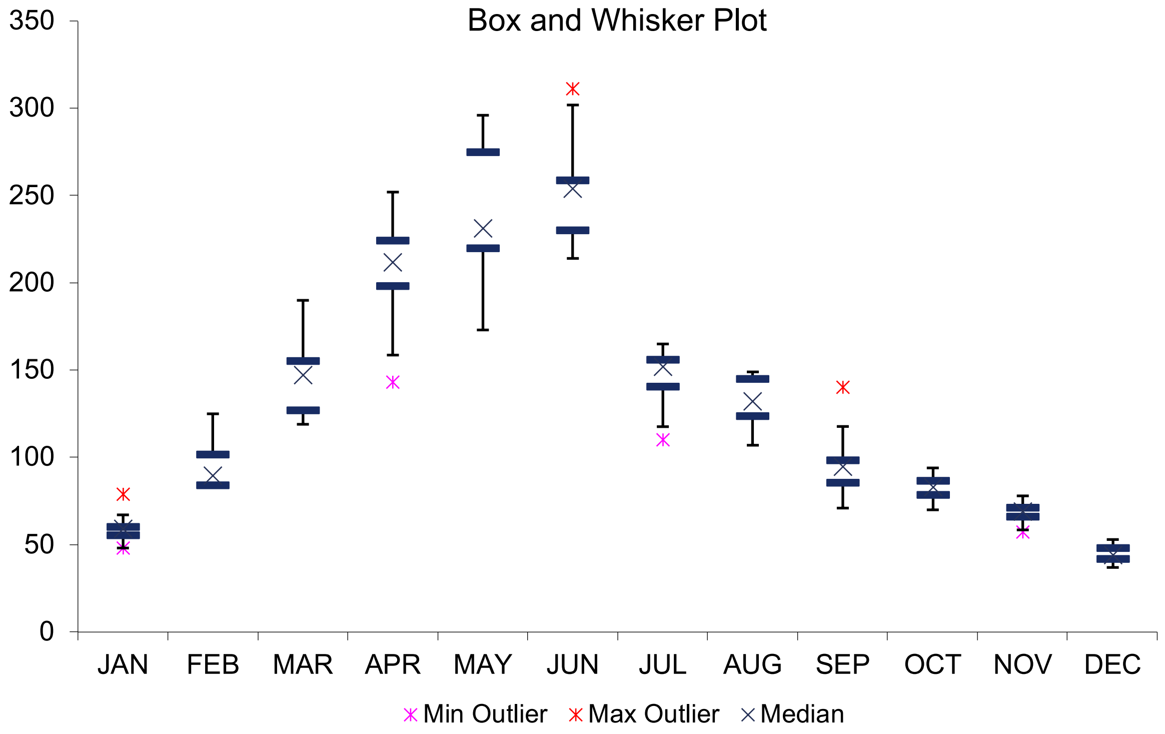

5.3.4.4. Species *Tilia concinna* Pigott, 2012

5.3.4.4.1. Köppen profile, distribution, and climate map – GBIF occurrences of *Tilia concinna*; excluding duplicate occurrences herbarium specimens (n = 1).

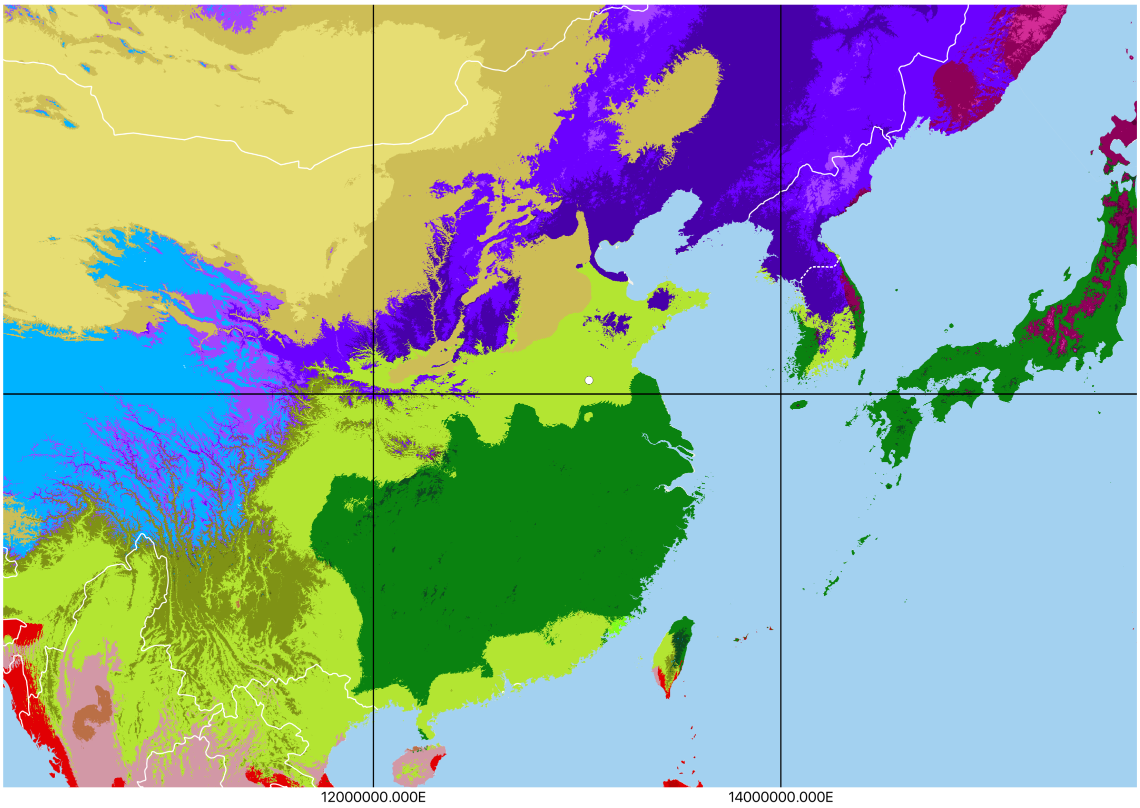

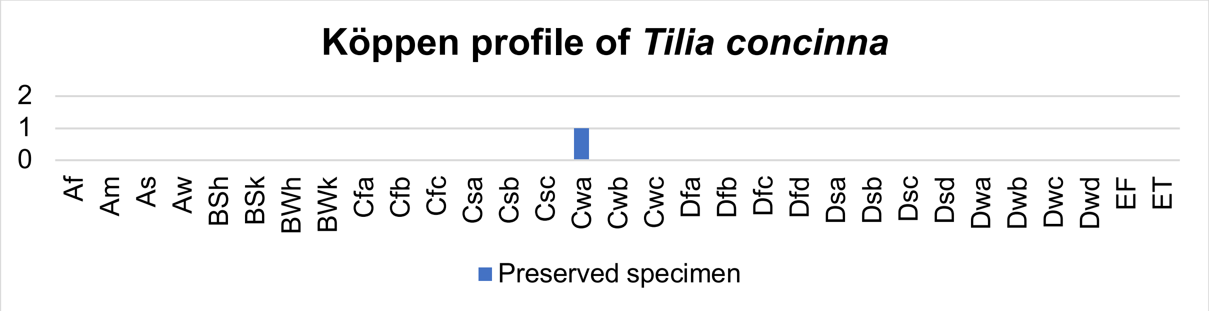

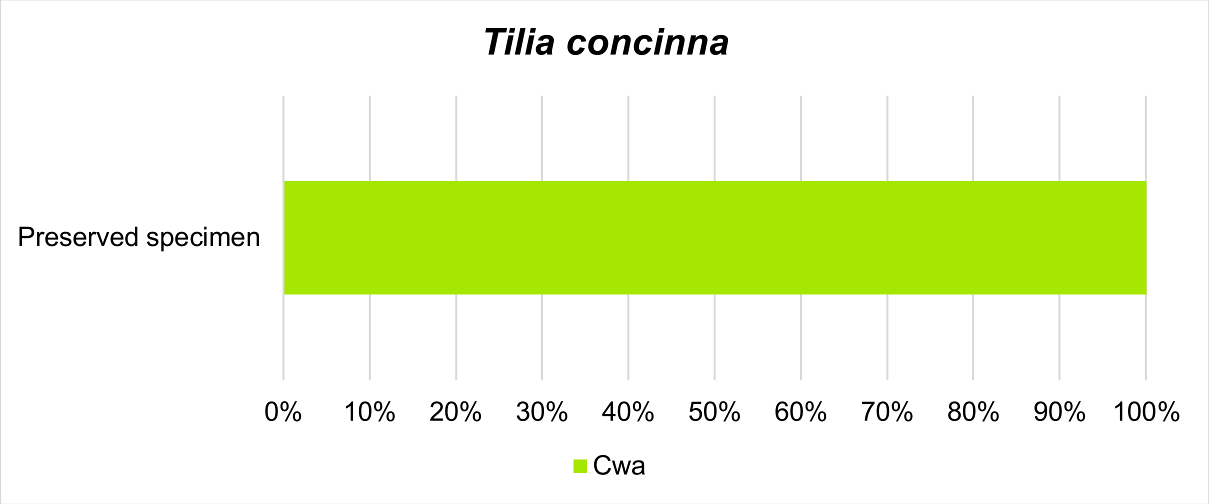

5.3.4.4.2. Biome profile, distribution, and biome map – GBIF occurrences of *Tilia concinna*; excluding duplicate occurrences herbarium specimens (n = 1).

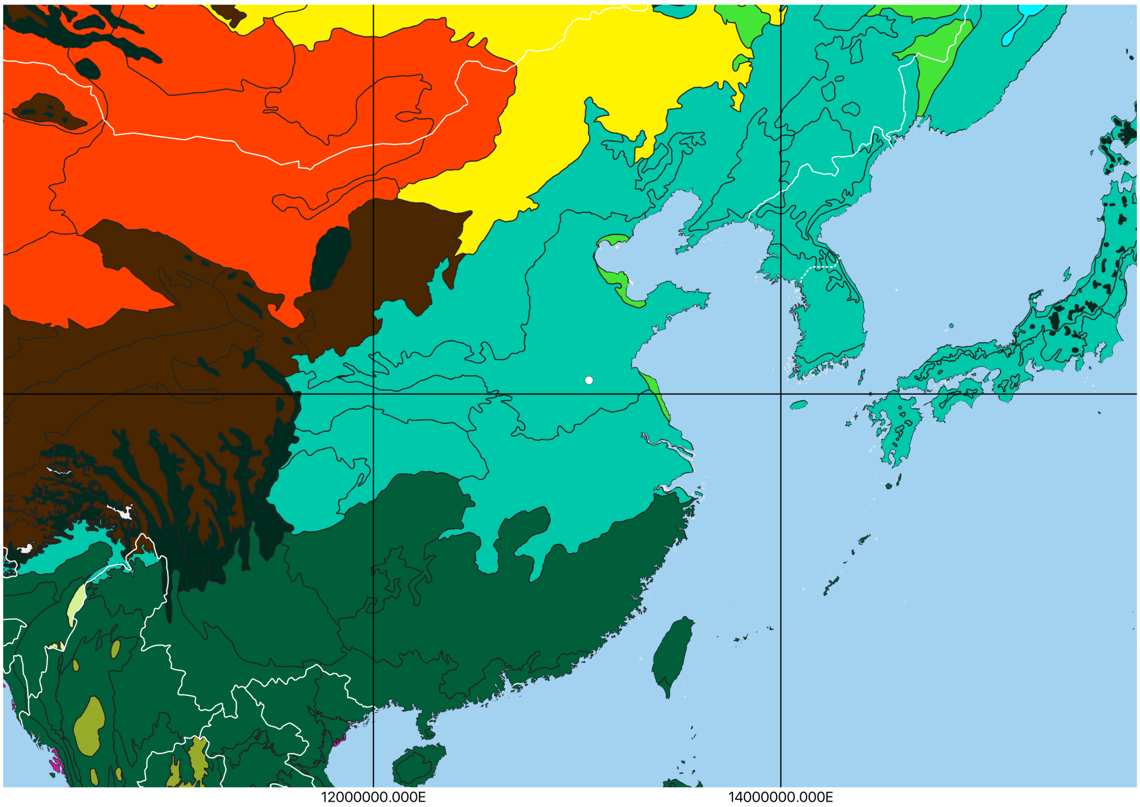

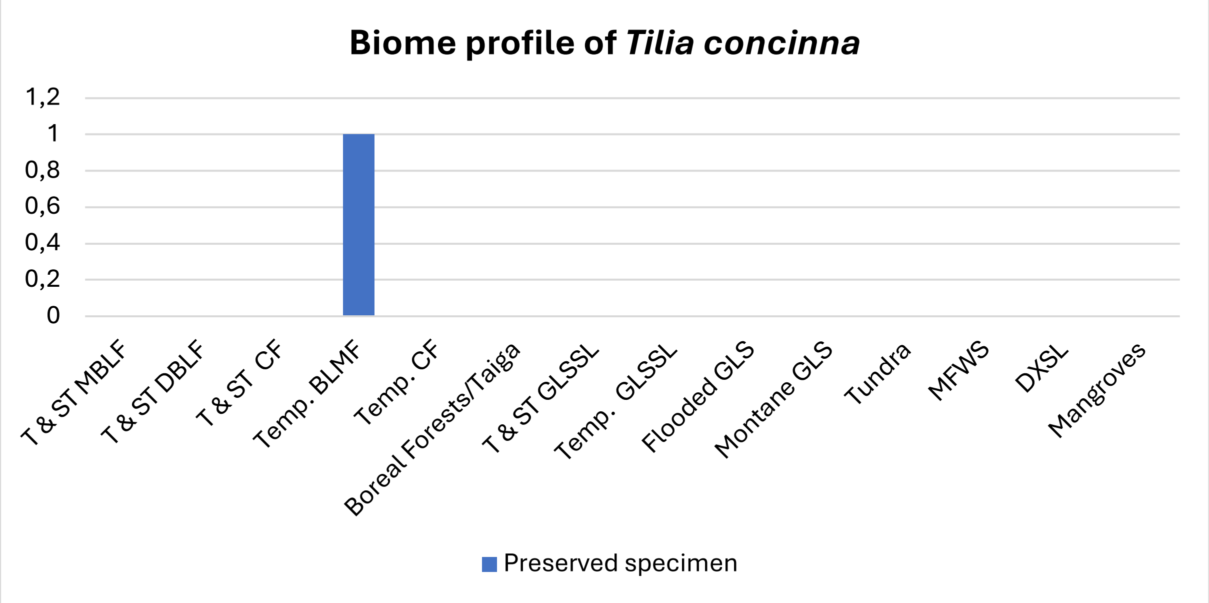

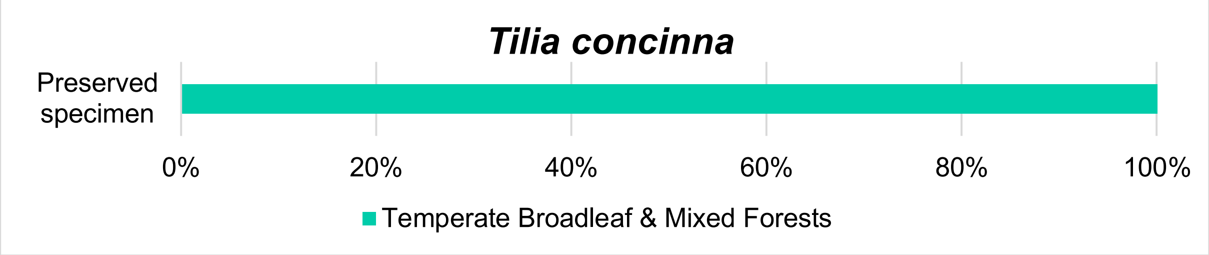

5.3.4.4.3. Climate graphs - based on 1 *Tilia concinna* occurrences in GBIF
5.3.4.4.3.1. MMT [°C]

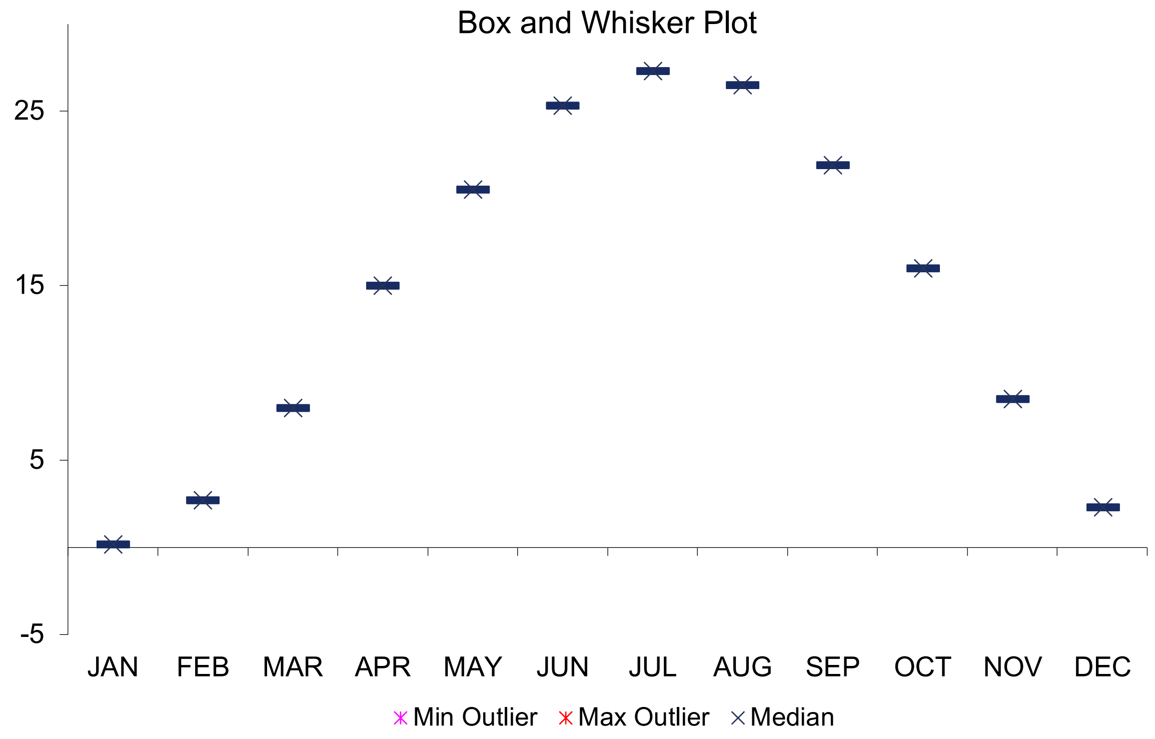

5.3.4.4.3.2. MinMT [°C]

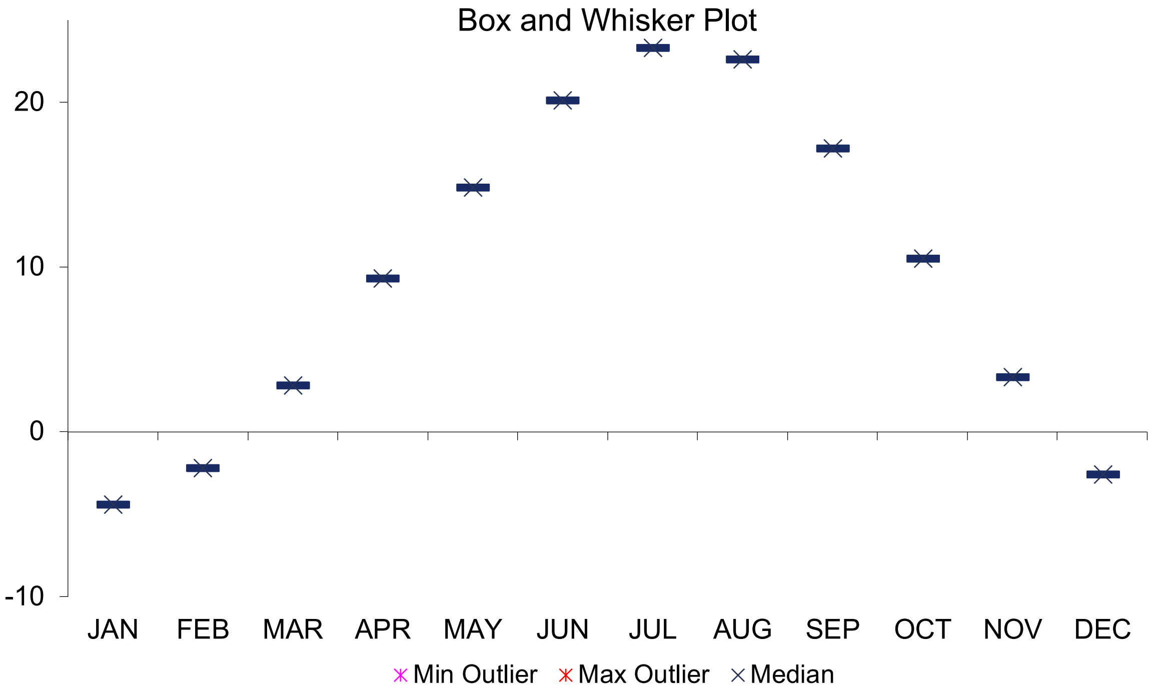

5.3.4.4.3.3. MMP [mm]

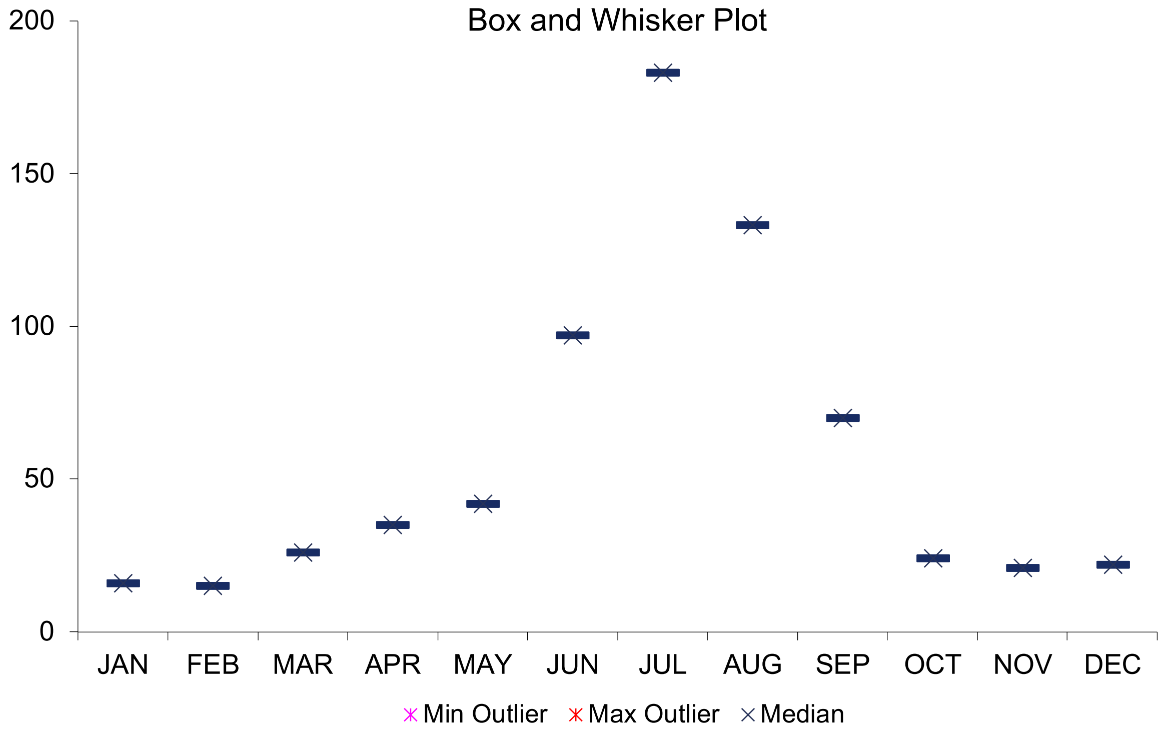

5.3.4.5. Species *Tilia mandshurica* Rupr. et Maxim., 1856

5.3.4.5.1. Köppen profile, distribution, and climate map – GBIF occurrences of *Tilia mandshurica*; excluding duplicate occurrences (n = 388), herbarium specimens (n = 158).

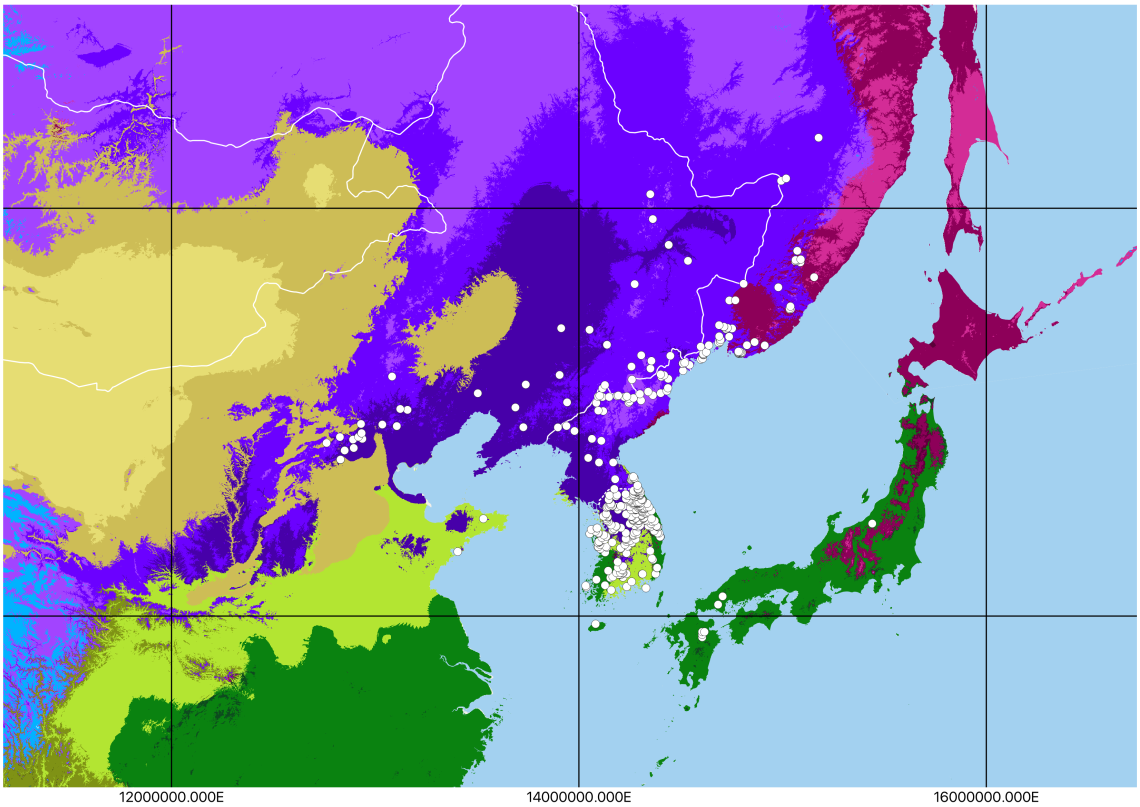

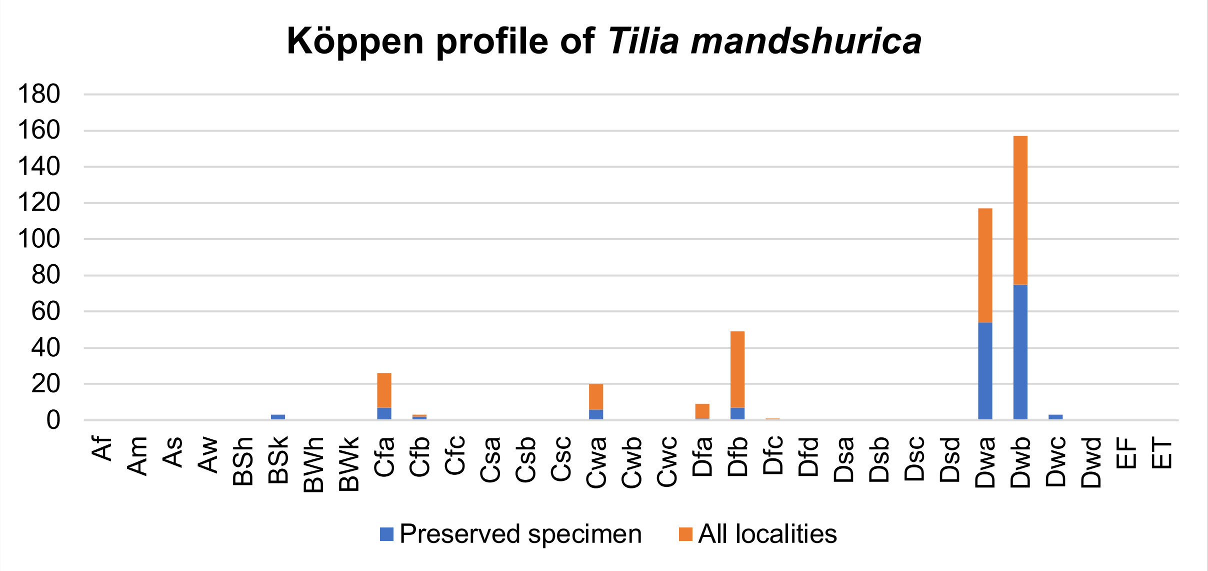

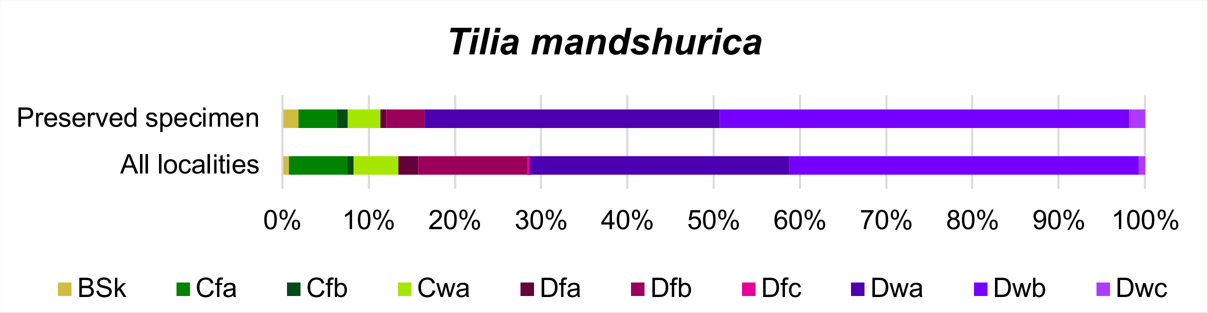

5.3.4.5.2. Biome profile, distribution, and biome map – GBIF occurrences of *Tilia callidonta*; excluding duplicate occurrences (n = 384), herbarium specimens (n = 158).

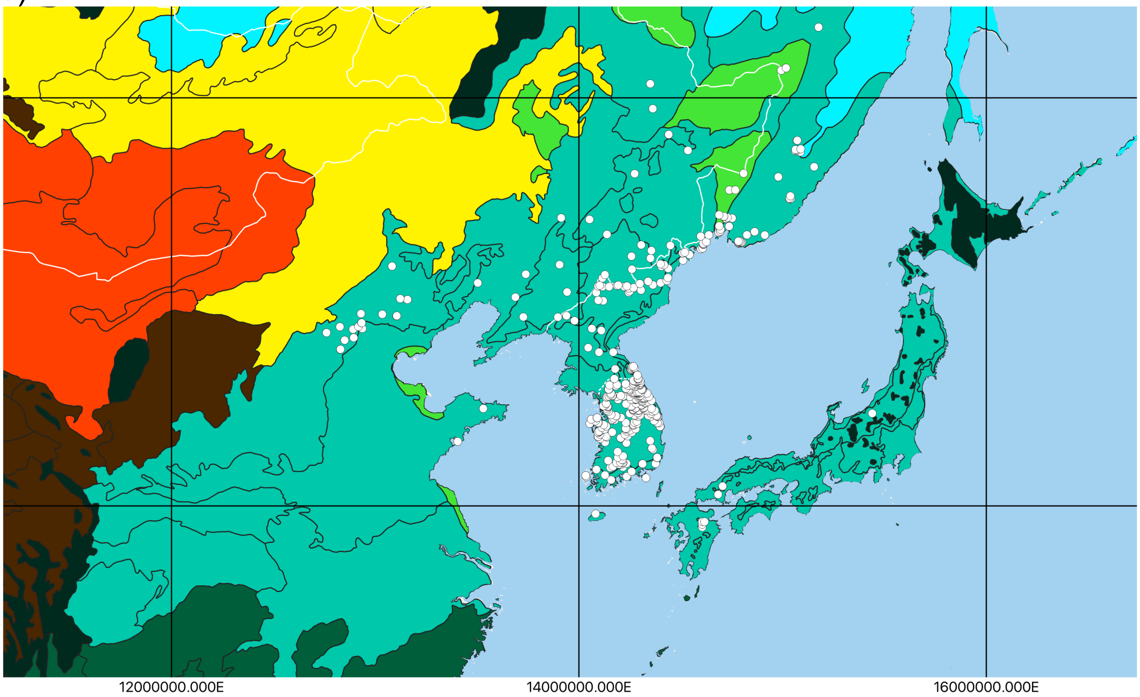

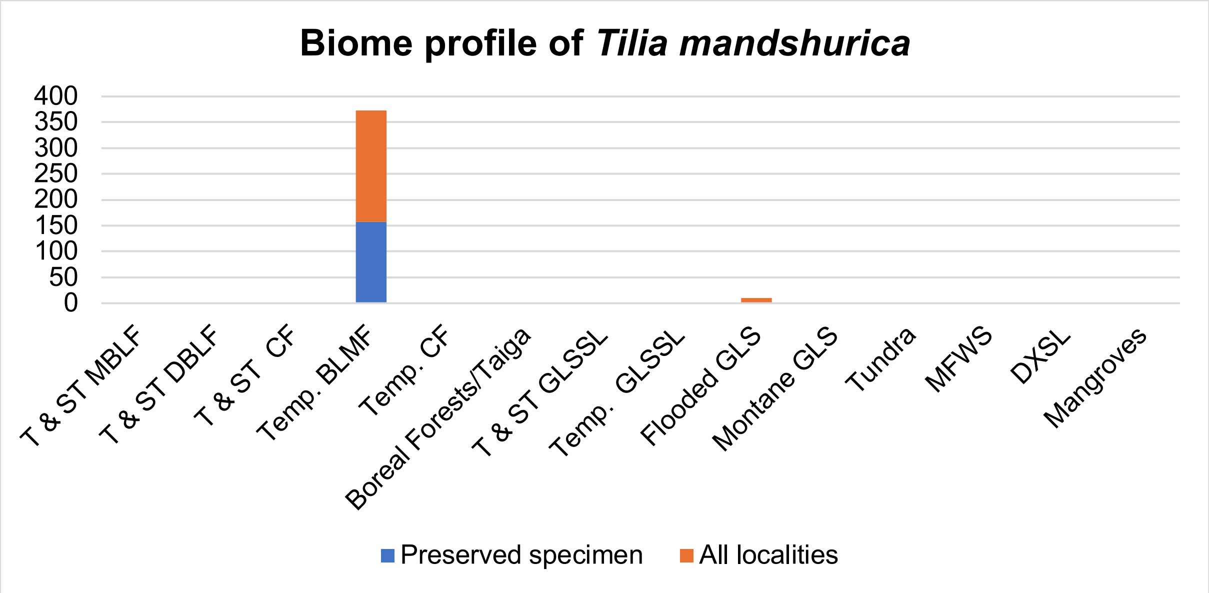

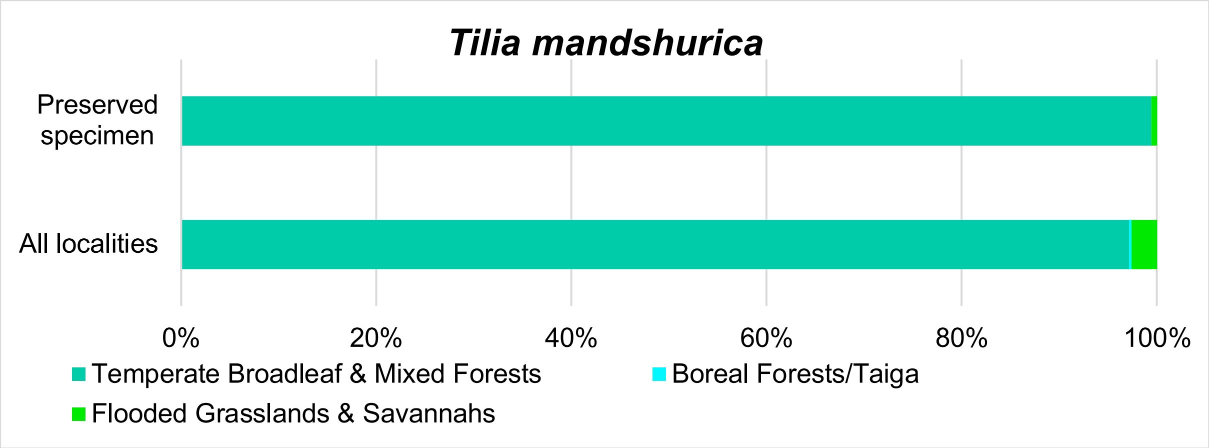

5.3.4.5.3. Climate graphs - based on 386 *Tilia callidonta* occurrences in GBIF

5.3.4.5.3.1. MMT [°C]

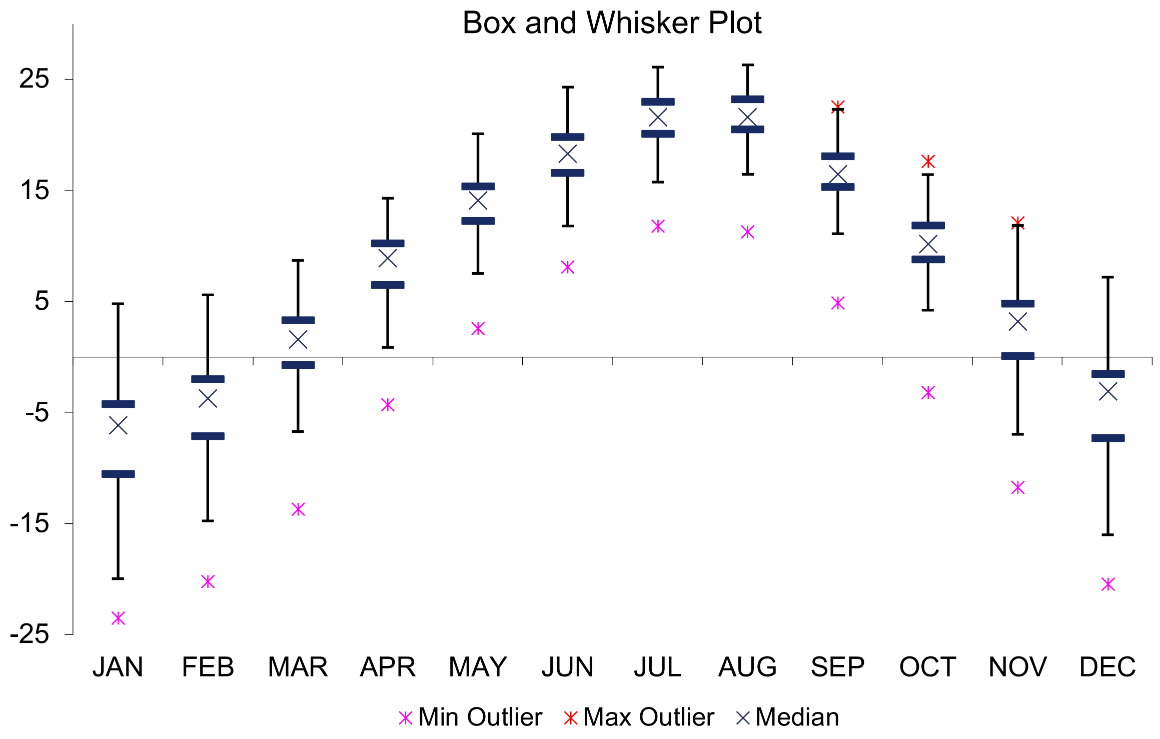

5.3.4.5.3.2. MinMT [°C]

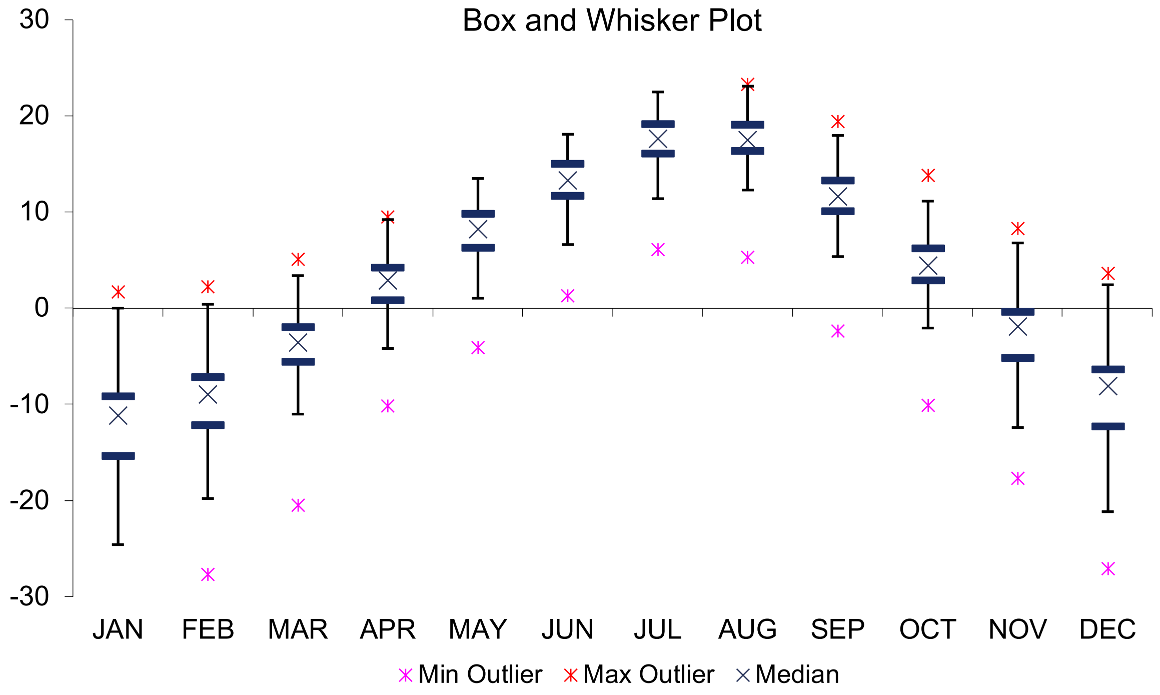

5.3.4.5.3.3. MMP [mm]

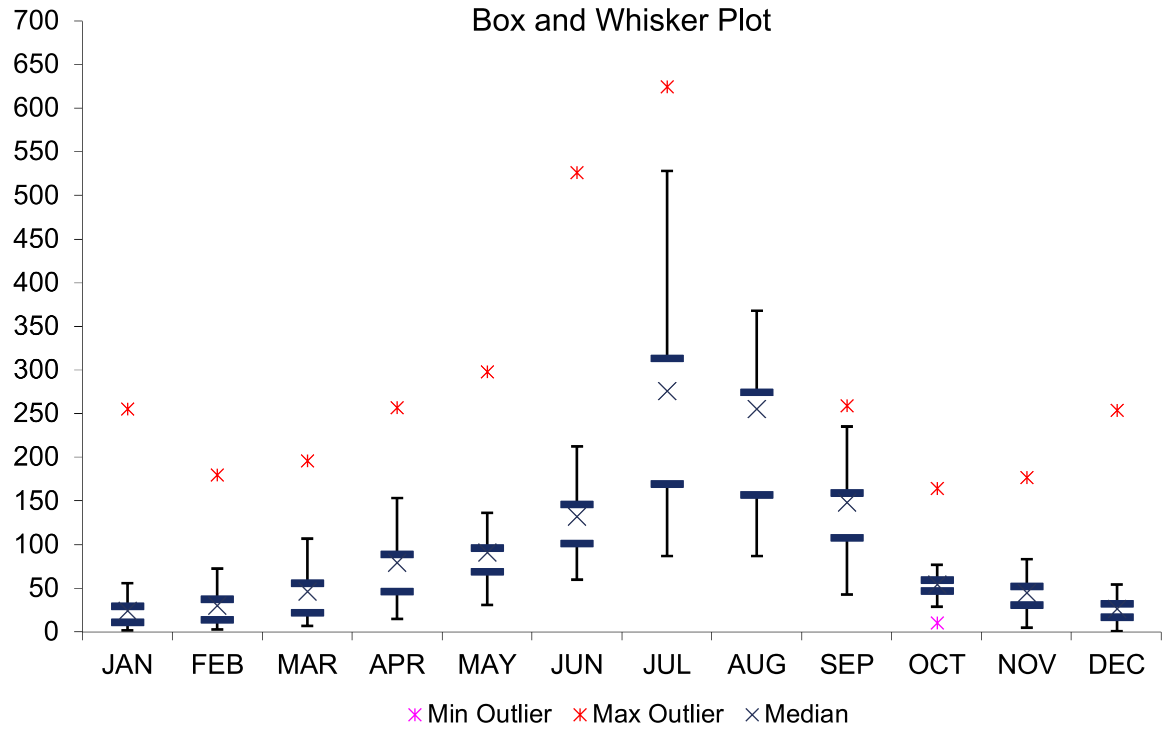

5.3.4.6. Species *Tilia maximowicziana* Hung T.Chang, 1982

5.3.4.6.1. Köppen profile, distribution, and climate map – GBIF occurrences of *Tilia maximowicziana*; excluding duplicate occurrences (n = 386), herbarium specimens (n = 361).

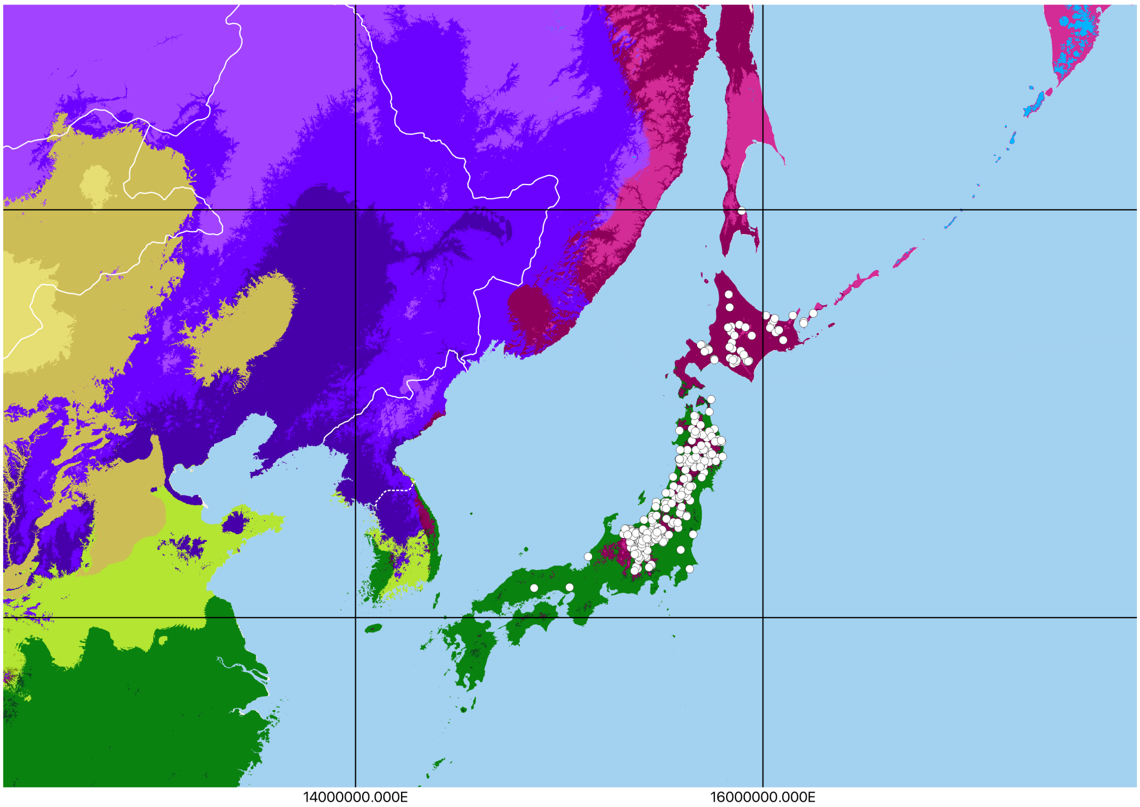

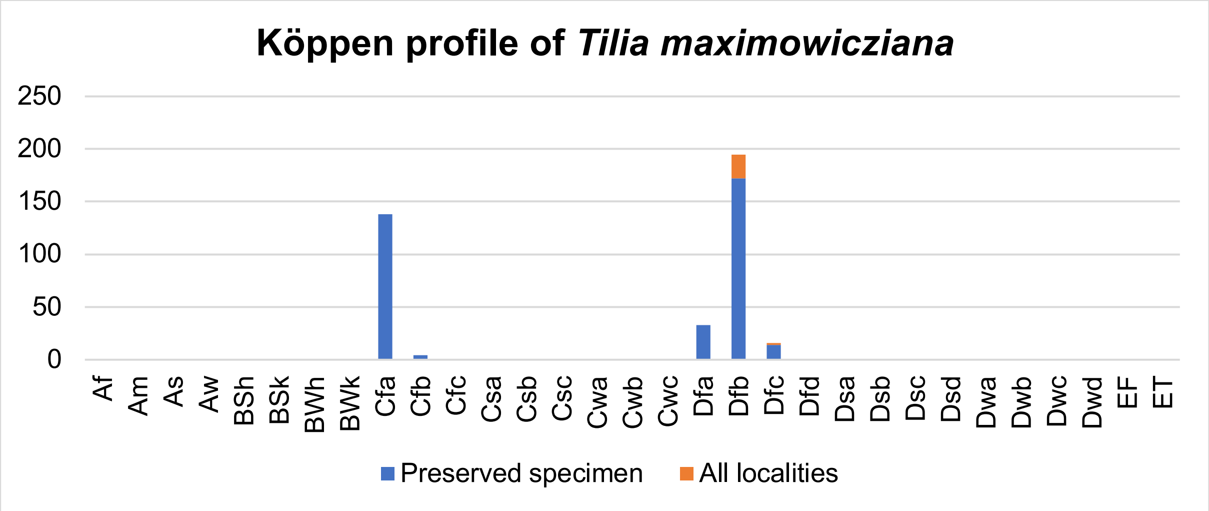

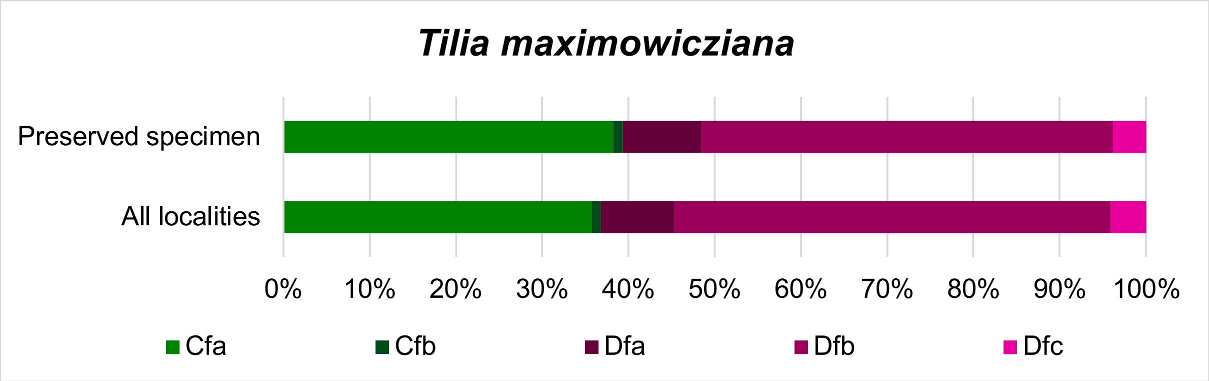

5.3.4.6.2. Biome profile, distribution, and biome map – GBIF occurrences of *Tilia maximowicziana*; excluding duplicate occurrences (n = 382), herbarium specimens (n = 357).

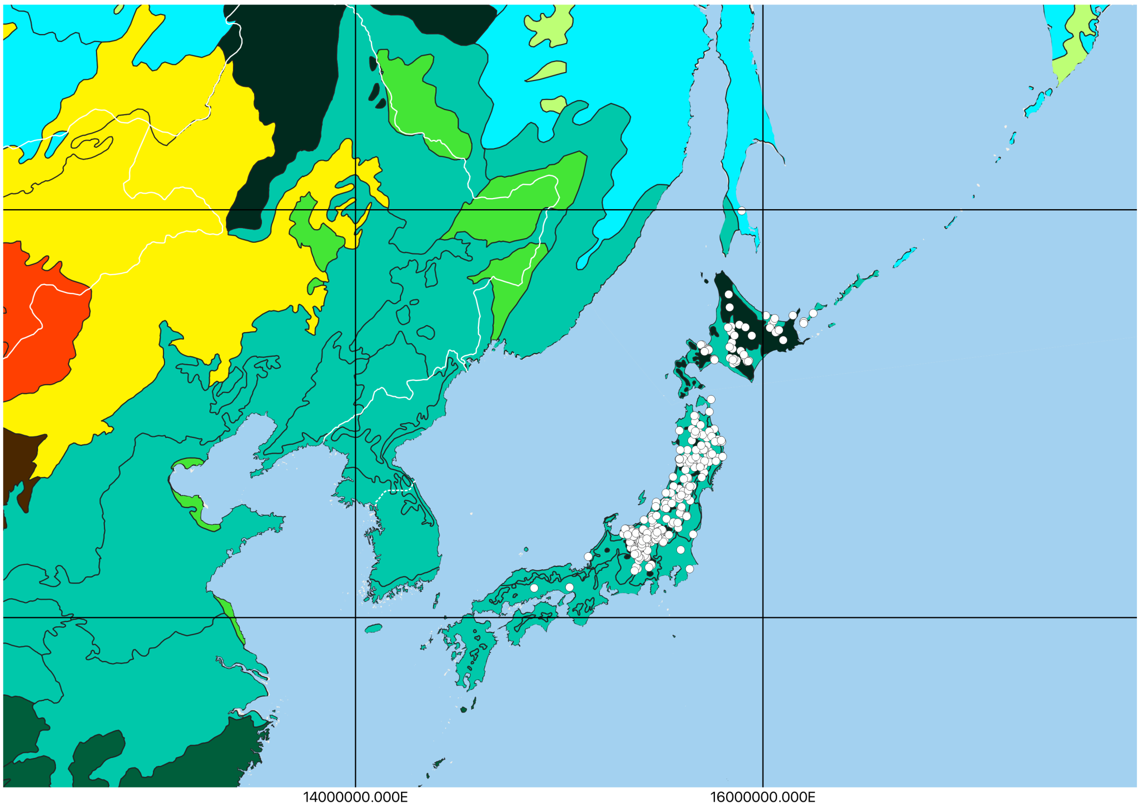

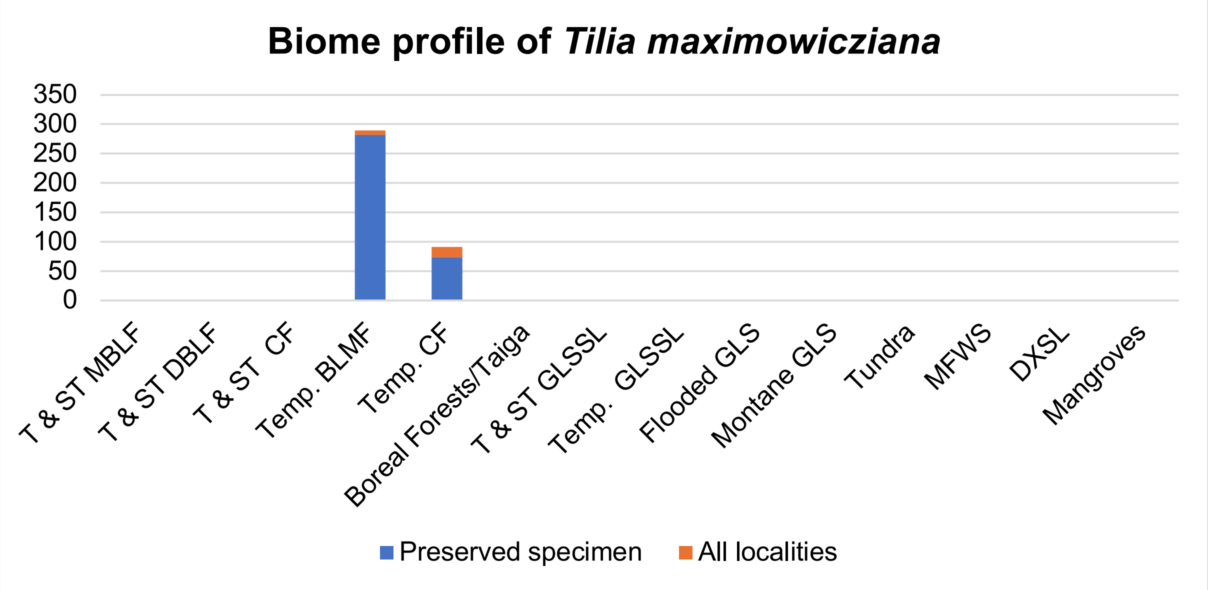

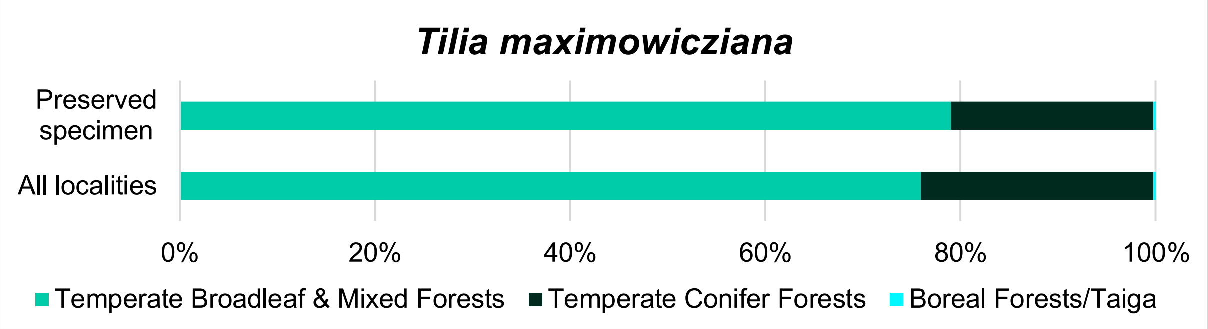

5.3.4.6.3. Climate graphs - based on 385 *Tilia maximowicziana* occurrences in GBIF

5.3.4.6.3.1. MMT [°C]

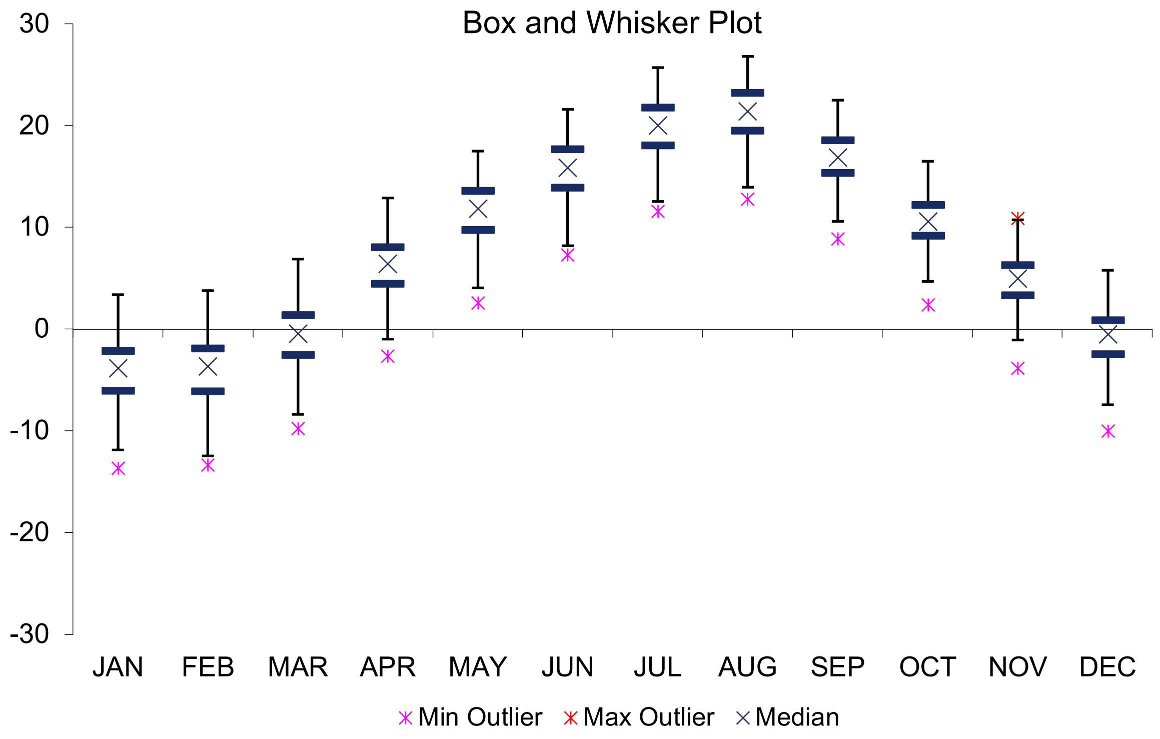

5.3.4.6.3.2. MinMT [°C]

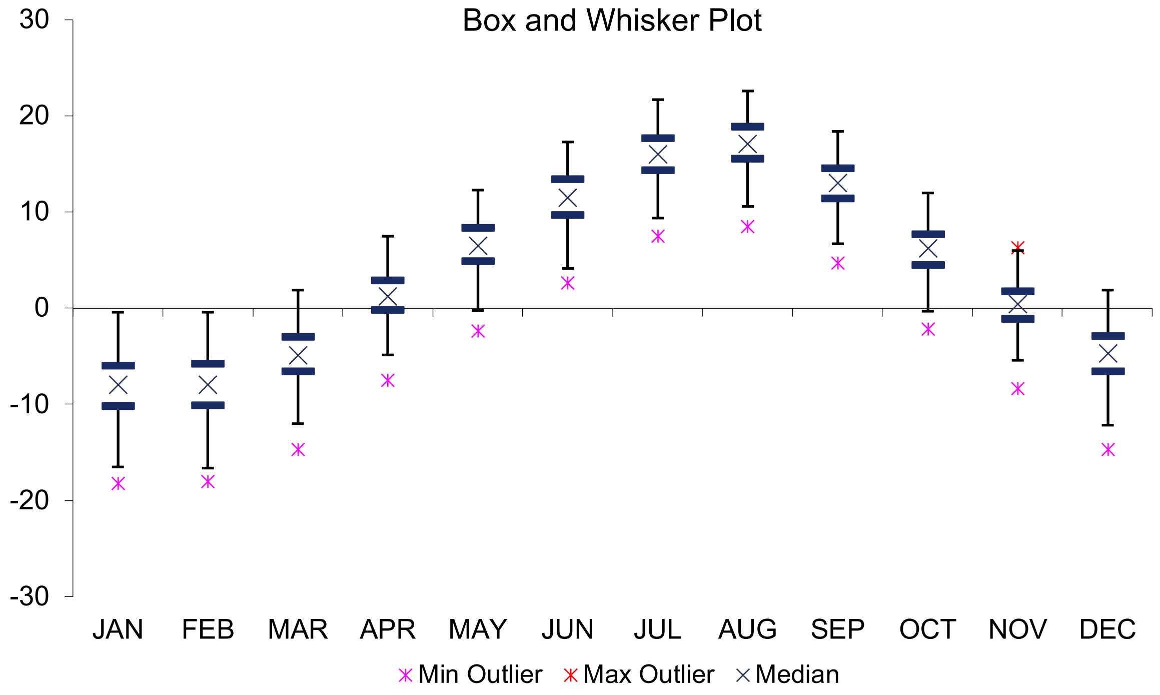

5.3.4.6.3.3. MMP [mm]

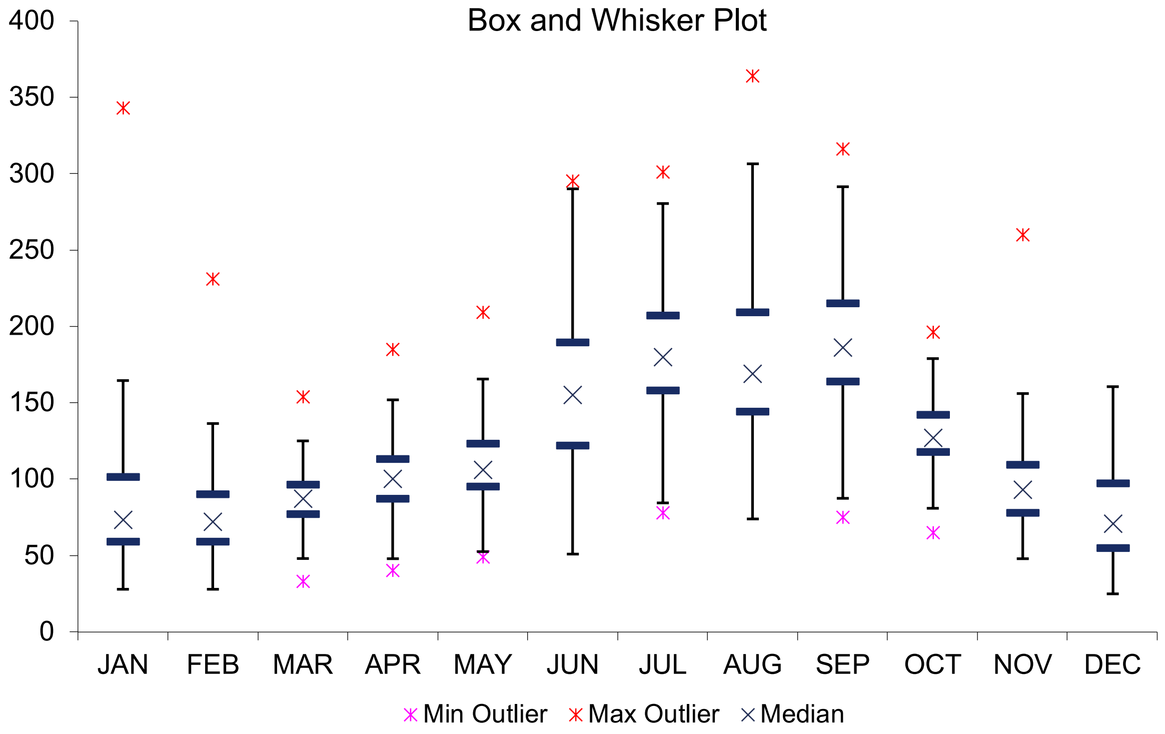

5.3.4.7. Species *Tilia miqueliana* Maxim., 1856

5.3.4.7.1. Köppen profile, distribution, and climate map – GBIF occurrences of *Tilia miqueliana*; excluding duplicate occurrences (n = 20), herbarium specimens (n = 17).

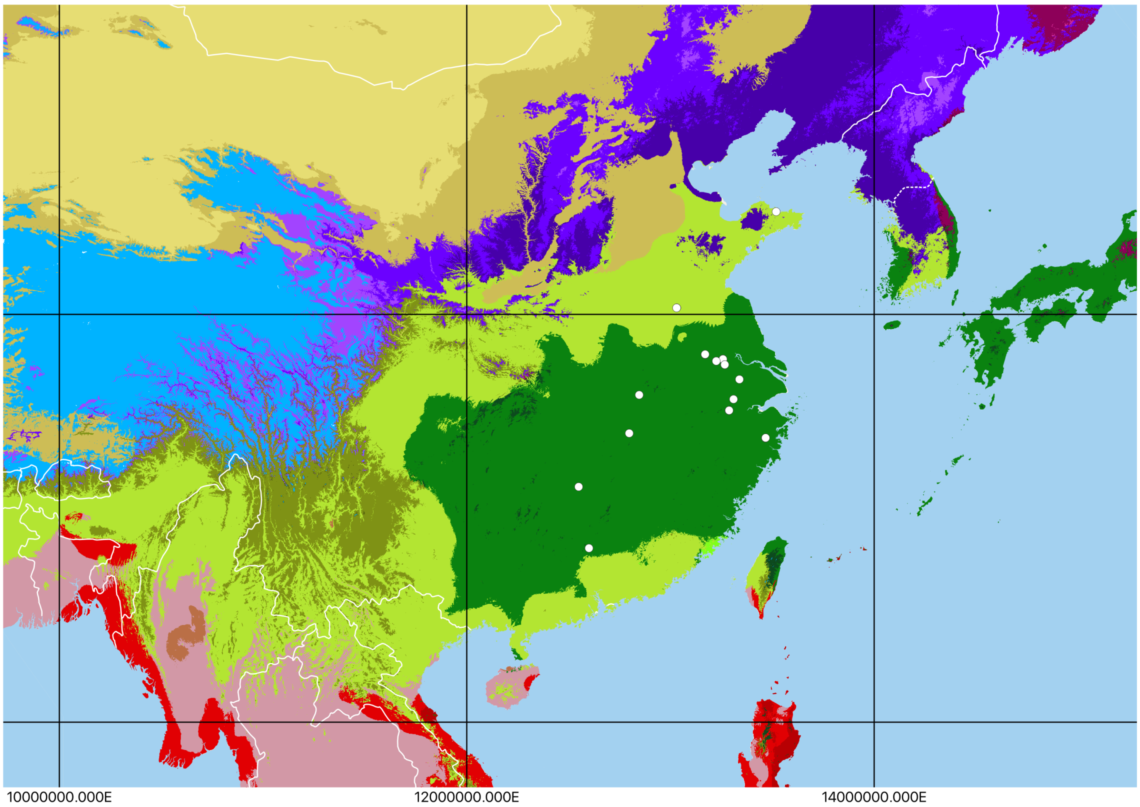

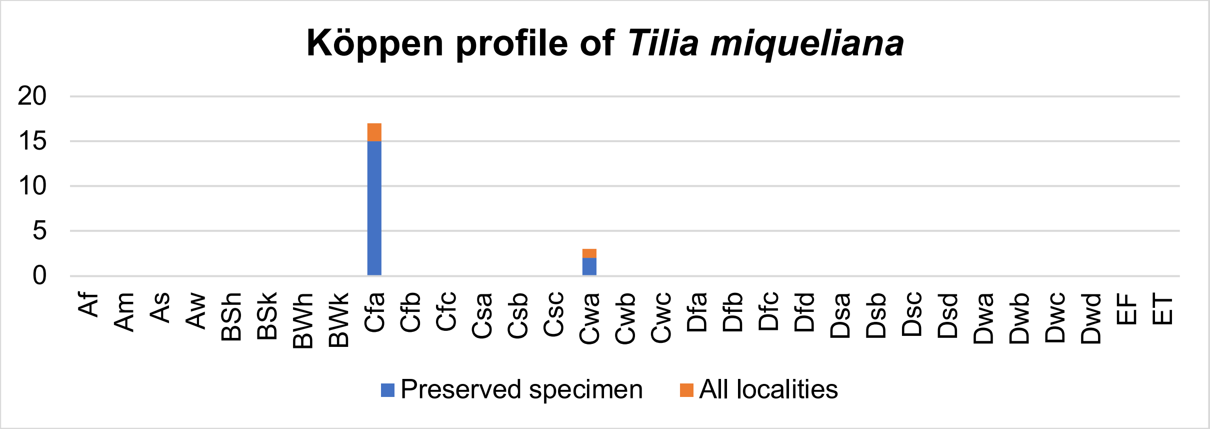

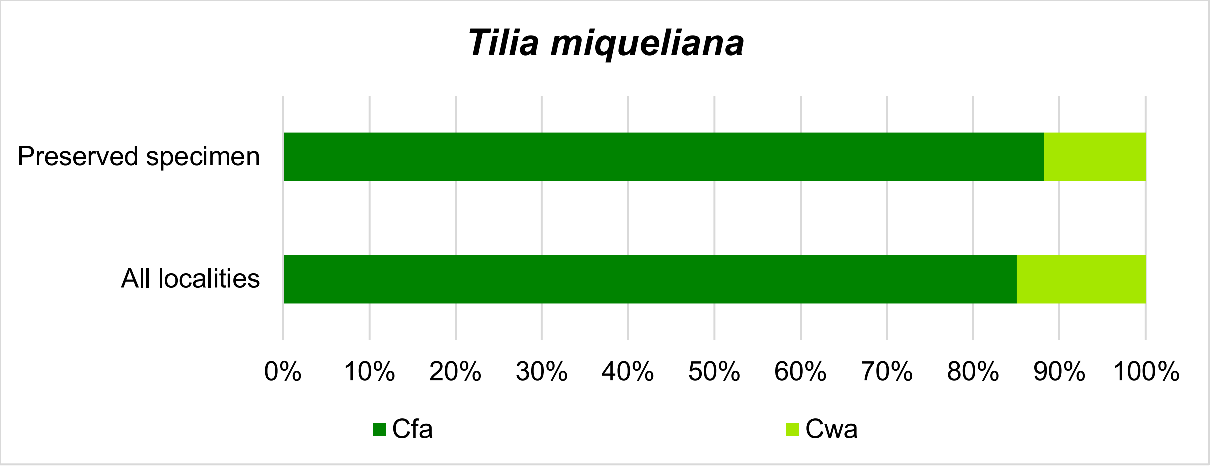

5.3.4.7.2. Biome profile, distribution, and biome map – GBIF occurrences of *Tilia miqueliana*; excluding duplicate occurrences (n = 20), herbarium specimens (n = 17).

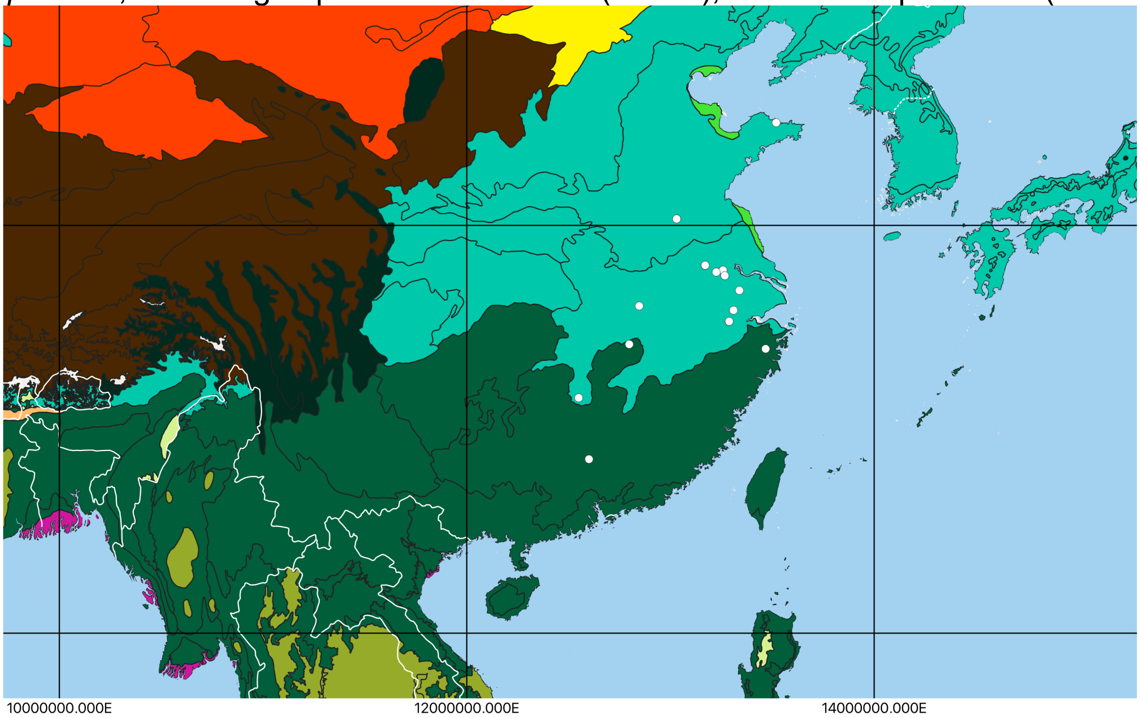

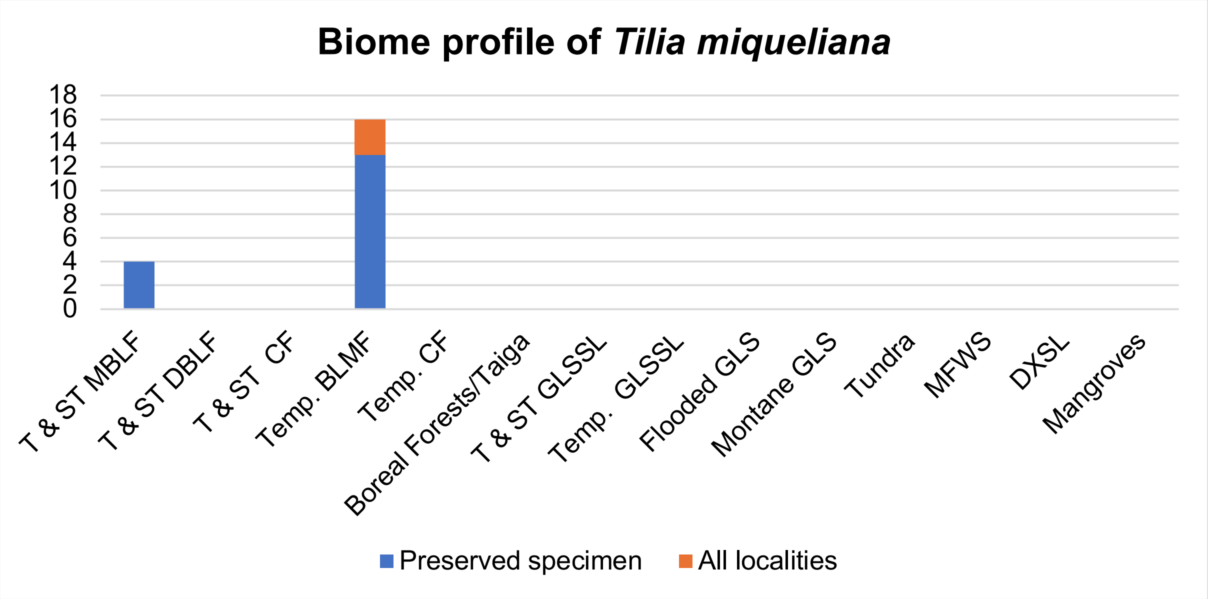

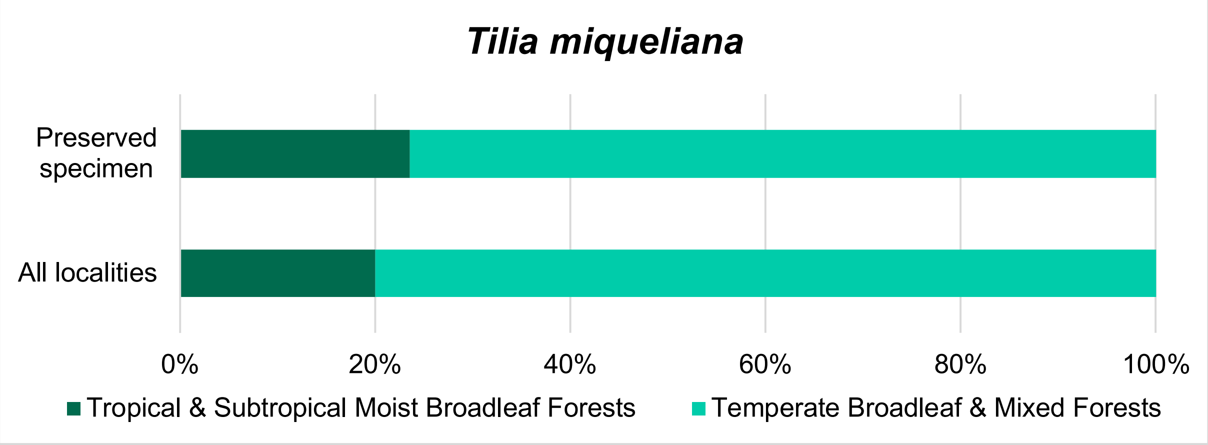

5.3.4.7.3. Climate graphs - based on 20 *Tilia miqueliana* occurrences in GBIF

5.3.4.7.3.1. MMT [°C]

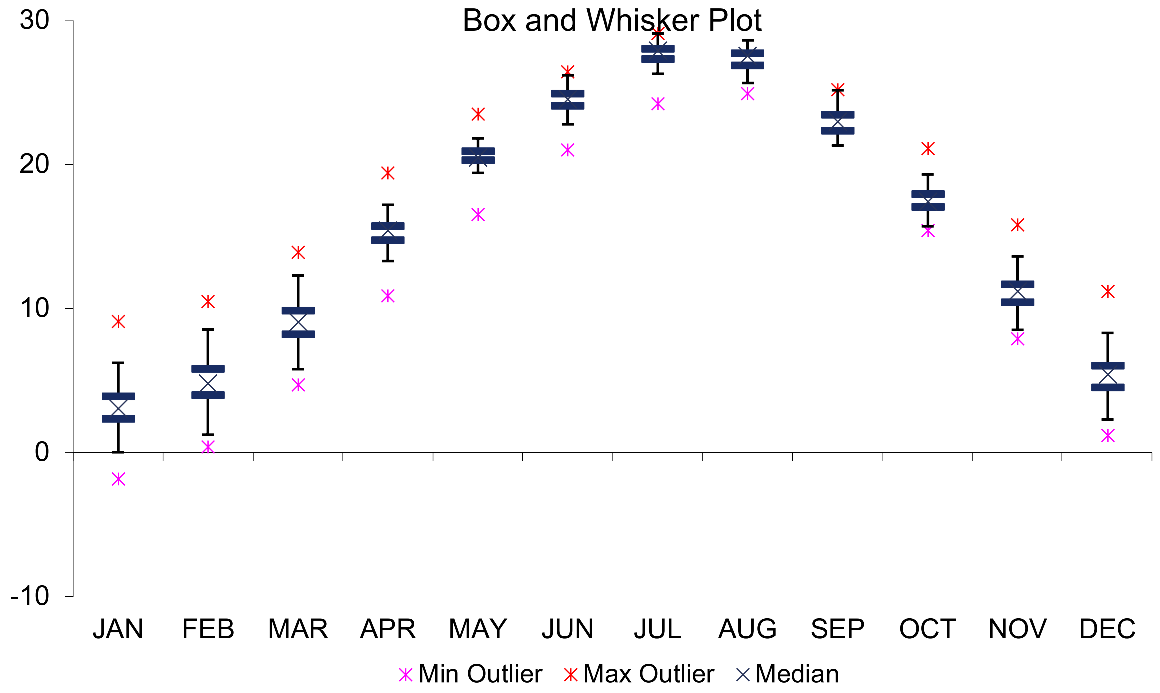

5.3.4.7.3.2. MinMT [°C]

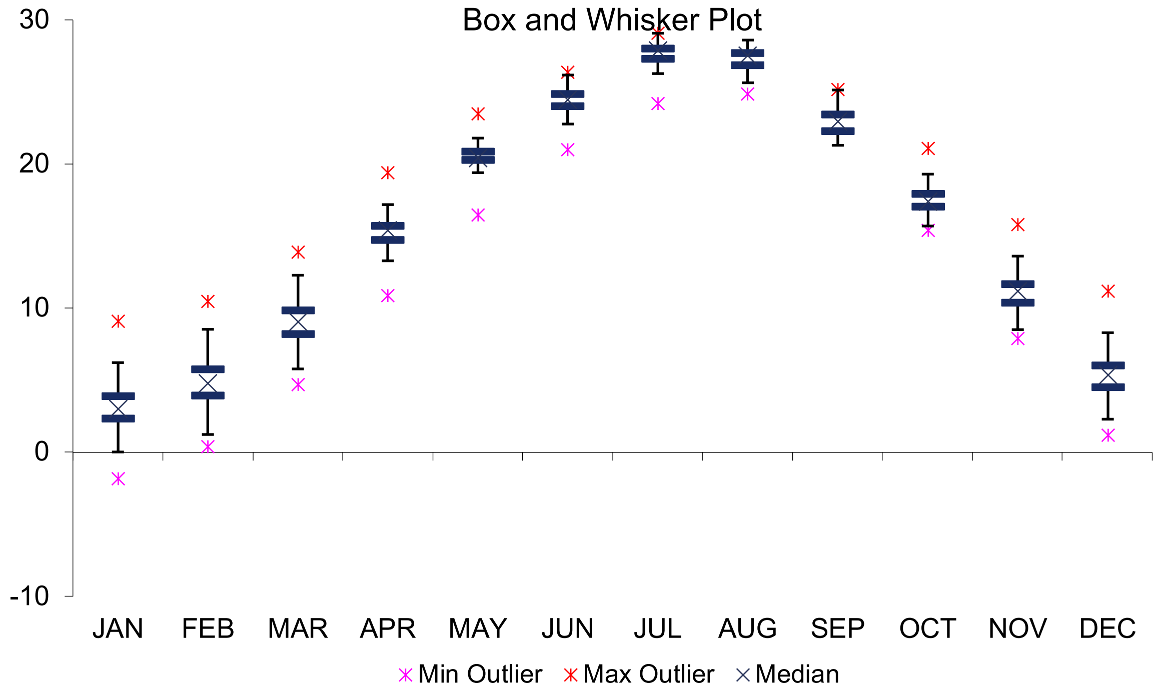

5.3.4.7.3.3. MMP [mm]

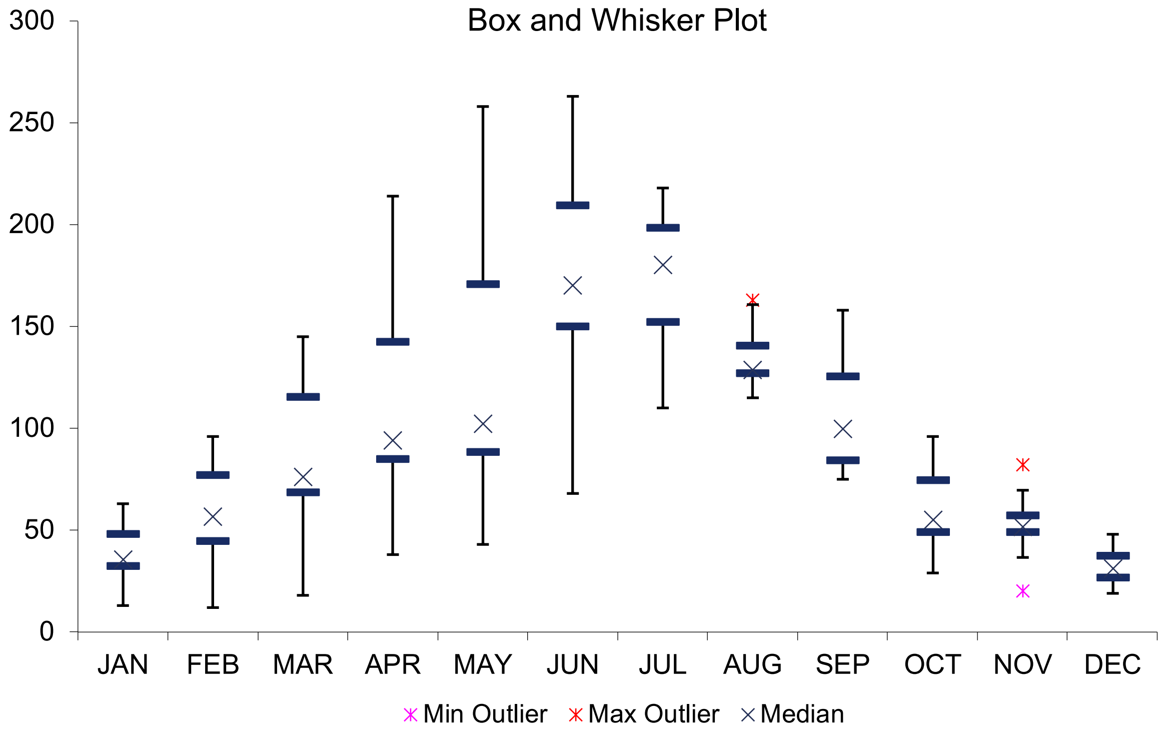

5.3.4.8. Species *Tilia nobilis* Rehder et E.H.Wilson, 1915

5.3.4.8.1. Köppen profile, distribution, and climate map – GBIF occurrences of *Tilia nobilis*; excluding duplicate occurrences herbarium specimens (n = 15).

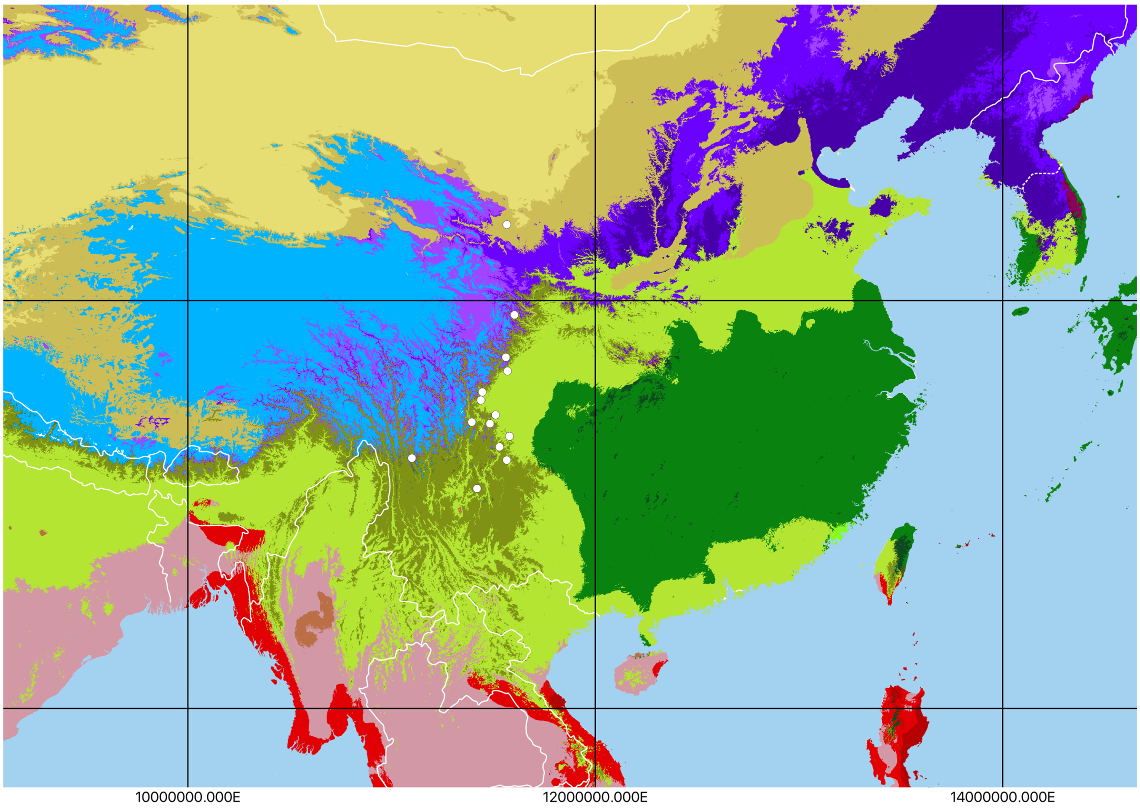

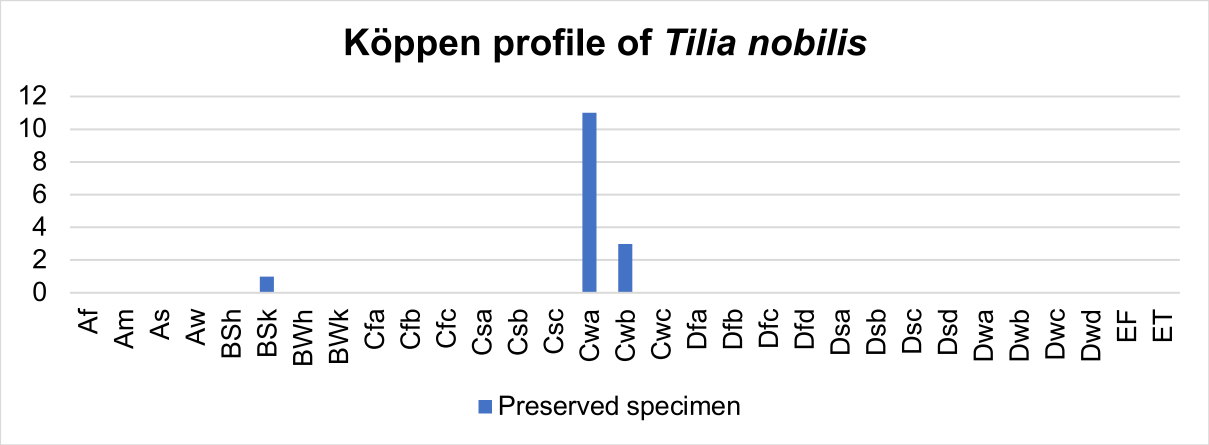

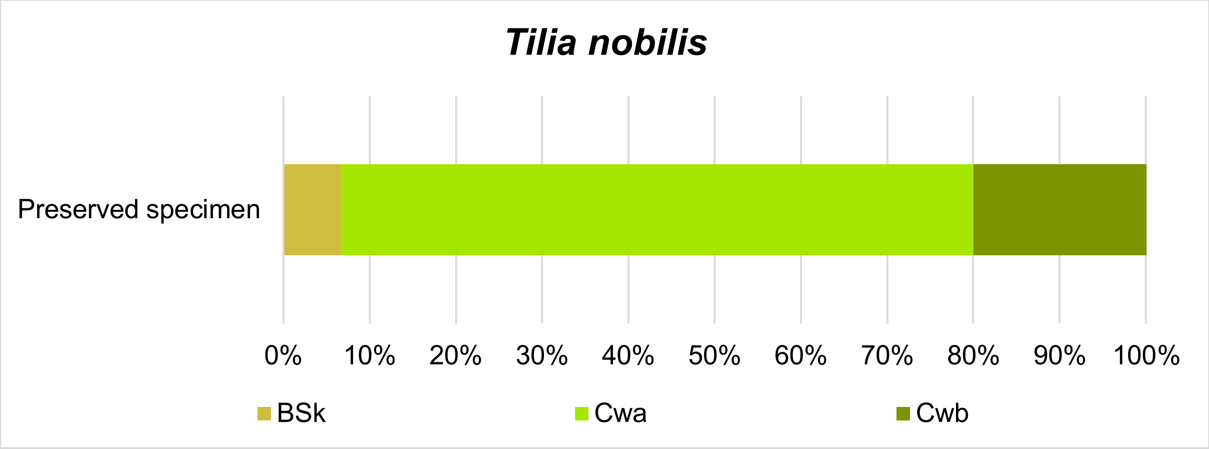

5.3.4.8.2. Biome profile, distribution, and biome map – GBIF occurrences of *Tilia nobilis*; excluding duplicate occurrences herbarium specimens (n = 15).

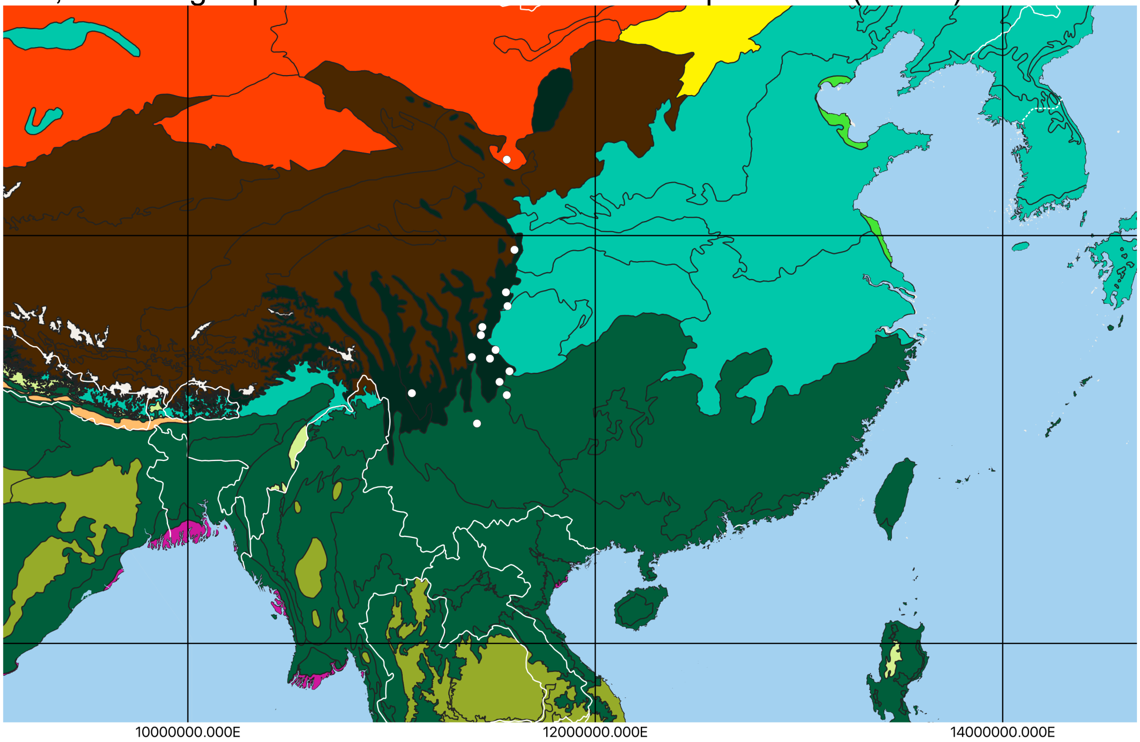

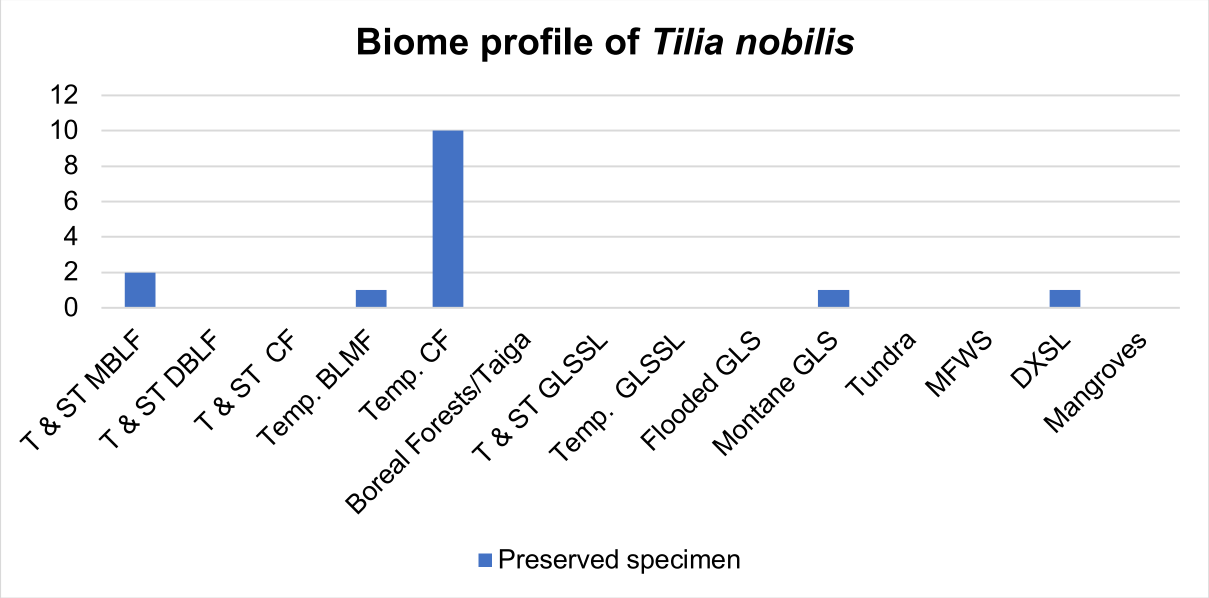

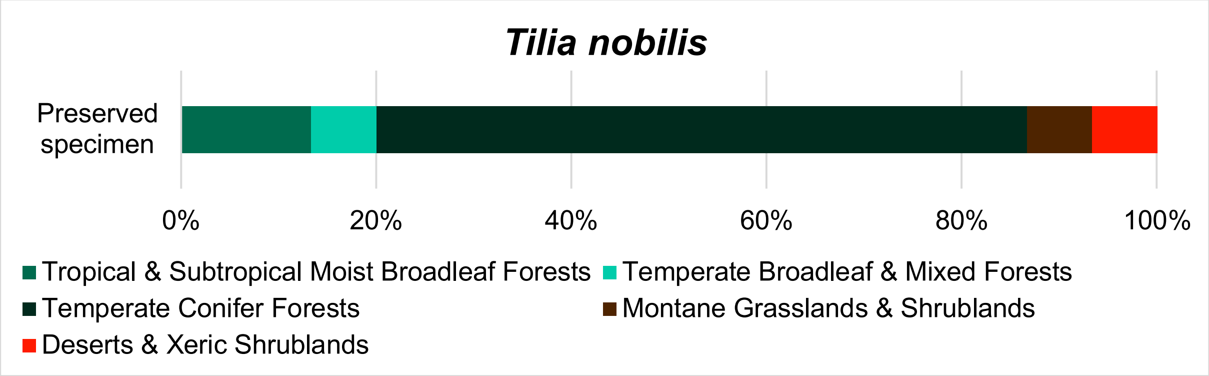

5.3.4.8.3. Climate graphs - based on 15 *Tilia nobilis* occurrences in GBIF

5.3.4.8.3.1. MMT [°C]

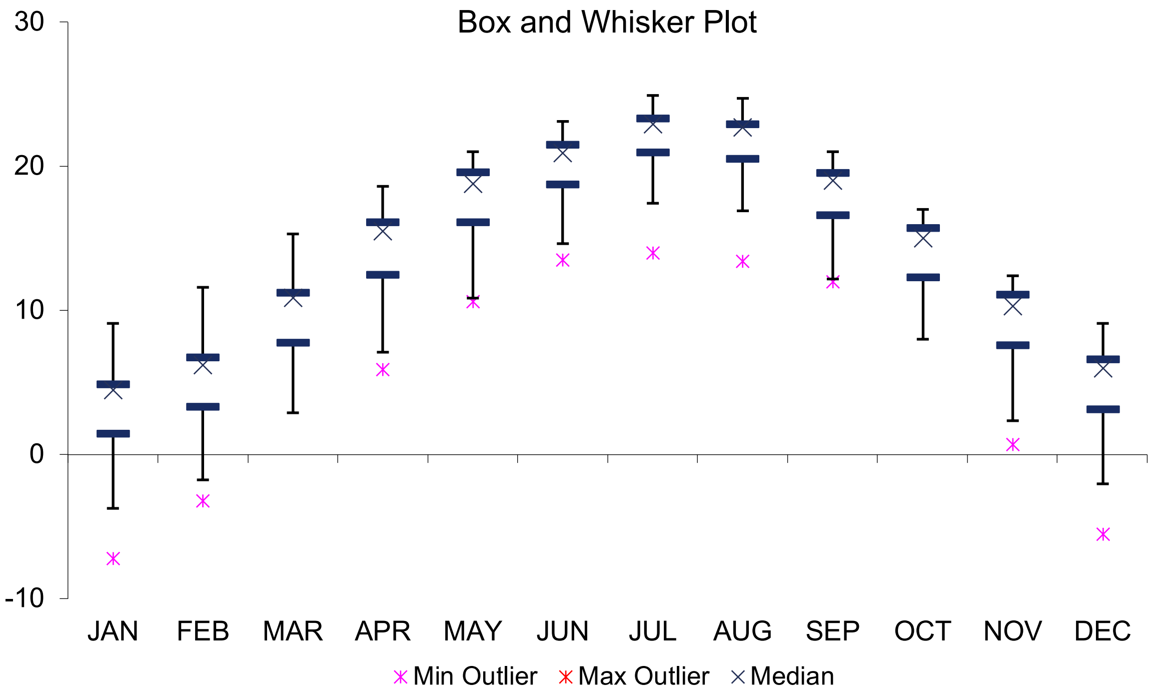

5.3.4.8.3.2. MinMT [°C]

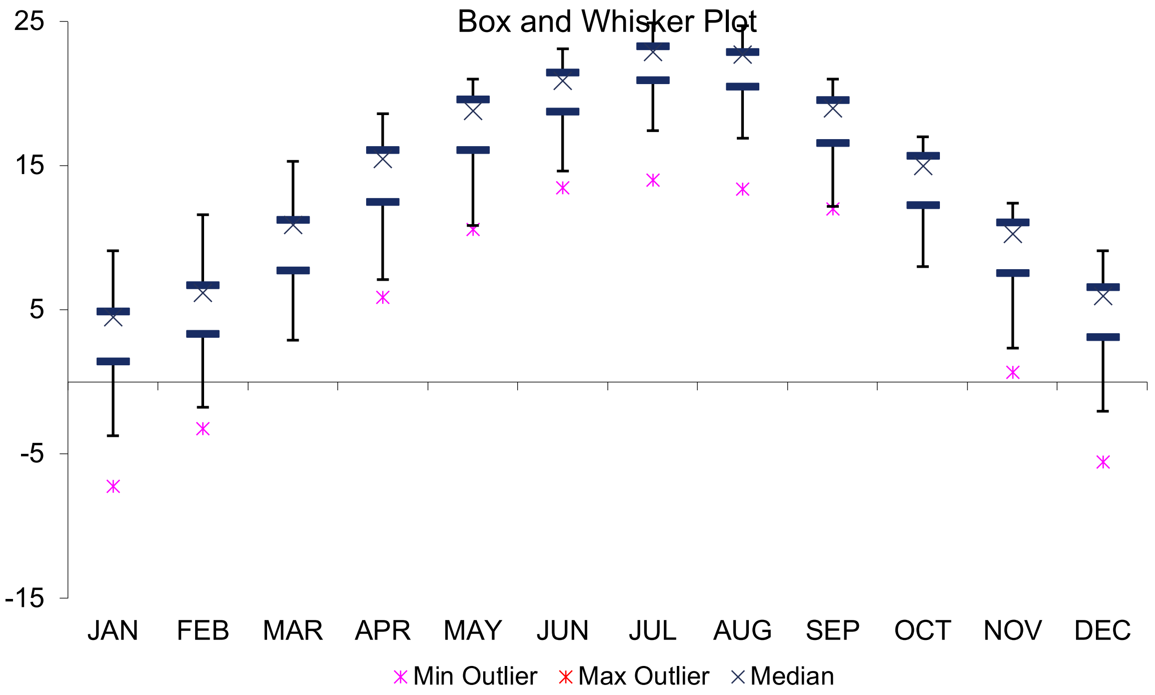

5.3.4.8.3.3. MMP [mm]

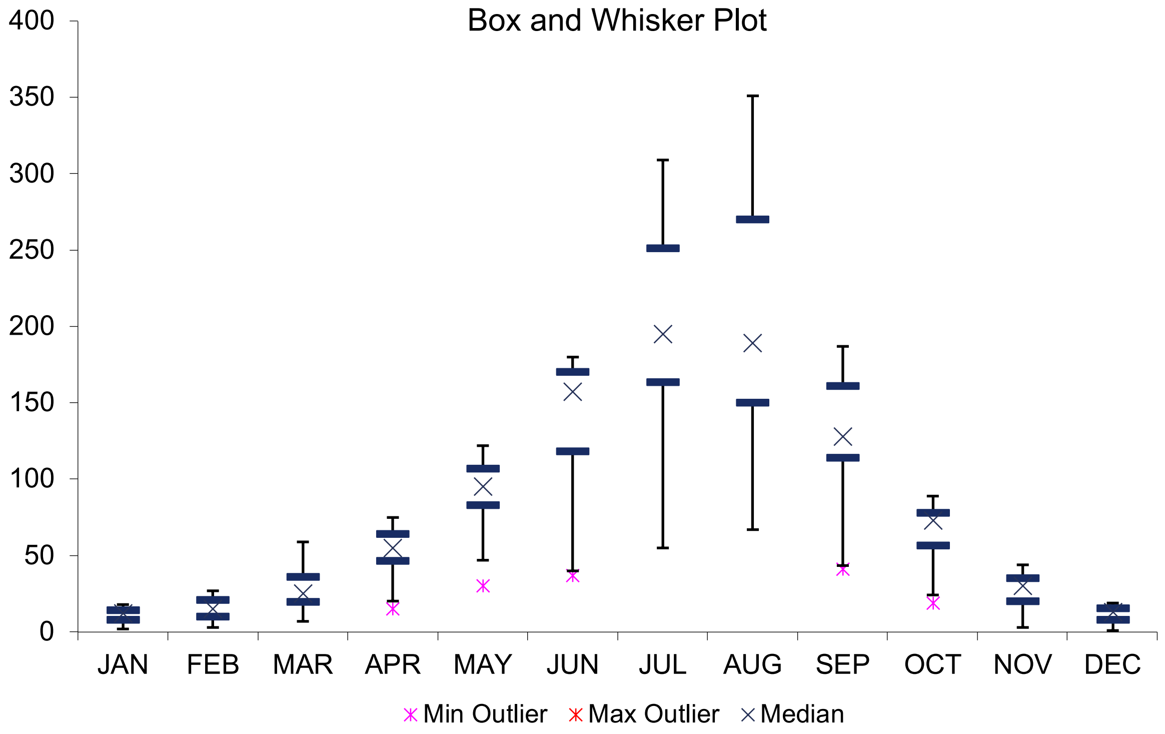

5.3.4.9. Species *Tilia oliveri* Szyszył., 1927

5.3.4.9.1. Köppen profile, distribution, and climate map – GBIF occurrences of *Tilia oliveri*; excluding duplicate occurrences herbarium specimens (n = 47).

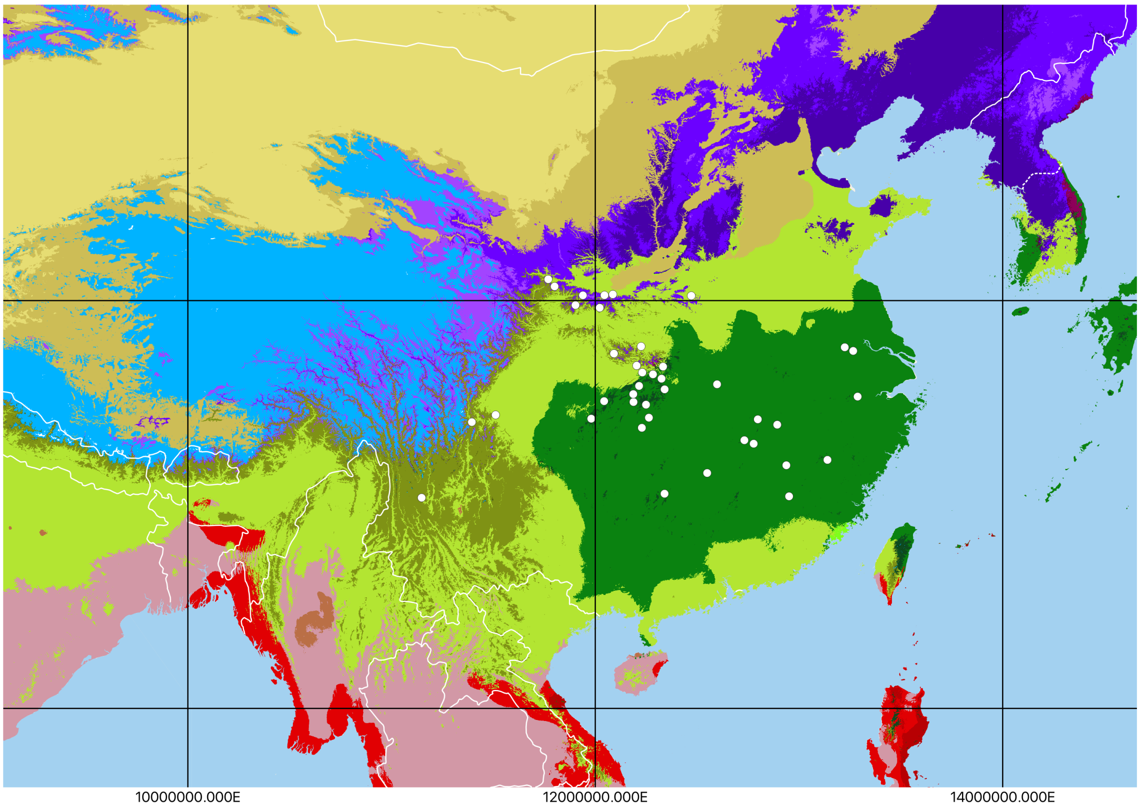

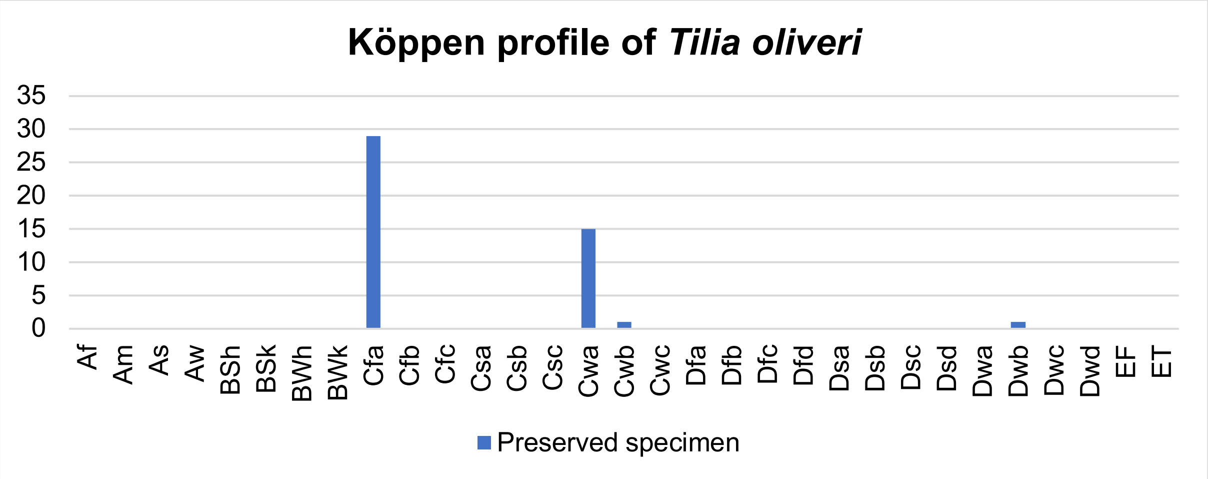

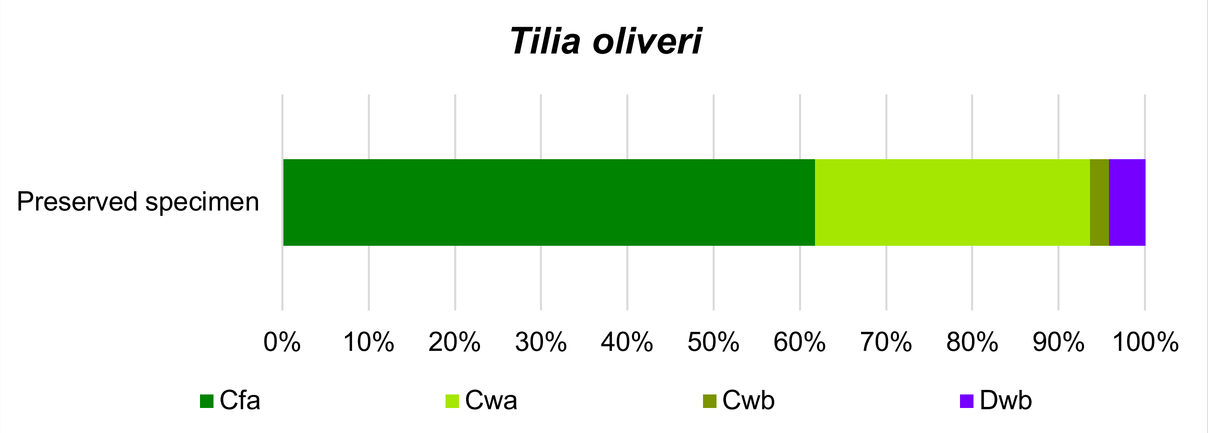

5.3.4.9.2. Biome profile, distribution, and biome map – GBIF occurrences of *Tilia oliveri*; excluding duplicate occurrences herbarium specimens (n = 47).

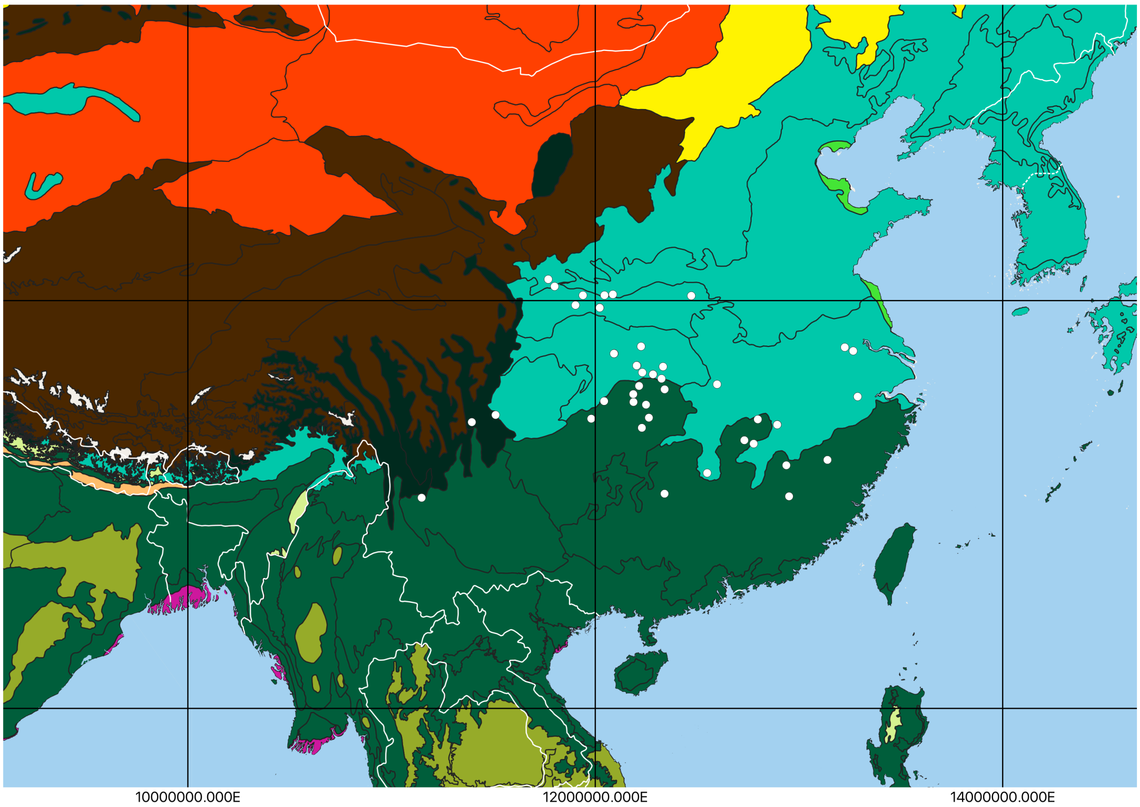

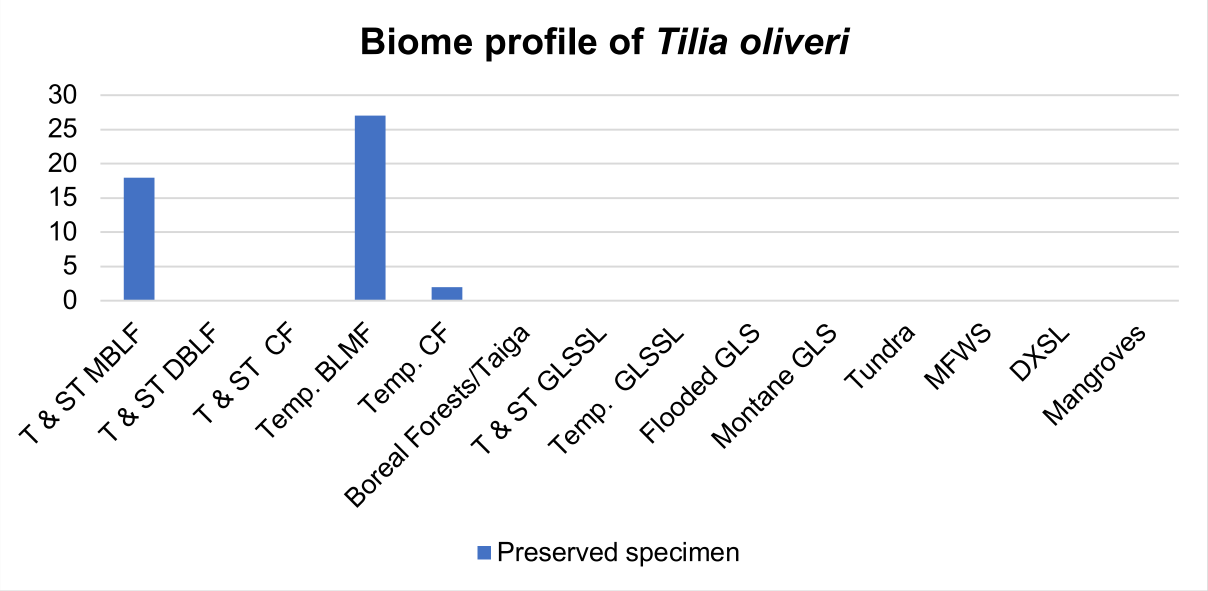

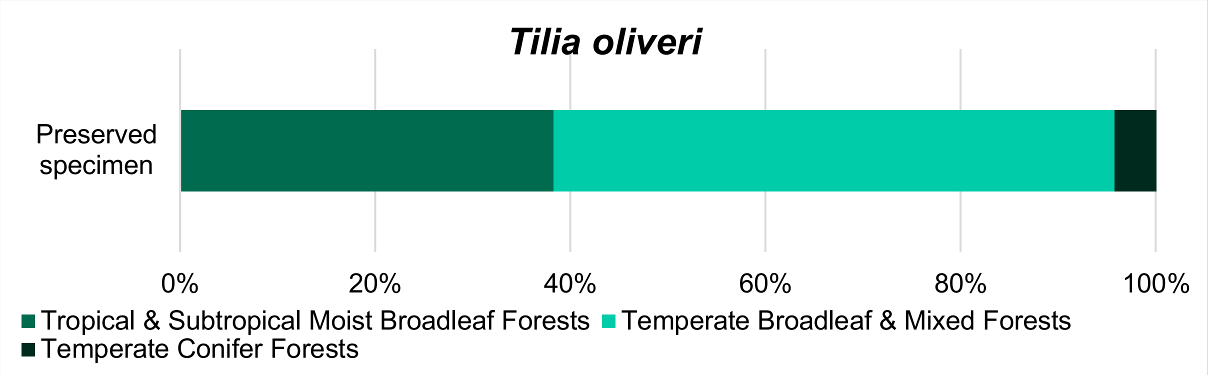

5.3.4.9.3. Climate graphs - based on 47 *Tilia oliveri* occurrences in GBIF

5.3.4.9.3.1. MMT [°C]

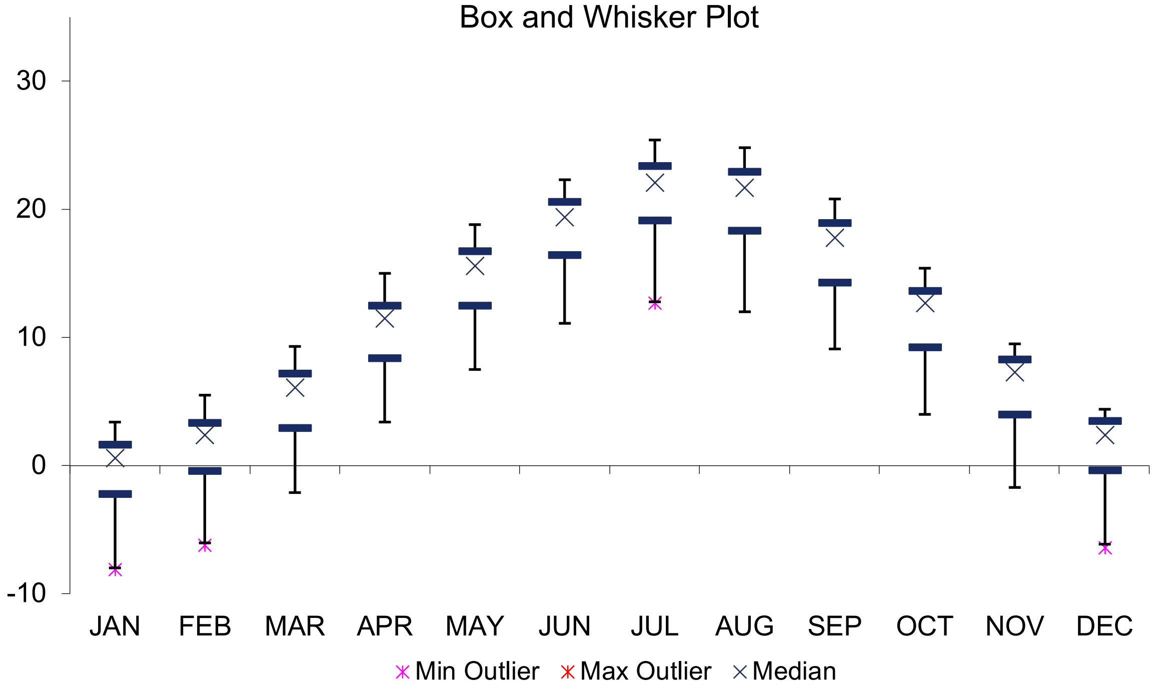

5.3.4.9.3.2. MinMT [°C]

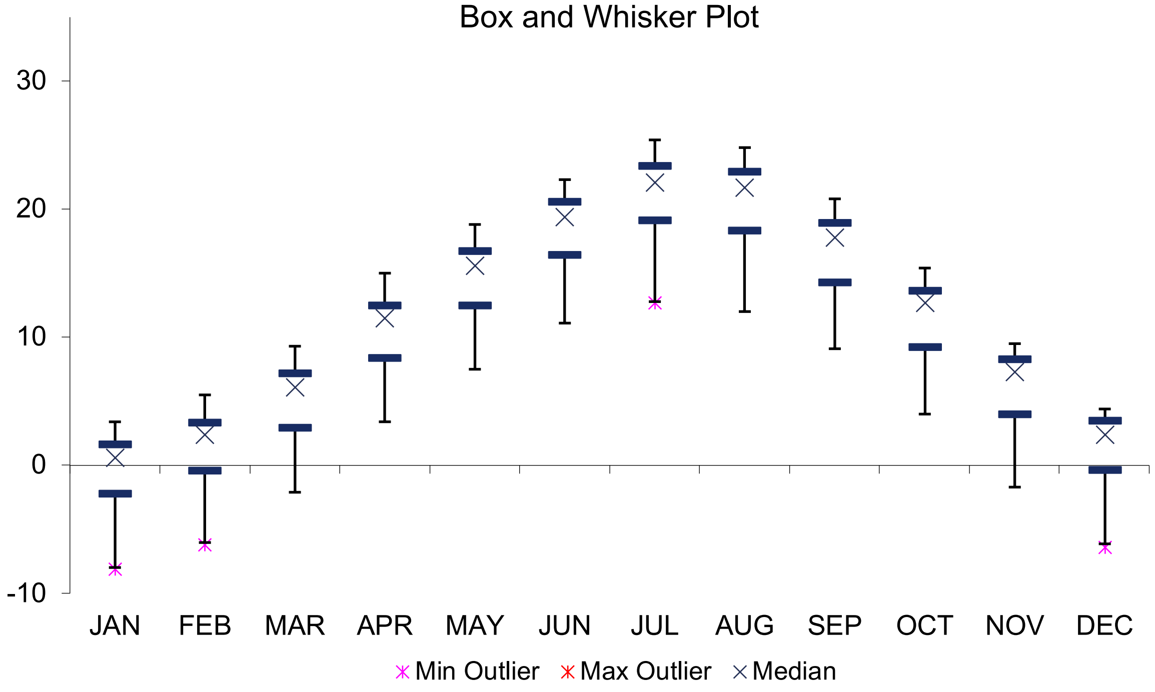

5.3.4.9.3. MMP [mm]

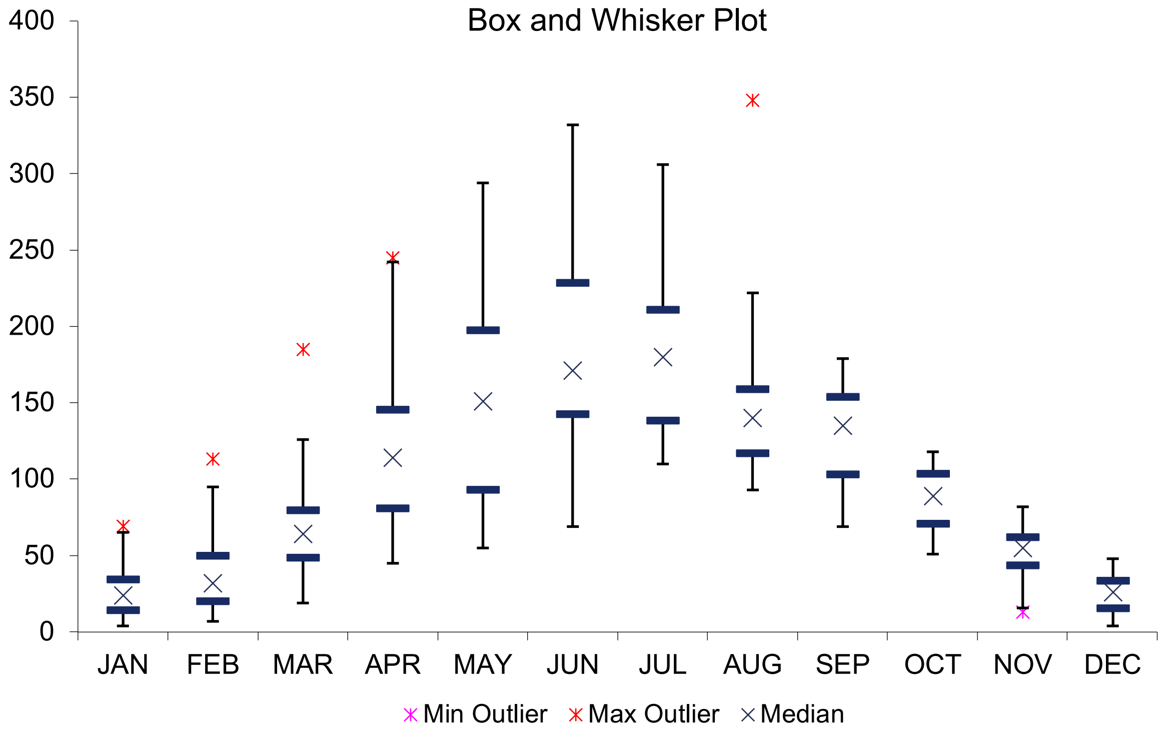

Box and Whisker Plot
400
350
300
250
200
150
100
50
0
JAN
FEB
MAR
APR
MAY
JUN
JUL
AUG
SEP
OCT
NOV
DEC
Min Outlier
Max Outlier
Median

5.3.4.10. Species *Tilia tuan* Szyszył., 1927

5.3.4.10.1. Köppen profile, distribution, and climate map – GBIF occurrences of *Tilia tuan*; excluding duplicate occurrences herbarium specimens (n = 81).

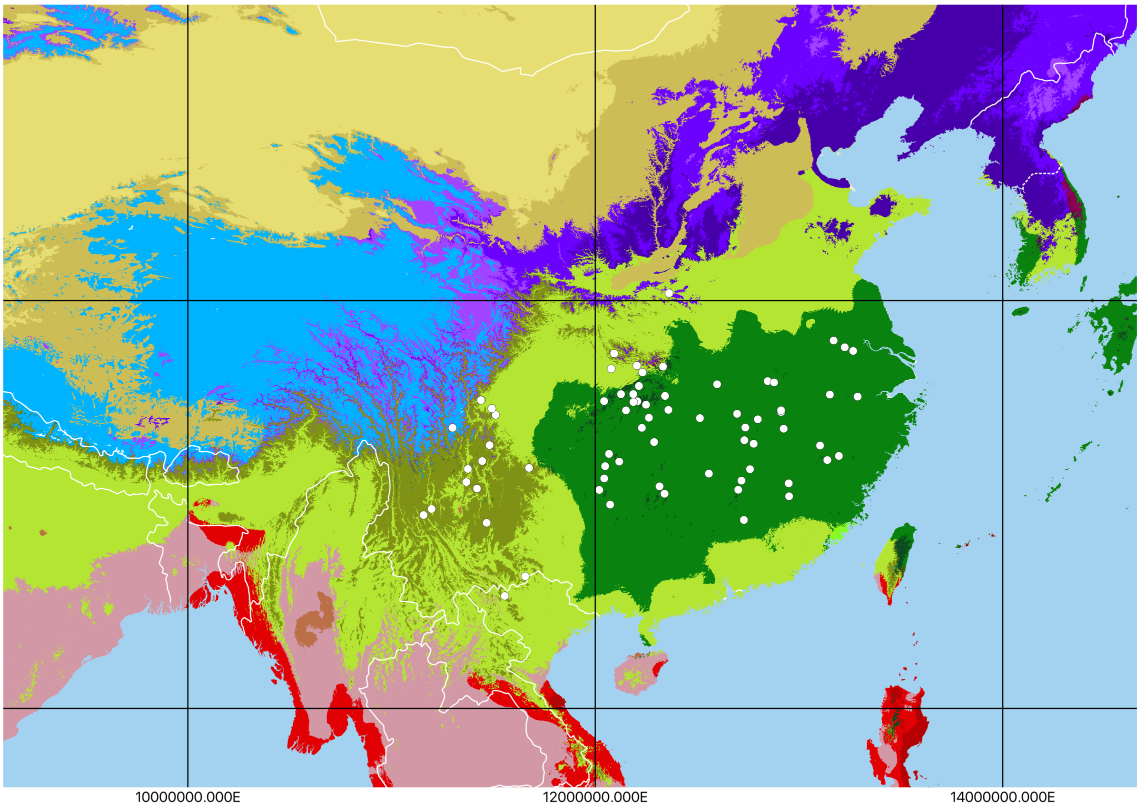

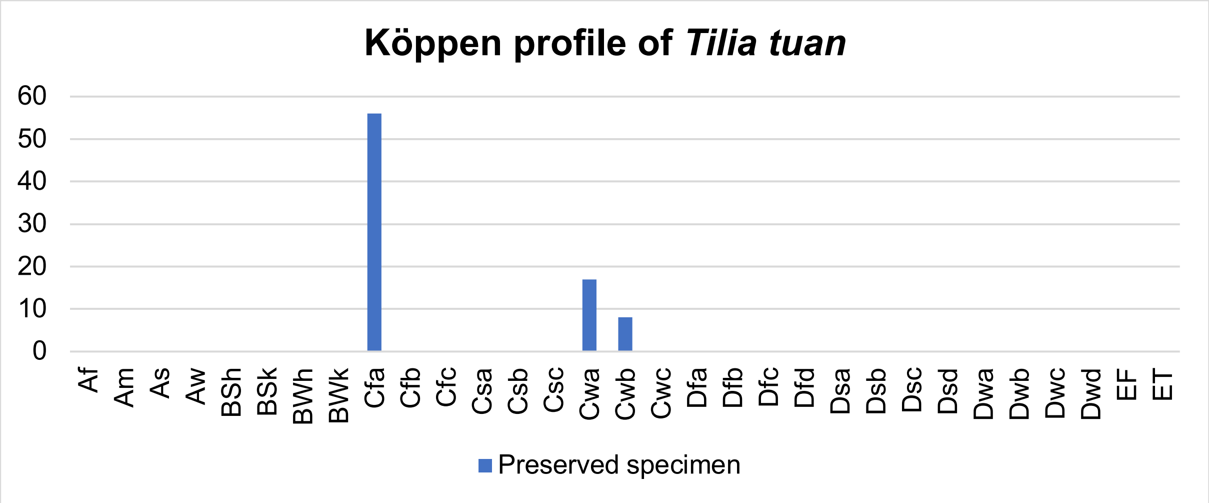

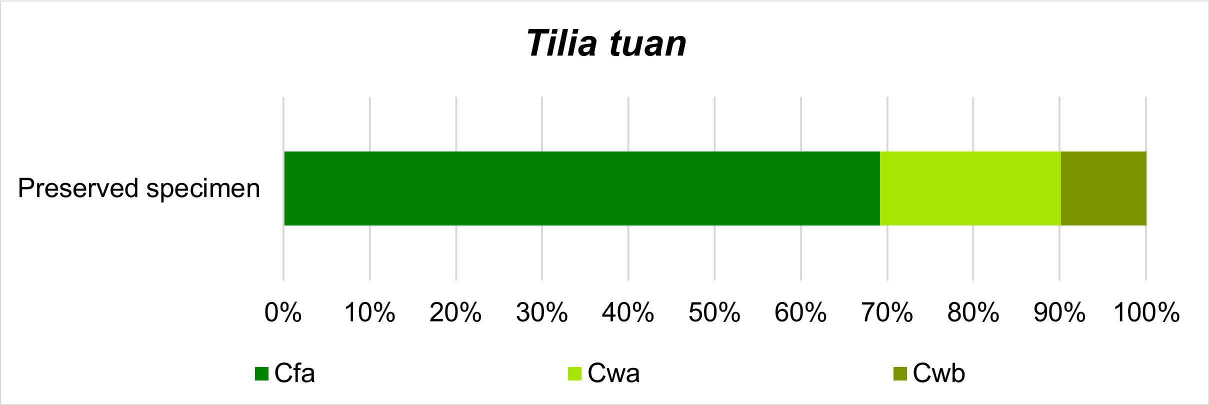

5.3.4.10.2. Biome profile, distribution, and biome map – GBIF occurrences of *Tilia tuan*; excluding duplicate occurrences herbarium specimens (n = 81).

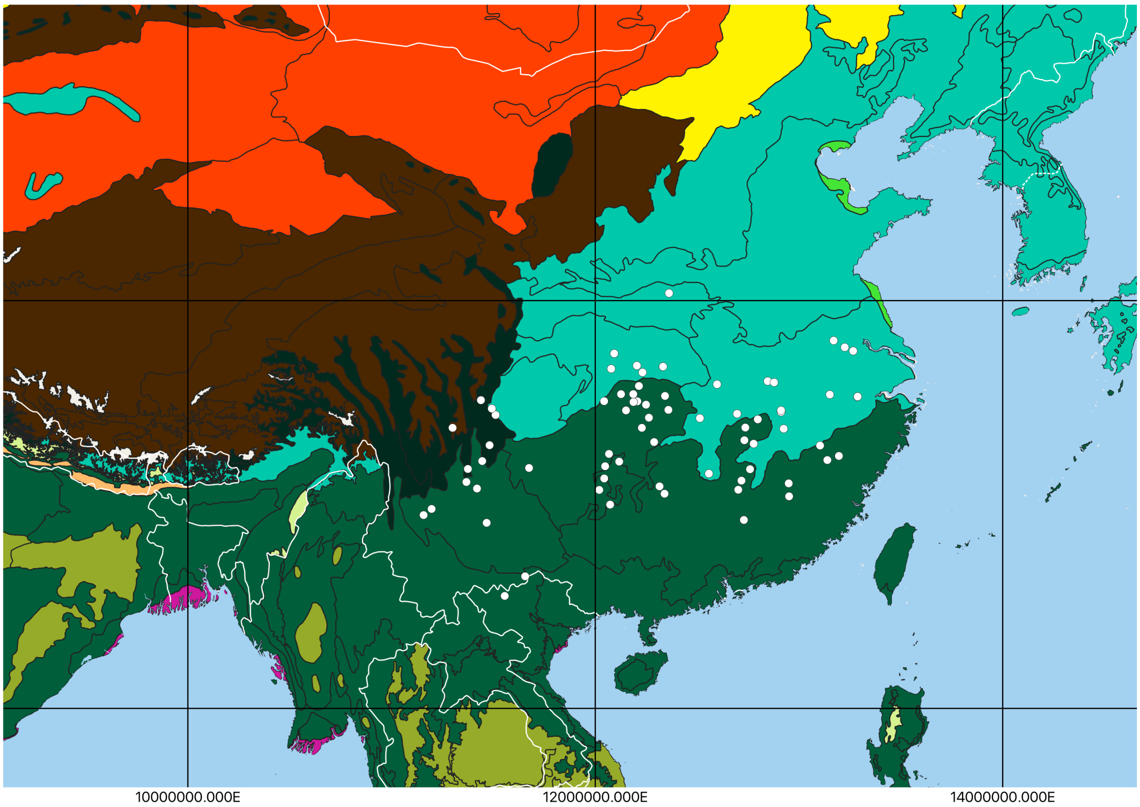

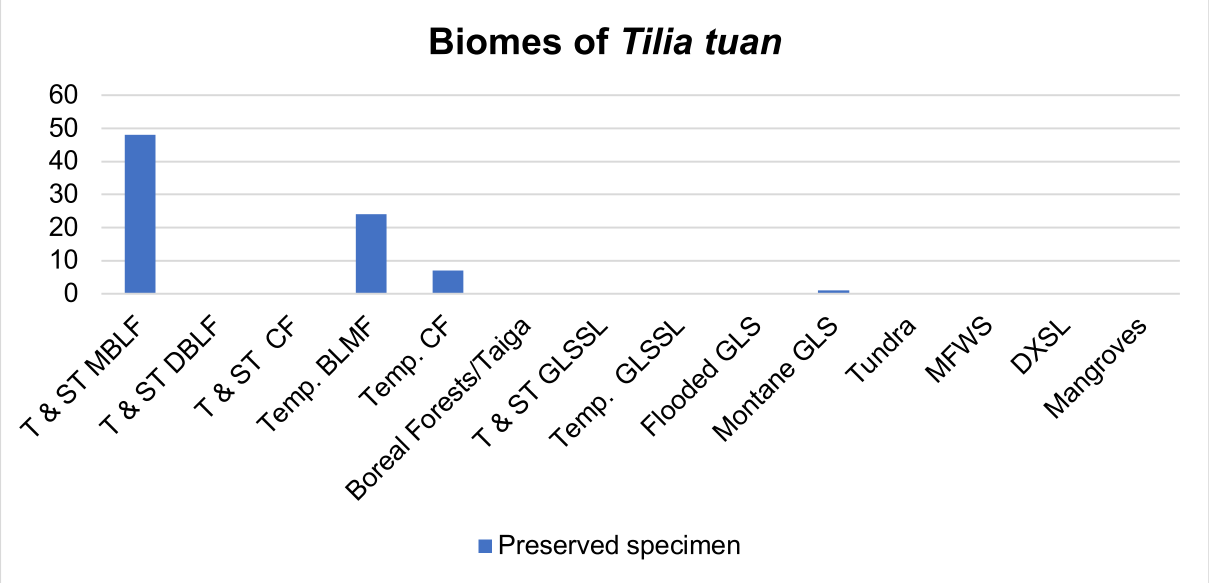

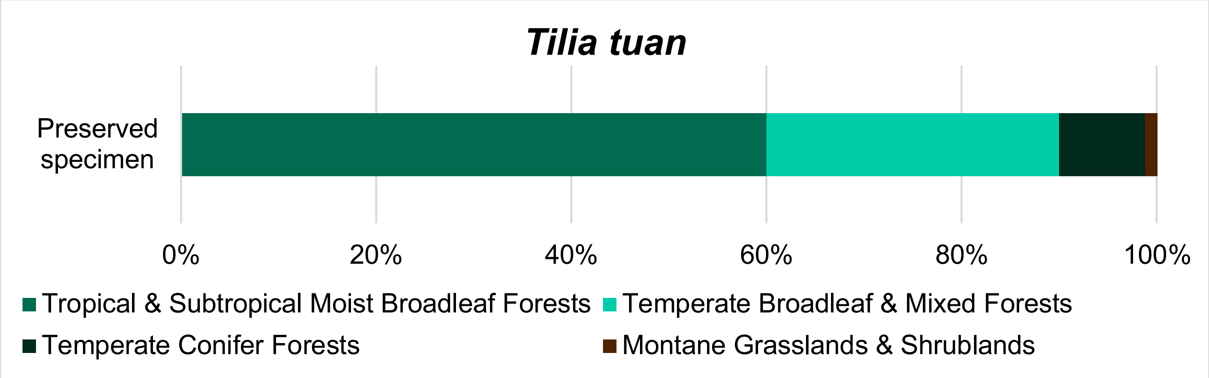

5.3.4.10.3. Climate graphs - based on 81 *Tilia tuan* occurrences in GBIF

5.3.4.10.3.1. MMT [°C]

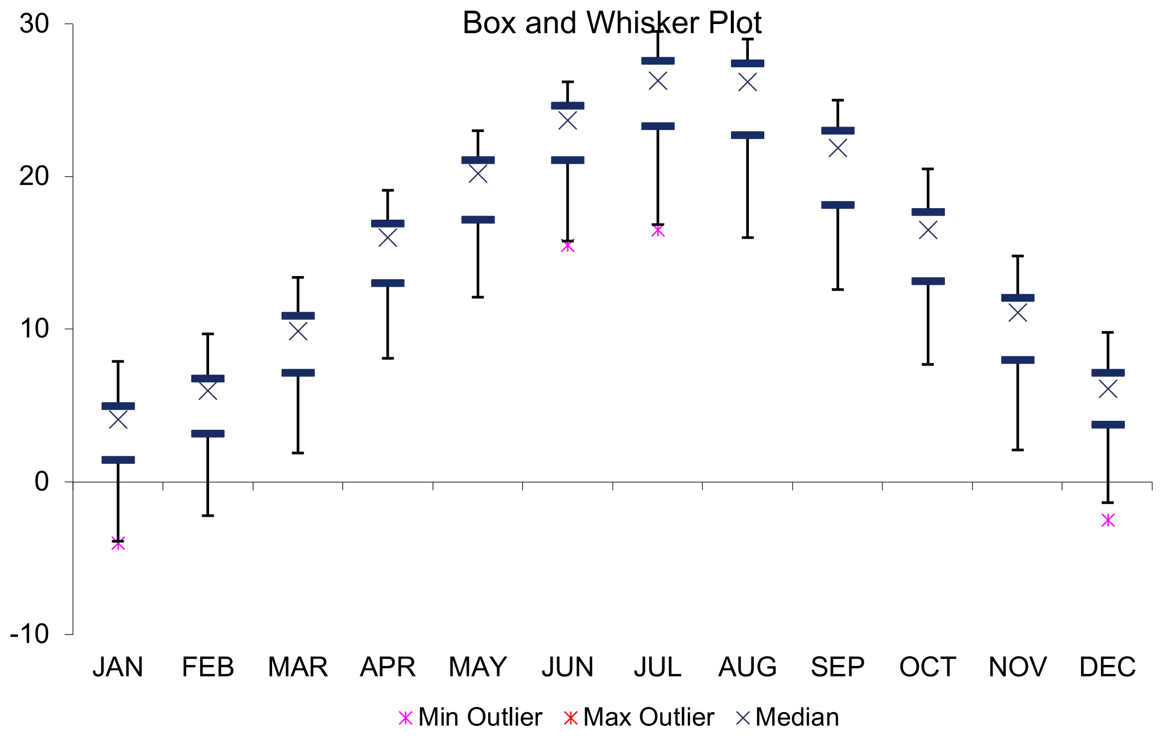

5.3.4.10.3.2. MinMT [°C]

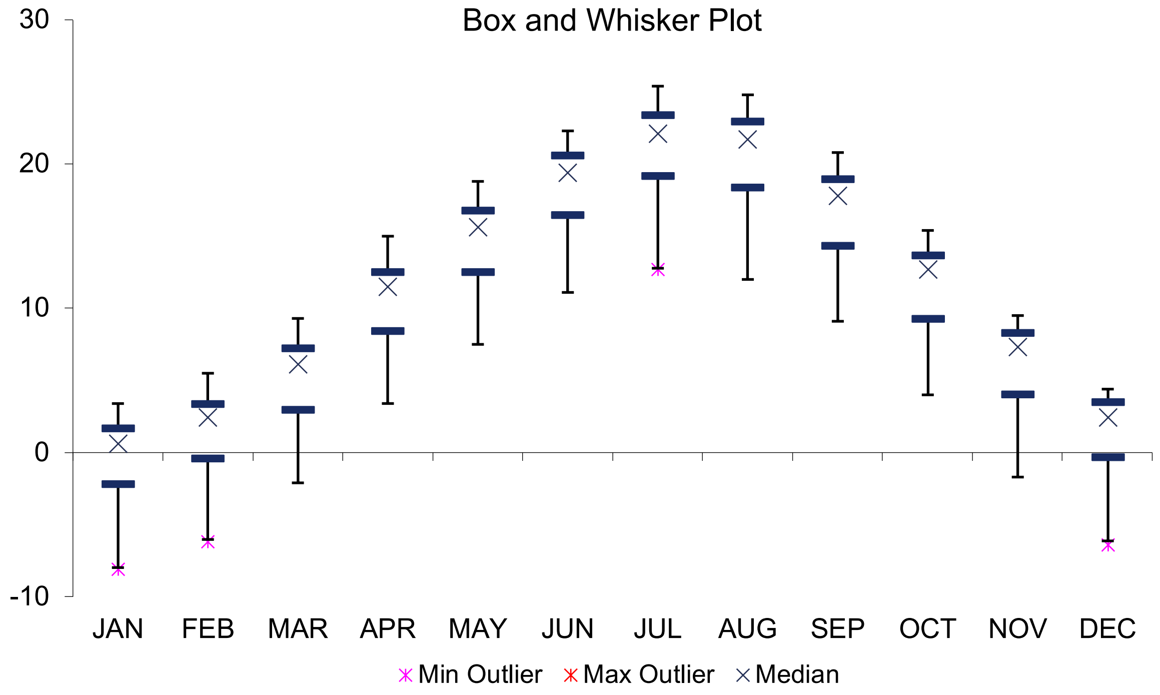

5.3.4.10.3.3. MMP [mm]

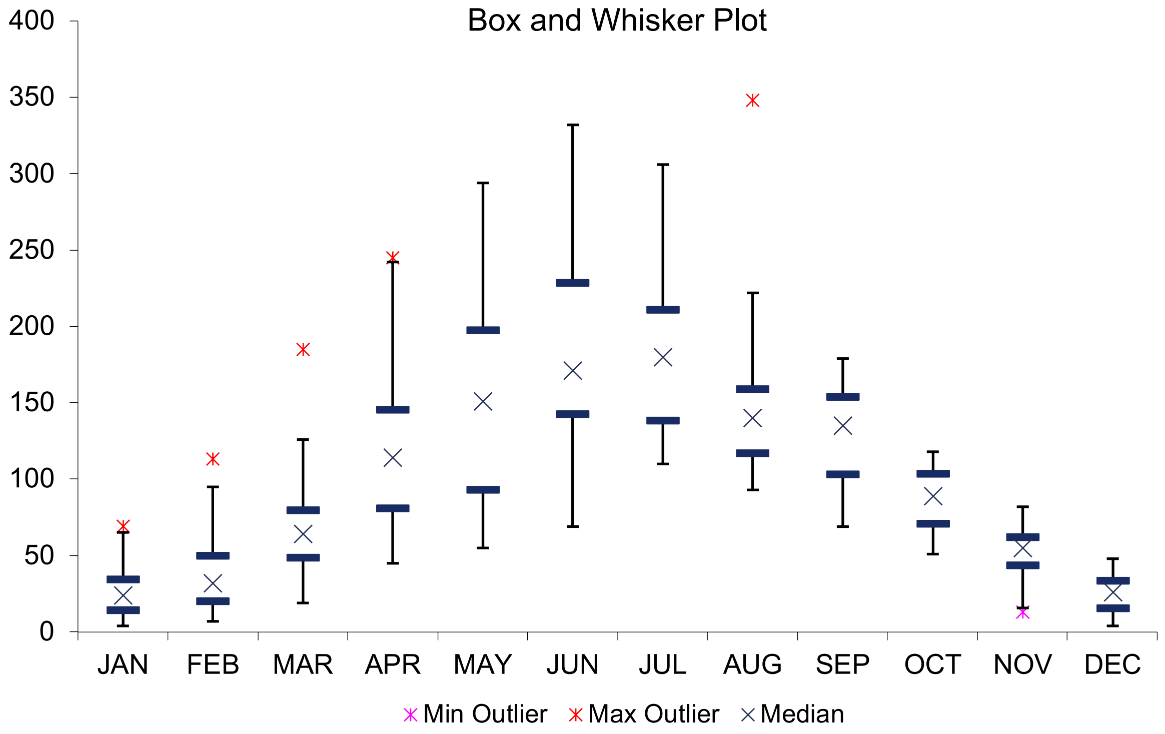

References:

Cui, D., Liang, S., Wang, D., and Liu, Z. 2021. A 1 km global dataset of historical (1979–2013) and future (2020–2100) Köppen–Geiger climate classification and bioclimatic variables. Earth Syst. Sci. Data, 13, 5087–5114. https://doi.org/10.5194/essd-13-5087-2021

Kottek, M., Grieser, J., Beck, C., Rudolf, B., and Rubel, F. 2006. World map of the Köppen-Geiger climate classification updated. Meteorol. Z., 15, 259–263.

Olson, D.M., Dinerstein, E., Wikramanayake, E.D., Burgess, N.D., Powell, G.V.N., Underwood, E.C., D'Amico, J.A., Itoua, I., Strand, H.E., Morrison, J.C., Loucks, C.L., Allnutt, T.F., Ricketts, T.H., Kura, Y., Lamoreux, J.F., Wettengel, W.W., Hedao, P., and Kassem, K.R. 2001 Terrestrial ecosystems of the world: A new map of life on Earth. *BioScience*, 51, 933-938.

Peel, M. C., Finlayson, B. L., and McMahon, T. A. 2007. Updated world map of the Köppen-Geiger climate classification, Hydrol. Earth Syst. Sci., 11, 1633–1644, https://doi.org/10.5194/hess-11-1633-2007.

Rubel, F., Brugger, K., Haslinger, K., and Auer, I. 2017. The climate of the European Alps: Shift of very high resolution Köppen- Geiger climate zones 1800–2100, Meteorol. Z., 26, 115–125, https://doi.org/10.1127/metz/2016/0816.

GBIF Datasets:

Craigia W.W.Sm. et W.E.Evans (downloaded 25 November 2024)
https://doi.org/10.15468/dl.smspt4

Mortoniodendron Standl. et Steyerm. (downloaded 25 November 2024)
https://doi.org/10.15468/dl.xuyb52

Tilia L. North and Central America (downloaded 25 November 2024)
https://doi.org/10.15468/dl.kmnb4p

Tilia L. Europe and Asiadownloaded 25 November 2024)
https://doi.org/10.15468/dl.uuskzp
