# Supplementary material for: Morphology and ultrastructure of Tilioideae pollen: how to differentiate Craigia, Mortoniodendron, and Tilia
Source: Bot Stud. 2025 Jun 9;66:15. doi: 10.1186/s40529-025-00463-1 (PMC12149084; doi:10.1186/s40529-025-00463-1)
Supplement: Supplementary file 4 — Supplementary Material 4. Pollen morphology of six Mortoniodendron species and pollen wall ultrastructure of six Tilia species. [file 40529_2025_463_MOESM4_ESM.pdf]

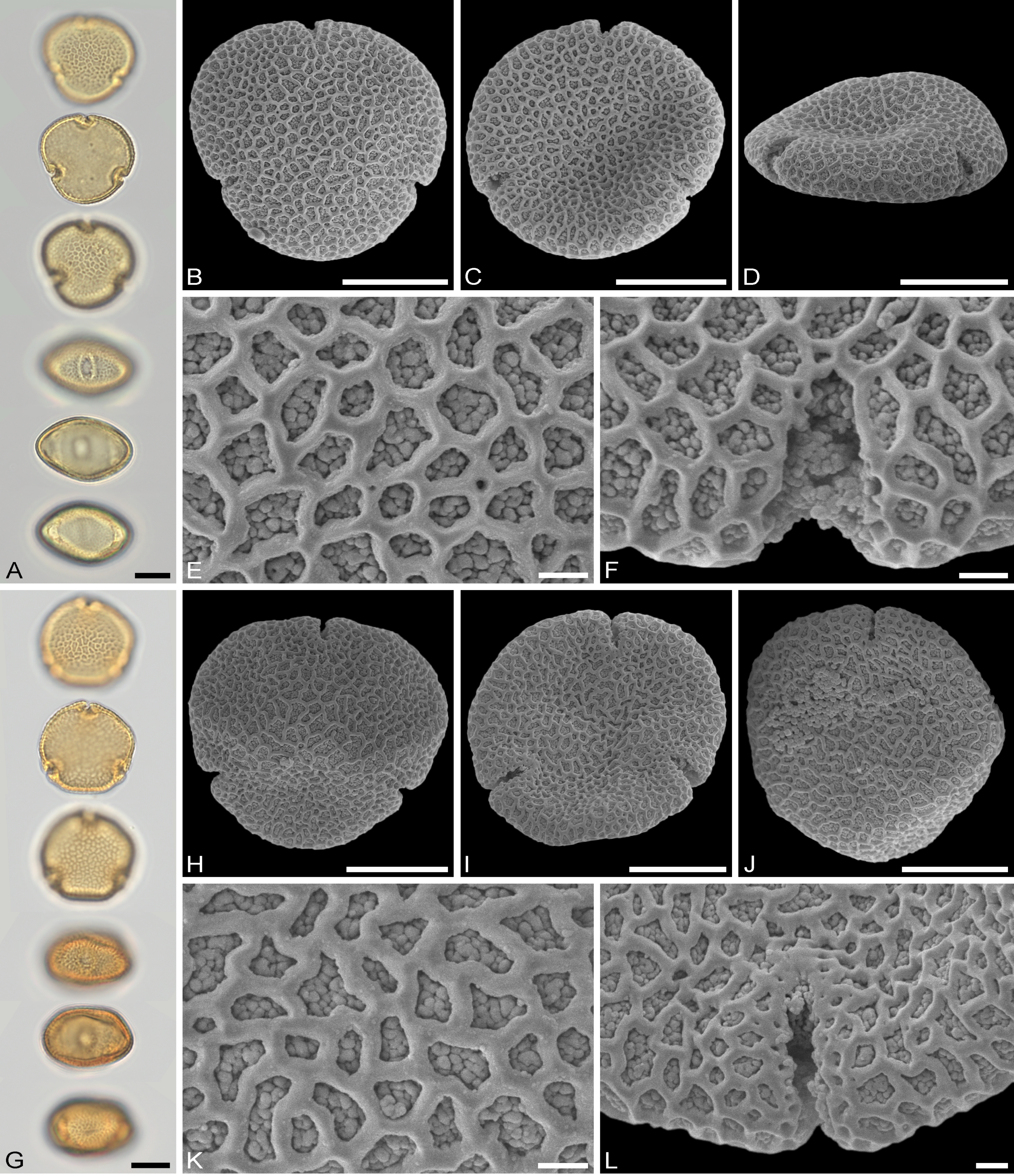

Supplementary Fig 1. Pollen morphology of *Mortoniodendron*. **A**–**F** *M. abelianum* (MO 05015040; Costa Rica). **G**–**L** *M. apetalum* (MO 6297780; Costa Rica). **A, G** LM micrographs. **B**–**D, H**–**J** SEM overview micrographs (**B, C, H**–**J** polar view; **D** equatorial view). **E, F, K, L** close-up SEM micrographs (**E, K** polar area; **F, L** aperture). Scale bars: 10 µm (**A**–**D, G**–**J**), 1 µm (**E, F, K, L**).

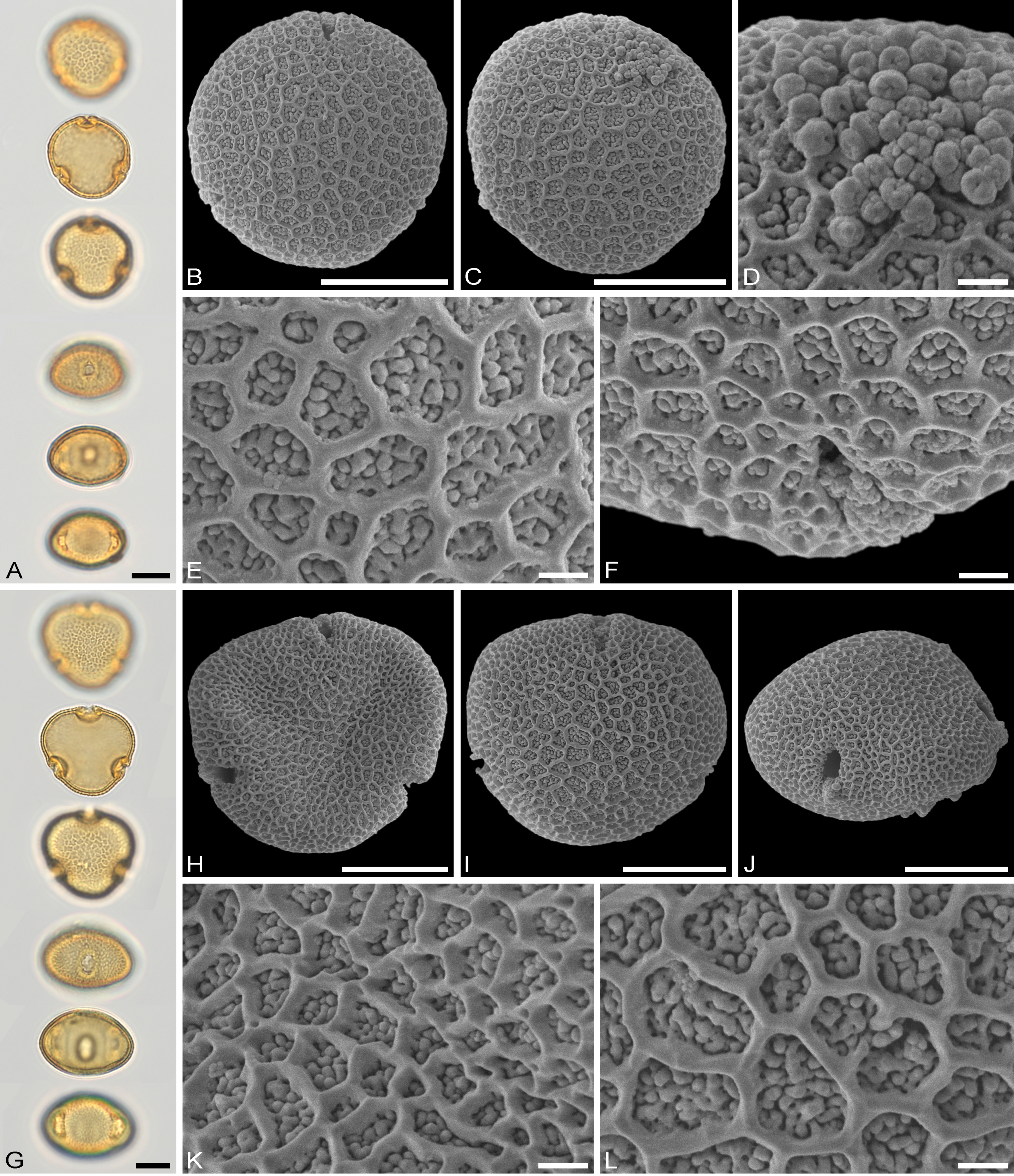

Supplementary Fig 2. Pollen morphology of *Mortoniodendron*. **A**–**F** *M. hirsutum* (MO 6066092; Panama). **G**–**L** *M. costaricense* (MO 3414249; Mexico). **A, G** LM micrographs. **B**, **C, H**–**J** SEM overview micrographs (**B, C, H, I** polar view; **H** proximal hemisphere; **I** distal hemisphere; **J** equatorial view). **D**–**F, K, L** close-up SEM micrographs (**D** Ubisch bodies on exine; **E, K** polar area; **F** aperture; **K** proximal pole; **L** distal pole). Scale bars: 10 µm (**A**–**C, G**–**J**), 1 µm (**D**–**F, K, L**).

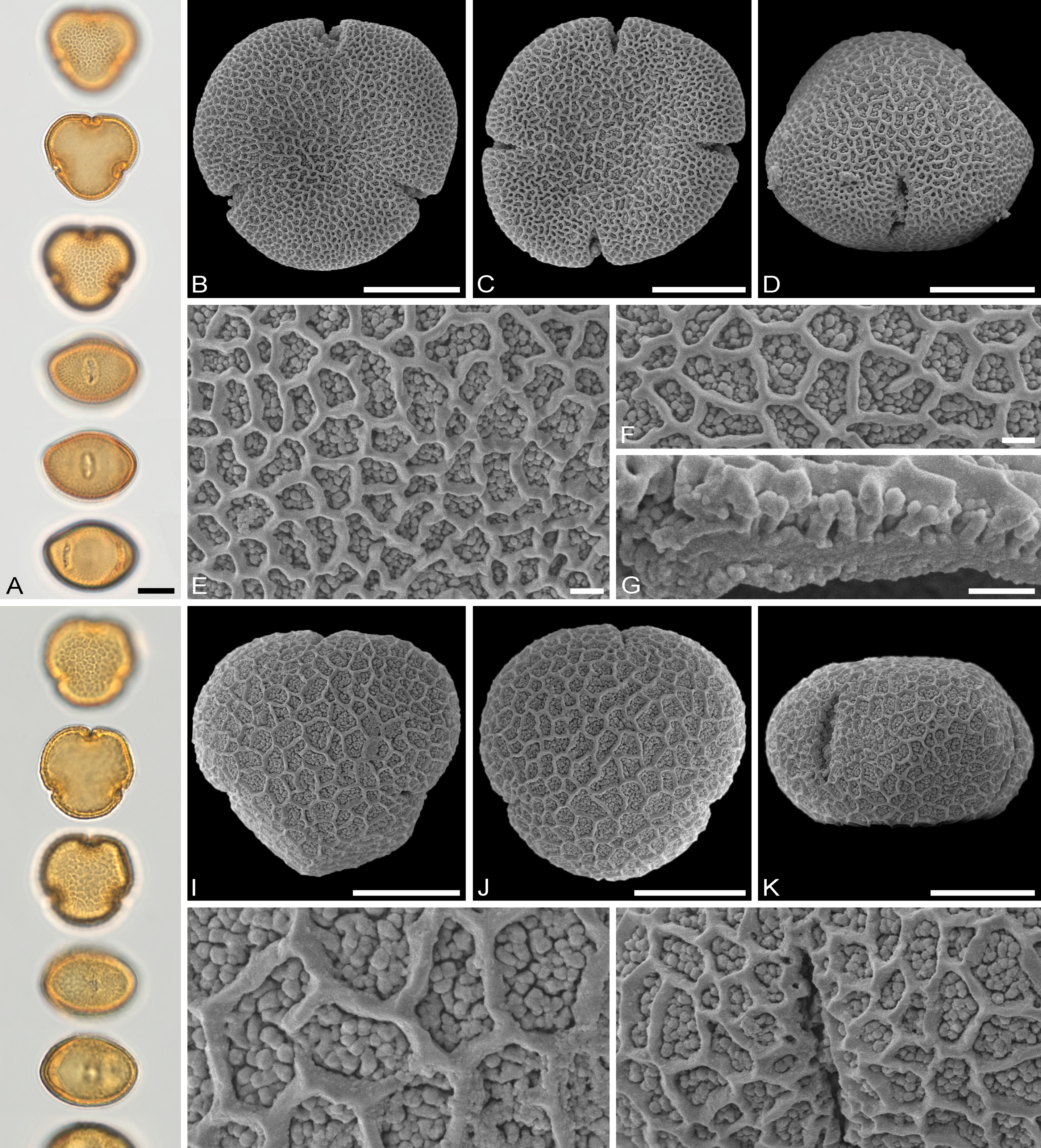

Supplementary Fig 3. Pollen morphology of *Mortoniodendron*. **A**–**G** *M. sulcatum* (MO 6709840; Mexico). **H**–**M** *M. uxpanapense* (MO 05027111; Mexico). **A, H** LM micrographs. **B**–**D, I**–**K** SEM overview micrographs (**B, C, I, J** polar view; **D, K** equatorial view). **E**–**G, L**–**M** close-up SEM micrographs (**E, F, L** polar area; **G** pollen wall fracture; **M** aperture). Scale bars: 10 µm (**A**–**D, H**–**K**), 1 µm (**E**–**G, L, M**).

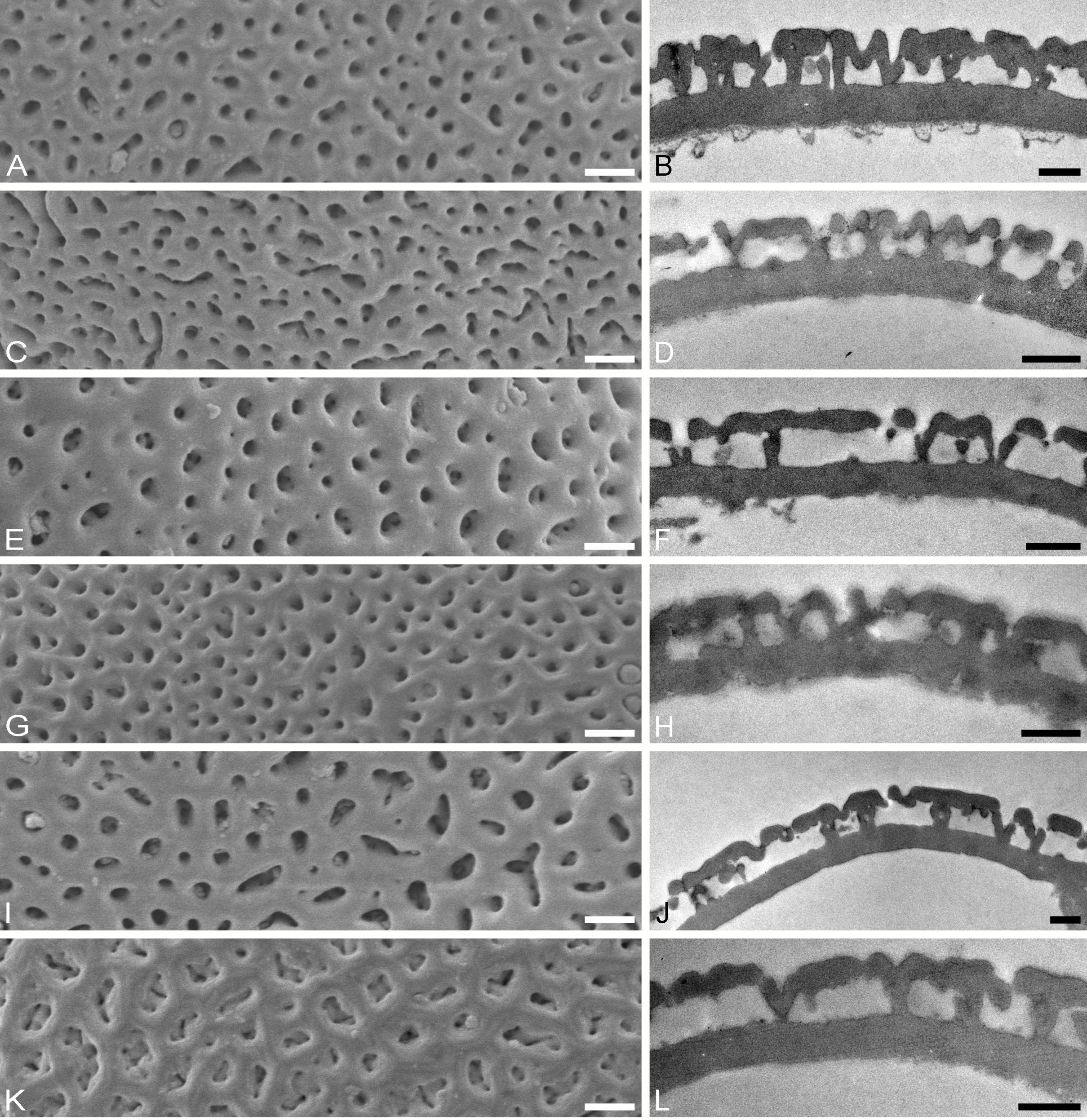

Supplementary Fig 4. Pollen wall comparison in *Tilia*. SEM micrographs (**A, C, E, G, I, K**), TEM micrographs (**B, D, F, H, J, L**). **A, B** *Tilia americana* (WU 0151993). **C, D** *T. chinensis* (WU 0151971). **E, F** *T. cordata* (WU 097095). **G, H** *T. japonica* (WU 0151997). **I, J** *T. platyphyllos* (WU 0151970). **K, L** *T. tomentosa* (WU 0151994). Scale bars: 1 µm (**A**– **L**).
